# Iron‐Catalyzed Highly Stereospecific Glycosylation with Glycal Epoxides

**DOI:** 10.1002/anie.202517634

**Published:** 2025-10-03

**Authors:** Xiao‐Wen Zhang, Le Yin, Dakang Zhang, Zixiang Jiang, Pinzhi Wang, Hao Xu

**Affiliations:** ^1^ Department of Chemistry Brandeis University 415 South Street Waltham Massachusetts 02453 USA

**Keywords:** Catalysis, Glycosylation, Iron, Oligosaccharides, Proteoglycan

## Abstract

Complex carbohydrates are essential to understanding life processes, but their synthesis is still challenging. In principle, complex glycans could be rapidly assembled via reiterative and stereospecific glycosylation with glycal epoxides. However, the existing stereospecific glycosylation methods with glycal epoxides are ineffective for the vast majority of secondary sugar acceptors, because they often induce irreversible glycal epoxide decomposition and concurrent S_N_1‐type glycosylation, affording an inseparable mixture of diastereomeric glycosylation products in low yields. We report herein a new catalytic, highly stereospecific glycosylation method for glycal epoxides using readily available iron catalysts. This method is effective for a wide variety of glycal epoxides and glycosyl acceptors, including previously challenging, sterically hindered secondary acceptors and electron‐deficient glucuronic ester epoxides. It also facilitates the assembly of an array of biologically important glycosidic linkages that were previously difficult to obtain in high stereoselectivity. Kinetic studies revealed that this iron‐catalyzed glycosylation proceeds through S_N_2‐type pathways with both primary and hindered secondary acceptors.

## Graphical abstract

We report an iron‐catalyzed, highly stereospecific glycosylation method with glycal epoxides, which is effective for a broad range of substrates, including previously challenging, sterically hindered secondary acceptors and glucuronic ester epoxide donors. Kinetic studies revealed that this iron‐catalyzed glycosylation proceeds through S_N_2‐type pathways with hindered secondary acceptors.

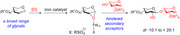

## Introduction

**Figure 1:**
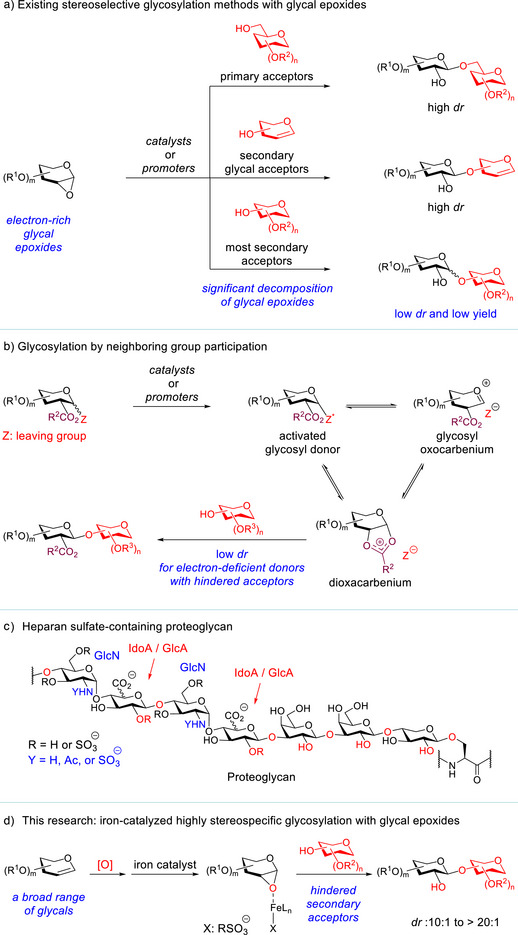
a) Existing stereoselective glycosylation methods with glycal epoxides. b) Glycosylation by neighboring group participation. c) Proteoglycan structure. d) Iron‐catalyzed highly stereospecific glycosylation with glycal epoxides.

Complex carbohydrates play an essential role in numerous life processes, but it is still difficult to obtain these valuable molecules in high homogeneity and sufficient quantities through synthesis. Although a glycal epoxide (1,2‐anhydrosugar) is not involved in polysaccharide biosynthesis,^[^ ^1^ ^]^ complex glycans could in principle get assembled via reiterative and stereospecific glycosylation of glycal epoxides. The pioneering studies by Schuerch^[^ ^2^ ^]^ and Danishefsky^[^ ^3^, ^4^, ^5^ ^]^ demonstrated the power of this approach for complex‐glycan assembly. These seminal contributions also inspired the development of an array of valuable glycosylation methods, which add to the repertoire for complex‐carbohydrate synthesis.^[^ ^6^, ^7^, ^8^, ^9^, ^10^, ^11^, ^12^, ^13^, ^14^, ^15^, ^16^, ^17^ ^]^ These reported stereospecific glycosylation methods with glycal epoxides are *effective for primary acceptors and secondary glycal acceptors* (Figure 1a).^[^ ^3^, ^4^, ^5^, ^6^, ^7^, ^8^, ^9^, ^13^, ^14^, ^15^, ^16^, ^17^, ^18^, ^19^ ^]^ However, they are *ineffective for the vast majority of secondary sugar acceptors that are more sterically hindered*, because the catalysts and promoters used in these methods do not induce stereospecific, S_N_2‐type glycosylation of a glycal epoxide with a hindered secondary acceptor. Instead, they often mediate irreversible glycal‐epoxide ring‐opening and decomposition with concurrent S_N_1‐type glycosylation, affording a diastereomeric mixture in low yields.^[^ ^9^, ^20^ ^]^ Additionally, these methods are predominantly tailored for electron‐rich glycal epoxides but they are *ineffective for electron‐deficient, glucuronic ester epoxides* ^[^ ^21^ ^]^ that are valuable building blocks for glycosaminoglycan (GAG) synthesis.^[^ ^22^ ^]^ Therefore, a generally applicable and highly stereospecific glycosylation method with glycal epoxides has yet to be developed.

Alternatively, glycosylation by leveraging neighboring group participation has been remarkably successful.^[^ ^23^ ^]^ However, this strategy becomes *less effective in glycosylation between electron‐deficient glycosyl donors and hindered secondary acceptors*: low stereoselectivity is often observed (Figure 1b).^[^ ^24^, ^25^, ^26^, ^27^, ^28^, ^29^ ^]^ The stereochemical erosion could arise from competing reactions with multiple intermediates in equilibrium,^[^ ^30^ ^]^ which is the intrinsic limitation of this approach (Figure 1b). This approach is also problematic for glucuronic ester donors that are critical for the synthesis of GAGs and proteoglycans, because these donors are difficult to activate due to their inherent low reactivty (Figure 1c).^[^ ^22^, ^31^, ^32^ ^]^

We envisioned that these two challenges could be addressed with a catalyst that can induce highly stereospecific glycosylation of a glycal epoxide with a variety of sterically hindered glycosyl acceptors. To provide a broadly applicable glycosylation approach that also complements the existing methods, we report herein an iron‐catalyzed highly stereospecific glycosylation method with glycal epoxides (Figure 1d). This method is effective for previously challenging hindered secondary acceptors and electron‐deficient glucuronic ester epoxides. Our kinetic studies revealed that this glycosylation proceeds through S_N_2‐type pathways with both primary and hindered secondary acceptors.

## Results and Discussion

**Figure 2:**
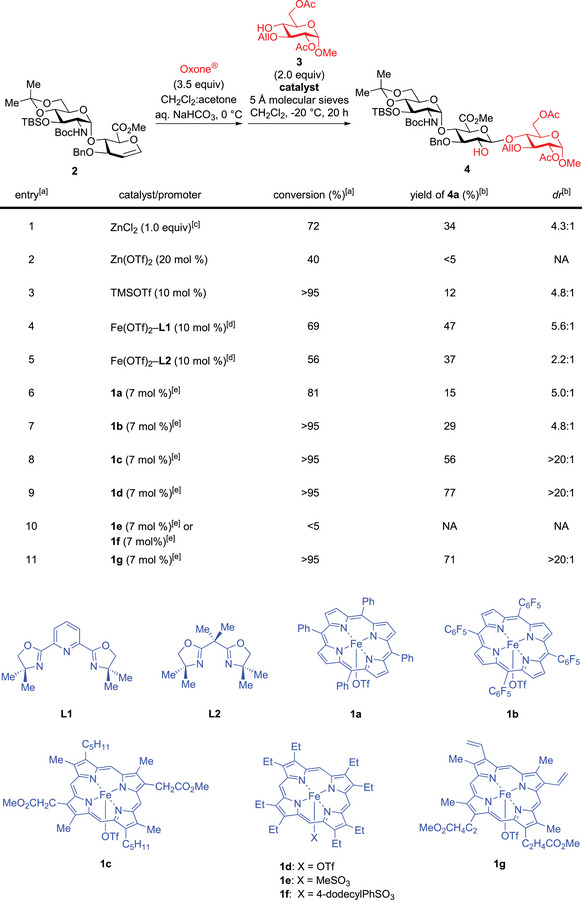
Catalyst discovery for the iron‐catalyzed stereospecific glycosylation with glycal epoxides. ^a)^ Epoxidation was carried out in a biphasic reaction medium with Oxone^®^ and acetone. The glycal epoxide was dried azeotropically with toluene, assayed by ^1^H NMR, and then directly used. The glycosylation was carried out at −20 °C in CH_2_Cl_2_. The reaction was quenched by methanol and imidazole for conversion measurement. ^b)^ Isolated yield; *dr* was determined by ^1^H NMR analysis. ^c)^ 0 °C in THF. ^d)^ CH_2_Cl_2_/MeCN (10:1) as the solvent. ^e)^ Iron(III) porphyrin catalysts 1 were formed in situ from the corresponding iron porphyrin chloride (10 mol%) and AgOTf or silver sulfonates (7 mol%).

We selected a glucosamine (GlcN)‐α‐1,4‐glucuronic acid (GlcA)‐derived glycal **2** as the model substrate for catalyst discovery, because it is directly related to these two synthetic challenges (Figure 2).^[^ ^22^, ^33^ ^]^ It is worth noting that our attempts to glycosylate a sterically hindered acceptor **3** with several common glycosyl donors related to **2** were unsuccessful. Epoxidation of **2** in a biphasic reaction medium with Oxone^[^ ^34^ ^]^ quantitatively afforded the corresponding α‐epoxide (Figure S1, *dr* > 20:1, ^3^ *J*_H1‐H2_ = 2.4 Hz),^[^ ^35^ ^]^ which was azeotropically dried and used directly. Exploration of an array of previously reported Brønsted acid and Lewis acid catalysts revealed that they mostly induce glycal epoxide decomposition and thereby are unsuitable for the desired glycosylation (entries 1–3 in Figure 2 and Table S1).^[^ ^3^, ^4^, ^5^, ^6^, ^7^, ^8^, ^9^, ^13^, ^14^, ^15^, ^18^, ^19^ ^]^ Notably, glycosylation using the stoichiometric ZnCl_2_ method reported by Danishefsky^[^ ^3^, ^4^, ^5^ ^]^ afforded trisaccharide **4** in modest yield and *dr* (entry 1, 34% yield, *dr*: 4.3:1).

We observed that both Fe(OTf)_2_–**L1** ^[^ ^36^ ^]^ and Fe(OTf)_2_–**L2** ^[^ ^37^ ^]^ catalysts evidently improve the yields of glycosylation with modest *dr* (entries 4–5). Entirely by serendipity, we observed that although two iron(III) porphyrin catalysts, Fe(TPP)OTf (**1a**)^[^ ^38^, ^39^ ^]^ and Fe(F_20_‐TPP)OTf (**1b**),^[^ ^40^, ^41^ ^]^ induced moderate *dr* (entries 6–7), iron catalyst **1c** ^[^ ^42^ ^]^ did promote an entirely stereospecific glycosylation with **3**, affording **4** (entry 8, *dr* > 20:1, ^3^ *J*_H1‐H2_ = 7.6 Hz). We further discovered that iron(III) octaethylporphyrin triflate, Fe(OEP)OTf (**1d**),^[^ ^43^ ^]^ generated in situ from Fe(OEP)Cl and AgOTf, catalyzes the exclusively stereospecific glycosylation in high yield (entry 9, 77% yield). With this electron‐deficient glycal epoxide, two iron catalysts with more coordinating sulfonate anions (**1e**–**1f**) led to low reactivity (entry 10, <5% conversion). Most notably, readily available hemin‐derived catalyst **1g** ^[^ ^44^ ^]^ is equally effective in promoting the entirely stereospecific glycosylation of this glycal epoxide (entry 11, 71% yield, *dr* > 20:1).

**Figure 3:**
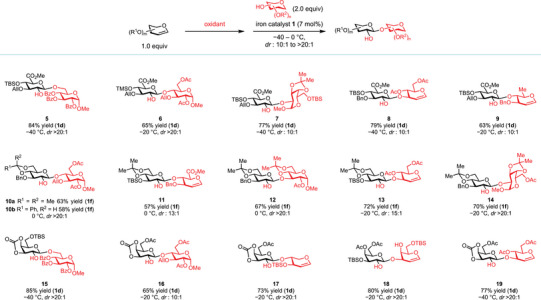
Iron‐catalyzed highly stereospecific glycosylation with monosaccharide‐derived glycal epoxides. All yields are isolated yields.

**Figure 4:**
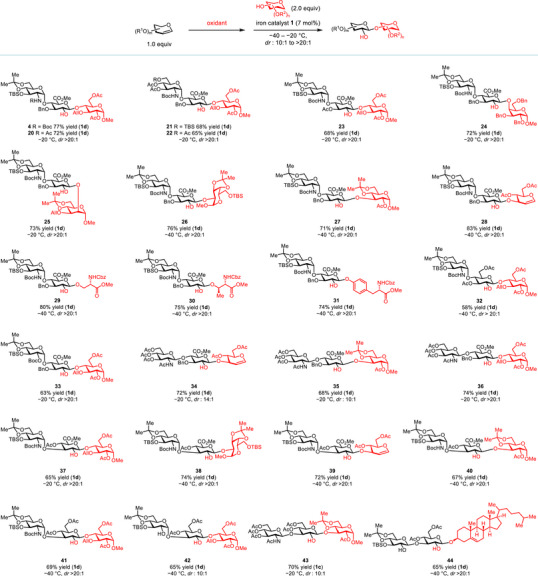
Iron‐catalyzed highly stereospecific glycosylation with complex glycal epoxides. All yields are isolated yields.

With the optimal catalysts in hand, we evaluated a variety of glycals and glycosyl acceptors to determine the generality of this method (Figures 3 and 4). Glucuronic ester epoxides are not suitable substrates for the existing glycosylation methods with sugar acceptors,^[^ ^21^, ^45^ ^]^ but catalysts **1d** and **1g** are effective for the stereospecific glycosylation of these donors with both primary and hindered secondary acceptors in excellent *dr* (products **5**–**9**, *dr* from 10:1 to >20:1), including 6‐deoxy acceptors that are incorporated in marine oligosaccharides.

Electron‐rich glucal epoxides are valuable synthetic intermediates, but with hindered secondary acceptors, the known catalysts and promoters often mediate their irreversible opening and decomposition with concurrent S_N_1‐type glycosylation, which affords a diastereomeric mixture in low yields.^[^ ^3^, ^4^, ^5^, ^6^, ^7^, ^8^, ^9^, ^13^, ^14^, ^15^, ^18^, ^19^, ^20^ ^]^ Although iron triflate catalysts (**1d** and **1g**) induced only moderately stereospecific glycosylation with electron‐rich glucal epoxides, iron catalysts **1e** and **1f** with more coordinating sulfonate anions did promote highly stereospecific glycosylation with a broad array of sterically hindered, secondary acceptors (products **10**–**14**, *dr* from 13:1 to >20:1). In particular, these two catalysts are effective in forging the β‐1,4‐linkages in high *dr* between glucal epoxides and glucose and glucuronic acid‐derived acceptors (products **10** and **11**), which was previously difficult.^[^ ^3^, ^4^, ^5^, ^6^, ^7^, ^8^, ^9^, ^13^, ^14^, ^15^, ^18^, ^19^ ^]^

Galactals are often used as building blocks in complex‐glycan synthesis and we discovered that they can be engaged in this iron‐catalyzed stereospecific glycosylation with a range of acceptors, including sterically demanding ones from glucose and xylal (products **15**–**19**, *dr* from 10:1 to >20:1).

Building upon these successes, we explored a broad range of easily prepared, disaccharide‐based glycals to evaluate the synthetic utility and functional‐group compatibility of this method (Figure 4). First, iron catalyst **1d** is compatible with an array of GlcN‐α‐1,4‐GlcA‐derived donors that are structural motifs of heparan sulfate.^[^ ^46^ ^]^ These donors are readily assembled with hindered acceptor **3** in high yields (products **4**, **20**–**23**, *dr* > 20:1). Notably, structural variations either on the glucosamine or on the glucuronic acid domain do not compromise the high stereoselectivity. Next, sterically demanding β‐1,4‐ and β‐1,2‐linkages can be exclusively formed between glycal **2** and galactose‐ and mannose‐based acceptors (products **24**–**25**). Additionally, a wide variety of glycosyl acceptors, including serine, threonine, and tyrosine methyl esters can smoothly participate in this reaction, all of which deliver a single diastereomeric product in high yields (products **26**–**31**, *dr* > 20:1). Furthermore, both a GlcN‐α‐1,4‐glucose(Glu)‐derived epoxide and a Glu‐α‐1,4‐GlcA‐derived epoxide are excellent substrates and the corresponding glycosylation products with **3** are obtained as single diastereomers (products **32**–**33**).

Next, we investigated a disaccharide donor derived from GlcN‐β‐1,4‐GlcA that is the repeating unit of hyaluronic acid, another valuable member of GAGs.^[^ ^47^ ^]^ It is readily joined with a list of secondary acceptors in high yields (products **34**–**36**, *dr* from 10:1 to >20:1). We subsequently evaluated an array of disaccharide‐based glycals that are synthetically valuable. They include the epoxides derived from GlcN‐α‐1,3‐GlcA, GlcN‐α‐1,3‐Glu, Glu‐α‐1,3‐Glu, GlcN‐β‐1,3‐Glu, and Glu‐β‐1,3‐Glu. We observed that all of them can be connected with a variety of hindered secondary acceptors in good yields (products **37**–**44**, *dr* from 10:1 to >20:1). Notably, this method is effective with cholesterol, a steroidal acceptor, which makes it valuable for stereoselective saponin synthesis.^[^ ^48^ ^]^

**Scheme 1:**
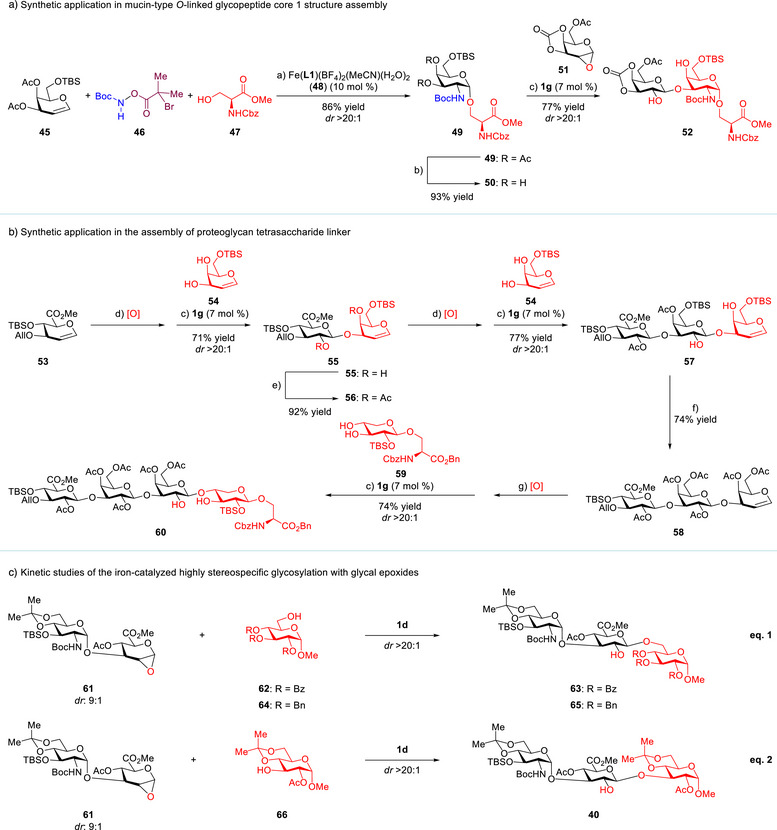
Synthetic applications and kinetic studies of the iron‐catalyzed stereospecific glycosylation with glycal epoxides. a) The reaction was carried out with iron catalyst **48** (10 mol%), **45** (1.0 equiv), **47** (1.4 equiv), and **46** (1.5 equiv) in the presence of 5 Å molecular sieves in CH_2_Cl_2_ at −40 °C. b) Na_2_CO_3_, MeOH, −10 °C. c) The reaction was carried out with iron catalyst **1g** (7 mol%) in the presence of 5 Å molecular sieves in CH_2_Cl_2_ at −20 °C. d) Oxone^®^ (3.0 equiv), 5:1 CH_2_Cl_2_/acetone, saturated NaHCO_3_ solution, 0 °C. e) Ac_2_O, Et_3_N, DMAP, CH_2_Cl_2_, 22 °C. f) TBAF, AcOH, in THF, 0 °C; then Ac_2_O, Et_3_N, DMAP, in CH_2_Cl_2_, 22 °C. g) DMDO (1.1 equiv), CH_2_Cl_2_/acetone, 0 °C. See Supporting Information for details.

The most prevalent *O*‐linked protein glycosylation in eukaryotes is mucin‐type glycosylation, in which 8 GalNAc‐α‐1‐*O*‐serine or threonine‐derived glyco‐amino acids serve as core structures for further enzymatic elaboration.^[^ ^49^ ^]^ Notably, the core 1 structure^[^ ^50^, ^51^, ^52^, ^53^ ^]^ can be rapidly synthesized using this method (Scheme 1a). We discovered that a galactal‐derived donor **45** and a serine methyl ester **47** can be assembled, via the iron‐catalyzed glycal 1,2‐*cis*‐aminoglycosylation,^[^ ^33^, ^54^, ^55^ ^]^ affording **49** (*dr* > 20:1). The di‐deacetylated product **50** can thereby be engaged in this iron‐catalyzed stereospecific glycosylation to deliver **52** (*dr* > 20:1), which is easily converted to core 1 (Figure S5).

Proteoglycans are heavily glycosylated proteins that have basic units containing a core protein covalently attached to GAGs.^[^ ^56^ ^]^ A tetrasaccharide fragment connected entirely via 1,2‐*trans*‐glycosidic linkages serves as a bridge to attach the GAG to a serine residue of the core protein (Figure 1c). Several truly impressive proteoglycan syntheses have been reported,^[^ ^28^, ^31^ ^]^ which enables important biological studies; however, it is still difficult to precisely control the stereoselectivity in each chemical glycosylation during proteoglycan synthesis.^[^ ^57^, ^58^, ^59^, ^60^, ^61^ ^]^ We envisioned that these 1,2‐*trans*‐glycosidic bonds could be formed efficiently via the iron‐catalyzed, reiterative and stereospecific glycosylation with glycal epoxides (Scheme 1b). First, hemin‐derived catalyst **1g** catalyzed the glycosylation of 6‐*O*‐TBS‐galactal (**54**) with a glucuronic ester epoxide from **53**, delivering a single diastereomeric, β‐1,3‐linked disaccharide **55** (71%). Next, the same catalyst promoted the exclusively stereospecific glycosylation of the newly generated glycal epoxide from **56** with acceptor **54**, affording trisaccharide **57** (77%). Most notably, a third iron‐catalyzed glycosylation effectively connected the trisaccharide donor with a xylose‐β‐1‐*O*‐serine‐derived acceptor **59**, providing tetrasaccharide **60** in 74% yield (*dr* > 20:1). It is worth noting that acceptor **59** was also synthesized via the iron‐catalyzed stereospecific glycosylation of a xylal epoxide with a serine benzyl ester (*dr*: 12.5:1, Figure S7).

Jacobsen's elegant synthetic and mechanistic studies of metal salen‐catalyzed stereoselective epoxide ring‐opening have established the cooperative homo‐bimetallic mechanism as a general principle in asymmetric catalysis.^[^ ^62^, ^63^, ^64^, ^65^, ^66^ ^]^ Therefore, we are interested in investigating the mechanism of this stereospecific glycosylation. Preliminary kinetic studies of this iron‐catalyzed glycosylation of **61** with primary acceptor **62** or **64** (eq. 1 in Scheme 1c) suggested that its initial rate has a *first‐order* dependence on both the iron catalyst **1d** and glycal epoxide **61**, but a *zero‐order* dependence on acceptor **62** or **64** (Figures S8–S11). Interestingly, a much slower initial rate was observed with a more nucleophilic primary acceptor **64** (Figures S10 and S11). Further kinetic studies revealed that the initial rate of glycosylation of **61** with secondary acceptor **66** (eq. 2) has a *first‐order* dependence on each of the iron catalyst **1d**, glycal epoxide **61** and acceptor **66** (Figures S12–S14).

The *zero‐order* rate dependence on a primary acceptor is particularly intriguing, as it implies two possible mechanistic scenarios. First, the glycosylation might proceed through an S_N_1‐type pathway with a primary acceptor even though it proceeds through an S_N_2‐type pathway with a secondary acceptor. It is difficult to envision that a more nucleophilic primary acceptor would participate in an S_N_1‐type reaction when a less nucleophilic secondary acceptor reacts via an S_N_2‐type pathway. Alternatively, both glycosylations might occur via S_N_2‐type pathways,^[^ ^67^ ^]^ but the primary acceptor could strongly coordinate with the iron catalyst, such that the concentration of the active iron catalyst has an inverse first‐order dependence on the primary acceptor (Figure S15). Therefore, the overall zero‐order rate dependence on a primary acceptor was observed.

To differentiate these two mechanistic possibilities, we measured the glycosylation rates of both primary and secondary acceptor **64** and **66** (1:1) in competition with donor **61**: the initial rate with secondary acceptor **66** drops more than 4‐fold, but the one with primary acceptor **64** remains the same (Figure S16). These results suggested that the primary acceptor indeed coordinates with the catalyst and effectively decreases the concentration of active iron catalyst available for glycosylation.

**Scheme 2:**
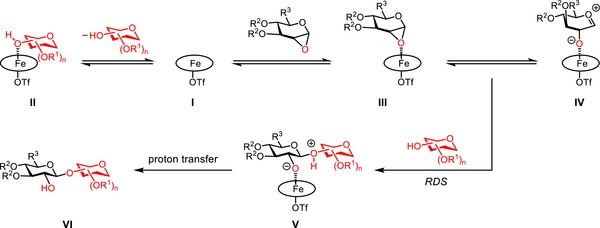
Mechanistic working hypothesis for the iron‐catalyzed stereospecific glycosylation with glycal epoxides.

These data help us propose a mechanistic working hypothesis for this glycosylation (Scheme 2). First, an iron(III) porphyrin triflate catalyst **I** could readily coordinate with a primary glycosyl acceptor, generating complex **II** and thereby decreasing the concentration of active iron catalyst in solution. A secondary glycosyl acceptor may be too hindered to coordinate with **I**, such that the concentration of active iron catalyst is unaffected. Catalyst **I** could react with a glycal α‐epoxide to form iron catalyst–epoxide complex **III**, presumably with a significantly elongated C1─O bond,^[^ ^68^ ^]^ which might be in equilibrium with a zwitterionic intermediate **IV**. Notably, it is difficult to distinguish intermediate **III** from intermediate **IV** experimentally.^[^ ^69^, ^70^ ^]^ In the presence of a glycosyl acceptor, the rate‐determining glycosylation could occur with **III**/**IV** through an S_N_2‐type pathway, delivering intermediate **V**, which is converted to the glycosylation product **VI** via proton transfer.

## Conclusion

In conclusion, we have developed an iron‐catalyzed highly stereospecific glycosylation method with glycal epoxides. This method is effective for a wide variety of previously difficult hindered secondary acceptors and electron‐deficient glucuronic ester epoxides. It addresses the challenges associated with the existing glycosylation methods with glycal epoxides and the standard neighboring group participation glycosylation strategies. Mechanistic studies revealed a unique S_N_2‐type glycosylation mechanism with both primary and hindered secondary acceptors. Our current effort focuses on its synthetic applications in glycosaminoglycan assembly.

## Conflict of Interests

The subject matter described in this article is included in patent applications filed by Brandeis University.

## Supporting information



Supporting Information

## Data Availability

The data that support the findings of this study are available from the corresponding author upon reasonable request.
