# Supplementary material for: Iron‐Catalyzed Highly Stereospecific Glycosylation with Glycal Epoxides
Source: Angew Chem Int Ed Engl. 2025 Oct 3;64(48):e202517634. doi: 10.1002/anie.202517634 (PMC12532074; doi:10.1002/anie.202517634)
Supplement: Supplementary file 1 — Supporting Information [file ANIE-64-e202517634-s001.pdf]

Supporting Information

Iron-Catalyzed Highly Stereospecific Glycosylation with Glycal Epoxides

Xiao-Wen Zhang,[‡] Le Yin,[‡] Dakang Zhang,[‡] Zixiang Jiang,[‡] Pinzhi Wang, and Hao Xu*

haohxu@brandeis.edu

Department of Chemistry, Brandeis University, 415 South Street, Waltham, Massachusetts 02453, United States

A. General Information

B. Catalyst Discovery for the Iron-Catalyzed Stereospecific Glycosylation with Glycal Epoxides

C. General Procedures for the Iron-Catalyzed Stereospecific Glycosylation with Glycal Epoxides

D. Synthetic Applications of the Iron-Catalyzed Stereospecific Glycosylation with Glycal Epoxides

E. Preliminary Kinetic Studies of the Iron-Catalyzed Stereospecific Glycosylation with Glycal Epoxides

F. References

G. NMR Spectra

A. General Information

General Procedures. All reactions were performed in oven-dried or flame-dried round-bottom flasks and vials. Stainless steel syringes and cannula were used to transfer air- and moisture-sensitive liquids. Flash chromatography was performed using silica gel 60 (230−400 mesh) from Sigma–Aldrich.

Materials. Commercial reagents were purchased from Sigma–Aldrich, TCI, Oakwood Chemicals, Combi-Blocks, Chem-Impex, Thermo Fischer Scientific and used as received. All solvents were used after being freshly distilled unless otherwise noted.

Instrumentation. Proton nuclear magnetic resonance (^{1}H NMR) spectra and carbon nuclear magnetic resonance (^{13}C NMR) spectra were recorded on Advance NEO 400 (400 MHz) and Varian 400-MR (400 MHz). Chemical shifts for protons are reported in parts per million downfield from tetramethylsilane and are referenced to the NMR solvent residual peak ($CHCl_3$ δ 7.26, CD_3OD δ 3.31, acetone-d_6 δ 2.05, C_6D_6 δ 7.16). Chemical shifts for carbons are reported in parts per million downfield from tetramethylsilane and are referenced to the carbon resonances of the NMR solvent ($CDCl_3$ δ 77.0, CD_3OD δ 49.0, acetone-d_6 δ 29.8, C_6D_6 δ 128.0). Data are represented as follows: chemical shift, multiplicity (br = broad, s = singlet, d = doublet, t = triplet, q = quartet, quint = quintet, m = multiplet), coupling constants in Hertz (Hz), and integration. The mass spectroscopic data were obtained using a Bruker timsTOF Pro instrument by electrospray ionization (ESI). Infrared (IR) spectra were obtained using a Nicolet IR200 spectrometer with a diamond ATR. Data are represented as follows: frequency of absorption (cm^{-1}) and absorption strength (s = strong, m = medium, w = weak). Optical rotations were measured on a Jasco P-2000 Polarimeter. The cuvette dimension is 10 cm and holds 1.5 mL.

Abbreviations Used: THF–tetrahydrofuran, EtOAc–ethyl acetate, Et_2O–diethyl ether, CH_2Cl_2–dichloromethane, TMSOTf–trimethylsilyl trifluoromethanesulfonate, TLC–thin layer chromatography, Boc_2O–di-*tert*-butyl dicarbonate, DMAP–4-dimethylaminopyridine, Et_3N−triethylamine, TBSCl−*tert*-butyldimethylsilyl chloride, Ac_2O−acetic anhydride, AcCl−acetyl chloride, TBAF−tetra-*n*-butylammonium fluoride, OTf−trifluoromethanesulfonate,

$AgSO_3C_6H_4C_{12}H_{25}$−silver 4-dodecylbenzenesulfonate (mixture of isomers), OEP−octaethylporphyrin, DMDO−dimethyldioxirane, All−allyl, Cbz−benzyloxycarbonyl.

B. Catalyst Discovery for the Iron-Catalyzed Stereospecific Glycosylation with Glycal Epoxides

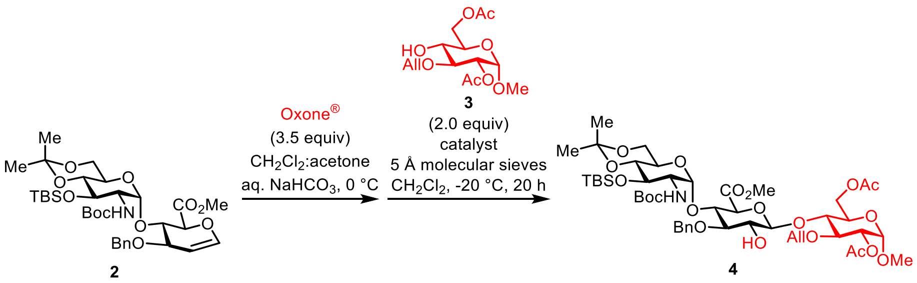

a. General Procedures for Catalyst Discovery

Procedures for Glycal Epoxidation

Procedure A

To a 25 mL flask equipped with a stir bar at 0 °C, were added glycal **2**[1] (81.5 mg, 0.12 mmol, 1.0 equiv) in CH_2Cl_2/acetone mixture (v/v: 5:1, 2.0 mL) and saturated aqueous $NaHCO_3$ solution (3.4 mL), followed by addition of Oxone® ($KHSO_5 \cdot 0.5KHSO_4 \cdot 0.5K_2SO_4$) (258.6 mg, 0.42 mmol, 3.5 equiv) in H_2O (2.2 mL) dropwise. After stirring vigorously at 0 °C for 2 h, the reaction mixture was extracted with CH_2Cl_2 (3 mL × 3). The combined organic phase was dried over anhydrous Na_2SO_4 and concentrated *in vacuo*. The residue was further azeotropically dried with anhydrous toluene (1.0 mL). The obtained glycal epoxide was assayed by ^{1}H NMR to get the diastereomeric ratio (*dr* > 20:1) and directly used in the next step.

Procedure B

For glycals that are hydrophilic, an alternative epoxidation procedure can be applied.

To a 25 mL flask equipped with a stir bar at 0 °C, were added glycal **2** (81.5 mg, 0.12 mmol, 1.0 equiv) in CH_2Cl_2, followed by the addition of freshly prepared dimethyldioxirane (DMDO in acetone, 0.06 M, 3 mL, 0.18 mmol, 1.5 equiv)[2] dropwise. After stirring at 0 °C for 2 h, the

reaction mixture was concentrated *in vacuo*, then the residue was re-dissolved in anhydrous CH_2Cl_2 (2 mL) and dried over anhydrous Na_2SO_4. The organic phase concentrated *in vacuo*. The residue was further dried azeotropically with anhydrous toluene (1.0 mL). The obtained glycal epoxide was assayed by ^{1}H NMR to get the diastereomeric ratio (*dr* > 20:1) and directly used in the next step.

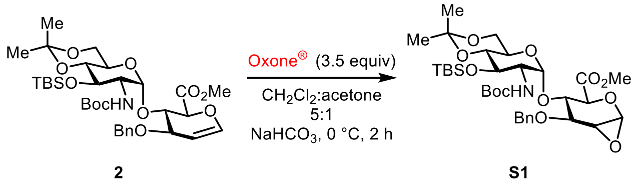

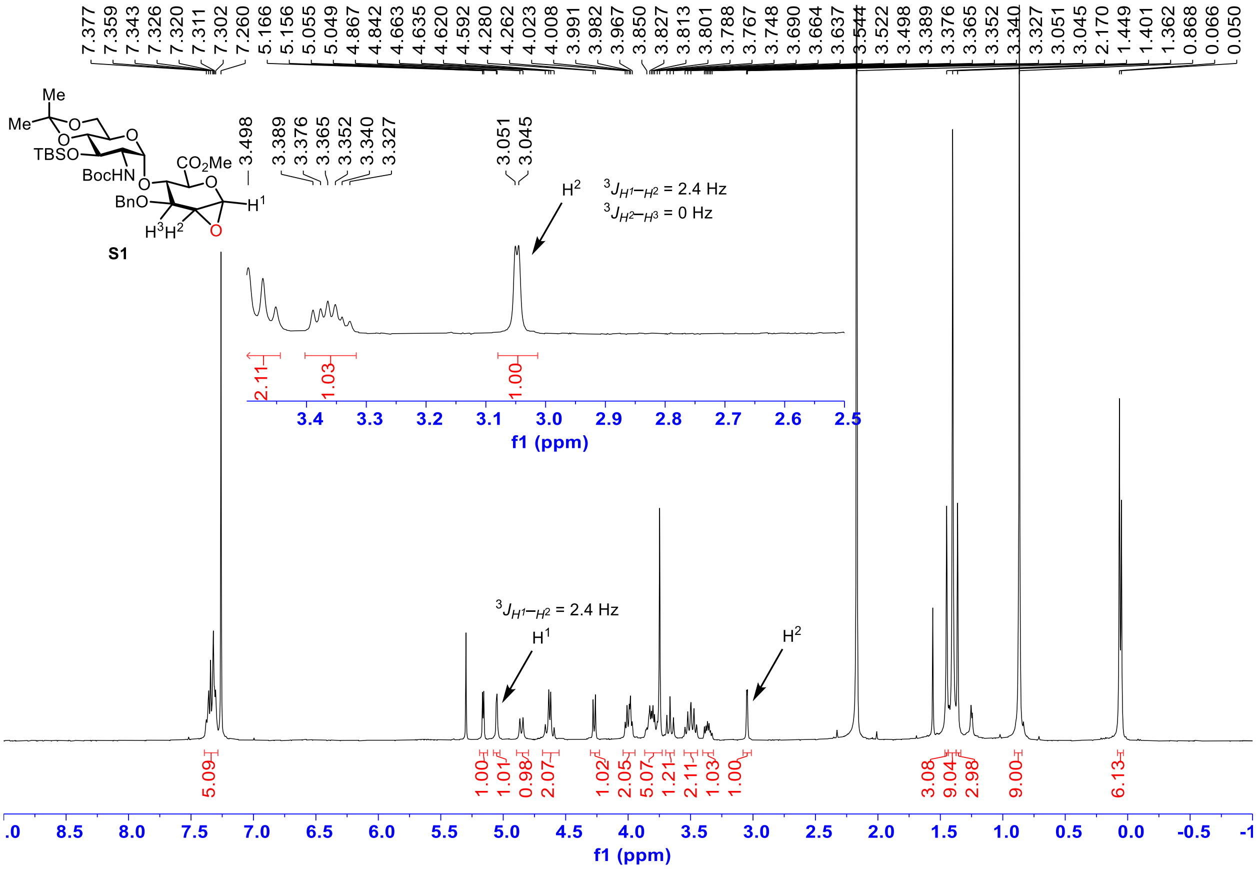

Figure S1. Stereochemistry Determination of Glycal Epoxide **S1**.

The stereochemistry of the glycal epoxide **S1** was determined by ^{1}H NMR analysis. It is known that the C(2)–H chemical shift of a glucal α-epoxide is around 3.0 ppm, whereas that of a glucal β-epoxide is around 3.3 ppm.[3]

Additionally, the $^3J_{H^1-H^2}$ value for a typical glucal α-epoxide is around 2.4 Hz, reflecting the dihedral angle ($\Phi_{1,2}$) that approaches 0°, whereas the $^3J_{H^2-H^3}$ value is < 0.5 Hz because the dihedral angle ($\Phi_{2,3}$) approaches 90°, characteristic of a *trans*-relationship between H^2 and H^3 that is pseudo-axial.[3] As a result, the H^2 signal of a glucal α-epoxide appears as a doublet.

In contrast, while a glucal β-epoxide retains a similar $\Phi_{1,2}$ and thus a comparable $^3J_{H^1-H^2}$, its $\Phi_{2,3}$ is around 45°, leading to a $^3J_{H^2-H^3}$ around 1.7 Hz.[3] This causes the H^2 signal to appear as a doublet of doublets (though often observed as a triplet due to the broadening line width).

Based on these data, we have assigned glycal epoxide **S1** as an α-epoxide (*dr* > 20:1) with $^3J_{H^1-H^2}$ = 2.4 Hz and $^3J_{H^2-H^3}$ = 0 Hz. This assignment was further corroborated by the stereochemistry analysis of the iron catalyzed stereospecific glycosylation product 1,2-*trans*-β-glycoside **4**.

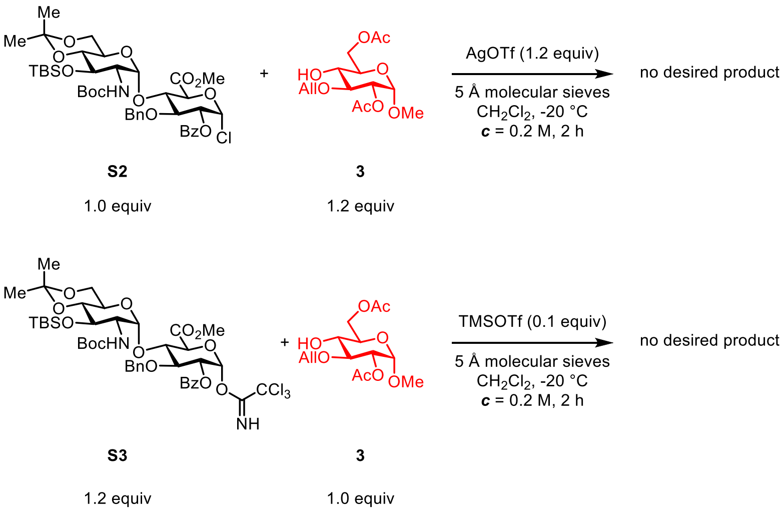

Figure S2. Glycosylation Attempts with Common Glucuronic Ester Donors Related to **2**.

In the attempted AgOTf-promoted glycosylation between a glycosyl chloride **S2** and a hindered glycosyl acceptor **3**, low conversion (<5%) was observed and there was no desired product obtained. In the attempted TMSOTf-catalyzed glycosylation between a glycosyl trichloroacetamide donor **S3** and acceptor **3**, low conversion (<5%) was observed and there was no desired product obtained.

Procedures for Preparation of Iron(III) Porphyrin Triflate and Other Sulfonate Catalysts

To a flame-dried sealable 2-dram vial equipped with a stir bar were added an iron(III) porphyrin chloride catalyst (0.012 mmol, 10 mol %) and AgOTf (2.2 mg, 0.0084 mmol, 7 mol %). After the vial was evacuated and backfilled with N_2 three times, anhydrous CH_2Cl_2 (0.3 mL) was added and the solution was stirred at room temperature for 10 min.

To prepare other iron(III) porphyrin sulfonate catalysts, the corresponding silver sulfonate (0.0084 mmol, 7 mol %) was used instead of AgOTf, and the solution was stirred for 30–60 min at room temperature.

Procedures for the Iron-Catalyzed Stereospecific Glycosylation with Glycal Epoxides

Procedure C

To a flame-dried sealable 2-dram vial equipped with a stir bar were added glycosyl acceptor **3** (0.24 mmol, 2.0 equiv), an iron catalyst (0.0084 mmol, 7 mol %), and freshly activated 5 Å molecular sieves, powder (*ca.* 100 mg). After the vial was evacuated and backfilled with N_2 three times, anhydrous CH_2Cl_2 (0.3 mL) was added and the solution was stirred at -40 °C for 10 min. The aforementioned glycal epoxide (0.12 mmol, 1.0 equiv) was dissolved in anhydrous CH_2Cl_2 (0.5 mL) and transferred to the mixture at -40 °C dropwise. The reaction mixture was kept at -20 °C for 20 h and then quenched with MeOH (50 μL) and imidazole (8.5 mg in 1 mL CH_2Cl_2) at the same temperature. The mixture was filtered through a piece of cotton and eluted with EtOAc (2 mL × 2). The organic layer was then concentrated *in vacuo*. The *dr* was determined based on the ^{1}H NMR analysis of the crude reaction mixture. The residue was

purified through column chromatography (hexanes/EtOAc: from 20:1 to 2:1) to afford the desired glycosylation product **4**.

Table S1. Catalyst Discovery for the Iron-Catalyzed Stereospecific Glycosylation with Glycal Epoxide

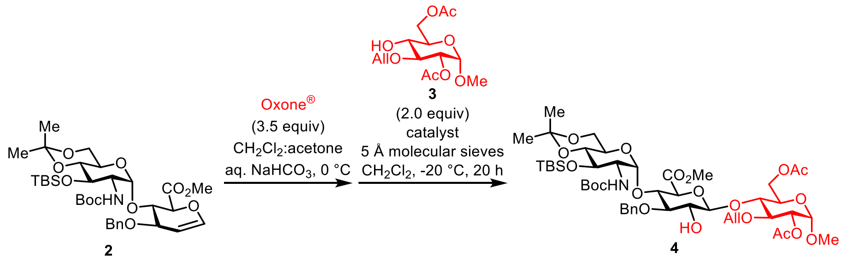

entry[a]	catalyst/promotor	conversion (%)[a]	yield of **4a** (%)[b]	*dr*[b]
1	methanesulfonic acid (10 mol %)	>95	<5	NA
2	camphorsulfonic acid (10 mol %)	>95	<5	NA
3	**S4a** (10 mol %)	<10	<5	NA
4	$(PhO)_2POOH$ (10 mol %)	<10	<5	NA
5	**S4b** (10 mol %)	<10	<5	NA
6	**S4c** (10 mol %)	<10	<5	NA
7	**S4d** (10 mol %)	<10	<5	NA
8	$ZnCl_2$ (1.0 equiv)[c]	72	34	4.3:1
9	$Zn(OTf)_2$ (20 mol %)	40	<5	NA
10	TMSOTf (10 mol %)	>95	12	4.8:1
11	$Fe(OTf)_2$–**L1** (10 mol %)[d]	69	47	5.6:1
12	$Fe(OTf)_2$–**L2** (10 mol %)[d]	56	37	2.2:1
13	**1a** (7 mol %)[e]	81	15	5.0:1
14	**1b** (7 mol %)[e]	>95	29	4.8:1
15	**1c** (7 mol %)[e]	>95	56	>20:1
16	**1d** (7 mol %)[e]	>95	77	>20:1
17	**1e** (7 mol %)[e] or **1f** (7 mol%)[e]	<5	NA	NA
18	**1g** (7 mol %)[e]	>95	71	>20:1

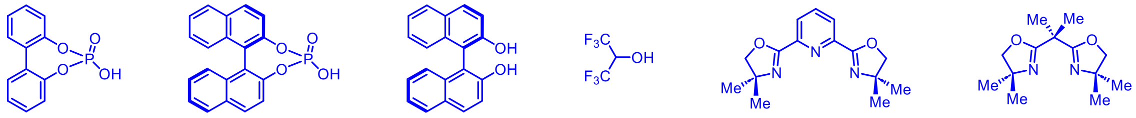

S4a **S4b** **S4c** **S4d** **L1** **L2**

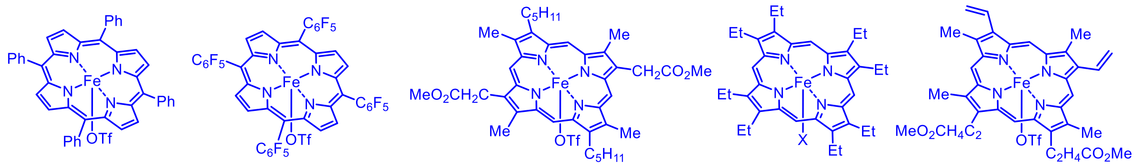

1a **1b** **1c** **1d**: X = OTf; **1e**: X = $MeSO_3$; **1f**: X = 4-dodecylPhSO$_3$ **1g**

[a]Epoxidation was carried out in a biphasic reaction medium with Oxone® and acetone. The glycal epoxide was dried azeotropically with toluene, assayed by ^{1}H NMR, and then directly used. The glycosylation was carried out at -20 °C in CH_2Cl_2. The reaction was quenched by methanol and imidazole for conversion measurement. [b]Isolated yield; *dr* was determined by ^{1}H NMR analysis. [c]0 °C in THF. [d]CH_2Cl_2/MeCN (10:1) as the solvent. [e]Iron(III) porphyrin triflate/sulfonate catalysts **1** were formed *in situ* from the corresponding iron porphyrin chloride and AgOTf or silver sulfonates.

2 was synthesized according to a literature procedure.[1]

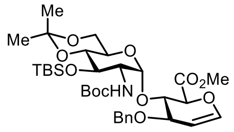

2

Methyl 3-*O*-benzyl-4-*O*-(2-*tert*-butoxycarbonylamino-3-*O*-*tert*-butyldimethylsilyl-4,6-*O*-isopropylidene-2-deoxy-α-D-glucopyranosyl)-D-glucuronal (**2**): $[\alpha]_D^{23}$ +27.9 (acetone, *c* = 1.0); IR ν_{max} (neat)/cm^{-1}: 2952 (w), 2928 (w), 2357 (w), 2359 (w), 1763 (w), 1721 (m), 1648 (w), 1501 (m), 1367 (w), 1129 (s), 1072 (s), 996 (m), 875 (m), 863 (m); ^{1}H NMR (400 MHz, $CDCl_3$) δ 7.37 – 7.25 (m, 5H), 6.67 (d, *J* = 6.3 Hz, 1H), 5.03 – 4.94 (m, 2H), 4.82 (d, *J* = 1.8 Hz, 1H), 4.57 – 4.48 (m, 2H), 4.42 (d, *J* = 11.3 Hz, 1H), 4.36 (q, *J* = 2.4 Hz, 1H), 3.90 – 3.76 (m, 3H), 3.71 (t, *J* = 10.3 Hz, 1H), 3.62 (td, *J* = 9.4, 4.9 Hz, 1H), 3.56 (s, 3H), 3.58 – 3.46 (m, 2H), 1.47 (s, 3H), 1.42 (s, 9H), 1.39 (s, 3H), 0.86 (s, 9H), 0.05 (s, 3H), 0.04 (s, 3H); ^{13}C NMR (100 MHz, $CDCl_3$) δ 168.2, 155.3, 145.6, 137.7, 128.5 (2C), 128.1 (2C), 128.0, 99.5, 99.2, 98.1, 79.7, 74.8, 74.0, 72.4, 71.2, 69.7, 67.1, 64.6, 62.4, 55.6, 52.3, 29.2, 28.6 (3C), 25.9 (3C), 19.1, 18.4, -4.0, -4.9; HRMS: m/z (ESI) calcd for $C_{34}H_{54}NO_{11}Si^+$, $[M + H]^+$, 680.3461, found 680.3458. $^{1}J_{^{13}C1-H1}$ = 168.3 Hz.

3 was synthesized according to the following procedure.

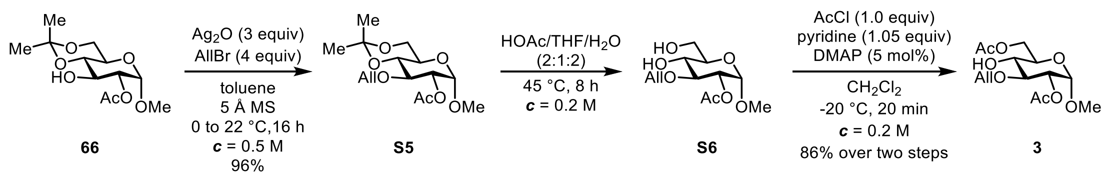

To a 100 mL flame-dried round bottom flask equipped with a stir bar, compound **66**[4] (1.00 g, 3.62 mmol, 1.0 equiv), Ag_2O (2.51 g, 10.82 mmol, 3.0 equiv), freshly activated 5 Å molecular sieves (powder, ca. 500 mg), and anhydrous toluene (7.2 mL) were added. The mixture was cooled to 0 °C before allyl bromide (1.25 mL, 14.48 mmol, 4.0 equiv) was added dropwise. The reaction mixture was then stirred at room temperature for 16 h until the starting material **66** was fully consumed (monitored by TLC). The reaction mixture was then filtered through a short pad of Celite®, rinsed with acetone, and concentrated *in vacuo*. The residue was purified through column chromatography (hexanes/EtOAc: from 100:1 to 5:1) to afford the desired product **S5** (1.10 g, 96% yield) as white foam.

To a 50 mL flame-dried round bottom flask equipped with a stir bar, compound **S5** (1.10 g, 3.48 mmol, 1.0 equiv) and a mixture of AcOH/THF/H_2O (2:1:2 vol/vol/vol, 17.4 mL) were added. The mixture was stirred at 45 °C for 8 h until the starting material **S5** was fully consumed (monitored by TLC). The reaction mixture was concentrated *in vacuo* and the residue was further azeotropically dried with toluene (7 mL× 4) to remove AcOH and H_2O. The desired product **S6** was obtained as white foam and directly used in the next step without further purification.

To a 50 mL flame-dried round bottom flask equipped with a stir bar, the crude product **S6** from the last step, DMAP (21.2 mg, 0.174 mmol, 5 mol %), and anhydrous CH_2Cl_2 (17.4 mL) were added. The mixture was cooled to -20 °C before anhydrous pyridine (0.30 mL, 3.65 mmol, 1.05 equiv) and AcCl (1M in CH_2Cl_2, 3.48 mL, 3.48 mmol, 1.0 equiv) were added dropwise sequentially. The reaction mixture was stirred at -20 °C for 20 min until the starting material **S6** was fully consumed (monitored by TLC). The reaction mixture was then quenched with H_2O (10 mL) and the organic phase was separated from the aqueous one. The aqueous phase was further extracted with CH_2Cl_2 (10 mL × 2). The combined organic phase was washed with brine

(15 mL) and dried over Na_2SO_4. After concentration *in vacuo*, the residue was purified through column chromatography (hexanes/EtOAc: from 100:1 to 2:1) to afford the desired product **3** (0.95 g, 86% yield over two steps) as colorless oil.

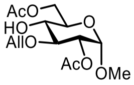

3

Methyl 2,6-di-*O*-acetyl-3-*O*-allyl-α-D-glucopyranoside (**3**): $[\alpha]_D^{23}$ +128.6 (acetone, *c* = 1.0); IR ν_{max} (neat)/cm^{-1}: 3480 (w), 2924 (w), 2360 (w), 2341 (w), 1740 (s), 1369 (m), 1232 (s), 1036 (s), 917 (w); ^1H NMR (400 MHz, $CDCl_3$) δ 6.02 – 5.81 (m, 1H), 5.26 (dd, *J* = 17.2, 1.6 Hz, 1H), 5.17 (dd, *J* = 10.4, 1.5 Hz, 1H), 4.88 (d, *J* = 3.7 Hz, 1H), 4.76 (dd, *J* = 10.0, 3.6 Hz, 1H), 4.46 (dd, *J* = 12.2, 4.4 Hz, 1H), 4.31 – 4.18 (m, 3H), 3.79 – 3.67 (m, 2H), 3.48 (t, *J* = 10.3 Hz, 1H), 3.38 (s, 3H), 2.82 (brs, 1H), 2.12 (s, 3H), 2.11 (s, 3H); ^{13}C NMR (100 MHz, $CDCl_3$) δ 171.6, 170.4, 134.9, 117.3, 97.3, 79.1, 74.2, 73.3, 70.2, 69.5, 63.1, 55.3, 21.2, 21.0; HRMS: m/z (ESI) calcd for $C_{14}H_{23}O_8^+$, $[M + H]^+$, 319.1387, found 319.1382.

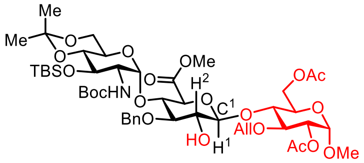

4

Methyl 2-*tert*-butoxycarbonylamino-3-*O*-*tert*-butyldimethylsilyl-4,6-*O*-isopropylidene-2-deoxy-α-D-glucopyranosyl-(1→4)-(methyl 3-*O*-benzyl-β-D-glucopyranosyluronate)-(1→4)-2,6-di-*O*-acetyl-3-*O*-allyl-α-D-glucopyranoside (**4**): $[\alpha]_D^{23}$ +63.3 (acetone, *c* = 1.0); IR ν_{max} (neat)/cm^{-1}: 3454 (w), 2929 (w), 2360 (w), 1741 (s), 1653 (w), 1367 (s), 1232 (s), 1026 (s), 987 (s), 913 (m), 874 (m); ^1H NMR (400 MHz, $CDCl_3$) δ 7.33 (d, *J* = 4.4 Hz, 5H), 5.84 (ddt, *J* = 16.2, 10.6, 5.3 Hz, 1H), 5.35 (d, *J* = 3.9 Hz, 1H), 5.22 (dd, *J* = 16.2, 1.9 Hz, 1H), 5.11 (dd, *J* = 10.6, 1.9 Hz, 1H), 4.92 – 4.80 (m, 3H), 4.81 – 4.70 (m, 2H), 4.48 (d, *J* = 7.6 Hz, 1H), 4.45 (d, *J* = 9.9 Hz, 1H), 4.34 (dd, *J* = 12.1, 4.5 Hz, 1H), 4.28 (dd, *J* = 12.9, 5.3 Hz, 1H), 4.10 (dd, *J* = 12.9, 5.3 Hz, 1H), 4.00 (t, *J* = 8.9 Hz, 1H), 3.88 (d, *J* = 9.8 Hz, 1H), 3.85 – 3.73 (m, 4H), 3.78 (s, 3H),

3.71 – 3.59 (m, 2H), 3.61 – 3.54 (m, 2H), 3.54 – 3.43 (m, 2H), 3.35 (s, 3H), 3.21 (td, J = 9.9, 5.1 Hz, 1H), 3.01 (brs, 1H), 2.10 (s, 6H), 1.43 (s, 3H), 1.35 (s, 3H), 1.33 (s, 9H), 0.86 (s, 9H), 0.06 (s, 3H), 0.05 (s, 3H); ^{13}C NMR (100 MHz, $CDCl_3$) δ 171.3, 170.3, 168.4, 155.2, 137.8, 135.0, 128.8 (2C), 128.5 (2C), 128.2, 116.4, 103.7, 99.4, 99.3, 97.0, 84.2, 79.8, 78.2, 77.9, 75.2, 74.9, 74.63, 74.59, 74.2, 74.1, 73.3, 71.5, 68.9, 64.4, 62.9, 62.0, 55.4, 55.3, 52.8, 29.2, 28.6 (3C), 25.9 (3C), 21.1, 21.1, 19.0, 18.4, -4.0, -4.9; HRMS: m/z (ESI) calcd for $C_{48}H_{76}NO_{20}Si^+$, $[M + H]^+$, 1014.4724, found 1014.4706. $^1J_{^{13}C^1-H^1}$ = 161.3 Hz, 178.3 Hz, 175.3 Hz. $^3J_{H^1-H^2}$ = 7.6 Hz.

The C1 stereochemistry was determined by measuring $^1J^{13}_{C1-H1}$ through un-decoupled HSQC experiments. The C2 stereochemistry was determined by measuring $^3J_{H^1-H^2}$ (7.6 Hz).

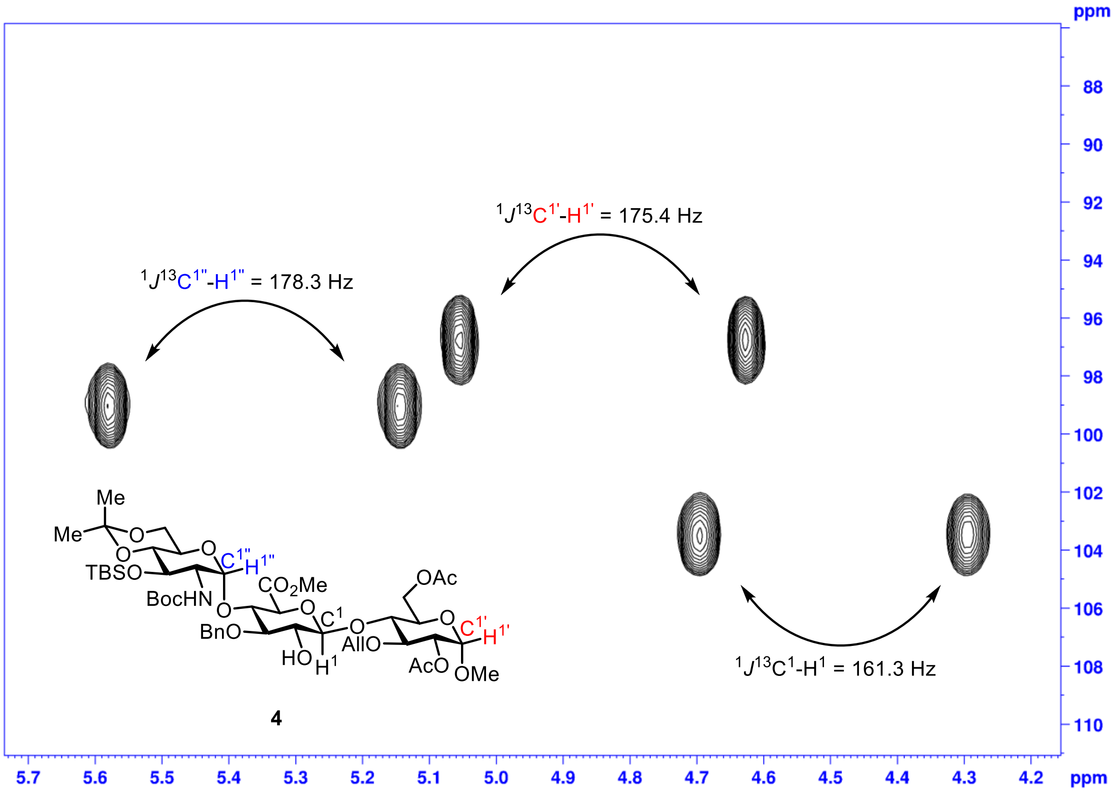

Figure S3. Un-decoupled HSQC Analysis to Determine Stereochemistry of **4**.

The 1,2-*cis*-glycoside **S7** was isolated together with **4** as an inseparable mixture in non-stereospecific glycosylation (entries 8–14).

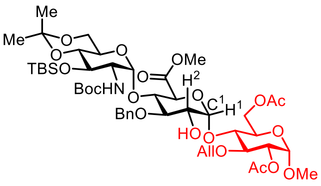

S7

Methyl 2-*tert*-butoxycarbonylamino-3-*O*-*tert*-butyldimethylsilyl-4,6-*O*-isopropylidene-2-deoxy-α-D-glucopyranosyl-(1→4)-(methyl 3-*O*-benzyl-α-D-glucopyranosyluronate)-(1→4)-2,6-di-*O*-acetyl-3-*O*-allyl-α-D-glucopyranoside (**S7**): ^{1}H NMR (400 MHz, $CDCl_3$) δ 7.37 – 7.27 (m, 5H), 5.84 (ddt, *J* = 16.2, 10.6, 5.3 Hz, 1H), 5.31 – 5.17 (m, 4H), 5.12 (d, *J* = 10.3 Hz, 1H), 5.01 (d, *J* = 9.8 Hz, 1H), 4.93 – 4.77 (m, 3H), 4.64 (d, *J* = 10.5 Hz, 1H), 4.38 – 4.17 (m, 4H), 4.14 – 3.93 (m, 2H), 3.92 – 3.70 (m, 7H), 3.75 (s, 3H), 3.69 – 3.42 (m, 3H), 3.38 (s, 3H), 3.01 (brs, 1H), 2.13 (s, 3H), 2.08 (s, 3H), 1.44 (s, 3H), 1.36 (s, 12H), 0.87 (s, 9H), 0.07 (s, 3H), 0.05 (s, 3H); ^{13}C NMR (100 MHz, $CDCl_3$) δ 170.7, 170.2, 169.1, 155.4, 137.5, 134.1, 128.7 (2C), 128.3 (2C), 128.1, 117.5, 100.1, 99.5, 99.4, 97.4, 80.1, 79.6, 79.1, 78.7, 77.6, 74.4, 73.9, 73.9, 73.6, 72.8, 71.9, 71.4, 70.2, 69.6, 68.5, 64.5, 63.1, 62.8, 62.1, 55.6, 55.4, 29.8, 28.6 (3C), 25.9 (3C), 21.2, 21.0, -3.9, -4.9; $^{1}J_{^{13}C^{1}-H^{1}}$ = 170.3 Hz, 170.5 Hz, 172.8 Hz. $^{3}J_{H^{1}-H^{2}}$ = 2.7 Hz.

b. Synthesis of the Hemin-Derived Iron(III) Porphyrin Chloride Catalyst S9

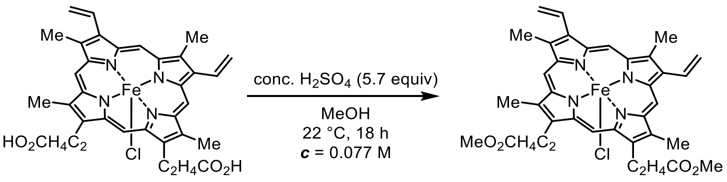

S8 **S9**

To a 100 mL oven-dried round-bottom flask equipped with a magnetic stirring bar were added hemin (1.5 g, 2.3 mmol, 1 equiv), MeOH (30 mL), and concentrated H_2SO_4 (0.7 mL, 13 mmol, 5.7 equiv). The reaction was stirred for 18 hours at room temperature. H_2O (30 mL) was added

to the reaction mixture, followed by extraction with CH_2Cl_2 (30 mL × 4). The combined organic layer was washed with water (30 mL × 2) and brine (30 mL × 1), dried over Na_2SO_4, and concentrated *in vacuo*. The residue was further dried azeotropically with anhydrous toluene (10 mL x 2) and subsequently dried *in vacuo* to afford catalyst **S9** as dark purple powder (1.49 g, 95% yield).

C. General Procedures for the Iron-Catalyzed Stereospecific Glycosylation with Glycal Epoxides

Procedure C

To a flame-dried sealable 2-dram vial equipped with a stir bar were added a glycosyl acceptor (0.24 mmol, 2.0 equiv), an iron catalyst (0.0084 mmol, 7 mol %), and freshly activated 5 Å molecular sieves, powder (*ca.* 100 mg). After the vial was evacuated and backfilled with N_2 three times, anhydrous CH_2Cl_2 (0.3 mL) was added and the solution was stirred at -40 °C for 10 min. A freshly prepared glycal epoxide (0.12 mmol, 1.0 equiv) was dissolved in anhydrous CH_2Cl_2 (0.5 mL) and transferred to the mixture at -40 °C dropwise. The reaction mixture was kept at -20 °C for 20 h and then quenched with MeOH (50 μL) and imidazole (8.5 mg in 1mL CH_2Cl_2) at the same temperature. The mixture was filtered through a piece of cotton and eluted with EtOAc (2 mL × 2). The organic layer was then concentrated *in vacuo*. The *dr* was determined based on the ^{1}H NMR analysis of the crude reaction mixture. The residue was purified through column chromatography to afford the desired glycosylation product.

Procedure D

To a flame-dried sealable 2-dram vial equipped with a stir bar were added a glycal epoxide (0.12 mmol, 1.0 equiv) and freshly activated 5 Å molecular sieves, powder (*ca.* 100 mg). After the vial was evacuated and backfilled with N_2 three times, a glycosyl acceptor (0.24 mmol, 2.0 equiv) was dissolved in anhydrous CH_2Cl_2 (0.5 mL) and was transferred to the vial dropwise. After the mixture was stirred at -78 °C for 10 min, an iron catalyst (0.0084 mmol, 7 mol %) in anhydrous CH_2Cl_2 (0.3 mL) was added at -78 °C dropwise. The reaction mixture was kept at -20 °C for 20 h and then quenched with MeOH (50 μL) and imidazole (8.5 mg in 1 mL CH_2Cl_2) at the same temperature. The mixture was filtered through a piece of cotton and eluted with EtOAc (2 mL × 2). The organic layer was then concentrated *in vacuo*. The *dr* was determined based on the ^{1}H NMR analysis of the crude reaction mixture. The residue was purified through a silica gel flash column to afford the desired glycosylation product.

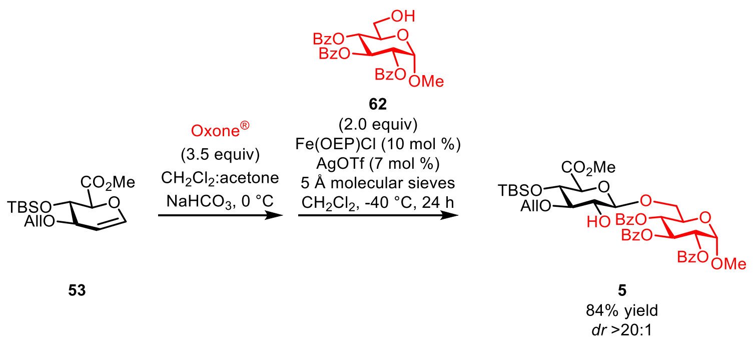

53 was synthesized according to a literature procedure.[5] **62** was synthesized according to another literature procedure.[6]

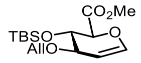

53

Methyl 3-*O*-allyl-4-*O*-*tert*-butyldimethylsilyl-D-glucuronal (**53**): $[\alpha]_D^{23}$ −1.9 (acetone, *c* = 1.0); IR ν_{max} (neat)/cm^{-1}: 2952 (w), 2928 (w), 2857 (w), 1760 (m), 1736 (w), 1649 (m), 1463 (m), 1247 (m), 1104 (s), 1064 (m), 868 (m), 837 (s), 778 (s); ^{1}H NMR (400 MHz, $CDCl_3$) δ 6.54 (d, *J* = 6.3 Hz, 1H), 5.85 (ddt, *J* = 16.1, 10.8, 5.6 Hz, 1H), 5.24 (dd, *J* = 16.1, 1.7 Hz, 1H), 5.15 (dd, *J* = 10.8, 1.7 Hz, 1H), 4.90 (ddd, *J* = 6.3, 4.8, 1.4 Hz, 1H), 4.50 (dd, *J* = 3.8, 1.1 Hz, 1H), 4.33 (td, *J* = 3.6, 1.4 Hz, 1H), 4.06 – 3.87 (m, 2H), 3.71 (s, 3H), 3.62 (t, *J* = 3.6 Hz, 1H), 0.88 (s, 9H), 0.13 (s, 3H), 0.12 (s, 3H); ^{13}C NMR (100 MHz, $CDCl_3$) δ 168.9, 144.8, 134.8, 117.2, 98.4, 76.0, 70.8, 68.8, 67.6, 52.3, 25.8 (3C), 18.2, -4.5, -4.8; HRMS: m/z (ESI) calcd for $C_{16}H_{29}O_5Si^+$, [M + H]$^+$, 329.1779, found 329.1777.

The reaction was carried out on a 0.12 mmol scale using the glycal as the limiting reagent by following **Procedure A** for epoxidation and **Procedure D** for the iron-catalyzed stereospecific glycosylation with the following modifications: glycosylation was carried out at −40 °C for 24 h. Catalyst **1d** (7 mol %) and primary acceptor **62** (0.24 mmol, 2 equiv) were used. The *dr* of the corresponding glycal α-epoxide is >20:1. The *dr* of the glycosylation product determined based on crude reaction mixture is >20:1. The desired product **5** was purified through a silica gel flash column (hexanes/EtOAc: from 20:1 to 4:1) as white foam (86.0 mg, 84% yield).

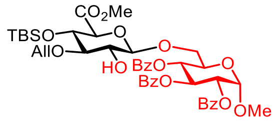

5

Methyl (methyl 3-*O*-allyl-4-*O*-*tert*-butyldimethylsilyl-β-D-glucopyranosyluronate)-(1→6)-2,3,4-tri-*O*-benzoyl-α-D-glucopyranoside (**5**): $[\alpha]_D^{23}$ +43.5 (acetone, *c* = 1.0); IR ν_{max} (neat)/cm^{-1}: 2929 (w), 2856 (w), 1725 (s), 1451 (w), 1278 (s), 1249 (s), 1134 (m), 1092 (s), 1068 (s), 1041 (s), 1026 (s), 853 (m), 837 (s), 779 (m), 706 (s); ^{1}H NMR (400 MHz, $CDCl_3$) δ 8.00 – 7.90 (m, 4H), 7.89 – 7.80 (m, 2H), 7.61 – 7.46 (m, 2H), 7.47 – 7.33 (m, 5H), 7.33 – 7.22 (m, 2H), 6.16 (t, *J* = 9.7 Hz, 1H), 5.99 (ddt, *J* = 16.3, 10.7, 5.6 Hz, 1H), 5.75 (t, *J* = 9.8 Hz, 1H), 5.37 – 5.20 (m, 3H), 5.16 (dd, *J* = 10.7, 1.7 Hz, 1H), 4.57 (dd, *J* = 11.9, 5.6 Hz, 1H), 4.32 – 4.10 (m, 4H), 3.88 (t, *J* = 8.9 Hz, 1H), 3.78 (d, *J* = 9.4 Hz, 1H), 3.74 (s, 3H), 3.71 – 3.56 (m, 2H), 3.45 (s, 3H), 3.29 (t, *J* = 8.6 Hz, 1H), 3.22 (brs, 1H), 0.83 (s, 9H), 0.10 (s, 3H), 0.01 (s, 3H); ^{13}C NMR (100 MHz, $CDCl_3$) δ 168.8, 166.1, 165.89, 165.87, 135.4, 133.8, 133.5, 133.3, 130.2 (2C), 130.1 (2C), 129.8 (2C), 129.3, 129.2, 128.8, 128.64 (2C), 128.55 (2C), 128.4 (2C), 116.7, 103.8, 97.3, 83.3, 76.8, 75.0, 73.7, 72.1, 71.7, 70.5, 69.0, 68.3, 68.1, 55.9, 52.4, 25.9 (3C), 18.1, -3.8, -5.3; HRMS: m/z (ESI) calcd for $C_{44}H_{55}O_{15}Si^+$, $[M + H]^+$, 851.3305, found 851.3327. $^1J_{^{13}C1–H1}$ = 159.6 Hz, 173.5 Hz.

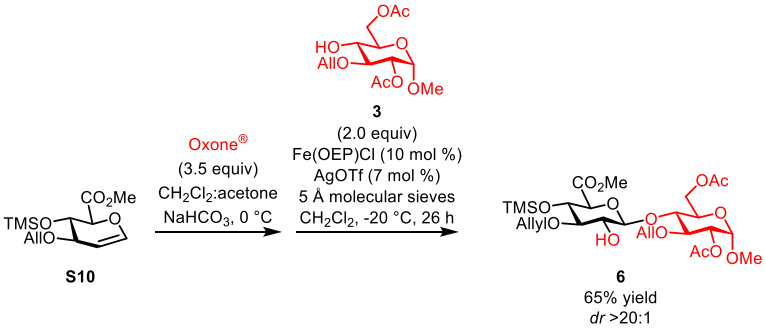

S10 was synthesized according to a literature procedure.[5]

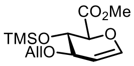

S10

Methyl 3-*O*-allyl-4-*O*-trimethylsilyl-D-glucuronal (**S10**): $[\alpha]_D^{23}$ −2.7 (acetone, *c* = 1.0); IR ν_{max} (neat)/cm^{-1}: 2954 (w), 1757 (m), 1648 (m), 1437 (w), 1249 (s), 1201 (m), 1122 (s), 1104 (s), 1063 (m), 879 (a), 839 (s), 751 (s); ^{1}H NMR (400 MHz, $CDCl_3$) δ 6.55 (d, *J* = 6.3 Hz, 1H), 5.86 (ddt, *J* = 17.3, 10.4, 5.6 Hz, 1H), 5.25 (dd, *J* = 17.3, 1.7 Hz, 1H), 5.16 (dd, *J* = 10.4, 1.7 Hz,1H), 4.92 (ddd, *J* = 6.1, 4.6, 1.3 Hz, 1H), 4.50 (dd, *J* = 4.1, 1.1 Hz, 1H), 4.31 (td, *J* = 3.9, 1.3 Hz, 1H), 4.11 – 3.89 (m, 2H), 3.72 (s, 3H), 3.65 (t, *J* = 4.1 Hz, 1H), 0.17 (s, 9H); ^{13}C NMR (100 MHz, $CDCl_3$) δ 168.9, 144.7, 134.7, 117.2, 98.8, 76.1, 71.1, 69.0, 67.7, 52.3, 0.2 (3C); HRMS: m/z (ESI) calcd for $C_{13}H_{23}O_5Si^+$, $[M + H]^+$, 287.1309, found 287.1316.

The reaction was carried out on a 0.12 mmol scale using the glycal as the limiting reagent by following **Procedure A** for epoxidation and **Procedure D** for the iron-catalyzed stereospecific glycosylation for 26 h. Catalyst **1d** (7 mol %) and secondary acceptor **3** (0.24 mmol, 2 equiv) were used. The *dr* of the corresponding glycal α-epoxide is >20:1. The *dr* of the glycosylation product determined based on crude reaction mixture is >20:1. The desired product **6** was purified through a silica gel flash column (hexanes/EtOAc: from 20:1 to 3:1) as colorless oil (48.5 mg, 65% yield).

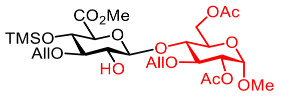

6

Methyl (methyl 3-*O*-allyl-4-*O*-trimethylsilyl-β-D-glucopyranosyluronate)-(1→4)-2,6-di-*O*-acetyl-3-*O*-allyl-α-D-glucopyranoside (**6**): $[\alpha]_D^{23}$ +36.2 (acetone, *c* = 1.0); IR ν_{max} (neat)/cm^{-1}: 3488 (w), 2929 (w), 2857 (w), 1742 (m), 1369 (w), 1233 (m), 1139 (m), 1034 (s), 916 (m), 853 (m), 837 (s), 779 (m); ^{1}H NMR (400 MHz, $CDCl_3$) δ 6.01 – 5.75 (m, 2H), 5.28 (d, *J* = 17.4 Hz, 1H), 5.25 – 5.14 (m, 2H), 5.10 (d, *J* = 10.3 Hz, 1H), 4.83 (d, *J* = 3.7 Hz, 1H), 4.77 (dd, *J* = 9.8, 3.6 Hz, 1H), 4.54 – 4.43 (m, 2H), 4.37 (dd, *J* = 11.9, 4.3 Hz, 1H), 4.42 – 4.21 (m, 3H), 4.11 (dd, *J* = 12.5, 5.7 Hz, 1H), 3.91 – 3.70 (m, 4H), 3.74 (s, 3H), 3.66 (t, *J* = 9.2 Hz, 1H), 3.50 – 3.44 (m, 1H), 3.36 (s, 3H), 3.26 – 3.16 (m, 1H), 2.98 (d, *J* = 2.9 Hz, 1H), 2.10 (s, 6H), 0.09 (s, 9H); ^{13}C NMR (100 MHz, $CDCl_3$) δ 171.3, 170.3, 168.8, 135.1, 135.0, 117.2, 116.2, 103.9, 97.0, 83.8,

78.3, 78.0, 76.5, 74.5, 74.4, 74.2, 73.4, 72.4, 68.9, 63.0, 55.3, 52.4, 21.2, 21.1, 0.4 (3C); HRMS: m/z (ESI) calcd for $C_{27}H_{45}O_{14}Si^+$, $[M + H]^+$, 621.2573, found 621.2575. $^1J_{^{13}C1–H1}$ = 171.7 Hz, 161.7 Hz.

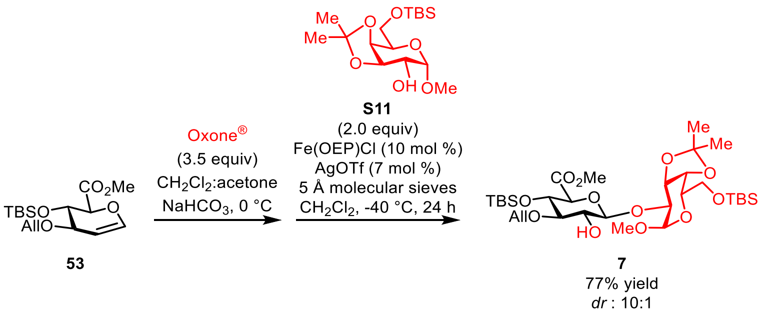

S11 was synthesized according to a literature procedure.[7]

The reaction was carried out on a 0.12 mmol scale using the glycal as the limiting reagent by following **Procedure A** for epoxidation and **Procedure D** for the iron-catalyzed stereospecific glycosylation with the modifications: glycosylation was carried out at −40 °C for 24 h. Catalyst **1d** (7 mol %) and secondary acceptor **S11** (0.24 mmol, 2 equiv) were used. The *dr* of the corresponding glycal α-epoxide is >20:1. The *dr* of the glycosylation product determined based on crude reaction mixture is 10:1. The desired product **7** was purified through a silica gel flash column (hexanes/EtOAc: from 20:1 to 4:1) as colorless oil (63.7 mg, 77% yield).

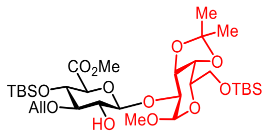

7

Methyl (methyl 3-*O*-allyl-4-*O*-*tert*-butyldimethylsilyl-β-D-glucopyranosyluronate)-(1→2)-6-*O*-*tert*-butyldimethylsilyl-3,4-*O*-isopropylidene-α-D-galactopyranoside (**7**): $[\alpha]_D^{22}$ +42.7 (acetone, *c* = 1.0); IR ν_{max} (neat)/cm^{-1}: 2953 (m), 2930 (m), 2857 (m), 2361 (w), 1757 (m), 1250 (s), 1070 (s), 1053 (s), 837 (s), 779 (s); ^1H NMR (400 MHz, $CDCl_3$) δ 6.07 – 5.83 (m, 1H), 5.25

(dd, J = 17.3, 1.8 Hz, 1H), 5.13 (dd, J = 10.5, 1.7 Hz, 1H), 4.74 (d, J = 3.5 Hz, 1H), 4.61 (d, J = 7.9 Hz, 1H), 4.49 (dd, J = 12.3, 5.5 Hz, 1H), 4.31 (dd, J = 8.2, 5.3 Hz, 1H), 4.23 – 4.10 (m, 2H), 3.97 (td, J = 6.6, 2.5 Hz, 1H), 3.91 – 3.76 (m, 5H), 3.73 (s, 3H), 3.57 (t, J = 8.4 Hz, 1H), 3.36 (s, 3H), 3.29 (t, J = 8.6 Hz, 1H), 3.01 (brs, 1H), 1.49 (s, 3H), 1.32 (s, 3H), 0.89 (s, 9H), 0.83 (s, 9H), 0.08 (s, 3H), 0.07 (s, 6H), -0.02 (s, 3H); ^{13}C NMR (100 MHz, $CDCl_3$) δ 168.9, 135.5, 116.6, 109.4, 103.6, 99.2, 83.4, 77.5, 76.7, 75.6, 74.9, 73.9, 73.6, 71.9, 68.0, 62.4, 55.5, 52.3, 28.4, 26.6, 26.0 (3C), 25.9 (3C), 18.4, 18.1, -3.8, -5.2 (two peaks overlapped, 2C), -5.4; HRMS: m/z (ESI) calcd for $C_{32}H_{61}O_{12}Si_2^+$, [M + H]$^+$, 693.3696, found 693.3716. $^1J_{^{13}C1-H1}$ = 162.5 Hz, 170.0 Hz.

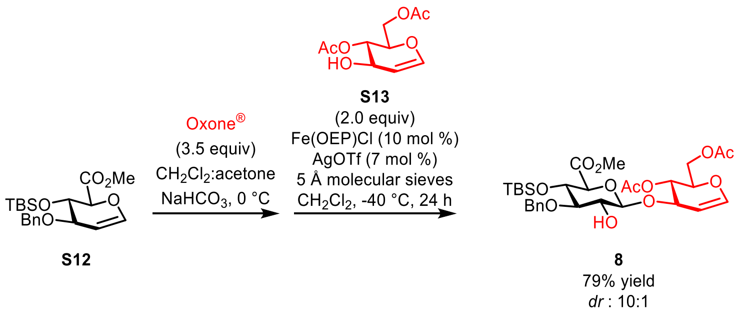

S12 was synthesized according to a literature procedure.[5] **S13** was synthesized according to another literature procedure.[8]

The reaction was carried out on a 0.12 mmol scale using the glycal as the limiting reagent by following **Procedure A** for epoxidation and **Procedure D** for the iron-catalyzed stereospecific glycosylation with the modifications: glycosylation was carried out at −40 °C for 24 h. Catalyst **1d** (7 mol %) and secondary acceptor **S13** (0.24 mmol, 2 equiv) were used. The *dr* of the corresponding glycal α-epoxide is >20:1. The *dr* of the glycosylation product determined based on crude reaction mixture is 10:1. The desired product **8** was purified through a silica gel flash column (hexanes/EtOAc: from 20:1 to 4:1) as colorless oil (59.1 mg, 79% yield).

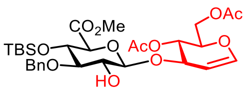

8

4,6-Di-*O*-acetyl-1,5-anhydro-3-*O*-(methyl 3-*O*-benzyl-4-*O*-*tert*-butyldimethylsilyl-β-D-glucopyranosyluronate)-2-deoxy-D-*arabino*-hex-1-enitol (**8**): $[\alpha]_D^{23}$ −18.5 (acetone, *c* = 1.5); IR ν_{max} (neat)/cm^{-1}: 3471 (w), 2954 (m), 2856 (m), 2360 (m), 1746 (s), 1649 (m), 1366 (m), 1218 (s), 1138 (s), 1042 (s), 837 (s), 779 (s); ^{1}H NMR (400 MHz, $CDCl_3$) δ 7.38 – 7.29 (m, 4H), 7.29 – 7.22 (m, 1H), 6.46 (d, *J* = 6.2 Hz, 1H), 5.23 (t, *J* = 5.4 Hz, 1H), 5.03 (d, *J* = 11.3 Hz, 1H), 4.92 (dd, *J* = 6.3, 3.6 Hz, 1H), 4.69 (d, *J* = 11.3 Hz, 1H), 4.48 (d, *J* = 7.6 Hz, 1H), 4.33 – 4.24 (m, 4H), 4.02 – 3.81 (m, 2H), 3.75 (s, 3H), 3.63 – 3.50 (m, 1H), 3.40 (t, *J* = 8.5 Hz, 1H), 2.86 (brs, 1H), 2.08 (s, 3H), 2.07 (s, 3H), 0.82 (s, 9H), -0.01 (s, 3H), -0.04 (s, 3H); ^{13}C NMR (100 MHz, $CDCl_3$) δ 171.0, 170.0, 168.9, 145.7, 138.9, 128.3 (2C), 127.6 (2C), 127.5, 100.7, 98.3, 83.9, 76.7, 74.7, 74.5, 73.9, 71.9, 71.2, 67.9, 61.6, 52.4, 25.8 (3C), 21.0, 20.9, 18.1, -3.9, -5.2; HRMS: m/z (ESI) calcd for $C_{30}H_{45}O_{12}Si^+$, $[M + H]^+$, 625.2675, found 625.2682. $^1J_{^{13}C1\text{–}H1}$ = 160.6 Hz.

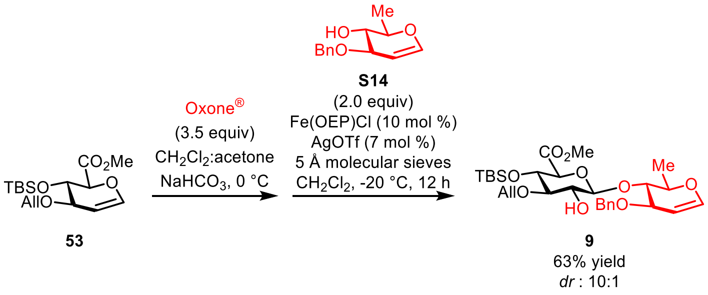

S14 was synthesized according to a literature procedure.[9]

The reaction was carried out on a 0.12 mmol scale using the glycal as the limiting reagent by following **Procedure A** for epoxidation and **Procedure D** for the iron-catalyzed stereospecific glycosylation with the modifications: glycosylation was carried out at −20 °C and 0.3 M for 24 h. Catalyst **1d** (7 mol %) and primary acceptor **S14** (0.24 mmol, 2 equiv) were used. The *dr* of the corresponding glycal α-epoxide is >20:1. The *dr* of the glycosylation product determined based on crude reaction mixture is 10:1. The desired product **9** was purified through a silica gel flash column (hexanes/EtOAc: from 20:1 to 4:1) as colorless oil (42.8 mg, 63% yield).

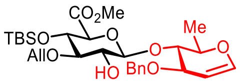

9

1,5-Anhydro-3-*O*-benzyl-2,6-dideoxy-4-*O*-(methyl 3-*O*-allyl-4-*O*-*tert*-butyldimethylsilyl-β-D-glucopyranosyluronate)-D-*arabino*-hex-1-enitol (**9**): $[\alpha]_D^{23}$ −30.6 (acetone, *c* = 0.5); IR ν_{max} (neat)/cm^{-1}: 3456 (w), 2928 (w), 2856 (w), 1752 (m), 1648 (w), 1248 (m), 1139 (m), 1058 (s), 1040 (s), 915 (m), 837 (s), 779 (s); ^{1}H NMR (400 MHz, acetone-d_6) δ 7.40 – 7.30 (m, 4H), 7.30 – 7.22 (m, 1H), 6.36 (dd, *J* = 6.2, 1.4 Hz, 1H), 6.04 – 5.91 (m, 1H), 5.24 (dd, *J* = 17.4, 1.9 Hz, 1H), 5.09 (dd, *J* = 10.5, 1.8 Hz, 1H), 4.83 (dd, *J* = 6.2, 3.2 Hz, 1H), 4.73 (d, *J* = 7.8 Hz, 1H), 4.69 – 4.55 (m, 4H), 4.18 (dd, *J* = 12.5, 5.8 Hz, 1H), 4.10 – 3.97 (m, 2H), 3.91 (d, *J* = 9.5 Hz, 1H), 3.86 – 3.74 (m, 2H), 3.65 (s, 3H), 3.48 – 3.40 (m, 1H), 3.31 (t, *J* = 8.7 Hz, 1H), 1.41 (d, *J* = 6.6 Hz, 3H), 0.84 (s, 9H), 0.11 (s, 3H), 0.02 (s, 3H); ^{13}C NMR (100 MHz, acetone-d_6) δ 169.7, 145.1, 140.3, 136.7, 129.0 (2C), 128.4 (2C), 128.0, 116.0, 104.3, 101.2, 84.6, 80.1, 77.0, 75.8, 74.6, 74.3, 74.3, 73.0, 71.0, 52.4, 26.2 (3C), 18.6, 17.4, -3.6, -5.0; HRMS: m/z (ESI) calcd for $C_{29}H_{45}O_9Si^+$, $[M + H]^+$, 565.2827, found 565.2833. $^1J_{^{13}C1-H1}$ = 160.8 Hz.

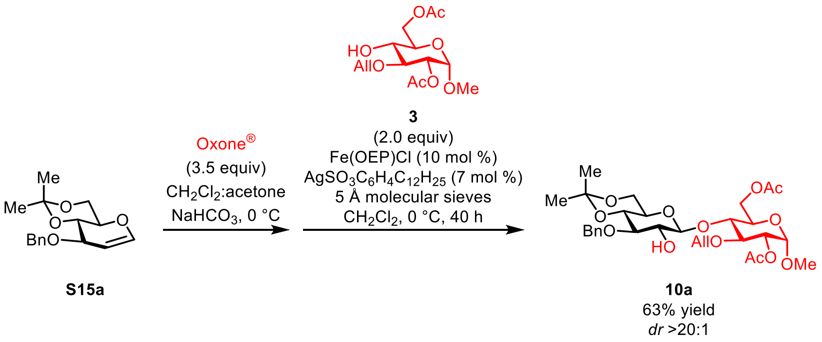

S15a was synthesized according to a literature procedure.[10]

The reaction was carried out on a 0.12 mmol scale using the glycal as the limiting reagent by following **Procedure A** for epoxidation and **Procedure D** for the iron-catalyzed stereospecific glycosylation with the modifications: glycosylation was carried out at 0 °C and 0.3 M for 40 h. Catalyst **1f** (7 mol %) and secondary acceptor **3** (0.24 mmol, 2 equiv) were used. The *dr* of the

corresponding glycal α-epoxide is 16:1. The *dr* of the glycosylation product determined based on crude reaction mixture is >20:1. The desired product **10a** was purified through a silica gel flash column (hexanes/EtOAc: from 20:1 to 2:1) as white foam (46.1 mg, 63% yield).

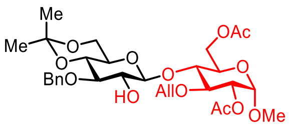

10a

Methyl 3-*O*-benzyl-4,6-*O*-isopropylidene-β-D-glucopyranosyl-(1→4)-2,6-di-*O*-acetyl-3-*O*-allyl-α-D-glucopyranoside (**10a**): $[\alpha]_D^{23}$ +88.6 (acetone, *c* = 1.0); IR ν_{max} (neat)/cm^{-1}: 2905 (w), 1739 (s), 1369 (m), 1232 (s), 1173 (m), 1031 (s), 916 (m), 856 (m), 754 (m), 698 (m); ^{1}H NMR (400 MHz, $CDCl_3$) δ7.39 – 7.27 (m, 5H), 5.85 (ddt, *J* = 17.2, 10.5, 5.2 Hz, 1H), 5.22 (dd, *J* = 17.2, 1.8 Hz, 1H), 5.12 (dd, *J* = 10.5, 1.8 Hz, 1H), 4.89 (d, *J* = 11.7 Hz, 1H), 4.84 (d, *J* = 3.6 Hz, 1H), 4.77 (dd, *J* = 9.9, 3.6 Hz, 1H), 4.71 (d, *J* = 11.7 Hz, 1H), 4.53 (dd, *J* = 5.5, 2.2 Hz, 1H), 4.46 (dd, *J* = 12.1, 2.1 Hz, 1H), 4.39 (dd, *J* = 12.1, 5.2 Hz, 1H), 4.28 (dd, *J* = 12.1, 5.2 Hz, 1H), 4.14 (dd, *J* = 12.6, 5.5 Hz, 1H), 3.89 (dd, *J* = 10.8, 5.3 Hz, 1H), 3.86 – 3.78 (m, 2H), 3.77 – 3.61 (m, 3H), 3.49 – 3.41 (m, 2H), 3.35 (s, 3H), 3.21 (td, *J* = 9.9, 5.3 Hz, 1H), 2.77 (brs, 1H), 2.10 (s, 3H), 2.08 (s, 3H), 1.46 (s, 3H), 1.41 (s, 3H); ^{13}C NMR (100 MHz, $CDCl_3$) δ171.1, 170.3, 138.7, 135.0, 128.5 (2C), 128.0 (2C), 127.9, 116.1, 104.2, 99.4, 97.0, 81.2, 78.4, 78.3, 74.6, 74.5, 74.2, 74.1, 73.3, 68.7, 67.5, 62.9, 62.3, 55.3, 29.2, 21.13, 21.05, 19.2; HRMS: m/z (ESI) calcd for $C_{30}H_{43}O_{13}^+$, $[M + H]^+$, 611.2698, found 611.2692. $^{1}J_{^{13}C1–H1}$ = 174.0 Hz, 162.0 Hz.

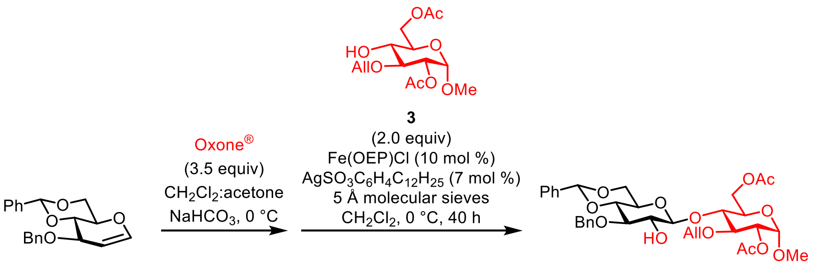

S15b

10b
58% yield
dr >20:1

S15b was synthesized according to a literature procedure.[11]

The reaction was carried out on a 0.12 mmol scale using the glycal as the limiting reagent by following **Procedure A** for epoxidation and **Procedure D** for the iron-catalyzed stereospecific glycosylation with the modifications: glycosylation was carried out at 0 °C and 0.3 M for 40 h. Catalyst **1f** (7 mol %) and secondary acceptor **3** (0.24 mmol, 2 equiv) were used. The *dr* of the corresponding glycal α-epoxide is 10:1. The *dr* of the glycosylation product determined based on crude reaction mixture is >20:1. The desired product **10b** was purified through a silica gel flash column (CH_2Cl_2/EtOAc: from 20:1 to 3:1) as white foam (45.8 mg, 58% yield).

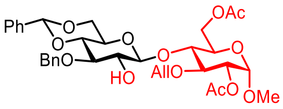

10b

Methyl 3-*O*-benzyl-4,6-*O*-benzylidene-β-D-glucopyranosyl-(1→4)-2,6-di-*O*-acetyl-3-*O*-allyl-α-D-glucopyranoside (**10b**): $[\alpha]_D^{23}$ +33.8 (acetone, *c* = 0.33); IR ν_{max} (neat)/cm^{-1}: 2923 (m), 1741 (s), 1645 (m), 1370 (m), 1236 (s), 1071 (s), 1042 (s), 916 (w), 698 (m); ^{1}H NMR (400 MHz, $CDCl_3$) δ 7.48 (dd, *J* = 7.3, 2.5 Hz, 2H), 7.41 – 7.28 (m, 8H), 5.86 (ddd, *J* = 17.4, 10.6, 5.2 Hz, 1H), 5.56 (s, 1H), 5.24 (d, *J* = 17.4, 1.8 Hz, 1H), 5.14 (dd, *J* = 10.6, 1.8 Hz, 1H), 4.98 (d, *J* = 11.5 Hz, 1H), 4.85 (d, *J* = 3.6 Hz, 1H), 4.83 – 4.71 (m, 2H), 4.60 (d, *J* = 7.7 Hz, 1H), 4.49 (dd, *J* = 12.0, 2.1 Hz, 1H), 4.40 (dd, *J* = 12.0, 4.4 Hz, 1H), 4.36 – 4.26 (m, 2H), 4.17 (dd, *J* = 12.6, 5.2 Hz, 1H), 3.90 – 3.79 (m, 2H), 3.79 – 3.69 (m, 2H), 3.69 – 3.59 (m, 2H), 3.51 (td, *J* = 8.1, 2.5 Hz, 1H), 3.46 – 3.39 (m, 1H), 3.37 (s, 3H), 2.81 (d, *J* = 2.6 Hz, 1H), 2.12 (s, 3H), 2.10 (s, 3H); ^{13}C NMR (100 MHz, $CDCl_3$) δ 171.2, 170.3, 138.4, 137.3, 135.0, 129.2, 128.6 (2C), 128.4 (2C), 128.2 (2C), 128.0, 126.1 (2C), 116.1, 104.2, 101.4, 97.0, 81.5, 80.8, 78.4, 78.3, 74.9, 74.8, 74.1, 73.3, 68.9, 68.7, 66.6, 63.0, 55.3, 21.2, 21.1; HRMS: m/z (ESI) calcd for $C_{34}H_{43}O_{13}^+$, $[M + H]^+$, 659.2698, found 659.2711. $^1J_{^{13}C1–H1}$ = 161.3 Hz, 172.2 Hz.

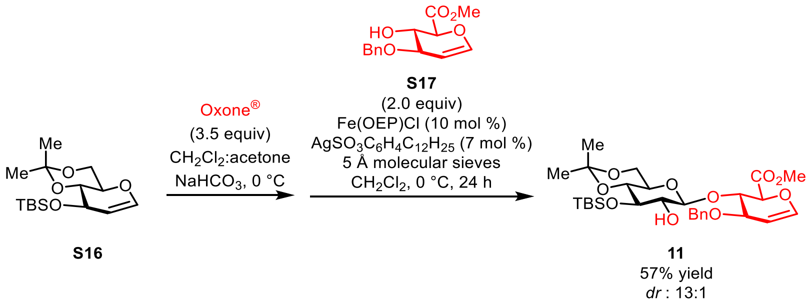

S16 was synthesized according to a literature procedure.[12] **S17** was synthesized according to another literature procedure.[5]

The reaction was carried out on a 0.3 mmol scale using the glycal as the limiting reagent by following **Procedure A** for epoxidation and **Procedure D** for the iron-catalyzed stereospecific glycosylation with the modifications: glycosylation was carried out at 0 °C and 0.3 M for 24 h. Catalyst **1f** (7 mol %) and secondary acceptor **S17** (0.6 mmol, 2 equiv) were used. The *dr* of the corresponding glycal α-epoxide is 15:1. The *dr* of the glycosylation product determined based on crude reaction mixture is 13:1. The desired product **11** was purified through a silica gel flash column (hexanes/acetone: from 20:1 to 5:1) as white foam (247.3 mg, 57% yield).

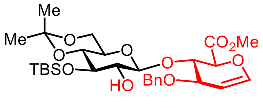

11

Methyl 3-*O*-benzyl-4-*O*-(3-*O*-*tert*-butyldimethylsilyl-4,6-*O*-isopropylidene-β-D-glucopyranosyl)-D-glucuronal (**11**): $[\alpha]_D^{23}$ −27.0 (acetone, *c* = 1.0); IR ν_{max} (neat)/cm^{-1}: 2928 (w), 2855 (w), 1764 (m), 1651 (m), 1456 (m), 1247 (m), 1203 (m), 1086 (s), 1059 (s), 836 (s), 779 (s); ^{1}H NMR (400 MHz, $CDCl_3$) δ 7.37 – 7.25 (m, 5H), 6.61 (d, *J* = 6.3 Hz, 1H), 4.99 (t, *J* = 5.9 Hz, 1H), 4.79 (t, *J* = 1.8 Hz, 1H), 4.57 – 4.48 (m, 2H), 4.47 – 4.38 (m, 2H), 3.92 – 3.79 (m, 2H), 3.71 (t, *J* = 10.5 Hz, 1H), 3.56 (s, 3H), 3.55 (t, *J* = 8.9 Hz, 1H), 3.44 (t, *J* = 9.2 Hz, 1H), 3.36 (t, *J* = 8.1 Hz, 1H), 3.22 (td, *J* = 9.9, 5.3 Hz, 1H), 2.38 (brs, 1H), 1.43 (s, 3H), 1.38 (s, 3H), 0.87 (s, 9H), 0.08 (s, 3H), 0.06 (s, 3H); ^{13}C NMR (100 MHz, $CDCl_3$) δ 168.3, 145.3, 138.0, 128.5 (2C), 128.0 (2C), 127.9, 103.2, 99.5, 98.6, 75.2, 74.9, 73.7, 73.5, 72.9, 69.7, 67.9, 67.8,

62.2, 52.4, 29.1, 25.9 (3C), 19.1, 18.5, -4.2, -4.7; HRMS: m/z (ESI) calcd for $C_{29}H_{45}O_{10}Si^+$, [M + H]$^+$, 581.2777, found 581.2771. $^1J^{13}{}_{C1–H1}$ = 161.5 Hz.

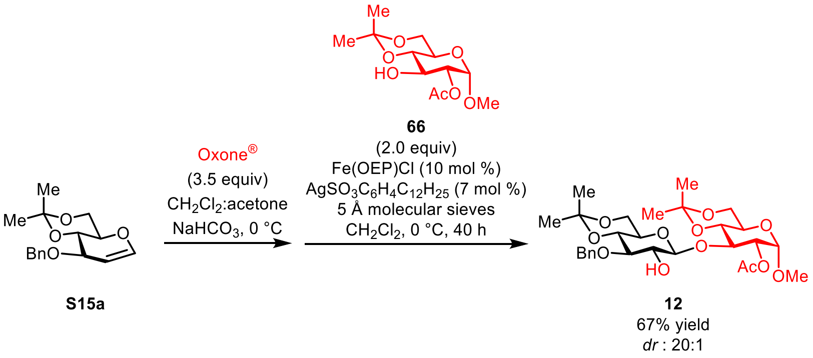

The reaction was carried out on a 0.12 mmol scale using the glycal as the limiting reagent by following **Procedure A** for epoxidation and **Procedure D** for the iron-catalyzed stereospecific glycosylation with the modifications: glycosylation was carried out at 0 °C and 0.3 M for 40 h. Catalyst **1f** (7 mol %) and secondary acceptor **66** (0.24 mmol, 2 equiv) were used. The *dr* of the corresponding glycal α-epoxide is 16:1. The *dr* of the glycosylation product determined based on crude reaction mixture is 20:1. The desired product **12** was purified through a silica gel flash column (hexanes/EtOAc: from 20:1 to 2:1) as white foam (45.6 mg, 67% yield).

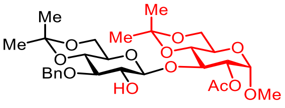

12

Methyl 3-*O*-benzyl-4,6-*O*-isopropylidene-β-D-glucopyranosyl-(1→3)-2-*O*-acetyl-4,6-*O*-isopropylidene-α-D-glucopyranoside (**12**): $[\alpha]_D^{23}$ +54.9 (acetone, *c* = 1.0); IR ν_{max} (neat)/cm^{-1}: 2912 (w), 2359 (w), 1743 (m), 1372 (m), 1236 (m), 1198 (m), 1093 (s), 1054 (s), 854 (s), 698 (m); ^{1}H NMR (400 MHz, $CDCl_3$) δ 7.46 – 7.18 (m, 5H), 4.90 (dd, *J* = 9.7, 3.8 Hz, 1H), 4.88 – 4.76 (m, 3H), 4.48 (d, *J* = 7.9 Hz, 1H), 4.09 (t, *J* = 9.2 Hz, 1H), 3.90 – 3.84 (m, 2H), 3.79 – 3.73 (m, 2H), 3.72 – 3.64 (m, 3H), 3.53 (td, *J* = 8.3, 2.3 Hz, 1H), 3.45 (t, *J* = 8.8 Hz, 1H), 3.37 (s, 3H), 3.21 (td, *J* = 10.0, 5.3 Hz, 1H), 2.96 (d, *J* = 2.4 Hz, 1H), 2.07 (s, 3H), 1.50 (s, 3H), 1.46 (s, 3H), 1.43 (s, 3H), 1.41 (s, 3H); ^{13}C NMR (100 MHz, $CDCl_3$) δ 170.5, 139.1, 128.4 (2C), 127.9 (2C),

127.6, 102.6, 100.1, 99.5, 98.0, 80.7, 74.9, 74.3, 74.0, 73.5, 72.8, 72.1, 67.8, 63.3, 62.44, 62.37, 55.4, 29.3, 29.1, 21.2, 19.3, 19.2; HRMS: m/z (ESI) calcd for $C_{28}H_{41}O_{12}^{+}$, $[M + H]^{+}$, 569.2593, found 569.2605. ${}^{1}J_{{}^{13}C1\text{–}H1}$ = 162.4 Hz, 173.2 Hz.

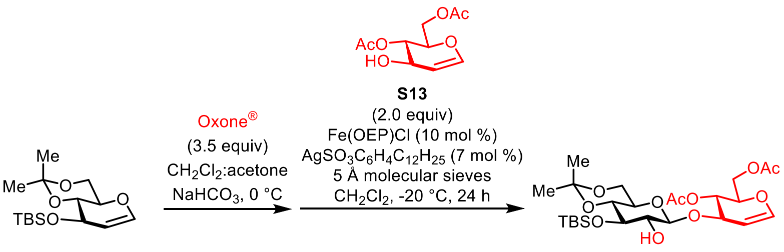

S16

13
72% yield
dr : 15:1

The reaction was carried out on a 0.12 mmol scale using the glycal as the limiting reagent by following **Procedure A** for epoxidation and **Procedure D** for the iron-catalyzed stereospecific glycosylation with the modifications: glycosylation was carried out at 0.3 M for 24 h. Catalyst **1f** (7 mol %) and secondary acceptor **S13** (0.24 mmol, 2 equiv) were used. The *dr* of the corresponding glycal α-epoxide is 15:1. The *dr* of the glycosylation product determined based on crude reaction mixture is 15:1. The desired product **13** was purified through a silica gel flash column (hexanes/EtOAc: from 20:1 to 3:1) as white foam (47.1 mg, 72% yield).

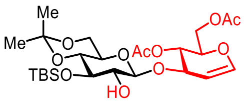

13

4,6-Di-*O*-acetyl-1,5-anhydro-3-*O*-(3-*O*-*tert*-butyldimethylsilyl-4,6-*O*-isopropylidene-β-D-glucopyranosyl)-2-deoxy-D-*arabino*-hex-1-enitol (**13**): $[\alpha]_D^{23}$ −29.8 (acetone, *c* = 1.0); IR ν_{max} (neat)/cm^{-1}: 2929 (w), 2856 (w), 2360 (w), 1745 (s), 1650 (w), 1370 (m), 1226 (s), 1089 (s), 1059 (s), 836 (s), 779 (s); ^{1}H NMR (400 MHz, $CDCl_3$) δ 6.57 – 6.35 (d, *J* = 6.3 Hz, 1H), 5.24 (t, *J* = 4.8 Hz, 1H), 4.93 (dd, *J* = 6.3, 3.8 Hz, 1H), 4.46 (d, *J* = 7.8 Hz, 1H), 4.39 – 4.25 (m, 3H), 4.23 (t, *J* = 4.3 Hz, 1H), 3.88 (dd, *J* = 10.7, 5.3 Hz, 1H), 3.72 (t, *J* = 10.5 Hz, 1H), 3.54 (t, *J* = 8.7 Hz, 1H), 3.45 (t, *J* = 9.2 Hz, 1H), 3.32 (td, *J* = 8.1, 3.0 Hz, 1H), 3.21 (td, *J* = 9.9, 5.3 Hz, 1H),

2.64 (d, J = 3.0 Hz, 1H), 2.08 (s, 3H), 2.07 (s, 3H), 1.45 (s, 3H), 1.38 (s, 3H), 0.88 (s, 9H), 0.09 (s, 3H), 0.07 (s, 3H); ^{13}C NMR (100 MHz, $CDCl_3$) δ 170.9, 169.8, 145.3, 101.5, 99.5, 98.5, 75.3, 75.1, 73.9, 73.6, 70.9, 68.2, 67.5, 62.4, 61.8, 29.1, 25.9 (3C), 21.1, 21.0, 19.1, 18.5, -4.3, -4.7; HRMS: m/z (ESI) calcd for $C_{25}H_{43}O_{11}Si^+$, $[M + H]^+$, 547.2569, found 547.2566. $^1J_{^{13}C1–H1}$ = 158.4 Hz.

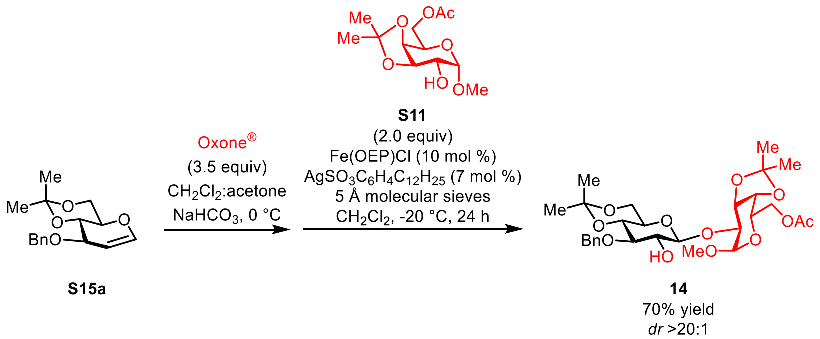

The reaction was carried out on a 0.12 mmol scale using the glycal as the limiting reagent by following the **Procedure A** for epoxidation and **Procedure D** for the iron-catalyzed stereospecific glycosylation with the modifications: glycosylation was carried out at 0.3 M for 24 h. Catalyst **1f** (7 mol %) and secondary acceptor **S11** (0.24 mmol, 2 equiv) were used. The *dr* of the corresponding glycal α-epoxide is 16:1. The *dr* of the glycosylation product determined based on crude reaction mixture is >20:1. The desired product **14** was purified through a silica gel flash column (hexanes/EtOAc: from 20:1 to 3:2) as white foam (47.7 mg, 70% yield).

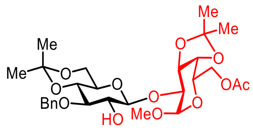

14

Methyl 3-*O*-benzyl-4,6-*O*-isopropylidene-β-D-glucopyranosyl-(1→2)-6-*O*-acetyl-3,4-*O*-isopropylidene-α-D-galactopyranoside (**14**): $[\alpha]_D^{23}$ +27.3 (acetone, c = 1.0); IR ν_{max} (neat)/cm^{-1}: 2922 (w), 2359 (w), 1741 (m), 1370 (m), 1235 (s), 1198 (m), 1062 (s), 1027 (s), 856 (m), 736 (m), 698 (m); ^{1}H NMR (400 MHz, $CDCl_3$) δ 7.40 – 7.35 (d, J = 7.3 Hz, 2H), 7.35 – 7.29 (t, J =

7.4 Hz, 2H), 7.29 – 7.24 (m, 1H), 4.83 (ABq, $\Delta\nu_{AB}$ = 20.6 Hz, J_{AB} = 11.9 Hz, 2H), 4.79 (d, J = 3.0 Hz, 1H), 4.59 (d, J = 7.6 Hz, 1H), 4.39 – 4.29 (m, 3H), 4.24 – 4.11 (m, 2H), 3.86 (dd, J = 10.8, 5.4 Hz, 1H), 3.84 – 3.72 (m, 2H), 3.70 (t, J = 9.3 Hz, 1H), 3.59 (t, J = 8.3 Hz, 1H), 3.51 (t, J = 8.8 Hz, 1H), 3.39 (s, 3H), 3.24 (td, J = 10.0, 5.5 Hz, 1H), 2.75 (brs, 1H), 2.09 (s, 3H), 1.50 (s, 3H), 1.46 (s, 3H), 1.41 (s, 3H), 1.33 (s, 3H); ^{13}C NMR (100 MHz, $CDCl_3$) δ 170.9, 139.1, 128.4 (2C), 127.9 (2C), 127.6, 109.8, 104.6, 99.6, 99.2, 80.8, 78.1, 75.3, 74.41, 74.38, 73.9, 73.8, 67.6, 65.8, 63.7, 62.3, 55.7, 29.3, 28.3, 26.5, 21.0, 19.2; HRMS: m/z (ESI) calcd for $C_{28}H_{41}O_{12}^+$, [M + H]$^+$, 569.2593, found 569.2588. $^1J_{^{13}C1\text{–}H1}$ = 162.2 Hz, 171.8 Hz.

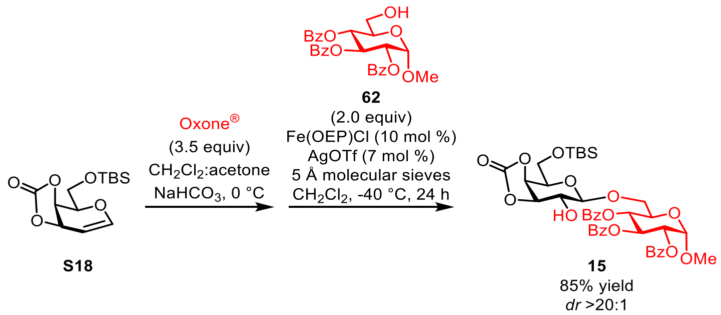

S18 was synthesized according to a literature procedure.[13]

The reaction was carried out on a 0.12 mmol scale using the glycal as the limiting reagent by following **Procedure A** for epoxidation and **Procedure C** for the iron-catalyzed stereospecific glycosylation with the modifications: glycosylation was carried out at −40 °C for 24 h. Catalyst **1d** (7 mol %) and primary acceptor **62** (0.24 mmol, 2 equiv) were used. The *dr* of the corresponding glycal α-epoxide is >20:1. The *dr* of the glycosylation product determined based on crude reaction mixture is >20:1. The desired product **15** was purified through a silica gel flash column (hexanes/EtOAc: from 20:1 to 2:1) as white foam (82.4 mg, 85% yield).

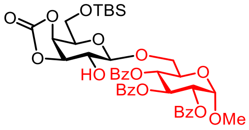

15

Methyl 6-*O*-*tert*-butyldimethylsilyl-3,4-*O*-carbonyl-β-D-galactopyranosyl-(1→6)-2,3,6-tri-*O*-benzoyl-α-D-glucopyranoside (**15**): $[\alpha]_D^{23}$ +35.7 (acetone, *c* = 1.0); IR ν_{max} (neat)/cm^{-1}: 3502 (w), 2929 (w), 2856 (w), 2360 (w), 1805 (m), 1728 (s), 1365 (m), 1262 (s), 1093 (s), 1027 (s), 839 (m), 709 (s); ^{1}H NMR (400 MHz, $CDCl_3$) δ 8.00 – 7.92 (m, 4H), 7.93 – 7.80 (m, 2H), 7.59 – 7.46 (m, 2H), 7.42 – 7.34 (m, 5H), 7.30 – 7.24 (m, 2H), 6.18 (t, *J* = 10.2 Hz, 1H), 5.81 (t, *J* = 9.9 Hz, 1H), 5.45 – 5.20 (m, 2H), 4.76 (d, *J* = 7.0 Hz, 1H), 4.68 (t, *J* = 6.8 Hz, 1H), 4.29 – 4.17 (m, 3H), 3.93 – 3.83 (m, 3H), 3.80 (t, *J* = 6.4 Hz, 1H), 3.73 – 3.62 (m, 2H), 3.46 (s, 3H), 0.88 (s, 9H), 0.08 (s, 6H); ^{13}C NMR (100 MHz, $CDCl_3$) δ 166.2, 165.9, 165.8, 154.1, 134.0, 133.5, 133.3, 130.1 (2C), 130.0 (2C), 129.7 (2C), 129.14, 129.11, 128.7 (2C), 128.6, 128.5 (2C), 128.4 (2C), 102.1, 97.4, 79.1, 74.4, 73.1, 72.6, 72.1, 70.4, 68.9, 68.1, 67.7, 61.3, 55.9, 25.9 (3C), 18.3, -5.4 (2C); HRMS: m/z (ESI) calcd for $C_{41}H_{49}O_{15}Si^+$, $[M + H]^+$, 809.2935, found 809.2902. $^1J_{^{13}C1-H1}$ = 160.0 Hz, 175.2 Hz.

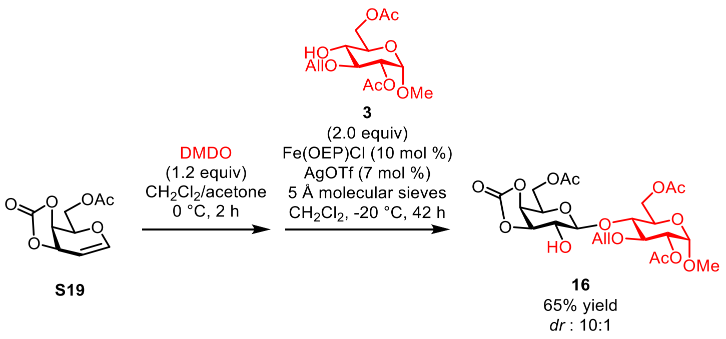

S19 was synthesized according to a literature procedure.[13]

The reaction was carried out on a 0.12 mmol scale using the glycal as the limiting reagent by following **Procedure B** for epoxidation and **Procedure C** for the iron-catalyzed stereospecific glycosylation with the modifications: DMDO (1.2 equiv) was used. Catalyst **1d** (7 mol %) and secondary acceptor **3** (0.24 mmol, 2 equiv) were used. The *dr* of the corresponding glycal α-epoxide is >20:1. The *dr* of the glycosylation product determined based on crude reaction mixture is 10:1. The desired product **16** was purified through a silica gel flash column (hexanes/acetone: from 20:1 to 3:2) as colorless oil (42.7 mg, 65% yield).

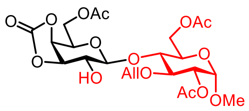

16

Methyl 6-*O*-acetyl-3,4-*O*-carbonyl-β-ᴅ-galactopyranosyl-(1→4)-2,6-di-*O*-acetyl-3-*O*-allyl-α-ᴅ-glucopyranoside (**16**): $[\alpha]_D^{23}$ +60.7 (acetone, *c* = 0.4); IR ν_{max} (neat)/cm^{-1}: 2923 (w), 2360 (m), 2342 (w), 1804 (m), 1737 (s), 1369 (m), 1232 (s), 1162 (m), 1033 (s), 692 (m); ^{1}H NMR (400 MHz, $CDCl_3$) δ 5.87 (ddt, *J* = 16.3, 10.6, 5.4 Hz, 1H), 5.24 (d, *J* = 16.3 Hz, 1H), 5.14 (d, *J* = 10.6 Hz, 1H), 4.85 (d, *J* = 3.7 Hz, 1H), 4.82 – 4.67 (m, 3H), 4.59 (d, *J* = 6.7 Hz, 1H), 4.44 – 4.37 (m, 2H), 4.37 – 4.28 (m, 2H), 4.25 (dd, *J* = 12.8, 5.4 Hz, 1H), 4.17 (dd, *J* = 12.8, 5.4 Hz, 1H), 4.05 (t, *J* = 6.1 Hz, 1H), 3.96 – 3.74 (m, 3H), 3.67 (d, *J* = 9.2 Hz, 1H), 3.63 (d, *J* = 3.2 Hz, 1H), 3.37 (s, 3H), 2.12 (s, 6H), 2.08 (s, 3H); ^{13}C NMR (100 MHz, $CDCl_3$) δ 171.5, 170.5, 170.3, 153.7, 134.8, 116.7, 102.3, 97.0, 78.4, 77.9, 77.8, 74.24, 74.17, 73.3, 71.3, 70.0, 68.8, 62.9, 62.2, 55.5, 21.2, 21.1, 20.9; HRMS: m/z (ESI) calcd for $C_{23}H_{33}O_{15}^+$, $[M + H]^+$, 549.1814, found 539.1813. $^1J_{^{13}C1\text{–}H1}$ = 163.1 Hz, 174.3 Hz.

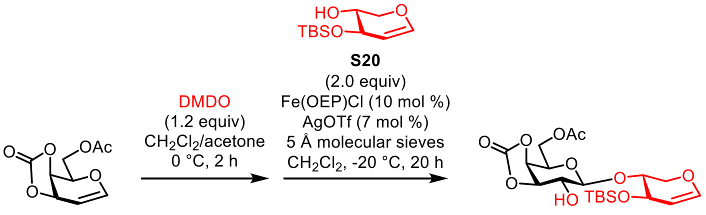

S19

17
73% yield
dr >20:1

S20 was synthesized according to a literature procedure.[1]

The reaction was carried out on a 0.12 mmol scale using the glycal as the limiting reagent by following **Procedure B** for epoxidation and **Procedure C** for the iron-catalyzed stereospecific glycosylation with the modifications: DMDO (1.2 equiv) was used. Catalyst **1d** (7 mol %) and secondary acceptor **S20** (0.24 mmol, 2 equiv) were used. The *dr* of the corresponding glycal α-epoxide is >20:1. The *dr* of the glycosylation product determined based on crude reaction

mixture is >20:1. The desired product **17** was purified through a silica gel flash column (hexanes/acetone: from 20:1 to 2:1) as colorless oil (40.3 mg, 73% yield).

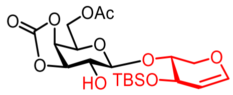

17

4-*O*-(6-*O*-Acetyl-3,4-*O*-carbonyl-β-D-galactopyranosyl)-1,5-anhydro-3-*O*-*tert*-butyldimethylsilyl-2-deoxy-D-*threo*-pent-1-enitol (**17**): $[\alpha]_D^{22}$ +48.1 (acetone, *c* = 1.0); IR ν_{max} (neat)/cm^{-1}: 2955 (w), 2929 (w), 1800 (s), 1745 (m), 1644 (m), 1370 (m), 1240 (s), 1060 (s), 1034 (s), 835 (s), 775 (s); ^{1}H NMR (400 MHz, $CDCl_3$) δ 6.43 (d, *J* = 6.1 Hz, 1H), 4.83 (t, *J* = 5.6 Hz, 1H), 4.77 (d, *J* = 6.9 Hz, 1H), 4.74 – 4.66 (m, 1H), 4.60 (d, *J* = 6.2 Hz, 1H), 4.37 (dd, *J* = 11.5, 6.5 Hz, 1H), 4.30 (dd, *J* = 11.6, 5.6 Hz, 1H), 4.16 – 4.05 (m, 2H), 4.02 – 3.97 (m, 1H), 3.94 (d, *J* = 12.1 Hz, 1H), 3.88 – 3.74 (m, 2H), 2.91 (brs, 1H), 2.10 (s, 3H), 0.89 (s, 9H), 0.10 (s, 6H); ^{13}C NMR (100 MHz, $CDCl_3$) δ 170.6, 153.7, 145.5, 102.2, 99.0, 77.5, 74.7, 74.1, 70.9, 69.6, 62.5, 62.4, 62.0, 25.9 (3C), 20.9, 18.2, -4.3, -4.5; HRMS: m/z (ESI) calcd for $C_{20}H_{33}O_{10}Si^+$, $[M + H]^+$, 461.1838, found 461.1828. $^1J_{^{13}C1\text{–}H1}$ = 162.0 Hz.

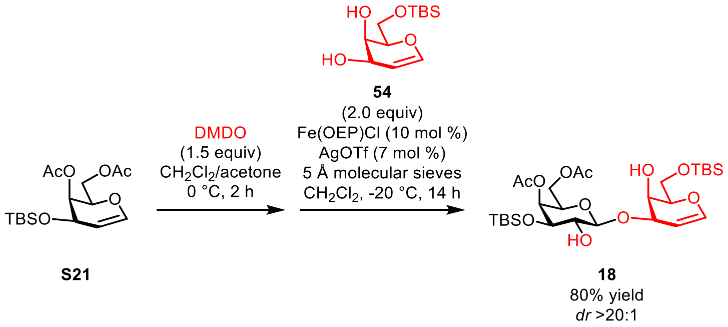

S21 was synthesized according to a literature procedure.[1] **54** was synthesized according to another literature procedure.[14]

The reaction was carried out on a 0.12 mmol scale using the glycal as the limiting reagent by following **Procedure B** for epoxidation and **Procedure D** for the iron-catalyzed stereospecific

glycosylation with the modifications: glycosylation was carried out at 0.3 M for 14 h. Catalyst **1d** (7 mol %) and diol acceptor **54** (0.24 mmol, 2 equiv) were used. The *dr* of the corresponding glycal α-epoxide is >20:1. The *dr* of the glycosylation product determined based on crude reaction mixture is >20:1. The desired product **18** was purified through a silica gel flash column (hexanes/EtOAc: from 20:1 to 4:1) as a colorless oil (59.6 mg, 80% yield).

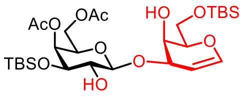

18

3-*O*-(4,6-Di-*O*-acetyl-3-*O*-*tert*-butyldimethylsilyl-β-D-galactopyranosyl)-1,5-anhydro-6-*O*-*tert*-butyldimethylsilyl-2-deoxy-D-*lyxo*-hex-1-enitol (**18**): $[\alpha]_D^{22}$ –9.6 (acetone, c = 1.0); IR ν_{max} (neat)/cm^{-1}: 2928 (m), 2857 (m), 1747 (s), 1472 (w), 1370 (m), 1234 (s), 1112 (s), 1069 (s), 835 (s), 777 (s); ^1H NMR (400 MHz, $CDCl_3$) δ 6.43 (dd, J = 6.3, 1.8 Hz, 1H), 5.21 (dd, J = 3.2, 1.2 Hz, 1H), 4.71 (dt, J = 6.3, 2.0 Hz, 1H), 4.57 – 4.48 (m, 2H), 4.27 – 4.01 (m, 3H), 3.98 – 3.80 (m, 4H), 3.73 – 3.62 (m, 2H), 3.24 (d, J = 2.9 Hz, 1H), 2.61 (d, J = 2.4 Hz, 1H), 2.09 (s, 3H), 2.05 (s, 3H), 0.89 (s, 9H), 0.85 (s, 9H), 0.10 (s, 3H), 0.08 (s, 9H); ^{13}C NMR (100 MHz, $CDCl_3$) δ 170.7, 170.2, 145.9, 101.9, 98.9, 76.8, 73.8, 72.9, 72.0, 71.4, 69.6, 64.3, 62.4, 62.3, 26.0 (3C), 25.7 (3C), 20.9, 20.8, 18.5, 18.2, -4.6, -4.9, -5.2, -5.3; HRMS: m/z (ESI) calcd for $C_{28}H_{53}O_{11}Si_2^+$, $[M + H]^+$, 621.3121, found 621.3111. $^1J_{^{13}C1–H1}$ = 158.3 Hz.

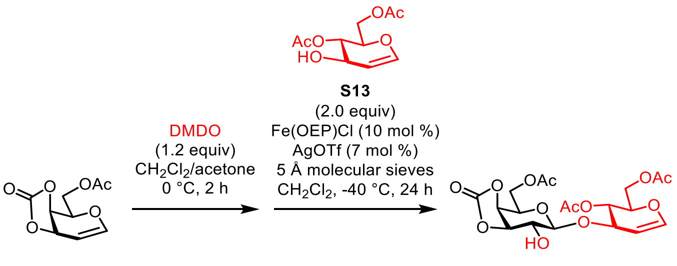

S19

19
77% yield
dr >20:1

The reaction was carried out on a 0.12 mmol scale using the glycal as the limiting reagent by following **Procedure B** for epoxidation and **Procedure C** for the iron-catalyzed stereospecific

glycosylation with the modifications: DMDO (1.2 equiv) was used and glycosylation was carried out at −40 °C for 24 h. Catalyst **1d** (7 mol %) and secondary acceptor **S13** (0.24 mmol, 2 equiv) were used. The *dr* of the corresponding glycal α-epoxide is >20:1. The *dr* of the glycosylation product determined based on crude reaction mixture is >20:1. The desired product **19** was purified through a silica gel flash column (hexanes/EtOAc: from 20:1 to 1:2) as colorless oil (42.7 mg, 77% yield).

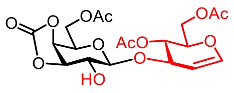

19

4,6-Di-*O*-acetyl-1,5-anhydro-3-*O*-(6-*O*-acetyl-3,4-*O*-carbonyl-β-D-galactopyranosyl)-2-deoxy-D-*arabino*-hex-1-enitol (**19**): $[\alpha]_D^{23}$ +64.1 (acetone, *c* = 1.0); IR ν_{max} (neat)/cm^{-1}: 2922 (w), 1801 (m), 1733 (s), 1648 (m), 1368 (m), 1224 (s), 1165 (m), 1068 (s), 1041 (s), 1026 (s), 979 (s), 899 (m); ^{1}H NMR (400 MHz, $CDCl_3$) δ 6.49 (d, *J* = 6.2 Hz, 1H), 5.21 (t, *J* = 4.2 Hz, 1H), 4.94 (dd, *J* = 6.3, 3.9 Hz, 1H), 4.78 (d, *J* = 6.9 Hz, 1H), 4.75 – 4.69 (m, 1H), 4.64 (d, *J* = 6.2 Hz, 1H), 4.50 – 4.20 (m, 6H), 4.09 (t, *J* = 6.5 Hz, 1H), 3.79 – 3.60 (m, 2H), 2.09 (s, 3H), 2.09 (s, 3H), 2.09 (s, 3H); ^{13}C NMR (100 MHz, $CDCl_3$) δ 171.1, 170.6, 170.1, 153.8, 145.9, 98.6, 97.3, 78.0, 74.2, 73.7, 70.7, 69.9, 69.6, 67.8, 62.4, 61.5, 21.0, 20.9, 20.8; HRMS: m/z (ESI) calcd for $C_{19}H_{25}O_{13}^+$, $[M + H]^+$, 461.1290, found 461.1297. $^1J_{^{13}C1-H1}$ = 161.6 Hz.

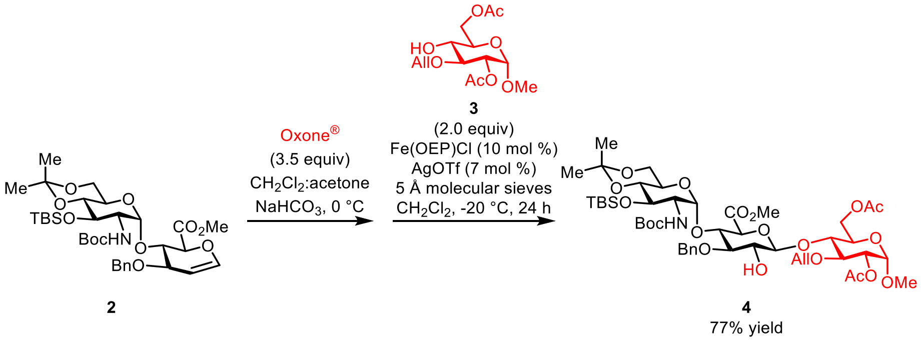

The reaction was carried out on a 0.12 mmol scale using the glycal as the limiting reagent by following **Procedure A** for epoxidation and **Procedure C** for the iron-catalyzed stereospecific glycosylation. Catalyst **1d** (7 mol %) and secondary acceptor **3** (0.24 mmol, 2 equiv) were used. The *dr* of the corresponding glycal α-epoxide is >20:1. The *dr* of the glycosylation product determined based on crude reaction mixture is >20:1. The desired product **4** was purified through a silica gel flash column (hexanes/EtOAc: from 20:1 to 3:2) as white foam (93.7 mg, 77% yield).

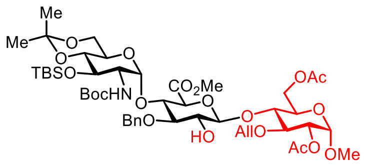

4

Methyl 2-*tert*-butoxycarbonylamino-3-*O*-*tert*-butyldimethylsilyl-4,6-*O*-isopropylidene-2-deoxy-α-D-glucopyranosyl-(1→4)-(methyl 3-*O*-benzyl-β-D-glucopyranosyluronate)-(1→4)-2,6-di-*O*-acetyl-3-*O*-allyl-α-D-glucopyranoside (**4**): $[\alpha]_D^{23}$ +63.3 (acetone, *c* = 1.0); IR ν_{max} (neat)/cm^{-1}: 3454 (w), 2929 (w), 2360 (w), 1741 (s), 1653 (w), 1367 (s), 1232 (s), 1026 (s), 987 (s), 913 (m), 874 (m); ^{1}H NMR (400 MHz, $CDCl_3$) δ 7.33 (d, *J* = 4.4 Hz, 5H), 5.84 (ddt, *J* = 16.2, 10.6, 5.3 Hz, 1H), 5.35 (d, *J* = 3.9 Hz, 1H), 5.22 (dd, *J* = 16.2, 1.9 Hz, 1H), 5.11 (dd, *J* = 10.6, 1.9 Hz, 1H), 4.92 – 4.80 (m, 3H), 4.81 – 4.70 (m, 2H), 4.48 (d, *J* = 7.6 Hz, 1H), 4.45 (d, *J* = 9.9 Hz, 1H), 4.34 (dd, *J* = 12.1, 4.5 Hz, 1H), 4.28 (dd, *J* = 12.9, 5.3 Hz, 1H), 4.10 (dd, *J* = 12.9, 5.3 Hz, 1H), 4.00 (t, *J* = 8.9 Hz, 1H), 3.88 (d, *J* = 9.8 Hz, 1H), 3.85 – 3.73 (m, 4H), 3.78 (s, 3H), 3.71 – 3.59 (m, 2H), 3.61 – 3.54 (m, 2H), 3.54 – 3.43 (m, 2H), 3.35 (s, 3H), 3.21 (td, *J* = 9.9, 5.1 Hz, 1H), 3.01 (brs, 1H), 2.10 (s, 6H), 1.43 (s, 3H), 1.35 (s, 3H), 1.33 (s, 9H), 0.86 (s, 9H), 0.06 (s, 3H), 0.05 (s, 3H); ^{13}C NMR (100 MHz, $CDCl_3$) δ 171.3, 170.3, 168.4, 155.2, 137.8, 135.0, 128.8 (2C), 128.5 (2C), 128.2, 116.4, 103.7, 99.4, 99.3, 97.0, 84.2, 79.8, 78.2, 77.9, 75.2, 74.9, 74.63, 74.59, 74.2, 74.1, 73.3, 71.5, 68.9, 64.4, 62.9, 62.0, 55.4, 55.3, 52.8, 29.2, 28.6 (3C), 25.9 (3C), 21.14, 21.08, 19.0, 18.4, -4.0, -4.9; HRMS: m/z (ESI) calcd for $C_{48}H_{76}NO_{20}Si^+$, [M + H]$^+$, 1014.4724, found 1014.4706. $^{1}J_{^{13}C1-H1}$ = 161.3 Hz, 178.3 Hz, 175.3 Hz.

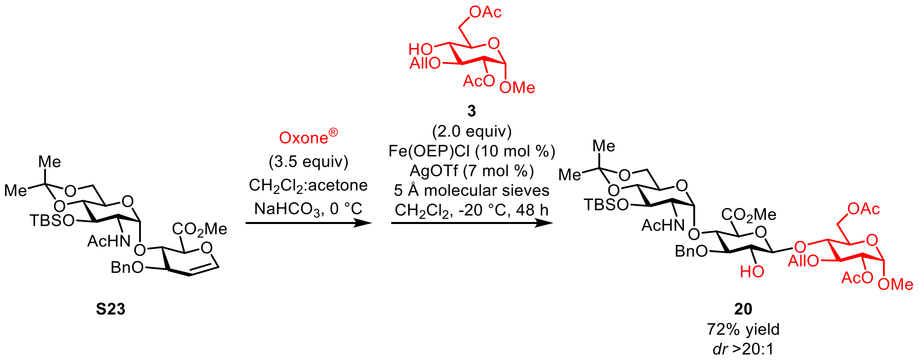

S23 was synthesized according to the following procedure.

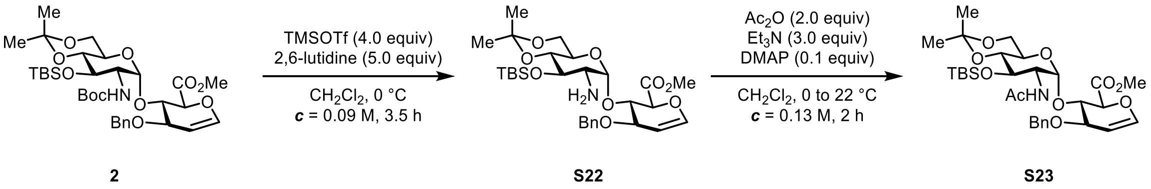

To a 25 mL flame-dried round bottom flask equipped with a stir bar, **2** (0.45 g, 0.66 mmol, 1.0 equiv) and anhydrous CH_2Cl_2 (6 mL) were added. The flask was cooled to 0 °C before 2,6-lutidine (0.39 mL, 3.30 mmol, 5.0 equiv) was then added dropwise followed by the slow addition of TMSOTf (586.1 mg, 2.64 mmol, 4.0 equiv) in CH_2Cl_2 (1.5 mL). The reaction mixture was stirred at 0 °C for 3.5 h, and then quenched with saturated aqueous $NaHCO_3$ solution (5 mL). After the organic phase was separated from the aqueous phase, the aqueous phase was further extracted with CH_2Cl_2 (5 mL 3). The combined organic layer was washed with brine, dried over anhydrous Na_2SO_4, and then concentrated *in vacuo*. The crude product **S22** was dried azeotropically with anhydrous toluene (5 mL × 2) and directly used in the next step without further purification.

To a solution of **S22** (0.66 mmol, 1.0 equiv) and DMAP (5.6 mg, 0.046 mmol, 0.1 equiv) in anhydrous CH_2Cl_2 (5 mL) were added Et_3N (0.19 mL, 1.38 mmol, 3.0 equiv) at 0 °C. After stirring at 0 °C for 10 min, Ac_2O (87 µL, 0.92 mmol, 2.0 equiv) was added dropwise. The reaction mixture was slowly warmed to room temperature and stirred for another 2 h. The reaction mixture was then quenched with saturated aqueous $NaHCO_3$ solution (5 mL) and the

organic phase was separated from the aqueous one. The aqueous phase was further extracted with CH_2Cl_2 (10 mL × 2). The combined organic phase was washed with brine (15 mL) and dried over Na_2SO_4. After concentration *in vacuo*, the residue was purified through column chromatography (hexanes/EtOAc: from 100:1 to 3:1) to afford the desired product **S23** as white foam (269.7 mg, 66% yield over two steps).

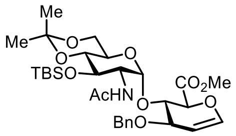

S23

Methyl 3-*O*-benzyl-4-*O*-(2-acetamido-3-*O*-*tert*-butyldimethylsilyl-4,6-*O*-isopropylidene-2-deoxy-α-D-glucopyranosyl)-D-glucuronal (**S23**): $[\alpha]_D^{23}$ +33.7 (acetone, *c* = 1.0); IR ν_{max} (neat)/cm^{-1}: 2992 (w), 2952 (m), 2927 (m), 2856 (m), 2360 (m), 2342 (m), 1763 (m), 1653 (m), 1373 (w), 1247 (m), 1201 (m), 1070 (s), 1037 (s), 866 (m); ^{1}H NMR (400 MHz, $CDCl_3$) δ 7.36 – 7.24 (m, 5H), 6.69 (d, J = 6.3 Hz, 1H), 5.38 (d, J = 9.5 Hz, 1H), 5.03 – 4.95 (m, 2H), 4.79 (dd, J = 2.9, 1.4 Hz, 1H), 4.50 (d, J = 11.3 Hz, 1H), 4.41 (d, J = 11.7 Hz, 1H), 4.39 (s, 1H), 4.19 (td, J = 9.4, 4.0 Hz, 1H), 3.86 (dd, J = 10.3, 4.9 Hz, 1H), 3.79 – 3.67 (m, 2H), 3.68 – 3.59 (m, 2H), 3.58 – 3.53 (m, 1H), 3.55 (s, 3H), 1.90 (s, 3H), 1.47 (s, 3H), 1.39 (s, 3H), 0.84 (s, 9H), 0.04 (s, 3H), 0.04 (s, 3H); ^{13}C NMR (100 MHz, $CDCl_3$) δ 169.7, 168.0, 145.7, 137.6, 128.6 (2C), 128.1 (2C), 128.0, 99.6, 98.0, 97.9, 74.7, 74.2, 71.5, 71.1, 69.7, 66.4, 64.7, 62.3, 54.0, 52.4, 29.2, 25.8 (3C), 23.5, 19.1, 18.3, -3.8, -4.9; HRMS: m/z (ESI) calcd for $C_{31}H_{48}NO_{10}Si^+$, $[M + H]^+$, 622.3042, found 622.3057. $^1J_{^{13}C1–H1}$ = 170.3 Hz.

The reaction was carried out on a 0.12 mmol scale using the glycal as the limiting reagent by following **Procedure A** for epoxidation and **Procedure C** for the iron-catalyzed stereospecific glycosylation. Catalyst **1d** (7 mol %) and secondary acceptor **3** (0.24 mmol, 2 equiv) were used. The *dr* of the corresponding glycal α-epoxide is >20:1. The *dr* of the glycosylation product determined based on crude reaction mixture is >20:1. The desired product **20** was purified through a silica gel flash column (hexanes/EtOAc: from 20:1 to 3:2) as white foam (82.5 mg, 72% yield).

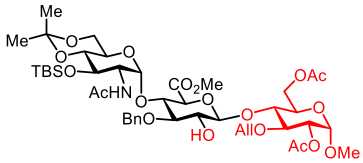

20

Methyl 2-acetamido-3-*O*-*tert*-butyldimethylsilyl-4,6-*O*-isopropylidene-2-deoxy-α-D-glucopyranosyl-(1→4)-(methyl 3-*O*-benzyl-β-D-glucopyranosyluronate)-(1→4)-2,6-di-*O*-acetyl-3-*O*-allyl-α-D-glucopyranoside (**20**): $[\alpha]_D^{23}$ +58.3 (acetone, *c* = 1.0); IR ν_{max} (neat)/cm^{-1}: 3347 (w), 2928 (w), 1747 (w), 1661 (w), 1519 (w), 1242 (m), 1130 (m), 1028 (s), 1043 (s), 864 (s), 837 (s), 778 (m); ^{1}H NMR (400 MHz, $CDCl_3$) δ 7.36 – 7.27 (m, 5H), 5.92 – 5.80 (m, 1H), 5.78 (d, *J* = 9.7 Hz, 1H), 5.23 (d, *J* = 17.7 Hz, 1H), 5.12 (d, *J* = 10.1 Hz, 1H), 5.05 (d, *J* = 10.9 Hz, 1H), 5.00 (d, *J* = 3.7 Hz, 1H), 4.84 (d, *J* = 3.5 Hz, 1H), 4.78 (dd, *J* = 9.8, 3.6 Hz, 1H), 4.66 (d, *J* = 10.9 Hz, 1H), 4.53 (d, *J* = 7.7 Hz, 1H), 4.45 (dd, *J* = 12.2, 2.3 Hz, 1H), 4.36 (dd, *J* = 12.1, 4.6 Hz, 1H), 4.28 (dd, *J* = 12.9, 5.2 Hz, 1H), 4.24 – 4.07 (m, 2H), 3.94 (t, *J* = 8.9 Hz, 1H), 3.87 (d, *J* = 8.3 Hz, 1H), 3.86 – 3.74 (m, 3H), 3.72 (s, 3H), 3.71 – 3.54 (m, 5H), 3.49 (t, *J* = 9.1 Hz, 1H), 3.43 (dd, *J* = 10.0, 4.9 Hz, 1H), 3.40 – 3.33 (m, 1H), 3.36 (s, 3H), 2.11 (s, 3H), 2.10 (s, 3H), 1.56 (s, 3H), 1.43 (s, 3H), 1.37 (s, 3H), 0.83 (s, 9H), 0.044 (s, 3H), 0.036 (s, 3H); ^{13}C NMR (100 MHz, $CDCl_3$) δ 171.4, 170.3, 169.9, 167.9, 137.7, 135.0, 128.8 (2C), 128.3, 128.0 (2C), 116.4, 103.6, 100.0, 99.5, 96.9, 82.7, 77.83, 77.80, 76.4, 75.5, 75.1, 74.63, 74.60, 74.0, 73.3, 71.3, 68.9, 64.8, 63.0, 62.0, 55.3, 54.0, 52.7, 29.2, 25.8 (3C), 23.0, 21.13, 21.08, 19.0, 18.3, -3.9, -4.9; HRMS: m/z (ESI) calcd for $C_{45}H_{70}NO_{19}Si^+$, $[M + H]^+$, 956.4306, found 956.4300. $^1J_{^{13}C1\text{–}H1}$ = 162.4 Hz, 175.2 Hz, 174.4 Hz.

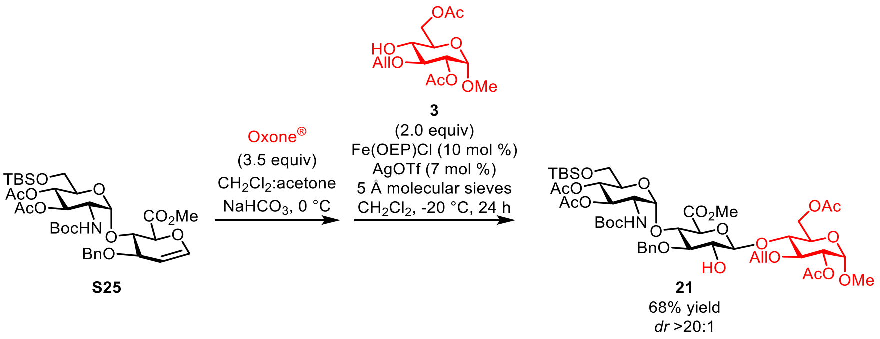

S25 was synthesized according to the following procedure.

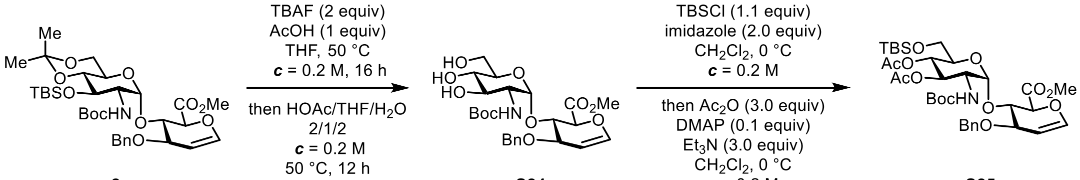

To a 100 mL flame-dried round bottom flask equipped with a stir bar, compound **2** (1.72 g, 2.53 mmol, 1.0 equiv) and anhydrous THF (7.2 mL) were added. The mixture was then added a premixed solution of TBAF (5 mL, 1 M in THF, 2.0 equiv) and AcOH (0.14 mL, 1.0 equiv). The reaction mixture was stirred for 16 h at 50 °C until the starting material **2** was fully consumed (monitored by TLC). The reaction mixture was then cooled down to room temperature and diluted with EtOAc (10 mL) and quenched with saturated aqueous $NaHCO_3$ solution (10 mL). The organic phase was separated from the aqueous one, and the aqueous phase was further extracted with EtOAc (15 mL × 3). The combined organic phase was washed with brine (20 mL) and dried over Na_2SO_4. After concentration *in vacuo*, the desired product was obtained as white foam and directly used in the next step without further purification.

To a 50 mL flame-dried round bottom flask equipped with a stir bar, crude product from the previous step (2.53 mmol, 1.0 equiv) and a mixture of AcOH/THF/H_2O (2:1:2 vol/vol/vol, 12.65 mL) were added. The mixture was stirred at 50 °C for 12 h until the starting material was fully consumed (monitored by TLC). The reaction mixture was concentrated *in vacuo* and the residue was further azeotropically dried with toluene (7 mL× 4) to remove AcOH and H_2O. The desired product **S24** was obtained as white foam and directly used in the next step without further purification.

To a solution of **S24** (2.53 mmol, 1.0 equiv) and TBSCl (419 mg, 2.78 mmol, 1.1 equiv) in anhydrous CH_2Cl_2 (12.7 mL) were added imidazole (345 mg, 5.06 mmol, 2.0 equiv) at 0 °C. After stirring at 0 °C for 3 h, DMAP (31 mg, 0.253 mmol, 0.1 equiv), Et_3N (1.06 mL, 7.59 mmol, 3.0 equiv) at 0 °C. After stirring at 0 °C for 10 min, Ac_2O (0.72 mL, 7.59 mmol, 3.0 equiv) was added dropwise. The reaction mixture stirred at 0 °C for another 2 h, then quenched

with saturated aqueous $NaHCO_3$ solution (10 mL). The organic phase was separated from the aqueous one, and the aqueous phase was further extracted with CH_2Cl_2 (15 mL × 3). The combined organic layer was washed with brine, dried over anhydrous Na_2SO_4, and then concentrated *in vacuo*. The residue was purified through column chromatography (hexanes/EtOAc: from 100:1 to 3:1) to afford the desired product **S25** (1.28 g, 70% yield over four steps) as white foam.

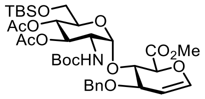

S25

Methyl 3-*O*-benzyl-4-*O*-(3,4-di-*O*-acetyl-6-*O*-*tert*-butyldimethylsilyl-2-*tert*-butoxycarbonylamino-2-deoxy-α-D-glucopyranosyl)-D-glucuronal (**S25**): $[\alpha]_D^{23}$ +47.7 (acetone, *c* = 1.0); IR ν_{max} (neat)/cm^{-1}: 2953 (m), 2929 (m), 2360 (w), 1751 (s), 1500 (m), 1366 (m), 1239 (s), 1037 (s), 1011 (s), 835 (s), 777 (m); ^{1}H NMR (400 MHz, $CDCl_3$) δ 7.46 – 7.12 (m, 5H), 6.65 (d, *J* = 6.3 Hz, 1H), 5.53 – 5.04 (m, 2H), 5.04 – 4.95 (m, 3H), 4.61 (d, *J* = 9.9 Hz, 1H), 4.51 (d, *J* = 11.2 Hz, 1H), 4.41 (d, *J* = 11.3 Hz, 1H), 4.37 (m, 1H), 4.07 – 3.85 (m, 2H), 3.82 – 3.72 (m, 1H), 3.69 – 3.62 (m, 2H), 3.51 (s, 3H), 2.00 (s, 3H), 1.99 (s, 3H), 1.40 (s, 9H), 1.18 – 0.64 (s, 9H), 0.02 (s, 6H); ^{13}C NMR (100 MHz, $CDCl_3$) δ 171.1, 169.4, 168.0, 155.3, 145.8, 137.7, 128.5 (2C), 128.1 (2C), 127.9, 98.8, 98.0, 80.1, 73.7, 73.5, 71.7, 71.2, 69.6, 68.8, 67.0, 62.4, 53.3, 52.2, 28.4 (3C), 25.9 (3C), 20.84, 20.79, 18.4, -5.4, -5.5; HRMS: m/z (ESI) calcd for $C_{35}H_{54}NO_{13}Si^+$, $[M + H]^+$, 724.3359, found 724.3366.

The reaction was carried out on a 0.12 mmol scale using the glycal as the limiting reagent by following **Procedure A** for epoxidation and **Procedure C** for the iron-catalyzed stereospecific glycosylation. Catalyst **1d** (7 mol %) and secondary acceptor **3** (0.24 mmol, 2 equiv) were used. The *dr* of the corresponding glycal α-epoxide is >20:1. The *dr* of the glycosylation product determined based on crude reaction mixture is >20:1. The desired product **21** was purified through a silica gel flash column (hexanes/EtOAc: from 20:1 to 2:1) as white foam (86.3 mg, 68% yield).

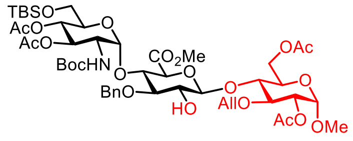

21

Methyl 3,4-di-*O*-acetyl-2-*tert*-butoxycarbonylamino-6-*O*-*tert*-butyldimethylsilyl-2-deoxy-α-D-glucopyranosyl-(1→4)-(methyl 3-*O*-benzyl-β-D-glucopyranosyluronate)-(1→4)-2,6-di-*O*-acetyl-3-*O*-allyl-α-D-glucopyranoside (**21**): $[\alpha]_D^{23}$ +83.7 (acetone, *c* = 1.0); IR ν_{max} (neat)/cm^{-1}: 3463 (w), 2930 (w), 2857 (w), 1741 (m), 1501 (w), 1367 (m), 1234 (s), 1135 (m), 1030 (s), 858 (w), 838 (m), 777 (w); ^{1}H NMR (400 MHz, $CDCl_3$) δ 7.36 – 7.24 (m, 5H), 5.84 (ddt, *J* = 16.2, 10.6, 5.4 Hz, 1H), 5.34 (d, *J* = 3.7 Hz, 1H), 5.22 (d, *J* = 16.2 Hz, 1H), 5.19 – 5.04 (m, 4H), 4.91 (d, *J* = 11.0 Hz, 1H), 4.83 (d, *J* = 3.6 Hz, 1H), 4.81 – 4.72 (m, 2H), 4.50 (d, *J* = 6.7 Hz, 1H), 4.45 (d, *J* = 12.0 Hz, 1H), 4.33 (dd, *J* = 12.1, 5.4 Hz, 1H), 4.27 (dd, *J* = 12.1, 5.4 Hz, 1H), 4.11 (dd, *J* = 12.8, 5.5 Hz, 1H), 4.01 (t, *J* = 8.6 Hz, 1H), 3.93 (td, *J* = 10.1, 3.6 Hz, 1H), 3.90 – 3.75 (m, 3H), 3.75 (s, 3H), 3.69 – 3.63 (d, *J* = 9.7 Hz, 2H), 3.63 – 3.55 (m, 3H), 3.52 (d, *J* = 9.9 Hz, 1H), 3.35 (s, 3H), 3.11 (brs, 1H), 2.10 (s, 3H), 2.09 (s, 3H), 2.00 (s, 3H), 1.97 (s, 3H), 1.32 (s, 9H), 0.86 (s, 9H), -0.00 (s, 6H); ^{13}C NMR (100 MHz, $CDCl_3$) δ 171.3, 171.2, 170.4, 168.9, 168.3, 155.2, 137.8, 135.0, 128.7 (2C), 128.2 (2C), 128.1, 116.4, 103.5, 98.5, 96.9, 83.5, 80.0, 78.0, 77.9, 75.04, 74.95, 74.7, 74.6, 74.1, 73.3, 72.1, 70.8, 68.9, 68.4, 62.9, 61.6, 55.3, 53.2, 52.7, 28.3 (3C), 26.0 (3C), 21.11, 21.05, 20.9, 20.8, 18.4, -5.4, -5.5; HRMS: m/z (ESI) calcd for $C_{49}H_{76}NO_{22}Si^+$, [M + H]$^+$, 1058.4623, found 1058.4599. $^1J_{^{13}C1-H1}$ = 163.2 Hz, 177.7 Hz, 175.7 Hz.

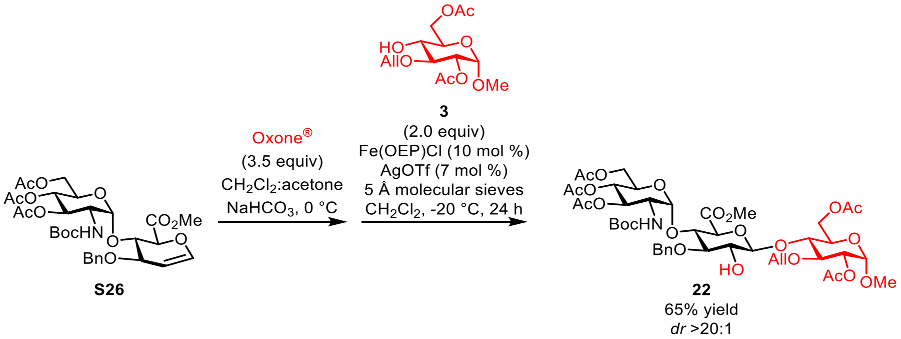

S26 was synthesized according to the following procedure.

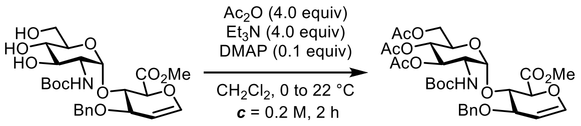

S24 **S26**

96% yield

To a solution of **S24** (210.2 mg, 0.4 mmol, 1.0 equiv) and DMAP (4.9 mg, 0.04 mmol, 0.1 equiv) in anhydrous CH_2Cl_2 (2 mL) was added Et_3N (0.22 mL, 1.6 mmol, 4.0 equiv) at 0 °C. After stirring at 0 °C for 10 min, Ac_2O (0.15 mL, 1.6 mmol, 4.0 equiv) was added dropwise. The reaction mixture was slowly warmed to room temperature and stirred for another 2 h, then quenched with saturated aqueous $NaHCO_3$ solution (2 mL). The organic phase was separated from the aqueous one, and the aqueous phase was further extracted with CH_2Cl_2 (3 mL × 3). The combined organic layer was washed with brine, dried over anhydrous Na_2SO_4, and then concentrated *in vacuo.* The residue was purified through column chromatography (hexanes/EtOAc: from 100:1 to 3:1) to afford the desired product **S26** (250.2 mg, 96% yield) as white foam.

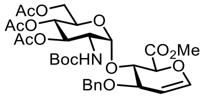

S26

Methyl 3-*O*-benzyl-4-*O*-(3,4,6-tri-*O*-acetyl-2-*tert*-butoxycarbonylamino-2-deoxy-α-D-glucopyranosyl)-D-glucuronal (**S26**): $[\alpha]_D^{23}$ +35.0 (acetone, *c* = 1.0); IR ν_{max} (neat)/cm^{-1}: 3355 (w), 2953 (w), 2922 (w), 2852 (w), 1746 (s), 1716 (s), 1649 (w), 1500 (w), 1366 (w), 1226 (s), 1165 (w), 1107 (w), 1037 (s), 1013 (m); ^{1}H NMR (400 MHz, $CDCl_3$) δ 7.31 (m, 5H), 6.65 (d, *J* = 6.3 Hz, 1H), 5.15 – 5.07 (m, 2H), 5.08 – 4.99 (m, 2H), 4.97 – 4.93 (m, 1H), 4.64 (d, *J* = 10.0 Hz, 1H), 4.53 (d, *J* = 11.2 Hz, 1H), 4.42 (d, *J* = 11.2 Hz, 1H), 4.38 (q, *J* = 2.2 Hz, 1H), 4.22 – 4.11 (m, 2H), 4.09 – 3.93 (m, 2H), 3.82 – 3.76 (m, 1H), 3.53 (s, 3H), 2.07 (s, 3H), 2.03 (s, 3H), 2.01 (s, 3H), 1.41 (s, 9H); ^{13}C NMR (100 MHz, $CDCl_3$) δ 171.0, 170.7, 169.5, 167.9, 155.2, 145.5, 137.6, 128.5 (2C), 128.1 (2C), 128.0, 98.6, 98.2, 80.2, 74.0, 73.4, 71.4, 69.6, 68.7, 68.3,

67.0, 62.2, 53.3, 52.2, 28.3 (3C), 20.79, 20.75, 20.7; HRMS: m/z (ESI) calcd for $C_{31}H_{42}NO_{14}^+$, $[M + H]^+$, 652.2600, found 652.2599.

The reaction was carried out on a 0.12 mmol scale using the glycal as the limiting reagent by following **Procedure A** for epoxidation and **Procedure C** for the iron-catalyzed stereospecific glycosylation. Catalyst **1d** (7 mol %) and secondary acceptor **3** (0.24 mmol, 2 equiv) were used. The *dr* of the corresponding glycal α-epoxide is >20:1. The *dr* of the glycosylation product determined based on crude reaction mixture is >20:1. The desired product **22** was purified through a silica gel flash column (hexanes/EtOAc: from 20:1 to 3:2) as white foam (76.9 mg, 65% yield).

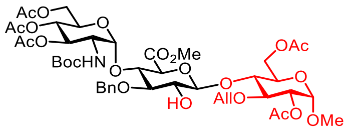

22

Methyl 3,4,6-tri-*O*-acetyl-2-*tert*-butoxycarbonylamino-2-deoxy-α-D-glucopyranosyl-(1→4)-(methyl 3-*O*-benzyl-β-D-glucopyranosyluronate)-(1→4)-2,6-di-*O*-acetyl-3-*O*-allyl-α-D-glucopyranoside (**22**): $[\alpha]_D^{23}$ +59.8 (acetone, *c* = 1.0); IR ν_{max} (neat)/cm^{-1}: 3371 (w), 2921 (w), 1744 (w), 1710 (w), 1506 (w), 1240 (s), 1223 (s), 1132 (m), 1030 (s), 917 (m), 754 (w); ^{1}H NMR (400 MHz, $CDCl_3$) δ 7.37 – 7.27 (m, 5H), 5.84 (ddt, *J* = 17.3, 10.6, 5.3 Hz, 1H), 5.41 (d, *J* = 3.7 Hz, 1H), 5.22 (dd, *J* = 17.3, 1.8 Hz, 1H), 5.16 – 5.02 (m, 4H), 4.95 (d, *J* = 10.8 Hz, 1H), 4.84 (d, *J* = 3.6 Hz, 1H), 4.81 – 4.74 (m, 2H), 4.56 – 4.49 (m, 1H), 4.45 (dd, *J* = 12.1, 2.2 Hz, 1H), 4.34 (dd, *J* = 12.2, 5.3 Hz, 1H), 4.26 (dd, *J* = 12.2, 5.3 Hz, 1H), 4.18 (dd, *J* = 12.5, 3.6 Hz, 1H), 4.12 (dd, *J* = 12.7, 5.5 Hz, 1H), 4.09 – 3.97 (m, 3H), 3.88 (d, *J* = 9.6 Hz, 1H), 3.86 – 3.73 (m, 5H), 3.73 – 3.56 (m, 4H), 3.35 (s, 3H), 3.21 (d, *J* = 2.6 Hz, 1H), 2.11 (s, 3H), 2.10 (s, 3H), 2.08 (s, 3H), 2.01 (s, 3H), 2.00 (s, 3H), 1.32 (s, 9H); ^{13}C NMR (100 MHz, $CDCl_3$) δ 171.4, 171.02, 170.96, 170.3, 169.4, 168.3, 155.1, 137.7, 134.9, 128.7 (2C), 128.3 (2C), 128.1, 116.4, 103.5, 98.7, 96.9, 83.4, 80.1, 77.9 (two peaks overlapped, 2C), 75.6, 74.9, 74.8 (two peaks overlapped, 2C), 74.1, 73.3, 71.7, 68.9, 68.8, 67.9, 63.0, 61.6, 55.4, 53.1, 52.8, 28.3 (3C), 21.2, 21.1, 20.9,

20.8, 20.7; HRMS: m/z (ESI) calcd for $C_{45}H_{64}NO_{23}^+$, $[M + H]^+$, 986.3864, found 986.3837. $^1J_{^{13}C1-H1}$ = 162.5 Hz, 172.3 Hz, 173.4 Hz.

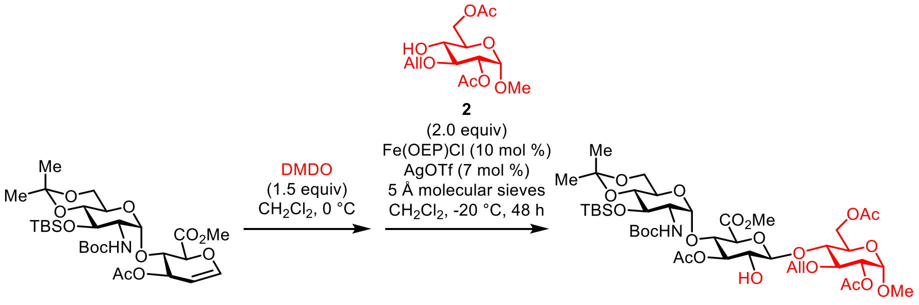

S27 was synthesized according to a literature procedure.[1]

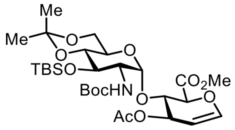

Methyl 3-*O*-acetyl-4-*O*-(2-*tert*-butoxycarbonylamino-3-*O*-*tert*-butyldimethylsilyl-4,6-*O*-isopropylidene-2-deoxy-α-D-glucopyranosyl)-D-glucuronal (S27): $[\alpha]_D^{23}$ +36.9 (acetone, *c* = 1.0); IR ν_{max} (neat)/cm^{-1}: 2954 (w), 2929 (w), 2857 (w), 1722 (s), 1650 (w), 1503 (m), 1368 (s), 1282 (s), 1172 (s), 1076 (s), 1035 (s), 875 (m), 837 (m), 779 (m); ^{1}H NMR (400 MHz, $CDCl_3$) δ 6.72 – 6.65 (d, *J* = 5.5 Hz, 1H), 5.07 (d, *J* = 3.9 Hz, 1H), 5.00 – 4.93 (m, 2H), 4.83 (d, *J* = 3.1 Hz, 1H), 4.51 (d, *J* = 9.9 Hz, 1H), 4.32 (d, *J* = 2.4 Hz, 1H), 3.87 – 3.80 (m, 2H), 3.77 (s, 3H), 3.70 (t, *J* = 10.3 Hz, 1H), 3.64 – 3.46 (m, 3H), 1.97 (s, 3H), 1.46 (s, 3H), 1.43 (s, 9H), 1.38 (s, 3H), 0.85 (s, 9H), 0.05 (s, 3H), 0.04 (s, 3H); ^{13}C NMR (100 MHz, $CDCl_3$) δ 169.7, 167.9, 155.3, 146.7, 99.6, 99.2, 97.1, 79.9, 74.8, 74.1, 72.0, 71.2, 64.8, 63.3, 62.4, 55.5, 52.5, 29.2, 28.5 (3C), 25.9 (3C), 21.1, 19.1, 18.4, -4.0, -4.9; HRMS: m/z (ESI) calcd for $C_{29}H_{50}NO_{12}Si^+$, $[M + H]^+$, 632.3097, found 632.3089. $^1J_{^{13}C1-H1}$ = 172.0 Hz.

The reaction was carried out on a 0.12 mmol scale using the glycal as the limiting reagent by following **Procedure B** for epoxidation and **Procedure C** for the iron-catalyzed stereospecific glycosylation. Catalyst **1d** (7 mol %) and secondary acceptor **2** (0.24 mmol, 2 equiv) were used. The *dr* of the corresponding glycal α-epoxide is >20:1. The *dr* of the glycosylation product determined based on crude reaction mixture is >20:1. The desired product **23** was purified through a silica gel flash column (hexanes/EtOAc: from 20:1 to 2:1) as white foam (78.7 mg, 68% yield).

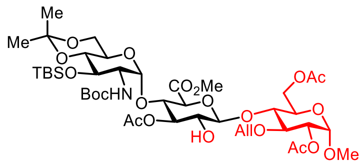

23

Methyl 2-*tert*-butoxycarbonylamino-3-*O*-*tert*-butyldimethylsilyl-4,6-*O*-isopropylidene-2-deoxy-α-D-glucopyranosyl-(1→4)-(methyl 3-*O*-acetyl-β-D-glucopyranosyluronate)-(1→4)-2,6-di-*O*-acetyl-3-*O*-allyl-α-D-glucopyranoside (**23**): $[\alpha]_D^{23}$ +85.3 (acetone, *c* = 1.0); IR ν_{max} (neat)/cm^{-1}: 3447 (w), 2929 (w), 1745 (s), 1504 (m), 1368 (s), 1228 (s), 1170 (s), 1130 (s), 1032 (s), 874 (s), 837 (s), 778 (s); ^{1}H NMR (400 MHz, $CDCl_3$) δ 5.81 (ddt, *J* = 16.3, 10.6, 5.4 Hz, 1H), 5.20 (d, *J* = 16.3 Hz, 1H), 5.16 – 5.06 (m, 2H), 4.93 (d, *J* = 3.3 Hz, 1H), 4.82 (d, *J* = 3.7 Hz, 1H), 4.79 – 4.72 (m, 1H), 4.56 (d, *J* = 7.8 Hz, 1H), 4.50 – 4.41 (m, 2H), 4.34 (dd, *J* = 12.3, 4.5 Hz, 1H), 4.26 (dd, *J* = 12.6, 5.4 Hz, 1H), 4.14 – 4.01 (m, 2H), 3.91 (d, *J* = 9.5 Hz, 1H), 3.86 – 3.71 (m, 4H), 3.7 (s, 3H), 3.67 – 3.57 (m, 2H), 3.53 – 3.67 (m, 4H), 3.34 (s, 3H), 3.31 – 3.20 (m, 2H), 2.11 (s, 3H), 2.093 (s, 3H), 2.089 (s, 3H), 1.425 (s, 9H), 1.417 (s, 3H), 1.34 (s, 3H), 0.84 (s, 9H), 0.03 (s, 3H), 0.02 (s, 3H); ^{13}C NMR (100 MHz, $CDCl_3$) δ 171.4, 170.9, 170.3, 167.9, 155.3, 135.0, 116.2, 103.9, 99.8, 99.4, 96.9, 79.9, 78.6, 77.9, 76.2, 74.9, 74.6, 74.5, 74.1, 73.3, 72.6, 70.9, 68.8, 64.7, 62.9, 61.8, 55.5, 55.3, 52.8, 29.1, 28.5 (3C), 25.9 (3C), 21.11 (two peaks overlapped, 2C), 21.05, 19.0, 18.3, -4.1, -5.0; HRMS: m/z (ESI) calcd for $C_{43}H_{72}NO_{21}Si^+$, [M + H]$^+$, 966.4361, found 966.4349. $^{1}J_{^{13}C1-H1}$ = 162.8 Hz, 175.5 Hz, 174.4 Hz.

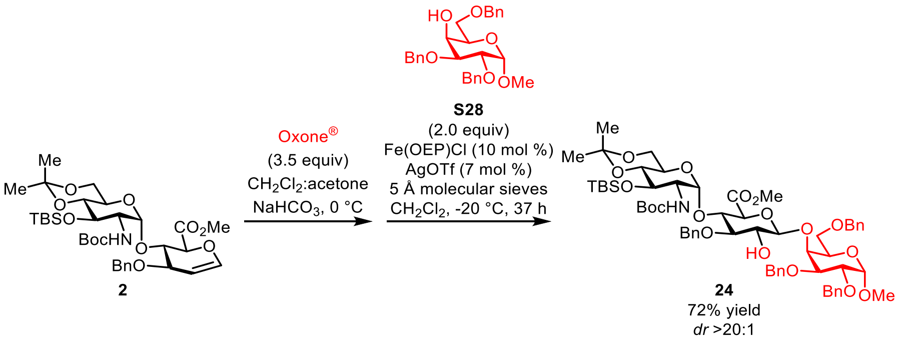

S28 was synthesized according to a literature procedure.[15]

The reaction was carried out on a 0.12 mmol scale using the glycal as the limiting reagent by following **Procedure A** for epoxidation and **Procedure C** for the iron-catalyzed stereospecific glycosylation. Catalyst **1d** (7 mol %) and secondary acceptor **S28** (0.24 mmol, 2 equiv) were used. The *dr* of the corresponding glycal α-epoxide is >20:1. The *dr* of the glycosylation product determined based on crude reaction mixture is >20:1. The desired product **24** was purified through a silica gel flash column (hexanes/EtOAc: from 20:1 to 6:1) as white foam (99.7 mg, 72% yield).

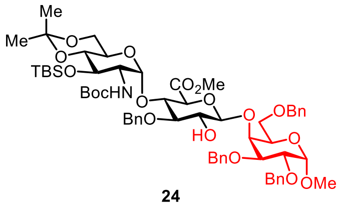

24

Methyl 2-*tert*-butoxycarbonylamino-3-*O*-*tert*-butyldimethylsilyl-4,6-*O*-isopropylidene-2-deoxy-α-D-glucopyranosyl-(1→4)-(methyl 3-*O*-benzyl-β-D-glucopyranosyluronate)-(1→4)-2,3,6-tri-*O*-benzyl-α-D-galactopyranoside (**24**): $[\alpha]_D^{23}$ +36.6 (acetone, *c* = 1.6); IR ν_{max} (neat)/cm^{-1}: 3437 (w), 2927 (w), 1719 (m), 1498 (w), 1129 (s), 1077 (s), 1040 (s), 1027(s), 836 (m), 734 (m), 696 (s); ^{1}H NMR (400 MHz, $CDCl_3$) δ 7.45 – 7.27 (m, 20H), 5.31 (d, *J* = 3.9 Hz, 1H), 5.03 (d, *J* = 10.1 Hz, 1H), 4.95 (d, *J* = 7.4 Hz, 1H), 4.92 (d, *J* = 6.2 Hz, 1H), 4.80 (d, *J* = 11.9 Hz, 1H), 4.74 (d, *J* = 11.6 Hz, 1H), 4.70 – 4.61 (m, 3H), 4.49 (d, *J* = 11.6 Hz, 1H), 4.43 – 4.36 (m, 2H), 4.07 (d, *J* = 2.6 Hz, 1H), 4.01 – 3.89 (m, 4H), 3.89 – 3.81 (m, 2H), 3.80 – 3.70 (m,

3H), 3.69 – 3.57 (m, 5H), 3.57 – 3.41 (m, 4H), 3.35 (s, 3H), 3.23 (td, J = 9.8, 5.1 Hz, 1H), 1.44 (s, 3H), 1.39 (s, 9H), 1.35 (s, 3H), 0.89 (s, 9H), 0.09 (s, 3H), 0.06 (s, 3H); ^{13}C NMR (100 MHz, $CDCl_3$) δ 168.3, 155.2, 138.3, 138.1, 137.9, 137.6, 128.9 (2C), 128.7 (2C), 128.6 (2C), 128.5 (three peaks overlapped, 6C), 128.29 (2C), 128.25, 128.1, 127.9, 127.8, 127.6 (2C), 106.0, 99.6, 99.3, 98.5, 83.3, 79.6, 79.5, 77.6, 76.7, 75.7, 75.3, 74.6 (two peaks overlapped, 2C), 74.5, 74.3, 73.6, 73.2, 71.6, 68.13, 68.06, 64.3, 62.0, 55.6, 55.5, 52.6, 29.2, 28.6 (3C), 26.0 (3C), 19.0, 18.4, -4.0, -4.9; HRMS: m/z (ESI) calcd for $C_{62}H_{86}NO_{18}Si^+$, $[M + H]^+$, 1160.5609, found 1160.5587. $^1J_{^{13}C1\text{–}H1}$ = 159.8 Hz, 177.7 Hz, 171.2 Hz.

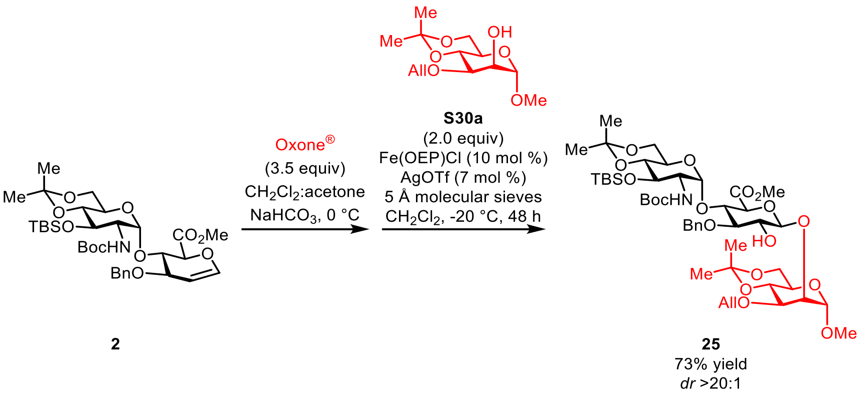

S30 was synthesized according to the following procedure.

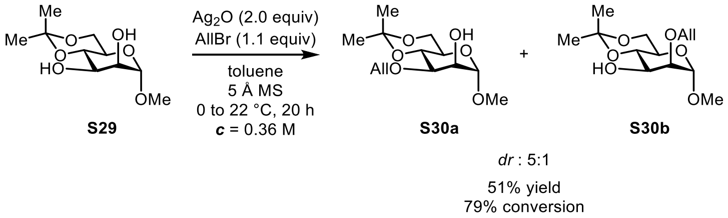

To a 100 mL flame-dried round bottom flask equipped with a stir bar, compound **S29**[16] (1.60 g, 7.2 mmol, 1.0 equiv), Ag_2O (3.38 g, 14.4 mmol, 2.0 equiv), freshly activated 5 Å molecular sieves (powder, *ca.* 1.0 g), and anhydrous toluene (20 mL) were added. The mixture was cooled to 0 °C before allyl bromide (0.69 mL, 7.92 mmol, 1.1 equiv) was added dropwise. The reaction mixture was then stirred at room temperature for 20 h. The reaction mixture was then filtered through a short pad of Celite® (rinsed with acetone) and concentrated *in vacuo*, the residue was

purified through column chromatography (hexanes/diethyl ether: from 100:1 to 3:2) to afford the desired product **S30a** (830 mg, 43% yield) as white foam.

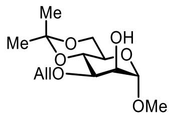

S30a

Methyl 3-*O*-allyl-4,6-*O*-isopropylidene-α-D-mannopyranoside (**S30a**): $[\alpha]_D^{23}$ +74.3 (acetone, *c* = 1.0); IR ν_{max} (neat)/cm^{-1}: 3468 (w), 2992 (w), 2916 (w), 2360 (w), 1371 (w), 1367 (s), 1266 (w), 1114 (m), 1084 (s), 1072 (s), 1048 (s), 1034 (s), 941 (m), 916 (m), 882 (m), 853 (s); ^{1}H NMR (400 MHz, $CDCl_3$) δ 6.11 – 5.73 (m, 1H), 5.30 (m, 1H), 5.18 (dt, *J* = 10.4, 1.5 Hz, 1H), 4.72 (d, *J* = 1.6 Hz, 1H), 4.25 (dd, *J* = 13.1, 5.5, 1H), 4.14 (dd, *J* = 13.1, 5.8, 1H), 4.07 – 3.97 (m, 2H), 3.90 – 3.77 (m, 2H), 3.70 – 3.56 (m, 2H), 3.35 (s, 3H), 2.62 (brs, 1H), 1.51 (s, 3H), 1.41 (s, 3H); ^{13}C NMR (100 MHz, $CDCl_3$) δ 134.7, 117.1, 101.0, 99.7, 75.6, 71.5, 71.2, 69.8, 64.0, 62.3, 54.9, 29.3, 19.2; HRMS: m/z (ESI) calcd for $C_{13}H_{23}O_6^+$, $[M + H]^+$, 275.1489, found 275.1495.

The reaction was carried out on a 0.12 mmol scale using the glycal as the limiting reagent by following **Procedure A** for epoxidation and **Procedure C** for the iron-catalyzed stereospecific glycosylation. Catalyst **1d** (7 mol %) and secondary acceptor **S30a** (0.24 mmol, 2 equiv) were used. The *dr* of the corresponding glycal α-epoxide is >20:1. The *dr* of the glycosylation product determined based on crude reaction mixture is >20:1. The desired product **25** was purified through a silica gel flash column (hexanes/EtOAc: from 20:1 to 4:1) as white foam (84.6 mg, 73% yield).

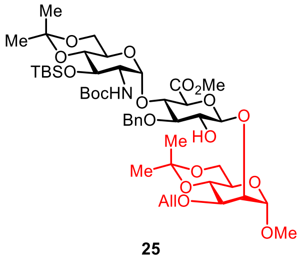

25

Methyl 2-*tert*-butoxycarbonylamino-3-*O*-*tert*-butyldimethylsilyl-4,6-*O*-isopropylidene-2-deoxy-α-D-glucopyranosyl-(1→4)-(methyl 3-*O*-benzyl-β-D-glucopyranosyluronate)-(1→2)-3-*O*-allyl-4,6-*O*-isopropylidene-α-D-mannopyranoside (**25**): $[\alpha]_D^{23}$ +12.2 (acetone, *c* = 1.0); IR ν_{max} (neat)/cm^{-1}: 2928 (w), 2360 (w), 1723 (w), 1501 (w), 1368 (w), 1171 (m), 1128 (s), 1073 (s), 1027 (s), 867 (s), 778 (m); ^{1}H NMR (400 MHz, $CDCl_3$) δ 7.37 – 7.22 (m, 5H), 5.88 (ddt, *J* = 16.7, 11.4, 6.0 Hz, 1H), 5.36 (d, *J* = 3.9 Hz, 1H), 5.28 (dd, *J* = 16.7, 1.7 Hz, 1H), 5.19 (d, *J* = 11.4, 1.7 Hz, 1H), 4.97 (d, *J* = 10.2 Hz, 1H), 4.92 (d, *J* = 10.2 Hz, 1H), 4.69 (d, *J* = 10.3 Hz, 1H), 4.61 (s, 1H), 4.58 (d, *J* = 8.5 Hz, 1H), 4.34 (s, 1H), 4.32 (dd, *J* = 14.9, 6.0 Hz, 1H), 4.22 – 4.03 (m, 3H), 3.98 (t, *J* = 9.0 Hz, 1H), 3.92 (d, *J* = 9.7 Hz, 1H), 3.88 – 3.68 (m, 9H), 3.67 – 3.54 (m, 3H), 3.53 – 3.42 (m, 2H), 3.32 (s, 3H), 3.21 (td, *J* = 9.8, 5.2 Hz, 1H), 1.51 (s, 3H), 1.43 (s, 3H), 1.40 (s, 3H), 1.35 (s, 12H), 0.87 (s, 9H), 0.07 (s, 3H), 0.05 (s, 3H); ^{13}C NMR (100 MHz, $CDCl_3$) δ 168.7, 155.2, 138.0, 134.1, 128.7 (2C), 128.5 (2C), 128.0, 118.6, 101.6 (two peaks overlapped, 2C), 99.8, 99.5, 99.4, 83.1, 79.7, 75.5, 75.0, 74.8, 74.6, 74.5, 73.6, 72.2, 71.7, 71.6, 70.9, 64.6, 64.4, 62.5, 62.0, 55.4, 55.1, 52.8, 29.5, 29.2, 28.6 (3C), 25.9 (3C), 19.5, 19.0, 18.4, -4.0, -4.9; HRMS: m/z (ESI) calcd for $C_{47}H_{76}NO_{18}Si^+$, $[M + H]^+$, 970.4826, found 970.4831. $^{1}J^{13}_{C1-H1}$ = 175.3 Hz, 158.1 Hz, 177.2 Hz.

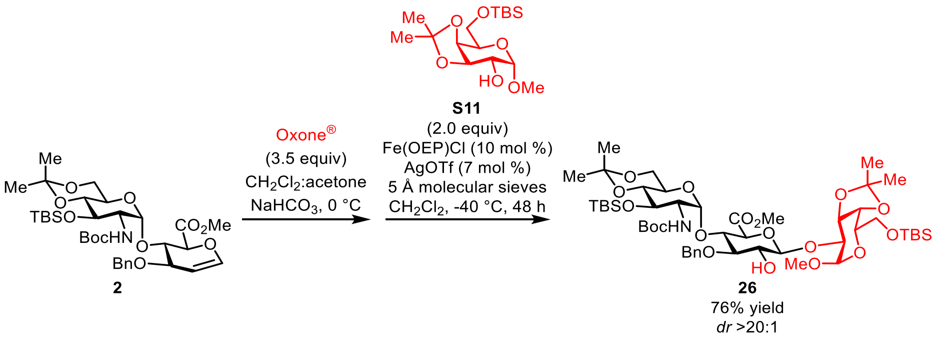

The reaction was carried out on a 0.12 mmol scale using the glycal as the limiting reagent by following **Procedure A** for epoxidation and **Procedure C** for the iron-catalyzed stereospecific glycosylation with the modifications: glycosylation was carried out at −40 °C. Catalyst **1d** (7 mol %) and secondary acceptor **S11** (0.24 mmol, 2 equiv) were used. The *dr* of the corresponding glycal α-epoxide is >20:1. The *dr* of the glycosylation product determined based

on crude reaction mixture is >20:1. The desired product **26** was purified through a silica gel flash column (hexanes/EtOAc: from 20:1 to 6:1) as white foam (95.4 mg, 76% yield).

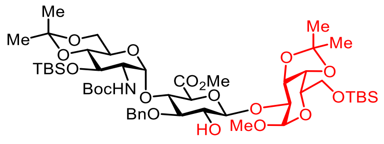

26

Methyl 2-*tert*-butoxycarbonylamino-3-*O*-*tert*-butyldimethylsilyl-4,6-*O*-isopropylidene-2-deoxy-α-D-glucopyranosyl-(1→4)-(methyl 3-*O*-benzyl-β-D-glucopyranosyluronate)-(1→2)-6-*O*-*tert*-butyldimethylsilyl-3,4-*O*-isopropylidene-α-D-galactopyranoside (**26**): $[\alpha]_D^{23}$ +36.1 (acetone, *c* = 1.0); IR ν_{max} (neat)/cm^{-1}: 3455 (w), 2929 (w), 1725 (m), 1505 (m), 1366 (s), 1217 (s), 1130 (s), 1070 (s), 1024 (s), 836 (s), 777 (s); ^{1}H NMR (400 MHz, $CDCl_3$) δ 7.37 – 7.29 (m, 4H), 7.28 – 7.23 (m, 1H), 5.33 (d, *J* = 3.8 Hz, 1H), 4.94 (d, *J* = 10.4 Hz, 1H), 4.91 (d, *J* = 9.8 Hz, 1H), 4.74 (d, *J* = 3.6 Hz, 1H), 4.71 (d, *J* = 10.8 Hz, 1H), 4.68 (d, *J* = 7.2 Hz, 1H), 4.32 (dd, *J* = 8.2, 5.3 Hz, 1H), 4.21 (dd, *J* = 5.3, 2.4 Hz, 1H), 4.08 – 3.93 (m, 2H), 3.90 – 3.83 (m, 4H), 3.83 – 3.73 (m, 6H), 3.72 – 3.57 (m, 3H), 3.54 – 3.41 (m, 2H), 3.35 (s, 3H), 3.23 (td, *J* = 9.9, 5.2 Hz, 1H), 1.51 (s, 3H), 1.43 (s, 3H), 1.35 (s, 3H), 1.33 (s, 12H), 0.89 (s, 9H), 0.87 (s, 9H), 0.08 (s, 6H), 0.06 (s, 3H), 0.05 (s, 3H); ^{13}C NMR (100 MHz, $CDCl_3$) δ 168.6, 155.2, 138.2, 128.6 (2C), 128.4 (2C), 127.9, 109.4, 103.1, 99.5, 99.4, 99.0, 83.8, 79.7, 76.8, 75.6, 74.9, 74.8, 74.7, 74.6, 74.5, 73.6, 71.6, 68.0, 64.4, 62.3, 62.0, 55.5, 55.4, 52.8, 29.2, 28.6 (3C), 28.4, 26.6, 25.94 (3C), 25.93 (3C), 19.0, 18.41, 18.38, -4.0, -4.9, -5.2, -5.4; HRMS: m/z (ESI) calcd for $C_{50}H_{86}NO_{18}Si_2^+$, [M + H]$^+$, 1044.5378, found 1044.5391. $^1J_{^{13}C1-H1}$ = 163.9 Hz, 176.5 Hz, 172.6 Hz.

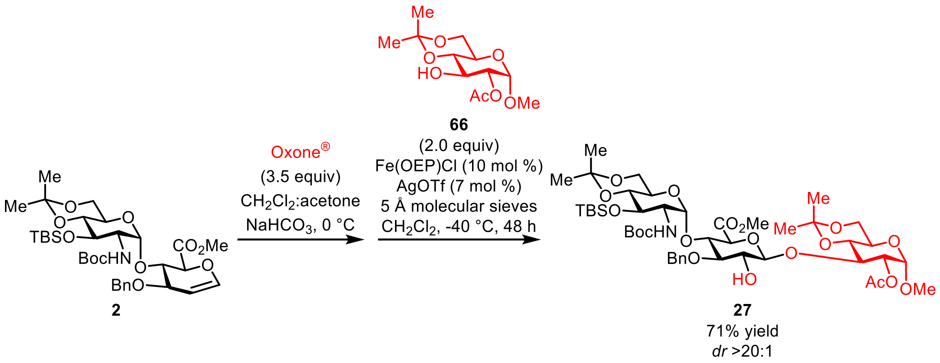

The reaction was carried out on a 0.12 mmol scale using the glycal as the limiting reagent by following the **Procedure A** for epoxidation and **Procedure C** for the iron-catalyzed stereospecific glycosylation with the modifications: glycosylation was carried out at −40 °C. Catalyst **1d** (7 mol %) and secondary acceptor **66** (0.24 mmol, 2 equiv) were used. The *dr* of the corresponding glycal α-epoxide is >20:1. The *dr* of the glycosylation product determined based on crude reaction mixture is >20:1. The desired product **27** was purified through a silica gel flash column (hexanes/EtOAc: from 20:1 to 2:1) as white foam (82.7 mg, 71% yield).

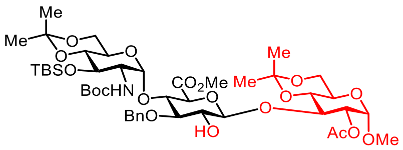

27

Methyl 2-*tert*-butoxycarbonylamino-3-*O*-*tert*-butyldimethylsilyl-4,6-*O*-isopropylidene-2-deoxy-α-D-glucopyranosyl-(1→4)-(methyl 3-*O*-benzyl-β-D-glucopyranosyluronate)-(1→3)-2-*O*-acetyl-4,6-*O*-isopropylidene-α-D-glucopyranoside (**27**): $[\alpha]_D^{23}$ +36.9 (acetone, *c* = 1.3); IR ν_{max} (neat)/cm^{-1}: 3461 (w), 2928 (w), 1723 (s), 1505 (m), 1368 (s), 1237 (s), 1170 (s), 1130 (s), 1047 (s), 987 (s), 875 (s), 778 (m); ^{1}H NMR (400 MHz, $CDCl_3$) δ 7.41 – 7.19 (m, 5H), 5.31 (d, *J* = 3.8 Hz, 1H), 4.94 (d, *J* = 10.0 Hz, 1H), 4.91 (d, *J* = 10.5 Hz, 1H), 4.85 (dd, *J* = 9.4, 3.8 Hz, 1H), 4.81 (d, *J* = 3.4 Hz, 1H), 4.71 (d, *J* = 10.4 Hz, 1H), 4.41 (d, *J* = 7.6 Hz, 1H), 4.09 (t, *J* = 9.1 Hz, 1H), 4.00 (t, *J* = 9.0 Hz, 1H), 3.90 – 3.75 (m, 5H), 3.76 (s, 3H), 3.70 – 3.57 (m, 5H), 3.51 – 3.41 (m, 2H), 3.35 (s, 3H), 3.30 – 3.15 (m, 2H), 2.03 (s, 3H), 1.47 (s, 3H), 1.42 (s, 3H), 1.40 (s, 3H), 1.34 (s, 3H), 1.33 (s, 9H), 0.86 (s, 9H), 0.06 (s, 3H), 0.04 (s, 3H); ^{13}C NMR (100 MHz, $CDCl_3$) δ 170.6, 168.5, 155.2, 137.9, 128.53 (2C), 128.52 (2C), 128.0, 102.1, 100.2, 99.6, 99.3, 97.9, 83.4, 79.6, 75.2, 74.7, 74.62, 74.57, 74.5, 73.2, 73.0, 71.7, 71.6, 64.4, 63.1, 62.4, 61.9, 55.5, 55.3, 52.6, 29.2, 28.9, 28.6 (3C), 25.9 (3C), 20.9, 19.04, 19.00, 18.4, -4.0, -5.0; HRMS: m/z (ESI) calcd for $C_{46}H_{74}NO_{19}Si^+$, [M + H]$^+$, 972.4619, found 972.4601. $^1J_{^{13}C1\text{–}H1}$ = 175.3 Hz, 175.7 Hz, 160.2 Hz.

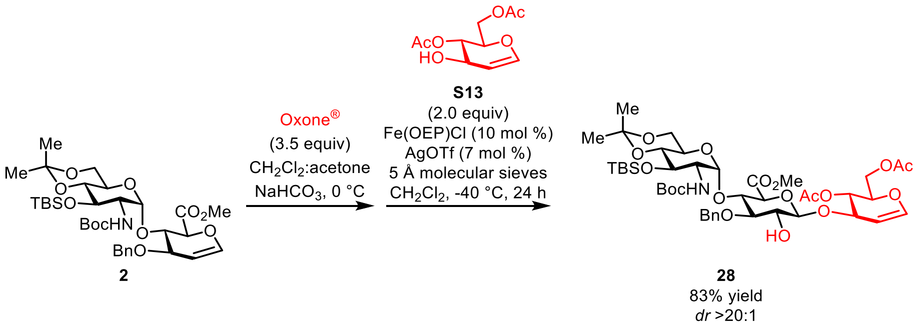

The reaction was carried out on a 0.12 mmol scale using the glycal as the limiting reagent by following the **Procedure A** for epoxidation and **Procedure C** for the iron-catalyzed stereospecific glycosylation with the modifications: glycosylation was carried out at −40 °C. Catalyst **1d** (7 mol %) and secondary acceptor **S13** (0.24 mmol, 2 equiv) were used. The *dr* of the corresponding glycal α-epoxide is >20:1. The *dr* of the glycosylation product determined based on crude reaction mixture is >20:1. The desired product **28** was purified through a silica gel flash column (hexanes/EtOAc: from 20:1 to 3:1) as white foam (92.1 mg, 83% yield).

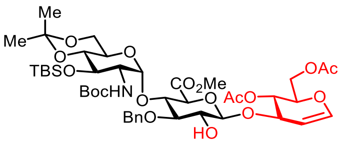

28

4,6-Di-*O*-acetyl-1,5-anhydro-3-*O*-[(2-*tert*-butoxycarbonylamino-3-*O*-*tert*-butyldimethylsilyl-4,6-*O*-isopropylidene-2-deoxy-α-D-glucopyranosyl)-(1→4)-(methyl 3-*O*-benzyl-β-D-glucopyranosyluronate)]-2-deoxy-D-*arabino*-hex-1-enitol (**28**): $[\alpha]_D^{23}$ −3.7 (acetone, *c* = 1.4); IR ν_{max} (neat)/cm^{-1}: 3371 (w), 2928 (w), 1745 (s), 1504 (w), 1367 (m), 1229 (s), 1130 (s), 1024 (s), 874 (s), 836 (m), 778 (m); ^{1}H NMR (400 MHz, $CDCl_3$) δ 7.44 – 7.17 (m, 5H), 6.47 (d, *J* = 6.2 Hz, 1H), 5.32 (d, *J* = 3.8 Hz, 1H), 5.20 (t, *J* = 5.1 Hz, 1H), 4.98 – 4.88 (m, 3H), 4.72 (d, *J* = 10.4 Hz, 1H), 4.46 (d, *J* = 7.5 Hz, 1H), 4.33 – 4.24 (m, 4H), 3.99 (t, *J* = 9.0 Hz, 1H), 3.89 (d, *J* = 9.6 Hz, 1H), 3.86 – 3.72 (m, 5H), 3.67 – 3.57 (m, 2H), 3.58 – 3.50 (m, 1H), 3.52 – 3.40 (m, 2H), 3.23 (td,*J* = 10.0, 5.2 Hz, 1H), 2.99 (brs, 1H), 2.09 (s, 3H), 2.07 (s, 3H), 1.44 (s, 3H), 1.36 (s, 3H), 1.35 (s, 9H), 0.87 (s, 9H), 0.07 (s, 3H), 0.05 (s, 3H); ^{13}C NMR (100 MHz, $CDCl_3$) δ 171.1, 170.1, 168.5, 155.2, 145.8, 137.8, 128.6 (2C), 128.5 (2C), 128.0, 100.4, 99.40, 99.35, 98.0, 83.6,

79.7, 74.9, 74.53, 74.49, 74.3, 73.7, 71.5, 71.1, 67.8 (two peaks overlapped, 2C), 64.4, 62.0, 61.5, 55.4, 52.8, 29.2, 28.5 (3C), 25.9 (3C), 21.0, 20.9, 19.0, 18.4, -4.0, -5.0; HRMS: m/z (ESI) calcd for $C_{44}H_{68}NO_{18}Si^+$, $[M + H]^+$, 926.4200, found 926.4187. $^1J_{^{13}C1\text{–}H1}$ = 176.4 Hz, 160.4 Hz.

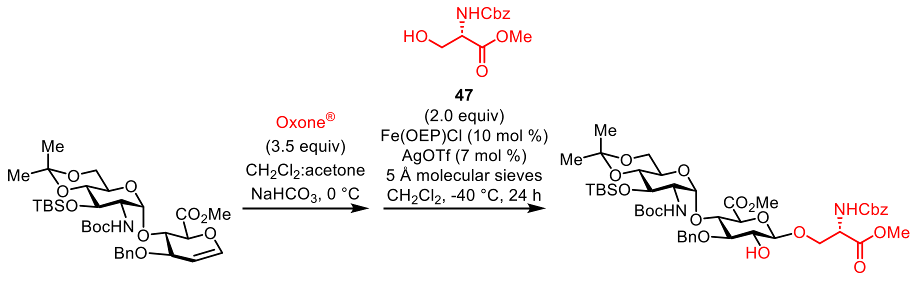

2 **29** 80% yield *dr* >20:1

47 was synthesized according to a literature procedure.[17]

The reaction was carried out on a 0.12 mmol scale using the glycal as the limiting reagent by following the **Procedure A** for epoxidation and **Procedure C** for the iron-catalyzed stereospecific glycosylation with the modifications: glycosylation was carried out at −40 °C. Catalyst **1d** (7 mol %) and primary acceptor **47** (0.24 mmol, 2 equiv) were used. The *dr* of the corresponding glycal α-epoxide is >20:1. The *dr* of the glycosylation product determined based on crude reaction mixture is >20:1. The desired product **29** was purified through a silica gel flash column (hexanes/EtOAc: from 20:1 to 2:1) as white foam (91.0 mg, 80% yield).

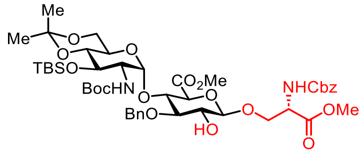

29

***N*-Benzyloxycarbonyl-*O*-[2-*tert*-butoxycarbonylamino-3-*O*-*tert*-butyldimethylsilyl-4,6-*O*-isopropylidene-2-deoxy-α-D-glucopyranosyl-(1→4)-(methyl 3-*O*-benzyl-β-D-glucopyranosyluronate)]-L-serine methyl ester** (**29**): $[\alpha]_D^{23}$ +9.6 (acetone, *c* = 1.5); IR ν_{max} (neat)/cm^{-1}: 2925 (w), 2360 (w), 1751 (w), 1721 (m), 1501 (s), 1212 (m), 1170 (s), 1073 (s),

1024 (s), 874 (m), 777 (m); ^{1}H NMR (400 MHz, acetone-d$_6$) δ 7.43 – 7.19 (m, 10H), 6.76 (d, J = 9.3 Hz, 1H), 5.90 (d, J = 10.0 Hz, 1H), 5.47 (d, J = 3.8 Hz, 1H), 5.11 (s, 2H), 5.08 (brs, 1H), 5.04 (d, J = 10.1 Hz, 1H), 4.70 (d, J = 10.1 Hz, 1H), 4.52 (tt, J = 6.5, 3.3 Hz, 1H), 4.46 (d, J = 7.8 Hz, 1H), 4.31 (dd, J = 10.0, 3.8 Hz, 1H), 4.07 (d, J = 9.4 Hz, 1H), 3.90 (t, J = 9.0 Hz, 1H), 3.88 – 3.77 (m, 6H), 3.75 – 3.62 (m, 6H), 3.55 – 3.40 (m, 2H), 3.25 (td, J = 9.7, 6.1 Hz, 1H), 1.49 (s, 3H), 1.35 (s, 9H), 1.34 (s, 3H), 0.89 (s, 9H), 0.10 (s, 3H), 0.09 (s, 3H); ^{13}C NMR (100 MHz, acetone-d$_6$) δ 171.1, 169.9, 157.1, 156.2, 139.4, 138.0, 129.4 (2C), 129.2 (2C), 129.0 (2C), 128.8 (2C), 128.7, 128.4, 104.5, 99.9, 99.3, 84.9, 79.2, 75.5, 75.3, 75.0, 74.4, 72.2, 70.9, 67.0, 64.9, 62.4, 56.4, 55.2, 55.1, 52.9, 52.6, 29.6, 28.8 (3C), 26.3 (3C), 19.3, 18.9, -3.8, -4.8; HRMS: m/z (ESI) calcd for $C_{46}H_{69}N_2O_{17}Si^+$, $[M + H]^+$, 949.4360, found 949.4337. $^{1}J_{^{13}C1\text{–}H1}$ = 161.8 Hz, 171.1 Hz.

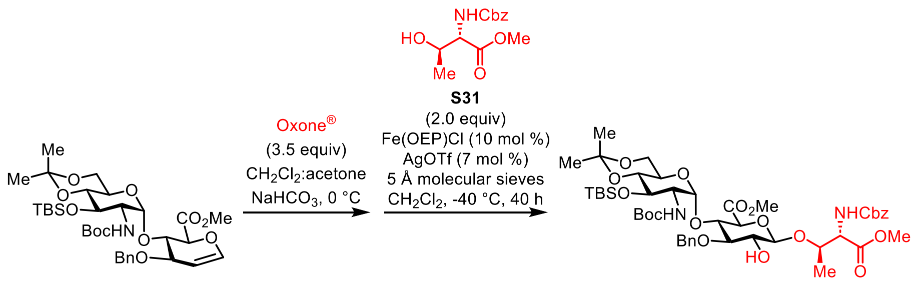

2

30
75% yield
dr >20:1

S31 was synthesized according to a literature procedure.[17]

The reaction was carried out on a 0.12 mmol scale using the glycal as the limiting reagent by following the **Procedure A** for epoxidation and **Procedure C** for the iron-catalyzed stereospecific glycosylation with the modifications: glycosylation was carried out at −40 °C. Catalyst **1d** (7 mol %) and secondary acceptor **S31** (0.24 mmol, 2 equiv) were used. The *dr* of the corresponding glycal α-epoxide is >20:1. The *dr* of the glycosylation product determined based on crude reaction mixture is >20:1. The desired product **30** was purified through a silica gel flash column (hexanes/EtOAc: from 20:1 to 2:1) as white foam (86.6 mg, 75% yield).

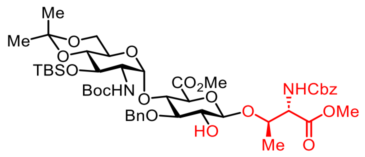

30

***N*-benzyloxycarbonyl-*O*-[2-*tert*-butoxycarbonylamino-3-*O*-*tert*-butyldimethylsilyl-4,6-*O*-isopropylidene-2-deoxy-α-D-glucopyranosyl-(1→4)-(methyl 3-*O*-benzyl-β-D-glucopyranosyluronate)]-L-threonine methyl ester** (**30**): $[\alpha]_D^{23}$ +6.3 (acetone, *c* = 1.0); IR ν_{max} (neat)/cm^{-1}: 3443 (w), 2928 (w), 1751 (s), 1722 (s), 1504 (m), 1171 (s), 1130 (s), 1025 (s), 875 (s), 836 (s), 777 (m); ^{1}H NMR (400 MHz, $CDCl_3$) δ 7.38 – 7.27 (m, 10H), 5.61 (d, *J* = 9.3 Hz, 1H), 5.31 (d, *J* = 3.8 Hz, 1H), 5.13 (s, 2H), 4.90 (d, *J* = 10.1 Hz, 1H), 4.83 (d, *J* = 10.7 Hz, 1H), 4.74 (d, *J* = 10.7 Hz, 1H), 4.48 – 4.39 (m, 1H), 4.36 (dd, *J* = 9.4, 2.3 Hz, 1H), 4.28 (d, *J* = 7.6 Hz, 1H), 3.97 (t, *J* = 9.2 Hz, 1H), 3.90 – 3.73 (m, 6H), 3.67 (s, 3H), 3.71 – 3.40 (m, 5H), 3.22 (td, *J* = 9.9, 5.1 Hz, 1H), 2.36 (brs, 1H), 1.44 (s, 3H), 1.36 (s, 3H), 1.34 (s, 9H), 1.26 (d, *J* = 6.1 Hz, 3H), 0.87 (s, 9H), 0.07 (s, 3H), 0.05 (s, 3H); ^{13}C NMR (100 MHz, $CDCl_3$) δ 170.8, 168.3, 156.9, 155.2, 137.9, 136.4, 128.72 (2C), 128.67 (2C), 128.4 (2C), 128.3 (2C), 128.2, 128.1, 101.0, 99.5, 99.4, 83.7, 79.8, 74.9, 74.83, 74.76, 74.6, 74.5, 74.1, 71.5, 67.3, 64.5, 62.0, 58.7, 55.5, 52.73, 52.68, 29.3, 28.6 (3C), 25.9 (3C), 19.1, 18.4, 17.5, -4.0, -4.9; HRMS: m/z (ESI) calcd for $C_{47}H_{71}N_2O_{17}Si^+$, $[M + H]^+$, 963.4517, found 963.4532. $^{1}J_{^{13}C1-H1}$ = 176.0 Hz, 160.6 Hz.

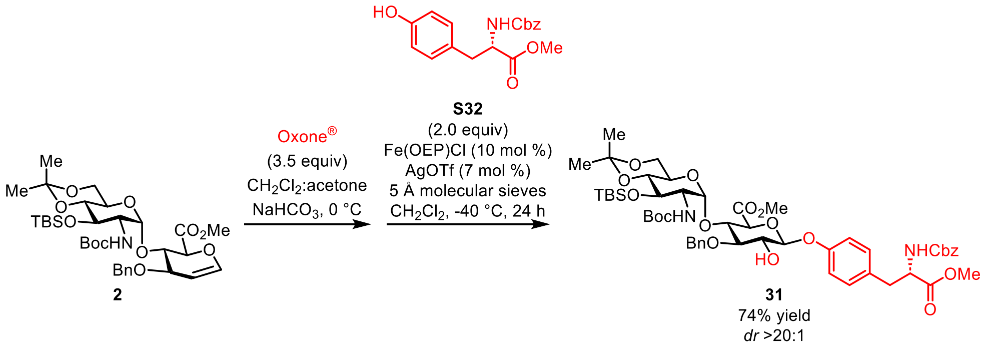

S32 was synthesized according to a literature procedure.[18]

The reaction was carried out on a 0.12 mmol scale using the glycal as the limiting reagent by following the **Procedure A** for epoxidation and **Procedure C** for the iron-catalyzed stereospecific glycosylation with the modifications: glycosylation was carried out at −40 °C. Catalyst **1d** (7 mol %) and acceptor **S32** (0.24 mmol, 2 equiv) were used. The *dr* of the corresponding glycal α-epoxide is >20:1. The *dr* of the glycosylation product determined based on crude reaction mixture is >20:1. The desired product **31** was purified through a silica gel flash column (hexanes/EtOAc: from 20:1 to 2:1) as white foam (90.9 mg, 74% yield).

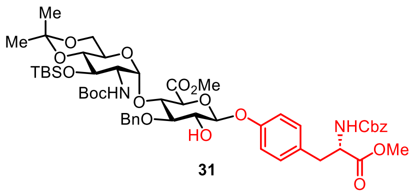

31

***N*-benzyloxycarbonyl-*O*-[2-*tert*-butoxycarbonylamino-3-*O*-*tert*-butyldimethylsilyl-4,6-*O*-isopropylidene-2-deoxy-α-D-glucopyranosyl-(1→4)-(methyl 3-*O*-benzyl-β-D-glucopyranosyluronate)]-L-tyrosine methyl ester** (**31**): $[\alpha]_D^{23}$ +10.7 (acetone, *c* = 1.0); IR ν_{max} (neat)/cm^{-1}: 2928 (w), 2360 (w), 1721 (m), 1508 (m), 1212 (m), 1172 (s), 1130 (s), 1074 (s), 1025 (s), 836 (s), 779 (m); ^{1}H NMR (400 MHz, acetone-d_6) δ 7.50 – 7.38 (m, 2H), 7.38 – 7.26 (m, 8H), 7.20 (d, *J* = 8.6 Hz, 2H), 6.98 (d, *J* = 8.6 Hz, 2H), 6.60 (d, *J* = 8.5 Hz, 1H), 5.86 (d, *J* = 10.0 Hz, 1H), 5.51 (d, *J* = 3.8 Hz, 1H), 5.21 (brs, 1H), 5.15 – 5.07 (m, 2H), 5.03 (s, 2H), 4.76 (d, *J* = 10.1 Hz, 1H), 4.54 – 4.37 (m, 1H), 4.31 (d, *J* = 9.1 Hz, 1H), 4.07 – 3.94 (m, 2H), 3.90 – 3.75 (m, 5H), 3.74 – 3.63 (m, 6H), 3.52 (dd, *J* = 9.9, 8.7 Hz, 1H), 3.37 – 3.22 (m, 1H), 3.12 (dd, *J* = 13.9, 5.3 Hz, 1H), 2.95 (dd, *J* = 13.9, 9.1 Hz, 1H), 1.50 (s, 3H), 1.35 (s, 9H), 1.34 (s, 3H), 0.90 (s, 9H), 0.11 (s, 3H), 0.09 (s, 3H); ^{13}C NMR (100 MHz, acetone-d_6) δ 173.0, 169.7, 157.2, 156.8, 156.2, 139.3, 138.1, 132.2, 131.2 (two peaks overlapped, 4C), 129.4 (2C), 129.2 (2C), 129.1 (2C), 128.7, 128.53, 128.46, 117.3 (2C), 101.9, 99.4, 84.9, 79.3, 75.53, 75.49, 75.3, 75.0, 74.5, 72.2, 66.7, 65.0, 62.4, 56.6, 56.5, 53.0, 52.3, 37.4, 29.6, 28.8 (3C), 26.4 (3C), 19.3, 18.9, -3.8, -4.8; HRMS: m/z (ESI) calcd for $C_{52}H_{73}N_2O_{17}Si^+$, [M + H]$^+$, 1025.4673, found 1025.4701. $^1J_{^{13}C1\text{–}H1}$ = 161.6 Hz, 175.8 Hz.

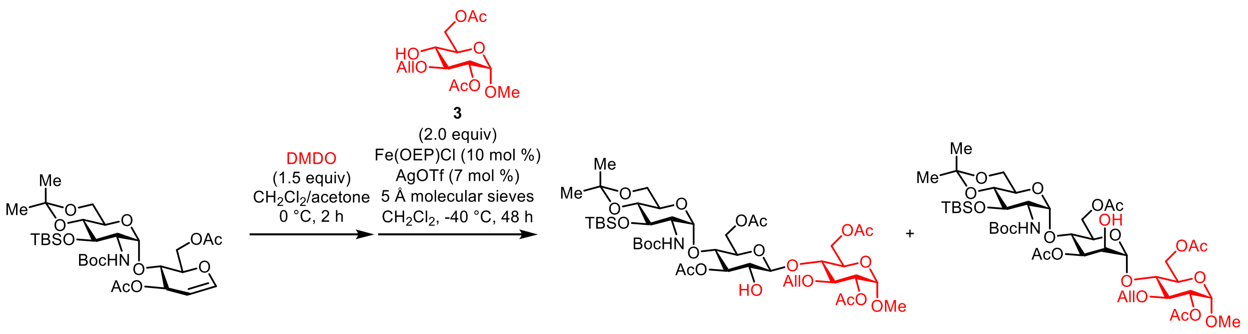

S33 was synthesized according to a literature procedure.[1]

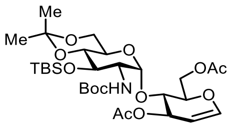

S33

3,6-Di-*O*-acetyl-1,5-anhydro-4-*O*-(2-*tert*-butoxycarbonylamino-3-*O*-*tert*-butyldimethylsilyl-4,6-*O*-isopropylidene-2-deoxy-α-D-glucopyranosyl)-2-deoxy-D-*arabino*-hex-1-enitol (S33): $[\alpha]_D^{23}$ +47.3 (acetone, *c* = 1.0); IR ν_{max} (neat)/cm^{-1}: 2955 (w), 2929 (w), 1745 (m), 1720 (m), 1651 (w), 1502 (m), 1367 (m), 1220 (s), 1170 (s), 1129 (s), 1068 (s), 1032 (s), 1017 (s), 944 (w), 876 (m), 837 (s), 778 (m); ^{1}H NMR (400 MHz, $CDCl_3$) δ 6.46 (d, *J* = 6.2 Hz, 1H), 5.17 (t, *J* = 4.4 Hz, 1H), 5.09 (d, *J* = 3.9 Hz, 1H), 4.87 (dd, *J* = 6.2, 3.7 Hz, 1H), 4.55 (d, *J* = 9.9 Hz, 1H), 4.39 (dd, *J* = 12.0, 3.4 Hz, 1H), 4.34 – 4.20 (m, 2H), 4.02 (t, *J* = 5.6 Hz, 1H), 3.84 (dd, *J* = 9.0, 4.0 Hz, 1H), 3.80 (d, *J* = 4.9 Hz, 1H), 3.70 (t, *J* = 10.2 Hz, 1H), 3.63 – 3.43 (m, 3H), 2.11 (s, 3H), 2.05 (s, 3H), 1.46 (s, 3H), 1.43 (s, 9H), 1.38 (s, 3H), 0.86 (s, 9H), 0.05 (s, 3H), 0.04 (s, 3H); ^{13}C NMR (100 MHz, $CDCl_3$) δ 170.4, 170.1, 155.2, 145.6, 99.4, 98.9, 98.2, 79.8, 74.7, 74.6, 71.1, 70.9, 68.1, 64.7, 62.2, 61.9, 55.4, 28.9, 28.3 (3C), 25.7 (3C), 21.1, 20.7, 18.9, 18.2, -4.2, -5.1; HRMS: m/z (ESI) calcd for $C_{30}H_{52}NO_{12}Si^+$, $[M + H]^+$, 646.3253, found 646.3255. $^1J_{^{13}C1-H1}$ = 173.0 Hz.

The reaction was carried out on a 0.12 mmol scale using the glycal as the limiting reagent by following **Procedure B** for epoxidation and **Procedure C** for the iron-catalyzed stereospecific glycosylation with the modifications: glycosylation was carried out at −40 °C. Catalyst **1d** (7 mol %) and secondary acceptor **3** (0.24 mmol, 2 equiv) were used. The *dr* of the corresponding glycal α-epoxide is 4.7:1. The *dr* of the glycosylation products determined based on crude reaction mixture is >20:1. The desired products **32** and **32'** were purified through a silica gel flash column (hexanes/EtOAc: from 20:1 to 2:1) both as white foam (68.3 mg, 58% yield for **32**, and 15.9 mg, 14% yield for **32'**).

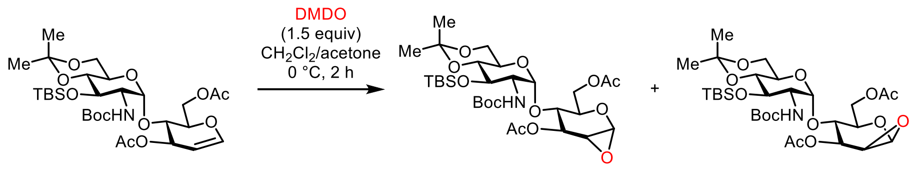

S33 **S34a** *dr* : 4.7:1 **S34b**

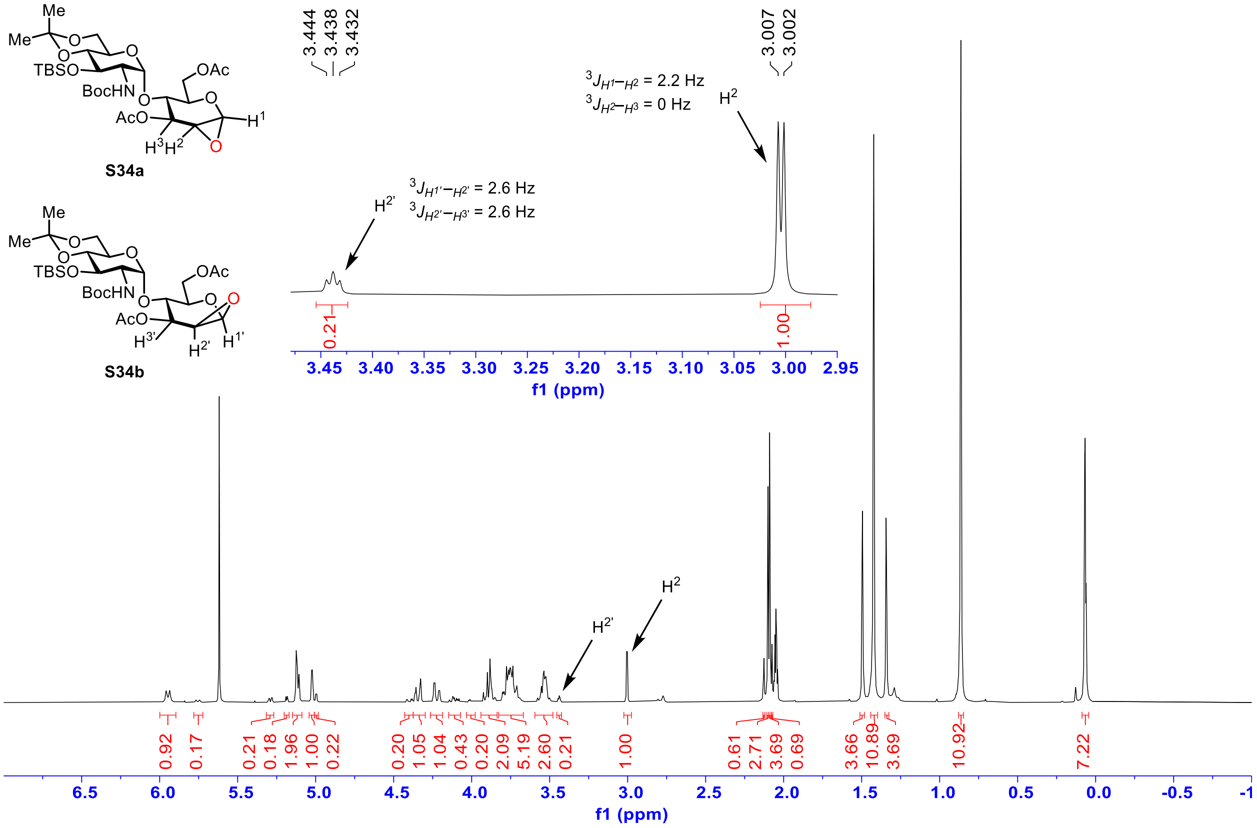

The stereochemistry of **S34a** and **S34b** was determined through ^{1}H NMR analysis. The C(2)–H chemical shift of **S34a** appears around 3.0 ppm, consistent with a glucal α-epoxide, while the corresponding chemical shift for **S34b** is around 3.4 ppm, which matches a glucal β-epoxide.[3]

The assignment is further corroborated by the $^{3}J_{H^1-H^2}$ and $^{3}J_{H^2-H^3}$ coupling constants: for **S34a** they are 2.2 Hz and 0 Hz, respectively, whereas **S34b** shows a comparable $^{3}J_{H^1-H^2}$ and $^{3}J_{H^2-H^3}$ value of 2.6 Hz.

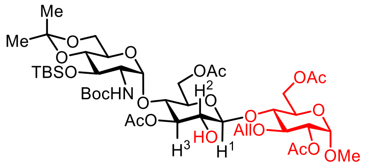

32

Methyl 2-*tert*-butoxycarbonylamino-3-*O*-*tert*-butyldimethylsilyl-4,6-*O*-isopropylidene-2-deoxy-α-D-glucopyranosyl-(1→4)-3,6-di-*O*-acetyl-β-D-glucopyranosyl-(1→4)-2,6-di-*O*-acetyl-3-*O*-allyl-α-D-glucopyranoside (**32**): $[\alpha]_D^{23}$ +79.2 (acetone, *c* = 1.0); IR ν_{max} (neat)/cm^{-1}: 3447 (w), 2970 (w), 2930 (w), 2360 (w), 1740 (s), 1506 (w), 1367 (s), 1228 (s), 1170 (m), 1128 (m), 1031 (s), 987 (s), 874 (m), 837 (m), 778 (m); ^{1}H NMR (400 MHz, $CDCl_3$) δ 5.86 (ddt, *J* = 17.3, 10.3, 5.2 Hz, 1H), 5.22 (dd, *J* = 17.3, 1.9 Hz, 1H), 5.18 – 5.02 (m, 3H), 4.84 (d, *J* = 3.6 Hz, 1H), 4.78 (dd, *J* = 9.9, 3.6 Hz, 1H), 4.56 (d, *J* = 8.2 Hz, 1H), 4.54 (d, *J* = 10.7 Hz, 1H), 4.49 (d, *J* = 11.2 Hz, 1H), 4.41 (d, *J* = 12.1 Hz, 1H), 4.38 – 4.26 (m, 2H), 4.23 – 4.11 (m, 2H), 3.93 – 3.72 (m, 5H), 3.72 – 3.61 (m, 2H), 3.56 (d, *J* = 9.4 Hz, 1H), 3.52 – 3.40 (m, 4H), 3.35 (s, 3H), 3.20 (d, *J* = 3.9 Hz, 1H), 2.13 (s, 3H), 2.11 (s, 3H), 2.10 (s, 3H), 2.07 (s, 3H), 1.45 (s, 9H), 1.44 (s, 3H), 1.37 (s, 3H), 0.85 (s, 9H), 0.05 (s, 3H), 0.04 (s, 3H); ^{13}C NMR (100 MHz, $CDCl_3$) δ 171.3, 171.2, 170.4, 170.3, 155.5, 135.1, 116.1, 103.4, 100.1, 99.5, 96.9, 80.1, 78.1, 77.8, 77.7, 74.8, 73.8, 73.41, 73.40, 73.3, 72.8, 70.9, 68.8, 65.1, 63.02, 62.97, 62.2, 55.8, 55.2, 29.0, 28.5 (3C), 25.9 (3C), 21.2, 21.1, 21.0, 20.9, 19.0, 18.3, -4.0, -4.8; HRMS: m/z (ESI) calcd for $C_{44}H_{74}NO_{21}Si^{+}$, $[M + H]^{+}$, 980.4517, found 980.4493. $^{1}J_{^{13}C1-H1}$ = 163.0 Hz, 175.0 Hz, 174.3 Hz. $^{3}J_{H^1-H^2}$ = 8.2 Hz. $^{3}J_{H^2-H^3}$ = 9.2 Hz.

The spectroscopic data of **32** align with the 1,2-*trans*-glycoside by the iron-catalyzed stereospecific glycosylation of the glycal α-epoxide. In this product, H^1 adopts an axial position, with dihedral angles of H^1–H^2 and H^2–H^3 approaching 180°.

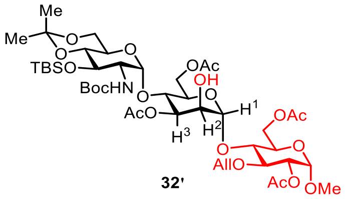

32'

Methyl 2-*tert*-butoxycarbonylamino-3-*O*-*tert*-butyldimethylsilyl-4,6-*O*-isopropylidene-2-deoxy-α-D-glucopyranosyl-(1→4)-3,6-di-*O*-acetyl-α-D-mannopyranosyl-(1→4)-2,6-di-*O*-acetyl-3-*O*-allyl-α-D-glucopyranoside (**32'**): $[\alpha]_D^{23}$ +82.7 (acetone, *c* = 0.3); IR ν_{max} (neat)/cm^{-1}: 3456 (w), 2970 (w), 2361 (m), 1739 (s), 1436 (w), 1366 (s), 1229 (s), 1212 (s), 1039 (m), 877 (m), 669 (w); ^{1}H NMR (400 MHz, $CDCl_3$) δ 5.87 (ddt, *J* = 17.2, 10.5, 5.3 Hz, 1H), 5.24 (dd, *J* = 17.2, 1.8 Hz, 1H), 5.19 – 5.08 (m, 4H), 4.88 (d, *J* = 3.6 Hz, 1H), 4.77 (dd, *J* = 10.0, 3.6 Hz, 1H), 4.65 (d, *J* = 10.0 Hz, 1H), 4.48 (dd, *J* = 12.2, 2.0 Hz, 1H), 4.41 (dd, *J* = 12.2, 2.3 Hz, 1H), 4.33 – 4.09 (m, 5H), 4.06 (dd, *J* = 2.2, 2.3 Hz, 1H), 3.97 – 3.75 (m, 6H), 3.71 – 3.63 (m, 2H), 3.56 – 3.47 (m, 3H), 3.39 (s, 3H), 2.131 (s, 3H), 2.126 (s, 3H), 2.12 (s, 3H), 2.10 (s, 3H), 1.454 (s, 9H), 1.446 (s, 3H), 1.38 (s, 3H), 0.86 (s, 9H), 0.07 (s, 3H), 0.06 (s, 3H); ^{13}C NMR (100 MHz, $CDCl_3$) δ 170.9, 170.6, 170.3, 169.9, 155.5, 134.7, 116.7, 101.9, 100.0, 99.5, 97.0, 80.0, 79.4, 78.4, 74.8, 74.3, 74.2, 73.8, 71.2, 70.7, 70.4, 69.3, 68.3, 65.0, 63.2 (two peaks overlapped, 2C), 62.2, 55.9, 55.4, 29.1, 28.6 (3C), 26.0 (3C), 21.4, 21.2, 20.96, 20.95, 19.1, 18.4, -3.9, -4.7; HRMS: m/z (ESI) calcd for $C_{44}H_{74}NO_{21}Si^+$, $[M + H]^+$, 980.4517, found 980.4505. $^1J_{^{13}C1-H1}$ = 173.0 Hz, 172.7 Hz, 171.7 Hz. $^3J_{H^1-H^2}$ = 2.2 Hz. $^3J_{H^2-H^3}$ = 2.3 Hz.

The spectroscopic data of **32'** align with the 1,2-*trans*-glycoside by the iron-catalyzed stereospecific glycosylation of the glycal β-epoxide. In this product, H^1 adopts an equatorial position, with dihedral angles of H^1–H^2 and H^2–H^3 approaching 60°.

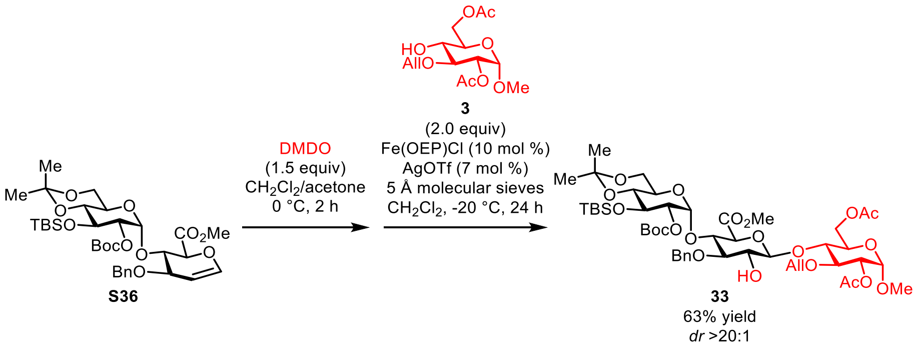

S36 was synthesized according to the following procedure.

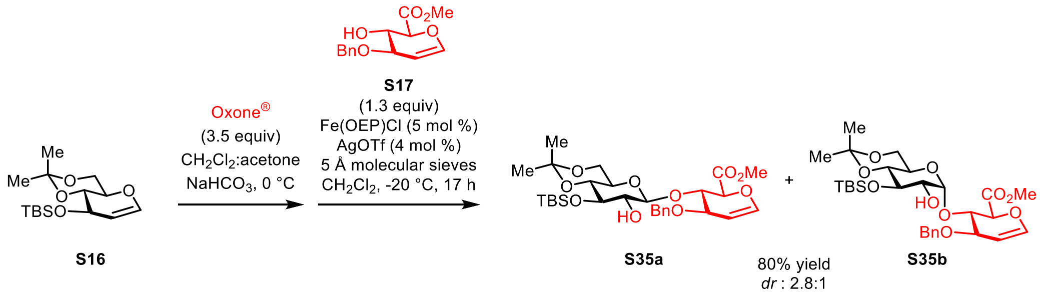

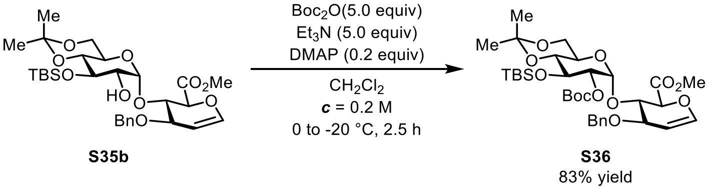

To a 500 mL flask equipped with a stir bar at 0 °C were added glycal **S16** (901.4 mg, 3.0 mmol, 1.0 equiv) in CH_2Cl_2/acetone mixture (v/v: 5:1, 50 mL) and saturated aqueous $NaHCO_3$ solution (85 mL). Oxone® ($KHSO_5 \cdot 0.5KHSO_4 \cdot 0.5K_2SO_4$) (6.46 g, 10.5 mmol, 3.5 equiv) in H_2O (55 mL) was then added to the mixture dropwise. After stirring vigorously at 0 °C for 2 h, the reaction mixture was extracted with CH_2Cl_2 (75 mL × 3). The combined organic phase was dried over anhydrous Na_2SO_4 and concentrated *in vacuo*. The residue was further azeotropically dried with anhydrous toluene (25 mL). The obtained glycal epoxide was directly used in the next step.

To a 100 mL flame-dried round bottom flask equipped with a stir bar were added glycosyl acceptor **S17** (1.03 g, 3.9 mmol, 1.3 equiv), Fe(OEP)Cl (80.1 mg, 0.15 mmol, 5 mol %), AgOTf (30.8 mg, 0.12 mmol, 4 mol %), and freshly activated 5 Å molecular sieves, powder (*ca.* 1.0 g). After the vial was evacuated and backfilled with N_2 three times, anhydrous CH_2Cl_2 (15 mL) was added. The solution was stirred at -78 °C for 20 min before a solution of the glycal epoxide (3.0 mmol, 1.0 equiv) obtained from the last step in anhydrous CH_2Cl_2 (5 mL) was added at -78 °C dropwise. The reaction mixture was stirred at -20 °C for 17 h, and then quenched with MeOH (1.0 mL) and imidazole (180 mg in 20 mL CH_2Cl_2) at the same temperature. The mixture was filtered through a pad of Celite® and eluted with EtOAc (30 mL × 2). The organic layer was then concentrated *in vacuo* and the residue was purified through column chromatography (hexanes/acetone: from 100:1 to 5:1) to afford **S35a** (1.01 g, 59% yield) and the desired product **S35b** (361 mg, 21% yield) both as white foam.

To a solution of **S35b** (762.6 mg, 1.31 mmol, 1.0 equiv) and DMAP (32.0 mg, 0.262 mmol, 0.2 equiv) in anhydrous CH_2Cl_2 (6.5 mL) were added Et_3N (0.91 mL, 6.55 mmol, 5.0 equiv) at 0 °C. After stirring at 0 °C for 10 min, Boc_2O (1.51 mL, 6.55 mmol, 5.0 equiv) was added dropwise. The reaction mixture was slowly warmed to room temperature and stirred for another 2.5 h, then quenched with saturated aqueous $NaHCO_3$ solution (10 mL). The organic phase was separated from the aqueous phase, the aqueous phase was extracted with CH_2Cl_2 (10 mL × 3). The combined organic layer was washed with brine, dried over anhydrous Na_2SO_4, and then concentrated *in vacuo.* The desired product **S36** was obtained through a silica gel flash column (hexanes/acetone: from 20:1 to 10:1) as white foam (676.5 mg, 83% yield).

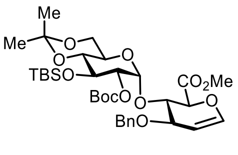

S36

Methyl 3-*O*-benzyl-4-*O*-(2-*O*-*tert*-butoxycarbonyl-3-*O*-*tert*-butyldimethylsilyl-4,6-*O*-isopropylidene-α-D-glucopyranosyl)-D-glucuronal (**S36**): $[\alpha]_D^{23}$ +41.2 (acetone, *c* = 1.0); IR ν_{max} (neat)/cm^{-1}: 2953 (w), 2929 (w), 1739 (m), 1650 (w), 1369 (w), 1278 (m), 1255 (m), 1168

(s), 1105 (s), 1083 (s), 977 (m), 869 (s), 837 (s), 779 (m); ^{1}H NMR (400 MHz, $CDCl_3$) δ 7.35 – 7.24 (m, 5H), 6.63 (d, *J* = 6.3 Hz, 1H), 5.31 (d, *J* = 4.1 Hz, 1H), 4.97 (ddd, *J* = 6.4, 5.0, 1.5 Hz, 1H), 4.85 (dd, *J* = 3.3, 1.3 Hz, 1H), 4.53 – 4.45 (m, 2H), 4.41 (d, *J* = 11.2 Hz, 1H), 4.34 – 4.30 (m, 1H), 3.89 – 3.81 (m, 3H), 3.78 – 3.63 (m, 2H), 3.56 (s, 3H), 3.48 (t, *J* = 9.1 Hz, 1H), 1.46 (s, 3H), 1.45 (s, 9H), 1.39 (s, 3H), 0.86 (s, 9H), 0.07 (s, 3H), 0.06 (s, 3H); ^{13}C NMR (100 MHz, $CDCl_3$) δ 168.5, 153.3, 145.3, 137.9, 128.5 (2C), 128.1 (2C), 127.9, 99.5, 98.6, 97.7, 82.5, 76.5, 74.3, 73.9, 73.7, 70.1, 69.7, 68.2, 64.0, 62.3, 52.3, 29.1, 27.9 (3C), 25.9 (3C), 19.1, 18.4, -4.2, -5.0; HRMS: m/z (ESI) calcd for $C_{34}H_{53}O_{12}Si^+$, $[M + H]^+$, 681.3301, found 681.3294. $^1J_{^{13}C1-H1}$ = 170.5 Hz.

The reaction was carried out on a 0.12 mmol scale using the glycal as the limiting reagent by following **Procedure B** for epoxidation and **Procedure C** for the iron-catalyzed stereospecific glycosylation. Catalyst **1d** (7 mol %) and secondary acceptor **S17** (0.24 mmol, 2 equiv) were used. The *dr* of the corresponding glycal α-epoxide is 10:1. The *dr* of the glycosylation product determined based on crude reaction mixture is >20:1. The desired product **33** was purified through a silica gel flash column (hexanes/EtOAc: from 20:1 to 2:1) as white foam (76.6 mg, 63% yield).

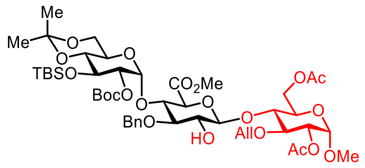

33

Methyl 2-*O*-*tert*-butoxycarbonyl-3-*O*-*tert*-butyldimethylsilyl-4,6-*O*-isopropylidene-α-D-glucopyranosyl-(1→4)-(methyl 3-*O*-benzyl-β-D-glucopyranosyluronate)-(1→4)-2,6-di-*O*-acetyl-3-*O*-allyl-α-D-glucopyranoside (**33**): $[\alpha]_D^{23}$ +103.3 (acetone, *c* = 1.0); IR ν_{max} (neat)/cm^{-1}: 2929 (w), 2360 (w), 1743 (s), 1369 (m), 1235 (s), 1133 (s), 1027 (s), 976 (m), 870 (s), 779 (m); ^{1}H NMR (400 MHz, $CDCl_3$) δ 7.45 – 7.28 (m, 5H), 5.84 (ddt, *J* = 17.2, 10.6, 5.4 Hz, 1H), 5.50 (d, *J* = 4.0 Hz, 1H), 5.21 (dd, *J* = 17.2, 1.8 Hz, 1H), 5.11 (dd, *J* = 10.6, 1.8 Hz, 1H), 4.89 – 4.80 (m, 2H), 4.80 – 4.70 (m, 2H), 4.65 (dd, *J* = 9.6, 4.0 Hz, 1H), 4.51 – 4.39 (m, 2H), 4.37 – 4.24 (m, 2H), 4.14 – 4.00 (m, 2H), 3.95 (d, *J* = 9.6 Hz, 1H), 3.92 – 3.75 (m, 4H), 3.77 (s, 3H), 3.71 – 3.57

(m, 3H), 3.56 – 3.43 (m, 2H), 3.37 – 3.28 (m, 4H), 2.69 (d, *J* = 3.0 Hz, 1H), 2.10 (s, 3H), 2.09 (s, 3H), 1.45 (s, 3H), 1.37 (s, 9H), 1.37 (s, 3H), 0.86 (s, 9H), 0.08 (s, 3H), 0.06 (s, 3H); ^{13}C NMR (100 MHz, $CDCl_3$) δ 171.2, 170.3, 168.6, 153.2, 138.4, 135.2, 128.8 (2C), 128.3 (2C), 128.1, 116.3, 103.7, 99.5, 97.3, 97.0, 84.3, 82.8, 78.4, 78.0, 76.0, 75.5, 75.2, 75.0, 74.3, 74.2, 74.1, 73.3, 70.1, 68.8, 64.1, 62.9, 62.0, 55.3, 52.7, 29.2, 27.9 (3C), 25.9 (3C), 21.14, 21.08, 19.1, 18.4, -4.2, -4.9; HRMS: m/z (ESI) calcd for $C_{48}H_{75}O_{21}Si^+$, $[M + H]^+$, 1015.4565, found 1015.4587. $^1J_{^{13}C1-H1}$ = 159.8 Hz, 176.3 Hz, 174.4 Hz.

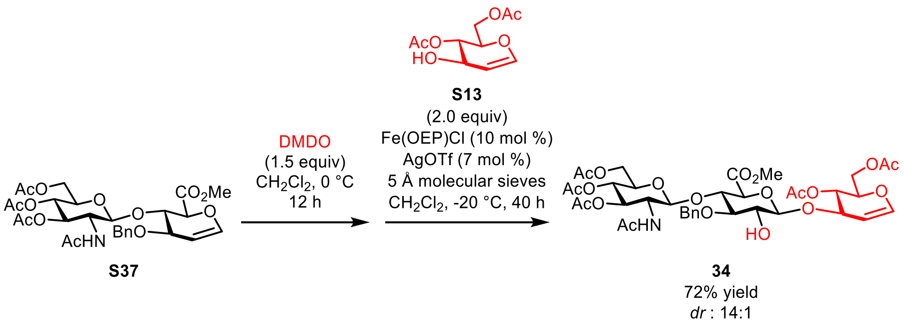

S37 was synthesized according to a literature procedure.[19]

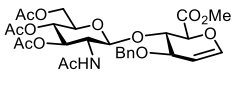

S37

Methyl 3-*O*-benzyl-4-*O*-(2-acetamido-3,4,6-tri-*O*-acetyl-2-deoxy-β-D-glucopyranosyl)-D-glucuronal (**S37**): $[\alpha]_D^{23}$ −3.9 (acetone, *c* = 1.0); IR ν_{max} (neat)/cm^{-1}: 2929 (s), 2876 (m), 2360 (m), 1741 (m), 1653 (w), 1367 (w), 1232 (m), 1026 (m); ^{1}H NMR (400 MHz, $CDCl_3$) δ 7.36 – 7.17 (m, 5H), 6.57 (d, *J* = 6.3 Hz, 1H), 5.67 (d, *J* = 8.2 Hz, 1H), 5.46 (t, *J* = 10.0 Hz, 1H), 5.15 (d, *J* = 8.3 Hz, 1H), 5.04 (t, *J* = 9.6 Hz, 1H), 4.99 (t, *J* = 5.7 Hz, 1H), 4.72 (s, 1H), 4.57 – 4.40 (m, 3H), 4.26 (dd, *J* = 12.3, 4.7 Hz, 1H), 4.14 (dd, *J* = 12.3, 2.4 Hz, 1H), 3.89 (d, *J* = 4.8 Hz, 1H), 3.83 – 3.73 (m, 1H), 3.62 (dt, *J* = 10.7, 8.2 Hz, 1H), 3.54 (s, 3H), 2.06 (s, 3H), 2.02 (s, 6H, two peaks overlapped), 1.92 (s, 3H); ^{13}C NMR (100 MHz, $CDCl_3$) δ 170.81, 170.76, 170.7, 169.6, 168.3, 145.1, 138.0, 128.4 (2C), 128.0 (2C), 127.8, 98.9, 98.7, 72.6, 72.4, 72.2, 71.8, 69.8, 68.8,

68.0, 62.2, 55.5, 52.4, 23.4, 20.9, 20.81, 20.77; HRMS: m/z (ESI) calcd for $C_{28}H_{36}NO_{13}^+$, [M + H]$^+$, 594.2181, found 594.2192. $^1J_{^{13}C1\text{–}H1}$ = 164.2 Hz.

The reaction was carried out on a 0.12 mmol scale using the glycal as the limiting reagent by following **Procedure B** for epoxidation and **Procedure C** for the iron-catalyzed stereospecific glycosylation. Catalyst **1d** (7 mol %) and secondary acceptor **S13** (0.24 mmol, 2 equiv) were used. The *dr* of the corresponding glycal α-epoxide is >20:1. The *dr* of the glycosylation product determined based on crude reaction mixture is 14:1. The desired product **34** was purified through a silica gel flash column (hexanes/acetone: from 20:1 to 3:2) as white foam (72.5 mg, 72% yield).

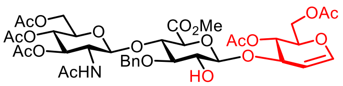

34

4,6-Di-*O*-acetyl-1,5-anhydro-3-*O*-[2-acetamido-3,4,6-tri-*O*-acetyl-2-deoxy-β-D-glucopyranosyl-(1→4)-(methyl 3-*O*-benzyl-β-D-glucopyranosyluronate)]-2-deoxy-D-*arabino*-hex-1-enitol (**34**): $[\alpha]_D^{23}$ −24.6 (acetone, *c* = 1.0); IR ν_{max} (neat)/cm^{-1}: 3355 (w), 2921 (w), 1741 (m), 1673 (m), 1372 (m), 1238 (s), 1222 (s), 1152 (m), 1073 (s), 1043 (s), 912 (w); ^{1}H NMR (400 MHz, $CDCl_3$) δ 7.45 – 7.20 (m, 5H), 6.47 (d, *J* = 6.2 Hz, 1H), 5.70 (d, *J* = 9.0 Hz, 1H), 5.26 (t, *J* = 4.3 Hz, 1H), 5.14 – 5.00 (m, 2H), 4.97 (d, *J* = 11.6 Hz, 1H), 4.93 (dd, *J* = 6.2, 3.6 Hz, 1H), 4.80 (d, *J* = 11.6 Hz, 1H), 4.68 (d, *J* = 8.4 Hz, 1H), 4.48 (d, *J* = 7.5 Hz, 1H), 4.35 – 4.25 (m, 3H), 4.21 (t, *J* = 4.1 Hz, 1H), 4.15 (dd, *J* = 12.3, 4.3 Hz, 1H), 4.07 – 3.97 (m, 2H), 3.97 – 3.88 (m, 2H), 3.84 (s, 3H), 3.60 – 3.43 (m, 3H), 2.89 (d, *J* = 2.8 Hz, 1H), 2.08 (s, 3H), 2.06 (s, 3H), 2.01 (s, 3H), 1.99 (s, 3H), 1.96 (s, 3H), 1.94 (s, 3H); ^{13}C NMR (100 MHz, $CDCl_3$) δ 170.9, 170.8, 170.6, 170.3, 169.8, 169.3, 169.2, 145.4, 138.8, 128.3 (2C), 127.5, 127.3 (2C), 100.9, 100.8, 97.9, 82.0, 78.4, 74.7, 74.0, 73.7, 73.2, 73.1, 71.9, 71.0, 68.1, 67.8, 61.8, 61.6, 54.3, 52.9, 23.2, 20.9, 20.8, 20.63, 20.59, 20.5; HRMS: m/z (ESI) calcd for $C_{38}H_{50}NO_{20}^+$, [M + H]$^+$, 840.2921, found 840.2918. $^1J_{^{13}C1\text{–}H1}$ = 159.7 Hz, 157.4 Hz.

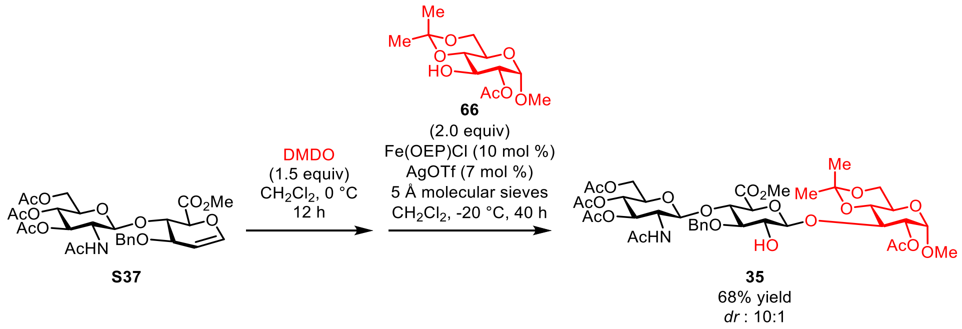

The reaction was carried out on a 0.12 mmol scale using the glycal as the limiting reagent by following the **Procedure B** for epoxidation and **Procedure C** for the iron-catalyzed stereospecific glycosylation. Catalyst **1d** (7 mol %) and secondary acceptor **66** (0.24 mmol, 2 equiv) were used. The *dr* of the corresponding glycal α-epoxide is >20:1. The *dr* of the glycosylation product determined based on crude reaction mixture is 10:1. The desired product **35** was purified through a silica gel flash column (hexanes/acetone: from 20:1 to 3:2) as white foam (72.2 mg, 68% yield).

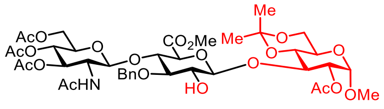

35

Methyl 2-acetamido-3,4,6-tri-*O*-acetyl-2-deoxy-β-D-glucopyranosyl-(1→4)-(methyl 3-*O*-benzyl-β-D-glucopyranosyluronate)-(1→3)-2-*O*-acetyl-4,6-*O*-isopropylidene-α-D-glucopyranoside (**35**): $[\alpha]_D^{23}$ +24.2 (acetone, *c* = 0.7); IR ν_{max} (neat)/cm^{-1}: 2923 (w), 2360 (w), 1743 (m), 1672 (w), 1369 (m), 1229 (s), 1034 (s), 909 (w), 740 (w), 699 (w); ^{1}H NMR (400 MHz, $CDCl_3$) δ 7.39 – 7.23 (m, 5H), 5.58 (d, *J* = 9.1 Hz, 1H), 5.13 – 4.99 (m, 2H), 4.92 (d, *J* = 11.7 Hz, 1H), 4.89 – 4.78 (m, 3H), 4.63 (d, *J* = 8.4 Hz, 1H), 4.41 (d, *J* = 8.0 Hz, 1H), 4.13 (dd, *J* = 12.3, 4.3 Hz, 1H), 4.09 – 3.96 (m, 3H), 3.92 (dd, *J* = 12.4, 2.3 Hz, 1H), 3.89 – 3.79 (m, 2H), 3.80 (s, 3H), 3.75 (t, *J* = 10.3 Hz, 1H), 3.72 – 3.63 (m, 2H), 3.54 – 3.42 (m, 3H), 3.36 (s, 3H), 2.79 (brs, 1H), 2.03 (s, 3H), 2.00 (s, 3H), 1.97 (s, 3H), 1.96 (s, 3H), 1.94 (s, 3H), 1.47 (s, 3H), 1.39 (s, 3H); ^{13}C NMR (100 MHz, $CDCl_3$) δ 171.1, 170.8, 170.54, 170.49, 169.5, 169.4, 139.0, 128.4 (2C), 127.6, 127.4 (2C), 102.5, 101.1, 100.0, 97.9, 82.1, 78.5, 75.6, 74.7, 74.6, 73.2, 72.9,

72.8, 72.1, 72.0, 68.2, 63.3, 62.4, 61.9, 55.4, 54.4, 52.9, 28.9, 23.4, 20.9, 20.77, 20.75, 20.7, 19.0; HRMS: m/z (ESI) calcd for $C_{40}H_{56}NO_{21}^{+}$, $[M + H]^{+}$, 886.3339, found 886.3345. $^{1}J_{^{13}C1\text{–}H1}$ = 161.4 Hz, 161.0 Hz, 170.9 Hz.

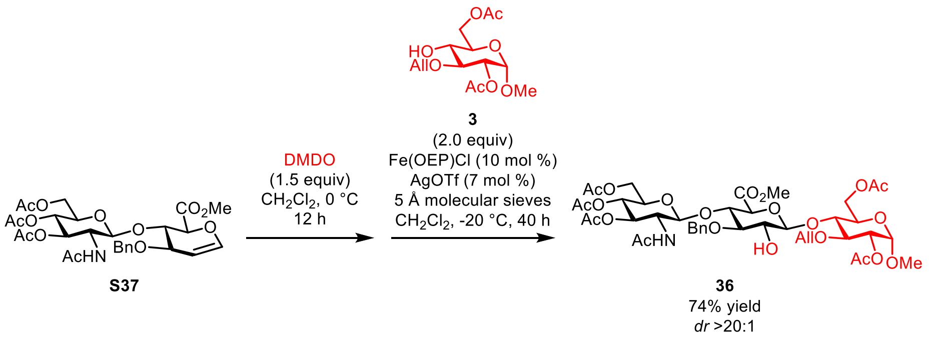

The reaction was carried out on a 0.12 mmol scale using the glycal as the limiting reagent by following the **Procedure B** for epoxidation and **Procedure C** for the iron-catalyzed stereospecific glycosylation. Catalyst **1d** (7 mol %) and secondary acceptor **3** (0.24 mmol, 2 equiv) were used. The *dr* of the corresponding glycal α-epoxide is >20:1. The *dr* of the glycosylation product determined based on crude reaction mixture is >20:1. The desired product **36** was purified through a silica gel flash column (hexanes/acetone: from 20:1 to 1:1) as white foam (82.1 mg, 74% yield).

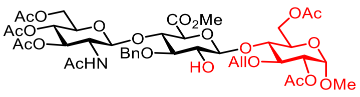

36

Methyl 2-acetamido-3,4,6-tri-*O*-acetyl-2-deoxy-β-D-glucopyranosyl-(1→4)-(methyl 3-*O*-benzyl-β-D-glucopyranosyluronate)-(1→4)-2,6-di-*O*-acetyl-3-*O*-allyl-α-D-glucopyranoside (**36**): $[\alpha]_D^{23}$ +55.9 (acetone, *c* = 1.0); IR ν_{max} (neat)/cm^{-1}: 3354 (w), 2933 (w), 1737 (s), 1666 (m), 1537 (w), 1440 (w), 1368 (s), 1240 (s), 1032 (s), 916 (m), 738 (w); ^{1}H NMR (400 MHz, $CDCl_3$) δ 7.35 – 7.29 (m, 5H), 5.82 (ddt, *J* = 17.3, 10.5, 5.2 Hz, 1H), 5.71 (d, *J* = 8.9 Hz, 1H), 5.19 (dd, *J* = 17.3, 1.9 Hz, 1H), 5.12 – 4.99 (m, 4H), 4.82 (d, *J* = 3.7 Hz, 1H), 4.77 (dd, *J* = 9.9, 3.6 Hz, 1H), 4.68 (d, *J* = 11.6 Hz, 1H), 4.57 (d, *J* = 8.5 Hz, 1H), 4.49 (d, *J* = 6.9 Hz, 1H), 4.44 (dd, *J* = 12.2,

2.1 Hz, 1H), 4.38 – 4.24 (m, 2H), 4.18 (dd, J = 12.8, 5.2 Hz, 1H), 4.12 (dd, J = 12.8, 5.2 Hz, 1H), 4.08 – 4.00 (m, 1H), 4.00 – 3.90 (m, 2H), 3.88 (d, J = 9.7 Hz, 1H), 3.86 – 3.76 (m, 5H), 3.72 – 3.63 (m, 1H), 3.57 – 3.51 (m, 1H), 3.51 – 3.40 (m, 2H), 3.34 (s, 3H), 2.76 (brs, 1H), 2.10 (s, 3H), 2.07 (s, 3H), 2.00 (s, 3H), 1.98 (s, 3H), 1.96 (s, 3H), 1.93 (s, 3H); ^{13}C NMR (100 MHz, $CDCl_3$) δ 171.04, 171.00, 170.7, 170.5, 170.2, 169.4, 169.3, 138.7, 135.1, 128.5 (2C), 127.8, 127.5 (2C), 115.9, 103.5, 101.0, 96.9, 82.3, 78.5, 78.3, 78.0, 75.1, 74.5, 74.0, 73.4, 73.2, 73.1, 72.0, 68.5, 68.1, 62.7, 61.8, 55.2, 54.3, 53.0, 23.3, 21.1, 21.0, 20.72, 20.65, 20.6; HRMS: m/z (ESI) calcd for $C_{42}H_{58}NO_{22}^+$, $[M + H]^+$, 928.3445, found 928.3457. $^1J_{^{13}C1\text{–}H1}$ = 161.8 Hz, 161.5 Hz, 173.2 Hz.

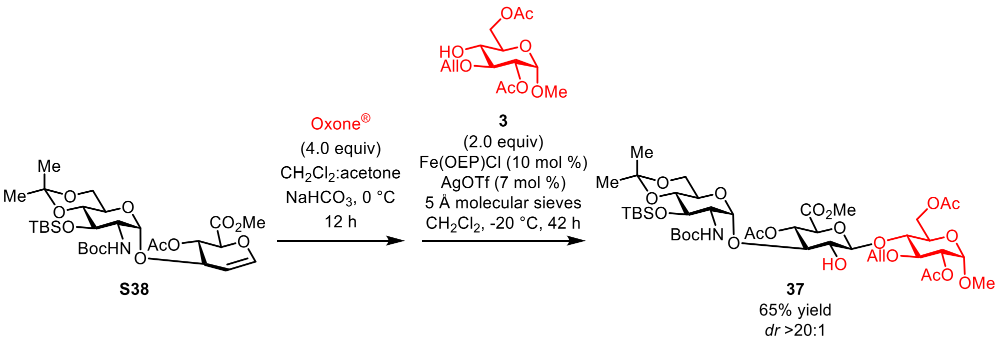

S38 was synthesized according to a literature procedure.[1]

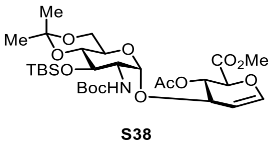

Methyl 4-*O*-acetyl-3-*O*-(2-*tert*-butoxycarbonylamino-3-*O*-*tert*-butyldimethylsilyl-4,6-*O*-isopropylidene-2-deoxy-α-D-glucopyranosyl)-D-glucuronal (**S38**): $[\alpha]_D^{25}$ +27.8 (acetone, c = 1.0); IR ν_{max} (neat)/cm^{-1}: 2930 (w), 1760 (m), 1707 (m), 1655 (m), 1520 (m), 1370 (m), 1242 (s), 1165 (m), 1131 (s), 1032 (m), 1012 (s), 914 (s), 795 (m); ^{1}H NMR (400 MHz, $CDCl_3$) δ 6.54 (d, J = 6.3 Hz, 1H), 5.36 (td, J = 3.1, 1.4 Hz, 1H), 4.95 – 4.81 (m, 2H), 4.79 – 4.74 (m, 1H), 4.70 (d, J = 10.1 Hz, 1H), 3.86 (ddd, J = 5.1, 2.6, 1.3 Hz, 1H), 3.80 – 3.70 (m, 5H), 3.65 (t, J = 10.3 Hz, 1H), 3.56 (td, J = 9.6, 5.0 Hz, 1H), 3.51 – 3.39 (m, 2H), 2.06 (s, 3H), 1.41 (s, 12H), 1.34 (s, 3H), 0.81 (s, 9H), 0.01 (s, 3H), 0.00 (s, 3H); ^{13}C NMR (100 MHz, $CDCl_3$) δ 169.5, 167.8, 155.3,

144.8, 100.1, 99.9, 99.3, 79.39, 74.8, 72.7, 71.1, 68.6, 68.0, 64.2, 62.2, 55.2, 52.6, 28.9, 28.4 (3C), 25.6 (3C), 20.8, 18.8, 18.1, -4.3, -5.1; HRMS: m/z (ESI) calcd for $C_{29}H_{50}NO_{12}Si^{+}$, [M + H]$^{+}$, 632.3097, found 632.3088. $^{1}J_{^{13}C1-H1}$ = 172.2 Hz.

The reaction was carried out on a 0.12 mmol scale using the glycal as the limiting reagent by following **Procedure A** for epoxidation and **Procedure C** for the iron-catalyzed stereospecific glycosylation with the modifications: Oxone® (4.0 equiv) (7 mol %) and secondary acceptor **3** (0.24 mmol, 2 equiv) were used for epoxidation. Catalyst **1d** (7 mol %) and secondary acceptor **3** (0.24 mmol, 2 equiv) were used. The *dr* of the corresponding glycal α-epoxide is 9:1. The *dr* of the glycosylation product determined based on crude reaction mixture is >20:1.The desired product **37** was purified through a silica gel flash column (hexanes/EtOAc: from 20:1 to 2:1) as white foam (75.3 mg, 65% yield).

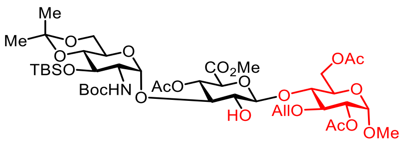

37

Methyl 2-*O*-*tert*-butoxycarbonyl-3-*O*-*tert*-butyldimethylsilyl-4,6-*O*-isopropylidene-α-D-glucopyranosyl-(1→3)-(methyl 4-*O*-acetyl-β-D-glucopyranosyluronate)-(1→4)-2,6-di-*O*-acetyl-3-*O*-allyl-α-D-glucopyranoside (**37**): $[\alpha]_D^{23}$ +66.6 (acetone, *c* = 1.0); IR ν_{max} (neat)/cm^{-1}: 2937 (w), 2360 (w), 1741 (s), 1367 (s), 1229 (s), 1217 (s), 1129 (m), 1076 (m), 1031 (s), 860 (m); ^{1}H NMR (400 MHz, $CDCl_3$) δ 5.89 – 5.75 (m, 1H), 5.19 (d, *J* = 17.3 Hz, 1H), 5.16 – 5.04 (m, 2H), 4.86 (d, *J* = 3.6 Hz, 1H), 4.82 (dd, *J* = 3.6, 1.7 Hz, 1H), 4.79 – 4.73 (m, 1H), 4.49 (d, *J* = 7.7 Hz, 1H), 4.48 – 4.26 (m, 4H), 4.18 – 4.08 (m, 1H), 3.93 – 3.79 (m, 7H), 3.73 – 3.66 (m, 5H), 3.64 – 3.54 (m, 2H), 3.53 – 3.44 (m, 2H), 3.34 (s, 3H), 2.08 (s, 3H), 2.070 (s, 3H), 2.066 (s, 3H), 1.44 (s, 3H), 1.41 (s, 9H), 1.36 (s, 3H), 0.83 (s, 9H), 0.04 (s, 6H); ^{13}C NMR (100 MHz, $CDCl_3$) δ 170.9, 170.2, 169.9, 167.4, 155.2, 135.1, 116.0, 103.4, 101.0, 99.5, 96.9, 82.7, 80.0, 78.4, 78.1, 74.6, 74.1, 73.2, 72.9, 72.8, 70.9, 70.4, 68.4, 64.9, 62.7, 62.0, 55.6, 55.2, 52.7, 29.0, 28.5 (3C), 25.8 (3C), 21.1, 21.0, 20.9, 19.0, 18.3, -4.1, -4.9; HRMS: m/z (ESI) calcd for $C_{43}H_{72}NO_{21}Si^{+}$, [M + H]$^{+}$, 966.4361, found 966.4385. $^{1}J_{^{13}C1-H1}$ = 162.1 Hz, 174.0 Hz, 174.7 Hz.

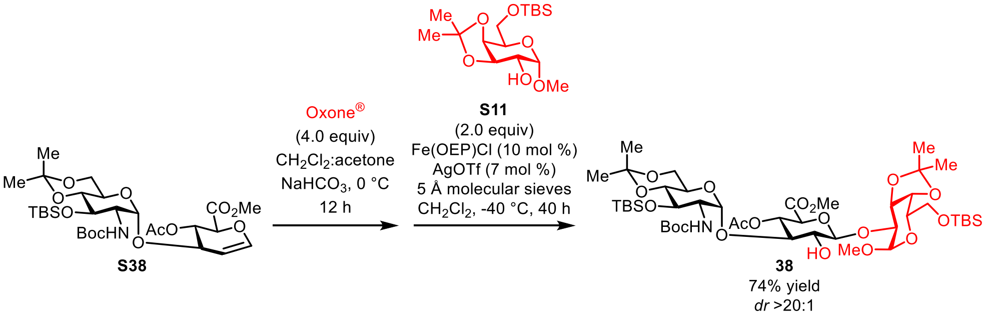

The reaction was carried out on a 0.12 mmol scale using the glycal as the limiting reagent by following **Procedure A** for epoxidation and **Procedure C** for the iron-catalyzed stereospecific glycosylation with the modifications: Oxone® (4.0 equiv) was used for epoxidation and glycosylation was carried out at −40 °C. Catalyst **1d** (7 mol %) and secondary acceptor **S11** (0.24 mmol, 2 equiv) were used. The *dr* of the corresponding glycal α-epoxide is 9:1. The *dr* of the glycosylation product determined based on crude reaction mixture is >20:1. The desired product **38** was purified through a silica gel flash column (hexanes/EtOAc: from 20:1 to 3:1) as white foam (88.4 mg, 74% yield).

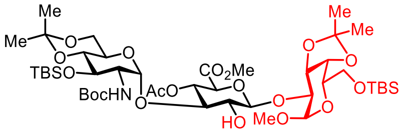

38

Methyl 2-*O*-*tert*-butoxycarbonyl-3-*O*-*tert*-butyldimethylsilyl-4,6-*O*-isopropylidene-α-D-glucopyranosyl-(1→3)-(methyl 4-*O*-acetyl-β-D-glucopyranosyluronate)-(1→2)-6-*O*-*tert*-butyldimethylsilyl-3,4-*O*-isopropylidene-α-D-galactopyranoside (**38**): $[\alpha]_D^{23}$ +58.6 (acetone, *c* = 1.0); IR ν_{max} (neat)/cm^{-1}: 3455 (w), 2930 (w), 1720 (m), 1502 (m), 1368 (m), 1221 (m), 1162 (s), 1130 (s), 1069 (s), 1028 (s), 836 (s), 777 (s); ^{1}H NMR (400 MHz, $CDCl_3$) δ 5.12 (t, *J* = 8.4 Hz, 1H), 4.85 (d, *J* = 3.1 Hz, 1H), 4.78 (d, *J* = 3.3 Hz, 1H), 4.66 (d, *J* = 7.4 Hz, 1H), 4.51 (d, *J* = 9.2 Hz, 1H), 4.43 – 4.32 (m, 1H), 4.25 – 4.16 (m, 1H), 4.01 – 3.93 (m, 2H), 3.93 – 3.72 (m, 6H),

3.69 (s, 3H), 3.68 – 3.51 (m, 5H), 3.49 (t, J = 9.5 Hz, 1H), 3.37 (s, 3H), 2.03 (s, 3H), 1.46 (s, 3H), 1.44 (s, 3H), 1.41 (s, 9H), 1.35 (s, 3H), 1.31 (s, 3H), 0.88 (s, 9H), 0.84 (s, 9H), 0.06 (s, 6H), 0.04 (s, 6H); ^{13}C NMR (100 MHz, $CDCl_3$) δ 169.8, 167.6, 155.4, 109.3, 102.9, 100.7, 99.3, 99.1, 80.1, 79.8, 77.5, 75.4, 74.8, 73.5, 72.6, 72.4, 71.4, 71.1, 67.9, 64.3, 62.3, 62.2, 55.7, 55.5, 52.8, 29.2, 28.6 (3C), 28.4, 26.6, 25.94 (3C), 25.92 (3C), 20.9, 19.1, 18.4, 18.3, -4.0, -4.8, -5.3, -5.4; HRMS: m/z (ESI) calcd for $C_{45}H_{82}NO_{19}Si_2^+$, $[M + H]^+$, 996.5014, found 996.5029. $^{1}J^{13}{}_{C1-H1}$ = 162.9 Hz, 173.5 Hz, 174.8 Hz.

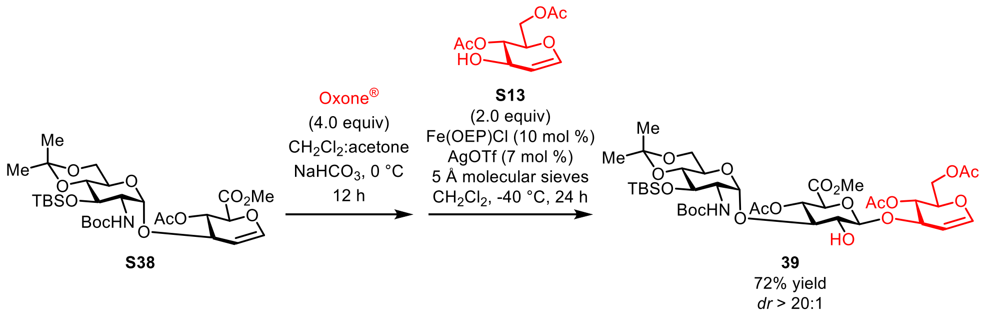

The reaction was carried out on a 0.12 mmol scale using the glycal as the limiting reagent by following **Procedure A** for epoxidation and **Procedure C** for the iron-catalyzed stereospecific glycosylation with the modifications: Oxone® (4.0 equiv) was used for epoxidation and glycosylation was carried out at −40 °C. Catalyst **1d** (7 mol %) and secondary acceptor **S13** (0.24 mmol, 2 equiv) were used. The *dr* of the corresponding glycal α-epoxide is 9:1. The *dr* of the glycosylation product determined based on crude reaction mixture is >20:1. The desired product **39** was purified through a silica gel flash column (hexanes/EtOAc: from 20:1 to 2:1) as white foam (75.9 mg, 72% yield).

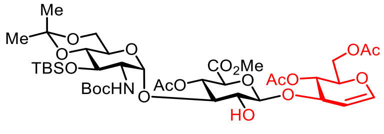

39

4,6-Di-*O*-acetyl-1,5-anhydro-3-*O*-[2-*O*-*tert*-butoxycarbonyl-3-*O*-*tert*-butyldimethylsilyl-4,6-*O*-isopropylidene-α-D-glucopyranosyl-(1→3)-(methyl 4-*O*-acetyl-β-D-glucopyranosyluronate)]-2-deoxy-D-*arabino*-hex-1-enitol (**39**): $[\alpha]_D^{23}$ +22.2 (acetone, *c* = 1.0); IR ν_{max} (neat)/cm^{-1}: 2970 (w), 2359 (w), 1739 (s), 1366 (m), 1228 (s), 1217 (s), 1128 (w), 1028 (m), 875 (m), 837 (m), 778 (w); ^{1}H NMR (400 MHz, $CDCl_3$) δ 6.45 (d, *J* = 6.2 Hz, 1H), 5.27 (t, *J* = 5.5 Hz, 1H), 5.12 (t, *J* = 9.7 Hz, 1H), 4.92 – 4.84 (m, 2H), 4.50 (d, *J* = 10.0 Hz, 1H), 4.46 (d, *J* = 7.6 Hz, 1H), 4.35 (dd, *J* = 12.8, 7.3 Hz, 1H), 4.29 – 4.22 (m, 3H), 3.97 – 3.74 (m, 4H), 3.71 (s, 3H), 3.73 – 3.61 (m, 2H), 3.57 (t, *J* = 9.3 Hz, 1H), 3.52 – 3.45 (m, 2H), 3.42 (brs, 1H), 2.08 (s, 3H), 2.07 (s, 3H), 2.05 (s, 3H), 1.43 (s, 3H), 1.41 (s, 9H), 1.36 (s, 3H), 0.84 (s, 9H), 0.04 (s, 3H), 0.03 (s, 3H); ^{13}C NMR (100 MHz, $CDCl_3$) δ 170.7, 169.9, 169.7, 167.4, 155.3, 145.6, 101.0, 100.7, 99.4, 98.3, 80.7, 79.8, 74.6, 74.0, 72.5, 72.1, 72.0, 71.1, 70.9, 67.8, 64.4, 62.1, 61.6, 55.6, 52.7, 29.0, 28.5 (3C), 25.9 (3C), 20.9, 20.84, 20.81, 19.0, 18.3, -4.1, -4.9; HRMS: m/z (ESI) calcd for $C_{39}H_{64}NO_{19}Si^+$, [M + H]$^+$, 878.3836, found 878.3829. $^1J_{^{13}C1-H1}$ = 160.6 Hz, 170.5 Hz.

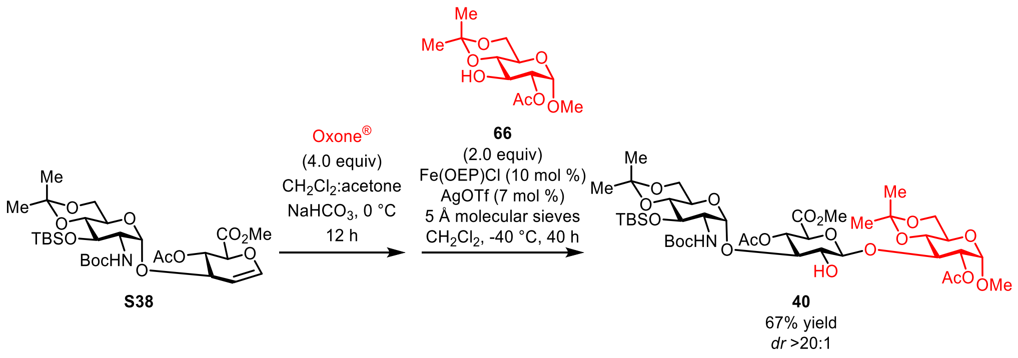

The reaction was carried out on a 0.12 mmol scale using the glycal as the limiting reagent by following **Procedure A** for epoxidation and **Procedure C** for the iron-catalyzed stereospecific glycosylation with the modifications: Oxone® (4.0 equiv) was used for epoxidation and glycosylation was carried out at −40 °C. Catalyst **1d** (7 mol %) and secondary acceptor **66** (0.24 mmol, 2 equiv) were used. The *dr* of the corresponding glycal α-epoxide is 9:1. The *dr* of the glycosylation product determined based on crude reaction mixture is >20:1. The desired product

40 was purified through a silica gel flash column (hexanes/EtOAc: from 20:1 to 2:1) as white foam (74.4 mg, 67% yield).

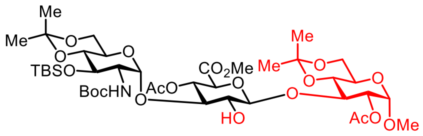

40

Methyl 2-*O*-*tert*-butoxycarbonyl-3-*O*-*tert*-butyldimethylsilyl-4,6-*O*-isopropylidene-α-D-glucopyranosyl-(1→3)-(methyl 4-*O*-acetyl-β-D-glucopyranosyluronate)-(1→3)-2-*O*-acetyl-4,6-*O*-isopropylidene-α-D-glucopyranoside (40): $[\alpha]_D^{23}$ +39.5 (acetone, *c* = 1.0); IR ν_{max} (neat)/cm^{-1}: 3454 (w), 2932 (w), 1747 (m), 1718 (m), 1503 (m), 1370 (m), 1231 (m), 1171 (m), 1079 (s), 1031 (s), 997 (s), 875 (m), 853 (m), 837 (m), 779 (m); ^{1}H NMR (400 MHz, C_6D_6) δ 5.16 (dd, *J* = 9.7, 3.8 Hz, 1H), 5.07 (t, *J* = 9.6 Hz, 1H), 5.00 (d, *J* = 3.8 Hz, 1H), 4.83 (d, *J* = 3.7 Hz, 1H), 4.70 (d, *J* = 10.1 Hz, 1H), 4.39 (t, *J* = 9.5 Hz, 1H), 4.35 – 4.23 (m, 2H), 4.19 (td, *J* = 10.0, 3.9 Hz, 1H), 3.95 (dd, *J* = 10.2, 5.2 Hz, 1H), 3.80 – 3.73 (m, 2H), 3.70 (t, *J* = 9.3 Hz, 1H), 3.67 – 3.50 (m, 6H), 3.47 (t, *J* = 9.4 Hz, 1H), 3.36 (s, 3H), 3.23 (td, *J* = 8.6, 2.8 Hz, 1H), 2.97 (s, 3H), 1.94 (s, 3H), 1.82 (s, 3H), 1.57 (s, 3H), 1.55 (s, 9H), 1.41 (s, 3H), 1.26 (s, 3H), 1.24 (s, 3H), 1.09 (s, 9H), 0.24 (s, 3H), 0.21 (s, 3H); ^{13}C NMR (100 MHz, C_6D_6) δ 170.4, 169.2, 167.6, 155.4, 101.6, 100.4, 99.7, 99.3, 98.5, 79.2, 77.8, 75.1, 73.9, 73.23, 73.19, 72.3, 71.9, 71.7, 70.5, 64.0, 63.7, 62.5, 62.4, 55.9, 54.8, 52.0, 29.4, 29.2, 28.5 (3C), 26.2 (3C), 20.6, 20.5, 19.0, 18.9, 18.7, -3.7, -4.9; HRMS: m/z (ESI) calcd for $C_{41}H_{70}NO_{20}Si^+$, [M + H]$^+$, 924.4255, found 924.4228. $^1J_{^{13}C1–H1}$ = 162.3 Hz, 175.3 Hz, 174.1 Hz.

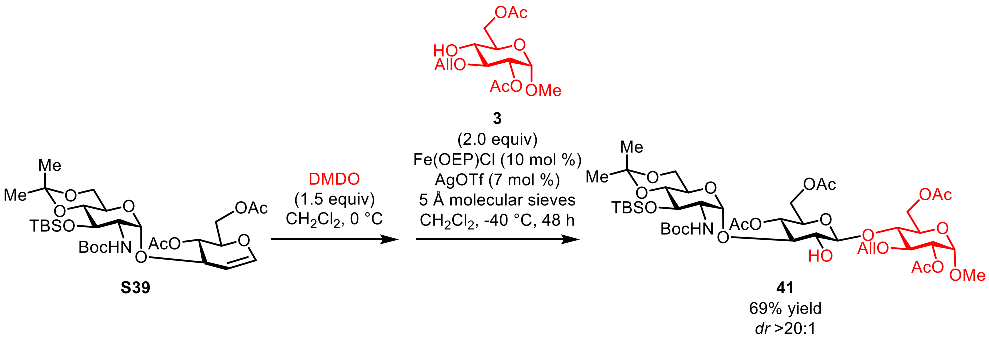

S39 was synthesized according to a literature procedure.[1]

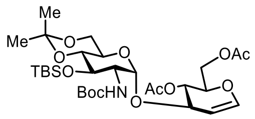

S39

4,6-Di-*O*-acetyl-1,5-anhydro-3-*O*-(2-*tert*-butoxycarbonylamino-3-*O*-*tert*-butyldimethylsilyl-4,6-*O*-isopropylidene-2-deoxy-α-D-glucopyranosyl)-2-deoxy-D-*arabino*-hex-1-enitol (**S39**): $[\alpha]_D^{23}$ +22.5 (acetone, *c* = 1.0); IR ν_{max} (neat)/cm^{-1}: 3423 (w), 2954 (w), 2929 (w), 1747 (m), 1719 (m), 1649 (w), 1505 (m), 1367 (m), 1230 (s), 1170 (s), 1129 (s), 1081 (s), 1033 (s), 1017 (s), 987 (m), 877 (s), 779 (m); ^{1}H NMR (400 MHz, $CDCl_3$) δ 6.37 (d, *J* = 6.2 Hz, 1H), 5.28 – 5.19 (m, 1H), 4.86 (d, *J* = 3.7 Hz, 1H), 4.82 (dd, *J* = 6.2, 2.7 Hz, 1H), 4.60 (d, *J* = 10.2 Hz, 1H), 4.38 (dd, *J* = 12.2, 5.0 Hz, 1H), 4.27 – 4.07 (m, 3H), 3.92 – 3.75 (m, 2H), 3.76 – 3.63 (m, 2H), 3.60 (t, *J* = 9.2 Hz, 1H), 3.50 (t, *J* = 8.4 Hz, 1H), 2.11 (s, 3H), 2.10 (s, 3H), 1.46 (s, 3H), 1.43 (s, 9H), 1.38 (s, 3H), 0.85 (s, 9H), 0.06 (s, 3H), 0.05 (s, 3H); ^{13}C NMR (100 MHz, $CDCl_3$) δ 170.8, 170.0, 155.4, 144.8, 101.8, 101.3, 99.6, 79.8, 75.3, 75.0, 74.3, 71.1, 68.2, 64.4, 62.4, 61.7, 55.7, 29.2, 28.6 (3C), 25.9 (3C), 21.1, 20.9, 19.1, 18.4, -4.1, -4.8; HRMS: m/z (ESI) calcd for $C_{30}H_{52}NO_{12}Si^+$, $[M + H]^+$, 646.3253, found 646.3239. $^1J_{^{13}C1-H1}$ = 173.0 Hz.

The reaction was carried out on a 0.12 mmol scale using the glycal as the limiting reagent by following **Procedure B** for epoxidation and **Procedure C** for the iron-catalyzed stereospecific glycosylation with the modifications: glycosylation was carried out at −40 °C. Catalyst **1d** (7 mol %) and secondary acceptor **3** (0.24 mmol, 2 equiv) were used. The *dr* of the corresponding glycal α-epoxide is 10:1. The *dr* of the glycosylation product determined based on crude reaction mixture is >20:1. The desired product **41** was purified through a silica gel flash column (hexanes/EtOAc: from 20:1 to 3:2) as white foam (81.1 mg, 69% yield).

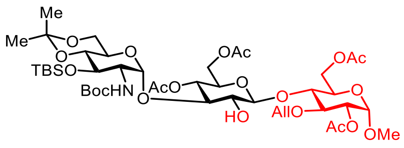

41

Methyl 2-*tert*-butoxycarbonylamino-3-*O*-*tert*-butyldimethylsilyl-4,6-*O*-isopropylidene-2-deoxy-α-D-glucopyranosyl-(1→3)-4,6-di-*O*-acetyl-β-D-glucopyranosyl-(1→4)-2,6-di-*O*-acetyl-3-*O*-allyl-α-D-glucopyranoside (**41**): $[\alpha]_D^{23}$ +69.4 (acetone, *c* = 1.0); IR ν_{max} (neat)/cm^{-1}: 3446 (w), 2929 (w), 2366 (w), 1743 (m), 1503 (w), 1368 (m), 1233 (s), 1171 (m), 1128 (m), 1073 (m), 1031 (s), 873 (m), 861 (m), 837 (m), 778 (m); ^{1}H NMR (400 MHz, $CDCl_3$) δ 5.85 (ddt, *J* = 17.2, 10.2, 5.3 Hz, 1H), 5.21 (d, *J* = 17.2 Hz, 1H), 5.15 – 5.00 (m, 2H), 4.86 – 4.79 (m, 2H), 4.76 (dd, *J* = 9.9, 3.5 Hz, 1H), 4.54 – 4.29 (m, 5H), 4.23 (dd, *J* = 12.6, 5.3 Hz, 1H), 4.13 (dd, *J* = 12.6, 5.3 Hz, 1H), 4.03 (dd, *J* = 12.3, 2.4 Hz, 1H), 3.97 (s, 1H), 3.92 – 3.79 (m, 5H), 3.77 – 3.67 (m, 2H), 3.63 – 3.48 (m, 4H), 3.43 (t, *J* = 8.0 Hz, 1H), 3.34 (s, 3H), 2.09 (s, 3H), 2.08 (s, 3H), 2.06 (s, 3H), 2.04 (s, 3H), 1.43 (s, 3H), 1.41 (s, 9H), 1.36 (s, 3H), 0.84 (s, 9H), 0.040 (s, 3H), 0.036 (s, 3H); ^{13}C NMR (100 MHz, $CDCl_3$) δ 170.8 (two peaks overlapped, 2C), 170.3, 167.0, 155.3, 135.1, 116.1, 103.4, 101.1, 99.5, 96.9, 84.6, 80.1, 78.08, 78.06, 74.7, 74.1, 73.5, 73.1, 71.8, 70.7, 68.8, 68.5, 65.0, 62.7, 62.1, 62.0, 55.7, 55.2, 29.1, 28.5 (3C), 25.9 (3C), 21.1 (two peaks overlapped, 2C), 21.0, 20.8, 19.0, 18.3, -4.1, -4.8; HRMS: m/z (ESI) calcd for $C_{44}H_{74}NO_{21}Si^+$, $[M + H]^+$, 980.4517, found 980.4533. ${}^1J_{^{13}C1\text{–}H1}$ = 162.1 Hz, 175.0 Hz, 174.7 Hz.

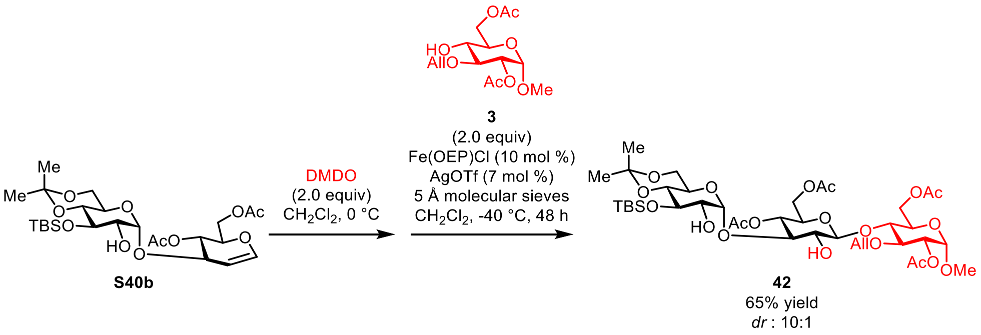

S40b was synthesized according to the following procedure.

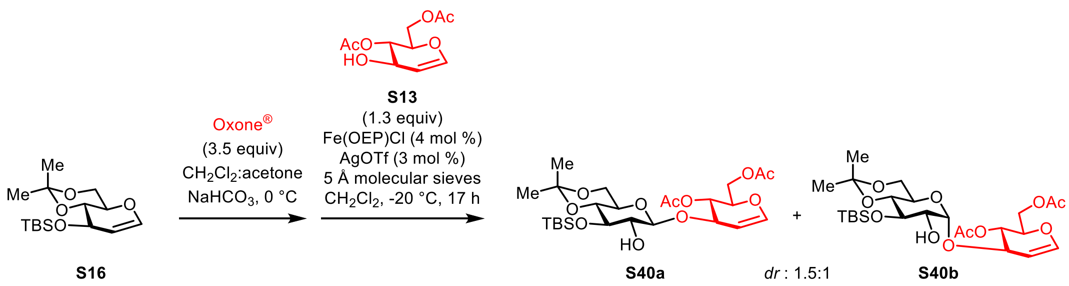

To a 500 mL flask equipped with a stir bar at 0 °C were added glycal **S16** (901.4 mg, 3.0 mmol, 1.0 equiv) in CH_2Cl_2/acetone mixture (v/v: 5:1, 50 mL) and saturated aqueous $NaHCO_3$ solution (85 mL). Oxone® ($KHSO_5 \cdot 0.5KHSO_4 \cdot 0.5K_2SO_4$) (6.46 g, 10.5 mmol, 3.5 equiv) in H_2O (55 mL) was then added to the mixture dropwise. After stirring vigorously at 0 °C for 2 h, the reaction mixture was extracted with CH_2Cl_2 (75 mL × 3). The combined organic phase was dried over anhydrous Na_2SO_4 and concentrated *in vacuo*. The residue was further azeotropically dried with anhydrous toluene (25 mL). The obtained glycal epoxide was directly used in the next step.

To a 100 mL flame-dried round bottom flask equipped with a stir bar were added glycosyl acceptor **S13** (0.90 g, 3.9 mmol, 1.3 equiv), Fe(OEP)Cl (74.5 mg, 0.12 mmol, 4 mol %), AgOTf (23.1 mg, 0.09 mmol, 3 mol %), and freshly activated 5 Å molecular sieves, powder (*ca.* 1.0 g). After the vial was evacuated and backfilled with N_2 three times, anhydrous CH_2Cl_2 (15 mL) was added, and the solution was stirred at -78 °C for 20 min. The glycal epoxide **S36** (0.95 g, 3.0 mmol, 1.0 equiv) was dissolved in anhydrous CH_2Cl_2 (5 mL) and added to the reaction mixture at -78 °C dropwise. The reaction mixture was kept at -20 °C for 16 h, and then quenched with MeOH (1.0 mL) and imidazole (180 mg in 20 mL CH_2Cl_2) at the same temperature. The mixture was filtered through a pad of Celite® and eluted with EtOAc (30 mL × 2). The organic layer was then concentrated *in vacuo* and the residue was purified through column chromatography (hexanes/acetone: from 100:1 to 5:1) to afford the desired product **S40a** (708 mg, 43% yield) and **S40b** (472 mg, 29% yield) as white foam.

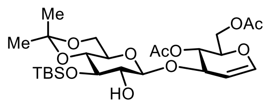

S40a

4,6-Di-*O*-acetyl-1,5-anhydro-3-*O*-(3-*O*-*tert*-butyldimethylsilyl-4,6-*O*-isopropylidene-β-D-glucopyranosyl)-2-deoxy-D-*arabino*-hex-1-enitol (**S40a**): $[\alpha]_D^{23}$ −46.5 (acetone, *c* = 1.0); IR ν_{max} (neat)/cm^{-1}: 3506 (w), 2954 (w), 2929 (w), 2857 (w), 1746 (s), 1650 (w), 1370 (m), 1231 (s), 1172 (m), 1091 (s), 1061 (s), 1034 (s), 854 (s), 838 (s), 780 (m); ^{1}H NMR (400 MHz, CDCl3) δ 6.45 (dd, J = 6.2, 1.1 Hz, 1H), 5.24 (t, J = 4.8 Hz, 1H), 4.93 (dd, *J* = 6.2, 3.9 Hz, 1H), 4.46 (d, J = 7.8 Hz, 1H), 4.38 – 4.19 (m, 4H), 3.88 (dd, J = 10.7, 5.3 Hz, 1H), 3.71 (t, J = 10.5 Hz, 1H), 3.54 (t, J = 8.7 Hz, 1H), 3.44 (t, J = 9.2 Hz, 1H), 3.32 (td, J = 8.1, 2.3 Hz, 1H), 3.20 (td, J = 9.9, 5.3 Hz, 1H), 2.64 (d, J = 3.1 Hz, 1H), 2.08 (s, 3H), 2.07 (s, 3H), 1.45 (s, 3H), 1.38 (s, 3H), 0.88 (s, 9H), 0.09 (s, 3H), 0.07 (s, 3H); ^{13}C NMR (100 MHz, $CDCl_3$) δ 170.9, 169.8, 145.3, 101.6, 99.5, 98.5, 75.3, 75.1, 73.9, 73.6, 70.9, 68.2, 67.5, 62.4, 61.8, 29.1, 25.9 (3C), 21.1, 21.0, 19.1, 18.5, -4.3, -4.7; HRMS: m/z (ESI) calcd for $C_{25}H_{43}O_{11}Si^+$, $[M + H]^+$, 547.2569, found 547.2581. $^{1}J_{^{13}C1\text{–}H1}$ = 157.7 Hz.

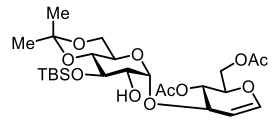

S40b

4,6-Di-*O*-acetyl-1,5-anhydro-3-*O*-(3-*O*-*tert*-butyldimethylsilyl-4,6-*O*-isopropylidene-α-D-glucopyranosyl)-2-deoxy-D-*arabino*-hex-1-enitol (**S40b**): $[\alpha]_D^{23}$ +21.8 (acetone, *c* = 1.0); IR ν_{max} (neat)/cm^{-1}: 3538 (w), 2929 (w), 2856 (w), 1745 (m), 1649 (w), 1370 (m), 1228 (s), 1131 (m), 1059 (s), 1036 (s), 863 (m), 837 (m), 780 (m); ^{1}H NMR (400 MHz, $CDCl_3$) δ 6.41 (d, *J* = 6.1 Hz, 1H), 5.23 (dd, *J* = 7.6, 5.8 Hz, 1H), 4.97 (d, *J* = 4.0 Hz, 1H), 4.83 (dd, *J* = 6.2, 3.1 Hz, 1H), 4.45 – 4.35 (m, 1H), 4.27 – 4.13 (m, 3H), 3.85 – 3.75 (m, 1H), 3.74 – 3.58 (m, 3H), 3.50 – 3.34 (m, 2H), 2.09 (s, 6H), 2.01 (d, *J* = 9.7 Hz, 1H), 1.44 (s, 3H), 1.38 (s, 3H), 0.88 (s, 9H), 0.09 (s, 3H), 0.07 (s, 3H); ^{13}C NMR (100 MHz, $CDCl_3$) δ 170.7, 170.5, 144.9, 101.2, 101.0, 99.5, 74.14, 74.05, 73.99, 73.6, 73.3, 68.3, 64.3, 62.5, 61.5, 29.2, 25.9 (3C), 21.1, 20.9, 19.1, 18.5, -4.4, -4.6; HRMS: m/z (ESI) calcd for $C_{25}H_{43}O_{11}Si^+$, $[M + H]^+$, 547.2569, found 547.2566. $^{1}J_{^{13}C1\text{–}H1}$ = 173.2 Hz.

The reaction was carried out on a 0.12 mmol scale using the glycal as the limiting reagent by following **Procedure B** for epoxidation and **Procedure C** for the iron-catalyzed stereospecific

glycosylation with the modifications: glycosylation was carried out at −40 °C. Catalyst **1d** (7 mol %) and secondary acceptor **S13** (0.24 mmol, 2 equiv) were used. The *dr* of the corresponding glycal α-epoxide is 10:1. The *dr* of the glycosylation product determined based on crude reaction mixture is 10:1. The desired product **42** was purified through a silica gel flash column (hexanes/EtOAc: from 20:1 to 3:2) as white foam (68.6 mg, 65% yield).

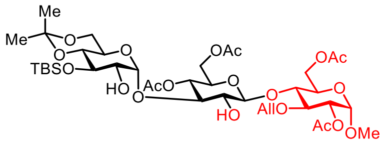

42

Methyl 3-*O*-*tert*-butyldimethylsilyl-4,6-*O*-isopropylidene-α-D-glucopyranosyl-(1→3)-4,6-di-*O*-acetyl-β-D-glucopyranosyl-(1→4)-2,6-di-*O*-acetyl-3-*O*-allyl-α-D-glucopyranoside (42): $[\alpha]_D^{23}$ +81.8 (acetone, *c* = 1.0); IR ν_{max} (neat)/cm^{-1}: 3467 (w), 2930 (w), 2856 (w), 2359 (w), 1742 (m), 1370 (m), 1234 (m), 1130 (m), 1033 (s), 944 (m), 862 (m), 837 (m), 779 (m); ^{1}H NMR (400 MHz, $CDCl_3$) δ 5.86 (ddt, *J* = 17.3, 10.5, 5.3 Hz, 1H), 5.22 (d, *J* = 17.3 Hz, 1H), 5.15 – 5.05 (m, 2H), 4.88 (d, *J* = 4.1 Hz, 1H), 4.84 (d, *J* = 3.5 Hz, 1H), 4.78 (dd, *J* = 9.8, 3.5 Hz, 1H), 4.56 – 4.44 (m, 2H), 4.43 – 4.30 (m, 2H), 4.25 (dd, *J* = 12.4, 5.3 Hz, 1H), 4.15 (dd, *J* = 12.4, 5.3 Hz, 1H), 4.05 (d, *J* = 12.2 Hz, 1H), 3.90 – 3.70 (m, 6H), 3.68 – 3.59 (m, 2H), 3.59 – 3.37 (m, 5H), 3.36 (s, 3H), 2.11 (s, 3H), 2.09 (s, 3H), 2.06 (s, 3H), 2.05 (s, 3H), 1.86 (d, *J* = 8.7 Hz, 1H), 1.44 (s, 3H), 1.37 (s, 3H), 0.87 (s, 9H), 0.09 (s, 3H), 0.07 (s, 3H); ^{13}C NMR (100 MHz, $CDCl_3$) δ 170.9, 170.8, 170.6, 170.3, 135.1, 116.3, 103.4, 101.6, 99.5, 97.0, 85.1, 78.02, 77.95, 74.1, 73.8, 73.5, 73.3, 73.2, 73.0, 71.9, 68.6 (two peaks overlapped, 2C), 64.8, 62.8, 62.1 (two peaks overlapped, 2C), 55.2, 29.1, 25.9 (3C), 21.1, 21.03, 21.01, 20.9, 19.1, 18.5, -4.3, -4.6; HRMS: m/z (ESI) calcd for $C_{39}H_{65}O_{20}Si^+$, [M + H]$^+$, 881.3833, found 881.3826. $^1J_{^{13}C1\text{–}H1}$ = 162.1 Hz, 172.9 Hz, 174.6 Hz.

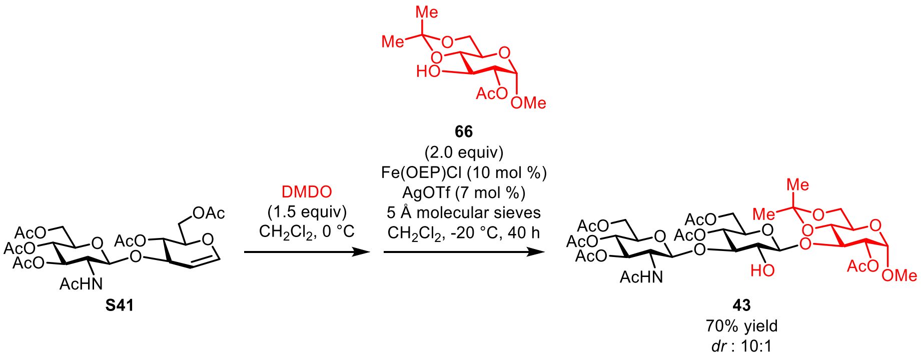

S41 was synthesized according to a literature procedure.[19]

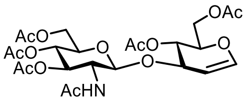

S41

4,6-Di-*O*-acetyl-3-*O*-(2-acetamido-3,4,6-tri-*O*-acetyl-2-deoxy-β-D-glucopyranosyl)-1,5-anhydro-2-deoxy-D-*arabino*-hex-1-enitol (S41): $[\alpha]_D^{23}$ −33.5 (acetone, *c* = 1.0); IR ν_{max} (neat)/cm^{-1}: 3284 (w), 2929 (w), 1739 (s), 1650 (m), 1367 (m), 1220 (s), 1037 (s), 902 (w); ^{1}H NMR (400 MHz, $CDCl_3$) δ 6.43 (d, *J* = 6.4 Hz, 1H), 5.84 (d, *J* = 8.2 Hz, 1H), 5.47 (t, *J* = 8.2 Hz, 1H), 5.19 (t, *J* = 4.1 Hz, 1H), 5.06 (d, *J* = 8.4 Hz, 1H), 5.01 (t, *J* = 9.7 Hz, 1H), 4.89 (dd, *J* = 6.3, 5.2 Hz, 1H), 4.48 – 4.31 (m, 2H), 4.23 (dd, *J* = 12.3, 4.8 Hz, 1H), 4.18 (t, *J* = 4.0 Hz, 1H), 4.15 – 4.05 (m, 2H), 3.74 (ddd, *J* = 10.2, 4.8, 2.4 Hz, 1H), 3.54 (dt, *J* = 10.8, 8.3 Hz, 1H), 2.08 (s, 3H), 2.062 (s, 3H), 2.060 (s, 3H), 2.004 (s, 3H), 1.998 (s, 3H), 1.90 (s, 3H); ^{13}C NMR (100 MHz, $CDCl_3$) δ 171.0, 170.8, 170.7, 170.6, 169.8, 169.6, 145.3, 97.6, 97.2, 73.7, 72.0, 71.9, 69.0, 68.9, 67.6, 62.2, 61.7, 55.8, 23.3, 20.99, 20.95, 20.83, 20.79, 20.75; HRMS: m/z (ESI) calcd for $C_{24}H_{34}NO_{14}^+$, $[M + H]^+$, 560.1974, found 560.1977. $^1J_{^{13}C1\text{–}H1}$ = 162.8 Hz.

The reaction was carried out on a 0.12 mmol scale using the glycal as the limiting reagent by following the **Procedure B** for epoxidation and **Procedure C** for the iron-catalyzed stereospecific glycosylation. Catalyst **1c** (7 mol %) and secondary acceptor **66** (0.24 mmol, 2 equiv) were used. The *dr* of the corresponding glycal α-epoxide is >20:1. The *dr* of the glycosylation product determined based on crude reaction mixture is 10:1. The desired product

43 was purified through a silica gel flash column (hexanes/acetone: from 20:1 to 3:2) as white foam (71.8 mg, 70% yield).

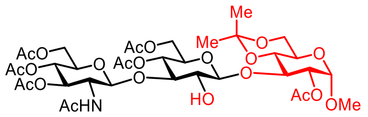

43

Methyl 2-acetamido-3,4,6-tri-*O*-acetyl-2-deoxy-β-D-glucopyranosyl-(1→3)-4,6-di-*O*-acetyl-β-D-glucopyranosyl-(1→3)-2-*O*-acetyl-4,6-*O*-isopropylidene-α-D-glucopyranoside (**43**): $[\alpha]_D^{23}$ +38.4 (acetone, *c* = 1.0); IR ν_{max} (neat)/cm^{-1}: 3329 (w), 2925 (w), 1740 (ms), 1649 (w), 1367 (m), 1223 (s), 1036 (s), 1036 (s), 901 (w); ^{1}H NMR (400 MHz, acetone-d_6) δ 6.96 (d, *J* = 8.6 Hz, 1H), 5.25 (dd, *J* = 10.5, 9.4 Hz, 1H), 5.14 (d, *J* = 8.4 Hz, 1H), 4.96 (t, *J* = 9.7 Hz, 1H), 4.89 – 4.75 (m, 3H), 4.65 (d, *J* = 7.8 Hz, 1H), 4.37 – 4.26 (m, 2H), 4.17 (dd, *J* = 12.1, 5.0 Hz, 1H), 4.09 – 3.98 (m, 3H), 3.88 – 3.69 (m, 7H), 3.58 (td, *J* = 9.9, 5.6 Hz, 1H), 3.41 (d, *J* = 8.1 Hz, 1H), 3.36 (s, 3H), 2.06 (s, 3H), 2.04 (s, 3H), 2.03 (s, 3H), 2.00 (s, 3H), 1.96 (s, 3H), 1.92 (s, 3H), 1.84 (s, 3H), 1.50 (s, 3H), 1.34 (s, 3H); ^{13}C NMR (100 MHz, acetone-d_6) δ 170.8 (two peaks overlapped, 2C), 170.7 (two peaks overlapped, 2C), 170.5, 170.2, 170.0, 102.4, 102.2, 100.3, 98.7, 82.9, 76.1, 74.8, 73.8, 73.5, 72.9, 72.5, 72.3, 69.8, 69.4, 64.5, 63.4, 63.0, 62.8, 55.7, 55.4, 29.5, 23.2, 21.0, 20.9, 20.72, 20.68, 20.6 (two peaks overlapped, 2C), 19.3; HRMS: m/z (ESI) calcd for $C_{36}H_{54}NO_{22}^+$, [M + H]$^+$, 852.3132, found 852.3110. $^1J_{^{13}C1-H1}$ = 159.7 Hz, 163.0 Hz, 173.7 Hz.

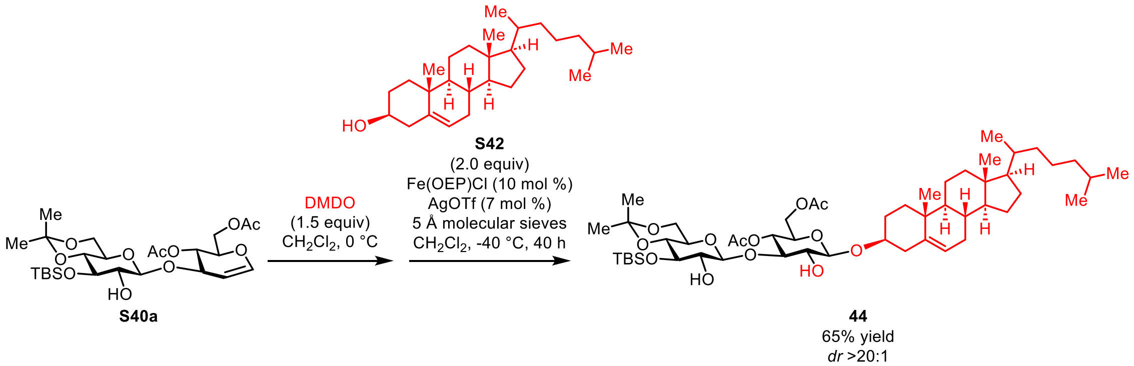

The reaction was carried out on a 0.12 mmol scale using the glycal as the limiting reagent by following **Procedure B** for epoxidation and **Procedure C** for the iron-catalyzed stereospecific glycosylation with the modifications: glycosylation was carried out at −40 °C. Catalyst **1d** (7 mol %) and secondary acceptor **S42** (0.24 mmol, 2 equiv) were used. The *dr* of the corresponding glycal α-epoxide is >20:1. The *dr* of the glycosylation product determined based on crude reaction mixture is >20:1. The desired product **44** was purified through a silica gel flash column (hexanes/EtOAc: from 20:1 to 6:1) as white foam (74.1 mg, 65% yield).

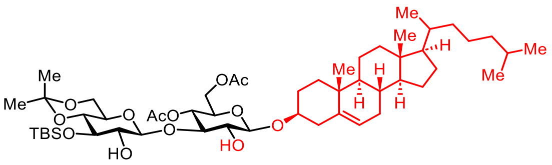

44

Cholesteryl 3-*O*-*tert*-butyldimethylsilyl-4,6-*O*-isopropylidene-β-D-glucopyranosyl-(1→3)-4,6-di-*O*-acetyl-β-D-glucopyranoside (**44**): $[\alpha]_D^{23}$ –17.1 (acetone, *c* = 1.0); IR ν_{max} (neat)/cm^{-1}: 2926 (m), 2851 (m), 2360 (w), 1746 (m), 1368 (m), 1231 (s), 1034 (s), 853 (s), 837 (s), 780 (m); ^{1}H NMR (400 MHz, $CDCl_3$) δ 5.35 (d, *J* = 5.1 Hz, 1H), 4.98 (t, *J* = 9.5 Hz, 1H), 4.45 (d, *J* = 7.6 Hz, 1H), 4.42 (d, *J* = 7.6 Hz, 1H), 4.24 (dd, *J* = 12.2, 5.3 Hz, 1H), 4.07 (dd, *J* = 12.1, 2.4 Hz, 1H), 3.83 (dd, *J* = 10.6, 5.3 Hz, 1H), 3.71 (t, *J* = 10.4 Hz, 1H), 3.65 (t, *J* = 9.2 Hz, 1H), 3.63 – 3.48 (m, 4H), 3.48 – 3.38 (m, 2H), 3.30 – 2.94 (brs, 1H), 3.18 (td, *J* = 9.8, 5.3 Hz, 1H), 2.40 – 2.21 (m, 2H), 2.05 (s, 3H), 2.00 (s, 3H), 2.00 – 1.75 (m, 6H), 1.72 – 1.29 (m, 13H), 1.44 (s, 3H), 1.38 (s, 3H), 1.20– 0.96 (m, 7H), 1.02– 0.97 (m, 1H), 1.00 (s, 3H), 0.90 (d, *J* = 6.4 Hz, 3H), 0.870 (s, 9H), 0.865 (d, *J* = 1.8 Hz, 3H), 0.85 (d, *J* = 1.8 Hz, 3H), 0.67 (s, 3H), 0.07 (s, 3H), 0.06 (s, 3H); ^{13}C NMR (100 MHz, $CDCl_3$) δ 170.9, 169.5, 140.5, 122.3, 106.2, 100.9, 99.5, 86.1, 79.7, 76.3, 75.2, 73.7, 73.2, 72.0, 68.8, 67.8, 62.6, 62.3, 56.9, 56.3, 50.3, 42.5, 39.9, 39.7, 39.0, 37.4, 36.9, 36.3, 35.9, 32.1 32.0, 29.7, 29.1, 28.4, 28.1, 25.9 (3C), 24.4, 24.0, 23.0, 22.7, 21.2, 21.0, 20.9, 19.5, 19.1, 18.9, 18.4, 12.0, -4.2, -4.7; HRMS: m/z (ESI) calcd for $C_{52}H_{89}O_{13}Si^+$, $[M + H]^+$, 949.6067, found 949.6083. $^1J_{^{13}C1\text{–}H1}$ = 159.3 Hz, 158.6 Hz.

D. Synthetic Applications of the Iron-Catalyzed Stereospecific Glycosylation with Glycal Epoxides

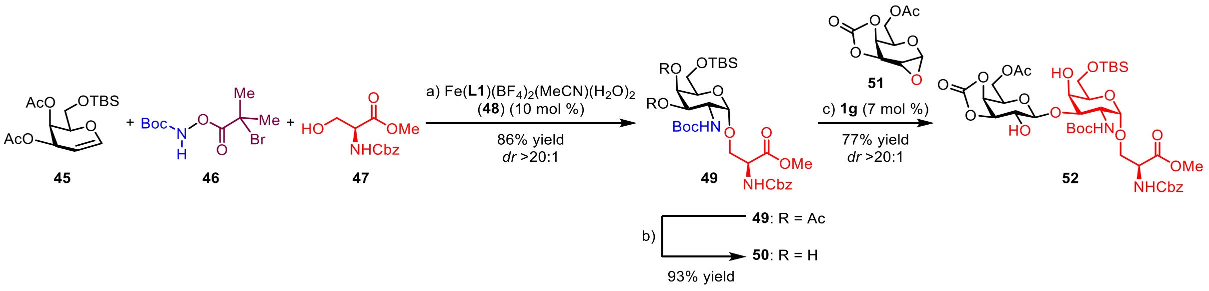

Figure S4. Synthetic Application in the Mucin-Type *O*-Linked Glycopeptide Core 1 Structure Assembly.

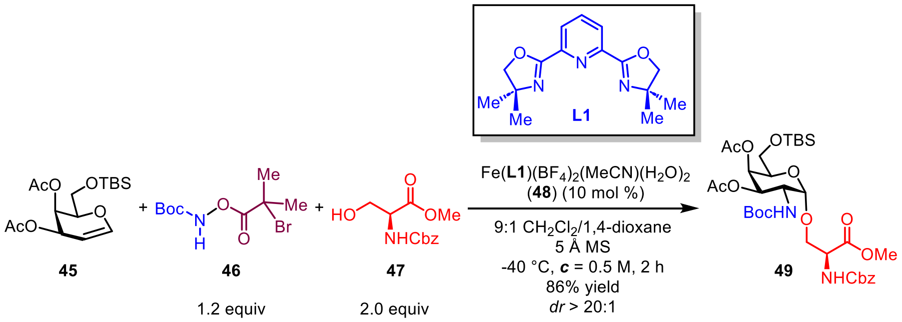

To a flame-dried 250 mL round bottom flask (flask **A**) equipped with a stir bar were added glycal **45** (6.89 g, 20 mmol, 1 equiv), serine methyl ester **47** (7.09 g, 28 mmol, 1.4 equiv), iron catalyst **48** Fe(**L1**)$(BF_4)_2(MeCN)(H_2O)_2$ (1.16 g, 2 mmol, 10 mol %), and freshly activated 5 Å powdered molecular sieves (*ca.* 8 g). After the flask was evacuated and backfilled with N_2 twice, anhydrous CH_2Cl_2 (16 mL) and freshly distilled 1,4-dioxane (4 mL) were added and the flask was cooled to -78 °C. To a flame-dried 50 mL round bottom flask (flask **B**) was added acyloxyl carbamate **46** (8.46 g, 30 mmol, 1.5 equiv). Flask **B** was evacuated and backfilled with N_2 twice and then anhydrous CH_2Cl_2 (20 mL) was added. The solution in flask **B** was added to flask **A** via a syringe in 10 min. The reaction was kept at -78 °C for an additional 3 min before switched to -40 °C. The reaction was kept at -40 °C for 2 h and quenched by precipitating the iron catalyst with Et_2O (80 mL) at the same temperature. The mixture was stirred for 2 min and subsequently

warmed to room temperature. The solution was then filtered through a short pad of Celite® and washed with saturated aqueous $NaHCO_3$ solution (30 mL). The organic phase was separated from the aqueous one, which was further extracted with CH_2Cl_2 (30 mL × 3). The combined organic phase was dried over anhydrous Na_2SO_4 and concentrated *in vacuo*. The residue was purified through a silica gel flash column (hexanes/EtOAc: from 20:1 to 2:1) to afford the desired product **49** as white foam (12.26 g, 86% yield).

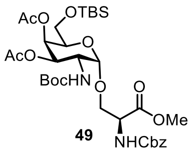

***N*-Benzyloxycarbonyl-*O*-(3,4-di-*O*-acetyl-2-*tert*-butoxycarbonylamino-6-*O*-*tert*-butyldimethylsilyl-2-deoxy-α-D-galactopyranosyl)-L-serine methyl ester** (**49**): $[\alpha]_D^{23}$ +52.1 (CH_2Cl_2, c = 1.0); IR ν_{max} (neat)/cm^{-1}: 3349 (w), 2954 (w), 2856 (w), 2362 (w), 1749 (s), 1720 (s), 1521 (m), 1456 (w), 1367 (m), 1242 (s), 1219 (s), 1171 (m), 1111 (m), 1067 (m), 1043(m), 839 (m), 778 (w); ^1H NMR (400 MHz, $CDCl_3$) δ 7.41 – 7.25 (m, 5H), 5.67 (d, J = 8.6 Hz, 1H), 5.39 (d, J = 3.7 Hz, 1H), 5.13 (ABq, $\Delta\nu_{AB}$ = 17.3 Hz, J_{AB} = 11.8 Hz, 2H), 4.98 (dd, J = 11.3, 3.2 Hz, 1H), 4.80 (d, J = 3.8 Hz, 1H), 4.56 (d, J = 9.6 Hz, 2H), 4.17 (td, J = 10.8, 3.7 Hz, 1H), 3.98 – 3.82 (m, 3H), 3.76 (s, 3H), 3.65 – 3.48 (m, 2H), 2.10 (s, 3H), 1.95 (s, 3H), 1.39 (s, 9H), 0.83 (s, 9H), −0.01 (s, 3H), −0.02 (s, 3H); ^{13}C NMR (100 MHz, $CDCl_3$) δ 170.5, 170.4, 170.0, 155.8, 155.2, 136.0, 128.5 (2C), 128.2, 128.1 (2C), 99.4, 79.8, 69.9, 69.6, 69.2, 67.2 (two peaks overlapped, 2C), 60.9, 54.2, 52.7, 48.9, 28.2 (3C), 24.9 (3C), 20.72, 20.66, 18.1, -5.6, -5.7; HRMS: m/z (ESI) calcd for $C_{33}H_{53}N_2O_{13}Si^+$, $[M + H]^+$, 713.3311, found 713.3292. $^1J_{^{13}C1–H1}$ = 175.4 Hz.

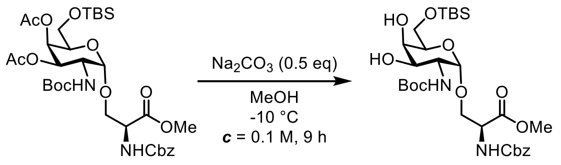

49 50

To a 50 mL over-dried flask equipped with a stir bar were added compound **49** (1.18 g, 1.66 mmol, 1.0 equiv) and MeOH (16.6 mL). The solution was cooled to -10 °C, and anhydrous Na_2CO_3 (powder, 88 mg, 0.83 mmol, 0.5 equiv) was then added. The reaction mixture was stirred at -10 °C for 9 h, with progress monitored by TLC. AcOH (95 µL, 1.66 mmol, 1.0 equiv) was added to quench the reaction and the solution was concentrated *in vacuo*. The residue was purified through column chromatography (hexanes/EtOAc: from 20:1 to 2:1) to afford the desired product **50** as white foam (971 mg, 93% yield).

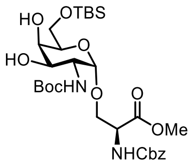

50

***N*-Benzyloxycarbonyl-*O*-(2-*tert*-butoxycarbonylamino-6-*O*-*tert*-butyldimethylsilyl-2-deoxy-α-D-galactopyranosyl)-L-serine methyl ester** (**50**): $[\alpha]_D^{23}$ +66.9 (acetone, *c* = 1.0); IR ν_{max} (neat)/cm^{-1}: 3441 (w), 2970 (m), 2360 (m), 1738 (s), 1521 (m), 1366 (s), 1217 (s), 1092 (m), 837 (m), 697 (w); ^{1}H NMR (400 MHz, acetone-d$_6$) δ 7.44 – 7.27 (m, 5H), 6.85 (d, *J* = 9.0 Hz, 1H), 5.38 (d, *J* = 9.4 Hz, 1H), 5.10 (ABq, $\Delta\nu_{AB}$ = 1.9 Hz, J_{AB} = 12.2 Hz, 2H), 4.77 (d, *J* = 3.7 Hz, 1H), 4.57 – 4.50 (m, 1H), 4.01 – 3.93 (m, 2H), 3.92 – 3.90 (m, 1H), 3.90 – 3.87 (m, 1H), 3.87 – 3.84 (m, 1H), 3.84 – 3.82 (m, 1H), 3.81 – 3.64 (m, 7H), 1.40 (s, 9H), 0.90 (s, 9H), 0.08 (s, 6H); ^{13}C NMR (100 MHz, acetone-d$_6$) δ 171.5, 157.1, 157.0, 138.0, 129.2 (2C), 128.8, 128.7 (2C), 100.0, 79.1, 72.3, 69.9, 69.5, 69.0, 67.0, 63.3, 55.3, 52.7, 52.4, 28.6 (3C), 26.3 (3C), 18.82, -5.13, -5.24; HRMS: m/z (ESI) calcd for $C_{29}H_{49}N_2O_{11}Si^+$, $[M + H]^+$, 629.3100, found 629.3079.

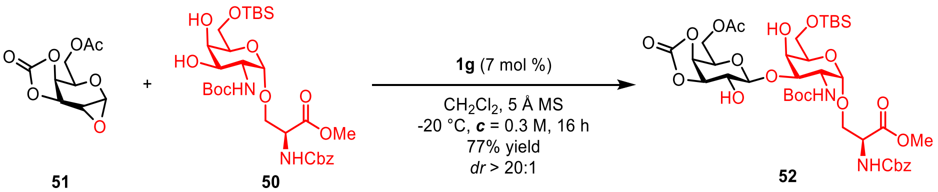

51 **50** **52**

To a flame-dried sealable 2-dram vial equipped with a stir bar were added glycal epoxide **51** (27.6 mg, 0.12 mmol, 1.0 equiv) and freshly activated 5 Å molecular sieves, powder (*ca.* 100 mg). After the vial was evacuated and backfilled with N_2 three times, glycosyl acceptor **50** (150.9 mg, 0.24 mmol, 2.0 equiv) was dissolved in anhydrous CH_2Cl_2 (0.3 mL) and transferred to the vial dropwise. After the mixture was stirred at -20 °C for 5 min, iron catalyst **1g** (6.7 mg, 0.0084 mmol, 7 mol %) in anhydrous CH_2Cl_2 (0.1 mL) was added at -20 °C dropwise. The reaction mixture was kept at -20 °C for 16 h and then quenched with MeOH (50 μL) and imidazole (8.5 mg in 1 mL CH_2Cl_2) at the same temperature. The mixture was filtered through a piece of cotton and eluted with EtOAc (2 mL × 2). The organic layer was then concentrated *in vacuo*. The residue was purified through column chromatography (hexanes/ethyl acetate: from 20:1 to 1:1) to afford the desired product **52** as white foam (79.4 mg, 77% yield).

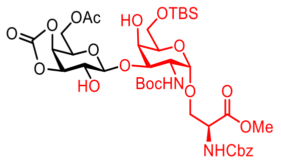

52

***N*-Benzyloxycarbonyl-*O*-[(6-*O*-acetyl-3,4-*O*-isopropylidene-β-D-galactopyranosyl)-(1→3)-(2-*tert*-butoxycarbonylamino-6-*O*-*tert*-butyldimethylsilyl-2-deoxy-α-D-galactopyranosyl)]-L-serine methyl ester** (**52**): $[\alpha]_D^{25}$ +39.2 (CH_2Cl_2, c = 1.0); IR ν_{max} (neat)/cm^{-1}: 3367 (w), 2930 (w), 2360 (w), 1801 (m), 1704 (s), 1519 (m), 1367 (m), 1229 (s), 1168 (s), 1134 (s), 1069 (s), 1032 (s), 980 (m), 837 (m), 777 (m); ^{1}H NMR (400 MHz, acetone-d_6) δ 7.41 – 7.29 (m, 5H), 6.77 (d, J = 9.1 Hz, 1H), 5.48 (d, J = 9.7 Hz, 1H), 5.13 (d, J = 12.2 Hz, 1H), 5.10 – 5.03 (m, 2H), 4.90 – 4.81 (m, 2H), 4.78 (d, J = 3.7 Hz, 1H), 4.60 (brs, 1H), 4.58 – 4.51 (m, 1H), 4.34 – 4.25 (m, 2H), 4.22 – 4.08 (m, 3H), 4.01 – 3.94 (m, 2H), 3.92 – 3.82 (m, 3H), 3.81 – 3.67 (m, 5H), 3.43 (brs, 1H), 2.03 (s, 3H), 1.40 (s, 9H), 0.90 (s, 9H), 0.09 (s, 6H); ^{13}C NMR (100 MHz, acetone-d_6) δ 171.6, 170.6, 157.0, 156.5, 155.4, 137.9, 129.3 (2C), 128.9, 128.8 (2C), 102.6, 100.2, 79.3, 78.8, 78.1, 75.1, 72.3, 70.7, 69.3, 69.0, 68.9, 67.1, 63.2, 63.0, 55.3, 52.7, 50.9, 28.6 (3C), 26.3 (3C), 20.7, 18.8, -5.1, -5.2; HRMS: m/z (ESI) calcd for $C_{38}H_{59}N_2O_{18}Si^+$, [M + H]$^+$, 859.3527, found 859.3554. $^1J_{^{13}C1\text{–}H1}$ = 166.3 Hz, 172.6 Hz.

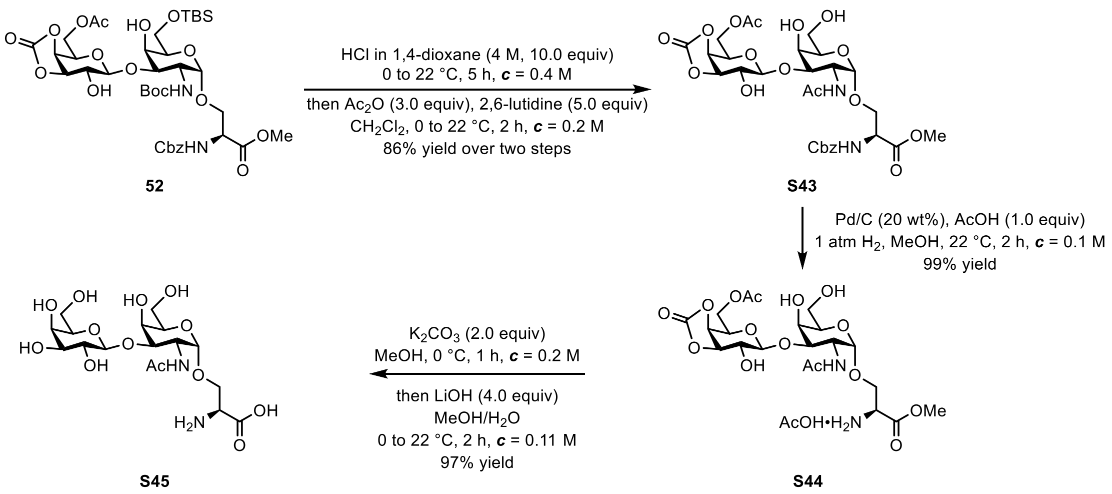

Figure S5. Post-Glycosylation Deprotection to Afford the Core 1 Structure.

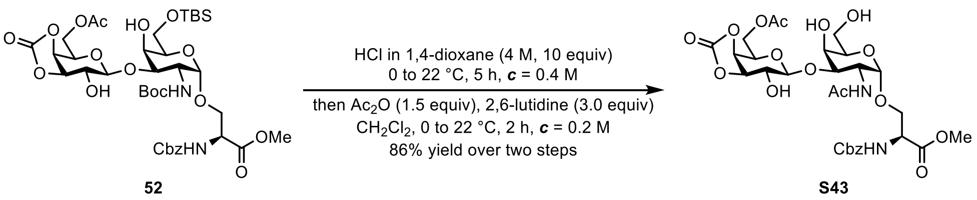

To a sealable 2-dram vial equipped with a stir bar was added compound **52** (86 mg, 0.1 mmol, 1.0 equiv). The vial was cooled to 0 °C, followed by addition of HCl (4 M solution in 1,4-dioxane, 0.25 mL, 1 mmol, 10 equiv). The reaction mixture was stirred for 10 min at 0 °C before being warmed to room temperature. The reaction mixture was stirred for another 5 h with the progress monitored by TLC until completion. The reaction mixture was concentrated *in vacuo* to afford the desired product as white foam which was directly used in the next step without further purification.

To a solution of the crude product (0.1 mmol, 1.0 equiv) obtained from the previous step in anhydrous CH_2Cl_2 (0.5 mL) was added 2,6-lutidine (35 μL, 0.3 mmol, 5.0 equiv) at 0 °C. After the mixture was stirred for 5 min at 0 °C, Ac_2O (15 μL, 0.15 mmol, 3.0 equiv) was added dropwise. The reaction mixture was slowly warmed to room temperature and stirred for another 2 h until the starting material was fully consumed (monitored by TLC). The reaction was then

diluted with CH_2Cl_2 (1 mL) and quenched with saturated aqueous NH_4Cl solution (1 mL). After the aqueous phase was extracted by CH_2Cl_2 (1.5 mL × 3), the combined organic layer was washed with brine, dried over anhydrous Na_2SO_4, and concentrated *in vacuo*. The desired product **S43** was obtained through column chromatography (hexanes/acetone: from 20:1 to 1:3) as white foam (59 mg, 86% yield over two steps).

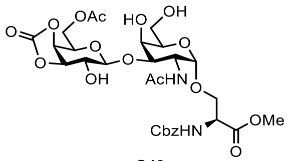

S43

***N*-Benzyloxycarbonyl-*O*-[(6-*O*-acetyl-3,4-*O*-isopropylidene-β-D-galactopyranosyl)-(1→3)-(2-acetamido-2-deoxy-α-D-galactopyranosyl)]-L-serine methyl ester** (**S43**): $[\alpha]_D^{23}$ +46.5 (acetone, c = 0.7); IR ν_{max} (neat)/cm^{-1}: 3351 (w), 2926 (w), 1802 (m), 1740 (m), 1712 (m), 1531 (m), 1369 (m), 1226 (s), 1168 (s), 1063 (s), 1029 (s), 775 (m); ^{1}H NMR (400 MHz, acetone-d$_6$) δ 7.29 – 7.16 (m, 5H), 4.97 (ABq, $\Delta\nu_{AB}$ = 1.1 Hz, J_{AB} = 12.2 Hz, 2H), 4.89 (dd, J = 7.5, 1.8 Hz, 1H), 4.73 – 4.61 (m, 3H), 4.38 (t, J = 4.0 Hz, 1H), 4.31 (dd, J = 10.9, 3.8 Hz, 1H), 4.27 – 4.12 (m, 2H), 4.07 (dd, J = 10.3, 4.3 Hz, 1H), 4.01 (d, J = 3.1 Hz, 1H), 3.88 – 3.76 (m, 3H), 3.72 (t, J = 6.1 Hz, 1H), 3.66 – 3.56 (m, 5H), 3.53 (t, J = 5.9 Hz, 1H), 1.91 (s, 3H), 1.74 (s, 3H); ^{13}C NMR (100 MHz, acetone-d$_6$) δ 171.7, 170.8 (two peaks overlapped, 2C), 157.0, 155.2, 137.9, 129.3 (2C), 128.9 (2C), 128.8, 103.0, 100.0, 79.1 (two peaks overlapped, 2C), 75.4, 72.2, 71.3, 69.8, 69.2, 68.9, 67.1, 63.1, 62.1, 55.4, 52.7, 49.2, 23.2, 20.7; HRMS: m/z (ESI) calcd for $C_{29}H_{39}N_2O_{17}^+$, $[M + H]^+$, 687.2243, found 687.2261. $^1J_{^{13}C1-H1}$ = 171.9 Hz, 164.0 Hz.

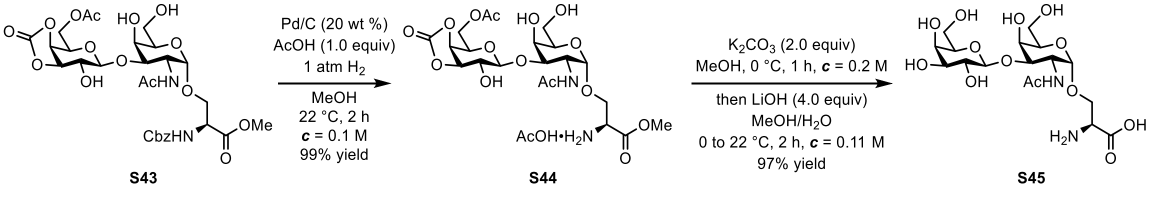

To a sealable 2-dram vial equipped with a stir bar was added compound **S43** (50 mg, 0.073 mmol, 1.0 equiv). After the vial was evacuated and backfilled with N_2 three times, Pd/C (10 mg, 20 wt %) was added. The vial was once again evacuated and backfilled with N_2 three times

before MeOH (0.73 mL) and AcOH (4.2 μL, 0.073 mmol, 1.0 equiv) were added sequentially. The vial was then evacuated and backfilled with H_2 three times. The solution was stirred for 2 h at room temperature, with progress monitored by TLC until completion. The reaction mixture was then filtered through a short pad of Celite® (washed with MeOH), and the filtrate was concentrated *in vacuo* to afford the desired product **S44** (44 mg, 99% yield) as a white solid, which was used directly in the next step without further purification.

To a flame-dried sealable 2-dram vial equipped with a stir bar were added compound **S44** from the previous step and anhydrous MeOH (0.36 mL). The solution was cooled to 0 °C, and anhydrous K_2CO_3 (powder, 20 mg, 0.146 mmol, 2.0 equiv) was then added. The reaction mixture was stirred for 1 h at 0 °C, with progress monitored by TLC until completion. LiOH (1 M aqueous solution, 0.29 mL, 0.29 mmol, 4.0 equiv) was then added at 0 °C. The reaction mixture was stirred at 0 °C for 10 min and then warmed to room temperature. The reaction mixture was stirred for another 2 h until the starting material was fully consumed (monitored by TLC). The reaction mixture was then diluted with MeOH and neutralized with Amberlite® IRC 120 H until pH = 7. The reaction mixture was then filtered through a piece of cotton (washed with MeOH), and the filtrate was concentrated *in vacuo* to afford the desired product **S45** (33 mg, 97% yield) as a white solid.

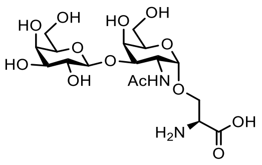

S45

***O*-[β-D-Galactopyranosyl-(1→3)-(2-acetamido-2-deoxy-α-D-galactopyranosyl)]-L-serine** (**S45**): ^{1}H NMR (400 MHz, CD_3OD) δ 4.84 (d, *J* = 3.9 Hz, 1H), 4.48 (dd, *J* = 11.1, 3.7 Hz, 1H), 4.38 (d, *J* = 7.5 Hz, 1H), 4.22 – 4.12 (m, 1H), 4.07 (dd, *J* = 11.0, 2.9 Hz, 1H), 3.91 (m, 3H), 3.85 – 3.78 (m, 3H), 3.78 – 3.65 (m, 4H), 3.57 – 3.47 (m, 2H), 3.44 (dd, *J* = 9.8, 3.2 Hz, 1H), 2.00 (s, 3H); ^{13}C NMR (100 MHz, CD_3OD) δ 172.9, 170.4, 105.2, 98.9, 77.3, 75.4, 73.3, 71.4, 71.0, 68.9, 68.8, 67.2, 61.6, 61.2, 54.7, 48.5, 21.7; HRMS: m/z (ESI) calcd for $C_{17}H_{31}N_2O_{13}^+$, $[M + H]^+$, 471.1821, found 471.1814. $^1J_{^{13}C1-H1}$ = 171.0 Hz, 158.6 Hz.

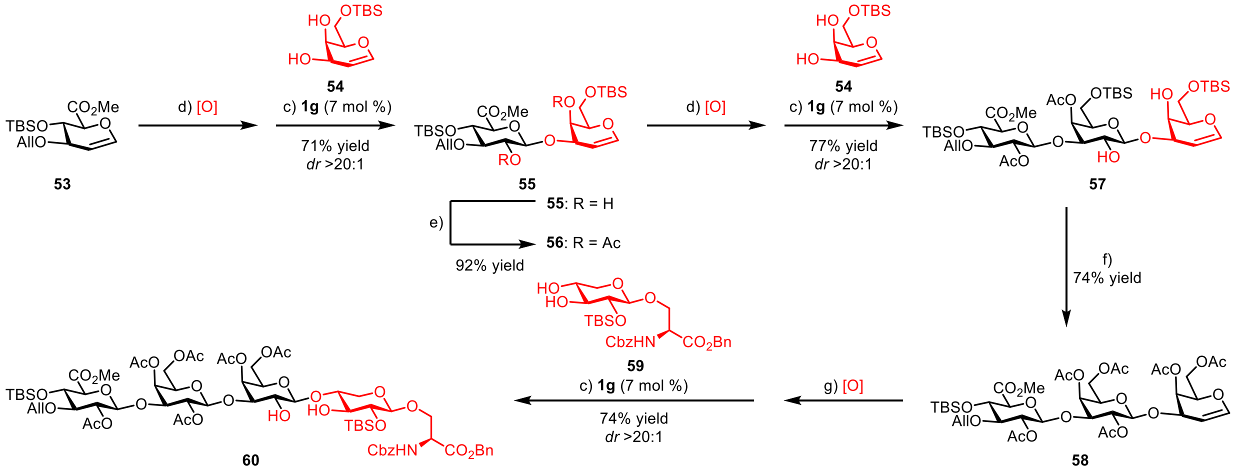

Figure S6. Synthetic Application in the Assembly of Proteoglycan Tetrasaccharide Linker.

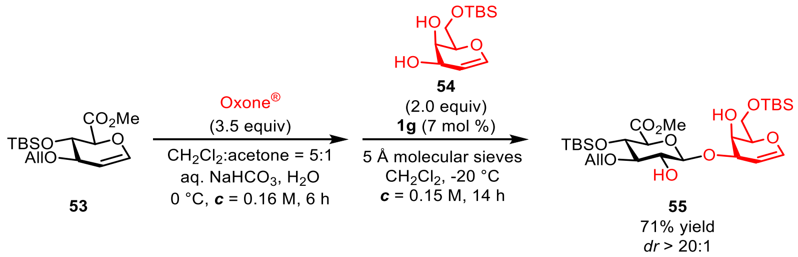

To a 100 mL flask equipped with a stir bar at 0 °C were added glycal **53** (657 mg, 2.0 mmol, 1.0 equiv) in CH_2Cl_2/acetone mixture (v/v: 5:1, 19.2 mL) and saturated aqueous $NaHCO_3$ solution (29 mL), followed by addition of Oxone® ($KHSO_5 \cdot 0.5KHSO_4 \cdot 0.5K_2SO_4$) (4.3 g, 7 mmol, 3.5 equiv) in H_2O (19 mL) dropwise. After stirring vigorously at 0 °C for 6 h with progress monitored by ^1H NMR, the reaction mixture was extracted with CH_2Cl_2 (20 mL × 3). The combined organic phase was dried over anhydrous Na_2SO_4, concentrated *in vacuo*. The residue was further dried azeotropically with anhydrous toluene (10 mL × 3) and directly used in the next step. The diastereomeric ratio of the obtained glycal epoxide was determined by ^1H NMR as 13:1.

To a flame-dried sealable 50 mL flask (flask A) equipped with a stir bar were added the glycosyl acceptor **54** (1.04 g, 4.0 mmol, 2.0 equiv), iron catalyst **1g** (111.1 mg, 0.14 mmol, 7 mol %), and freshly activated 5 Å molecular sieves, powder (*ca.* 2 g). After the flask was evacuated and

backfilled with N_2 three times, anhydrous CH_2Cl_2 (8.0 mL) was added, and the solution was stirred at -40 °C for 10 min. The glycal epoxide from **53** was dissolved in anhydrous CH_2Cl_2 (5.3 mL) and transferred to flask A at -40 °C dropwise. The reaction mixture was stirred at -20 °C for 14 h and quenched with a solution of imidazole (143 mg) in CH_2Cl_2 (14 mL) at the same temperature. The mixture was filtered through a pad of Celite® and eluted with EtOAc. The organic layer was then concentrated *in vacuo*, and the residue was purified through column chromatography (hexanes/EtOAc: from 20:1 to 7:1) to afford the corresponding glycosylation product **55** as a white solid (855 mg, 71% yield, *dr* >20:1, m.p. 114–116 °C).

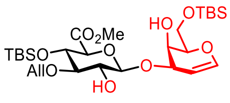

55

1,5-Anhydro-6-*O*-*tert*-butyldimethylsilyl-2-deoxy-3-*O*-(methyl 3-*O*-allyl-4-*O*-*tert*-butyldimethylsilyl-β-D-glucopyranosyluronate)-D-*lyxo*-hex-1-enitol (**55**): $[\alpha]_D^{22}$ −27.1 (acetone, *c* = 1.0); IR ν_{max} (neat)/cm^{-1}: 3455 (w), 2953 (m), 2929 (m), 2886 (m), 2857 (m), 2360 (w), 2342 (w), 1752 (s), 1651 (w), 1472 (w), 1463 (w), 1438 (w), 1365 (m), 1251 (m), 1230(m), 1217 (m), 1140 (m), 1074 (s), 1042 (m), 838 (s), 779 (m); ^{1}H NMR (400 MHz, $CDCl_3$) δ 6.42 (dd, *J* = 6.3, 1.8 Hz, 1H), 5.96 (ddt, *J* = 17.3, 10.8, 5.7 Hz, 1H), 5.27 (dd, *J* = 17.3, 1.8 Hz, 1H), 5.17 (dd, *J* = 10.8, 1.8 Hz, 1H), 4.68 (dt, *J* = 6.3, 2.1 Hz, 1H), 4.54 (d, *J* = 7.7 Hz, 1H), 4.47 (dt, *J* = 4.2, 2.0 Hz, 1H), 4.41 (dd, *J* = 12.3, 5.7 Hz, 1H), 4.21 (dd, *J* = 12.3, 5.7 Hz, 1H), 4.10 (s, 1H), 3.95 (dd, *J* = 9.4, 6.5 Hz, 1H), 3.92 – 3.78 (m, 4H), 3.75 (s, 3H), 3.58 (td, *J* = 8.4, 2.6 Hz, 1H), 3.29 (dt, *J* = 8.6, 4.0 Hz, 1H), 3.02 (d, *J* = 2.7 Hz, 1H), 2.67 (d, *J* = 2.8 Hz, 1H), 0.89 (s, 9H), 0.85 (s, 9H), 0.10 (s, 3H), 0.08 (s, 3H), 0.07 (s, 3H), -0.00 (s, 3H); ^{13}C NMR (100 MHz, $CDCl_3$) δ 168.2, 145.8, 135.0, 116.9, 101.9, 98.7, 83.1, 76.6, 76.5, 74.3, 73.9, 73.4, 71.7, 64.0, 61.9, 52.4, 25.9 (3C), 25.7 (3C), 18.3, 17.9, -4.0, -5.3, -5.4, -5.5; HRMS: m/z (ESI) calcd for $C_{28}H_{53}O_{10}Si_2^+$, $[M + H]^+$, 605.3172, found 605.3148. $^1J_{^{13}C1\text{–}H1}$ = 155.2 Hz.

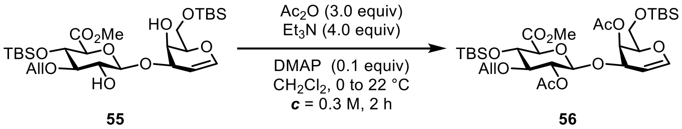

55 **56**

To a solution of **55** (855 mg, 1.4 mmol, 1.0 equiv) and DMAP (17 mg, 0.14 mmol, 0.1 equiv) in anhydrous CH_2Cl_2 (4.7 mL) was added Et_3N (0.78 mL, 5.6 mmol, 4.0 equiv) at 0 °C. After stirring at 0 °C for 10 min, Ac_2O (0.40 mL, 4.2 mmol, 3.0 equiv) was added dropwise. The reaction mixture was slowly warmed to room temperature and stirred for another 2 h, with progress monitored by TLC until completion. The reaction was then quenched with saturated aqueous $NaHCO_3$ solution (5 mL). The organic phase was separated from the aqueous one, which was then extracted with EtOAc (5 mL × 3). The combined organic layer was washed with brine, dried over anhydrous Na_2SO_4, and then concentrated *in vacuo*. The desired product **56** was obtained through column chromatography (hexanes/EtOAc: from 20:1 to 8:1) as a white solid (890 mg, 92% yield, m.p. 61.7–63.8 °C).

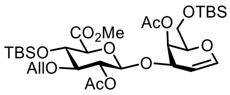

56

4-*O*-Acetyl-1,5-anhydro-6-*O*-*tert*-butyldimethylsilyl-2-deoxy-3-*O*-(methyl 2-*O*-acetyl-3-*O*-allyl-4-*O*-*tert*-butyldimethylsilyl-β-D-glucopyranosyluronate)-D-*lyxo*-hex-1-enitol (**56**): $[\alpha]_D^{22}$ −24.8 (acetone, *c* = 1.0); IR ν_{max} (neat)/cm^{-1}: 2954 (m), 2929 (m), 2887 (w), 2857 (m), 2360 (w), 1752 (s), 1646 (w), 1472 (w), 1463 (w), 1438 (w), 1373 (m), 1232 (s), 1143 (m), 1073 (s), 1044 (m), 839 (s), 780 (m); ^1H NMR (400 MHz, $CDCl_3$) δ 6.38 (dd, *J* = 6.2, 1.6 Hz, 1H), 5.82 (ddt, *J* = 17.3, 10.7, 5.5 Hz, 1H), 5.37 – 5.30 (m, 1H), 5.20 (dd, *J* = 17.3, 1.8 Hz, 1H), 5.12 (dd, *J* = 10.7, 1.8 Hz, 1H), 4.95 (dd, *J* = 8.6, 7.5 Hz, 1H), 4.71 (ddd, *J* = 6.4, 2.7, 1.4 Hz, 1H), 4.56 – 4.46 (m, 2H), 4.14 (dd, *J* = 12.4, 5.5 Hz, 1H), 4.09 – 4.01 (m, 2H), 3.95 (t, *J* = 8.9 Hz, 1H), 3.80 (d, *J* = 9.3 Hz, 1H), 3.73 (s, 3H), 3.73 – 3.64 (m, 2H), 3.35 (t, *J* = 8.6 Hz, 1H), 2.06 (s, 3H), 2.05 (s, 3H), 0.88 (s, 9H), 0.83 (s, 9H), 0.08 (s, 3H), 0.048 (s, 3H), 0.045 (s, 3H), -0.01 (s, 3H); ^{13}C NMR (100 MHz, $CDCl_3$) δ 170.1, 169.0, 168.6, 145.0, 134.4, 116.9, 99.0 (two peaks overlapped, 2C), 82.0, 76.8, 76.6, 73.3, 72.7, 71.6, 68.3, 65.1, 61.6, 52.3, 25.9 (3C), 25.7 (3C), 20.9, 20.7, 18.3, 17.9, -4.1, -5.3, -5.4, -5.5; HRMS: m/z (ESI) calcd for $C_{32}H_{57}O_{12}Si_2^+$, $[M + H]^+$, 689.3383, found 689.3399.

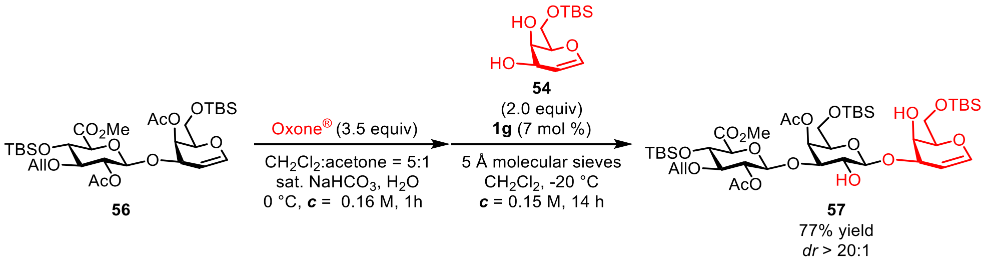

To a 50 mL flask equipped with a stir bar at 0 °C were added glycal **56** (689 mg, 1.0 mmol, 1.0 equiv) in CH_2Cl_2/acetone mixture (v/v: 5:1, 9.6 mL) and saturated aqueous $NaHCO_3$ solution (15 mL), followed by addition of Oxone® ($KHSO_5 \cdot 0.5KHSO_4 \cdot 0.5K_2SO_4$) (2.1 g, 3.5 mmol, 3.5 equiv) in H_2O (10 mL) dropwise. After stirring vigorously at 0 °C for 2 h, the reaction mixture was extracted with CH_2Cl_2 (20 mL × 3). The combined organic phase was dried over anhydrous Na_2SO_4, concentrated *in vacuo*. The residue was further dried azeotropically with anhydrous toluene (10 mL × 3) and directly used in the next step. The diastereomeric ratio of the obtained glycal epoxide was determined by ^{1}H NMR as >20:1.

To a flame-dried sealable 25 mL flask (flask A) equipped with a stir bar were added the glycosyl acceptor **54** (521 mg, 2.0 mmol, 2.0 equiv), iron catalyst **1g** (55.6 mg, 0.07 mmol, 7 mol %), and freshly activated 5 Å molecular sieves, powder (*ca.* 500 mg). After the flask was evacuated and backfilled with N_2 three times, anhydrous CH_2Cl_2 (4.0 mL) was added, and the solution was stirred at -40 °C for 10 min. The aforementioned glycal epoxide from **56** was dissolved in anhydrous CH_2Cl_2 (2.7 mL) and transferred to flask A at -40 °C dropwise. The reaction mixture was kept at -20 °C for 14 h and then quenched with a solution of imidazole (72 mg) in CH_2Cl_2 (7 mL) at the same temperature. The mixture was filtered through a pad of Celite® and eluted with EtOAc. The organic layer was then concentrated *in vacuo*, and the residue was purified through column chromatography (CH_2Cl_2/acetone: from 100:1 to 20:1) to afford the corresponding glycosylation product **57** as white foam (742 mg, 77% yield, *dr* >20:1).

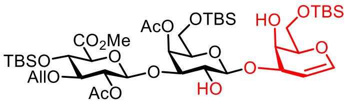

57

1,5-Anhydro-6-*O*-*tert*-butyldimethylsilyl-2-deoxy-3-*O*-[(methyl 2-*O*-acetyl-3-*O*-allyl-4-*O*-*tert*-butyldimethylsilyl-β-D-glucopyranosyluronate)-(1→3)-(4-*O*-acetyl-6-*O*-*tert*-

butoxycarbonyl-β-D-galactopyranosyl)]-D-*lyxo*-hex-1-enitol (**57**): $[\alpha]_D^{22}$ −10.0 (acetone, *c* = 1.0); IR ν_{max} (neat)/cm^{-1}: 3465 (w), 2970 (w), 2929 (w), 2857 (w), 2360 (w), 1740 (s), 1652 (w), 1437 (w), 1366 (m), 1229 (s), 1217 (s), 1091 (m), 838 (m), 779 (w); ^{1}H NMR (400 MHz, $CDCl_3$) δ 6.45 (dd, *J* = 6.3, 1.8 Hz, 1H), 5.83 (ddt, *J* = 17.2, 10.8, 5.6 Hz, 1H), 5.30 (d, *J* = 3.0 Hz, 1H), 5.21 (dd, *J* = 17.2, 1.8 Hz, 1H), 5.13 (dd, *J* = 10.8, 1.8 Hz, 1H), 4.96 (t, *J* = 7.8 Hz, 1H), 4.82 – 4.73 (m, 2H), 4.59 – 4.56 (m, 1H), 4.56 – 4.50 (m, 1H), 4.19 – 4.04 (m, 3H), 3.97 – 3.82 (m, 5H), 3.80 – 3.72 (m, 5H), 3.64 – 3.52 (m, 3H), 3.43 (d, *J* = 2.5 Hz, 1H), 3.36 (t, *J* = 8.3 Hz, 1H), 3.07 (s, 1H), 2.08 (s, 3H), 2.04 (s, 3H), 0.90 (s, 9H), 0.87 (s, 9H), 0.84 (s, 9H), 0.12 – 0.05 (m, 9H), 0.022 (s, 3H), 0.018 (s, 3H), -0.01 (s, 3H); ^{13}C NMR (100 MHz, $CDCl_3$) δ 169.7, 169.7, 168.4, 146.0, 134.3, 115.8, 101.0, 99.7, 98.8, 82.0, 77.7, 76.9, 76.4, 74.7, 73.5, 73.2, 72.4, 71.5, 71.3, 68.2, 64.3, 62.4, 61.6, 52.3, 25.9 (3C), 25.8 (3C), 25.7 (3C), 21.0, 20.6, 18.4, 18.2, 17.8, -4.1, -5.3, -5.35, -5.37, -5.56, -5.58; HRMS: m/z (ESI) calcd for $C_{44}H_{81}O_{17}Si_3^+$, $[M + H]^+$, 965.4776, found 965.4768. $^1J_{^{13}C1–H1}$ = 162.8, 162.8Hz.

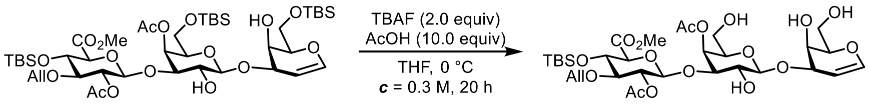

57 **S46**

To a 100 mL round bottom flask equipped with a stir bar were added **57** (483 mg, 0.50 mmol, 1.0 equiv) and THF (0.4 mL). The flask was cooled to 0 °C, and a premixed solution of TBAF (1.0 mL, 1 mmol, 2.0 equiv) and AcOH (0.29 mL, 5 mmol, 10.0 equiv) was added. The reaction mixture was stirred for 20 h at 0 °C, with progress monitored by TLC until completion. The reaction mixture was diluted with EtOAc (5 mL) and then quenched with saturated aqueous NH_4Cl solution (10 mL). The organic phase was separated from the aqueous phase, which was extracted with EtOAc (10 mL × 4). The combined organic phase was washed with brine (15 mL) and dried over Na_2SO_4. After concentration *in vacuo*, the crude product **S46** was used in the next step without further purification.

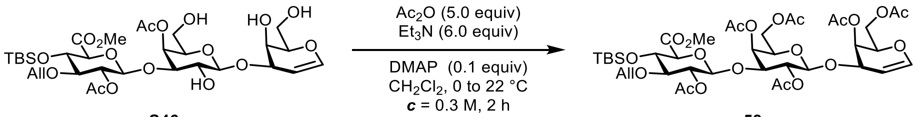

To a solution of **S46** obtained from the previous step and DMAP (6.1 mg, 0.05 mmol, 0.1 equiv) in anhydrous CH_2Cl_2 (3.3 mL) was added Et_3N (0.42 mL, 3.0 mmol, 6.0 equiv) at 0 °C. After stirring at 0 °C for 10 min, Ac_2O (0.24 mL, 2.5 mmol, 5.0 equiv) was added dropwise. The reaction mixture was slowly warmed to room temperature and stirred for another 2 h, then quenched with saturated aqueous $NaHCO_3$ solution (5 mL). The organic phase was separated from the aqueous one, which was extracted by EtOAc (5 mL × 3). The combined organic layer was washed with brine, dried over anhydrous Na_2SO_4, and then concentrated *in vacuo.* The desired product **58** was obtained through column chromatography (hexanes/acetone: from 100:1 to 3:1) as white foam (670 mg, 74% yield over two steps).

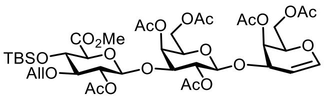

4,6-Di-*O*-*acetyl*-1,5-anhydro-2-deoxy-3-*O*-[(methyl 2-*O*-acetyl-3-*O*-allyl-4-*O*-*tert*-butyldimethylsilyl-β-D-glucopyranosyluronate)-(1→3)-(2,4,6-tri-*O*-acetyl-β-D-galactopyranosyl)]-D-*lyxo*-hex-1-enitol (**58**): $[\alpha]_D^{22}$ −3.8 (acetone, *c* = 1.0); IR ν_{max} (neat)/cm^{-1}: 2970(w), 2360 (w), 1743 (s), 1646 (w), 1437 (w), 1370 (s), 1227 (s), 1217 (s), 1142 (w), 1071 (m), 1044 (m), 839 (w), 780 (w); ^{1}H NMR (400 MHz, $CDCl_3$) δ 6.37 (d, *J* = 6.2 Hz, 1H), 5.80 (ddt, *J* = 15.8, 10.5, 5.4 Hz, 1H), 5.35 (d, *J* = 3.5 Hz, 1H), 5.29 (dd, *J* = 4.9, 2.3 Hz, 1H), 5.23 – 5.07 (m, 3H), 4.85 (t, *J* = 8.0 Hz, 1H), 4.79 (dd, *J* = 6.4, 3.2 Hz, 1H), 4.54 (d, *J* = 7.0 Hz, 1H), 4.53 – 4.46 (m, 1H), 4.43 (d, *J* = 8.1 Hz, 1H), 4.33 – 4.15 (m, 3H), 4.15 – 4.06 (m, 2H), 4.07 – 3.97 (m, 2H), 3.94 (t, *J* = 8.9 Hz, 1H), 3.83 (dd, *J* = 10.1, 3.4 Hz, 1H), 3.80 – 3.73 (m, 5H), 3.29 (t, *J* = 8.5 Hz, 1H), 2.11 (s, 3H), 2.09 (s, 6H), 2.08 (s, 3H), 2.07 (s, 3H), 2.03 (s, 3H), 0.83 (s, 9H), 0.07 (s, 3H), -0.01 (s, 3H); ^{13}C NMR (100 MHz, $CDCl_3$) δ 170.6, 170.6, 170.1, 170.0, 169.0, 168.9, 168.4, 144.7, 134.3, 116.0, 100.6, 98.95, 98.94, 81.9, 76.6, 75.0, 73.3, 73.2, 72.6, 71.6, 71.4, 70.6, 68.8, 67.1, 65.7, 62.2, 62.1, 52.4, 25.7 (3C), 20.9, 20.8, 20.8, 20.8, 20.7, 20.6, 17.9, -4.1, -5.4; HRMS: m/z (ESI) calcd for $C_{40}H_{61}O_{21}Si^+$, $[M + H]^+$, 905.3469, found 905.3439.

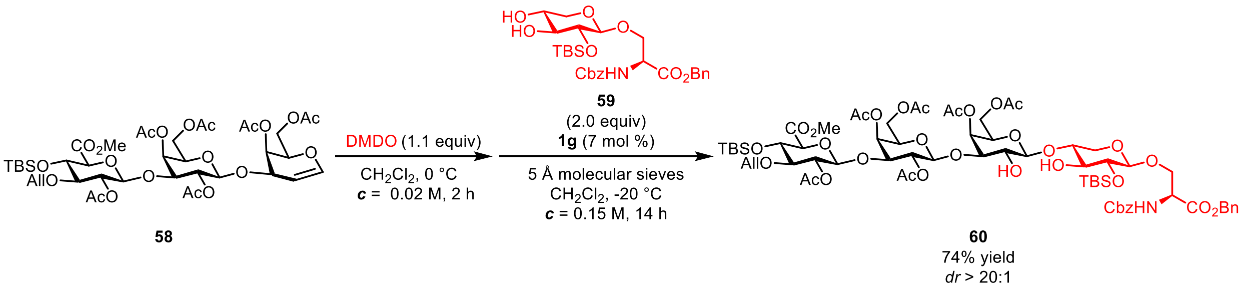

59 was synthesized according to the following procedure.

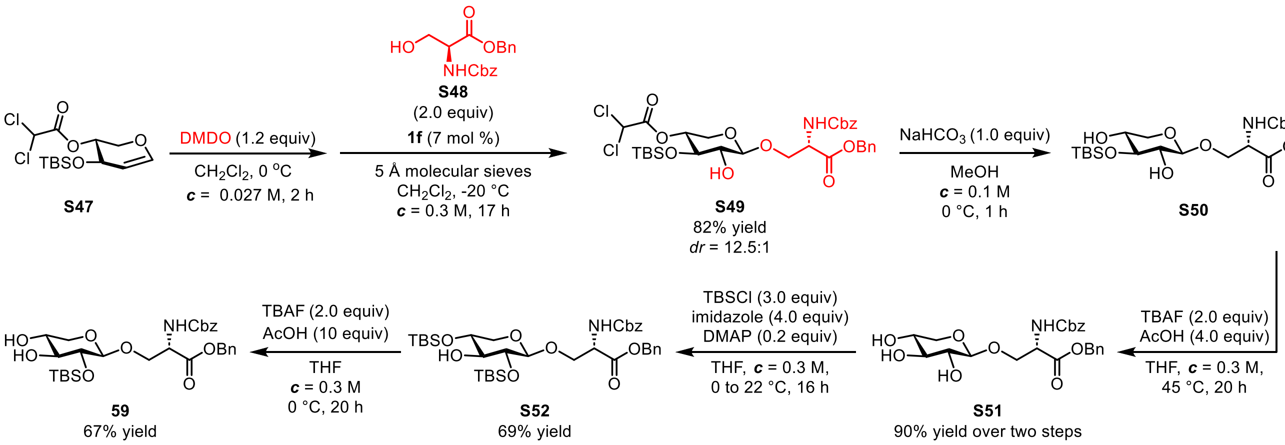

Figure S7. Synthesis of Xylose-β-1-*O*-serine-Derived Glycosyl Acceptor **59**.

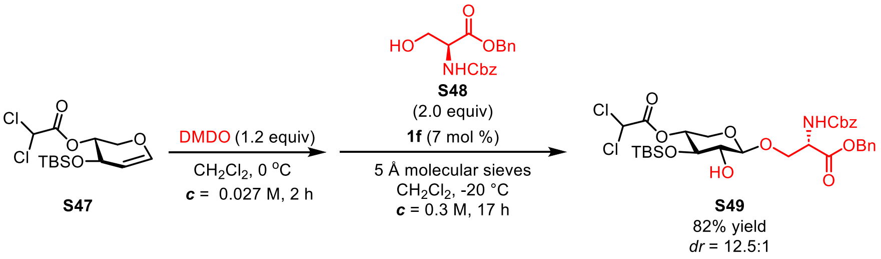

S47 was synthesized according to a modified literature procedure.[1] **S48** was synthesized according to a literature procedure.[20]

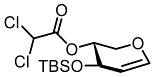

S47

1,5-Anhydro-3-*O*-*tert*-butyldimethylsilyl-2-deoxy-4-*O*-dichloroacetyl-D-*threo*-pent-1-enitol (**S47**): $[\alpha]_D^{23}$ −123.7 (acetone, *c* = 1.0); IR ν_{max} (neat)/cm^{-1}: 2955 (m), 2930 (m), 2858 (m), 2361 (m), 1749 (s), 1645 (m), 1364 (m), 1245 (s), 1073 (s), 837 (s), 778 (s); ^{1}H NMR (400 MHz, $CDCl_3$) δ 6.48 (d, *J* = 6.2 Hz, 1H), 5.95 (s, 1H), 4.86 (dt, *J* = 3.2, 1.6 Hz, 1H), 4.83 (ddd, *J* = 6.4, 4.8, 1.6 Hz, 1H), 4.16 (ddd, *J* = 12.2, 3.3, 1.6 Hz, 1H), 4.08 (dd, *J* = 12.2, 1.8 Hz, 1H), 4.04 – 3.97 (m, 1H), 0.89 (s, 9H), 0.12 (s, 3H), 0.11 (s, 3H); ^{13}C NMR (100 MHz, $CDCl_3$) δ 164.1, 145.8, 101.4, 73.6, 64.2, 62.4, 61.1, 25.9 (3C), 18.1, -4.2, -4.5; HRMS: m/z (ESI) calcd for $C_{13}H_{23}Cl_2O_4Si^+$, $[M + H]^+$, 341.0737, found 341.0742.

To a 25 mL flask equipped with a stir bar at 0 °C was added the glycal **S47** (34.1 mg, 0.1 mmol, 1.0 equiv) in CH_2Cl_2 (1.7 mL), followed by the addition of dimethyldioxirane (2 mL, DMDO in acetone, 0.06 M, 0.12 mmol, 1.2 equiv). After stirring at 0 °C for 2 h with progress monitored by ^{1}H NMR, the reaction mixture was concentrated *in vacuo*. The residue was re-dissolved in anhydrous CH_2Cl_2 (2 mL) and dried over anhydrous Na_2SO_4. The organic phase was transferred to a 2-dram vial and concentrated *in vacuo*. The residue was further dried azeotropically with anhydrous toluene (1 mL × 2) and directly used in the next step. The diastereomeric ratio of the obtained glycal epoxide was determined by ^{1}H NMR as 16.7:1.

To a flame-dried sealable 2-dram vial equipped with a stir bar were added iron catalyst **1f** (6.4 mg, 0.007 mmol, 7 mol %), acceptor **S48** (65.9 mg, 0.2 mmol, 2.0 equiv), and freshly activated 5 Å molecular sieves, powder (*ca.* 100 mg). After the vial was evacuated and backfilled with N_2 three times, anhydrous CH_2Cl_2 (0.33 mL) was added. The mixture was cooled to -20 °C and stirred for 5 min before a solution of glycal epoxide obtained from the previous step in anhydrous CH_2Cl_2 was added. The reaction was kept at -20 °C for 17 h and quenched with a solution of imidazole (7 mg) in CH_2Cl_2 (0.83 mL) at the same temperature. The mixture was filtered through a pad of Celite® silica gel and eluted with EtOAc. The organic layer was then concentrated *in vacuo* and the *dr* of the crude reaction mixture was determined as 12.5:1 based

on the ^{1}H NMR analysis. The residue was purified through column chromatography (hexanes/EtOAc: from 20:1 to 3:1) to afford the desired product **S49** as colorless oil (56.4 mg, 82% yield).

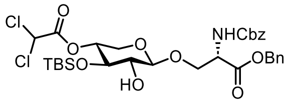

S49

***N*-Benzyloxycarbonyl-*O*-(3-*O*-*tert*-butyldimethylsilyl-4-*O*-dichloroacetyl-β-D-xylopyranosyl)-L-serine benzyl ester** (**S49**): $[\alpha]_D^{22}$ −25.3 (acetone, *c* = 0.7); IR ν_{max} (neat)/cm^{-1}: 3425 (w), 2955 (w), 2929 (w), 2887 (w), 2856 (w), 1750 (s), 1724 (s), 1515 (m), 1339 (m), 1252 (s), 1212 (s), 1165 (s), 1119 (s), 1068 (s), 993 (s), 838 (s), 780 (m); ^{1}H NMR (400 MHz, $CDCl_3$) δ 7.47 – 7.29 (m, 10H), 5.93 (s, 1H), 5.76 (d, *J* = 8.5 Hz, 1H), 5.27 – 5.16 (m, 2H), 5.16 – 5.04 (m, 2H), 4.80 – 4.73 (m, 1H), 4.63 – 4.57 (m, 1H), 4.28 – 4.19 (m, 2H), 3.92 (dd, *J* = 11.8, 5.1 Hz, 1H), 3.85 (d, *J* = 10.6 Hz, 1H), 3.71 (td, *J* = 8.2, 1.7 Hz, 1H), 3.28 (t, *J* = 7.8 Hz, 1H), 3.13 (ddd, *J* = 11.2, 9.0, 1.7 Hz, 1H), 2.64 (brs, 1H), 0.87 (s, 9H), 0.12 (s, 3H), 0.09 (s, 3H); ^{13}C NMR (100 MHz, $CDCl_3$) δ 169.9, 164.0, 156.2, 136.3, 135.2, 128.8 (two peaks overlapped, 4C), 128.7 (2C), 128.6 (2C), 128.4, 128.3, 103.6, 75.3, 73.8, 73.4, 69.9, 67.7, 67.3, 64.1, 61.4, 54.7, 25.9 (3C), 18.2, -4.14, -4.64; HRMS: m/z (ESI) calcd for $C_{31}H_{42}Cl_2NO_{10}^+$, $[M + H]^+$, 686.1950, found 686.1965. $^{1}J_{^{13}C1\text{–}H1}$ = 160.9 Hz.

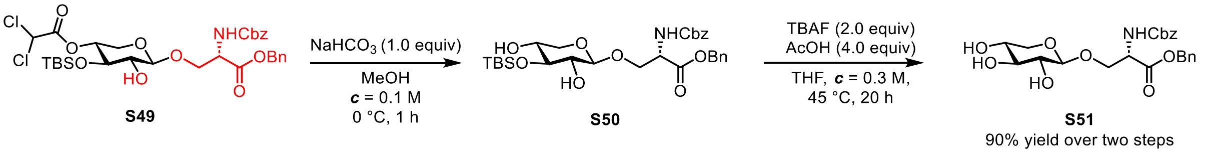

To a 2-dram vial equipped with a stir bar were added compound **S49** (412 mg, 0.6 mmol, 1.0 equiv) and MeOH (6 mL). The solution was cooled to 0 °C, and anhydrous $NaHCO_3$ (powder, 50.4 mg, 0.6 mmol, 1.0 equiv) was then added. The reaction was raised to room temperature and stirred for 1 h with progress monitored by TLC until completion. AcOH (34 µL, 0.6 mmol, 1.0 equiv) was then added to quench the reaction. The solution was concentrated *in vacuo* to afford

the crude desired product **S50** as colorless oil which was directly used in the next step without further purification.

To a sealable 2-dram vial equipped with a stir bar containing **S50** (0.60 mmol, 1.0 equiv) obtained from the last step was added THF (0.66 mL). The vial was cooled to 0 °C, and a premixed solution of TBAF (1 M in THF, 1.2 mL, 1.2 mmol, 2.0 equiv) and AcOH (0.14 mL, 2.4 mmol, 4 equiv) was added. The reaction mixture was stirred for 20 h at 0 °C, with progress monitored by TLC until completion. The reaction mixture was directly concentrated *in vacuo*, and the residue was purified through column chromatography (hexanes/acetone: from 100:1 to 1:1) to afford the desired product **S51** (249 mg, 90% yield over two steps) as colorless oil.

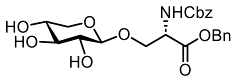

S51

***N*-Benzyloxycarbonyl-*O*-(β-D-xylopyranosyl)-L-serine benzyl ester** (**S51**): $[\alpha]_D^{22}$ -24.4 (acetone, *c* = 1.0); IR ν_{max} (neat)/cm^{-1}: 3455 (w), 3016 (w), 2970 (w), 2360 (w), 2342 (w), 1738 (s), 1522 (w), 1455 (w), 1366 (m), 1229 (s), 1217 (s), 1071 (s), 898 (w), 742 (w); ^{1}H NMR (400 MHz, acetone-d$_6$) δ 7.46 – 7.25 (m, 10H), 6.80 (brs, 1H), 5.24 (d, *J* = 12.7 Hz, 1H), 5.17 (d, *J* = 12.5 Hz, 1H), 5.15 – 5.04 (m, 2H), 4.79 – 4.47 (m, 2H), 4.40 (dd, *J* = 10.0, 3.5 Hz, 1H), 4.28 (d, *J* = 7.2 Hz, 1H), 4.17 (brs, 2H), 3.87 – 3.75 (m, 2H), 3.56 – 3.46 (m, 1H), 3.41 – 3.31 (m, 1H), 3.25 – 3.14 (m, 2H); ^{13}C NMR (100 MHz, acetone-d$_6$) δ 170.9, 157.3, 138.1, 137.2, 129.4 (two peaks overlapped, 4C), 128.9 (2C), 128.8 (2C), 128.7 (two peaks overlapped, 2C), 105.0, 77.2, 74.4, 70.8, 70.4, 67.4, 67.1, 66.5, 55.6; HRMS: m/z (ESI) calcd for $C_{23}H_{28}NO_9^+$, $[M + H]^+$, 462.1759, found 462.1773.

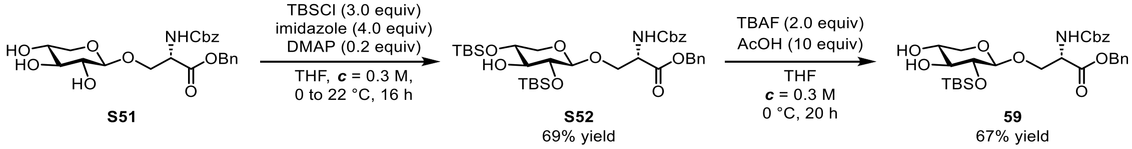

To a 25 mL flame-dried round bottom flask equipped with a stir bar were added **S51** (276 mg, 0.6 mmol, 1 equiv), imidazole (163 mg, 2.4 mmol, 4.0 equiv), and anhydrous THF (2 mL). The flask was cooled to 0 °C. TBSCl (272 mg, 1.8 mmol, 3.0 equiv) was then added. The reaction mixture was gradually warmed to room temperature and stirred for 16 h, with progress monitored until completion. The reaction mixture was quenched with MeOH (0.15 mL), diluted with EtOAc (5 mL), and washed with water (10 mL). The organic phase was separated from the aqueous one, which was then extracted with EtOAc (5 mL × 3). The combined organic phase was washed with brine (20 mL) and dried over Na_2SO_4. After concentration *in vacuo*, the residue was purified through column chromatography (hexanes/EtOAc: from 100:1 to 9:1) to afford the desired product **S52** (286 mg, 69% yield) as colorless oil.

To a sealable 2-dram vial equipped with a stir bar were added **S52** (286 mg, 0.41 mmol, 1.0 equiv) and THF (0.32 mL). The vial was cooled to 0 °C, and a premixed solution of TBAF (1 M in THF, 0.82 mL, 1 mmol, 2.0 equiv) and AcOH (0.23 mL, 4.1 mmol, 10.0 equiv) was added. The reaction mixture was stirred for 20 h at 0 °C, with progress monitored by TLC until completion. The reaction mixture was diluted with EtOAc (5 mL) and quenched with saturated aqueous NH_4Cl solution (10 mL). The organic phase was separated from the aqueous one, which was then extracted with EtOAc (5 mL × 4). The combined organic phase was washed with brine (15 mL) and dried over Na_2SO_4. After concentration *in vacuo,* the residue was purified through column chromatography (hexanes/acetone: from 100:1 to 3:1) to afford the desired product **59** (158 mg, 67% yield) as colorless oil.

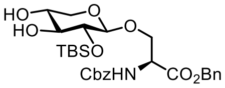

59

***N*-Benzyloxycarbonyl-*O*-(2-*O*-*tert*-butyldimethylsilyl-β-D-xylopyranosyl)-L-serine benzyl ester** (**59**): $[\alpha]_D^{22}$ +43.4 (acetone, *c* = 1.0); IR ν_{max} (neat)/cm^{-1}: 33442 (w), 2970 (w), 2951 (w), 2856 (w), 2360 (w), 2342 (w), 1738 (s), 1507 (w), 1456 (w), 1365 (s), 1229 (s), 1217 (s), 1142 (w), 1050 (m), 838 (m), 780 (w); ^{1}H NMR (400 MHz, $CDCl_3$) δ 7.40 – 7.30 (m, 10H), 5.65 (d, *J* = 8.4 Hz, 1H), 5.24 (d, *J* = 12.1 Hz, 1H), 5.19 – 5.10 (m, 3H), 4.63 (dt, *J* = 8.0, 3.3 Hz, 1H), 4.46

(d, J = 3.5 Hz, 1H), 4.17 (dd, J = 10.0, 3.6 Hz, 1H), 3.76 (dd, J = 10.0, 3.2 Hz, 1H), 3.70 (dd, J = 12.1, 2.8 Hz, 1H), 3.62 – 3.47 (m, 3H), 3.32 (dd, J = 12.1, 4.5 Hz, 1H), 3.24 (brs, 1H), 2.67 (brs, 1H), 0.89 (s, 9H), 0.11 (s, 3H), 0.09 (s, 3H); ^{13}C NMR (100 MHz, $CDCl_3$) δ 169.7, 155.6, 135.6, 134.8, 128.7 (two peaks overlapped, 4C), 128.59 (2C), 128.55 (2C), 128.3, 128.1, 101.1, 71.6, 70.8, 68.9, 68.3, 67.7, 67.2, 61.8, 54.1, 25.7 (3C), 18.0, -4.9, -5.0; HRMS: m/z (ESI) calcd for $C_{29}H_{42}NO_9Si^+$, $[M + H]^+$, 576.2623, found 576.2601.

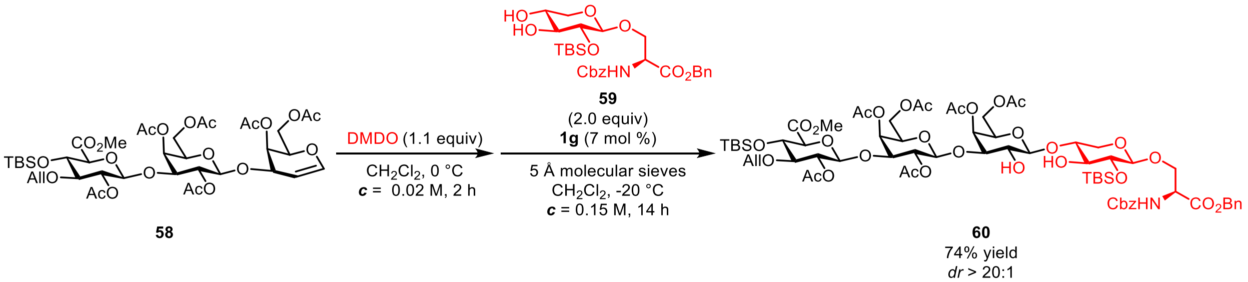

To a 25 mL flask equipped with a stir bar at 0 °C was added glycal **58** (90.5 mg, 0.1 mmol, 1.0 equiv) in CH_2Cl_2 (3.2 mL), followed by addition of dimethyldioxirane (0.06 M solution in acetone, 1.83 mL, 0.11 mmol, 1.1 equiv) dropwise. After stirring at 0 °C for 2 h, with progress monitored by ^{1}H NMR until completion. The reaction mixture was concentrated *in vacuo*, then the residue was re-dissolved in anhydrous CH_2Cl_2 (2 mL) and dried over anhydrous Na_2SO_4. The organic phase was concentrated *in vacuo*. The residue was further dried azeotropically with anhydrous toluene (1 mL × 2) and directly used in the next step. The diastereomeric ratio of the obtained glycal epoxide was determined by ^{1}H NMR as >20:1.

To a flame-dried sealable 2-dram vial equipped with a stir bar were added a glycosyl acceptor **59** (115 mg, 0.2 mmol, 2.0 equiv), iron catalyst **1g** (5.6 mg, 0.007 mmol, 7 mol %), and freshly activated 5 Å molecular sieves, powder (*ca.* 100 mg). After the vial was evacuated and backfilled with N_2, anhydrous CH_2Cl_2 (0.16 mL) was added and the solution was stirred at -78 °C for 10 min. A solution of the aforementioned glycal epoxide in anhydrous CH_2Cl_2 (0.3 mL) was then added to the mixture at -78 °C dropwise. The reaction mixture was kept at -20 °C for 24 h and then quenched with a solution of imidazole (7 mg) in CH_2Cl_2 (0.7 mL) at the same temperature. The mixture was filtered through a pad of Celite® silica gel and eluted with EtOAc. The organic layer was then concentrated *in vacuo*, and the residue was purified through column

chromatography (hexanes/acetone: from 20:1 to 2:1) to afford the corresponding glycosylation product **60** as white foam (111 mg, 74% yield).

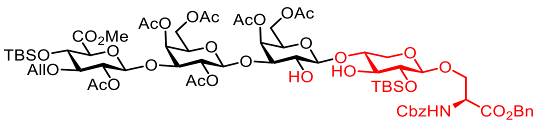

60

***N*-Benzyloxycarbonyl-*O*-[(methyl 2-*O*-acetyl-3-*O*-allyl-4-*O*-*tert*-butyldimethylsilyl-β-D-glucopyranosyluronate)-(1→3)-(2,4,6-tri-*O*-acetyl-β-D-galactopyranosyl)-(1→3)-(4,6-di-*O*-acetyl-β-D-galactopyranosyl)-(1→4)-(2-*O*-*tert*-butyldimethylsilyl-β-D-xylopyranosyl)]-L-serine benzyl ester (60)**: $[\alpha]_D^{22}$ +11.9 (acetone, *c* = 1.0); IR ν_{max} (neat)/cm^{-1}: 3490 (w), 2928 (w), 2857 (w), 1749 (s), 1501 (w), 1438 (w), 1371 (m), 1224 (s), 1141 (m), 1075 (s), 839 (m), 780 (m), 753 (w); ^{1}H NMR (400 MHz, acetone-d_6) δ 7.56 – 7.14 (m, 10H), 6.20 (d, *J* = 6.8 Hz, 1H), 5.87 (ddt, *J* = 16.2, 10.6, 5.4 Hz, 1H), 5.43 – 5.30 (m, 2H), 5.27 – 5.15 (m, 3H), 5.13 – 5.04 (m, 4H), 4.94 (d, *J* = 8.0 Hz, 1H), 4.83 – 4.75 (d, *J* = 7.6 Hz, 2H), 4.60 – 4.51 (m, 1H), 4.49 (d, *J* = 7.8 Hz, 1H), 4.38 (d, *J* = 3.9 Hz, 1H), 4.27 (d, *J* = 7.2 Hz, 1H), 4.24 – 4.13 (m, 4H), 4.13 – 4.03 (m, 3H), 4.03 – 3.96 (m, 2H), 3.96 – 3.83 (m, 6H), 3.75 (s, 3H), 3.72 – 3.64 (m, 1H), 3.60 (td, *J* = 9.3, 5.6 Hz, 1H), 3.47 (t, *J* = 8.0 Hz, 1H), 3.45 – 3.39 (m, 1H), 3.35 – 3.22 (m, 2H), 2.10 (s, 3H), 2.07 (s, 3H), 2.05 – 2.03 (m, 6H), 2.01 (s, 3H), 2.00 (s, 3H), 0.90 (s, 9H), 0.86 (s, 9H), 0.12 (s, 3H), 0.11 (s, 3H), 0.09 (s, 3H), 0.03 (s, 3H); ^{13}C NMR (100 MHz, acetone-d_6) δ 170.82, 170.79, 170.7(two peaks overlapped, 2C), 170.5, 170.1, 169.7, 169.3, 156.8, 138.1, 137.1, 135.9, 129.42 (2C), 129.36 (2C), 129.1, 128.9(2C), 128.80 (2C), 128.79, 116.8, 104.7, 104.6, 102.2, 101.5, 82.7, 81.1, 79.2, 77.0, 76.8, 76.6, 75.9, 74.3, 73.9, 73.1, 72.7, 72.1, 72.0, 71.2, 70.4, 70.1, 69.7, 67.6, 67.1, 64.5, 63.6, 62.8, 55.7, 52.7, 26.5 (3C), 26.3 (3C), 21.3, 21.2, 20.92, 20.87 (two peaks overlapped, 2C), 20.8, 19.0, 18.6, -3.6, -3.9, -4.1, -4.9; HRMS: m/z (ESI) calcd for $C_{69}H_{102}NO_{31}Si_2^+$, [M + H]$^+$, 1496.5969, found 1496.5943. $^1J_{^{13}C1\text{–}H1}$ = 162.3 Hz, 163.6 Hz, 163.6 Hz, 161.3 Hz.

E. Preliminary Kinetic Studies of the Iron-Catalyzed Stereospecific Glycosylation with Glycal Epoxides

To better understand the underlying mechanism of this iron-catalyzed stereospecific glycosylation with glycal epoxides, particularly the reaction rate order dependence over the iron catalyst and each substrate, we carried out kinetic studies by measuring the initial reaction rate of three selected model reactions.

Model Reaction A: the iron-catalyzed glycosylation between glycal epoxide **61** and primary glycosyl acceptor **62**.

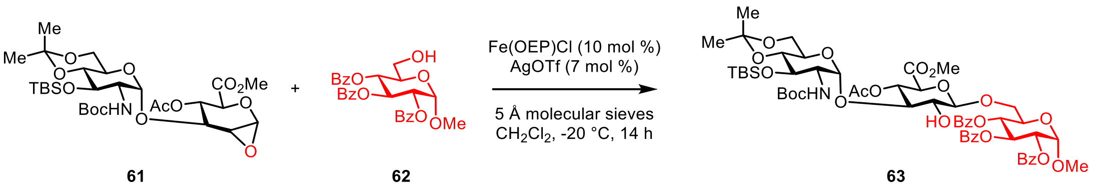

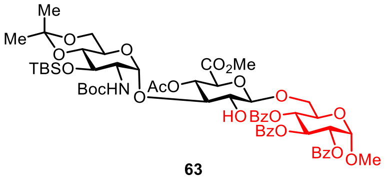

63

Methyl 2-*O*-*tert*-butoxycarbonyl-3-*O*-*tert*-butyldimethylsilyl-4,6-*O*-isopropylidene-α-D-glucopyranosyl-(1→3)-(methyl 4-*O*-acetyl-β-D-glucopyranosyluronate)-(1→6)-2,3,4-tri-*O*-benzoyl-α-D-glucopyranoside (**63**): ^{1}H NMR (400 MHz, $CDCl_3$) δ 7.98 – 7.89 (m, 4H), 7.87 – 7.80 (m, 2H), 7.55 – 7.43 (m, 2H), 7.41 – 7.31 (m, 5H), 7.26 (t, J = 7.7 Hz, 2H), 6.15 (t, J = 9.9 Hz, 1H), 5.73 (t, J = 9.9 Hz, 1H), 5.28 – 5.20 (m, 2H), 5.15 (t, J = 9.5 Hz, 1H), 4.88 (d, J = 3.8 Hz, 1H), 4.58 (d, J = 10.2 Hz, 1H), 4.31 – 4.19 (m, 2H), 4.16 (d, J = 10.3 Hz, 1H), 4.11 – 4.01 (m, 1H), 3.88 – 3.79 (m, 3H), 3.73 – 3.67 (m, 2H), 3.69 (s, 3H), 3.66 – 3.57 (m, 3H), 3.55 – 3.46 (m, 2H), 3.45 (s, 3H), 2.03 (s, 3H), 1.46 (s, 3H), 1.42 (s, 9H), 1.40 (s, 3H), 0.86 (s, 9H), 0.07 (s, 3H), 0.06 (s, 3H); ^{13}C NMR (100 MHz, $CDCl_3$) δ 169.6, 167.5, 166.1, 165.83, 165.79, 155.4, 133.8, 133.4, 133.2, 130.1 (2C), 130.0 (2C), 129.7 (2C), 129.2, 129.1, 128.6 (two peaks

overlapped, 3C), 128.5 (2C), 128.4 (2C), 103.5, 100.6, 99.3, 97.2, 79.8, 79.7, 74.7, 72.9, 72.2, 72.1, 71.4, 71.0, 70.4, 69.1, 68.5, 68.3, 64.1, 62.3, 55.9, 55.7, 52.8, 29.1, 28.6 (3C), 25.9 (3C), 20.9, 19.1, 18.4, -4.0, -4.8; $^{1}J_{^{13}C1\text{–}H1}$ = 175.4 Hz, 175.4 Hz, 160.0 Hz. HRMS: m/z (ESI) calcd for $C_{57}H_{76}NO_{22}Si^{+}$, $[M + H]^{+}$, 1154.4623, found 1154.4656.

Model Reaction B: the iron-catalyzed glycosylation between glycal epoxide **61** and primary glycosyl acceptor **64**.

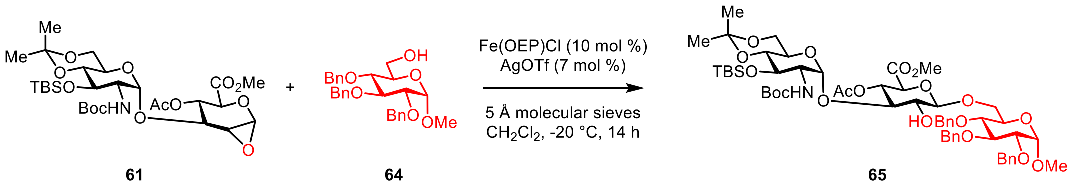

61 1.0 equiv *dr* : 9:1 | **64** 2.0 equiv | **65** 93% yield *dr* >20:1

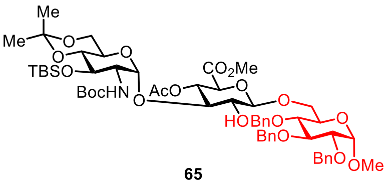

65

Methyl 2-*O*-*tert*-butoxycarbonyl-3-*O*-*tert*-butyldimethylsilyl-4,6-*O*-isopropylidene-α-D-glucopyranosyl-(1→3)-(methyl 4-*O*-acetyl-β-D-glucopyranosyluronate)-(1→6)-2,3,4-tri-*O*-benzyl-α-D-glucopyranoside (**65**): ^{1}H NMR (400 MHz, $CDCl_3$) δ 7.39 – 7.27 (m, 15H), 5.14 (t, *J* = 9.6 Hz, 1H), 4.99 (d, *J* = 10.9 Hz, 1H), 4.95 – 4.85 (m, 2H), 4.85 – 4.75 (m, 2H), 4.66 (d, *J* = 12.1 Hz, 1H), 4.64 – 4.57 (m, 2H), 4.51 (d, *J* = 10.0 Hz, 1H), 4.26 (d, *J* = 7.7 Hz, 1H), 4.11 (dd, *J* = 11.1, 2.4 Hz, 1H), 3.99 (t, *J* = 9.2 Hz, 1H), 3.96 – 3.77 (m, 5H), 3.72 (s, 3H), 3.70 – 3.63 (m, 2H), 3.65 – 3.55 (m, 2H), 3.56 – 3.43 (m, 4H), 3.38 (s, 3H), 3.20 (brs, 1H), 2.07 (s, 3H), 1.47 (s, 3H), 1.44 (s, 9H), 1.41 (s, 3H), 0.87 (s, 9H), 0.08 (s, 3H), 0.07 (s, 3H); ^{13}C NMR (100 MHz, $CDCl_3$) δ 169.8, 167.5, 155.3, 138.8, 138.4, 138.2, 128.6 (2C), 128.54 (2C), 128.48 (2C), 128.2 (2C), 128.0 (two peaks overlapped, 3C), 127.9 (2C), 127.8, 127.7, 103.5, 100.9, 99.5, 98.2, 82.0, 81.6, 79.9, 79.8, 78.2, 75.8, 75.1, 74.7, 73.5, 72.8, 72.3, 71.0, 70.7, 69.8, 69.4, 64.6, 62.2, 55.7,

55.4, 52.8, 29.1, 28.6 (3C), 25.9 (3C), 20.9, 19.0, 18.4, -4.1, -4.8; $^{1}J_{^{13}C1-H1}$ = 170.5 Hz, 172.0 Hz, 160.1 Hz. HRMS: m/z (ESI) calcd for $C_{57}H_{82}NO_{19}Si^{+}$, $[M + H]^{+}$, 1112.5245, found 1112.5216.

Model Reaction C: the iron-catalyzed glycosylation between glycal epoxide **61** and secondary glycosyl acceptor **66**.

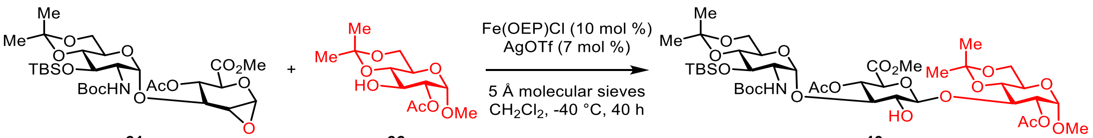

Since the glycal epoxide is reactive towards the glycosyl acceptor and moisture in the presence of an iron catalyst, we implemented kinetics studies by taking aliquots that are quenched (by imidazole in CH_2Cl_2 solution) at the same temperature of the reaction. We selected using ^{1}H NMR for product yield determination with 1,3,5-trimethoxybenzene added as the internal standard.

It is known from synthetic studies that this iron-catalyzed glycosylation is essentially halted at -78 °C. To facilitate the operation and ensure data reliability, we mixed all the substrates and the catalyst at -78 °C and kept it at that temperature for 3 min before moving the reaction to a -20 °C cold bath and initiating the kinetic measurement.

Compared to the synthetic experiment conditions, we decreased the concentration of glycal epoxide **61** in Model Reactions A–C from *0.15 M* to *0.1 M* and decreased the iron catalyst loading from *7 mol %* to *2.5–5.0 mol %* to ensure enough data points can be accurately collected before the yield reaches 20%.

a. Preliminary Kinetic Studies for Model Reaction A

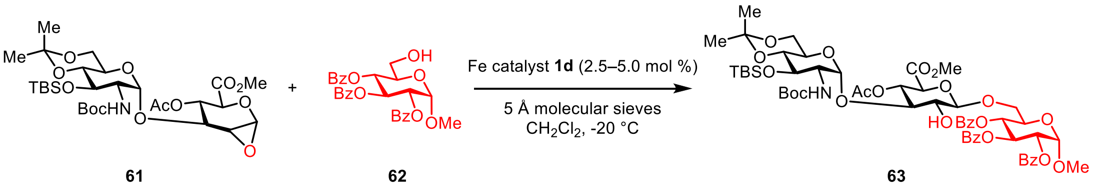

61
1.0–2.0 equiv
dr : 9:1

62
2.0–4.0 equiv

63
dr >20:1

To a flame-dried sealable 2-dram vial (vial A) equipped with a stir bar were added a glycosyl acceptor **62** (121–242 mg, 0.24–0.48 mmol, 2.0–4.0 equiv), Fe(OEP)Cl (2.3–4.6 mg, 0.0036–0.0072 mmol, 3–6 mol %), AgOTf (0.77–1.54 mg, 0.003–0.006 mmol, 2.5–5.0 mol %), and freshly activated 5 Å molecular sieves, powder (*ca.* 100 mg). After the vial was evacuated and backfilled with N_2 three times, anhydrous CH_2Cl_2 (0.6 mL) was added, and the solution was stirred at 0 °C for 10 min before cooled to -78 °C. To a second flame-dried sealable 2-dram vial (vial **B**) was added glycal epoxide **61** (78–156 mg, 0.12–0.24 mmol, 1.0–2.0 equiv)**.** Vial **B** was evacuated and backfilled with N_2 three times, and then anhydrous CH_2Cl_2 (0.6 mL) was added, and glycal epoxide **61** was transferred to vial A at -78 °C dropwise within 1 min. The reaction was kept at -78 °C for an additional 3 min before being transferred to -20 °C. After 5–90 min, an aliquot (0.05 mL of reaction mixture) was taken and quenched by MeOH (5 µL) and imidazole (0.2 mL, 0.125 M in CH_2Cl_2) at the same temperature. The mixture was filtered through a pad of silica gel and Na_2SO_4 and eluted with EtOAc (1 mL × 3). The filtrate was then concentrated *in vacuo*. The product distribution in the residue was determined through quantitative NMR analysis.

We first aimed to determine the initial rate order dependence on iron catalyst **1d**. In these experiments, the initial concentration of glycal epoxide **61** is 0.1 M, and the initial concentration of primary glycosyl acceptor **62** is 0.2 M. We measured the initial rates with iron catalyst loadings ranging from 2.5 mol %, 3.75 mol %, to 5.0 mol % (Figure S8).

Based on the concentration increase of glycosylation product **63**, the corresponding initial rates are 0.236 mM·min^{-1}, 0.351 mM·min^{-1}, and 0.460 mM·min^{-1}, respectively.

These data suggested that the initial rate has a first-order dependence on iron catalyst ***1d****.*

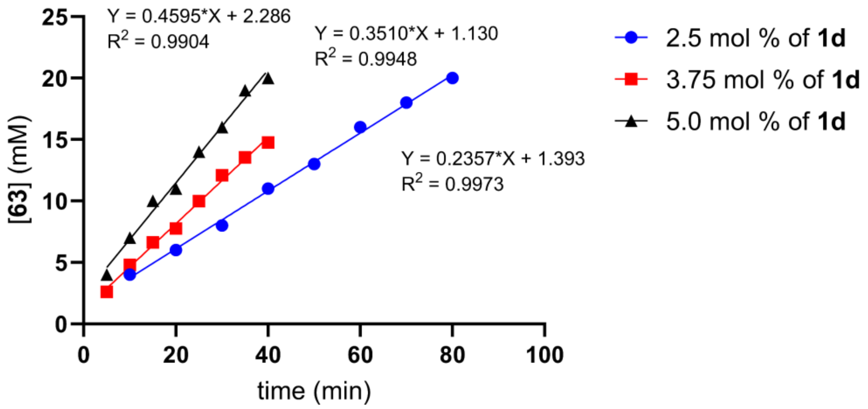

Figure S8. Initial Rate Measurement with Different Iron Catalyst Loadings. Measurement was based on the concentration increase of the glycosylation product **63**. Reactions were performed on a 0.12 mmol scale with epoxide **61** (1.0 equiv), glycosyl acceptor **62** (2.0 equiv) in CH_2Cl_2 with 5 Å molecular sieves at -20 °C.

We next evaluated the initial rate order dependence on glycal epoxide **61**. In these experiments, the initial concentration of glycosyl acceptor **62** is 0.2 M, the initial concentration of iron catalyst **1d** is 0.0025 M. We measured the initial rates when the concentration of glycal epoxide **61** varies from 0.1 M, 0.15 M to 0.2 M (Figure S9).

Based on the concentration increase of glycosylation product **63**, the corresponding initial rates are 0.236 $mM \cdot min^{-1}$, 0.349 $mM \cdot min^{-1}$, and 0.450 $mM \cdot min^{-1}$, respectively.

These data suggested that the initial rate has a first-order dependence on glycal epoxide ***61***.

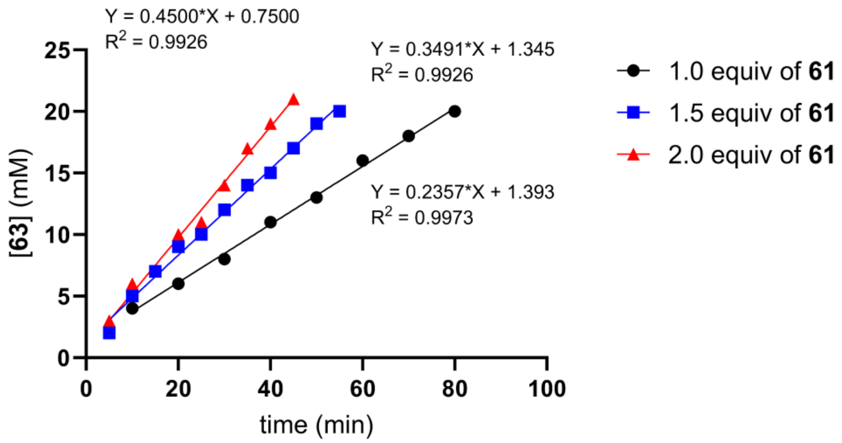

Figure S9. Initial Rate Measurement with Different Glycal Epoxide Concentrations. Measurement was based on concentration increase of the glycosylation product **63**. Reactions were performed on a 0.12 mmol scale with glycosyl acceptor **62** (2.0 equiv) and iron catalyst **1d** (2.5 mol %) in CH_2Cl_2 with 5 Å molecular sieves at -20 °C.

We further evaluated the initial rate order dependence on primary glycosyl acceptor **62**. In these experiments, the initial concentration of glycal epoxide **61** is 0.1 M, and the initial concentration of iron catalyst **1d** is 0.0025 M. We measured the initial rates with the primary glycosyl acceptor **62** ranging from 2.0 equiv, 3.0 equiv, to 4.0 equiv (Figure S10).

Based on the concentration increase of glycosylation product **63**, the corresponding initial rates are 0.236 $mM \cdot min^{-1}$, 0.240 $mM \cdot min^{-1}$, and 0.243 $mM \cdot min^{-1}$, respectively.

*These data suggested that the initial rate has zero-order dependence on primary acceptor **62**.*

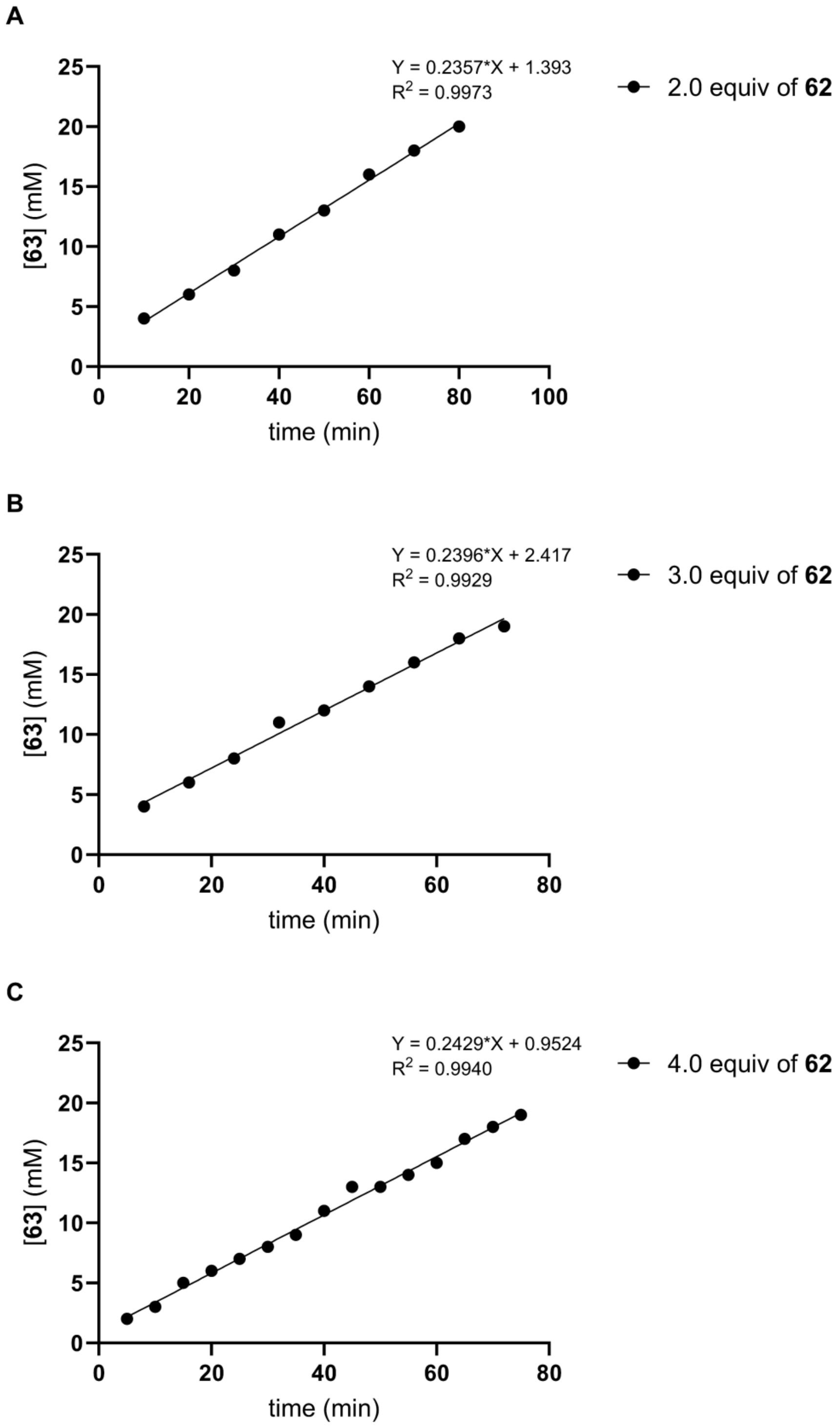

Figure S10. Initial Rate Measurement with Different Glycosyl Acceptor Concentrations. (**A**) Kinetic profile with 1.0 equiv of glycosyl acceptor **62**. (**B**) Kinetic profile with 2.0 equiv of

glycosyl acceptor **62**. (**C**) Kinetic profile with 3.0 equiv of glycosyl acceptor **62**. Reactions were performed on a 0.12 mmol scale with epoxide **61** (1.0 equiv), and iron catalyst **1d** (2.5 mol %) in CH_2Cl_2 with 5 Å molecular sieves at -20 °C.

These preliminary kinetic studies suggested that the initial rate has a *first-order* dependence on both the iron catalyst and the glycal epoxide **61**. However, the initial rate has *zero-order* dependence on glycosyl acceptor **62**.

b. Preliminary Kinetic Studies for Model Reaction B

To further investigate whether the zero-order rate dependence on a primary glycosyl acceptor is general, we next evaluated the kinetics of this glycosylation with a more nucleophilic primary acceptor **64**.

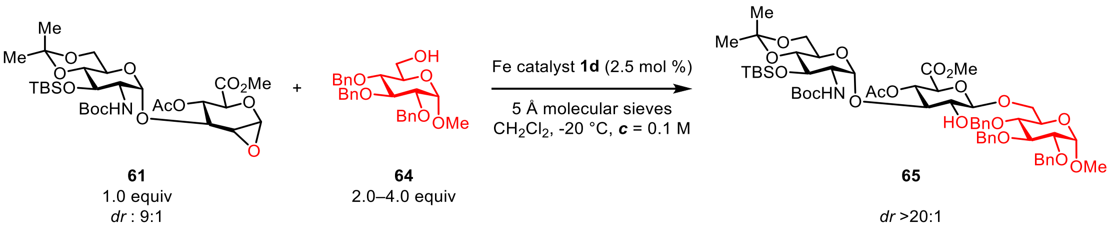

To a flame-dried sealable 2-dram vial (vial A) equipped with a stir bar were added a glycosyl acceptor **64** (112–223 mg, 0.24–0.48 mmol, 2.0–4.0 equiv), Fe(OEP)Cl (2.3 mg, 0.0036 mmol, 3 mol %), AgOTf (0.77 mg, 0.003 mmol, 2.5 mol %), and freshly activated 5 Å molecular sieves, powder (*ca.* 100 mg). After the vial was evacuated and backfilled with N_2 three times, anhydrous CH_2Cl_2 (0.6 mL) was added, and the solution was stirred at 0 °C for 10 min before cooled to -78 °C. To a second flame-dried sealable 2-dram vial (vial **B**) was added epoxide **61** (78, 0.12 mmol, 1.0 equiv). Vial **B** was evacuated and backfilled with N_2 three times, and then anhydrous CH_2Cl_2 (0.6 mL) was added, and epoxide **61** was transferred to vial **A** at -78 °C dropwise within 1 min. The reaction was kept at -78 °C for an additional 3 min before being transferred to -20 °C. After 20–200 min, an aliquot (0.05 mL of reaction mixture) was taken and quenched by MeOH (5 μL) and imidazole (0.2 mL, 0.125 M in CH_2Cl_2) at the same temperature.

The mixture was filtered through a pad of silica gel and Na_2SO_4 and eluted with EtOAc (1 mL × 3). The filtrate was then concentrated *in vacuo*. The product distribution in the residue was determined through quantitative NMR analysis.

We aimed to determine the initial rate order dependence on primary acceptor **64**. In these experiments, the initial concentration of glycal epoxide **61** is 0.1 M, and the initial concentration of iron catalyst **1d** is 0.0025 M. We measured the initial rates with primary acceptor loadings ranging from 2.0 equiv, 3.0 equiv, to 4.0 equiv (Figure S11).

Based on concentration increase of glycosylation product **65**, the corresponding initial rates are 0.091 mM·min^{-1}, 0.091 mM·min^{-1}, and 0.092 mM·min^{-1}, respectively.

These data suggest that the initial rate has zero-order dependence on primary acceptor ***64****.*

A

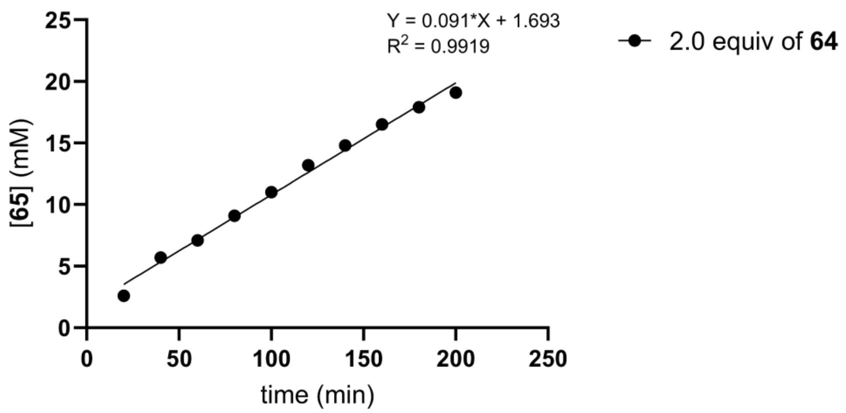

B

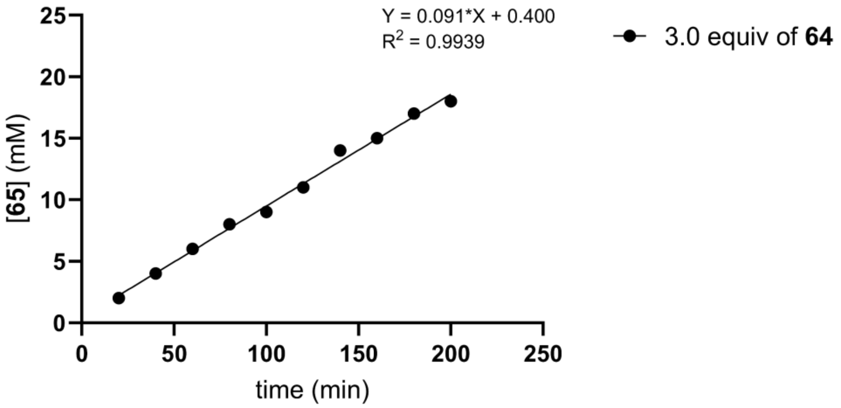

C

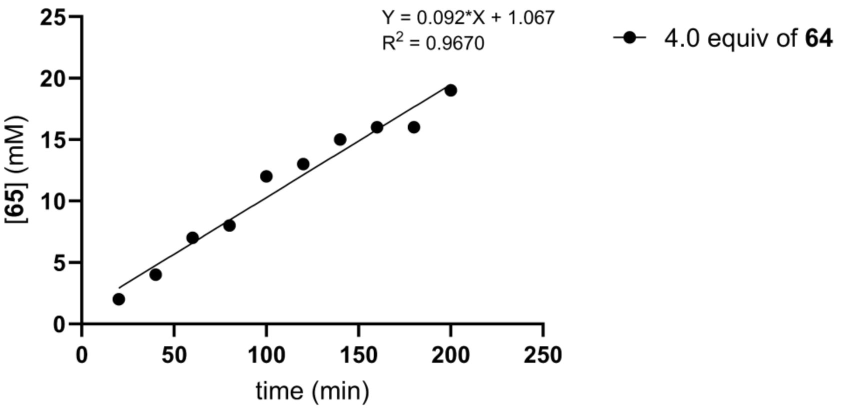

Figure S11. Initial Rate Measurement with Different Glycosyl Acceptor Concentrations. (**A**) Kinetic profile with 2.0 equiv of glycosyl acceptor **64**. (**B**) Kinetic profile with 3.0 equiv of glycosyl acceptor **64**. (**C**) Kinetic profile with 4.0 equiv of glycosyl acceptor **64**. Reactions were performed on a 0.12 mmol scale with epoxide **61** (1.0 equiv), and iron catalyst **1d** (2.5 mol %) in CH_2Cl_2 with 5 Å molecular sieves at -20 °C.

Comparing the initial rates of two primary glycosyl acceptors (**62** and **64**) with the same glycal epoxide, a more nucleophilic acceptor **64** seems to react slower than a less nucleophilic acceptor **62** (0.091 *vs* 0.235 mM·min^{-1}). Although the initial rates of both glycosylation reactions have

zero-order dependence on the acceptors, it is less likely that these glycosylation proceeds through S_N1-type pathways.

c. Preliminary Kinetic Studies for Model Reaction C

Furthermore, we studied the kinetics of this glycosylation with secondary acceptor **66**.

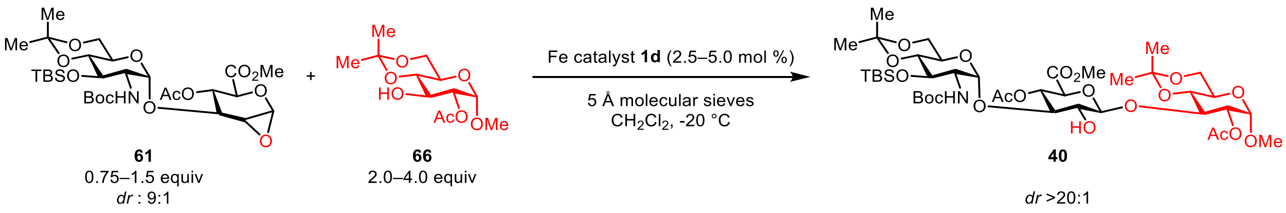

To a flame-dried sealable 2-dram vial (vial A) equipped with a stir bar were added a glycosyl acceptor **66** (66–132 mg, 0.24–0.48 mmol, 2.0–4.0 equiv), Fe(OEP)Cl (2.3–4.6 mg, 0.0036–0.0072 mmol, 3–6 mol %), AgOTf (0.77–1.54 mg, 0.003–0.006 mmol, 2.5–5.0 mol %), and freshly activated 5 Å molecular sieves, powder (*ca.* 100 mg). After the vial was evacuated and backfilled with N_2 three times, anhydrous CH_2Cl_2 (0.6 mL) was added, and the solution was stirred at 0 °C for 10 min before cooled to -78 °C. To a second flame-dried sealable 2-dram vial (vial **B**) was added epoxide **61** (78–156 mg, 0.12–0.24 mmol, 1.0–2.0 equiv)**.** Vial **B** was evacuated and backfilled with N_2 three times, and then anhydrous CH_2Cl_2 (0.6 mL) was added, and epoxide **61** was transferred to vial A at -78 °C dropwise within 1 min. The reaction was kept at -78 °C for an additional 3 min before being transferred to -20 °C. After 3–50 min, an aliquot (0.05 mL of reaction mixture) was taken and quenched by MeOH (5 μL) and imidazole (0.2 mL, 0.125 M in CH_2Cl_2) at the same temperature. The mixture was filtered through a pad of silica gel and Na_2SO_4 and eluted with EtOAc (1 mL × 3). The filtrate was then concentrated *in vacuo*. The product distribution in the residue was determined through quantitative NMR analysis.

We first aimed to determine the initial rate order dependence on iron catalyst **1d**. In these experiments, the initial concentration of glycal epoxide **61** is 0.1 M, and the initial concentration

of primary glycosyl acceptor **66** is 0.2 M. We measured the initial rates with iron catalyst loadings ranging from 2.5 mol %, 3.75 mol %, to 5.0 mol % (Figure S12).

Based on the concentration increase of glycosylation product **40**, the corresponding initial rates are 0.400 mM·min^{-1}, 0.626 mM·min^{-1}, and 0.795 mM·min^{-1}, respectively.

*These data suggest that the initial rate has a first-order dependence on iron catalyst **1d**.*

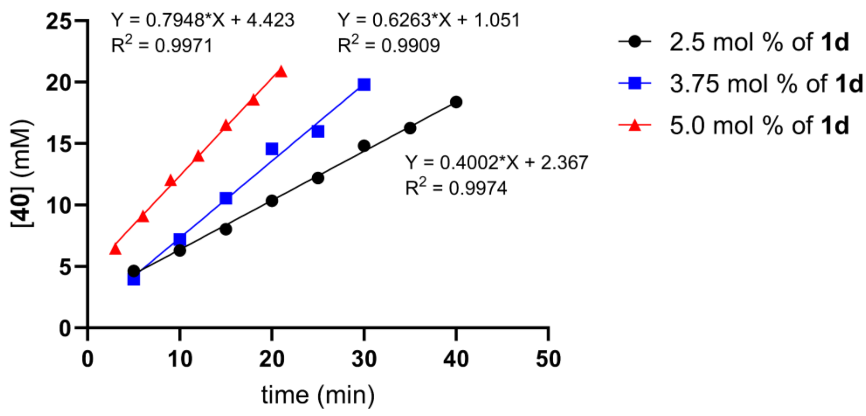

Figure **S12**. Initial Rate Measurement with Different Iron Catalyst Loadings. Measurement was based on the concentration increase of the glycosylation product **40**. Reactions were performed on a 0.12 mmol scale with epoxide **61** (1.0 equiv), glycosyl acceptor **66** (2.0 equiv) in CH_2Cl_2 with 5 Å molecular sieves at -20 °C.

We next evaluated the initial rate order dependence on glycal epoxide **61**. In these experiments, the initial concentration of glycosyl acceptor **66** is 0.2 M, and the initial concentration of iron catalyst **1d** is 0.0025 M. We measured the initial rates when the concentration of epoxide **61** varies from 0.075 M, 0.1 M to 0.15 M (Figure S13).

Based on the concentration increase of glycosylation product **40**, the corresponding initial rates are 0.300 mM·min^{-1}, 0.400 mM·min^{-1}, and 0.624 mM·min^{-1}, respectively.

*These data suggest that the initial rate has a first-order dependence on glycal epoxide **61**.*

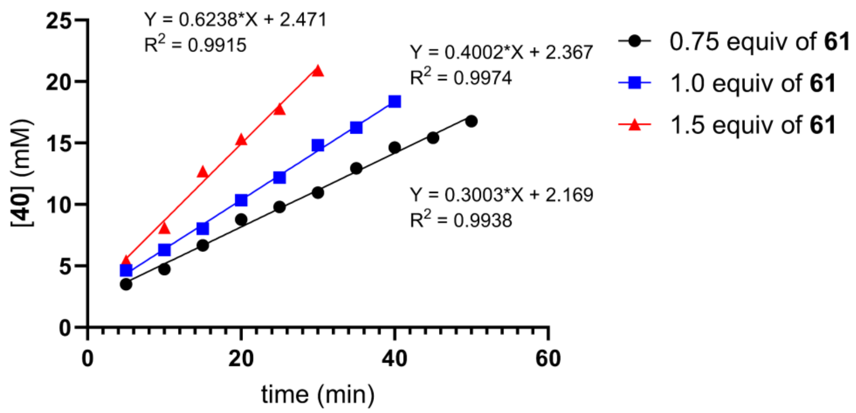

Figure S13. Initial Rate Measurement with Different Glycal Epoxide Concentrations. Measurement was based on concentration increase of the glycosylation product **40**. Reactions were performed on a 0.12 mmol scale with glycosyl acceptor **66** (2.0 equiv) and iron catalyst **1d** (2.5 mol %) in CH_2Cl_2 with 5 Å molecular sieves at -20 °C.

We further evaluated the initial rate order dependence on secondary glycosyl acceptor **66**. In these experiments, the initial concentration of epoxide **61** is 0.1 M, and the initial concentration of iron catalyst **1d** is 0.0025 M. We measured the initial rates with the secondary glycosyl acceptor **66** ranging from 2.0 equiv, 3.0 equiv, to 4.0 equiv (Figure S14).

Based on the concentration increase of glycosylation product **40**, the corresponding initial rates are 0.400 mM·min^{-1}, 0.608 mM·min^{-1}, and 0.785 mM·min^{-1}, respectively.

*These data suggested that the initial rate has a first-order dependence on secondary acceptor **66**.*

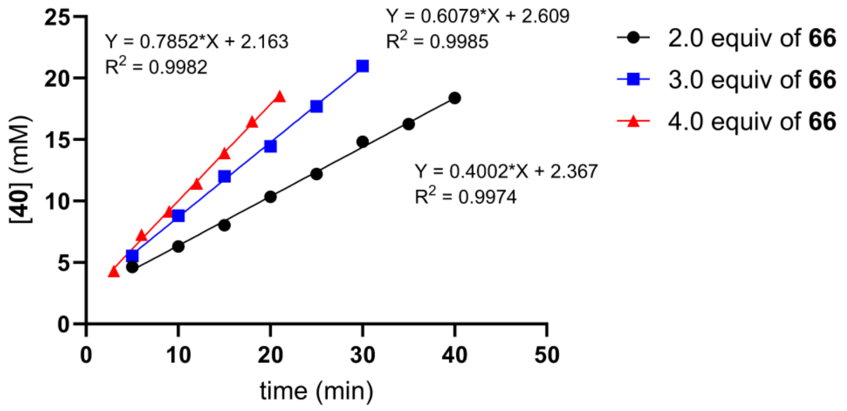

Figure S14. Initial Rate Measurement with Different Glycosyl Acceptor Concentrations. Measurement was based on concentration increase of the glycosylation product **40**. Reactions were performed on a 0.12 mmol scale with glycal epoxide **61** (1.0 equiv) and iron catalyst **1d** (2.5 mol %) in CH_2Cl_2 with 5 Å molecular sieves at -20 °C.

These preliminary kinetic studies suggested that the initial rate of this glycosylation with secondary acceptor **66** has a *first-order* dependence each on the iron catalyst, glycal epoxide **1d**, and glycosyl acceptor **66**.

The *zero-order* rate dependence on a primary acceptor is particularly intriguing, as it implies two possible mechanistic scenarios. First, the glycosylation might proceed through an S_N1-type pathway with a primary acceptor yet through an S_N2-type pathway with a secondary acceptor. Alternatively, both glycosylations might occur via S_N2-type pathways, but the primary acceptor could strongly coordinate with the iron catalyst, such that the concentration of the active iron catalyst has an inverse first-order dependence on the primary acceptor (Figure S15). Therefore, the overall zero-order rate dependence on a primary acceptor was observed.

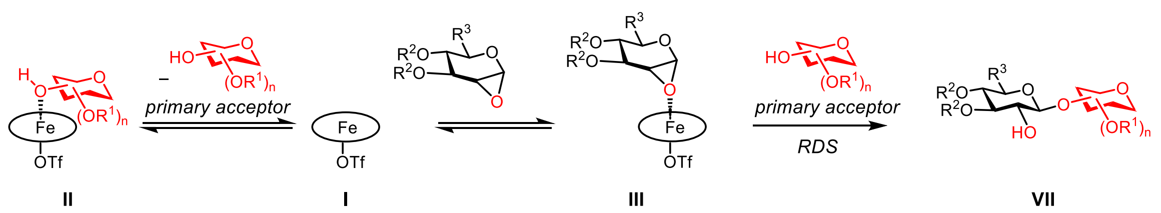

Figure S15. Mechanistic Working Hypothesis for Glycosylation with Primary Acceptors.

d. Competition Glycosylation Kinetics Between a Primary and a Secondary Glycosyl Acceptor

To differentiate these two mechanistic possibilities, we measured the glycosylation rates of both primary and secondary acceptor **64** and **66** (1:1) in competition with donor **61**.

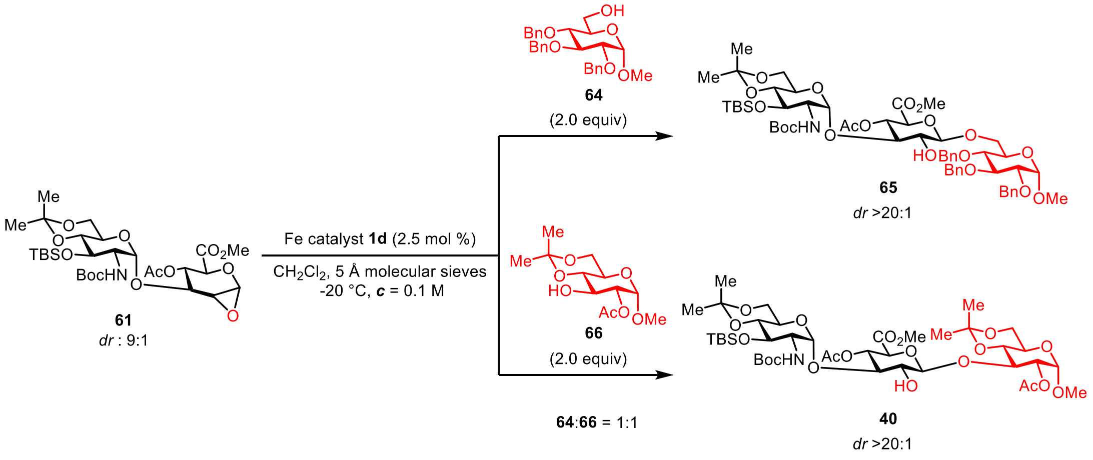

To a flame-dried sealable 2-dram vial (vial A) equipped with a stir bar were added primary glycosyl acceptor **64** (112 mg, 0.24 mmol, 2.0 equiv) and secondary glycosyl acceptor **66** (66 mg, 0.24 mmol, 2.0 equiv), Fe(OEP)Cl (2.3 mg, 0.0036 mmol, 3 mol %), AgOTf (0.77 mg, 0.003 mmol, 2.5 mol %), and freshly activated 5 Å molecular sieves, powder (*ca.* 100 mg). After the vial was evacuated and backfilled with N_2 three times, anhydrous CH_2Cl_2 (0.6 mL) was added, and the solution was stirred at 0 °C for 10 min before cooled to -78 °C. To a second flame-dried sealable 2-dram vial (vial **B**) was added glycal epoxide **61** (78 mg, 0.12 mmol, 1.0 equiv). Vial **B** was evacuated and backfilled with N_2 three times, and then anhydrous CH_2Cl_2 (0.6 mL) was added, and epoxide **61** was transferred to vial A at -78 °C dropwise within 1 min. The reaction was kept at -78 °C for an additional 3 min before being transferred to -20 °C. After 10–100 min, an aliquot (0.05 mL of reaction mixture) was taken and quenched by MeOH (5 μL) and imidazole (0.2 mL, 0.125 M in CH_2Cl_2) at the same temperature. The mixture was filtered through a pad of silica gel and Na_2SO_4 and eluted with EtOAc (1 mL × 3). The filtrate was then

concentrated *in vacuo*. The product distribution in the residue was determined through quantitative NMR analysis.

We aimed to determine the initial rate dependence on both primary acceptor **64** and secondary acceptor **66**. In these experiments, the initial concentration of glycal epoxide **61** is 0.1 M, the initial concentration of iron catalyst **1d** is 0.0025 M, and the initial concentrations of both glycosyl acceptors are 0.2 M (Figure S16).

Based on the concentration increase of glycosylation products **65** and **40**, the corresponding initial rates are 0.0835 mM·min^{-1} and 0.0881 mM·min^{-1}, respectively.

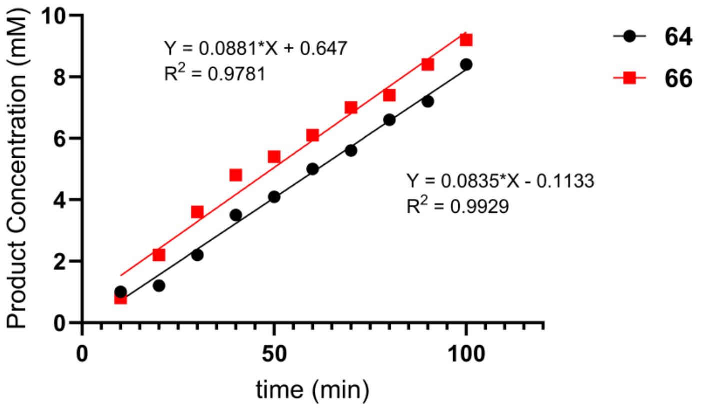

Figure S16. Competition Glycosylation Kinetics between a Primary and a Secondary Glycosyl Acceptor. Reactions were performed on a 0.12 mmol scale with glycal epoxide **61** (1.0 equiv), glycosyl acceptors **64** (2.0 equiv), acceptors **66** (2.0 equiv), and iron catalyst **1d** (2.5 mol %) in CH_2Cl_2 with 5 Å molecular sieves at -20 °C.

The initial rate with secondary acceptor **66** drops significantly (21% of its original rate), but the one with primary acceptor **64** remains more or less the same. These results suggested that the primary acceptor indeed coordinates with the catalyst and effectively decreases the concentration of active iron catalyst available for glycosylation.

F. References

1. Li, H.; Zhang, D.; Li, C.; Yin, L.; Jiang, Z.; Luo, Y.; Xu, H. Stereoselective Glycosylation for 1,2-*cis*-Aminoglycoside Assembly by Cooperative Atom Transfer Catalysis. *J. Am. Chem. Soc.* **2024**, *146*, 33316.

2. Taber, D. F.; DeMatteo, P. W.; Hassan, R. A. Simplified Preparation of Dimethyldioxirane (DMDO). In *Organic Syntheses*, pp 350-357.

3. Eby, R.; Srivastava, V. K. Conformational Analysis of 1,2-Anhydro-3,4,6-tri-*O*-Benzyl-α-D-Glucopyranose and -β-D-Mannopyranose. *Carbohydr. Res.* **1982**, *102*, 1.

4. Allen, C. L.; Miller, S. J. Chiral Copper(II) Complex-Catalyzed Reactions of Partially Protected Carbohydrates. *Org. Lett.* **2013**, *15*, 6178.

5. Schell, P.; Orgueira, H. A.; Roehrig, S.; Seeberger, P. H. Synthesis and Transformations of D-Glucuronic and L-Iduronic Acid Glycals. *Tetrahedron Lett.* **2001**, *42*, 3811.

6. Balmond, E. I.; Coe, D. M.; Galan, M. C.; McGarrigle, E. M. α-Selective Organocatalytic Synthesis of 2-Deoxygalactosides. *Angew. Chem. Int. Ed.* **2012**, *51*, 9152.

7. Yoshida, K.; Fujino, Y.; Itatsu, Y.; Inoue, H.; Kanoko, Y.; Takao, K.-i. Amine-Free Silylation of Alcohols under 4-Methylpyridine *N*-Oxide-Catalyzed Conditions. *Tetrahedron Lett.* **2016**, *57*, 627.

8. Filice, M.; Guisan, J. M.; Terreni, M.; Palomo, J. M. Regioselective Monodeprotection of Peracetylated Carbohydrates. *Nat. Protoc.* **2012**, *7*, 1783.

9. Nicolaou, K. C.; Rodríguez, R. M.; Mitchell, H. J.; Suzuki, H.; Fylaktakidou, K. C.; Baudoin, O.; van Delft, F. L. Total Synthesis of Everninomicin 13,384-1—Part 1: Retrosynthetic Analysis and Synthesis of the $A_1B(A)C$ Fragment. *Chem. Eur. J.* **2000**, *6*, 3095.

10. Blackburne, I.; Fredericks, P.; Guthrie, R. Studies on Unsaturated Sugars with Particular Reference to the Synthesis of 6-Deoxy-6-Fluoro Derivatives. *Aust. J. Chem.* **1976**, *29*, 381.

11. Griffith, D. A.; Danishefsky, S. J. Total Synthesis of Allosamidin: An Application of the Sulfonamidoglycosylation of Glycals. *J. Am. Chem. Soc.* **1991**, *113*, 5863.

12. Postema, M. H. D.; TenDyke, K.; Cutter, J.; Kuznetsov, G.; Xu, Q. Total Synthesis of Ipomoeassin F. *Org. Lett.* **2009**, *11*, 1417.

13. Meng, S.; Zhong, W.; Yao, W.; Li, Z. Stereoselective Phenylselenoglycosylation of Glycals Bearing a Fused Carbonate Moiety toward the Synthesis of 2-Deoxy-β-galactosides and β-Mannosides. *Org. Lett.* **2020**, *22*, 2981.

14. Paquette, L. A.; Oplinger, J. A. Synthesis of a Structurally Modified Glycal. (-)-(2*R*,4*S*)-2-Methyl-2-Vinyl-4-(Benzyloxy)-3,4-Dihydro-2*H*-Pyran. *J. Org. Chem.* **1988**, *53*, 2953.

15. Kalikanda, J.; Li, Z. Study of the Stereoselectivity of 2-Azido-2-Deoxygalactosyl Donors: Relationship to the Steric Factors of Glycosyl Acceptors. *Carbohydr. Res.* **2011**, *346*, 2380.

16. Evans, M. E.; Parrish, F. W. Monomolar Acetalations of Methyl α-D-Mannosides—Synthesis of Methyl α-D-Talopyranoside. *Carbohydr. Res.* **1977**, *54*, 105.

17. Marshall, J. A.; Beaudoin, S. Stereoselective Synthesis of Differentially Protected Derivatives of the Higher Amino Sugars Destomic Acid and Lincosamine from Serine and Threonine. *J. Org. Chem.* **1996**, *61*, 581.

18. Pehere, A. D.; Abell, A. D. New β-Strand Templates Constrained by Huisgen Cycloaddition. *Org. Lett.* **2012**, *14*, 1330.

19. Brik, A.; Yang, Y.-Y.; Ficht, S.; Wong, C.-H. Sugar-Assisted Glycopeptide Ligation. *J. Am. Chem. Soc.* **2006**, *128*, 5626.

20. Anžiček, N.; Williams, S.; Housden, M. P.; Paterson, I. Toward Aplyronine Payloads for Antibody–Drug Conjugates: Total Synthesis of Aplyronines A and D. *Org. Biomol. Chem.* **2018**, *16*, 1343.

G. NMR Spectra

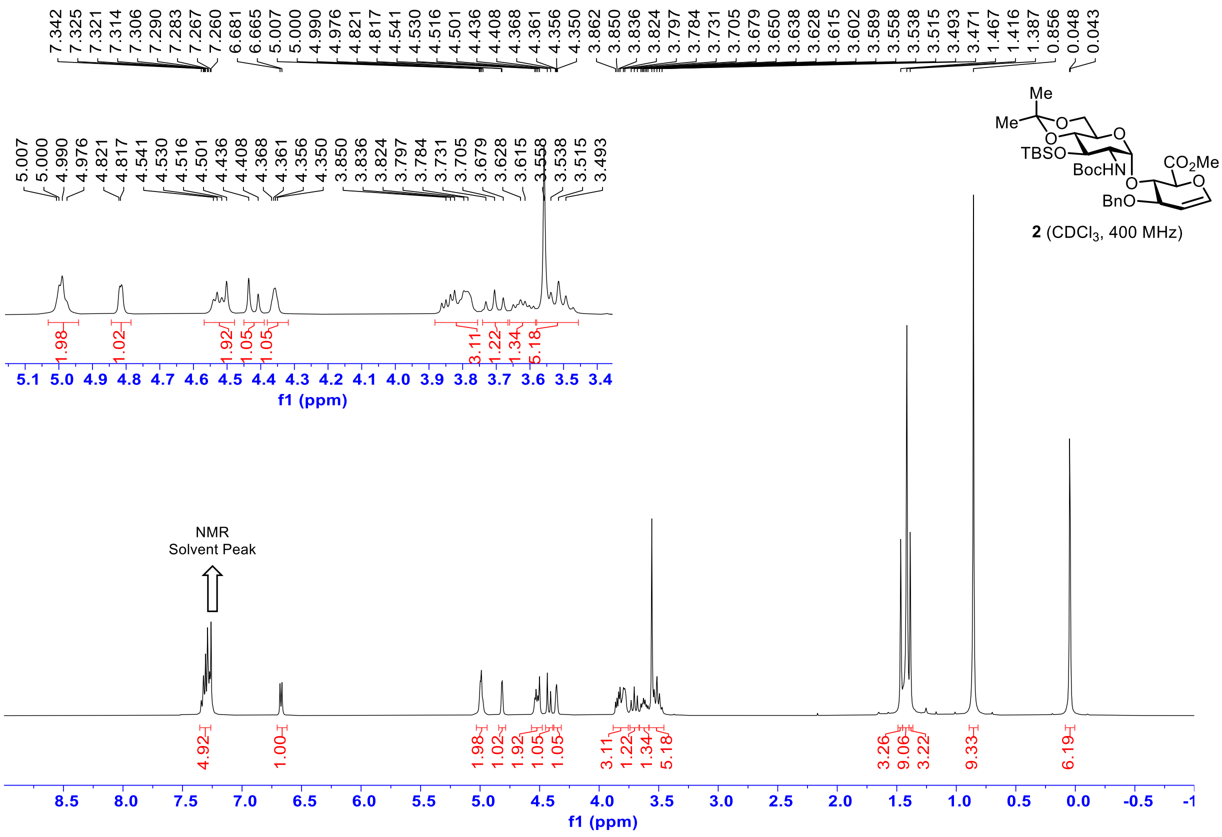

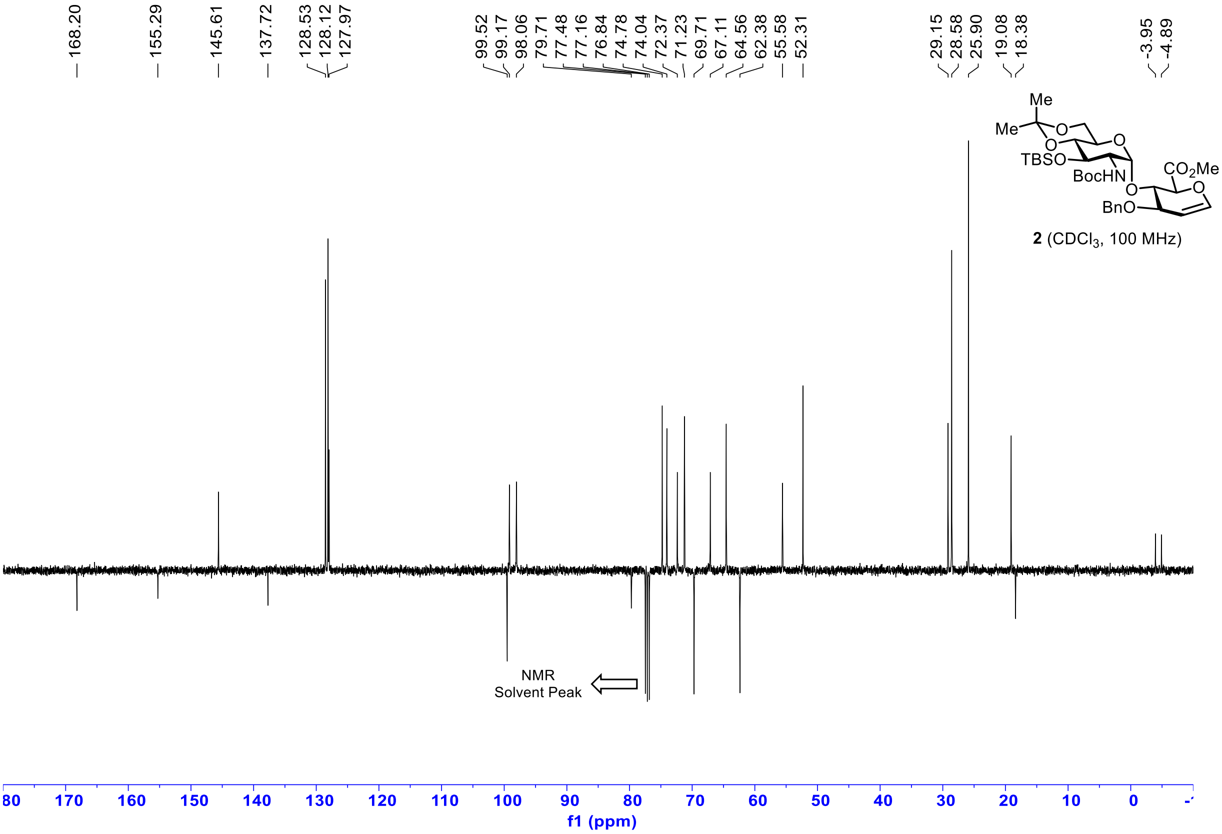

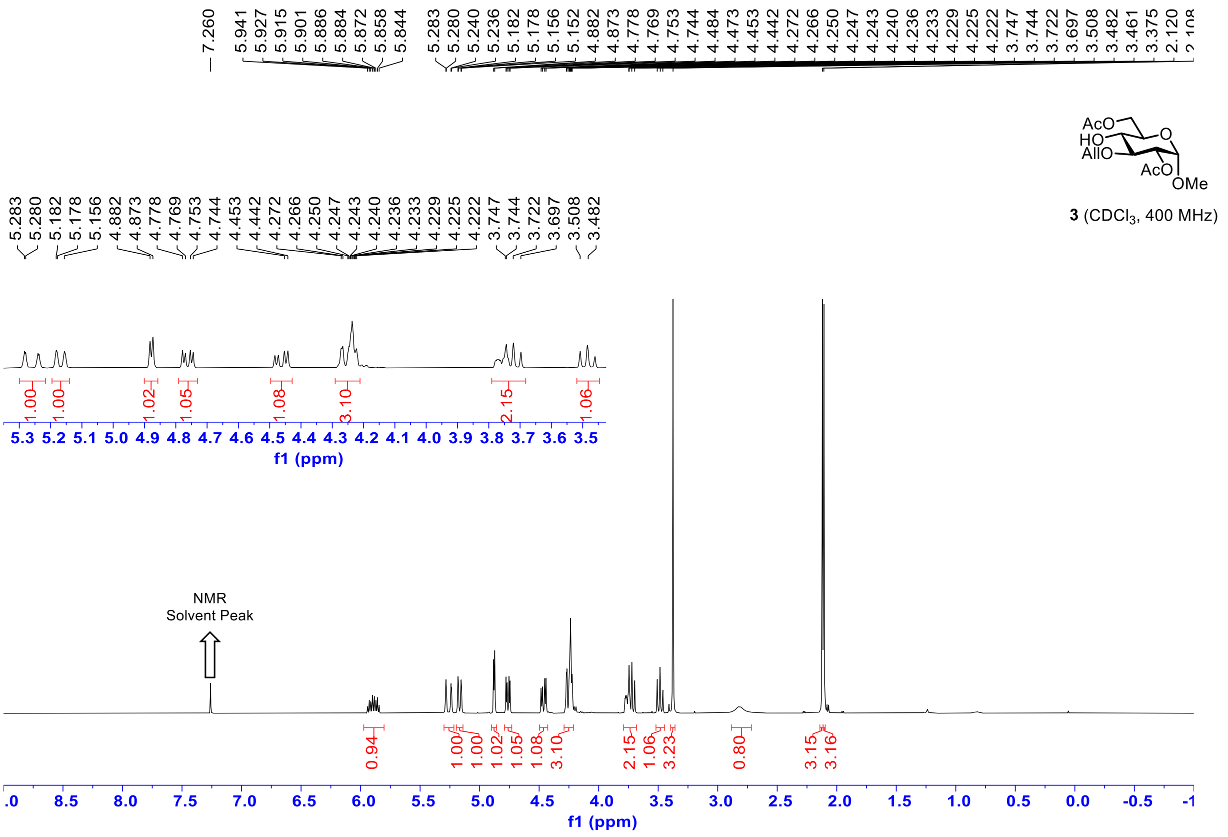
7.260
5.941 5.927 5.915 5.901 5.886 5.884 5.872 5.858 5.844
5.283 5.280 5.240 5.236 5.182 5.178 5.156 5.152 4.882 4.873 4.778 4.769 4.753 4.744 4.484 4.473 4.453 4.442 4.272 4.266 4.250 4.247 4.243 4.240 4.236 4.233 4.229 4.225 4.222 3.747 3.744 3.722 3.697 3.508 3.482 3.461 3.375 2.120
AcO
HO
AllO
AcO
OMe
3 (CDCl3, 400 MHz)
NMR
Solvent Peak
f1 (ppm)

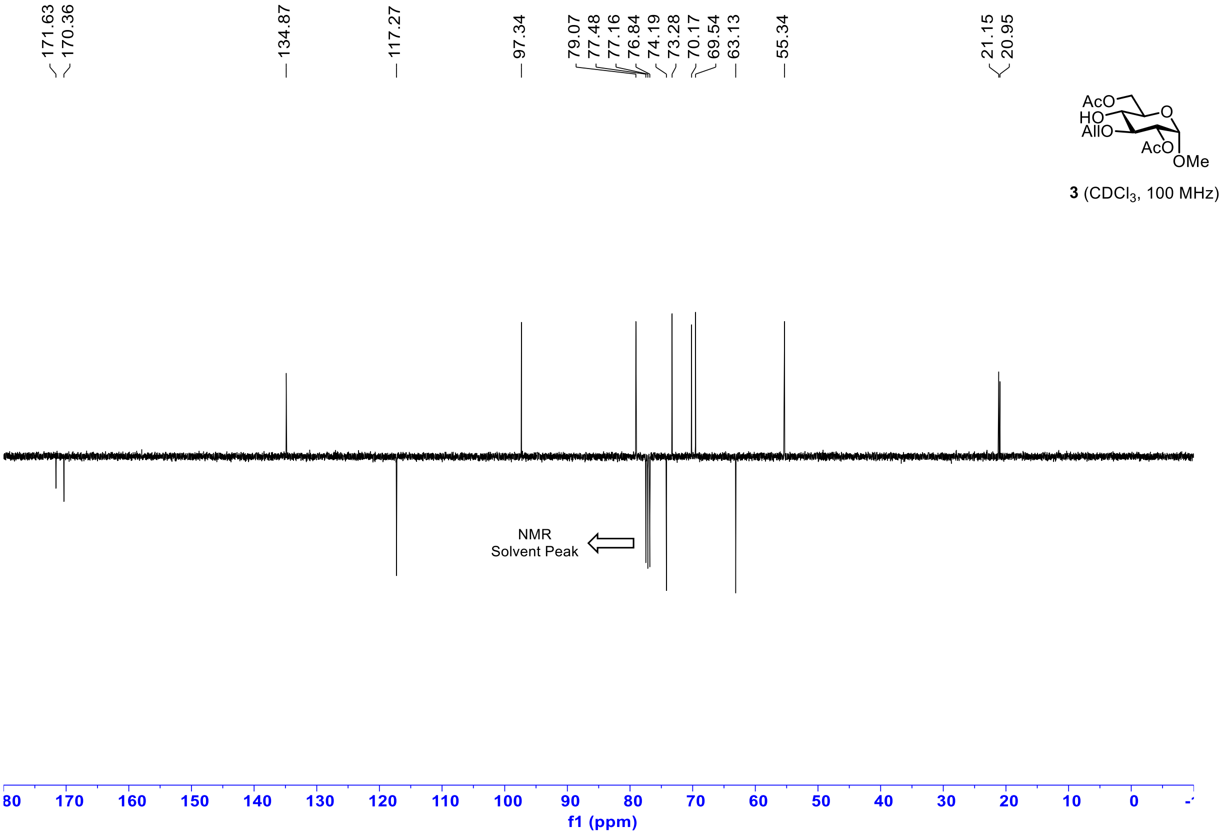
171.63 170.36
134.87
117.27
97.34
79.07 77.48 77.16 76.84 74.19 73.28 70.17 69.54 63.13
55.34
21.15 20.95
AcO
HO
AllO
AcO
OMe
3 (CDCl3, 100 MHz)
NMR
Solvent Peak
f1 (ppm)

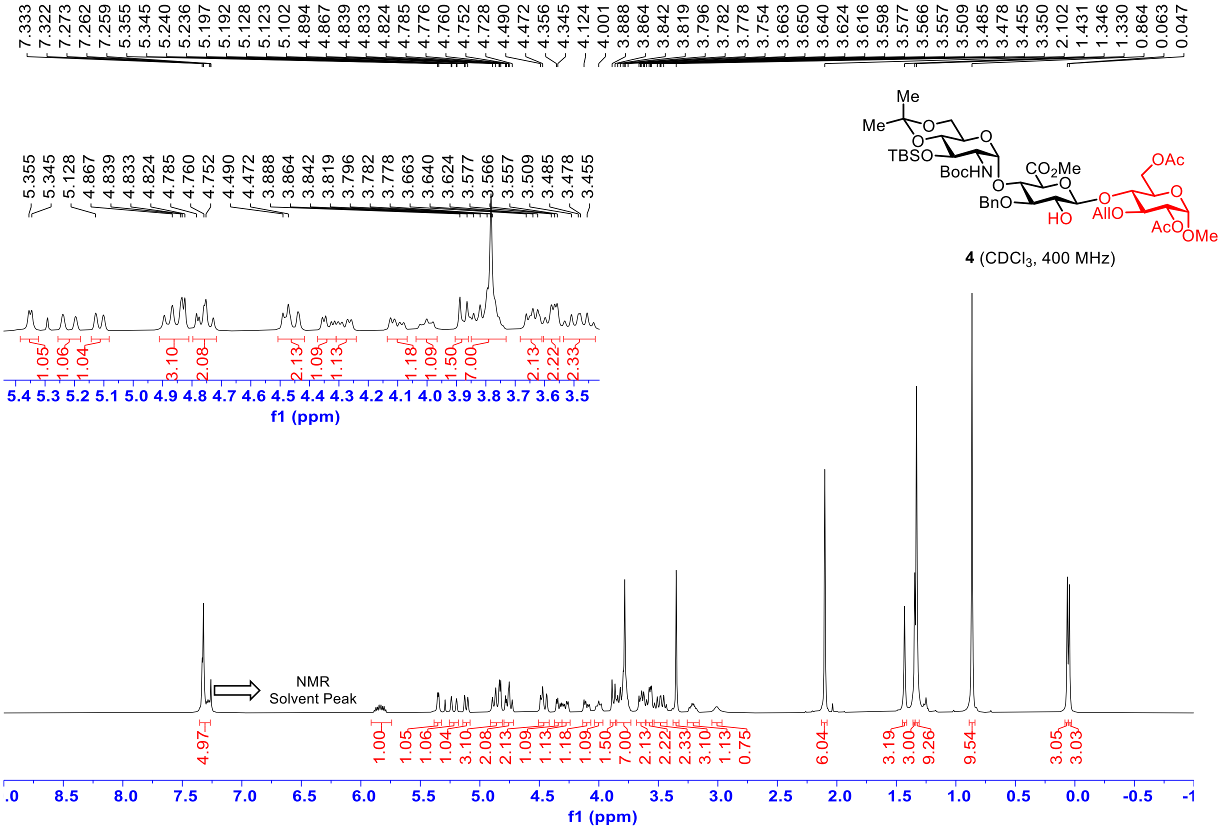
4 (CDCl$_3$, 400 MHz)
NMR Solvent Peak
f1 (ppm)

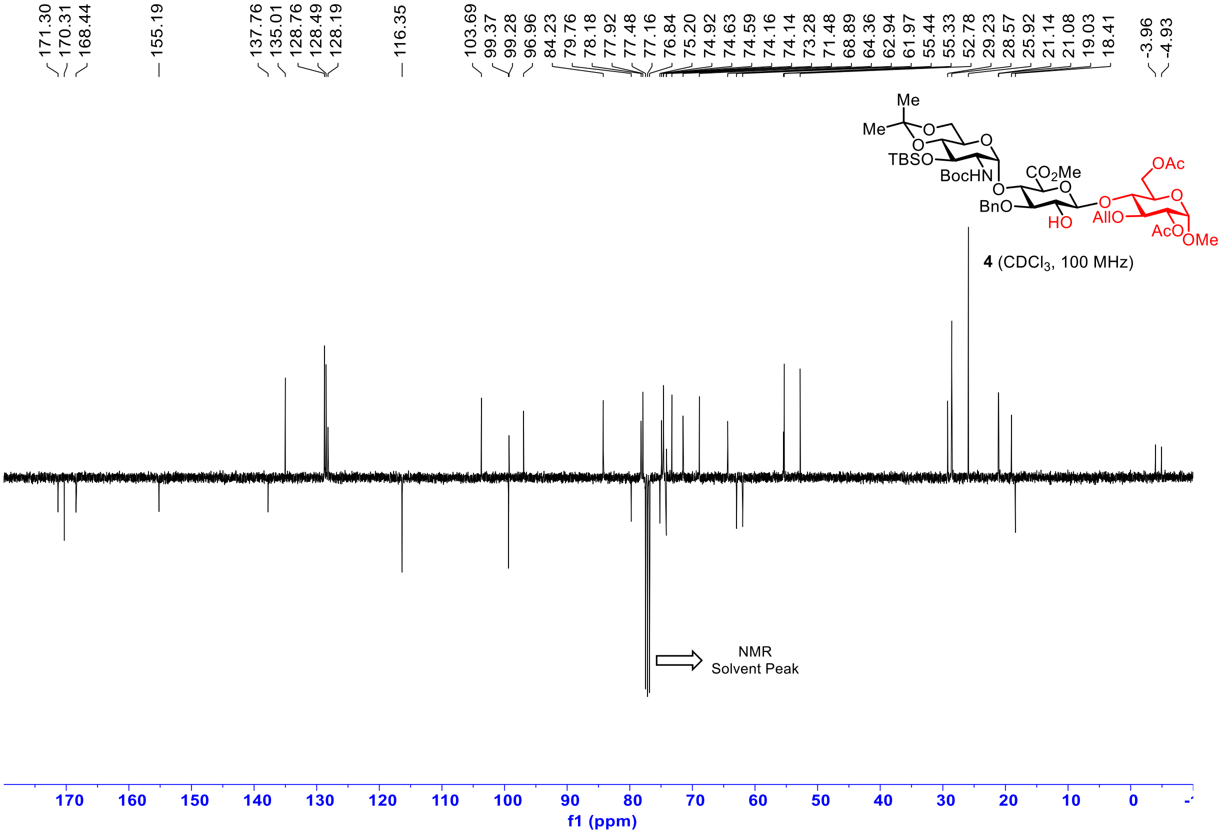
4 (CDCl$_3$, 100 MHz)
NMR Solvent Peak
f1 (ppm)

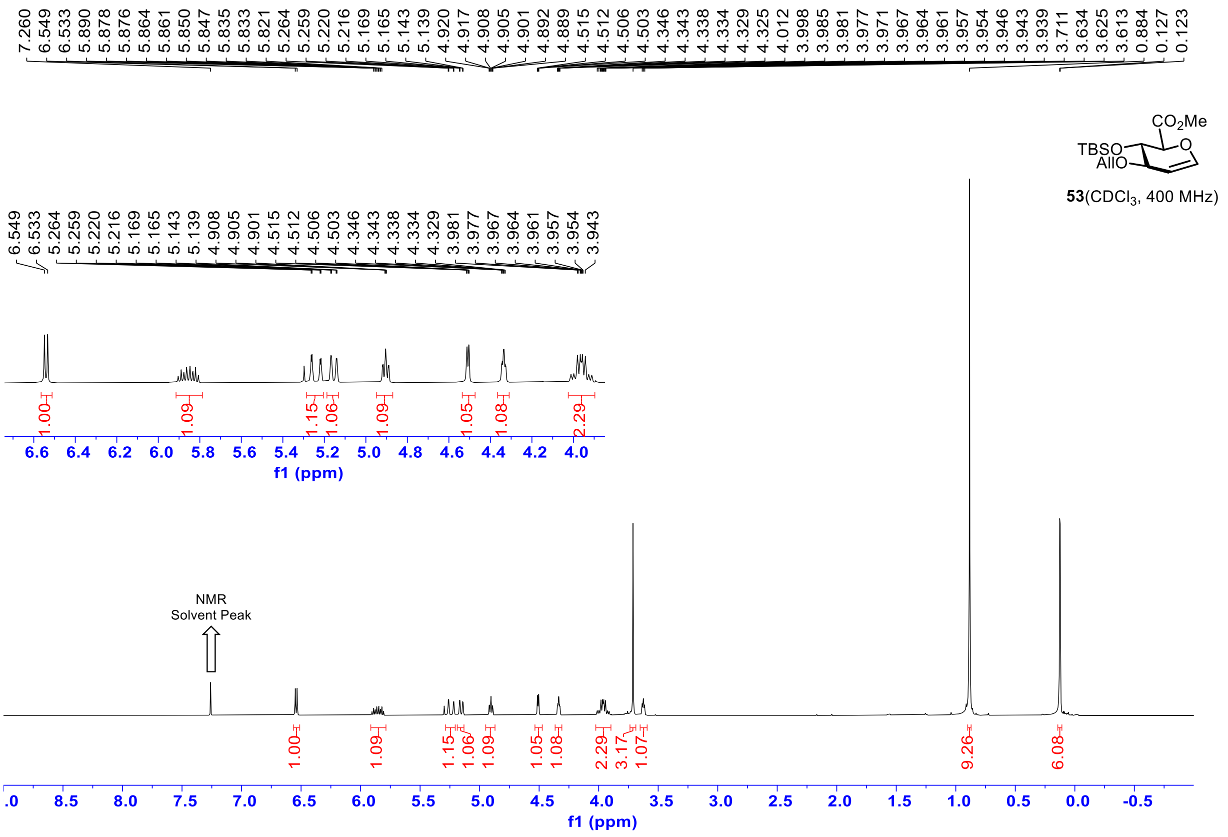
7.260 6.549 6.533 5.890 5.878 5.876 5.864 5.861 5.850 5.847 5.835 5.833 5.821 5.264 5.259 5.220 5.216 5.169 5.165 5.143 5.139 4.920 4.917 4.908 4.905 4.901 4.892 4.889 4.515 4.512 4.506 4.503 4.346 4.343 4.338 4.334 4.329 4.325 4.012 3.998 3.985 3.981 3.977 3.971 3.967 3.964 3.961 3.957 3.954 3.946 3.943 3.939 3.711 3.634 3.625 3.613 0.884 0.127 0.123
CO2Me
TBSO
AllO
O
53(CDCl3, 400 MHz)
NMR
Solvent Peak
f1 (ppm)

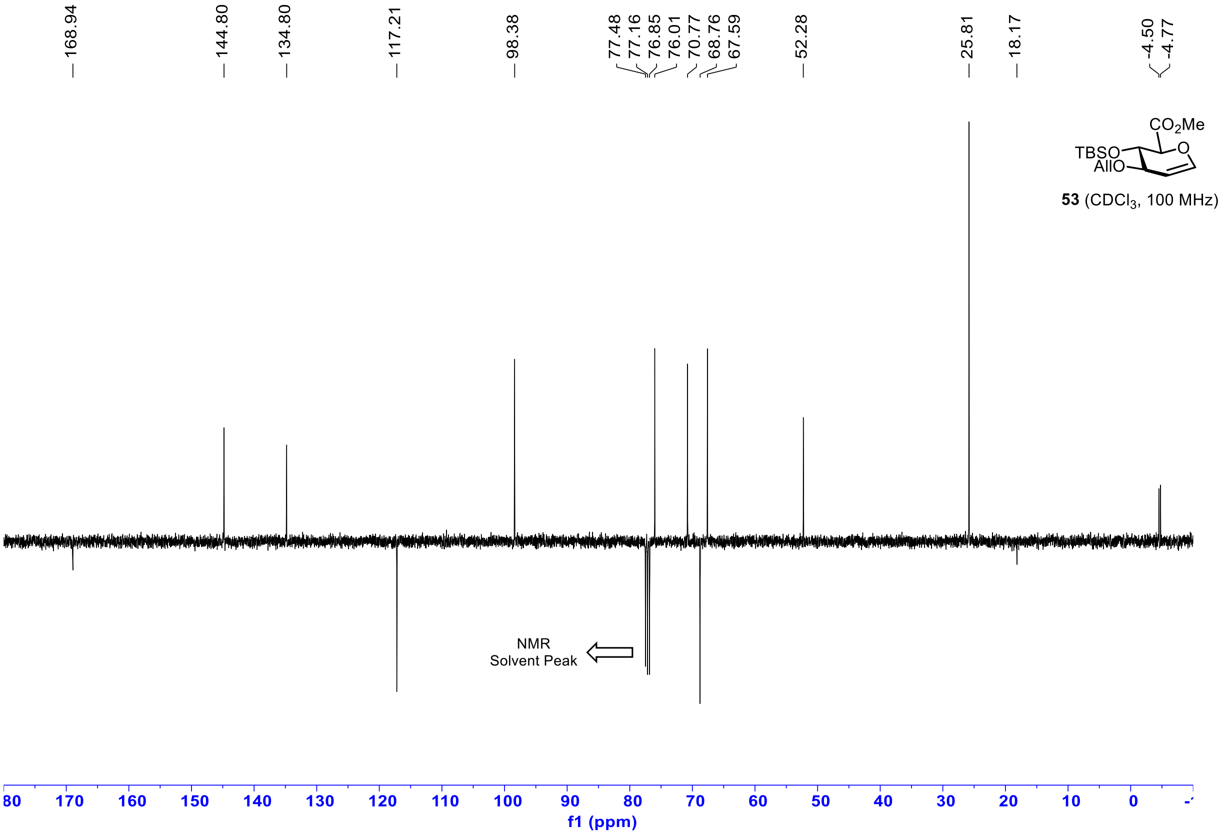
168.94 144.80 134.80 117.21 98.38 77.48 77.16 76.85 76.01 70.77 68.76 67.59 52.28 25.81 18.17 -4.50 -4.77
CO2Me
TBSO
AllO
O
53 (CDCl3, 100 MHz)
NMR
Solvent Peak
f1 (ppm)

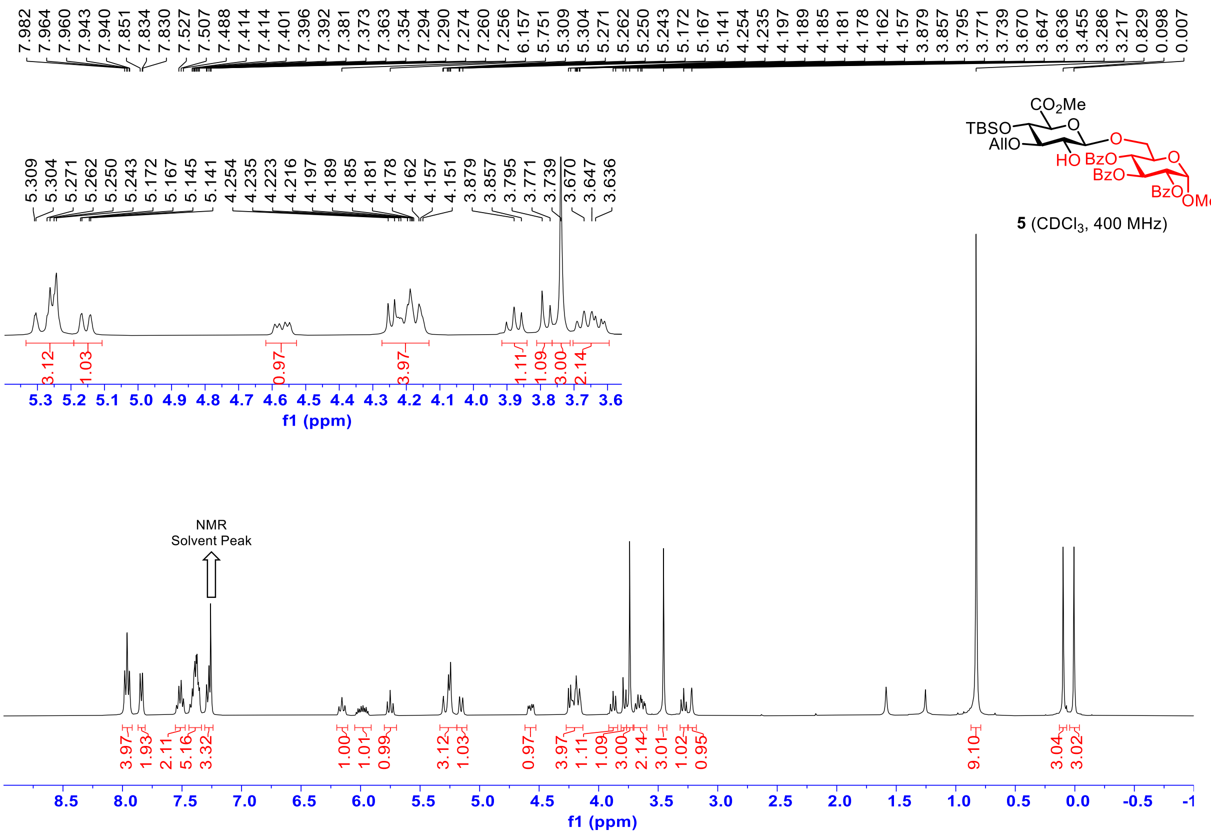
7.982 7.964 7.960 7.943 7.940 7.851 7.834 7.830 7.527 7.507 7.488 7.414 7.414 7.401 7.396 7.392 7.381 7.373 7.363 7.354 7.294 7.290 7.274 7.260 7.256 6.157 5.751 5.309 5.304 5.271 5.262 5.250 5.243 5.172 5.167 5.141 4.254 4.235 4.197 4.189 4.185 4.181 4.178 4.162 4.157 3.879 3.857 3.795 3.771 3.739 3.670 3.647 3.636 3.455 3.286 3.217 0.829 0.098 0.007
5.309 5.304 5.271 5.262 5.250 5.243 5.172 5.167 5.145 5.141 4.254 4.235 4.223 4.216 4.197 4.189 4.185 4.181 4.178 4.162 4.157 4.151 3.879 3.857 3.795 3.771 3.739 3.670 3.647 3.636
CO2Me
TBSO
AllO
O
HO BzO
BzO
BzO
OMe
5 (CDCl3, 400 MHz)
3.12 1.03 0.97 3.97 1.11 1.09 3.00 2.14
5.3 5.2 5.1 5.0 4.9 4.8 4.7 4.6 4.5 4.4 4.3 4.2 4.1 4.0 3.9 3.8 3.7 3.6
f1 (ppm)
NMR
Solvent Peak
3.97 1.93 2.11 5.16 3.32 1.00 1.01 0.99 3.12 1.03 0.97 3.97 1.11 1.09 3.00 2.14 3.01 1.02 0.95 9.10 3.04 3.02
8.5 8.0 7.5 7.0 6.5 6.0 5.5 5.0 4.5 4.0 3.5 3.0 2.5 2.0 1.5 1.0 0.5 0.0 -0.5 -1
f1 (ppm)

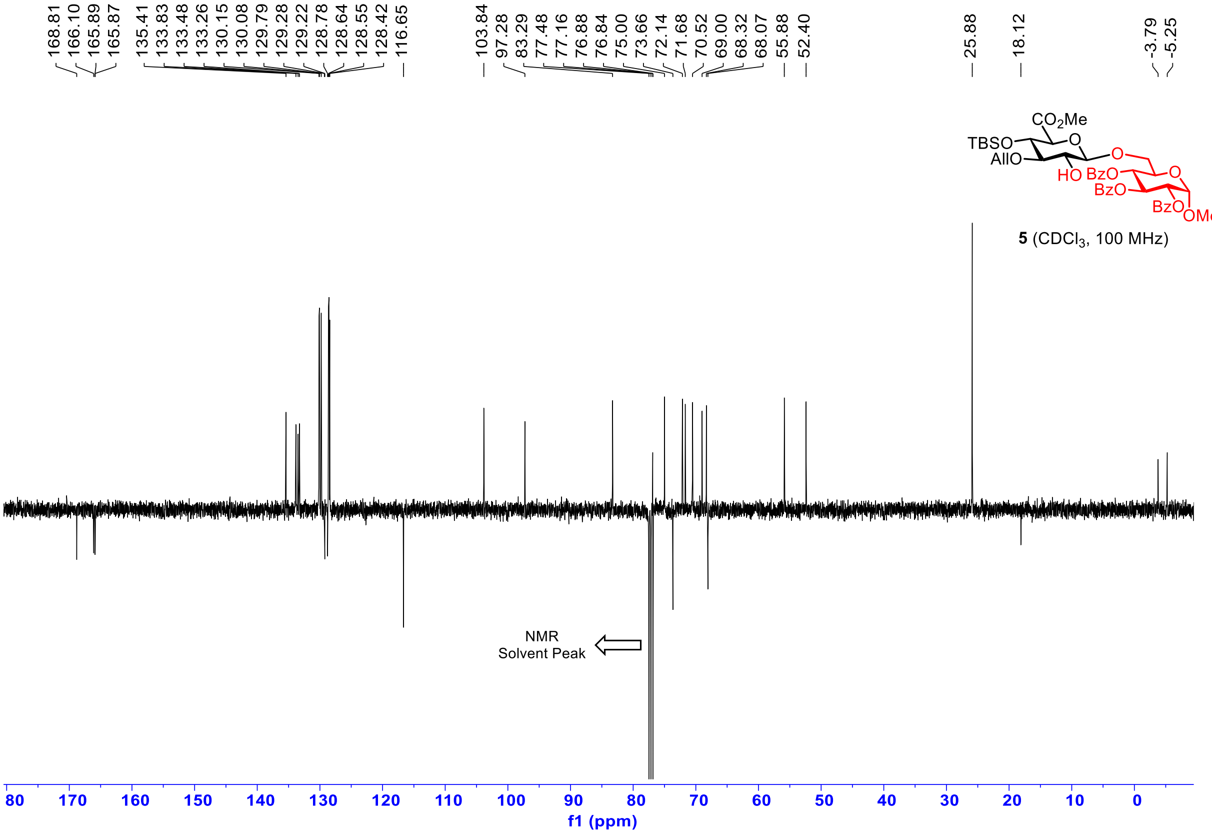
168.81 166.10 165.89 165.87 135.41 133.83 133.48 133.26 130.15 130.08 129.79 129.28 129.22 128.78 128.64 128.55 128.42 116.65 103.84 97.28 83.29 77.48 77.16 76.88 76.84 75.00 73.66 72.14 71.68 70.52 69.00 68.32 68.07 55.88 52.40 25.88 18.12 -3.79 -5.25
CO2Me
TBSO
AllO
O
HO BzO
BzO
BzO
OMe
5 (CDCl3, 100 MHz)
NMR
Solvent Peak
80 170 160 150 140 130 120 110 100 90 80 70 60 50 40 30 20 10 0
f1 (ppm)

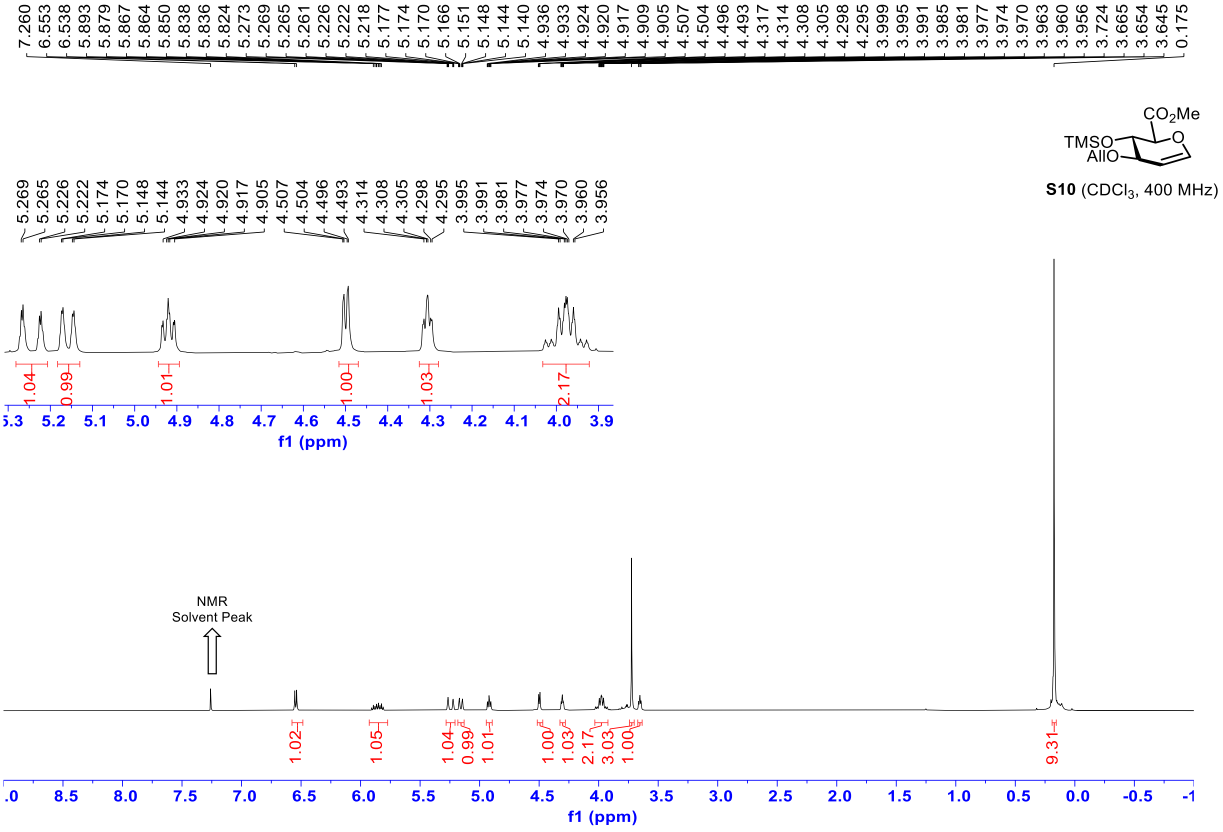
7.260 6.553 6.538 5.893 5.879 5.867 5.864 5.850 5.838 5.836 5.824 5.273 5.269 5.265 5.261 5.226 5.222 5.218 5.177 5.174 5.170 5.166 5.151 5.148 5.144 5.140 4.936 4.933 4.924 4.920 4.917 4.909 4.905 4.507 4.504 4.496 4.493 4.317 4.314 4.308 4.305 4.298 4.295 3.999 3.995 3.991 3.985 3.981 3.977 3.974 3.970 3.963 3.960 3.956 3.724 3.665 3.654 3.645 0.175
5.269 5.265 5.226 5.222 5.174 5.170 5.148 5.144 4.933 4.924 4.920 4.917 4.905 4.507 4.504 4.496 4.493 4.314 4.308 4.305 4.298 4.295 3.995 3.991 3.981 3.977 3.974 3.970 3.960 3.956
CO_2Me
TMSO
AllO
O
S10 ($CDCl_3$, 400 MHz)
1.04 0.99 1.01 1.00 1.03 2.17
f1 (ppm)
NMR Solvent Peak
1.02 1.05 1.04 0.99 1.01 1.00 1.03 2.17 3.03 1.00 9.31
f1 (ppm)

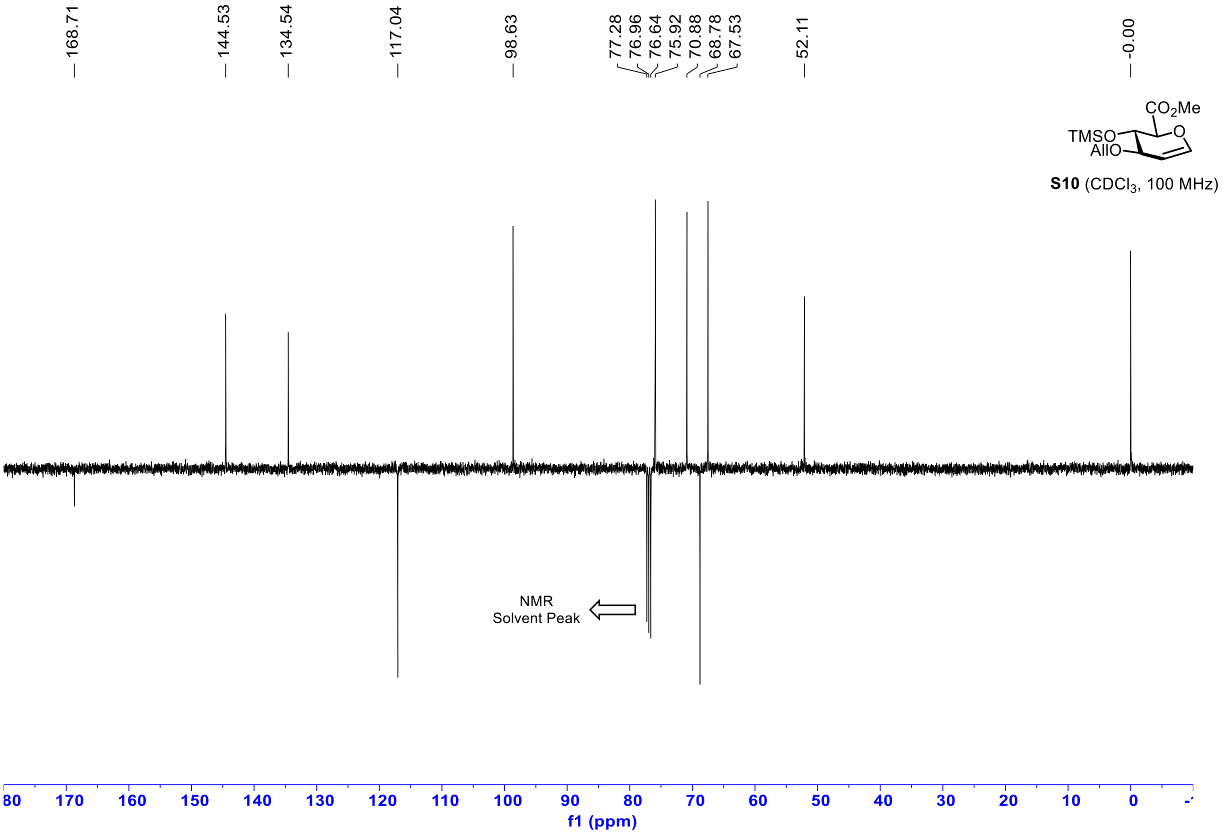
168.71 144.53 134.54 117.04 98.63 77.28 76.96 76.64 75.92 70.88 68.78 67.53 52.11 -0.00
CO_2Me
TMSO
AllO
O
S10 ($CDCl_3$, 100 MHz)
NMR Solvent Peak
f1 (ppm)

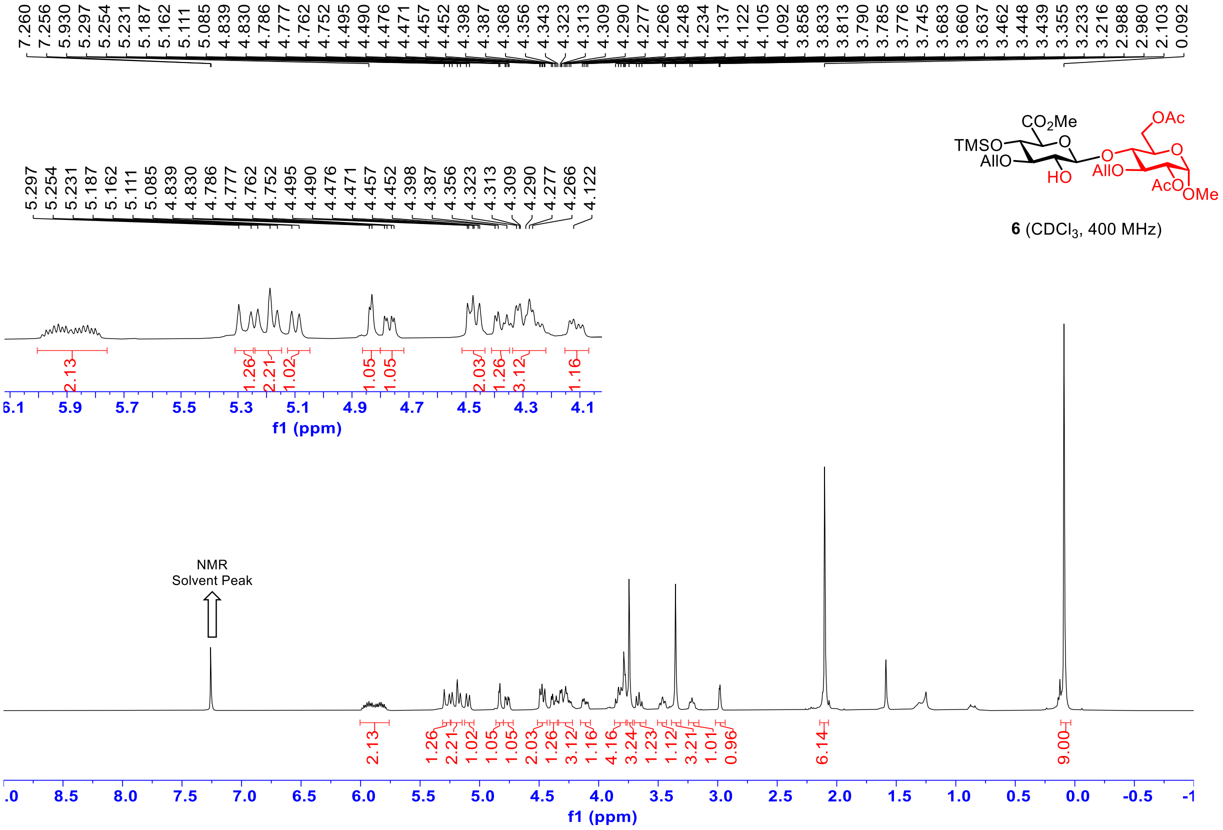

6 ($CDCl_3$, 400 MHz)

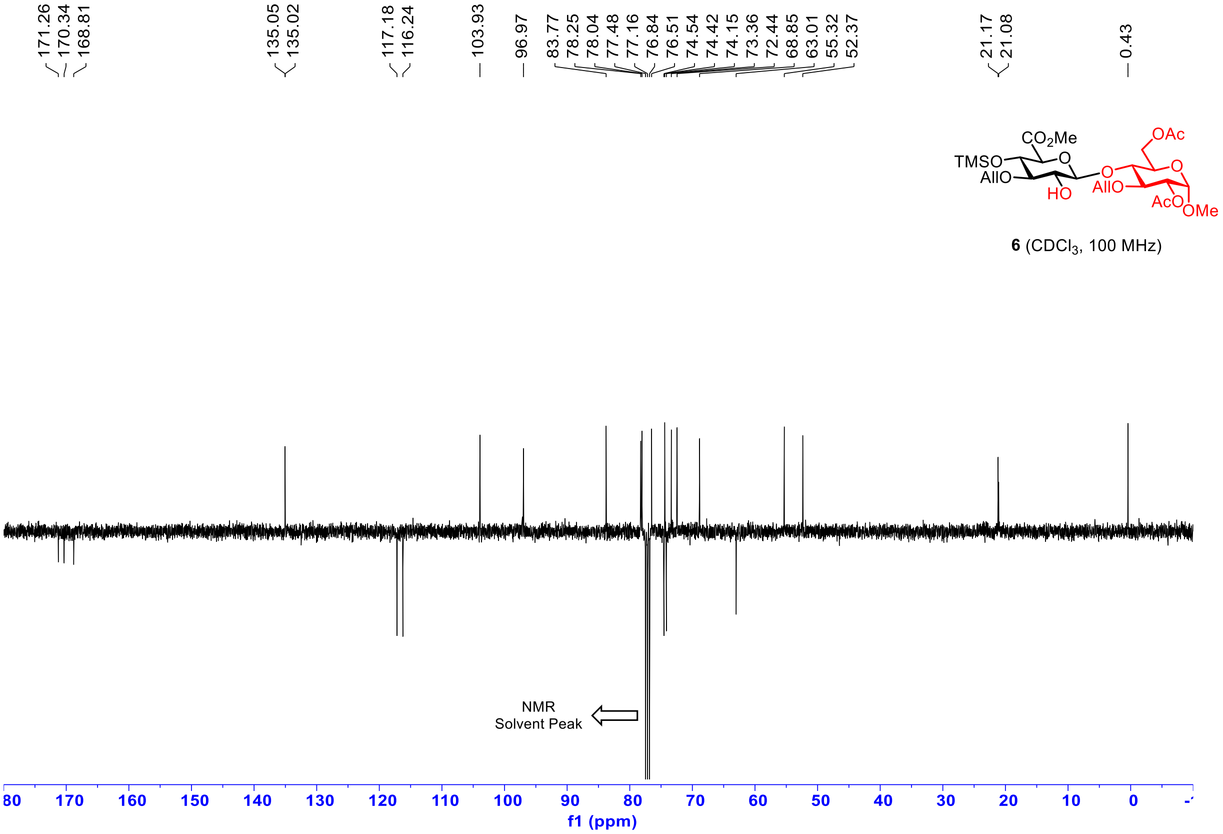

6 ($CDCl_3$, 100 MHz)

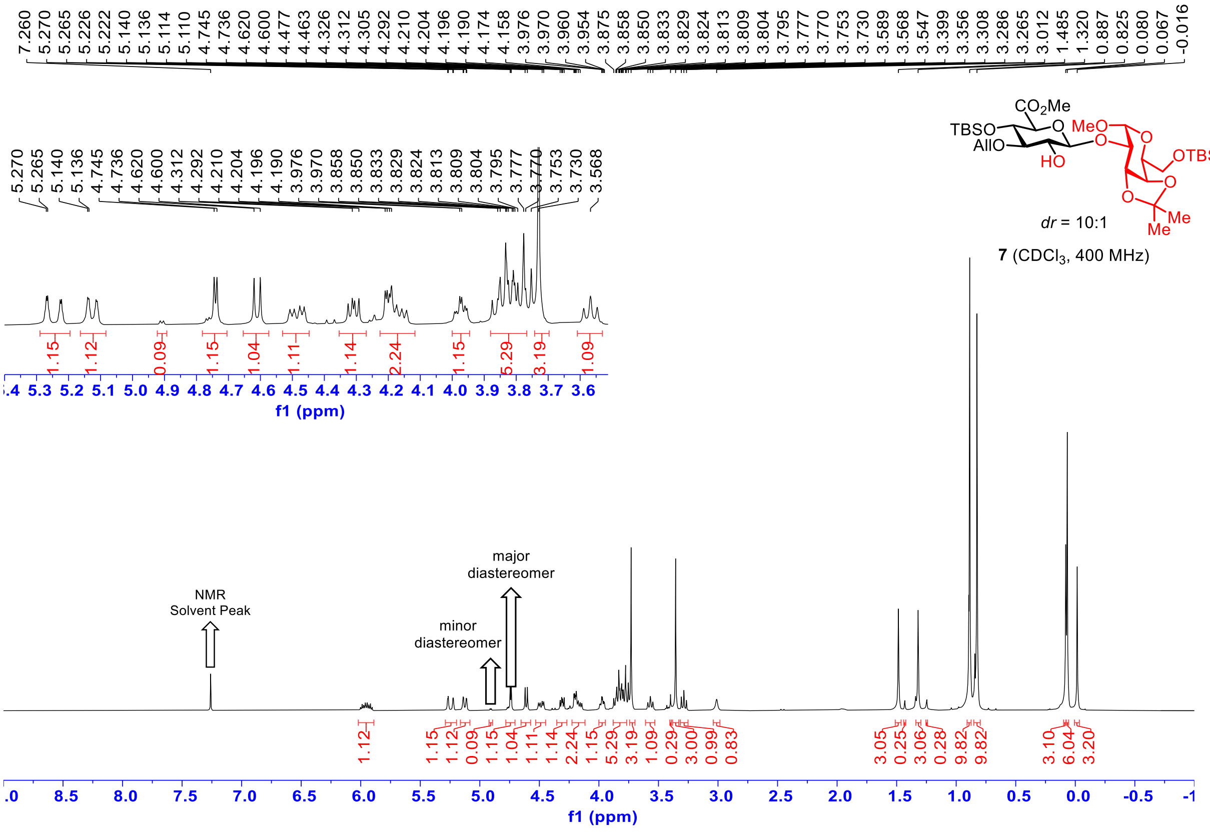

dr = 10:1
7 (CDCl3, 400 MHz)
NMR Solvent Peak
minor diastereomer
major diastereomer
f1 (ppm)

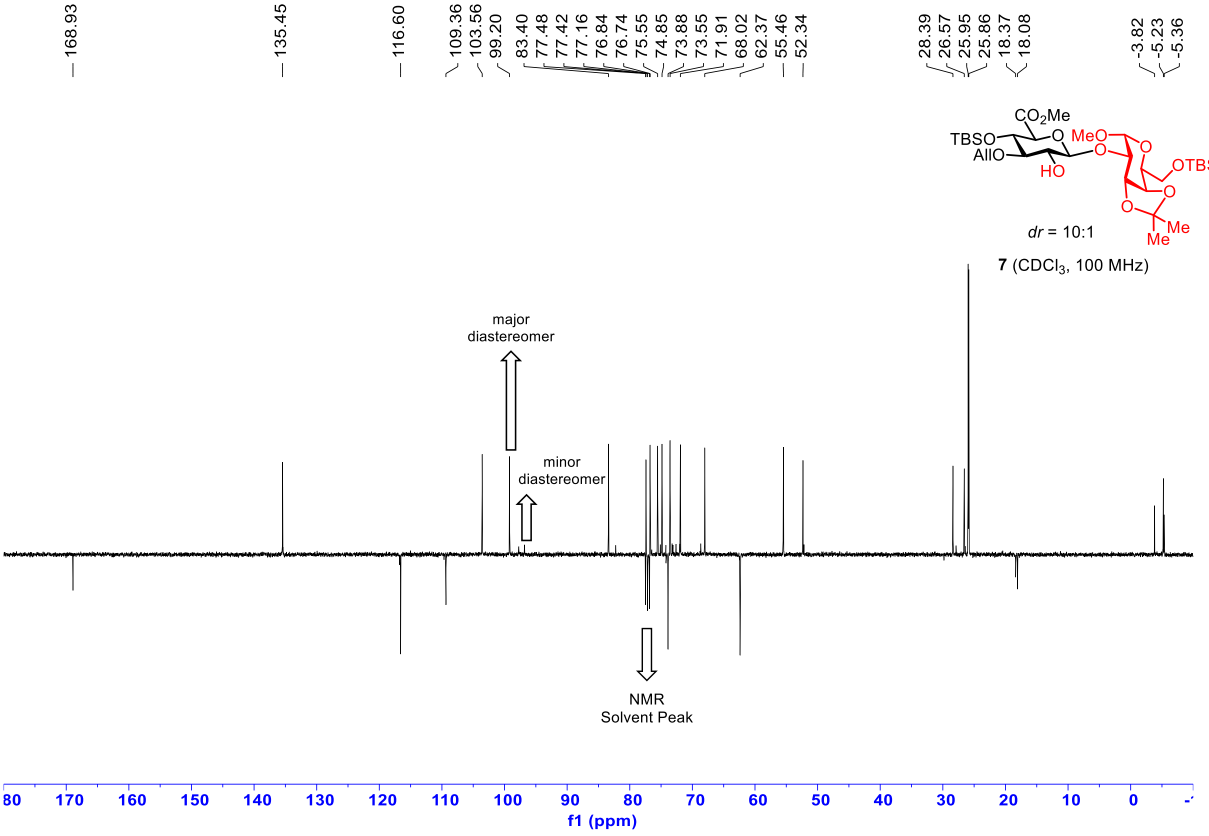

dr = 10:1
7 (CDCl3, 100 MHz)
major diastereomer
minor diastereomer
NMR Solvent Peak
f1 (ppm)

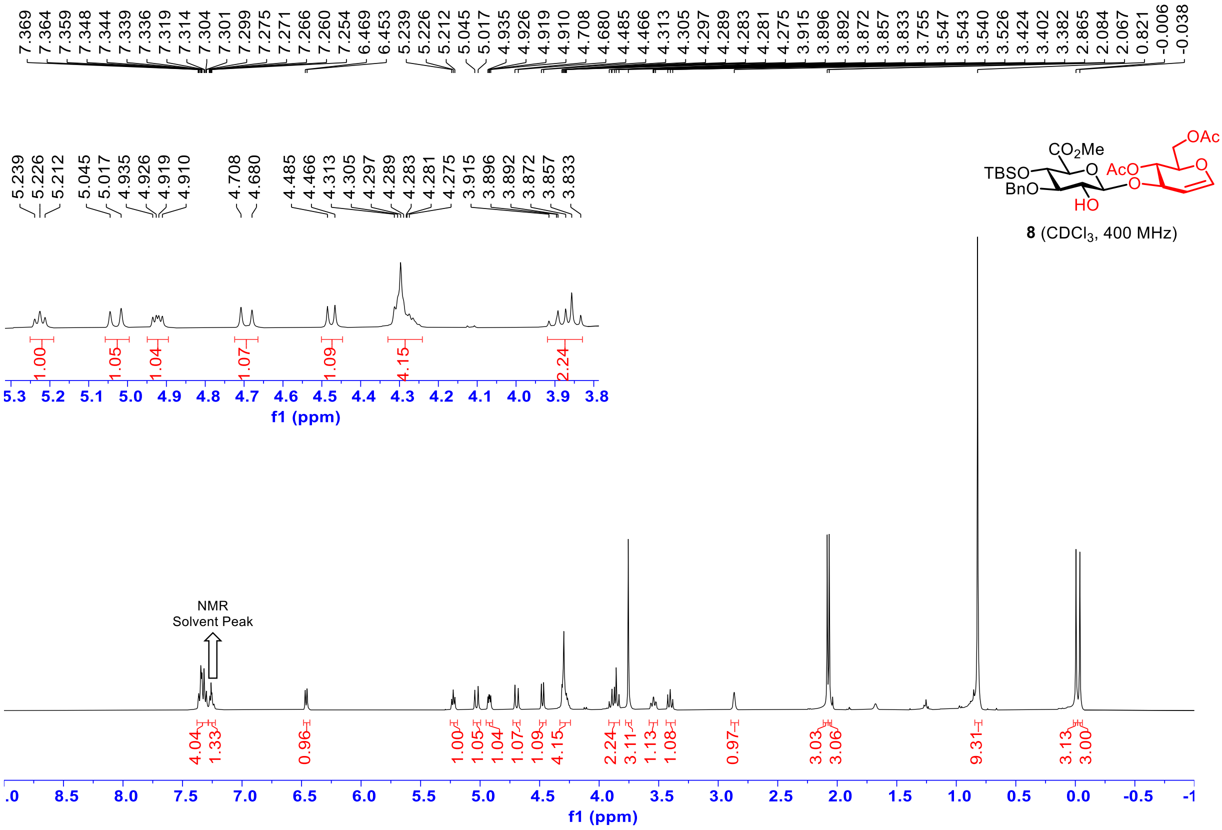

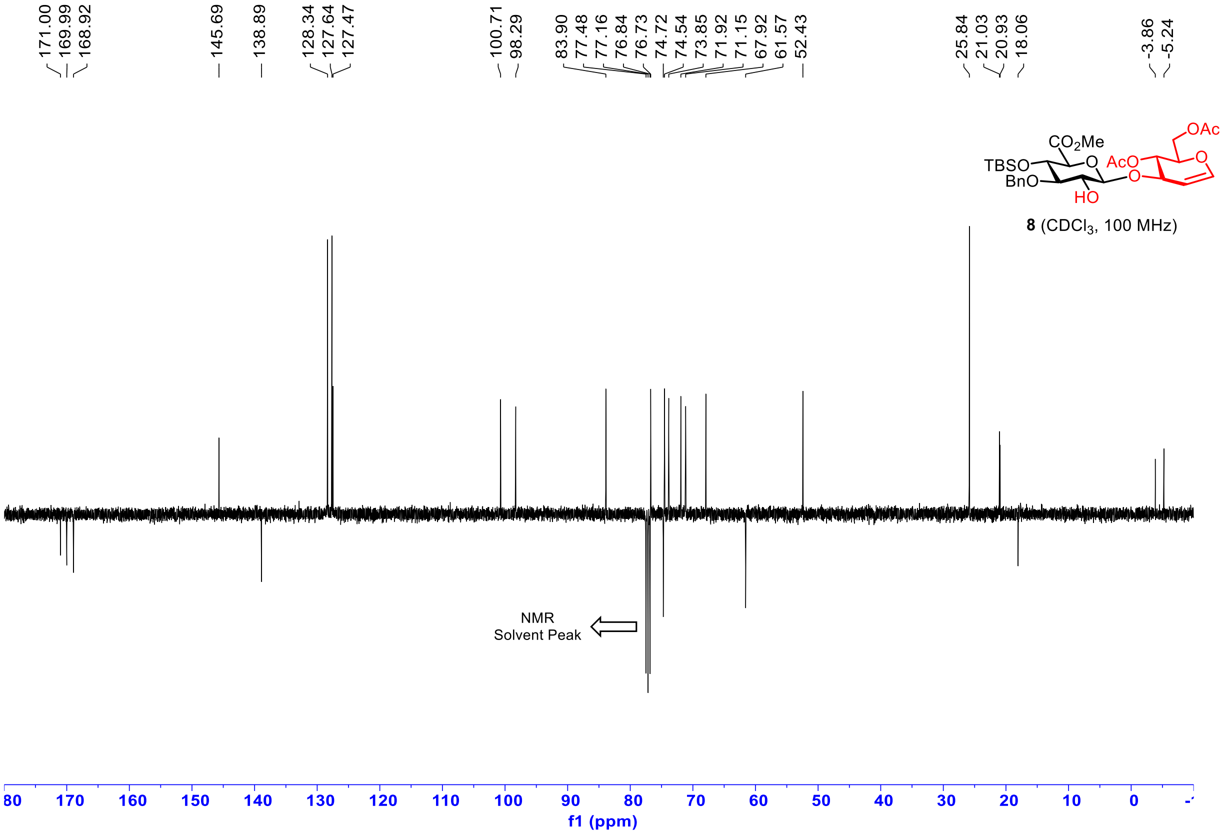

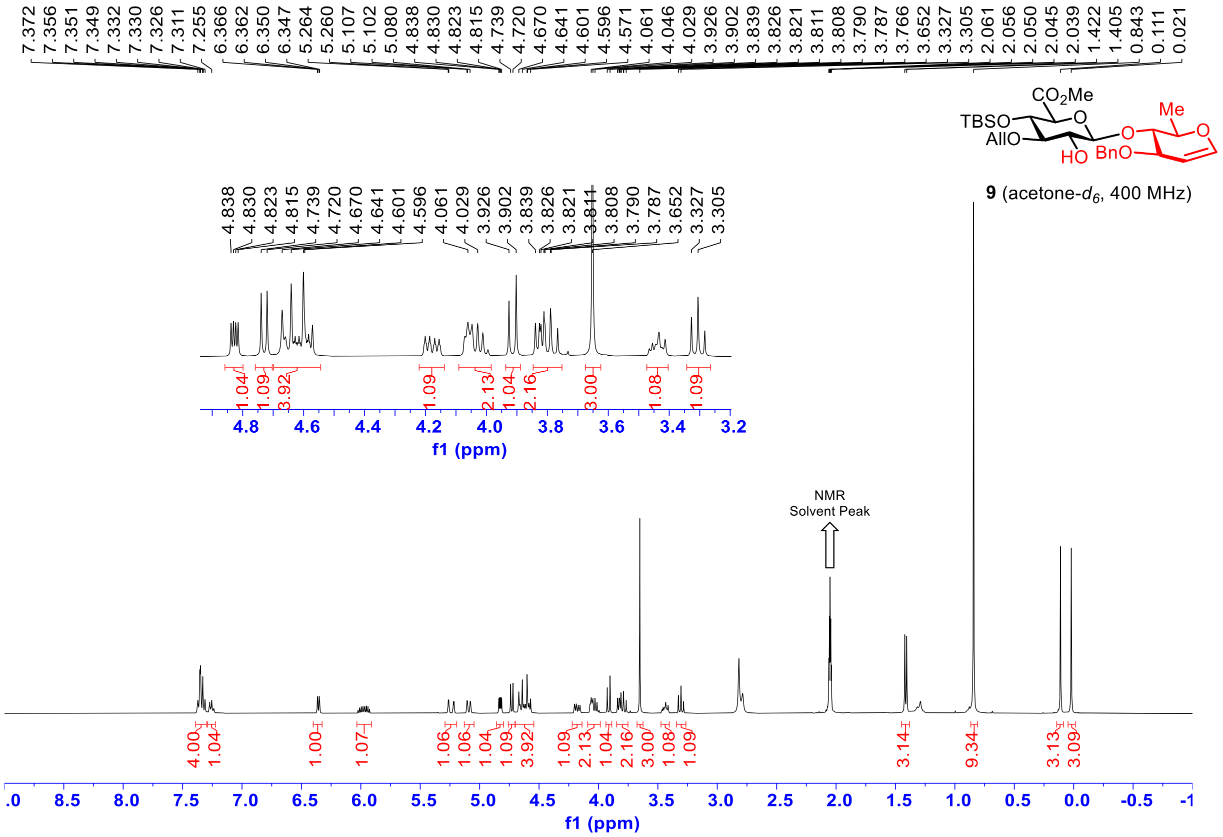
7.372 7.356 7.351 7.349 7.332 7.330 7.326 7.311 7.255 6.366 6.362 6.350 6.347 5.264 5.260 5.107 5.102 5.080 4.838 4.830 4.823 4.815 4.739 4.720 4.670 4.641 4.601 4.596 4.571 4.061 4.046 4.029 3.926 3.902 3.839 3.826 3.821 3.811 3.808 3.790 3.787 3.766 3.652 3.327 3.305 2.061 2.056 2.050 2.045 2.039 1.422 1.405 0.843 0.111 0.021
CO_2Me
TBSO
AllO
HO
BnO
Me
O
9 (acetone-d_6, 400 MHz)
NMR
Solvent Peak
f1 (ppm)

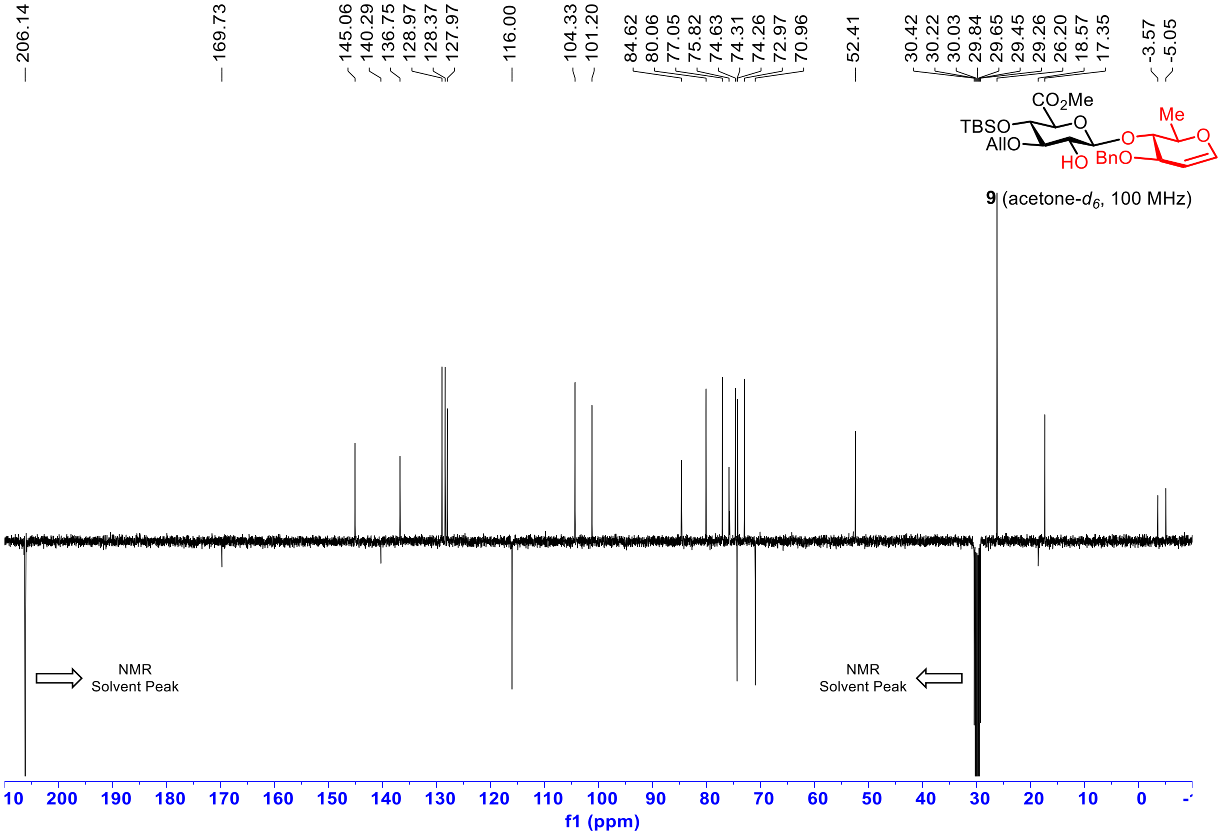
206.14 169.73 145.06 140.29 136.75 128.97 128.37 127.97 116.00 104.33 101.20 84.62 80.06 77.05 75.82 74.63 74.31 74.26 72.97 70.96 52.41 30.42 30.22 30.03 29.84 29.65 29.45 29.26 26.20 18.57 17.35 -3.57 -5.05
CO_2Me
TBSO
AllO
HO
BnO
Me
O
9 (acetone-d_6, 100 MHz)
NMR
Solvent Peak
f1 (ppm)

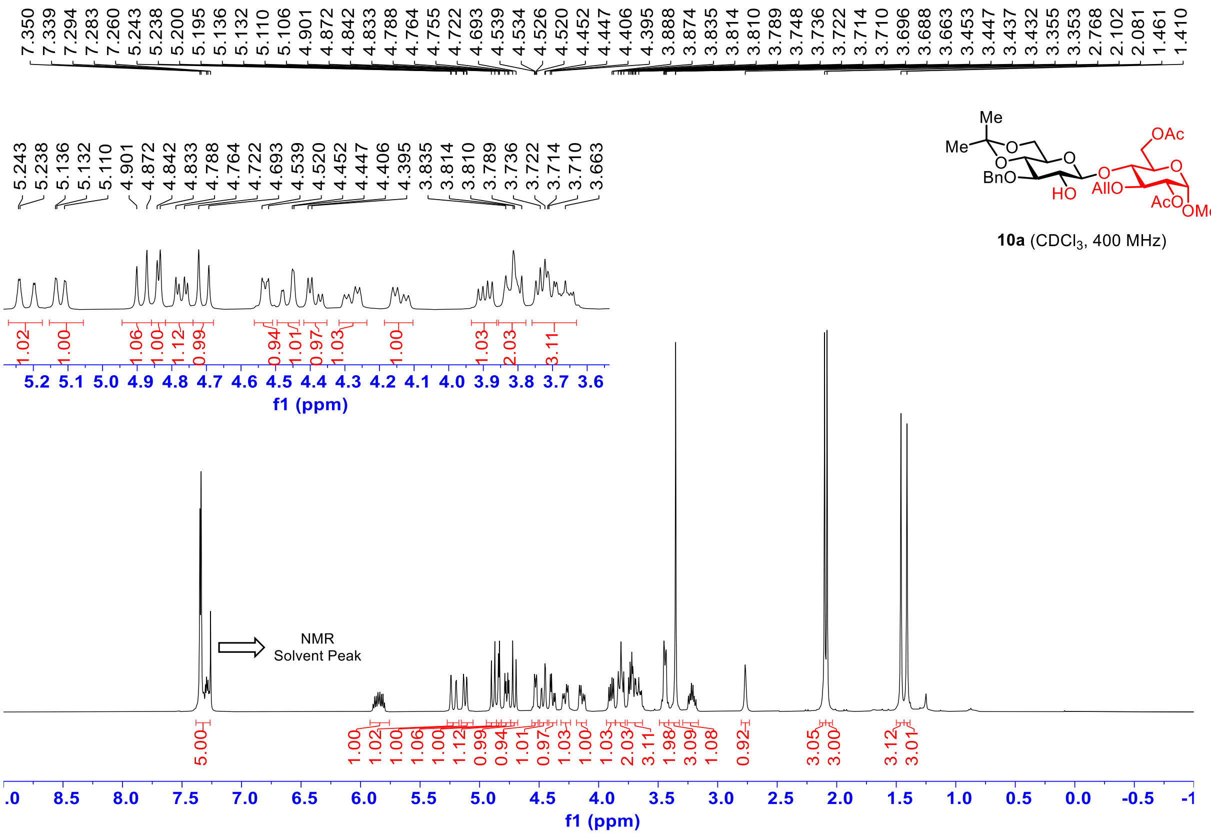

10a (CDCl$_3$, 400 MHz)
NMR Solvent Peak
f1 (ppm)

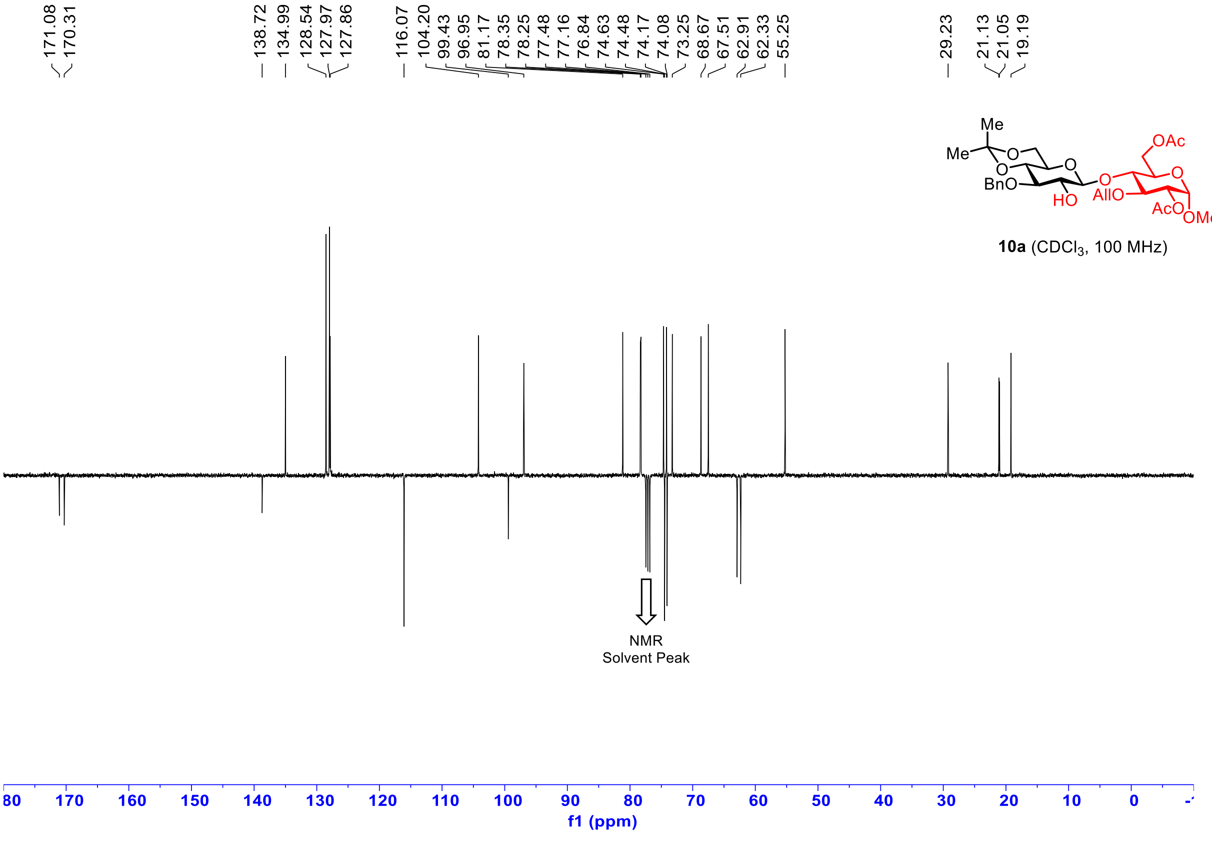

10a (CDCl$_3$, 100 MHz)
NMR Solvent Peak
f1 (ppm)

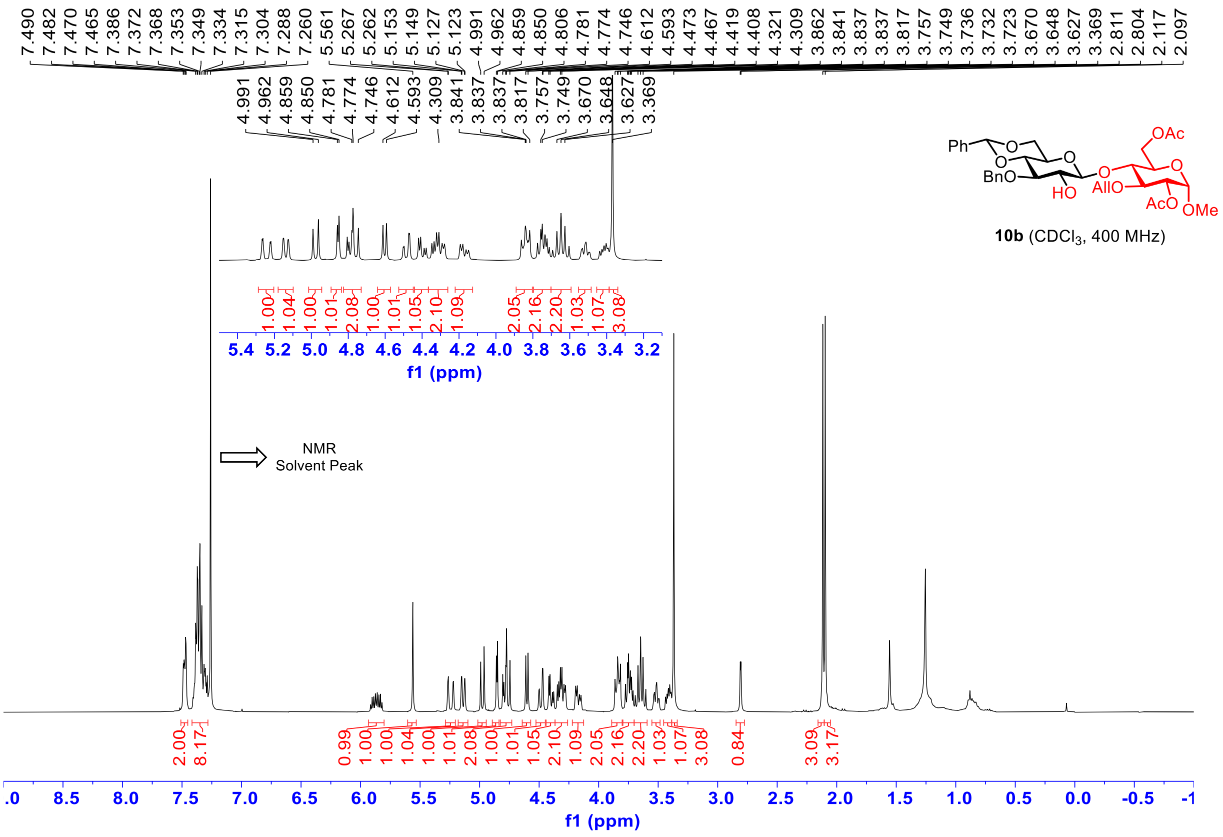
10b (CDCl3, 400 MHz)
NMR
Solvent Peak
f1 (ppm)

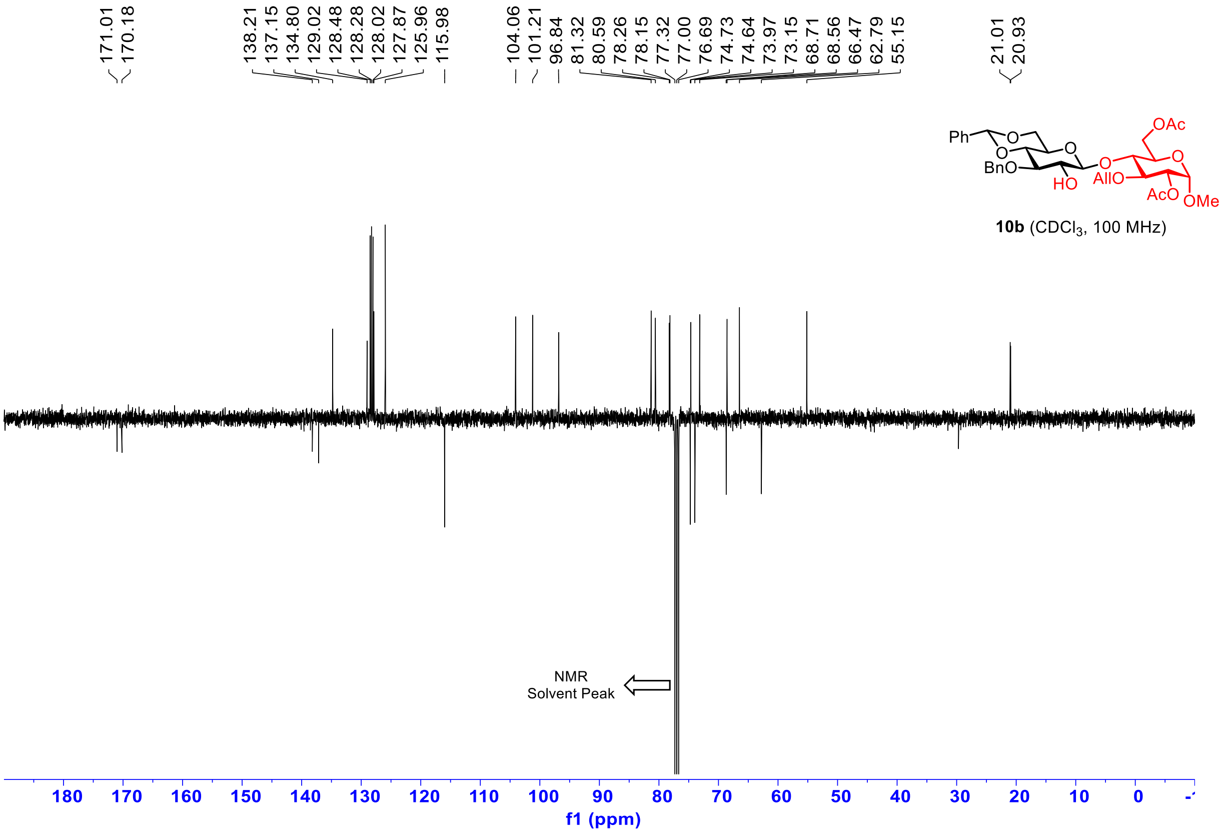
10b (CDCl3, 100 MHz)
NMR
Solvent Peak
f1 (ppm)

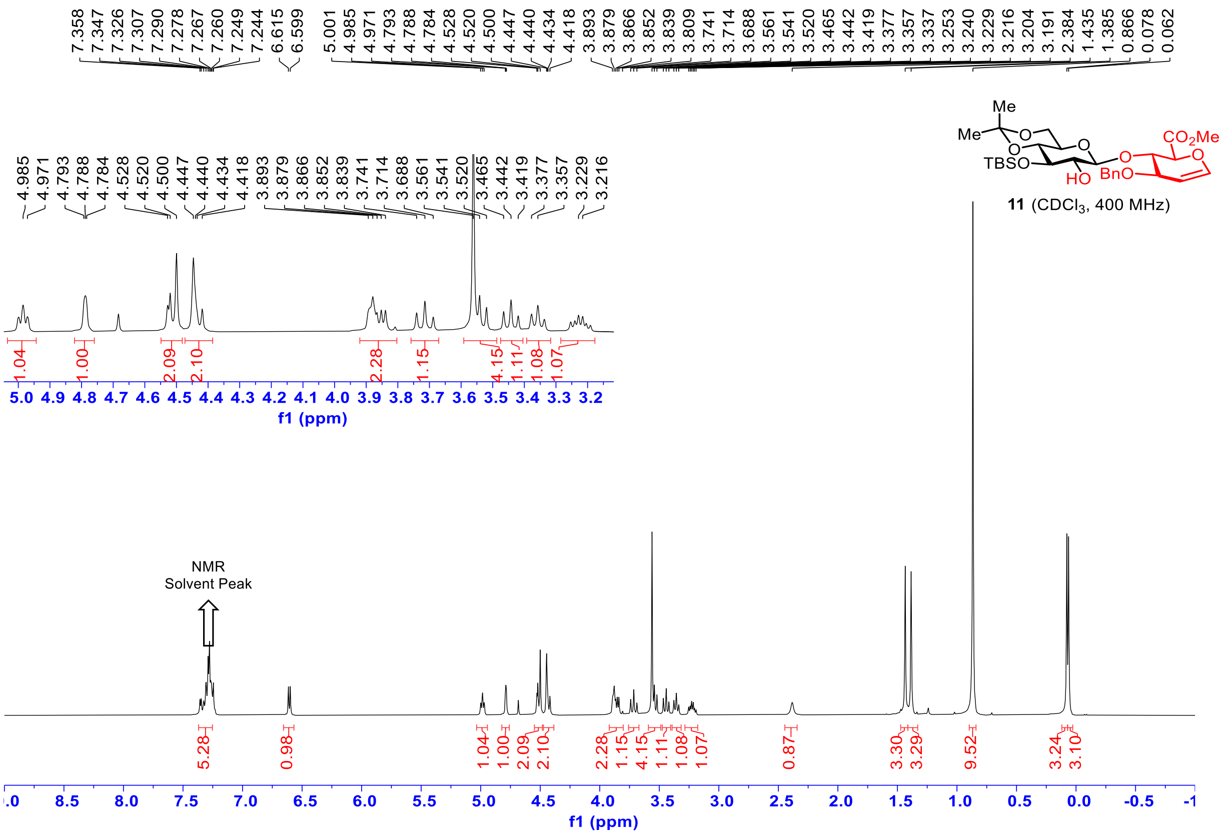
7.358 7.347 7.326 7.307 7.290 7.278 7.267 7.260 7.249 7.244 6.615 6.599 5.001 4.985 4.971 4.793 4.788 4.784 4.528 4.520 4.500 4.447 4.440 4.434 4.418 3.893 3.879 3.866 3.852 3.839 3.809 3.741 3.714 3.688 3.561 3.541 3.520 3.465 3.442 3.419 3.377 3.357 3.337 3.253 3.240 3.229 3.216 3.204 3.191 2.384 1.435 1.385 0.866 0.078 0.062
Me
Me
O
O
O
O
TBSO
HO
CO2Me
O
O
BnO
11 (CDCl3, 400 MHz)
NMR
Solvent Peak
f1 (ppm)
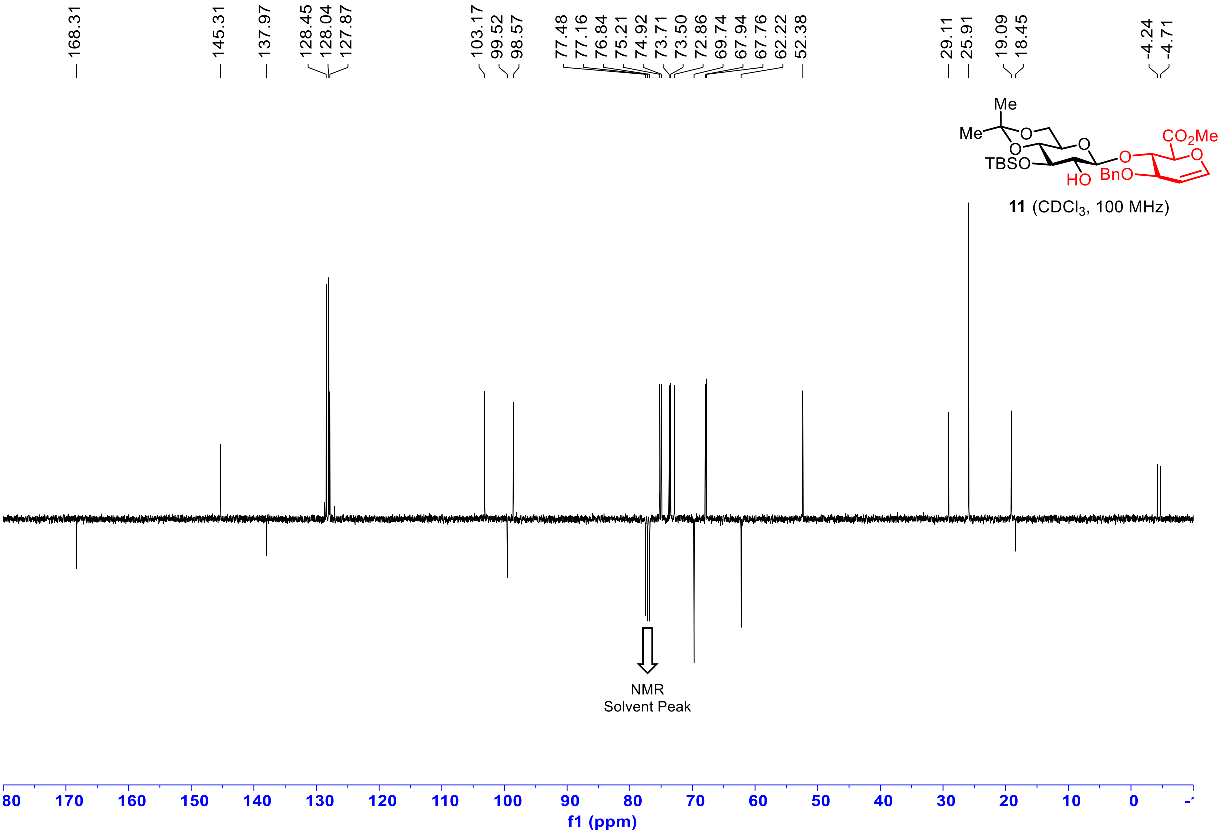
168.31 145.31 137.97 128.45 128.04 127.87 103.17 99.52 98.57 77.48 77.16 76.84 75.21 74.92 73.71 73.50 72.86 69.74 67.94 67.76 62.22 52.38 29.11 25.91 19.09 18.45 -4.24 -4.71
11 (CDCl3, 100 MHz)
NMR
Solvent Peak
f1 (ppm)

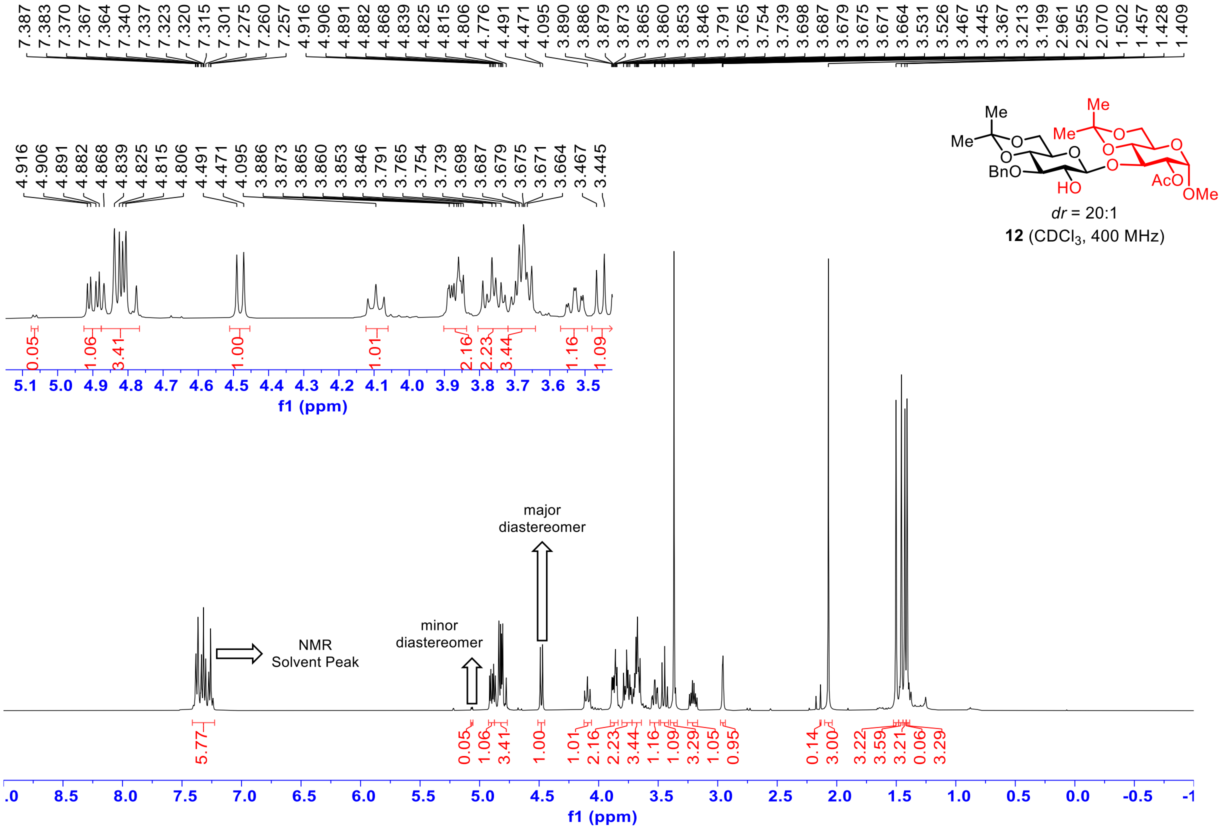
7.387 7.383 7.370 7.367 7.364 7.340 7.337 7.323 7.320 7.315 7.301 7.275 7.260 7.257 4.916 4.906 4.891 4.882 4.868 4.839 4.825 4.815 4.806 4.776 4.491 4.471 4.095 3.890 3.886 3.879 3.873 3.865 3.860 3.853 3.846 3.791 3.765 3.754 3.739 3.698 3.687 3.679 3.675 3.671 3.664 3.531 3.526 3.467 3.445 3.367 3.213 3.199 2.961 2.955 2.070 1.502 1.457 1.428 1.409
dr = 20:1
12 (CDCl$_3$, 400 MHz)
major diastereomer
minor diastereomer
NMR Solvent Peak
f1 (ppm)

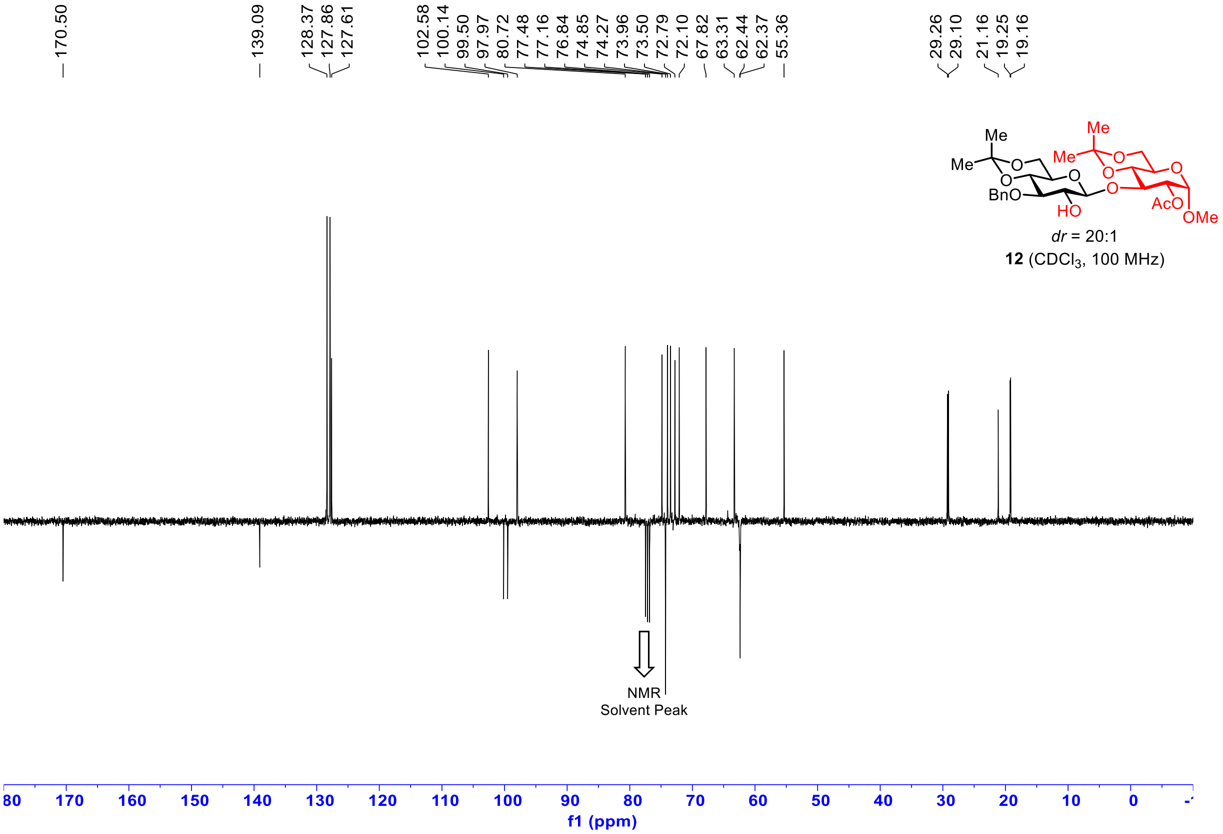
170.50 139.09 128.37 127.86 127.61 102.58 100.14 99.50 97.97 80.72 77.48 77.16 76.84 74.85 74.27 73.96 73.50 72.79 72.10 67.82 63.31 62.44 62.37 55.36 29.26 29.10 21.16 19.25 19.16
dr = 20:1
12 (CDCl$_3$, 100 MHz)
NMR Solvent Peak
f1 (ppm)

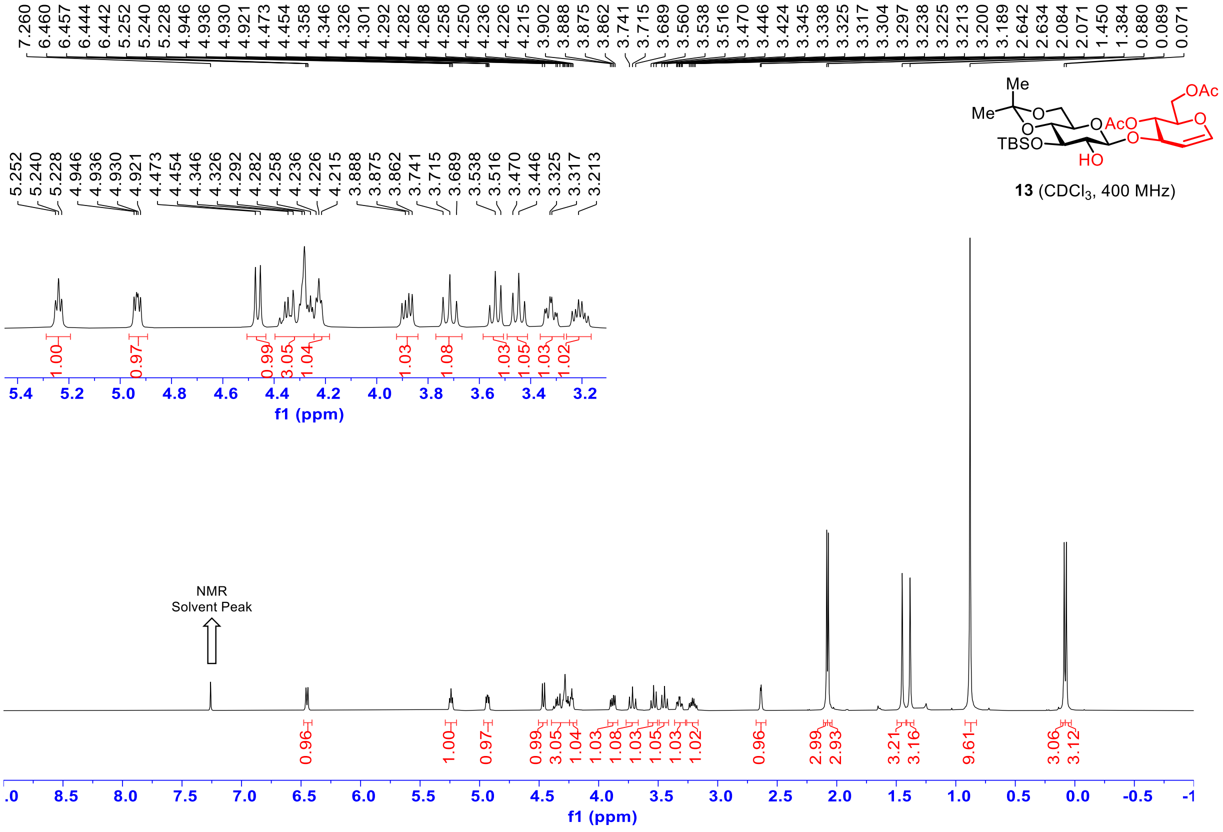

13 (CDCl3, 400 MHz)
NMR Solvent Peak
f1 (ppm)

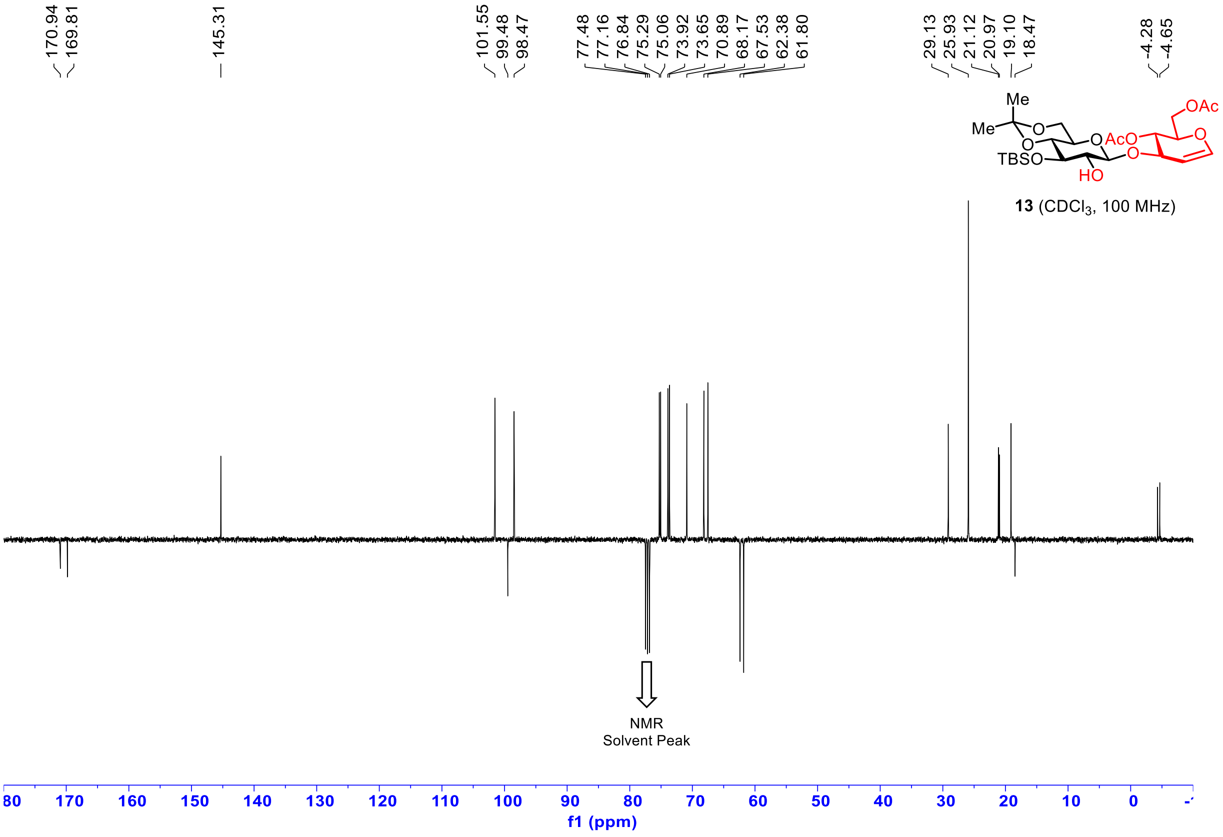

13 (CDCl3, 100 MHz)
NMR Solvent Peak
f1 (ppm)

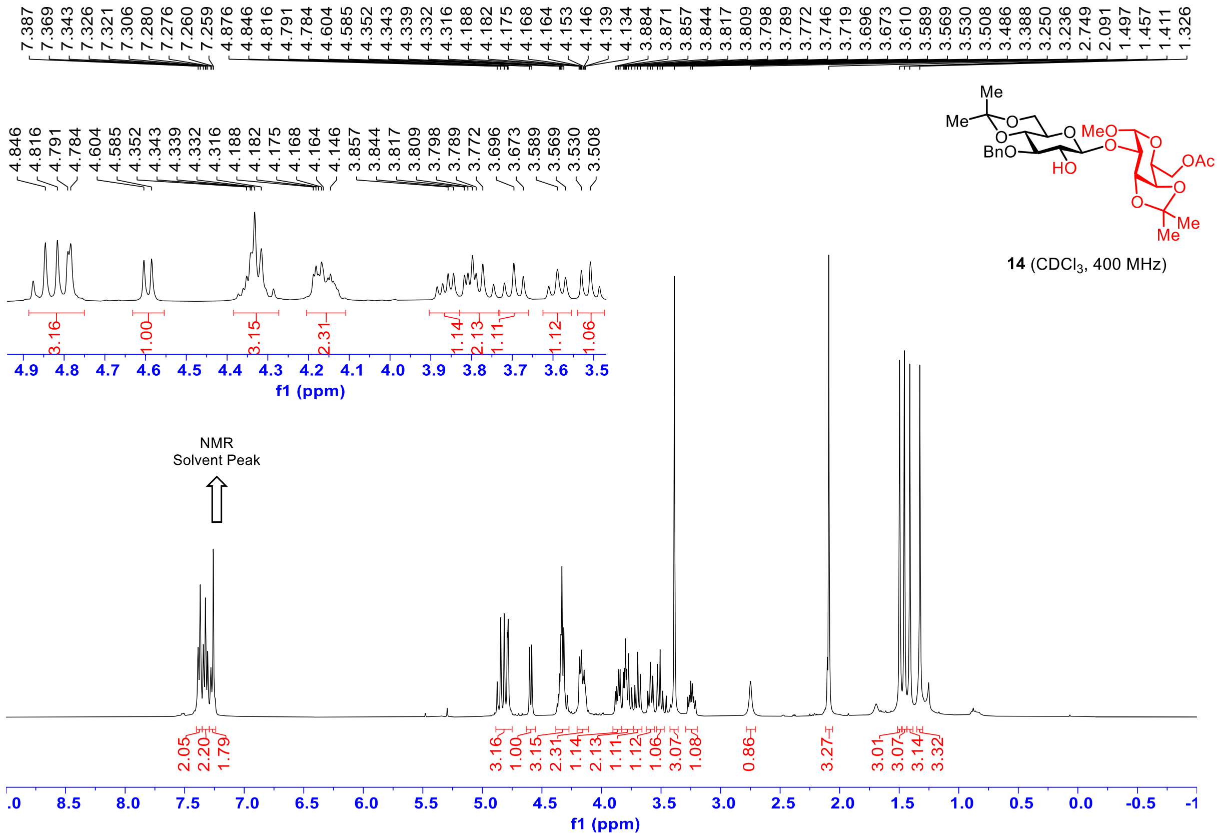
14 (CDCl$_3$, 400 MHz)
NMR Solvent Peak
f1 (ppm)

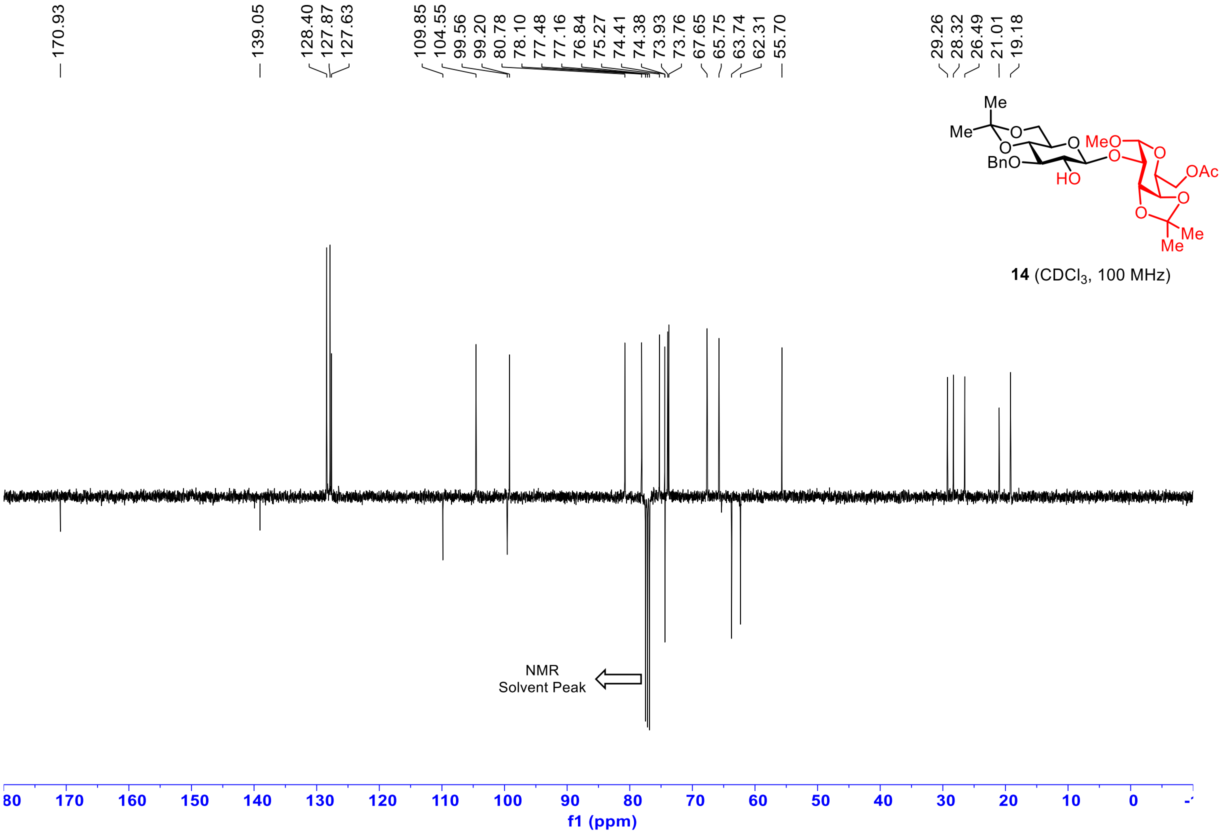
14 (CDCl$_3$, 100 MHz)
NMR Solvent Peak
f1 (ppm)

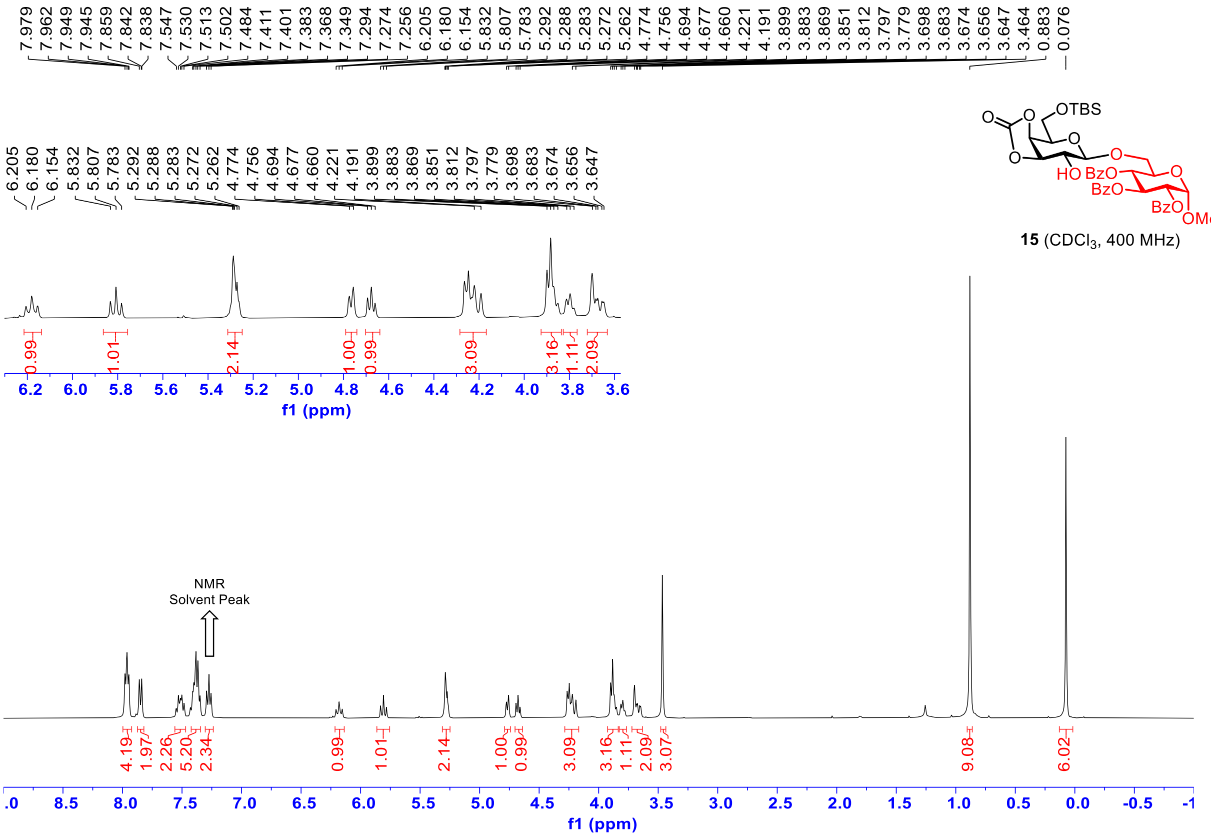
OTBS
HO BzO
BzO
BzO
OMe
15 (CDCl3, 400 MHz)
NMR Solvent Peak
f1 (ppm)

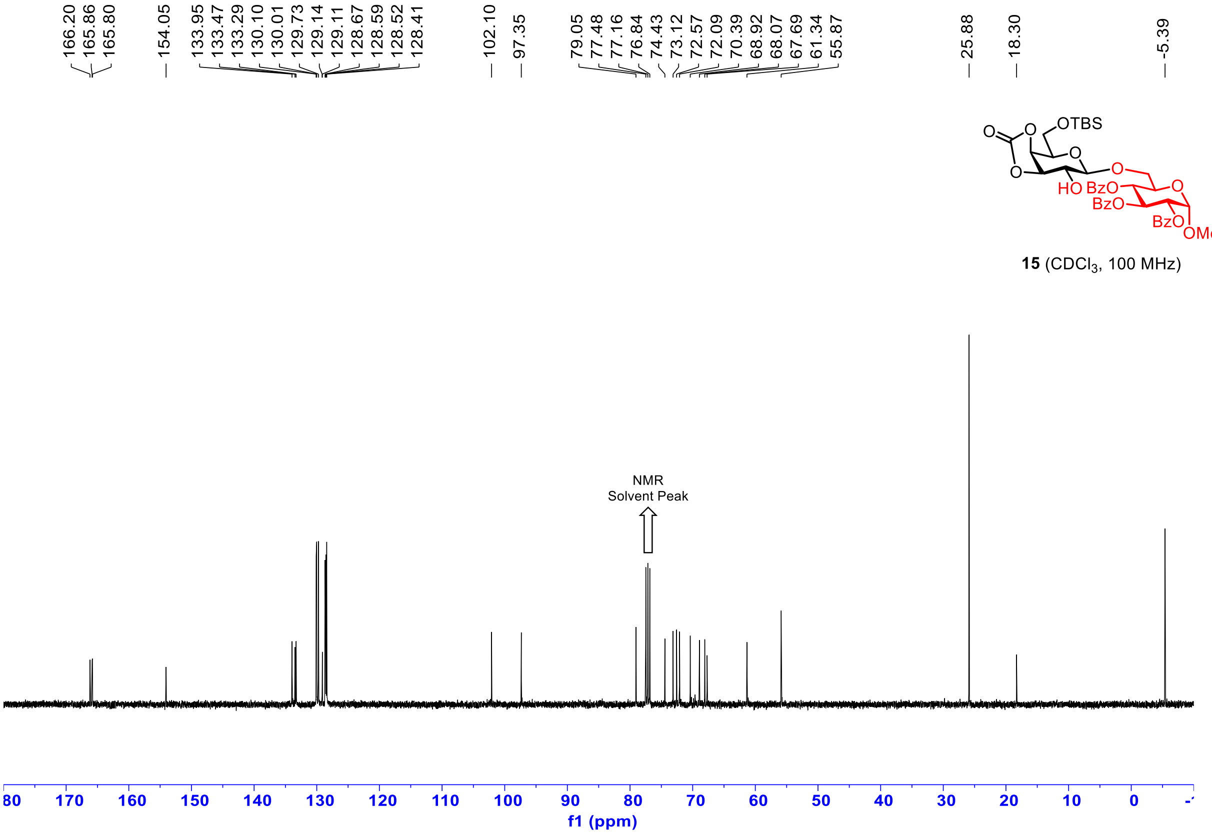
OTBS
HO BzO
BzO
BzO
OMe
15 (CDCl3, 100 MHz)
NMR Solvent Peak
f1 (ppm)

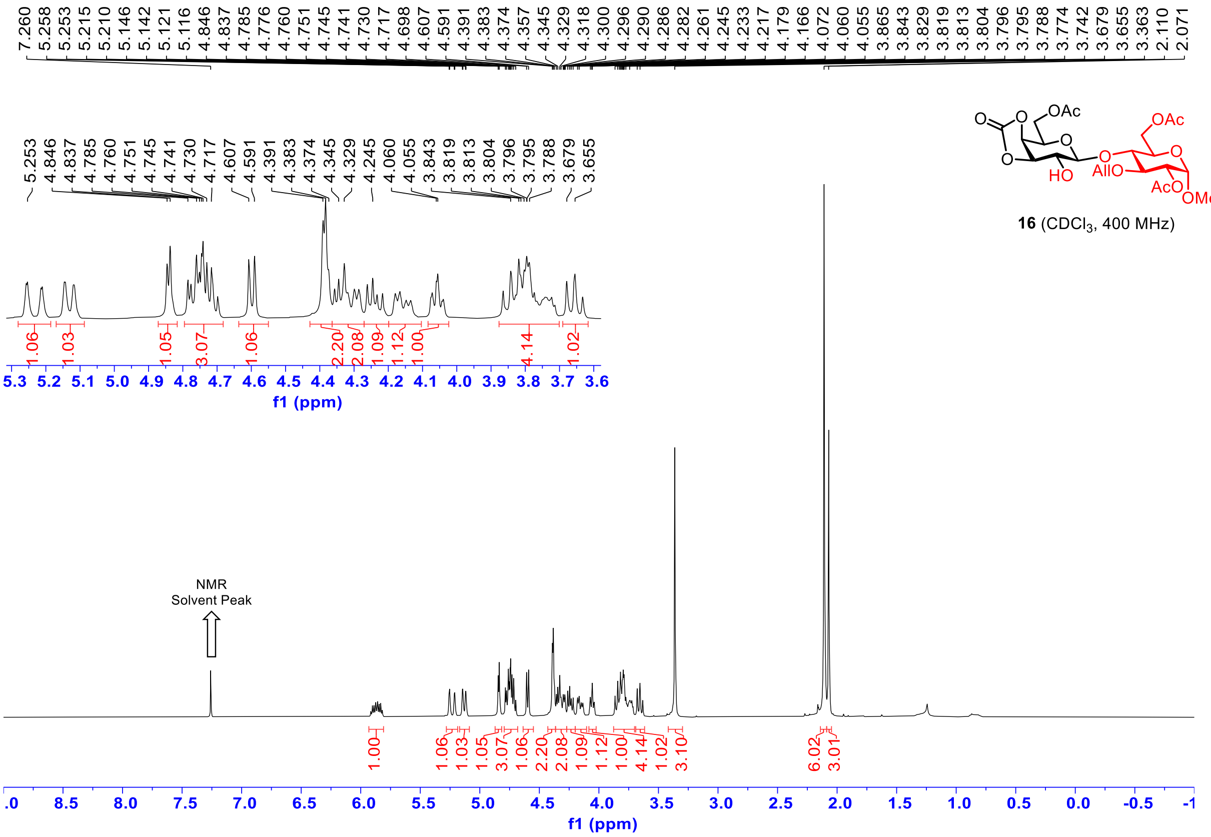
7.260 5.258 5.253 5.215 5.210 5.146 5.142 5.121 5.116 4.846 4.837 4.785 4.776 4.760 4.751 4.745 4.741 4.730 4.717 4.698 4.607 4.591 4.391 4.383 4.374 4.357 4.345 4.329 4.318 4.300 4.296 4.290 4.286 4.282 4.261 4.245 4.233 4.217 4.179 4.166 4.072 4.060 4.055 3.865 3.843 3.829 3.819 3.813 3.804 3.796 3.795 3.788 3.774 3.742 3.679 3.655 3.363 2.110 2.071
OAc
HO
AllO
AcO
OMe
16 (CDCl$_3$, 400 MHz)
NMR Solvent Peak
1.00 1.06 1.03 1.05 3.07 1.06 2.20 2.08 1.09 1.12 1.00 4.14 1.02 3.10 6.02 3.01
f1 (ppm)

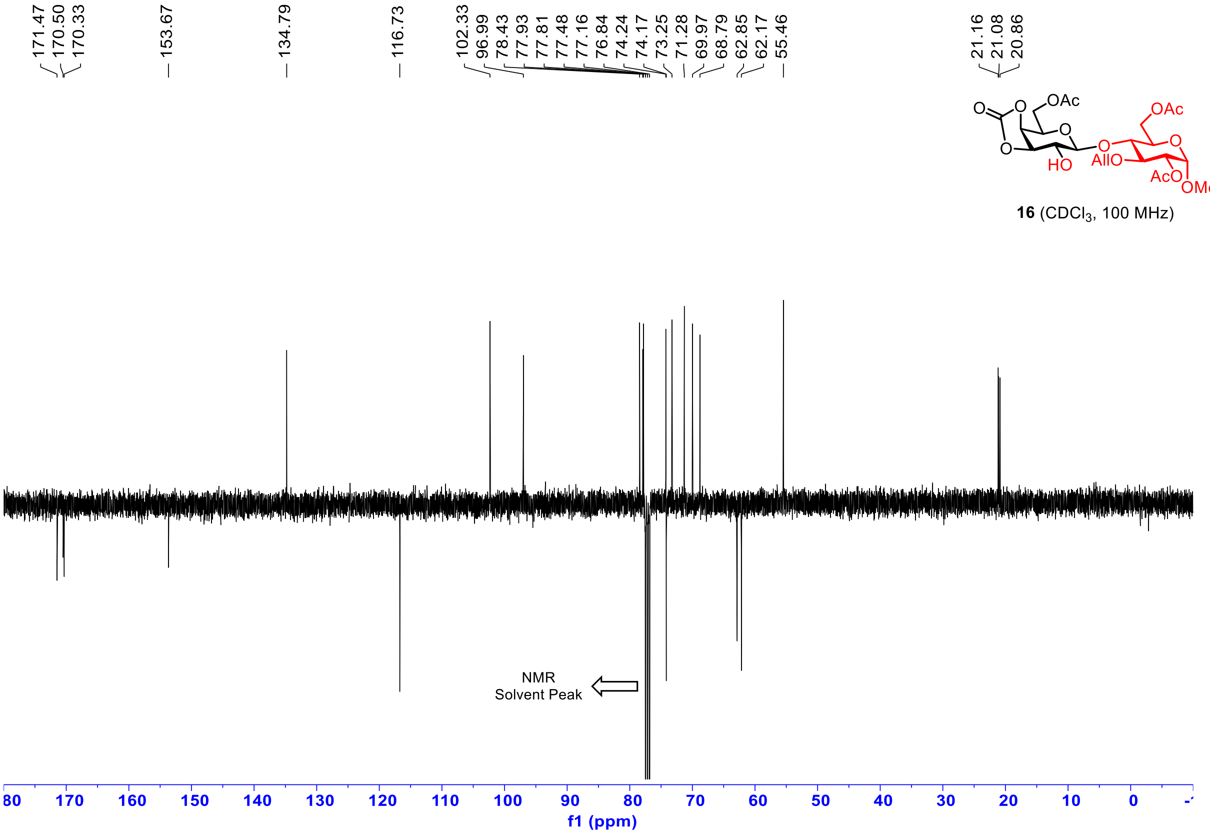
171.47 170.50 170.33 153.67 134.79 116.73 102.33 96.99 78.43 77.93 77.81 77.48 77.16 76.84 74.24 74.17 73.25 71.28 69.97 68.79 62.85 62.17 55.46 21.16 21.08 20.86
OAc
HO
AllO
AcO
OMe
16 (CDCl$_3$, 100 MHz)
NMR Solvent Peak
f1 (ppm)

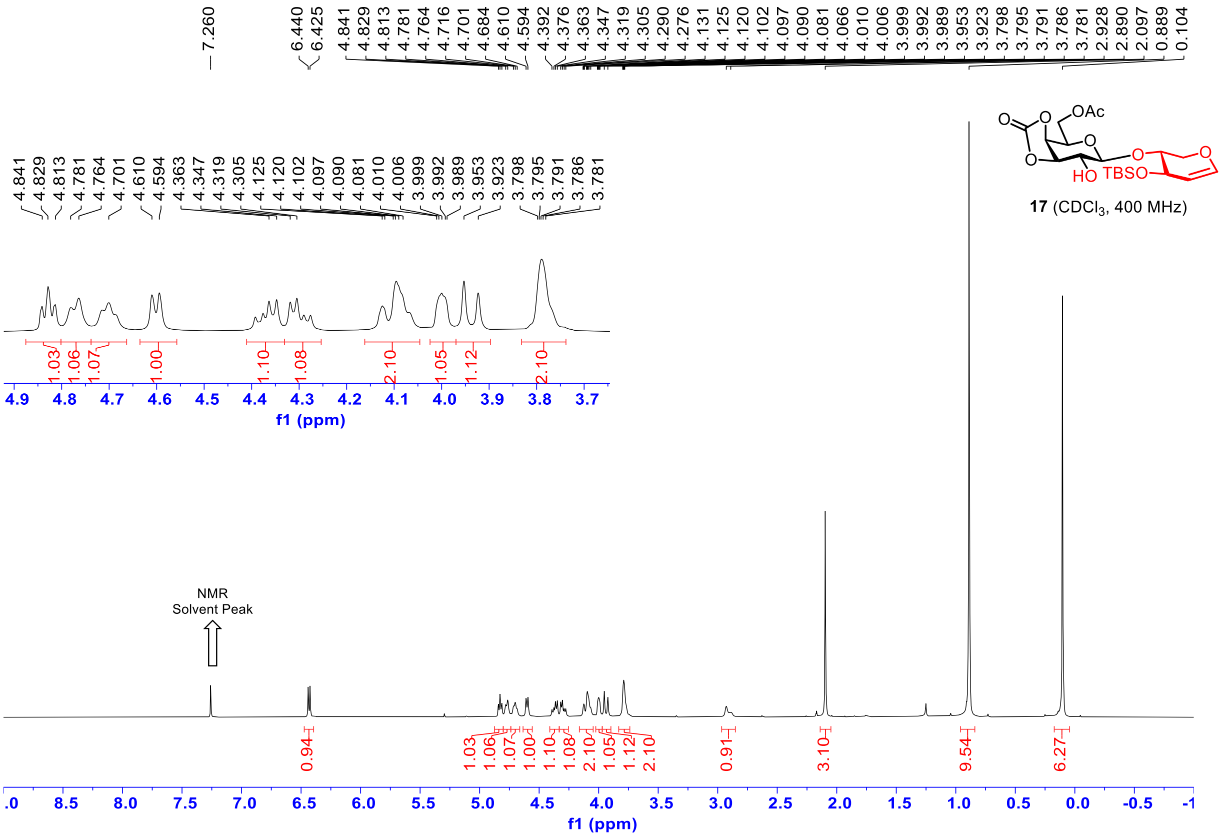
7.260
6.440
6.425
4.841
4.829
4.813
4.781
4.764
4.716
4.701
4.684
4.610
4.594
4.392
4.376
4.363
4.347
4.319
4.305
4.290
4.276
4.131
4.125
4.120
4.102
4.097
4.090
4.081
4.066
4.010
4.006
3.999
3.992
3.989
3.953
3.923
3.798
3.795
3.791
3.786
3.781
2.928
2.890
2.097
0.889
0.104
OAc
HO
TBSO
17 (CDCl3, 400 MHz)
NMR
Solvent Peak
f1 (ppm)

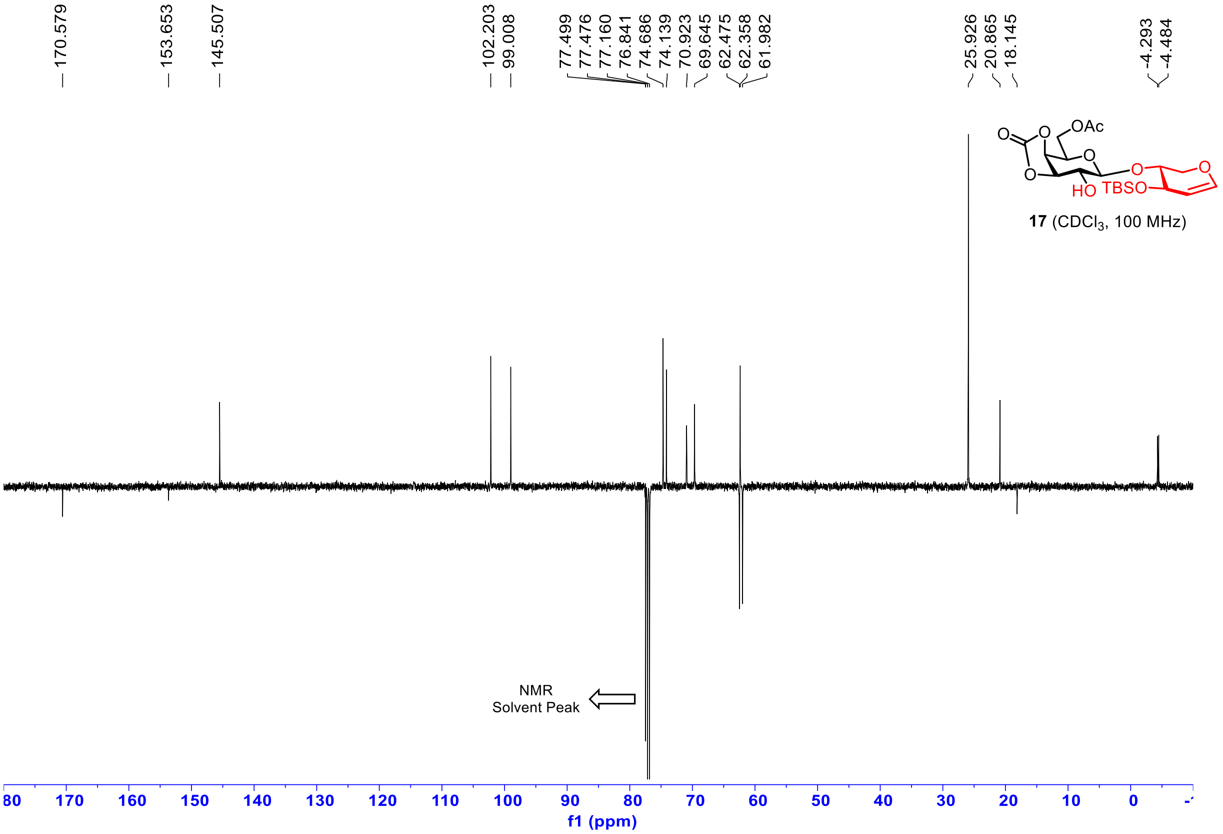
170.579
153.653
145.507
102.203
99.008
77.499
77.476
77.160
76.841
74.686
74.139
70.923
69.645
62.475
62.358
61.982
25.926
20.865
18.145
-4.293
-4.484
OAc
HO
TBSO
17 (CDCl3, 100 MHz)
NMR
Solvent Peak
f1 (ppm)

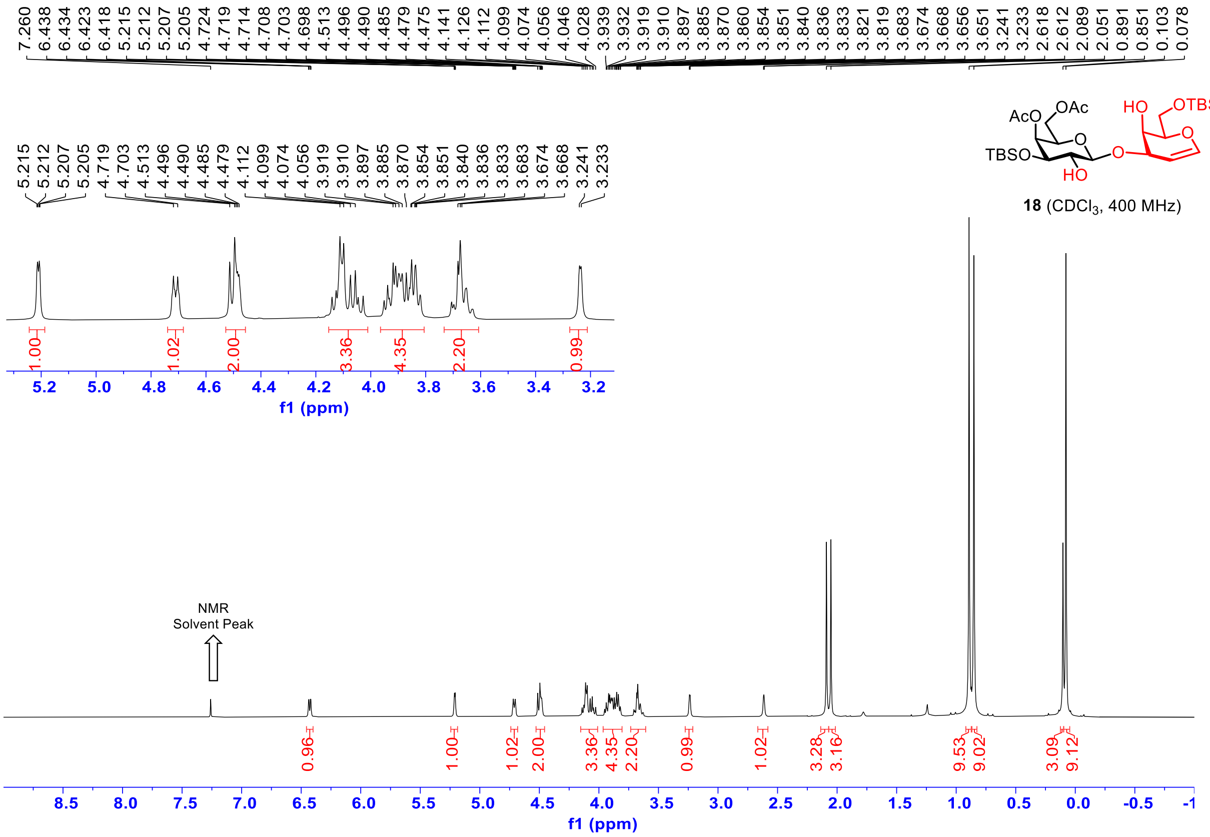
7.260 6.438 6.434 6.423 6.418 5.215 5.212 5.207 5.205 4.724 4.719 4.714 4.708 4.703 4.698 4.513 4.496 4.490 4.485 4.479 4.475 4.141 4.126 4.112 4.099 4.074 4.056 4.046 4.028 3.939 3.932 3.919 3.910 3.897 3.885 3.870 3.860 3.854 3.851 3.840 3.836 3.833 3.821 3.819 3.683 3.674 3.668 3.656 3.651 3.241 3.233 2.618 2.612 2.089 2.051 0.891 0.851 0.103 0.078
AcO
OAc
TBSO
HO
O
HO
OTBS
18 (CDCl3, 400 MHz)
NMR
Solvent Peak
0.96 1.00 1.02 2.00 3.36 4.35 2.20 0.99 1.02 3.28 3.16 9.53 9.02 3.09 9.12
f1 (ppm)

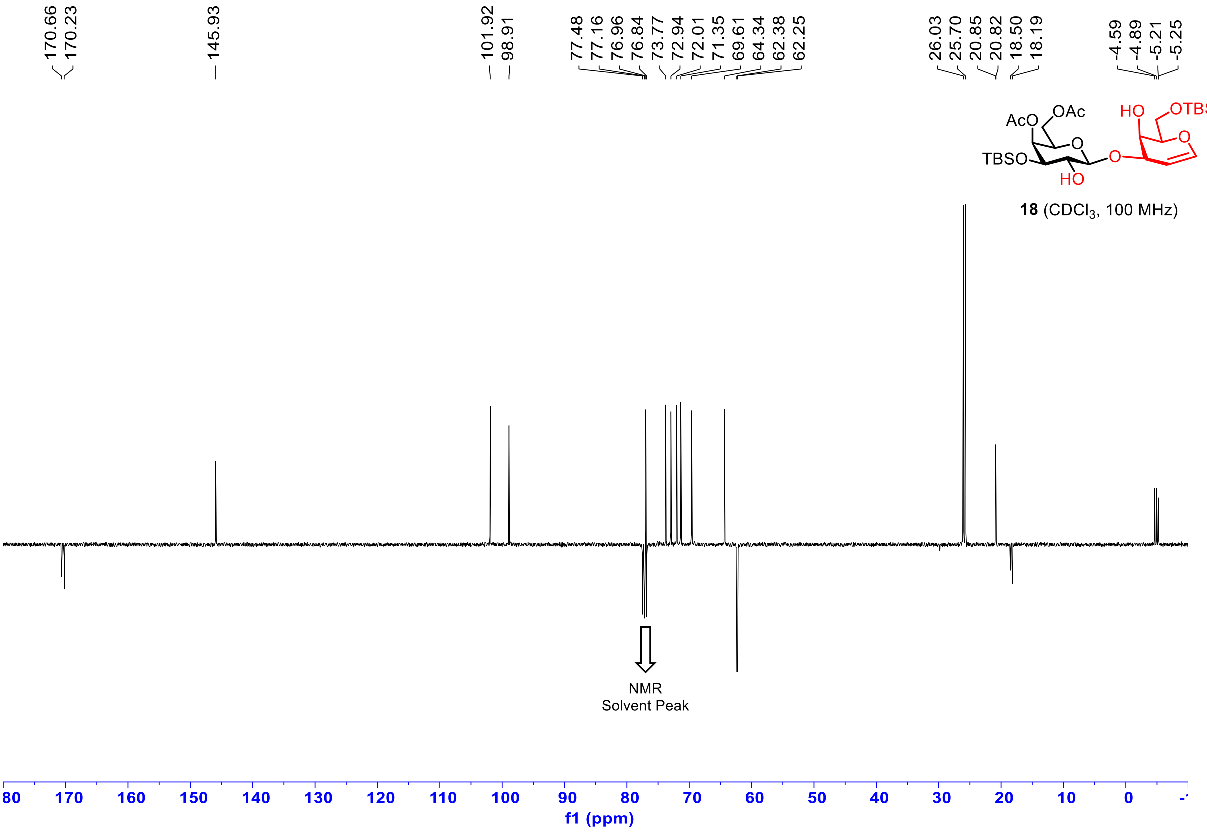
170.66 170.23 145.93 101.92 98.91 77.48 77.16 76.96 76.84 73.77 72.94 72.01 71.35 69.61 64.34 62.38 62.25 26.03 25.70 20.85 20.82 18.50 18.19 -4.59 -4.89 -5.21 -5.25
AcO
OAc
TBSO
HO
O
HO
OTBS
18 (CDCl3, 100 MHz)
NMR
Solvent Peak
f1 (ppm)

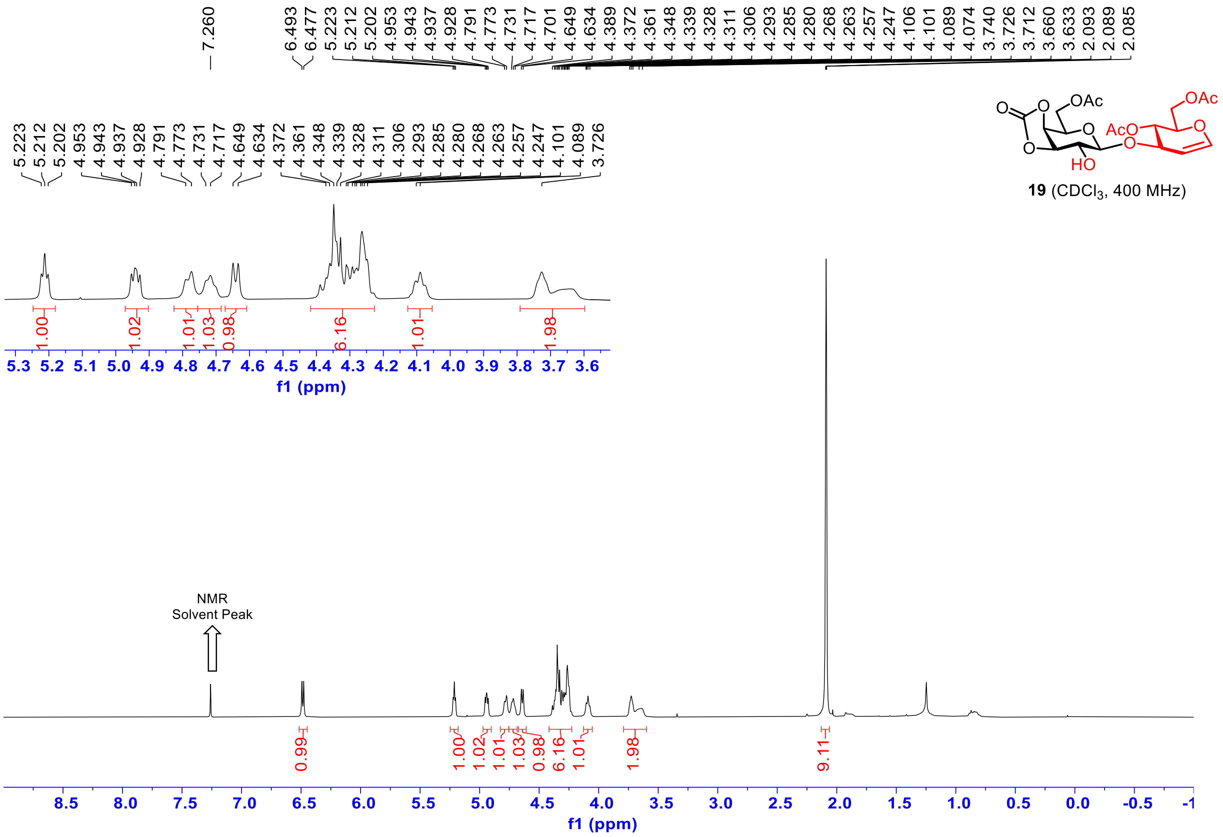

19 ($CDCl_3$, 400 MHz)

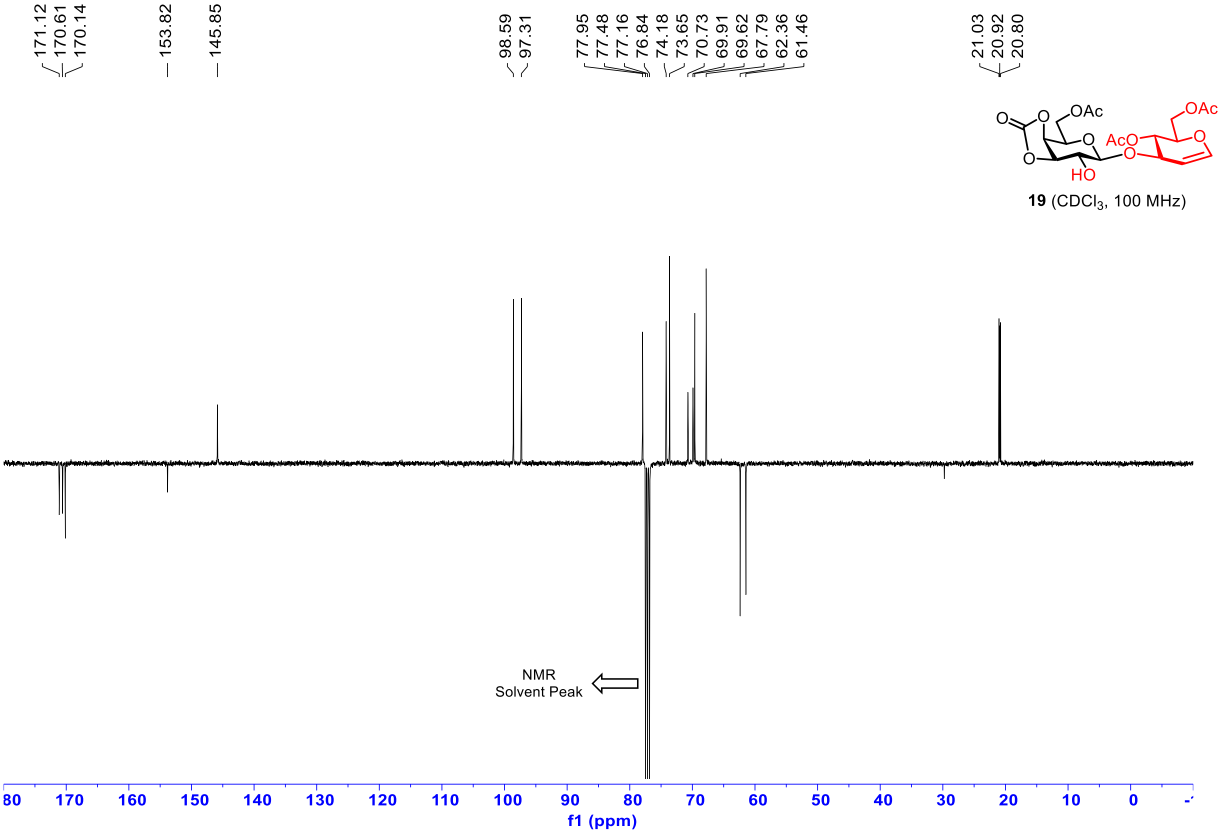

19 ($CDCl_3$, 100 MHz)

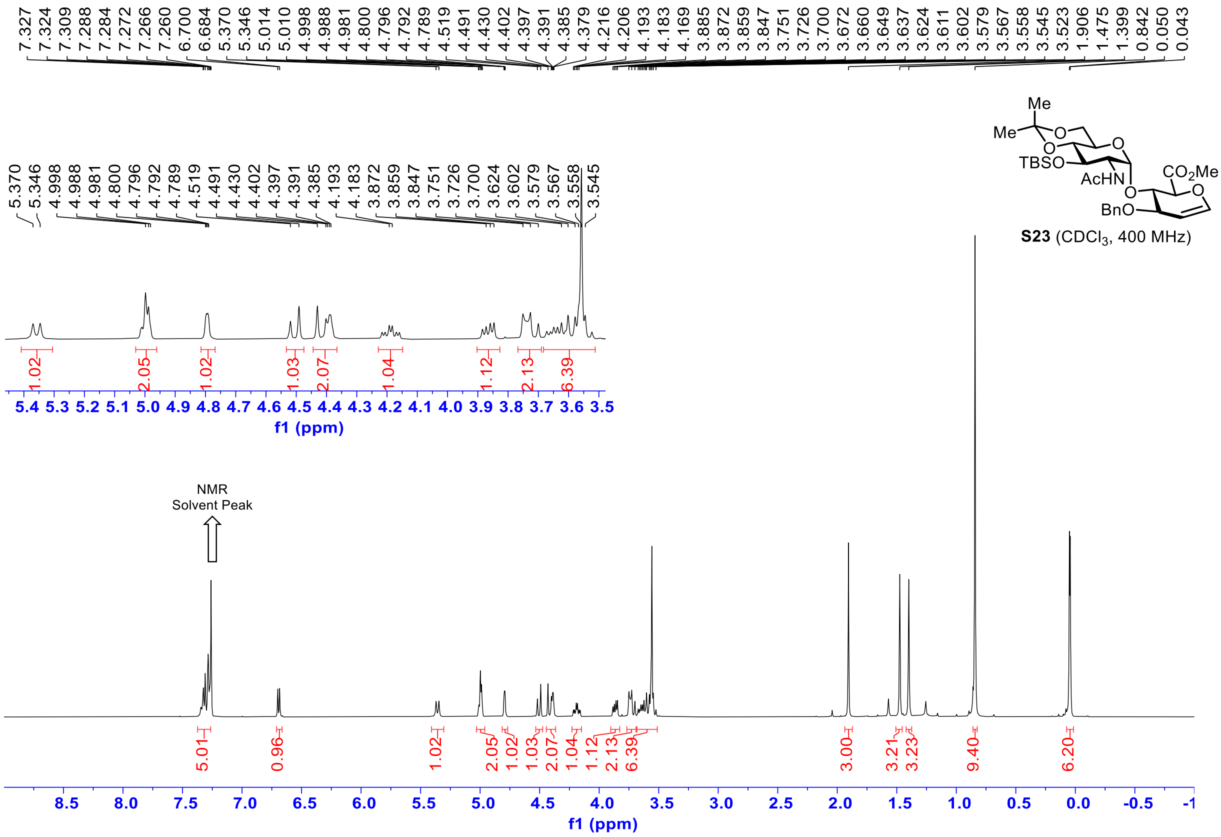
7.327 7.324 7.309 7.288 7.284 7.272 7.266 7.260 6.700 6.684 5.370 5.346 5.014 5.010 4.998 4.988 4.981 4.800 4.796 4.792 4.789 4.519 4.491 4.430 4.402 4.397 4.391 4.385 4.379 4.216 4.206 4.193 4.183 4.169 3.885 3.872 3.859 3.847 3.751 3.726 3.700 3.672 3.660 3.649 3.637 3.624 3.611 3.602 3.579 3.567 3.558 3.545 3.523 1.906 1.475 1.399 0.842 0.050 0.043
Me
Me
O
O
O
TBSO
AcHN
CO_2Me
O
O
BnO
S23 ($CDCl_3$, 400 MHz)
5.370 5.346 4.998 4.988 4.981 4.800 4.796 4.792 4.789 4.519 4.491 4.430 4.402 4.397 4.391 4.385 4.193 4.183 3.872 3.859 3.847 3.751 3.726 3.700 3.624 3.602 3.579 3.567 3.558 3.545
1.02 2.05 1.02 1.03 2.07 1.04 1.12 2.13 6.39
5.4 5.3 5.2 5.1 5.0 4.9 4.8 4.7 4.6 4.5 4.4 4.3 4.2 4.1 4.0 3.9 3.8 3.7 3.6 3.5
f1 (ppm)
NMR
Solvent Peak
5.01 0.96 1.02 2.05 1.02 1.03 2.07 1.04 1.12 2.13 6.39 3.00 3.21 3.23 9.40 6.20
8.5 8.0 7.5 7.0 6.5 6.0 5.5 5.0 4.5 4.0 3.5 3.0 2.5 2.0 1.5 1.0 0.5 0.0 -0.5
f1 (ppm)

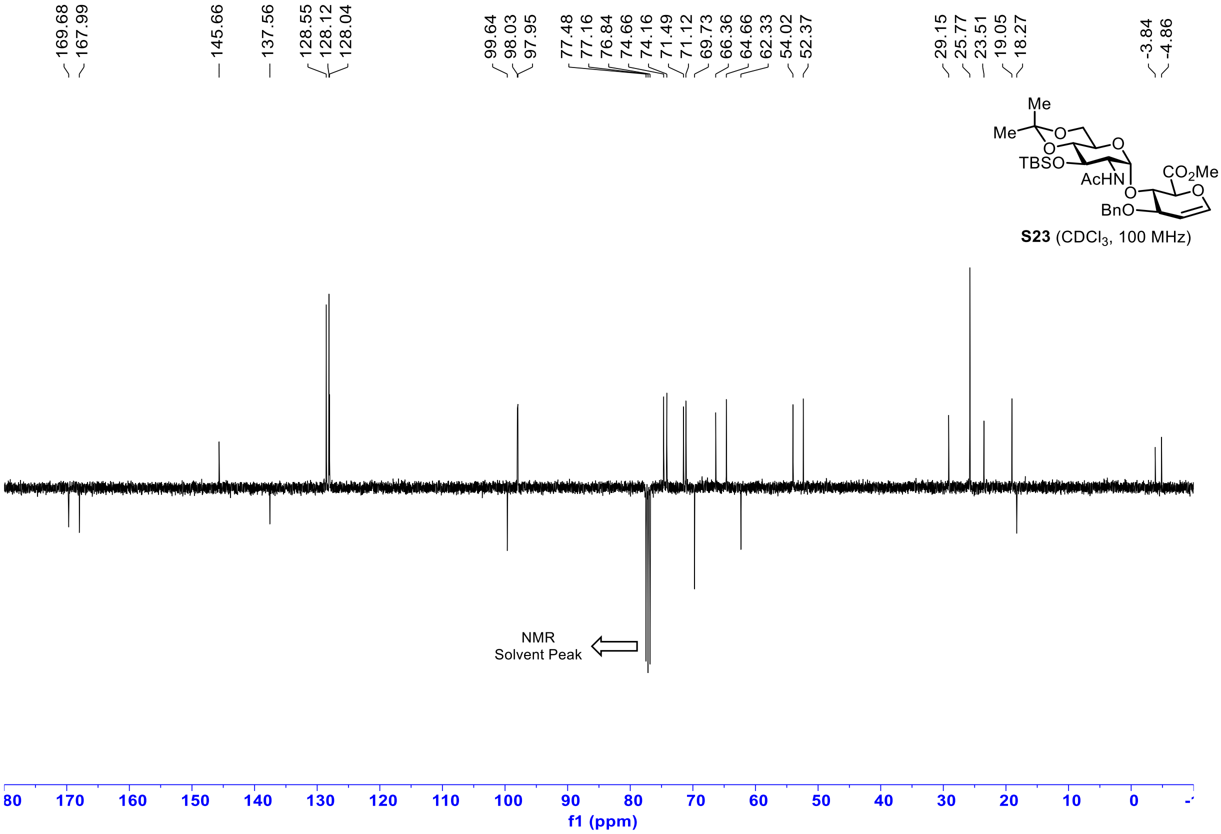
169.68 167.99 145.66 137.56 128.55 128.12 128.04 99.64 98.03 97.95 77.48 77.16 76.84 74.66 74.16 71.49 71.12 69.73 66.36 64.66 62.33 54.02 52.37 29.15 25.77 23.51 19.05 18.27 -3.84 -4.86
Me
Me
O
O
O
TBSO
AcHN
CO_2Me
O
O
BnO
S23 ($CDCl_3$, 100 MHz)
NMR
Solvent Peak
170 160 150 140 130 120 110 100 90 80 70 60 50 40 30 20 10 0
f1 (ppm)

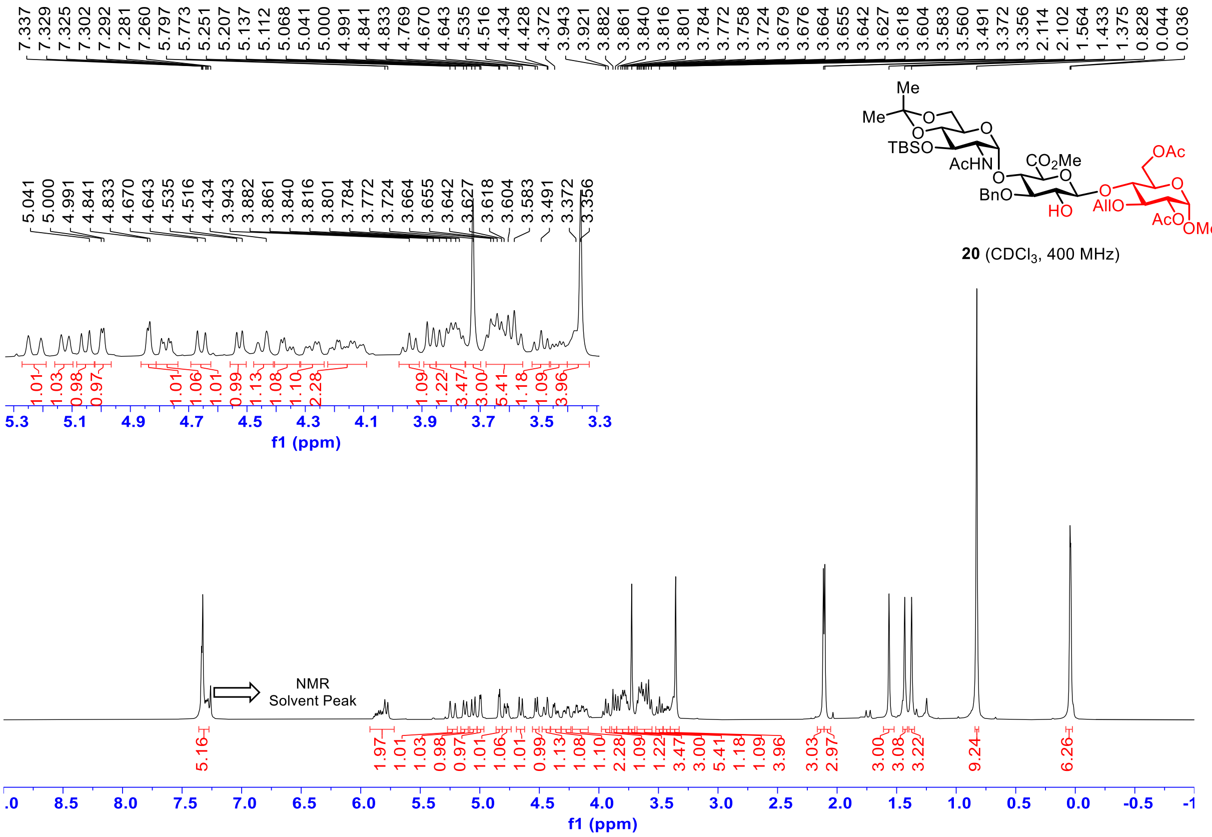
20 (CDCl3, 400 MHz)
NMR
Solvent Peak
f1 (ppm)

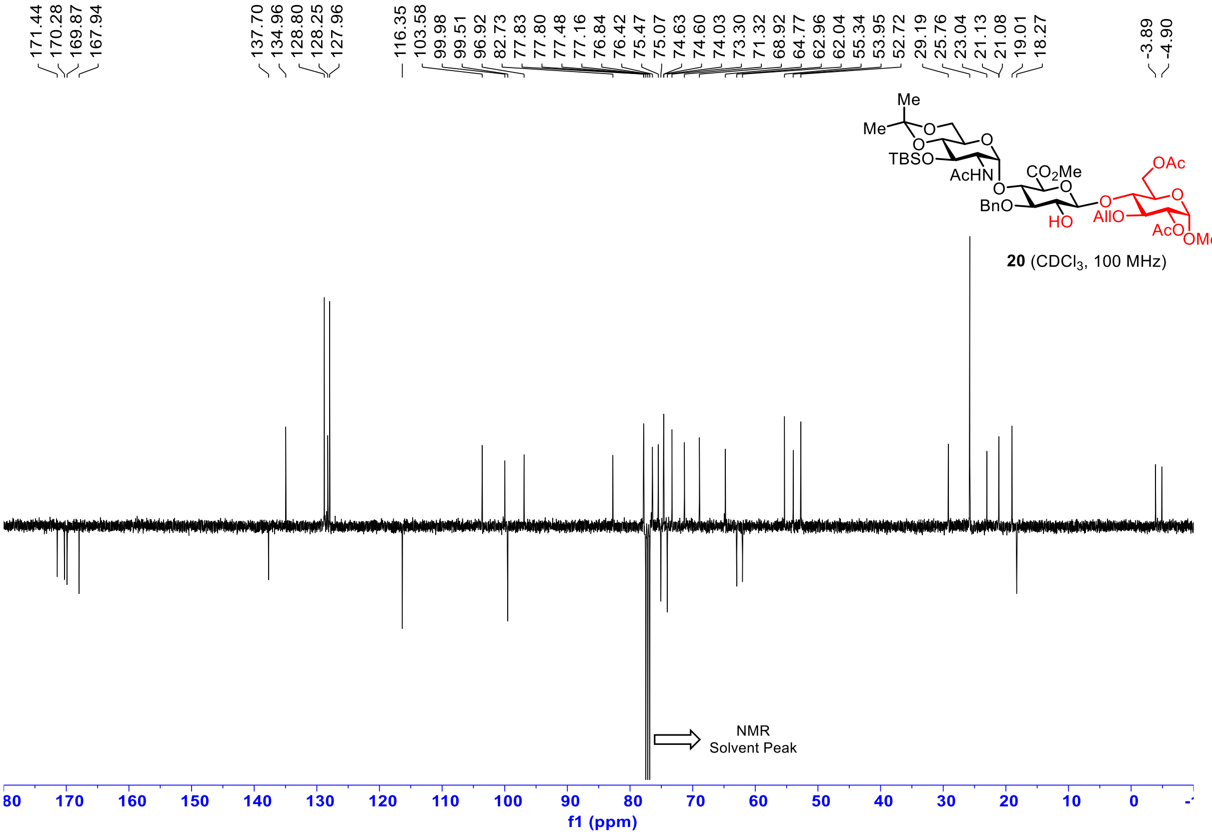
20 (CDCl3, 100 MHz)
NMR
Solvent Peak
f1 (ppm)

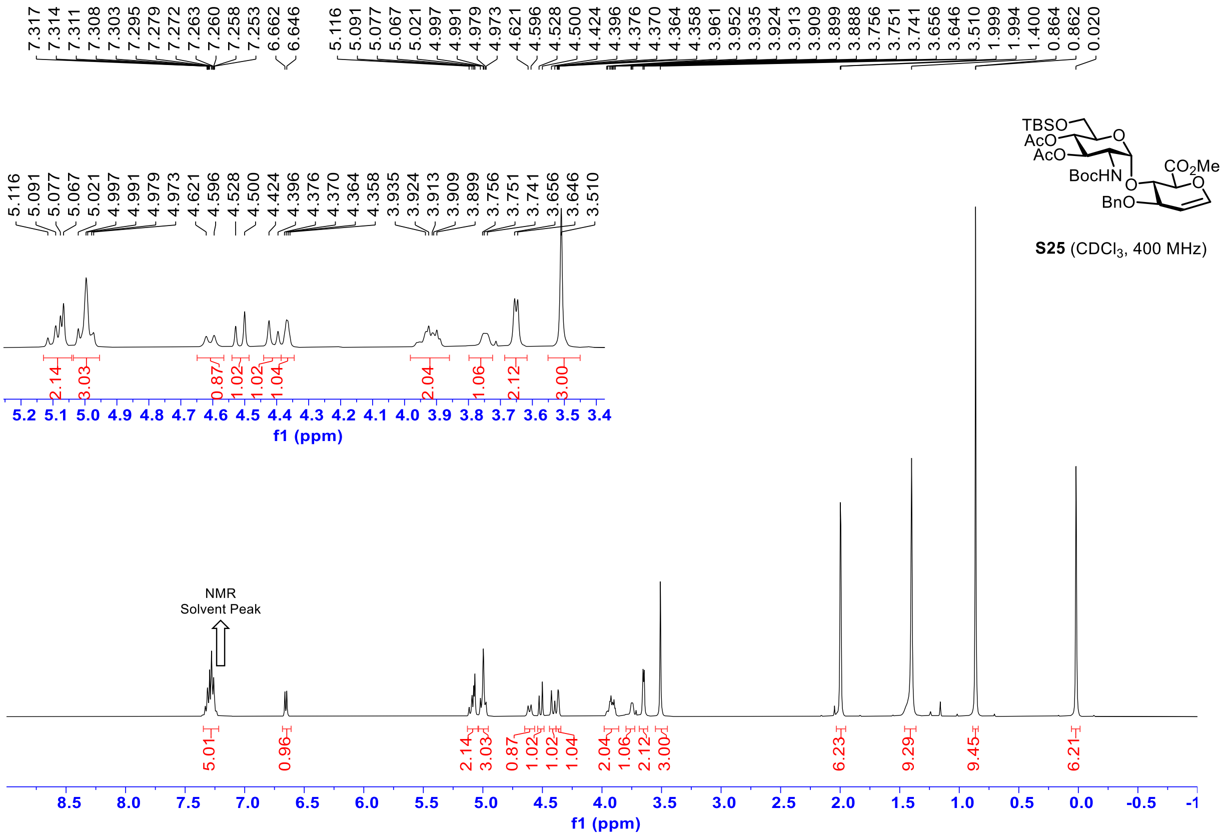

7.317
7.314
7.311
7.308
7.303
7.295
7.279
7.272
7.263
7.260
7.258
7.253
6.662
6.646
5.116
5.091
5.077
5.067
5.021
4.997
4.991
4.979
4.973
4.621
4.596
4.528
4.500
4.424
4.396
4.376
4.370
4.364
4.358
3.961
3.952
3.935
3.924
3.913
3.909
3.899
3.888
3.756
3.751
3.741
3.656
3.646
3.510
1.999
1.994
1.400
0.864
0.862
0.020
TBSO
AcO
AcO
BocHN
CO2Me
BnO
S25 (CDCl3, 400 MHz)
NMR
Solvent Peak
f1 (ppm)

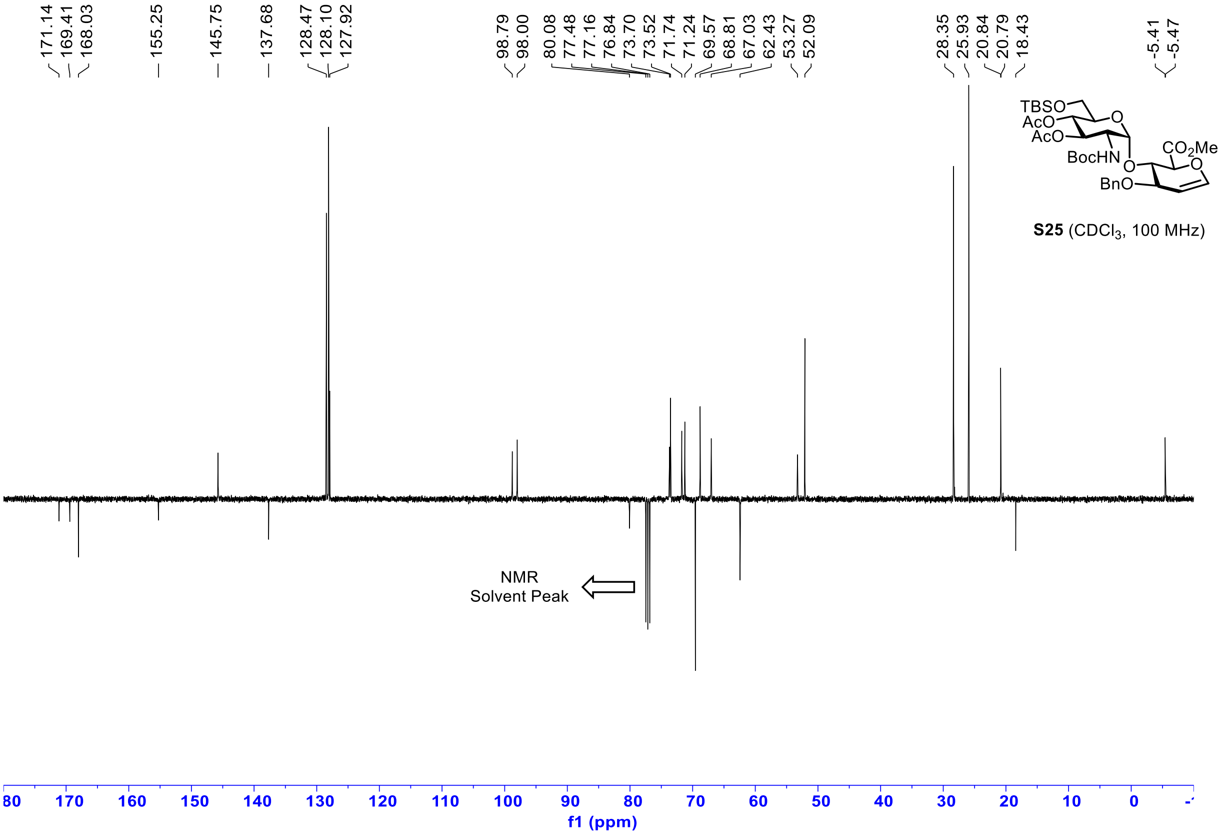

171.14
169.41
168.03
155.25
145.75
137.68
128.47
128.10
127.92
98.79
98.00
80.08
77.48
77.16
76.84
73.70
73.52
71.74
71.24
69.57
68.81
67.03
62.43
53.27
52.09
28.35
25.93
20.84
20.79
18.43
-5.41
-5.47
TBSO
AcO
AcO
BocHN
CO2Me
BnO
S25 (CDCl3, 100 MHz)
NMR
Solvent Peak
f1 (ppm)

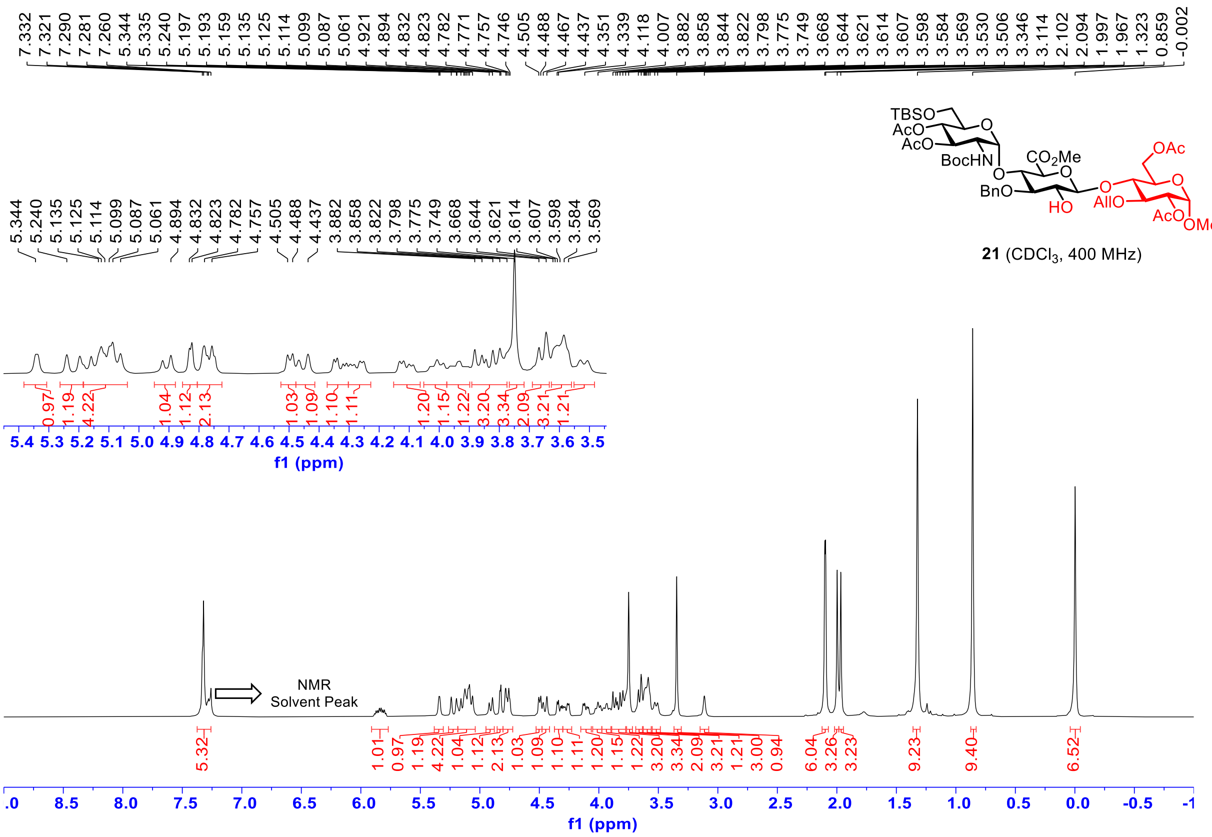
TBSO
AcO
AcO
BocHN
CO2Me
OAc
BnO
HO
AllO
AcO
OMe
21 (CDCl3, 400 MHz)
NMR
Solvent Peak
f1 (ppm)

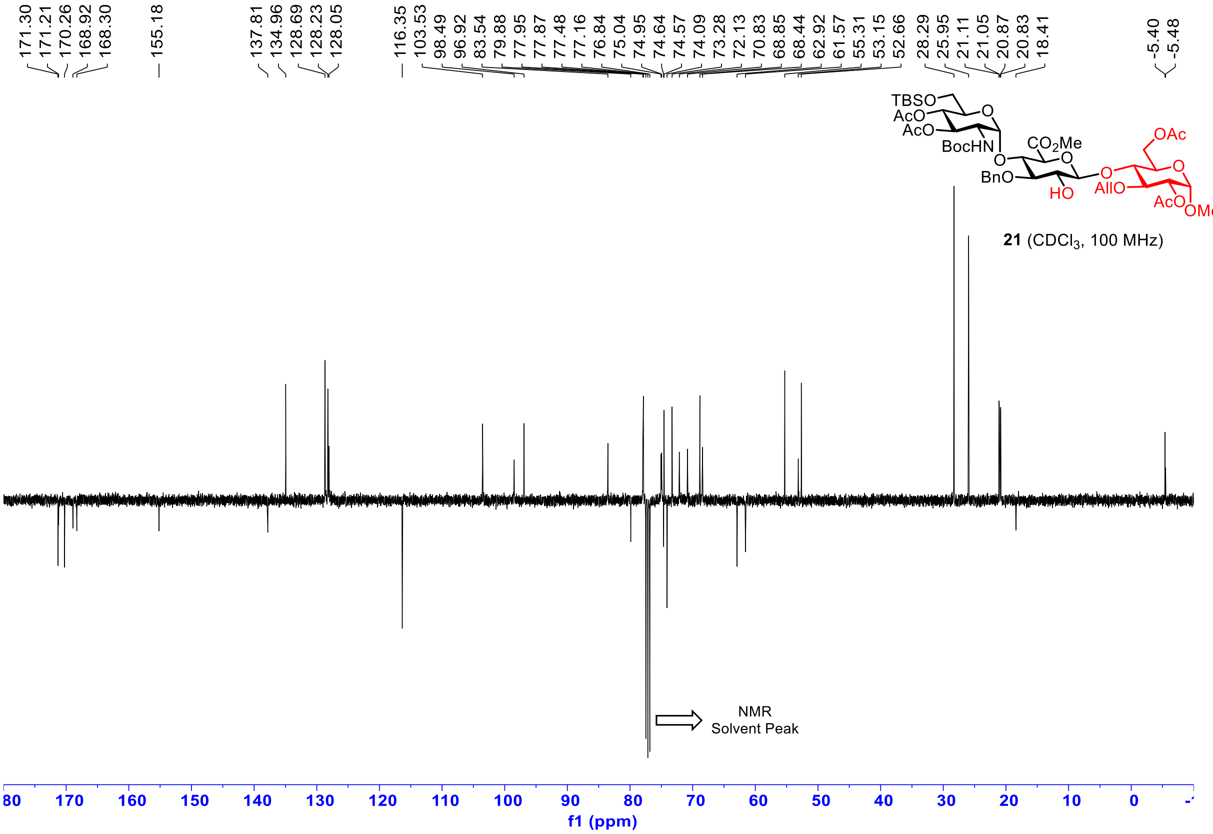
TBSO
AcO
AcO
BocHN
CO2Me
OAc
BnO
HO
AllO
AcO
OMe
21 (CDCl3, 100 MHz)
NMR
Solvent Peak
f1 (ppm)

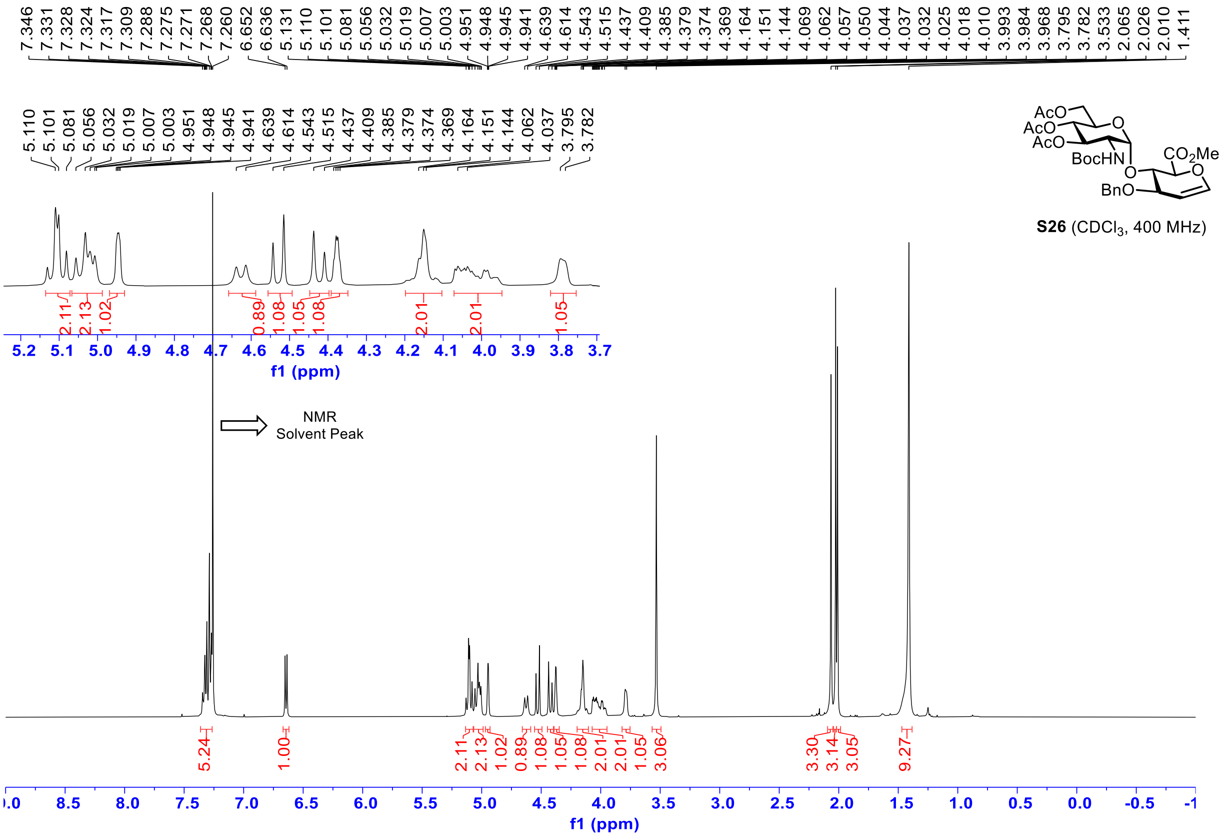

S26 (CDCl$_3$, 400 MHz)
NMR Solvent Peak
f1 (ppm)

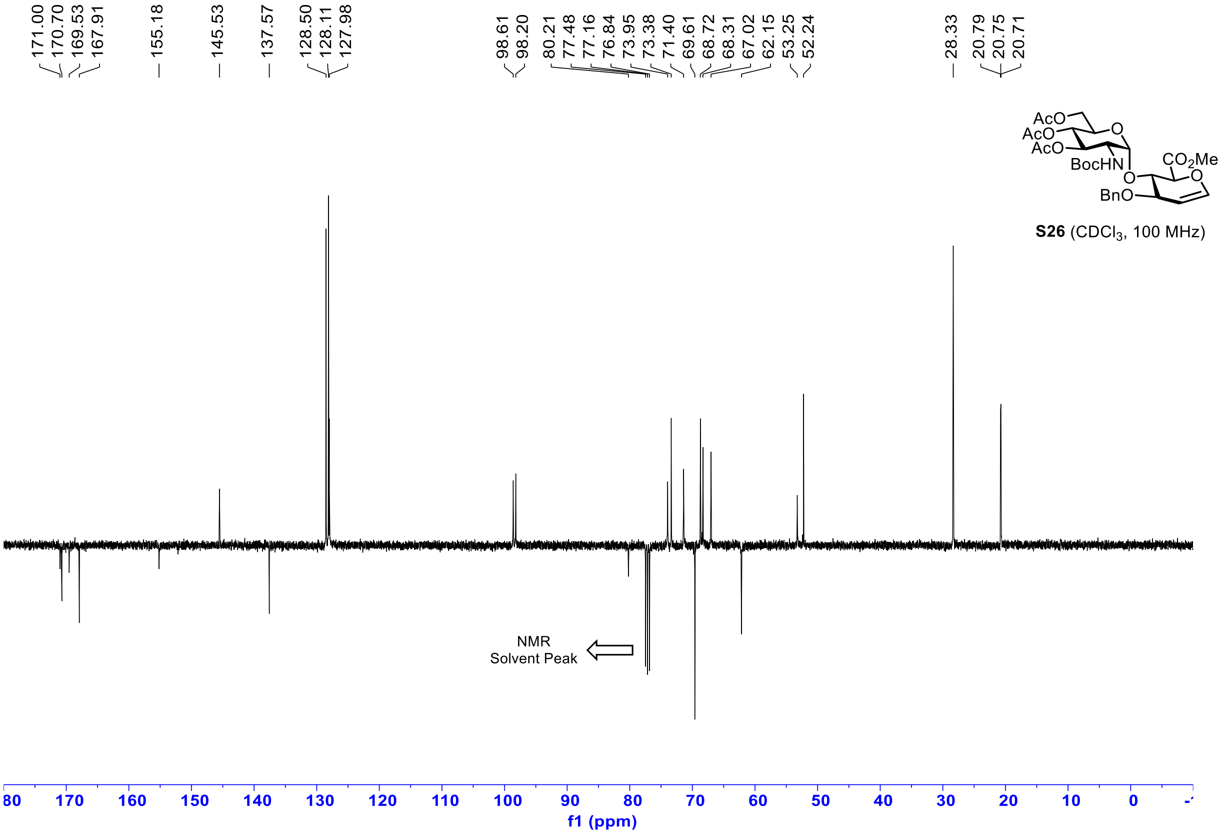

S26 (CDCl$_3$, 100 MHz)
NMR Solvent Peak
f1 (ppm)

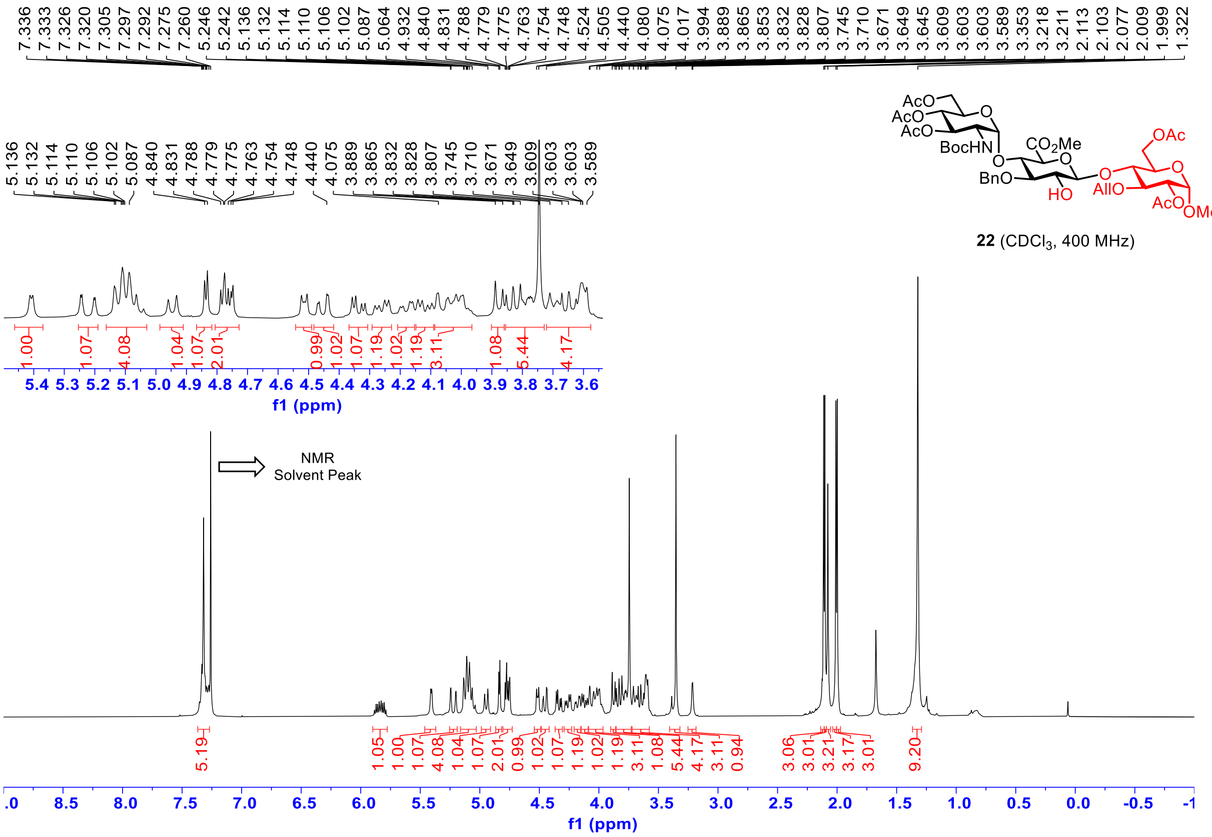

22 ($CDCl_3$, 400 MHz)

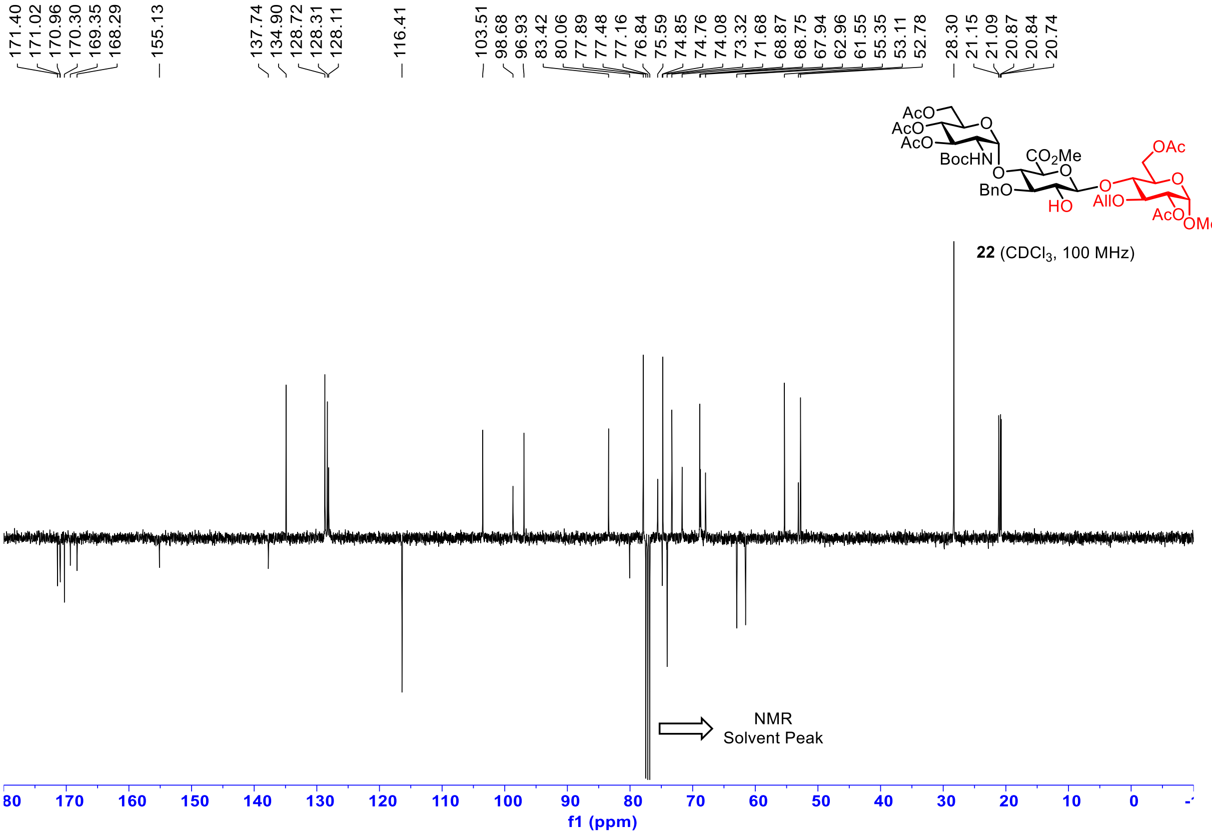

22 ($CDCl_3$, 100 MHz)

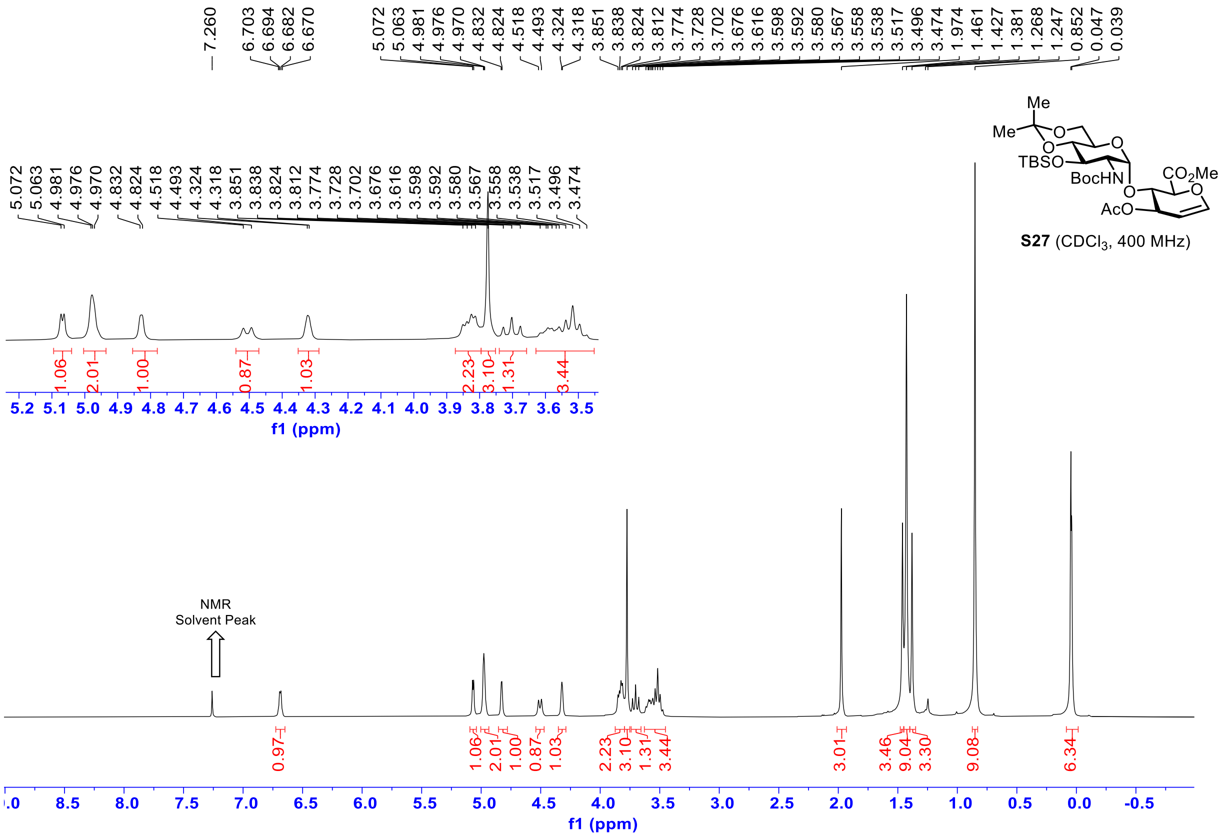
S27 (CDCl3, 400 MHz)
NMR Solvent Peak
f1 (ppm)

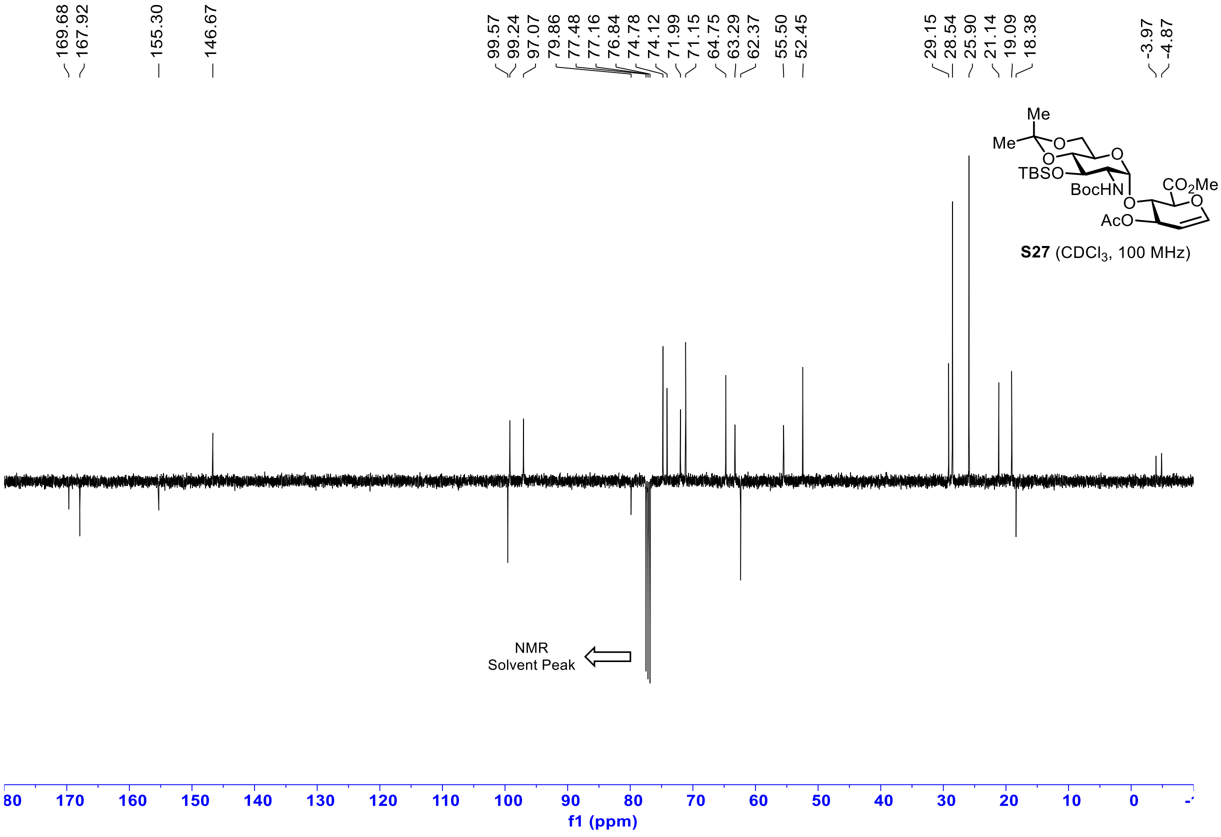
S27 (CDCl3, 100 MHz)
NMR Solvent Peak
f1 (ppm)

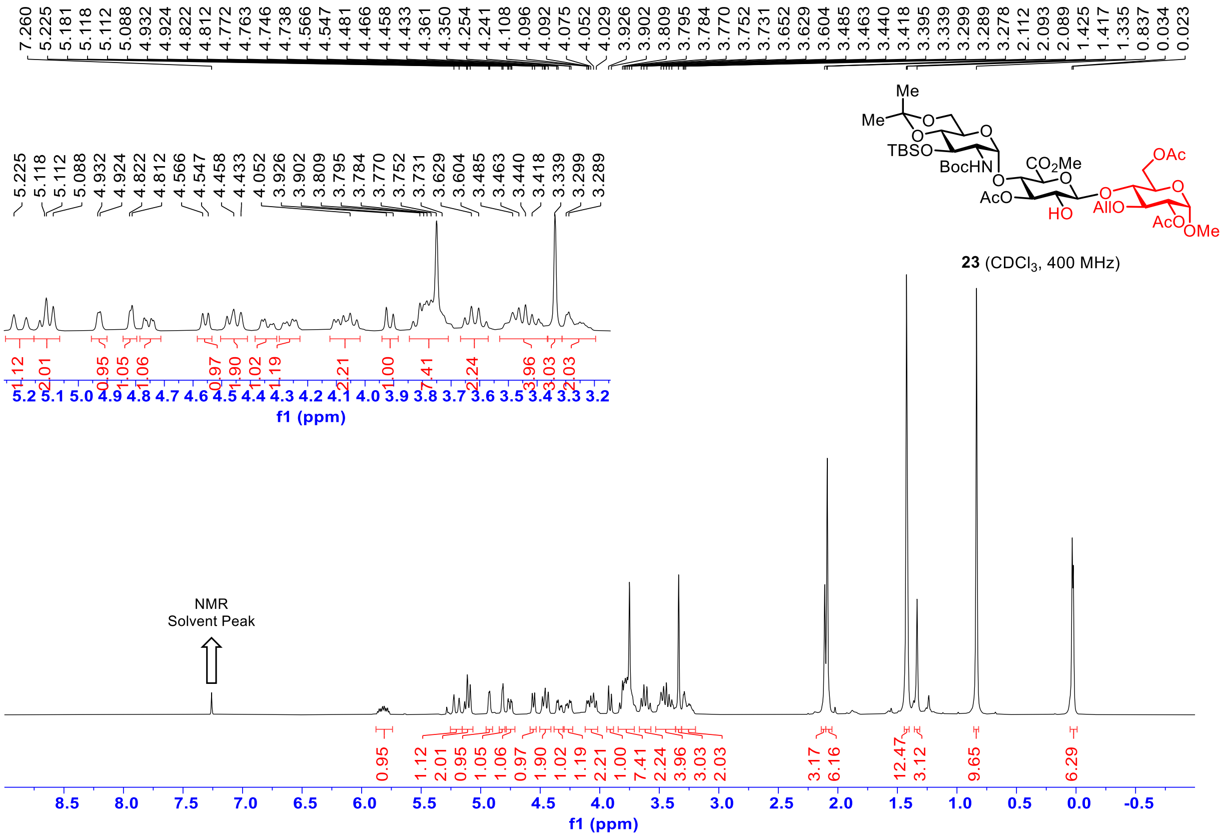
23 (CDCl3, 400 MHz)
NMR
Solvent Peak
f1 (ppm)

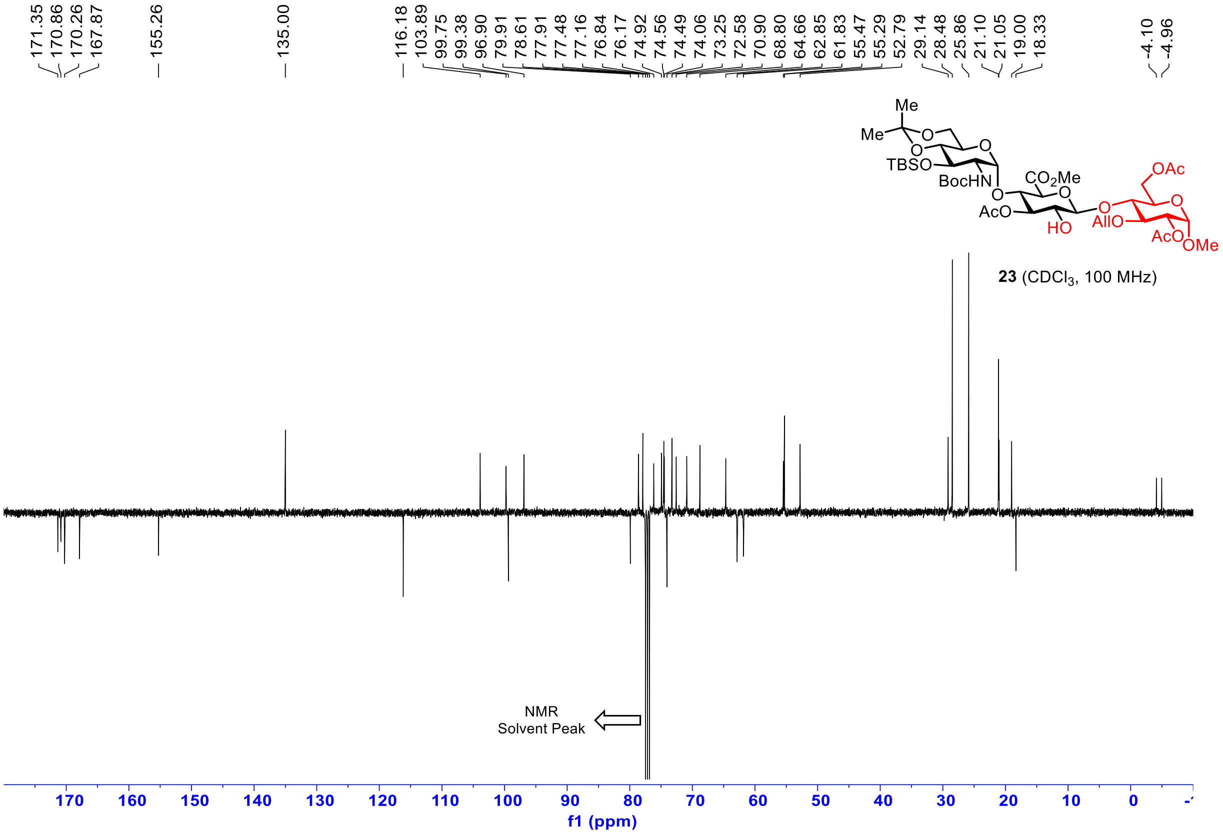
23 (CDCl3, 100 MHz)
NMR
Solvent Peak
f1 (ppm)

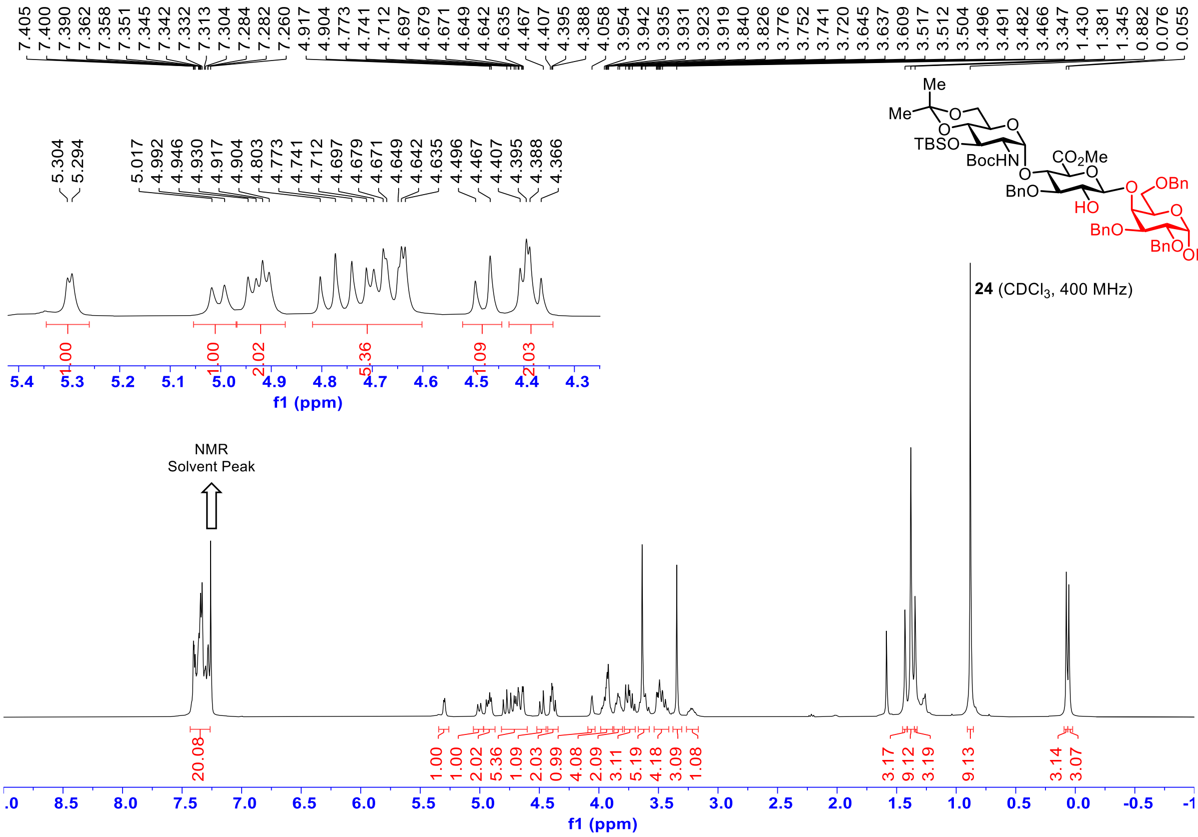

24 ($CDCl_3$, 400 MHz)

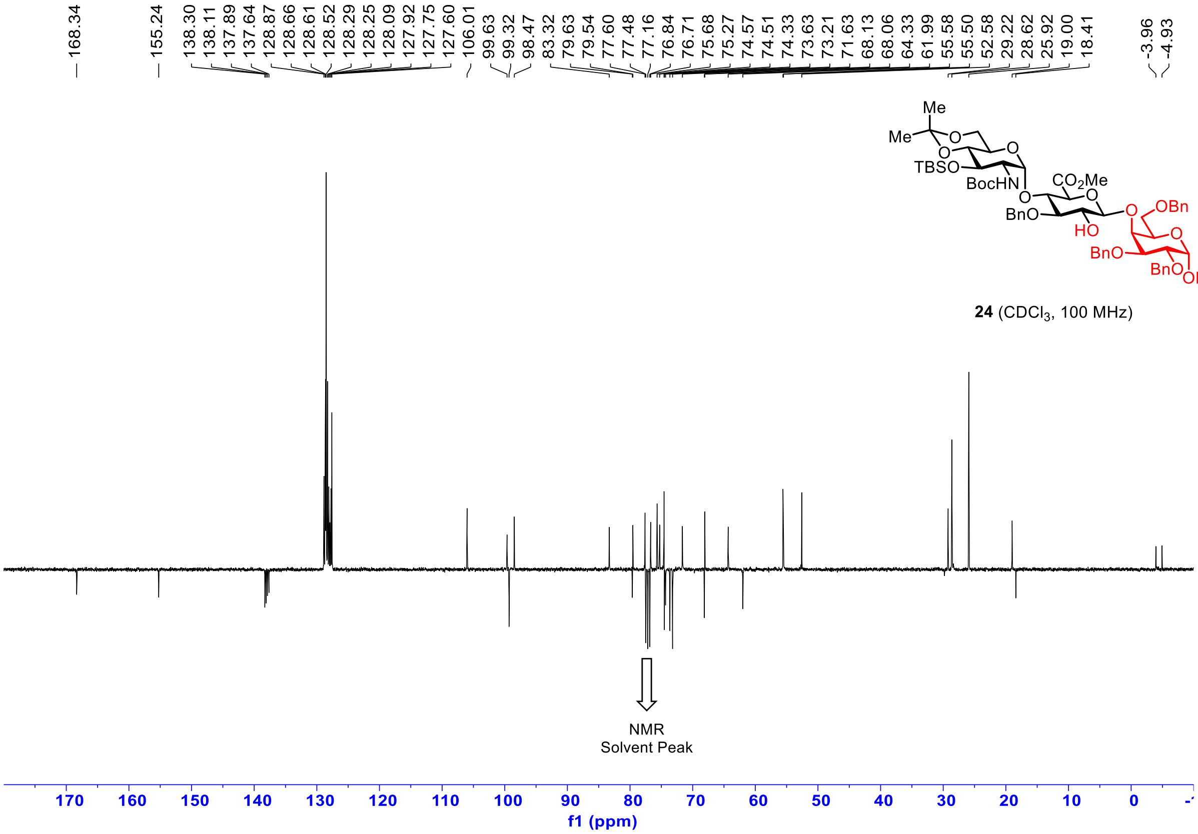

24 ($CDCl_3$, 100 MHz)

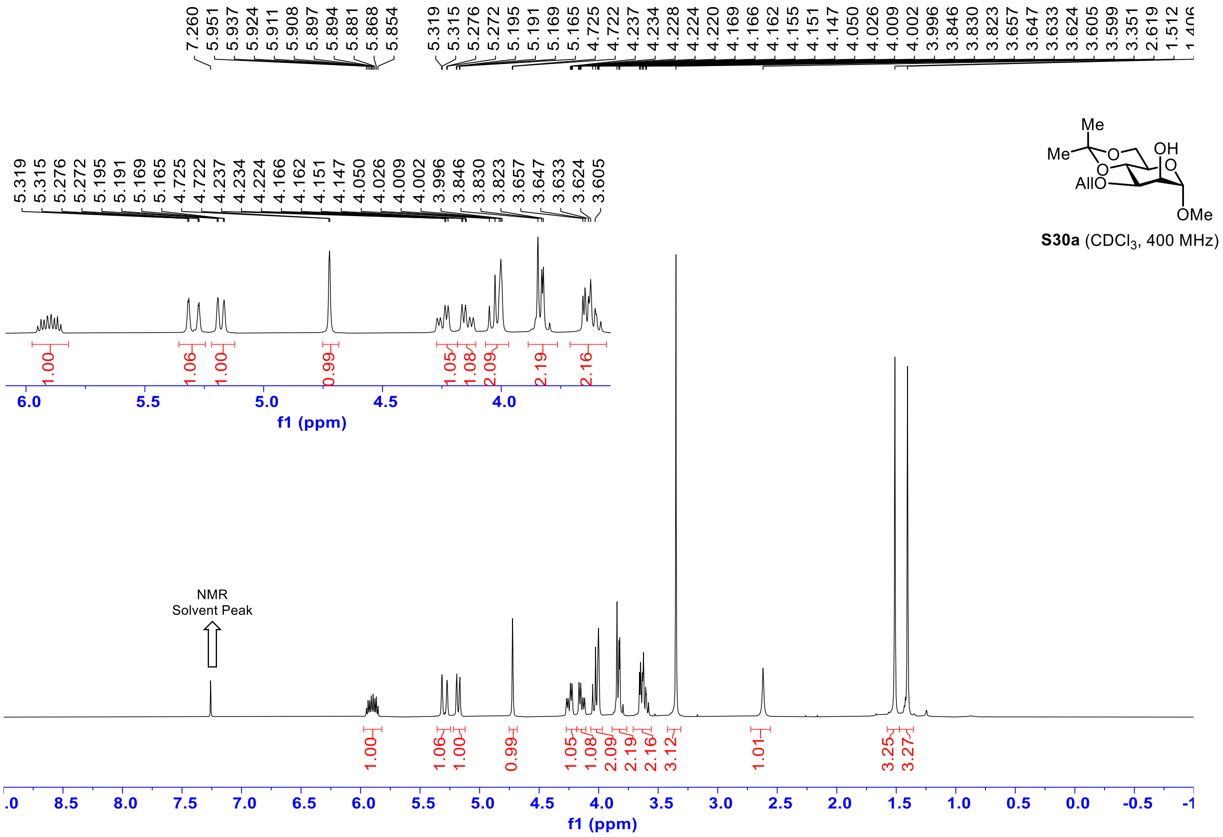
7.260
5.951
5.937
5.924
5.911
5.908
5.897
5.894
5.881
5.868
5.854
5.319
5.315
5.276
5.272
5.195
5.191
5.169
5.165
4.725
4.722
4.237
4.234
4.228
4.224
4.220
4.169
4.166
4.162
4.155
4.151
4.147
4.050
4.026
4.009
4.002
3.996
3.846
3.830
3.823
3.657
3.647
3.633
3.624
3.605
3.599
3.351
2.619
1.512
Me
Me
O
OH
O
O
AllO
OMe
S30a (CDCl3, 400 MHz)
NMR
Solvent Peak
1.00
1.06
1.00
0.99
1.05
1.08
2.09
2.19
2.16
3.12
1.01
3.25
3.27
f1 (ppm)

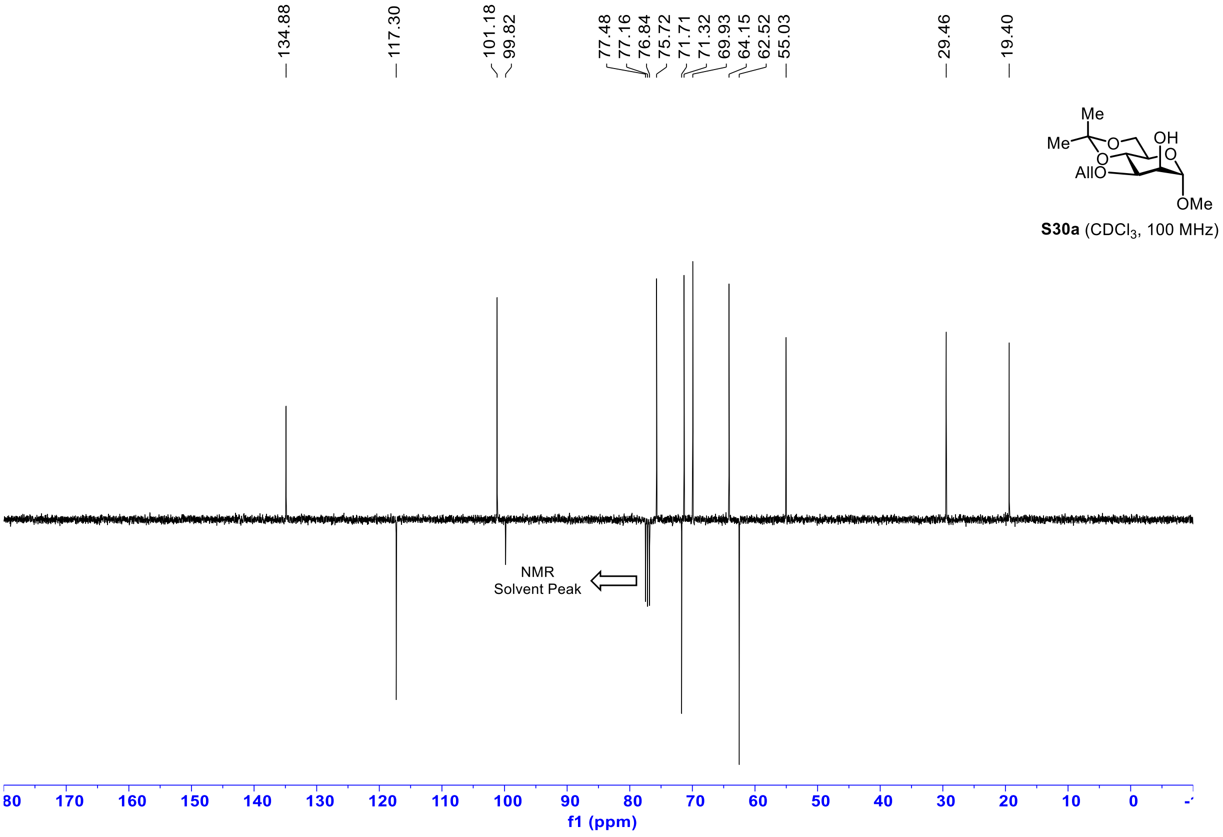
134.88
117.30
101.18
99.82
77.48
77.16
76.84
75.72
71.71
71.32
69.93
64.15
62.52
55.03
29.46
19.40
Me
Me
O
OH
O
O
AllO
OMe
S30a (CDCl3, 100 MHz)
NMR
Solvent Peak
f1 (ppm)

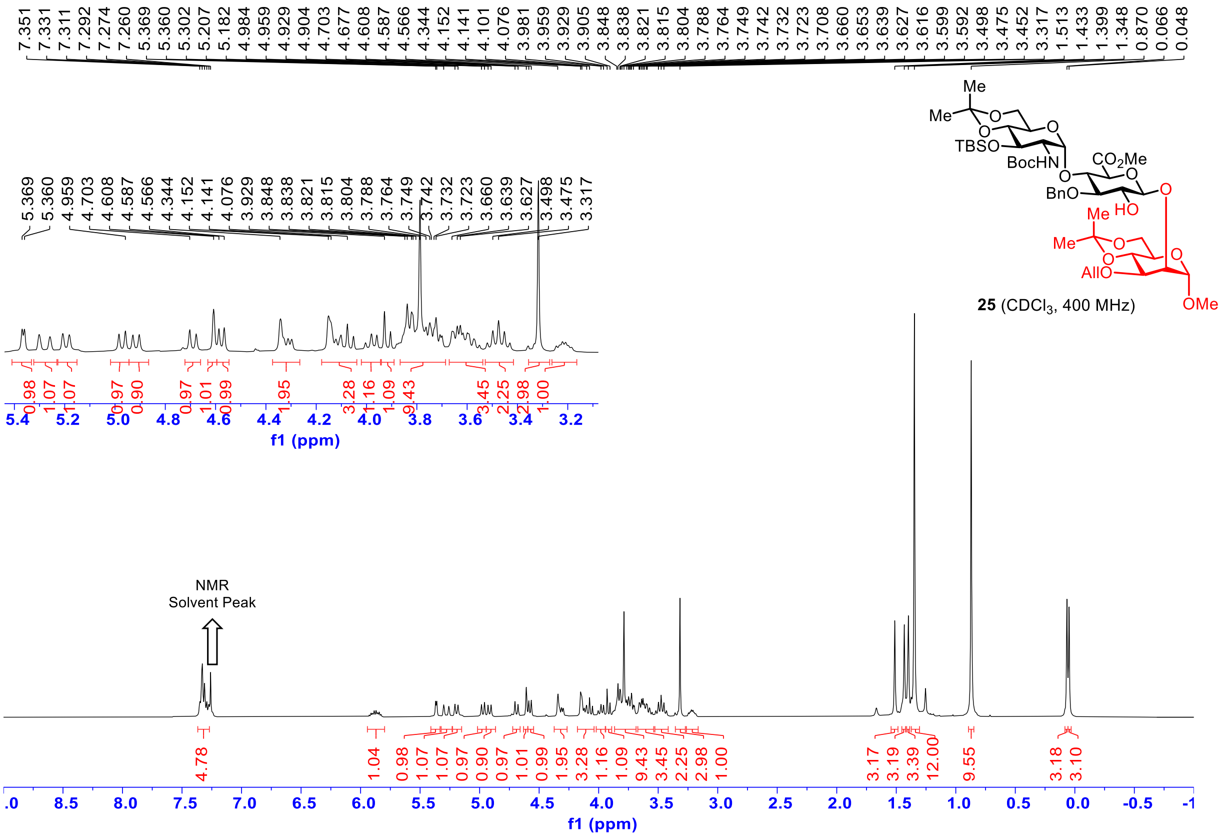
7.351 7.331 7.311 7.292 7.274 7.260 5.369 5.360 5.302 5.207 5.182 4.984 4.959 4.929 4.904 4.703 4.677 4.608 4.587 4.566 4.344 4.152 4.141 4.101 4.076 3.981 3.959 3.929 3.905 3.848 3.838 3.821 3.815 3.804 3.788 3.764 3.749 3.742 3.732 3.723 3.708 3.660 3.653 3.639 3.627 3.616 3.599 3.592 3.498 3.475 3.452 3.317 1.513 1.433 1.399 1.348 0.870 0.066 0.048
Me
Me
O
O
O
TBSO
BocHN
CO_2Me
O
BnO
HO
O
Me
Me
O
O
O
AllO
OMe
25 ($CDCl_3$, 400 MHz)
NMR
Solvent Peak
f1 (ppm)

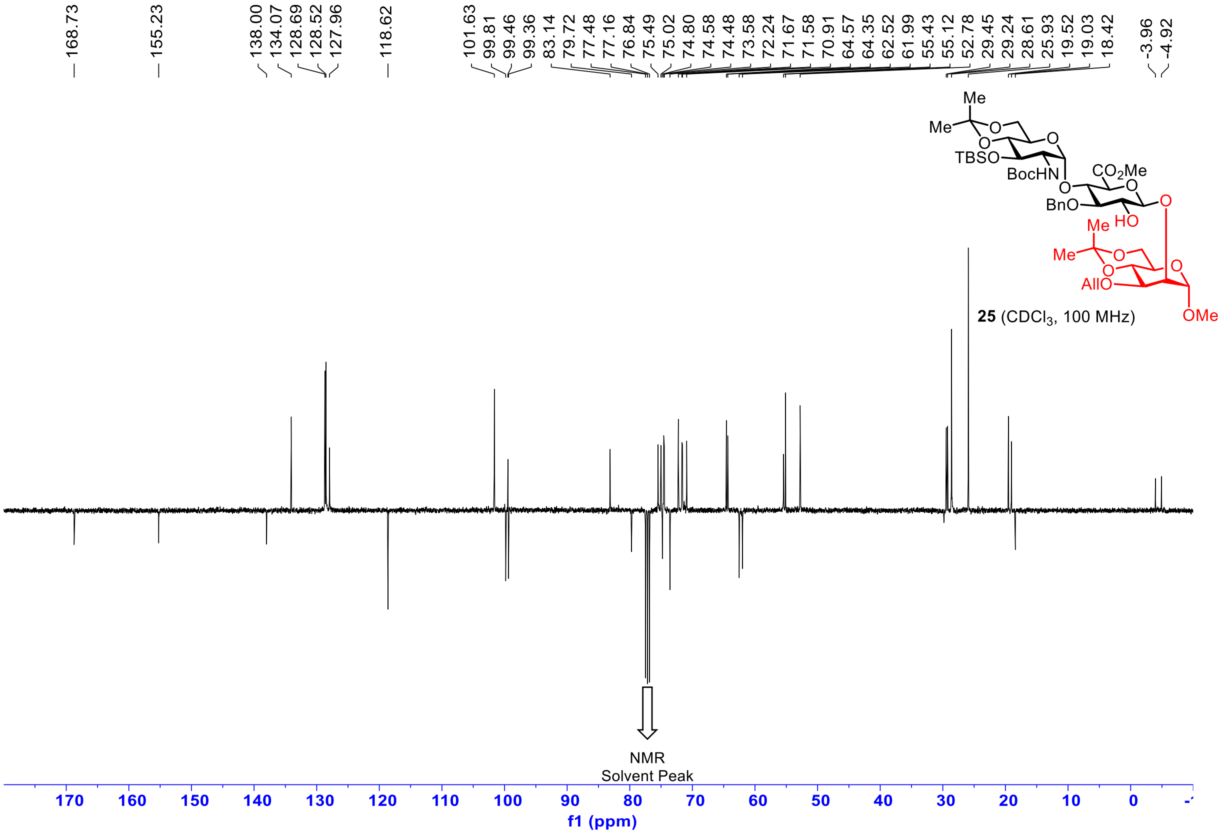
168.73 155.23 138.00 134.07 128.69 128.52 127.96 118.62 101.63 99.81 99.46 99.36 83.14 79.72 77.48 77.16 76.84 75.49 75.02 74.80 74.58 74.48 73.58 72.24 71.67 71.58 70.91 64.57 64.35 62.52 61.99 55.43 55.12 52.78 29.45 29.24 28.61 25.93 19.52 19.03 18.42 -3.96 -4.92
25 ($CDCl_3$, 100 MHz)
NMR
Solvent Peak
f1 (ppm)

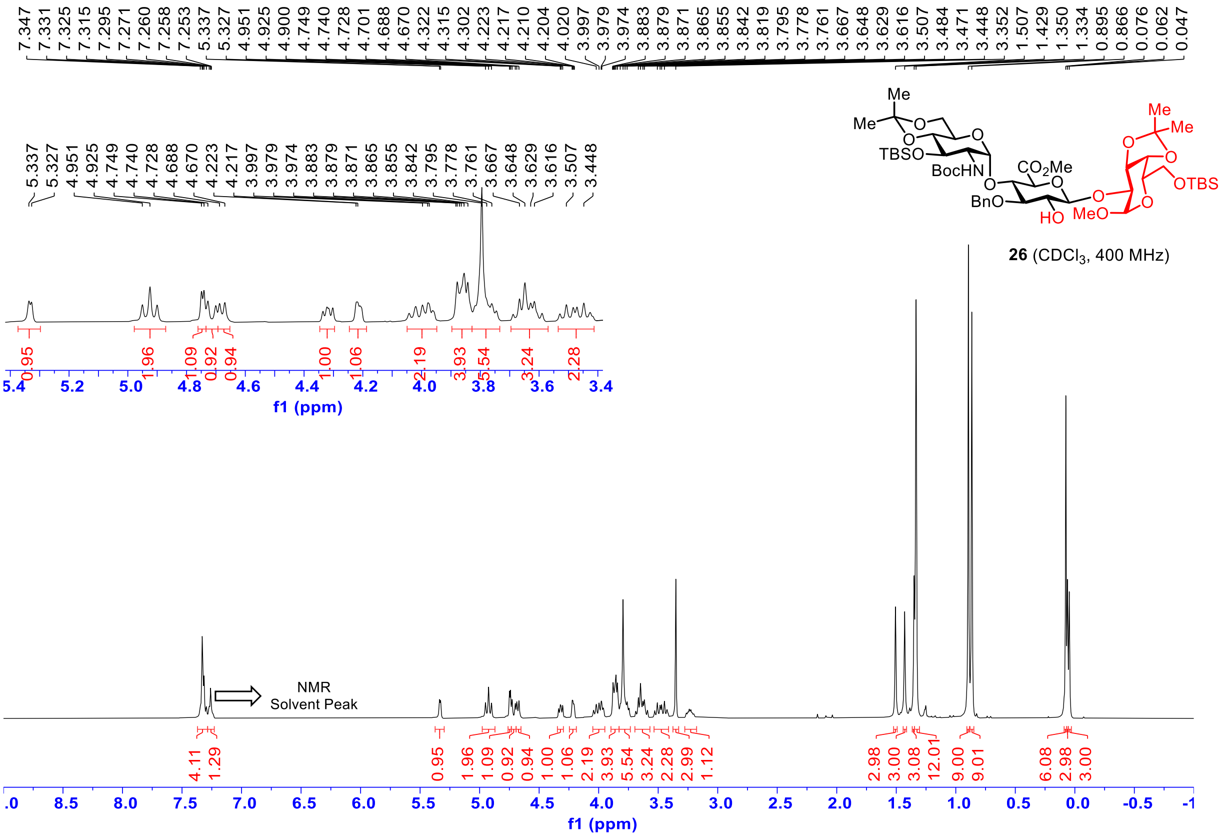

26 (CDCl3, 400 MHz)
NMR Solvent Peak
f1 (ppm)

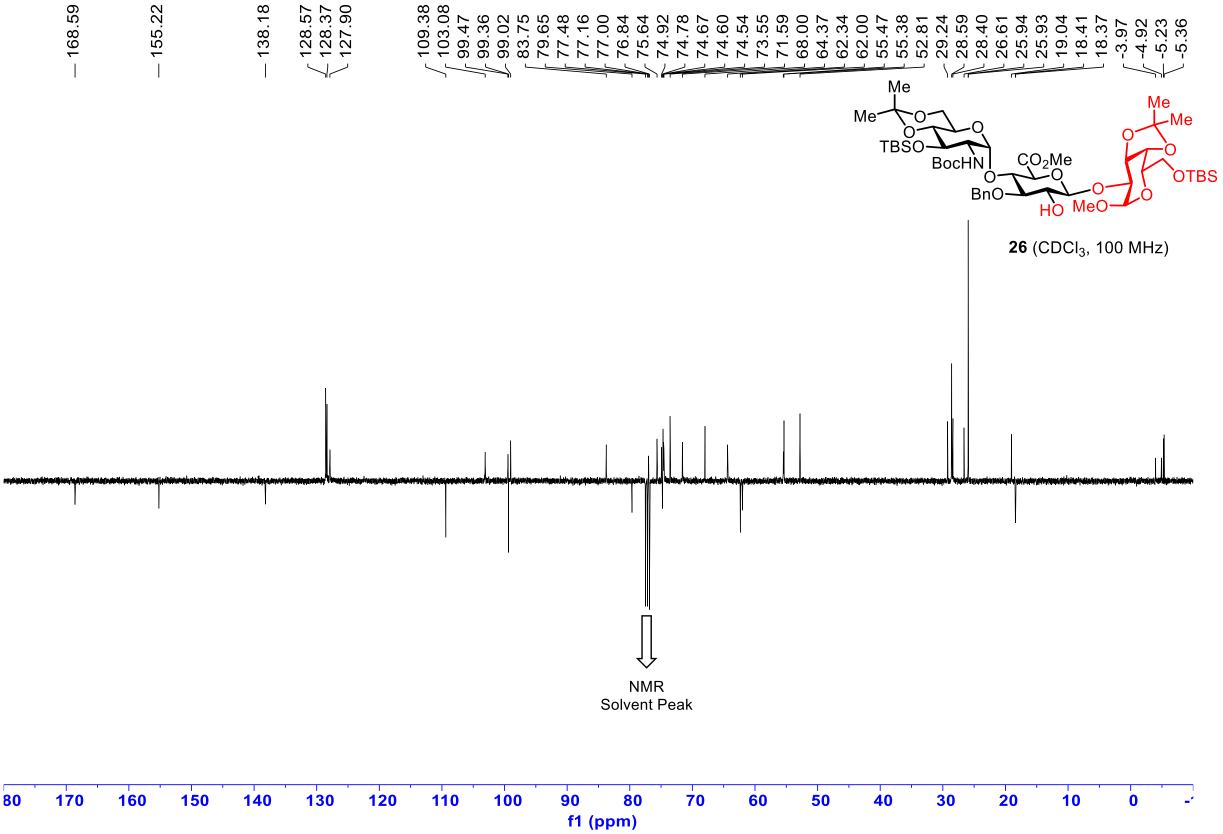

26 (CDCl3, 100 MHz)
NMR Solvent Peak
f1 (ppm)

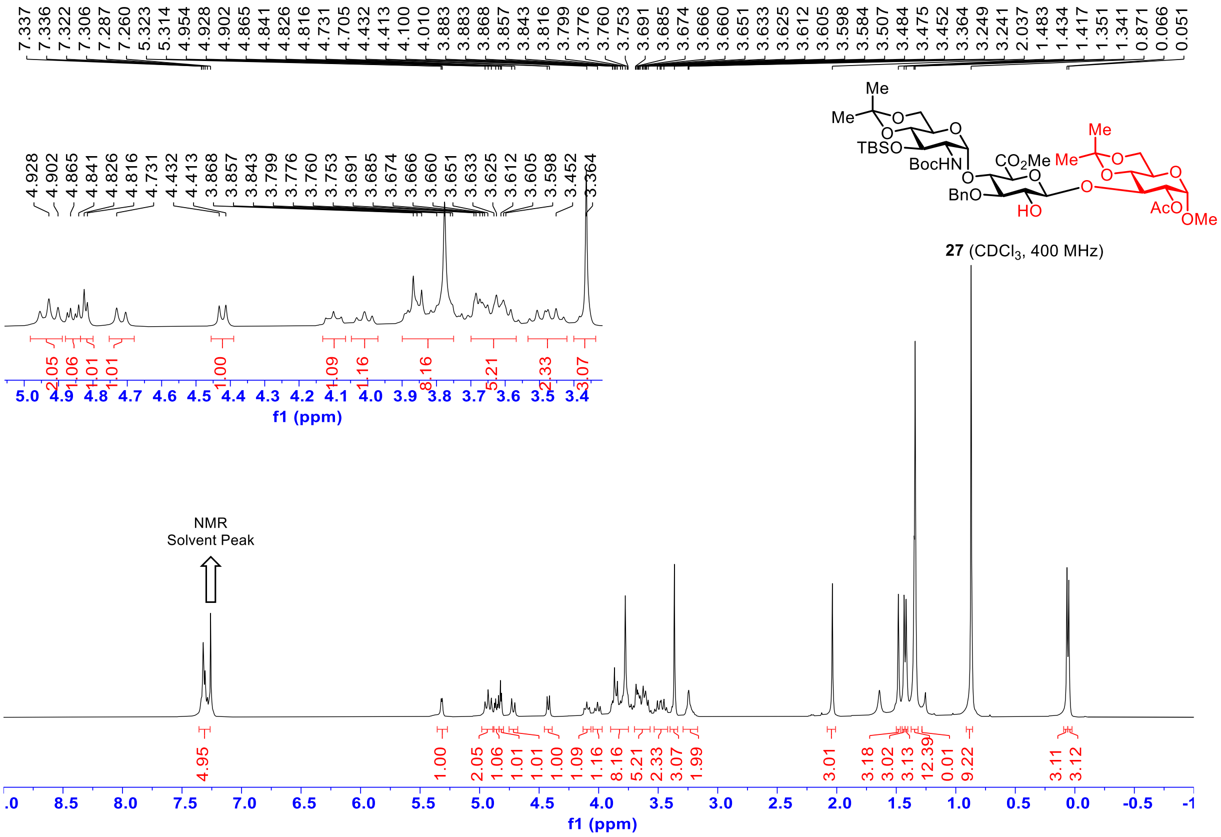
27 (CDCl$_3$, 400 MHz)
NMR Solvent Peak
f1 (ppm)

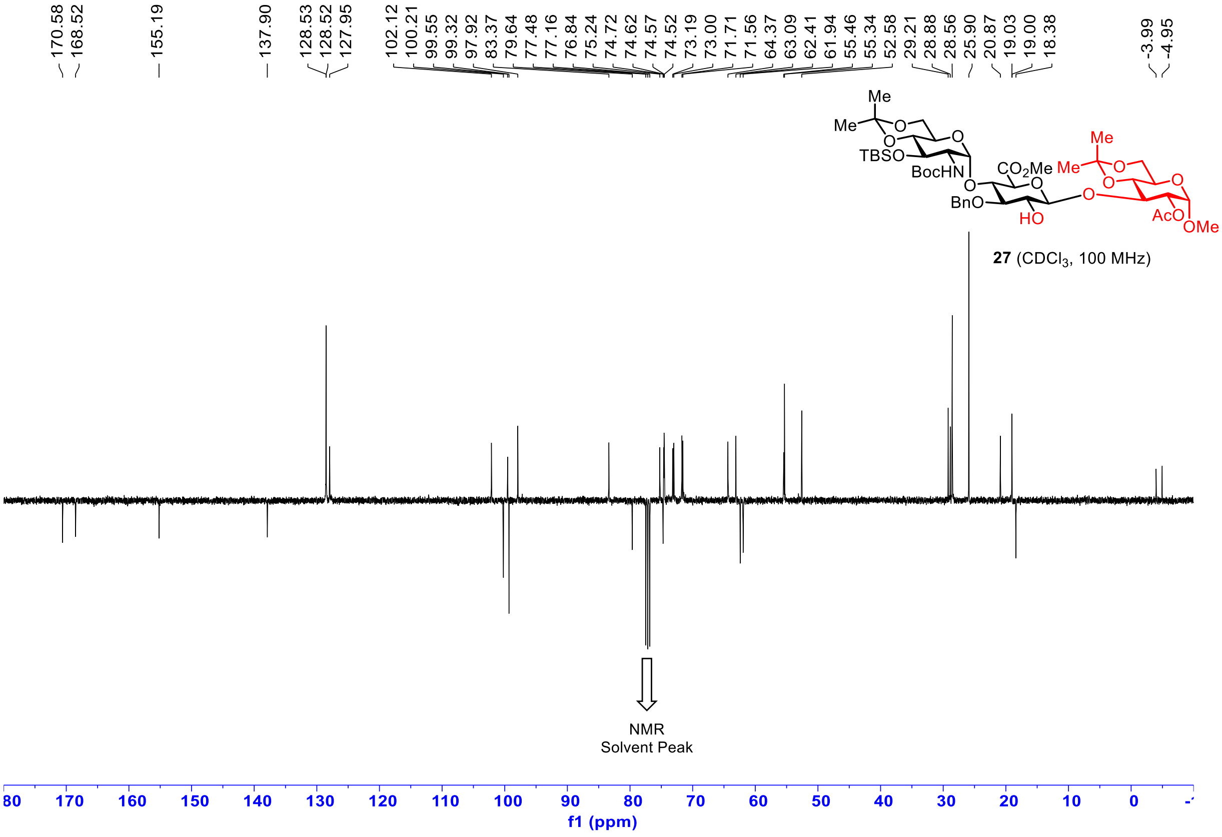
27 (CDCl$_3$, 100 MHz)
NMR Solvent Peak
f1 (ppm)

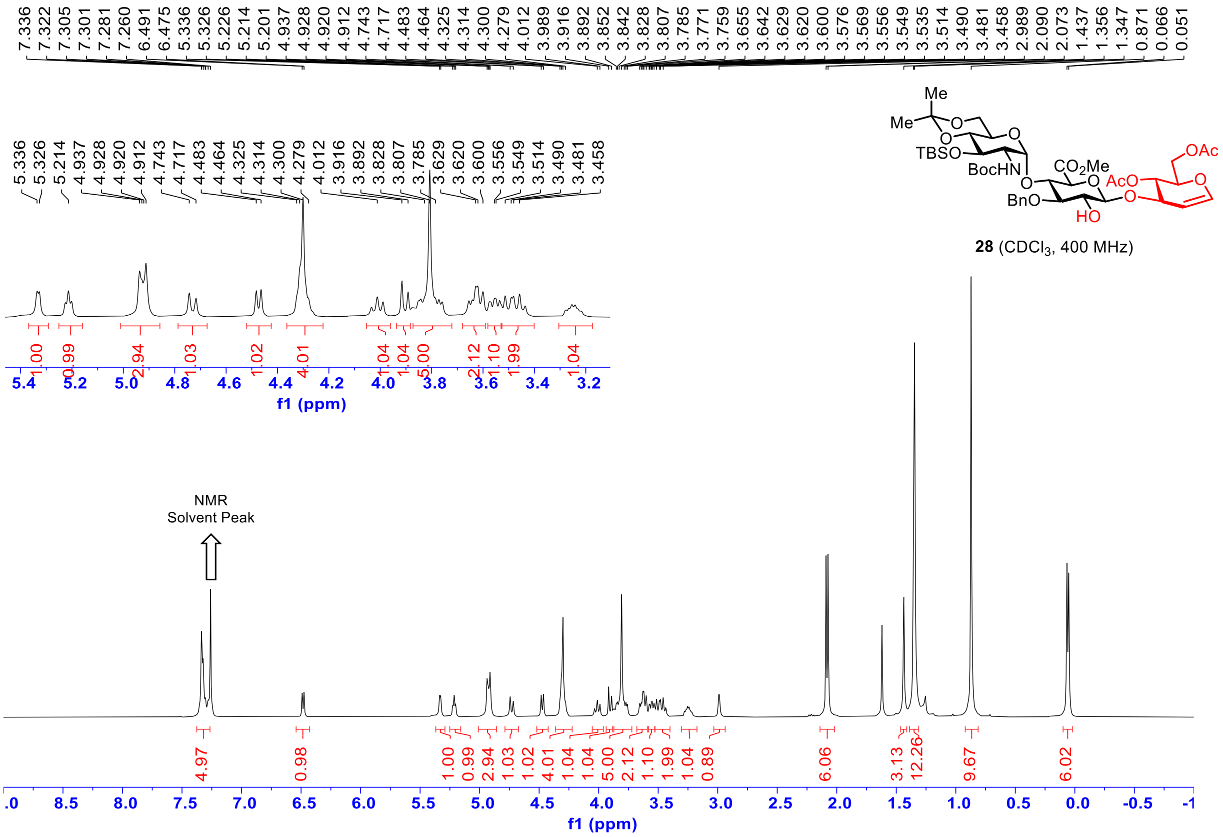

28 ($CDCl_3$, 400 MHz)

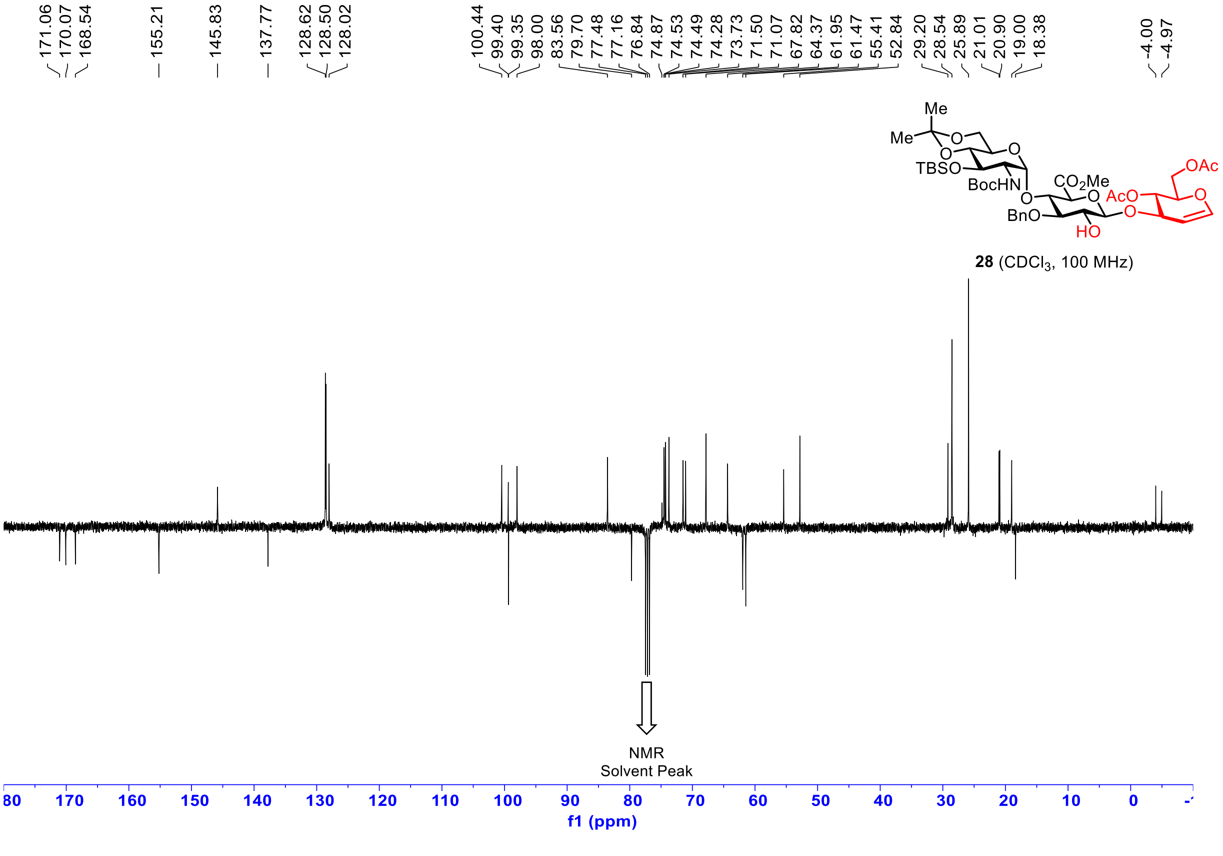

28 ($CDCl_3$, 100 MHz)

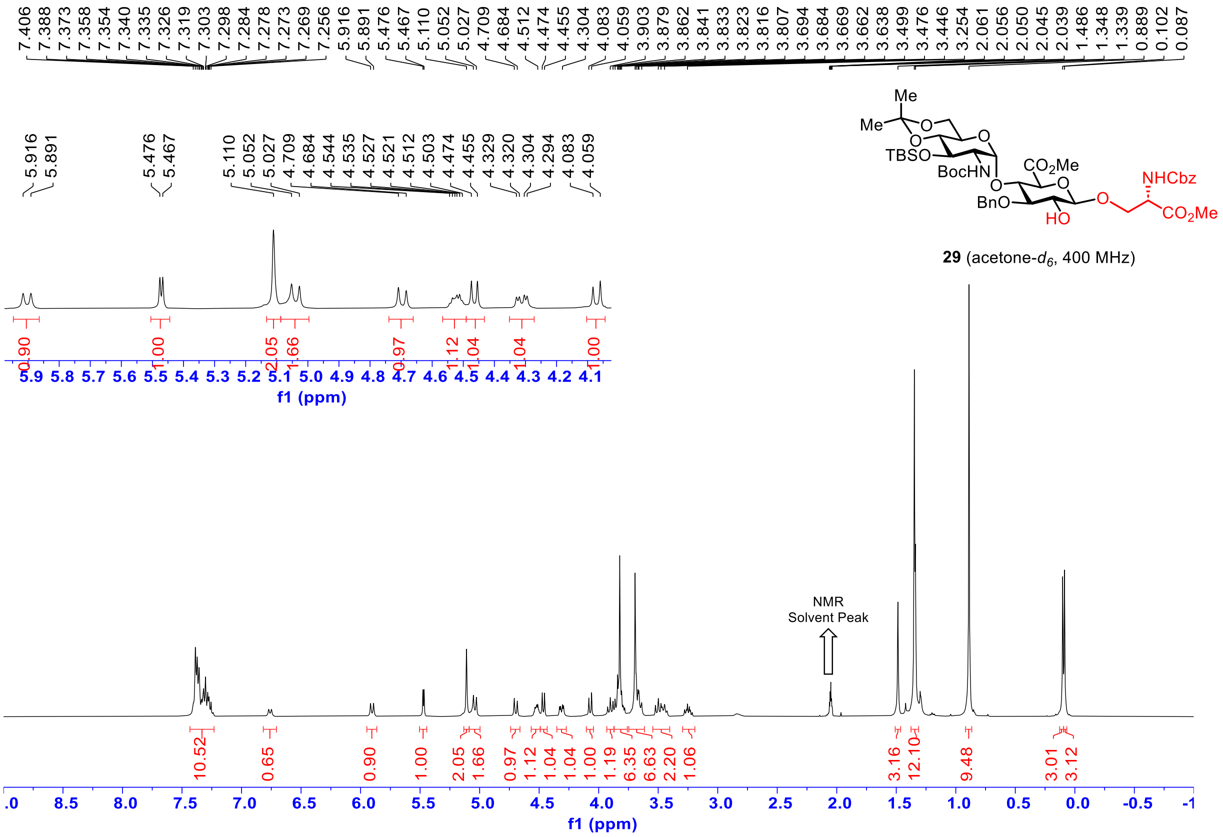

29 (acetone-d_6, 400 MHz)

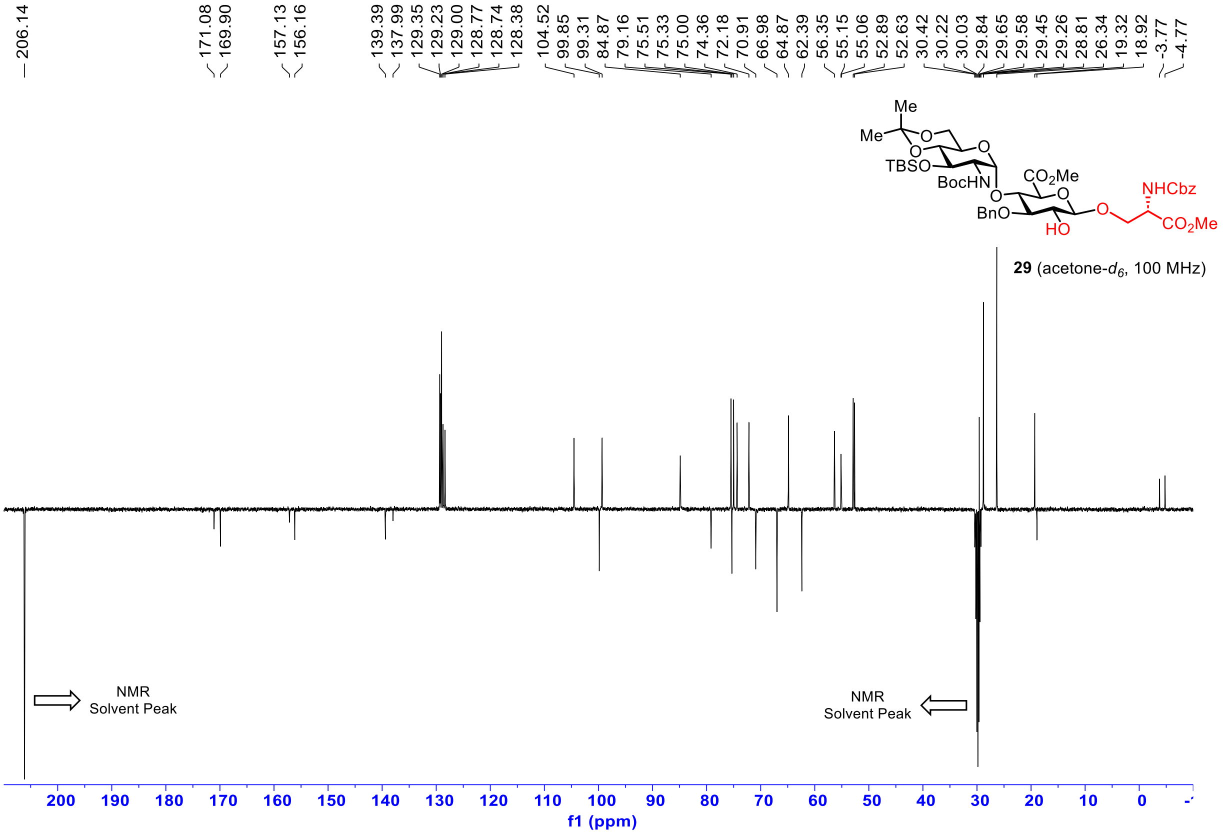

29 (acetone-d_6, 100 MHz)

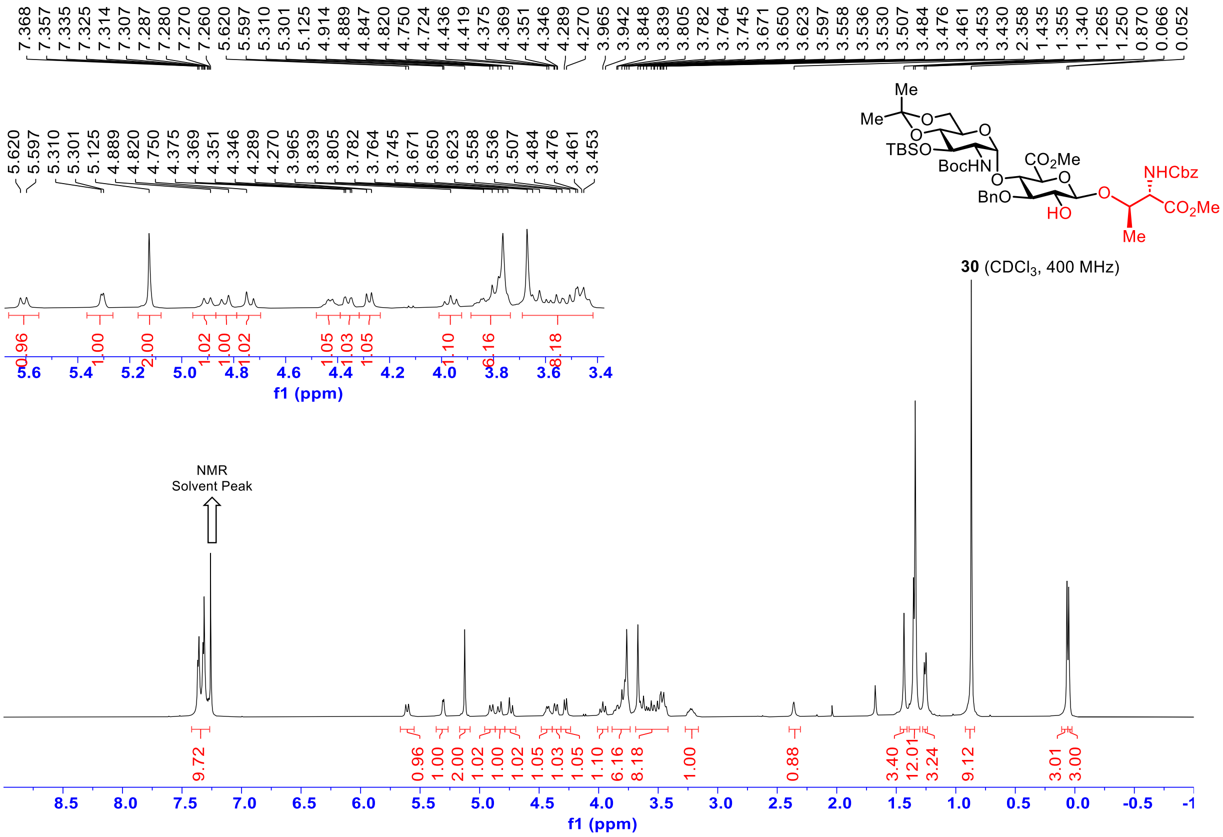

30 ($CDCl_3$, 400 MHz)

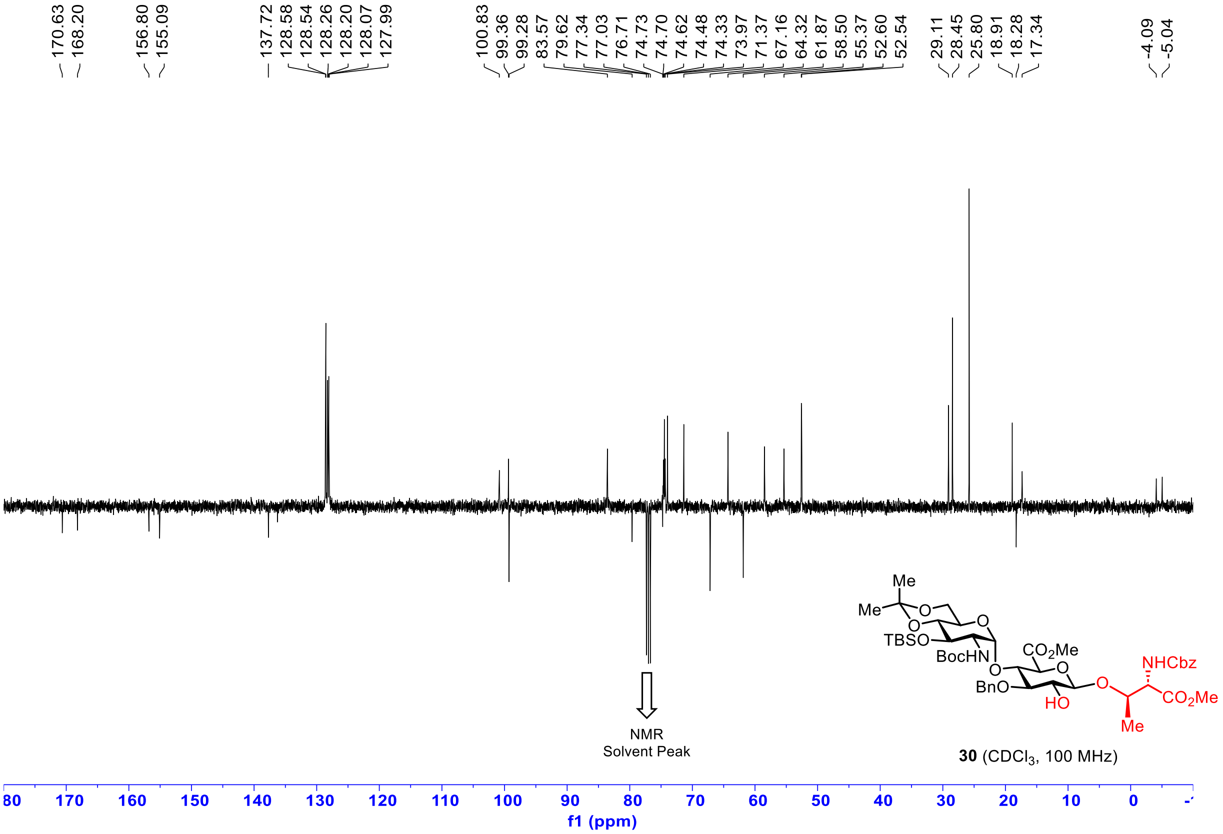

30 ($CDCl_3$, 100 MHz)

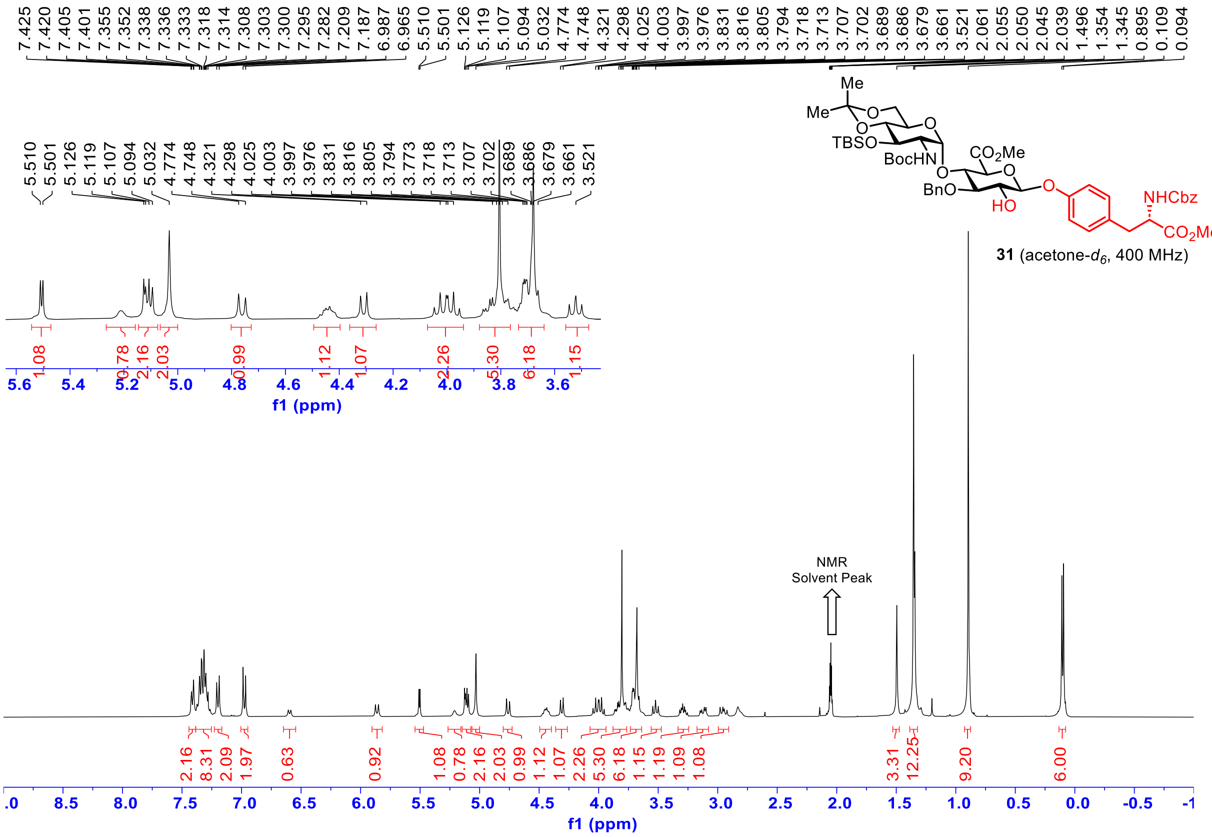
7.425 7.420 7.405 7.401 7.355 7.352 7.338 7.336 7.333 7.318 7.314 7.308 7.303 7.300 7.295 7.282 7.209 7.187 6.987 6.965 5.510 5.501 5.126 5.119 5.107 5.094 5.032 4.774 4.748 4.321 4.298 4.025 4.003 3.997 3.976 3.831 3.816 3.805 3.794 3.718 3.713 3.707 3.702 3.689 3.686 3.679 3.661 3.521 2.061 2.055 2.050 2.045 2.039 1.496 1.354 1.345 0.895 0.109 0.094
5.510 5.501 5.126 5.119 5.107 5.094 5.032 4.774 4.748 4.321 4.298 4.025 4.003 3.997 3.976 3.831 3.816 3.805 3.794 3.773 3.718 3.713 3.707 3.702 3.689 3.686 3.679 3.661 3.521
1.08 0.78 2.16 2.03 0.99 1.12 1.07 2.26 5.30 6.18 1.15
5.6 5.4 5.2 5.0 4.8 4.6 4.4 4.2 4.0 3.8 3.6
f1 (ppm)
Me
Me
TBSO
BocHN
CO2Me
BnO
HO
NHCbz
CO2Me
31 (acetone-d6, 400 MHz)
NMR Solvent Peak
2.16 8.31 2.09 1.97 0.63 0.92 1.08 0.78 2.16 2.03 0.99 1.12 1.07 2.26 5.30 6.18 1.15 1.19 1.09 1.08 3.31 12.25 9.20 6.00
.0 8.5 8.0 7.5 7.0 6.5 6.0 5.5 5.0 4.5 4.0 3.5 3.0 2.5 2.0 1.5 1.0 0.5 0.0 -0.5 -1
f1 (ppm)

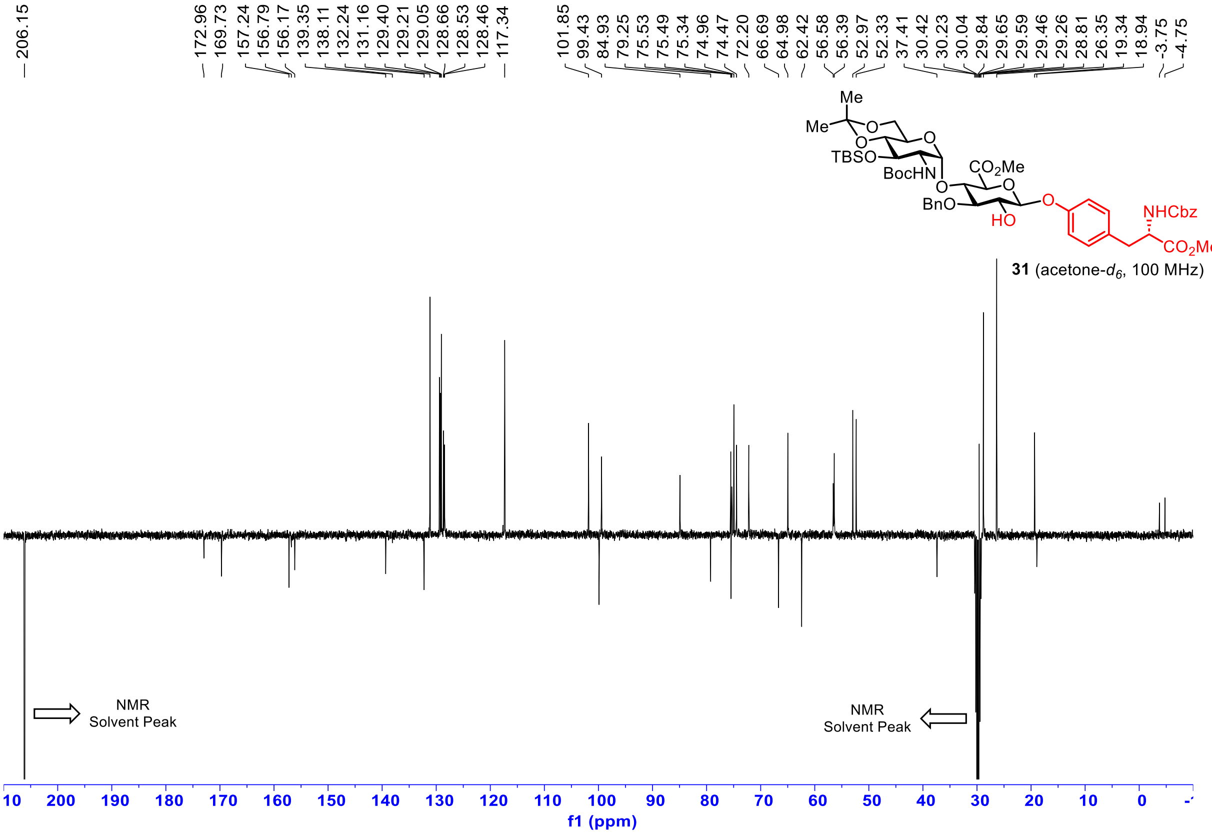
206.15 172.96 169.73 157.24 156.79 156.17 139.35 138.11 132.24 131.16 129.40 129.21 129.05 128.66 128.53 128.46 117.34 101.85 99.43 84.93 79.25 75.53 75.49 75.34 74.96 74.47 72.20 66.69 64.98 62.42 56.58 56.39 52.97 52.33 37.41 30.42 30.23 30.04 29.84 29.65 29.59 29.46 29.26 28.81 26.35 19.34 18.94 -3.75 -4.75
Me
Me
TBSO
BocHN
CO2Me
BnO
HO
NHCbz
CO2Me
31 (acetone-d6, 100 MHz)
NMR Solvent Peak
NMR Solvent Peak
10 200 190 180 170 160 150 140 130 120 110 100 90 80 70 60 50 40 30 20 10 0
f1 (ppm)

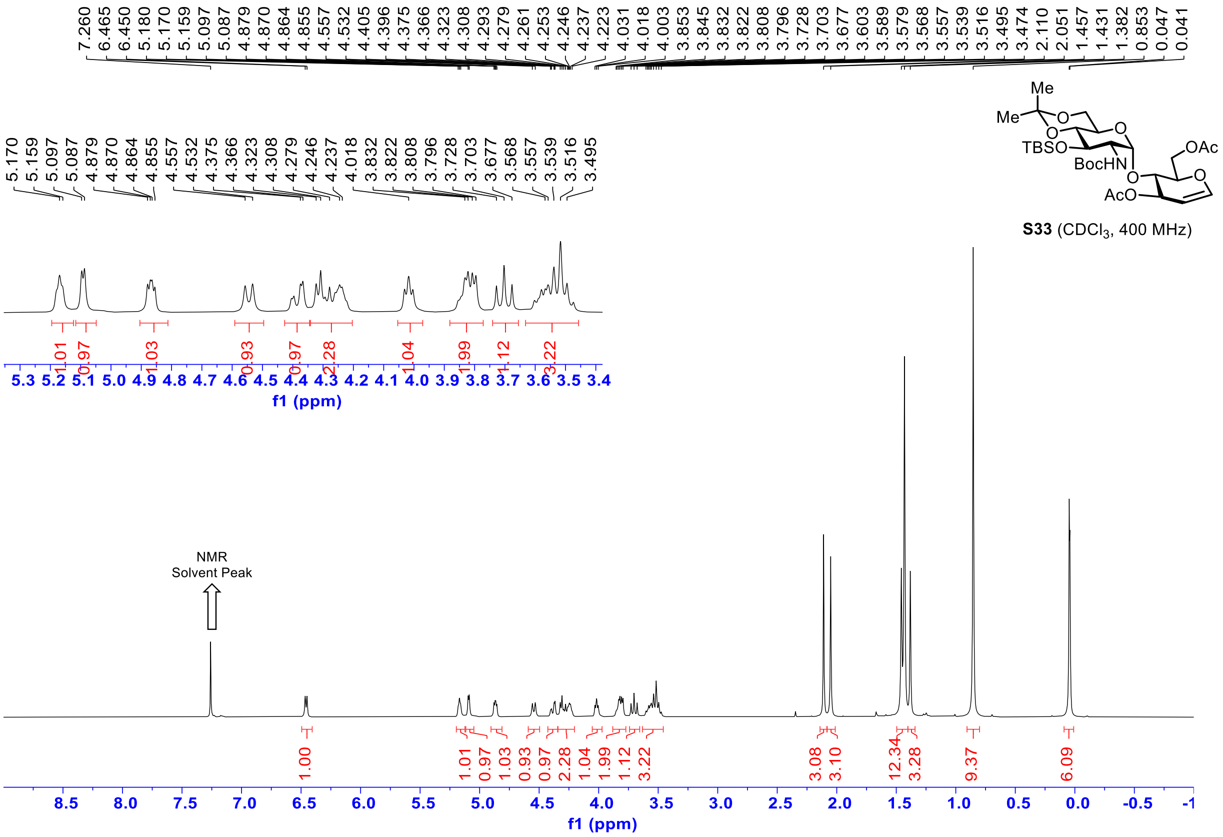
S33 (CDCl3, 400 MHz)
NMR
Solvent Peak
f1 (ppm)

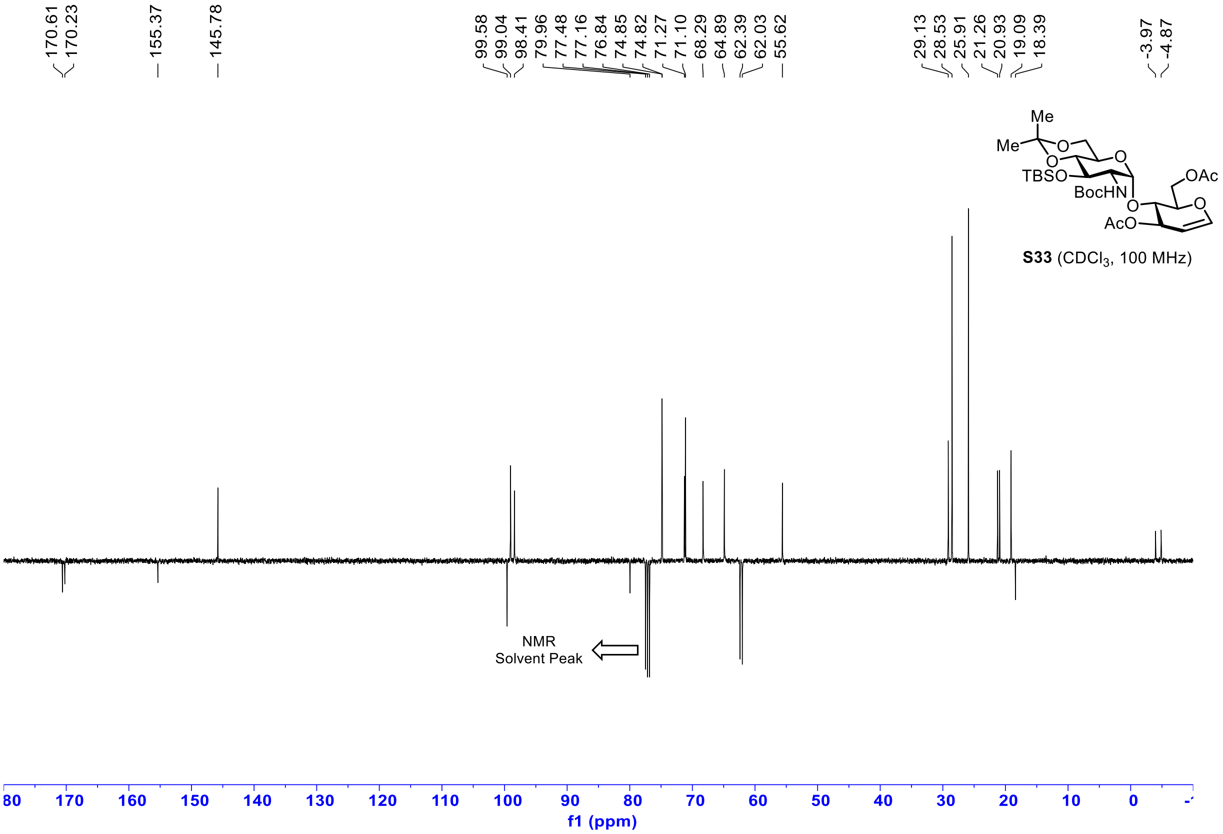
S33 (CDCl3, 100 MHz)
NMR
Solvent Peak
f1 (ppm)

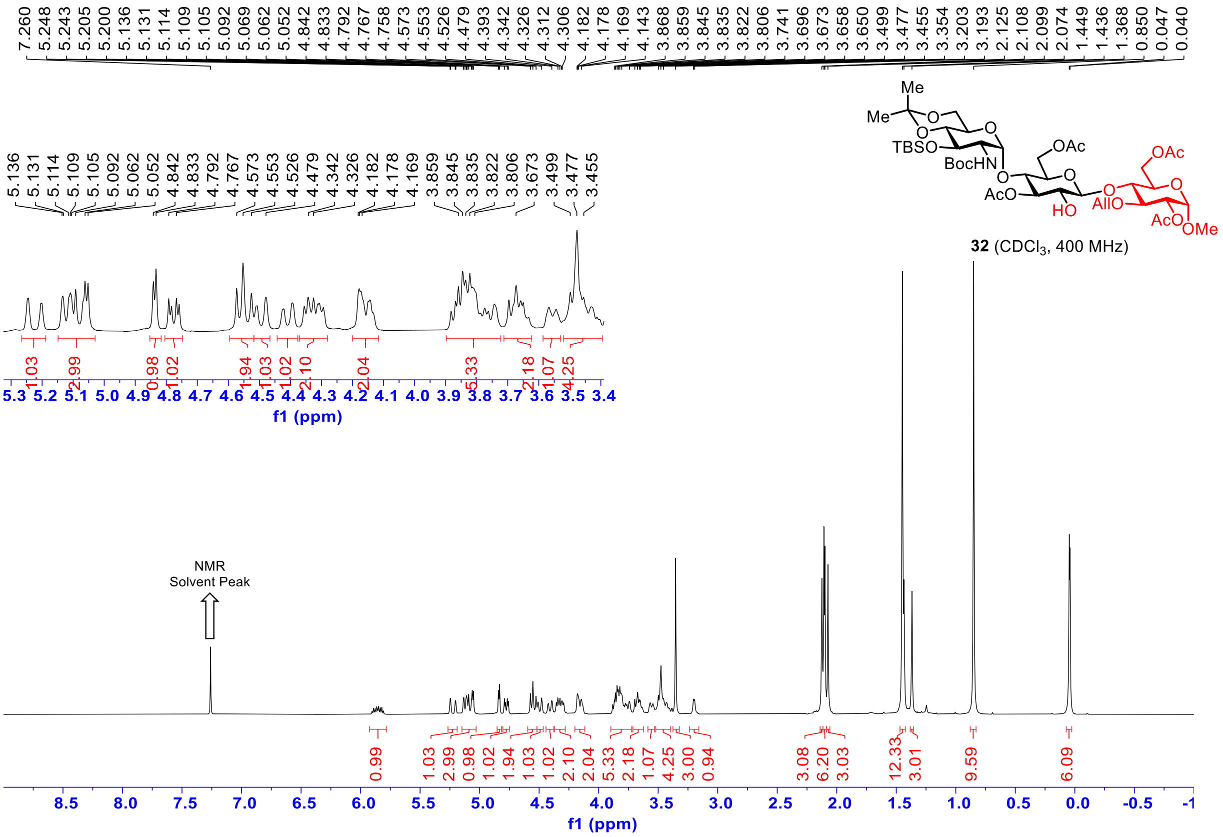
32 (CDCl$_3$, 400 MHz)
NMR Solvent Peak
f1 (ppm)

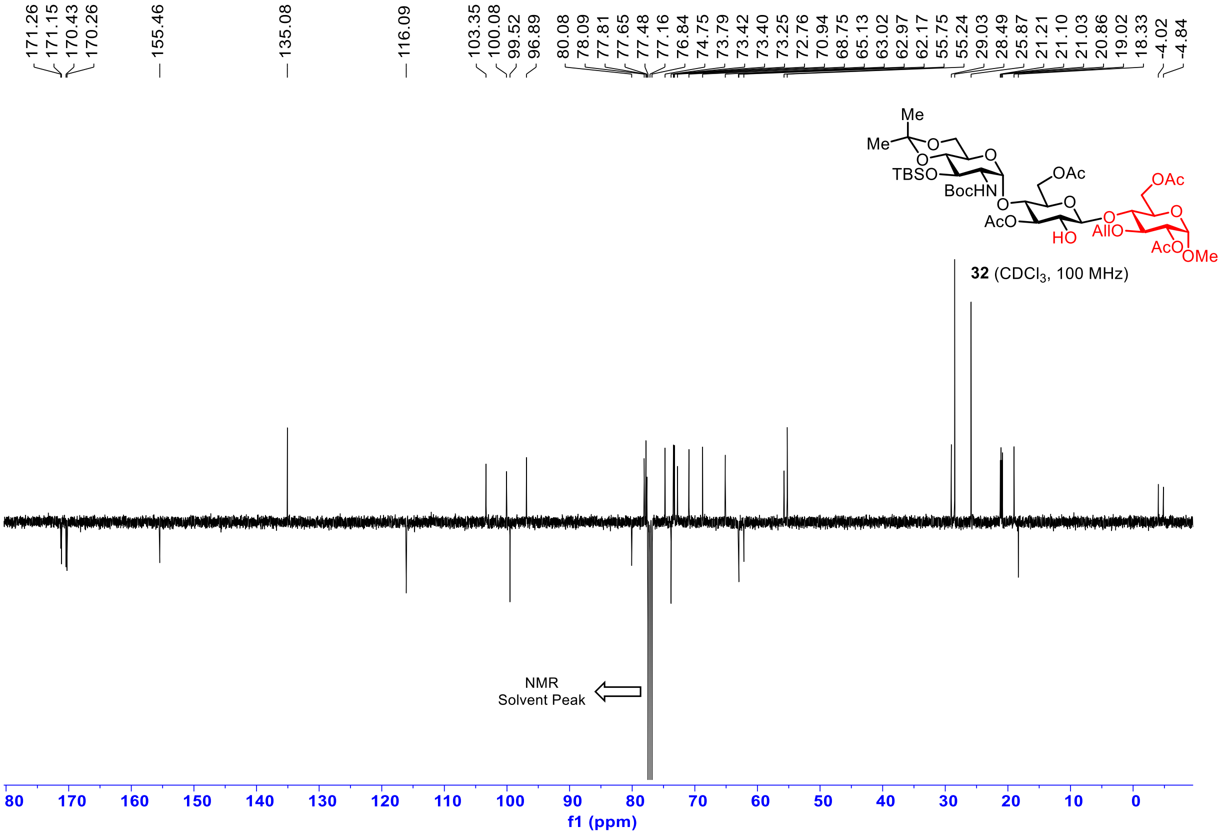
32 (CDCl$_3$, 100 MHz)
NMR Solvent Peak
f1 (ppm)

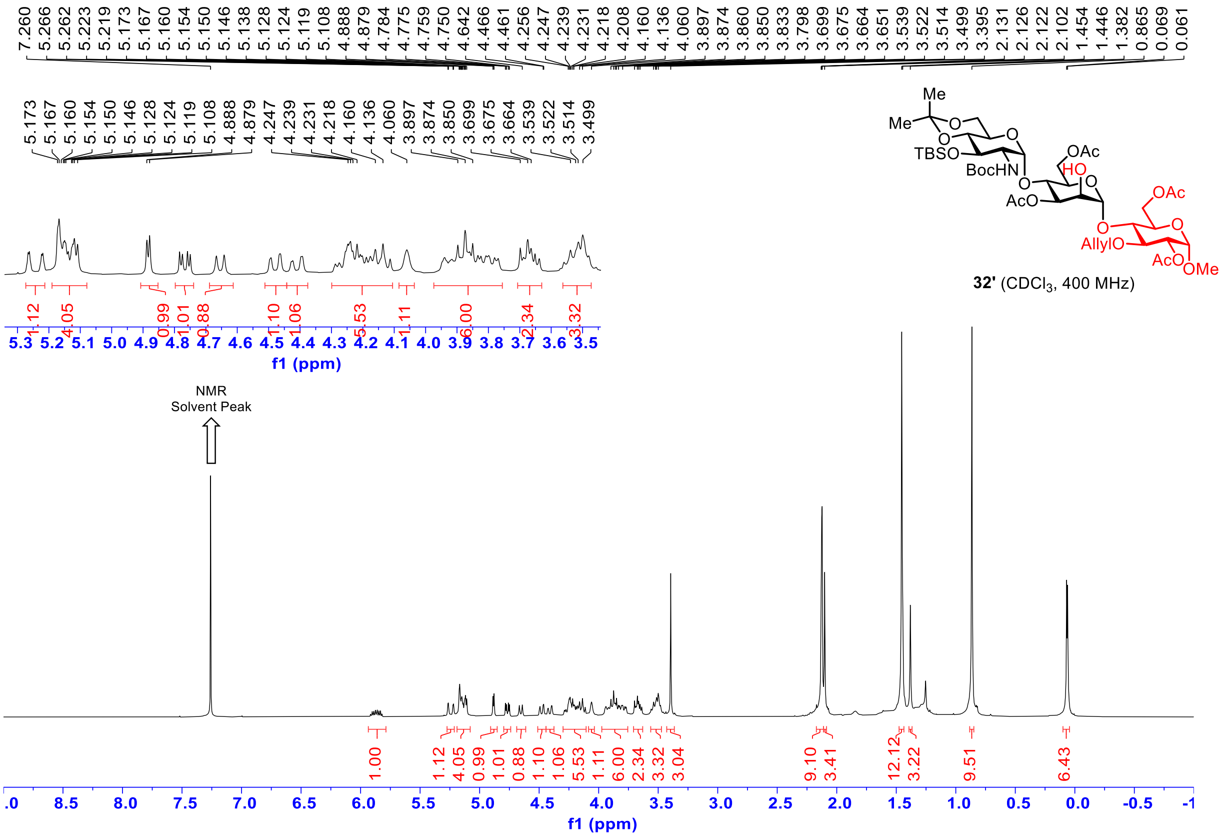
32' (CDCl3, 400 MHz)
NMR
Solvent Peak
f1 (ppm)

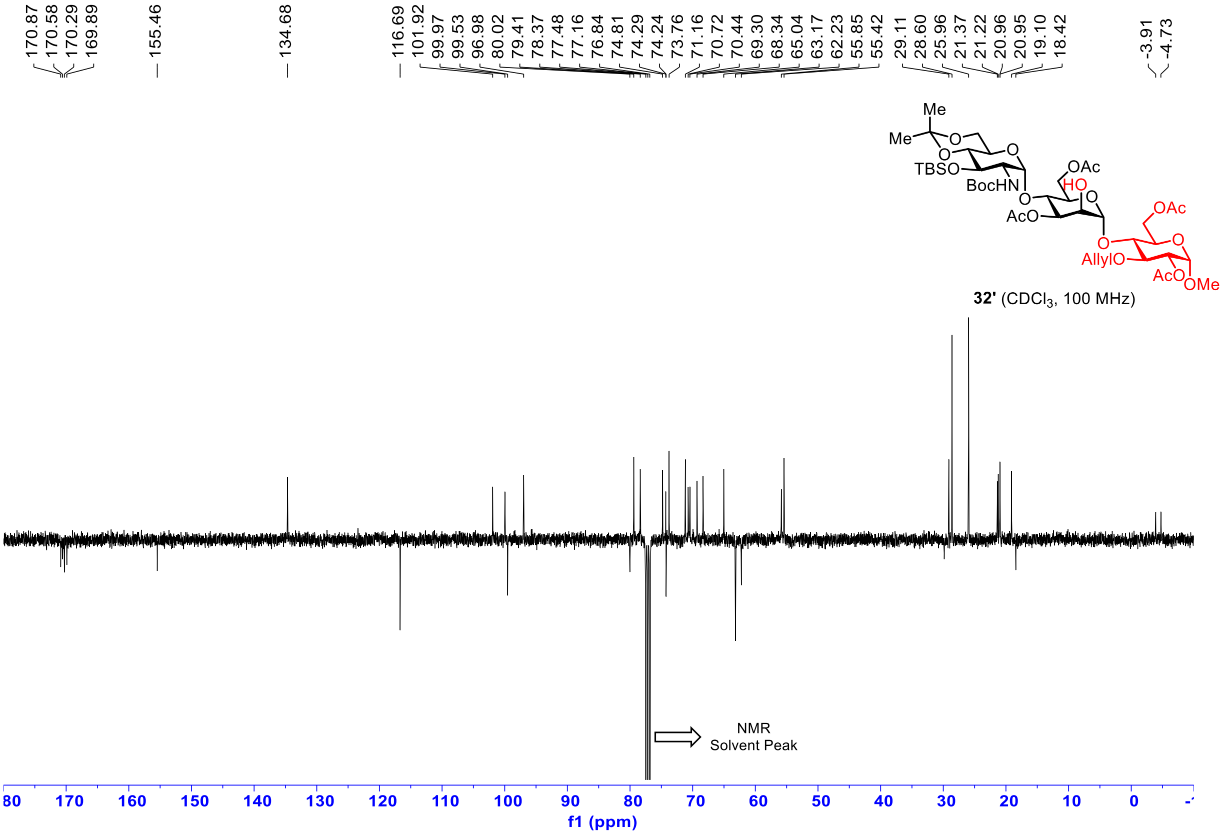
32' (CDCl3, 100 MHz)
NMR
Solvent Peak
f1 (ppm)

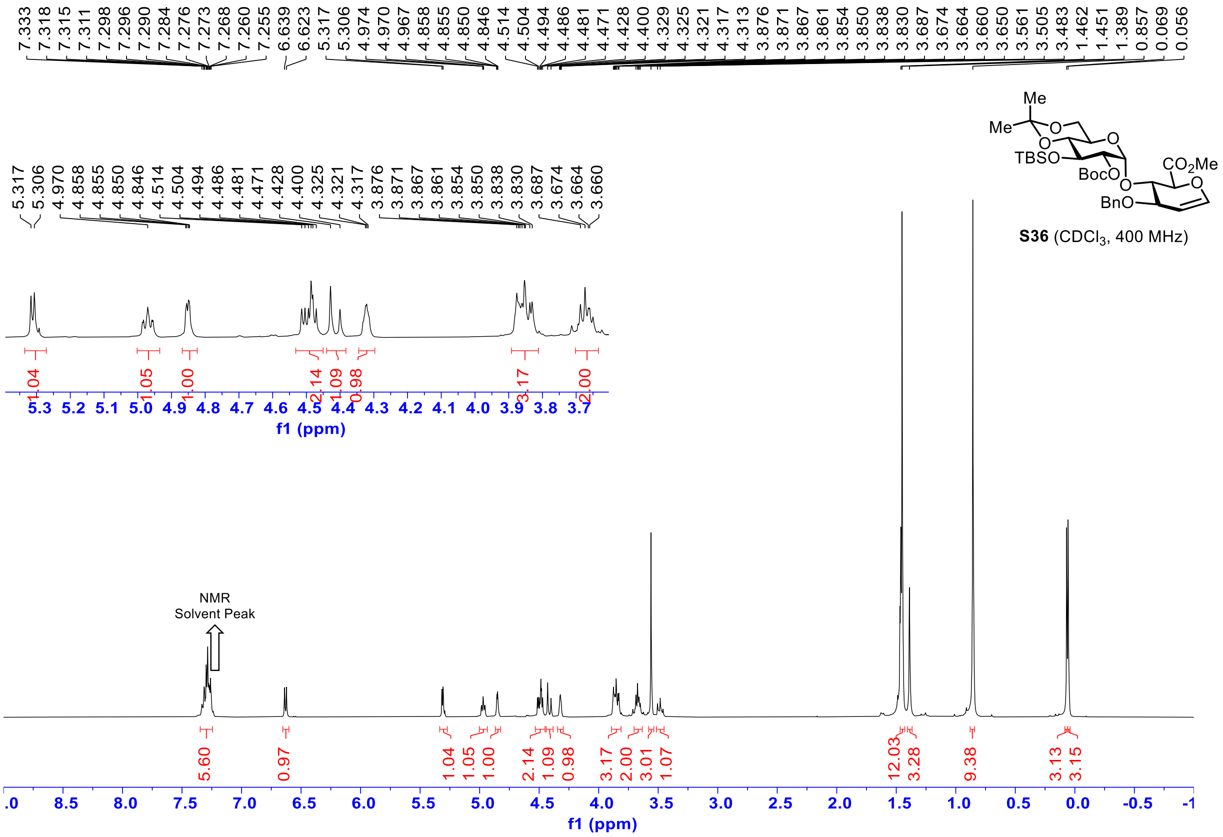
7.333 7.318 7.315 7.311 7.298 7.296 7.290 7.284 7.276 7.273 7.268 7.260 7.255 6.639 6.623 5.317 5.306 4.974 4.970 4.967 4.858 4.855 4.850 4.846 4.514 4.504 4.494 4.486 4.481 4.471 4.428 4.400 4.329 4.325 4.321 4.317 4.313 3.876 3.871 3.867 3.861 3.854 3.850 3.838 3.830 3.687 3.674 3.664 3.660 3.650 3.561 3.505 3.483 1.462 1.451 1.389 0.857 0.069 0.056
S36 ($CDCl_3$, 400 MHz)
NMR Solvent Peak
5.60 0.97 1.04 1.05 1.00 2.14 1.09 0.98 3.17 2.00 3.01 1.07 12.03 3.28 9.38 3.13 3.15
f1 (ppm)

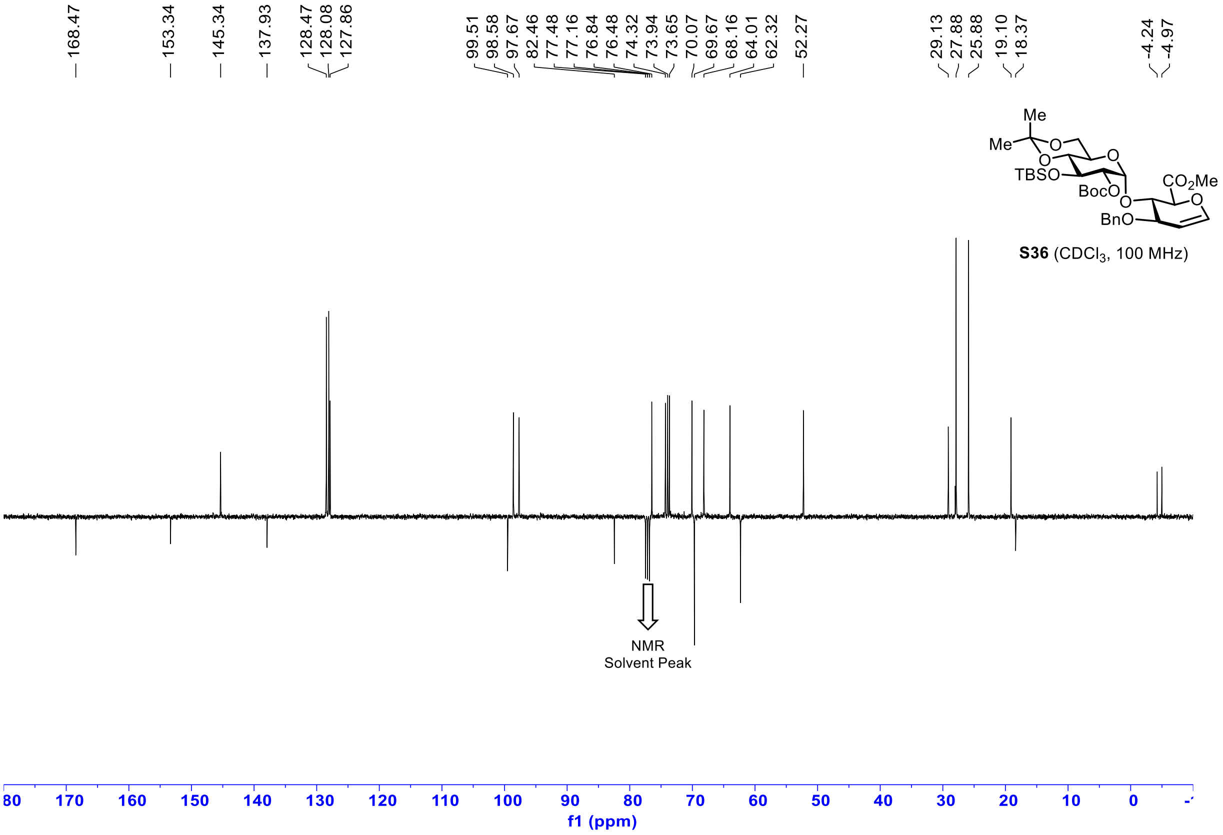
168.47 153.34 145.34 137.93 128.47 128.08 127.86 99.51 98.58 97.67 82.46 77.48 77.16 76.84 76.48 74.32 73.94 73.65 70.07 69.67 68.16 64.01 62.32 52.27 29.13 27.88 25.88 19.10 18.37 -4.24 -4.97
S36 ($CDCl_3$, 100 MHz)
NMR Solvent Peak
f1 (ppm)

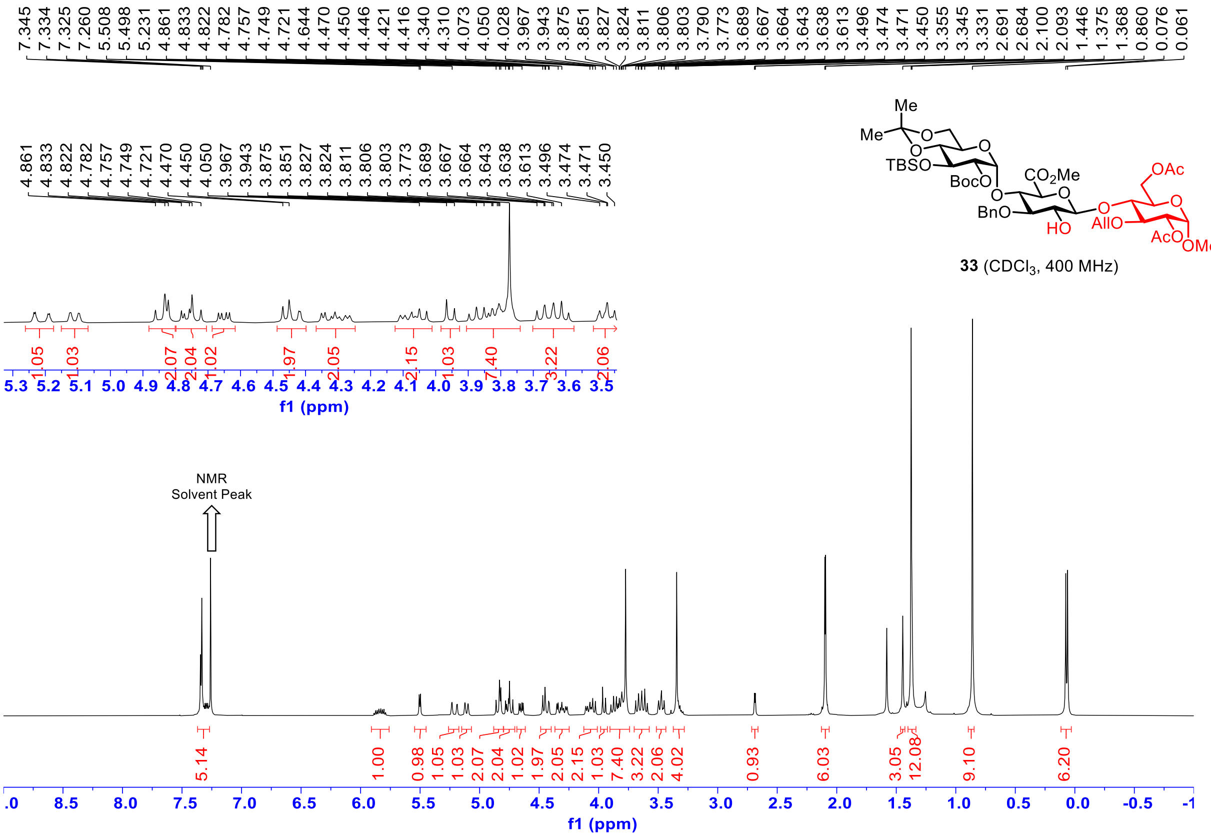
7.345 7.334 7.325 7.260 5.508 5.498 5.231 4.861 4.833 4.822 4.782 4.757 4.749 4.721 4.644 4.470 4.450 4.446 4.421 4.416 4.340 4.310 4.073 4.050 4.028 3.967 3.943 3.875 3.851 3.827 3.824 3.811 3.806 3.803 3.790 3.773 3.689 3.667 3.664 3.643 3.638 3.613 3.496 3.474 3.471 3.450 3.355 3.345 3.331 2.691 2.684 2.100 2.093 1.446 1.375 1.368 0.860 0.076 0.061
33 (CDCl$_3$, 400 MHz)
NMR Solvent Peak
f1 (ppm)

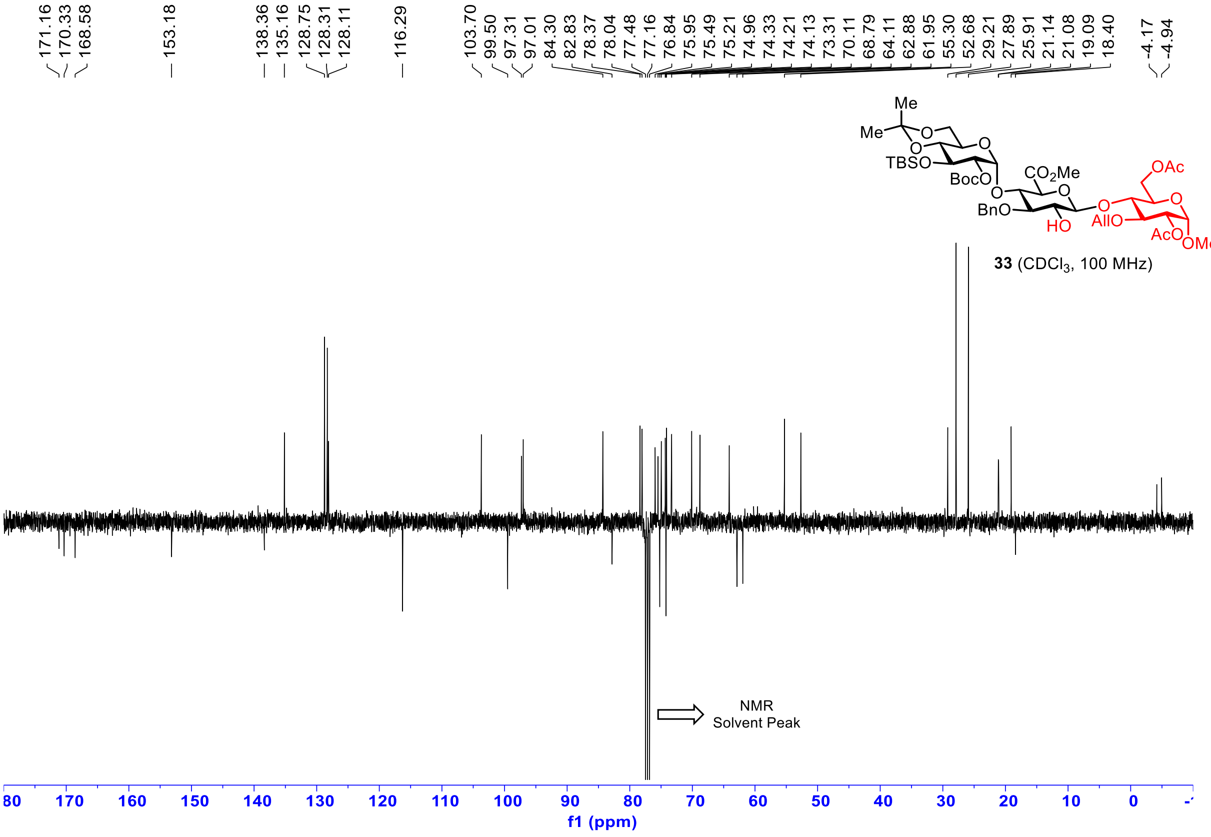
171.16 170.33 168.58 153.18 138.36 135.16 128.75 128.31 128.11 116.29 103.70 99.50 97.31 97.01 84.30 82.83 78.37 78.04 77.48 77.16 76.84 75.95 75.49 75.21 74.96 74.33 74.21 74.13 73.31 70.11 68.79 64.11 62.88 61.95 55.30 52.68 29.21 27.89 25.91 21.14 21.08 19.09 18.40 -4.17 -4.94
33 (CDCl$_3$, 100 MHz)
NMR Solvent Peak
f1 (ppm)

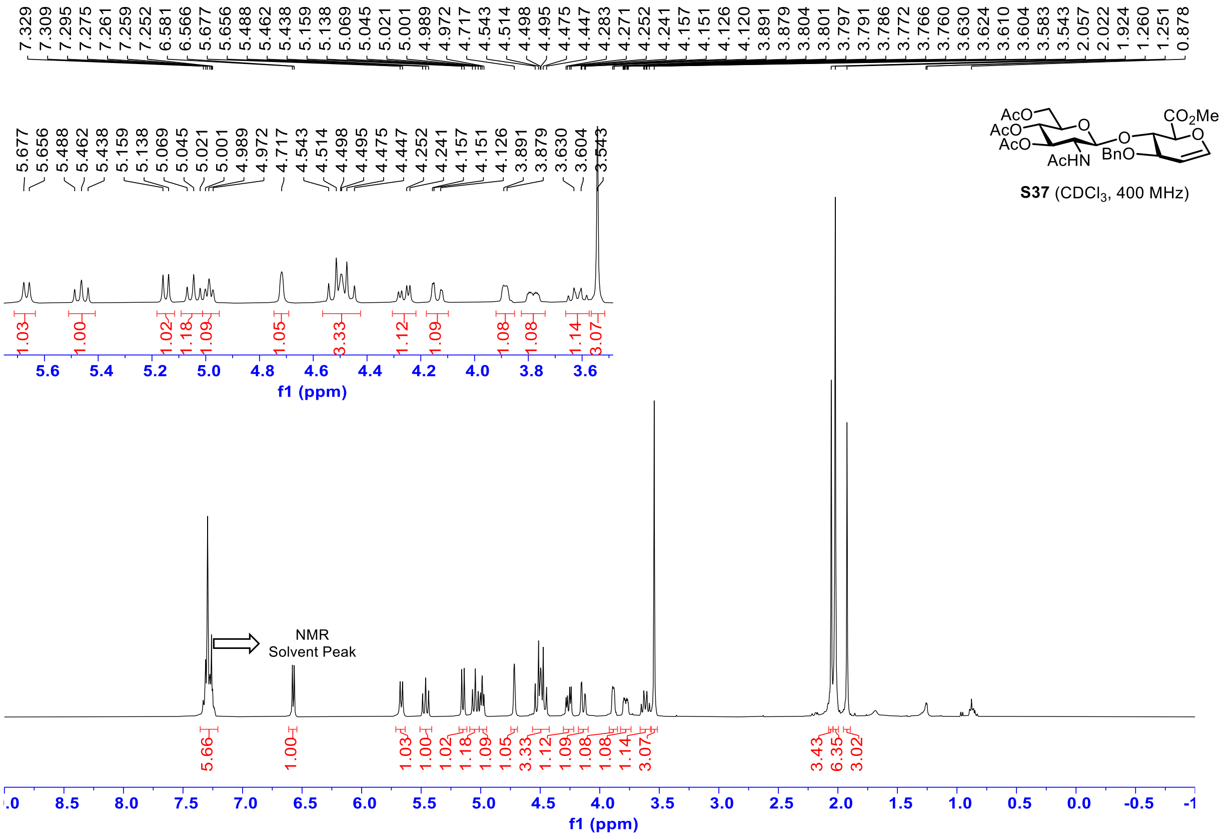
7.329 7.309 7.295 7.275 7.261 7.259 7.252 6.581 6.566 5.677 5.656 5.488 5.462 5.438 5.159 5.138 5.069 5.045 5.021 5.001 4.989 4.972 4.717 4.543 4.514 4.498 4.495 4.475 4.447 4.283 4.271 4.252 4.241 4.157 4.151 4.126 4.120 3.891 3.879 3.804 3.801 3.797 3.791 3.786 3.772 3.766 3.760 3.630 3.624 3.610 3.604 3.583 3.543 2.057 2.022 1.924 1.260 1.251 0.878
AcO
AcO
AcO
AcHN
O
CO2Me
O
BnO
O
S37 (CDCl3, 400 MHz)
NMR
Solvent Peak
f1 (ppm)

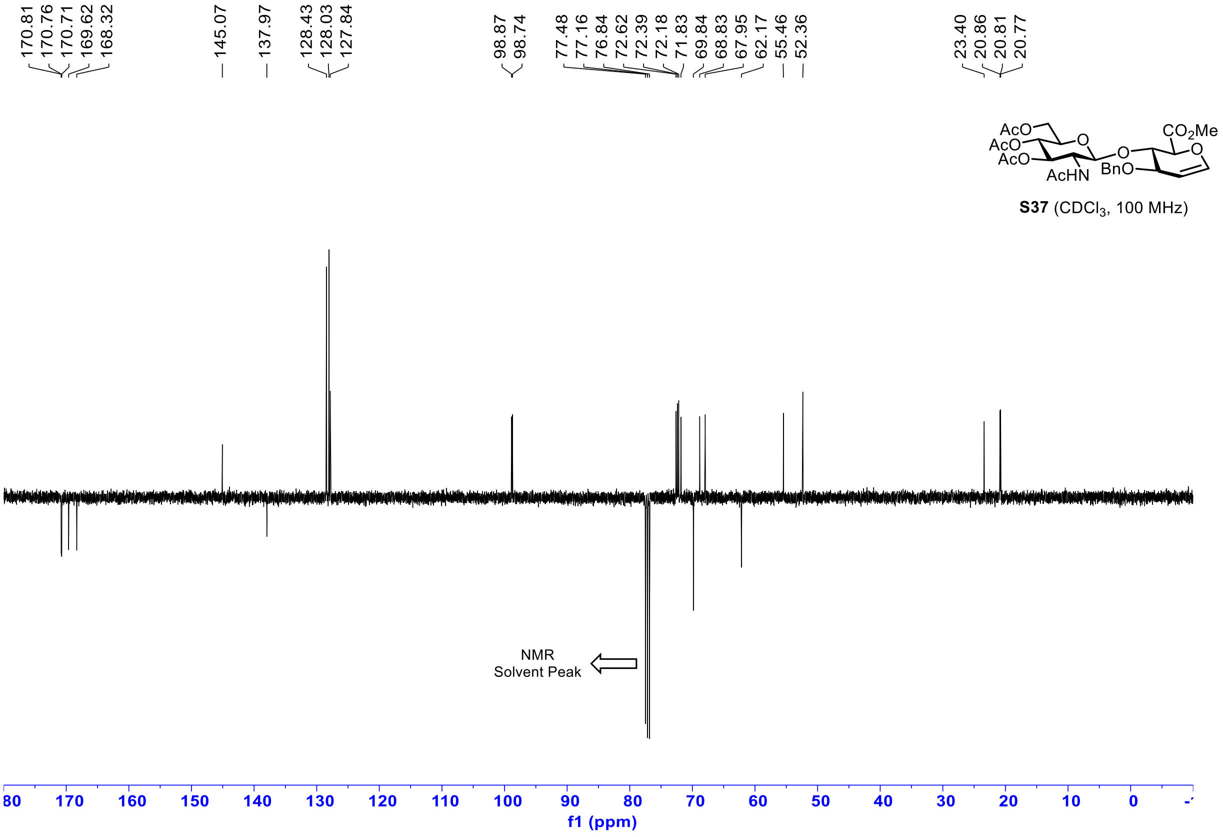
170.81 170.76 170.71 169.62 168.32 145.07 137.97 128.43 128.03 127.84 98.87 98.74 77.48 77.16 76.84 72.62 72.39 72.18 71.83 69.84 68.83 67.95 62.17 55.46 52.36 23.40 20.86 20.81 20.77
AcO
AcO
AcO
AcHN
O
CO2Me
O
BnO
O
S37 (CDCl3, 100 MHz)
NMR
Solvent Peak
f1 (ppm)

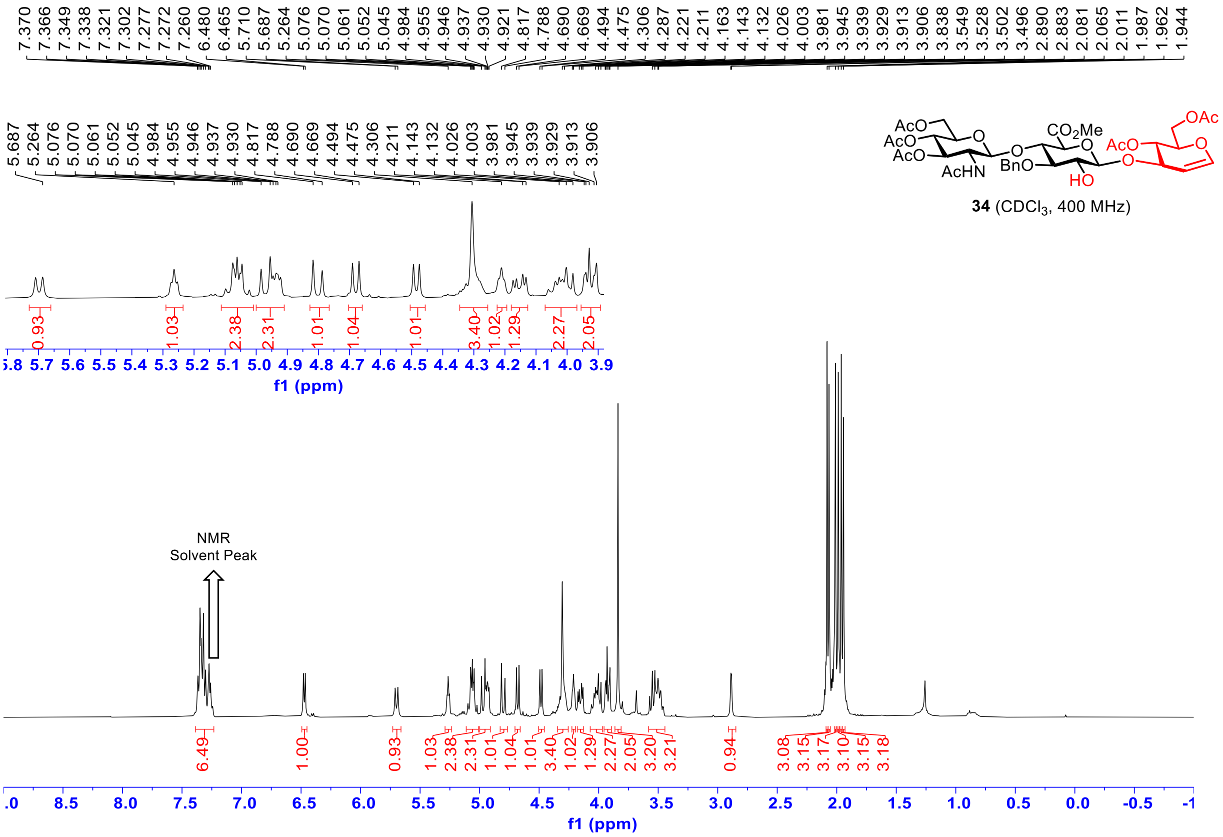
34 (CDCl3, 400 MHz)
NMR Solvent Peak
f1 (ppm)

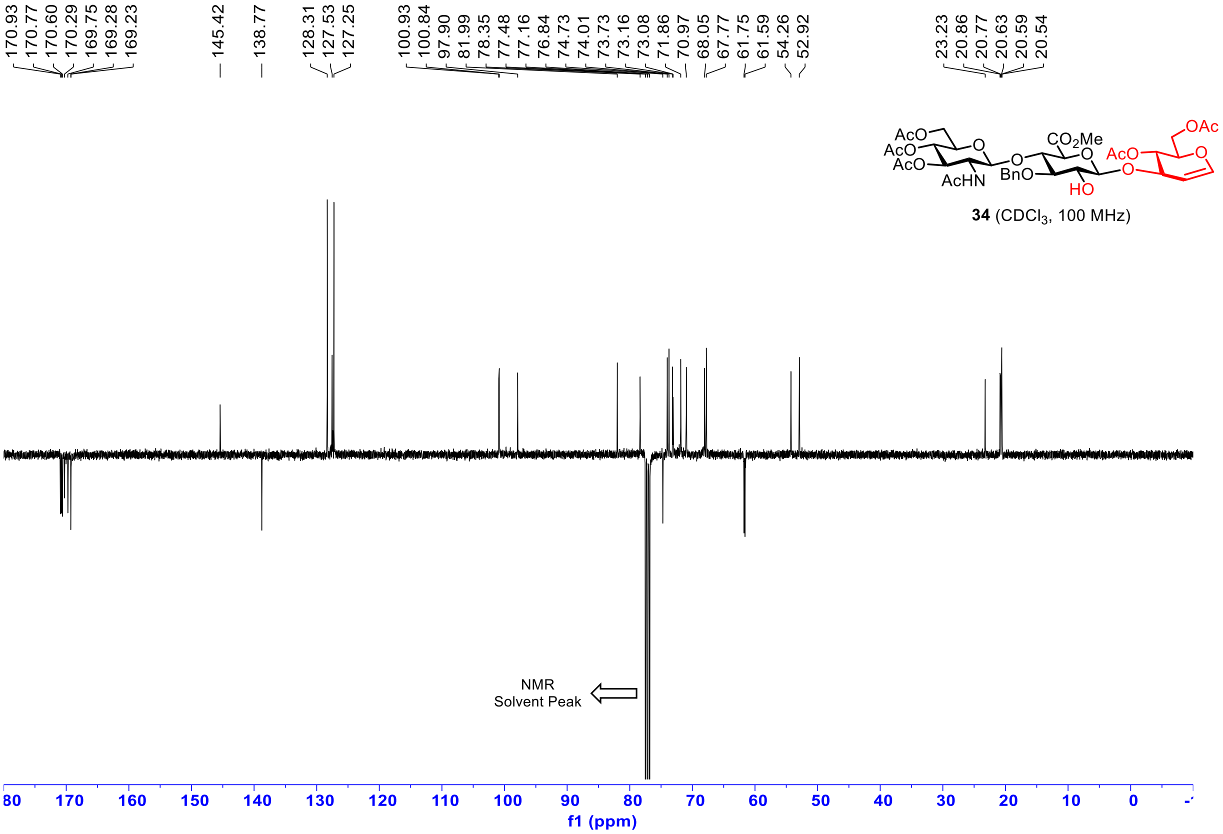
34 (CDCl3, 100 MHz)
NMR Solvent Peak
f1 (ppm)

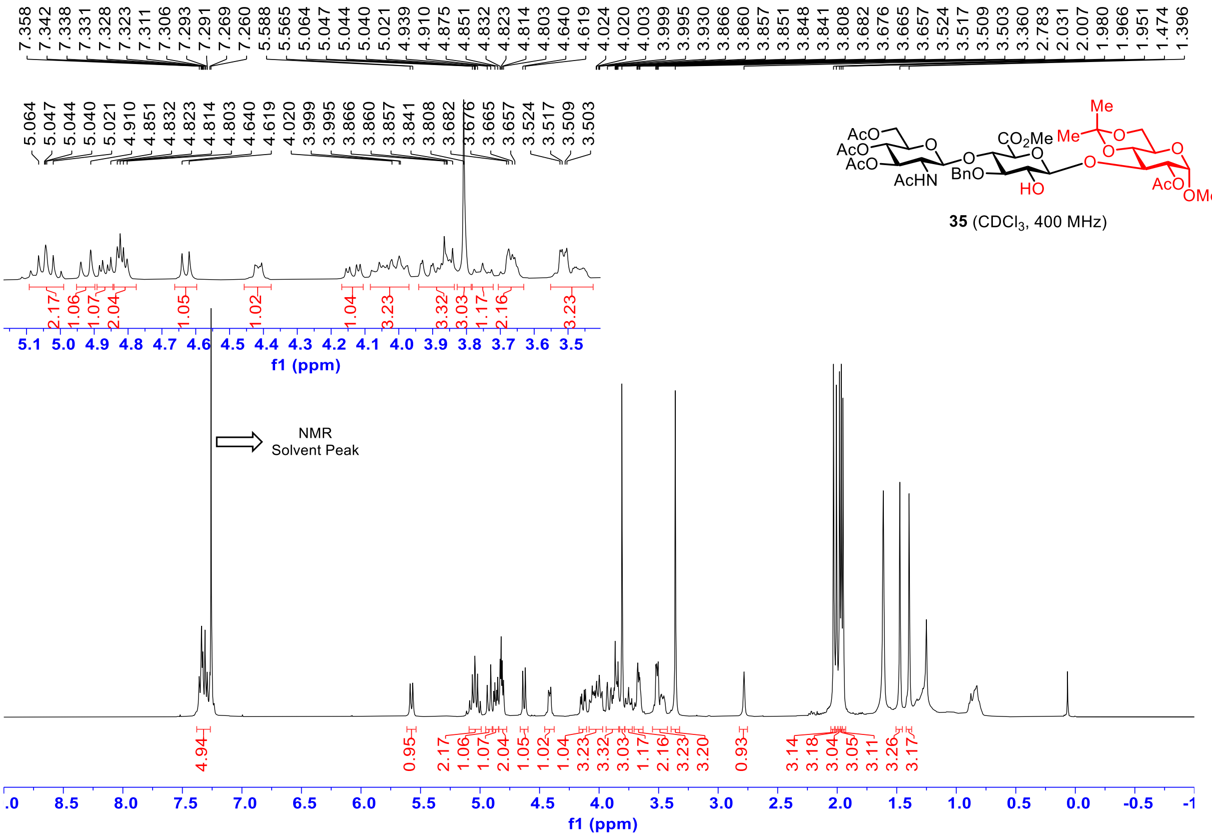

35 ($CDCl_3$, 400 MHz)

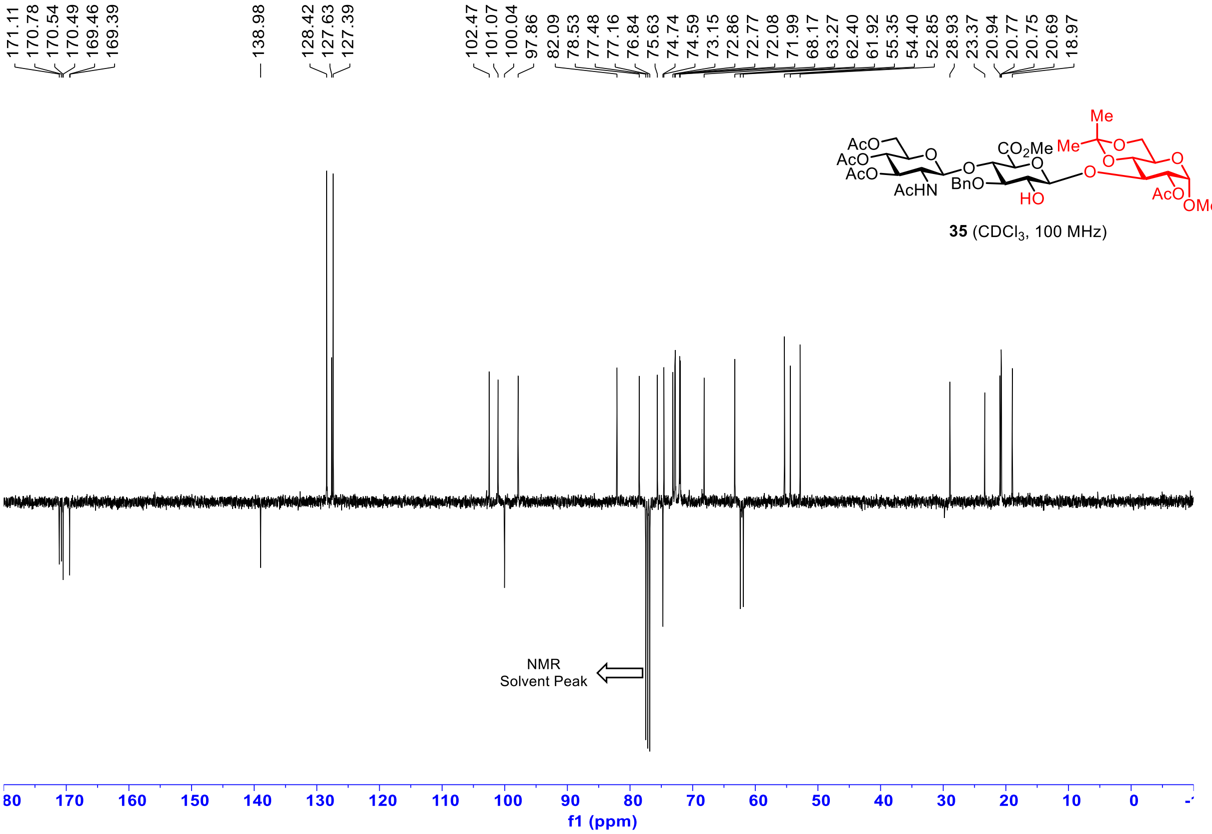

35 ($CDCl_3$, 100 MHz)

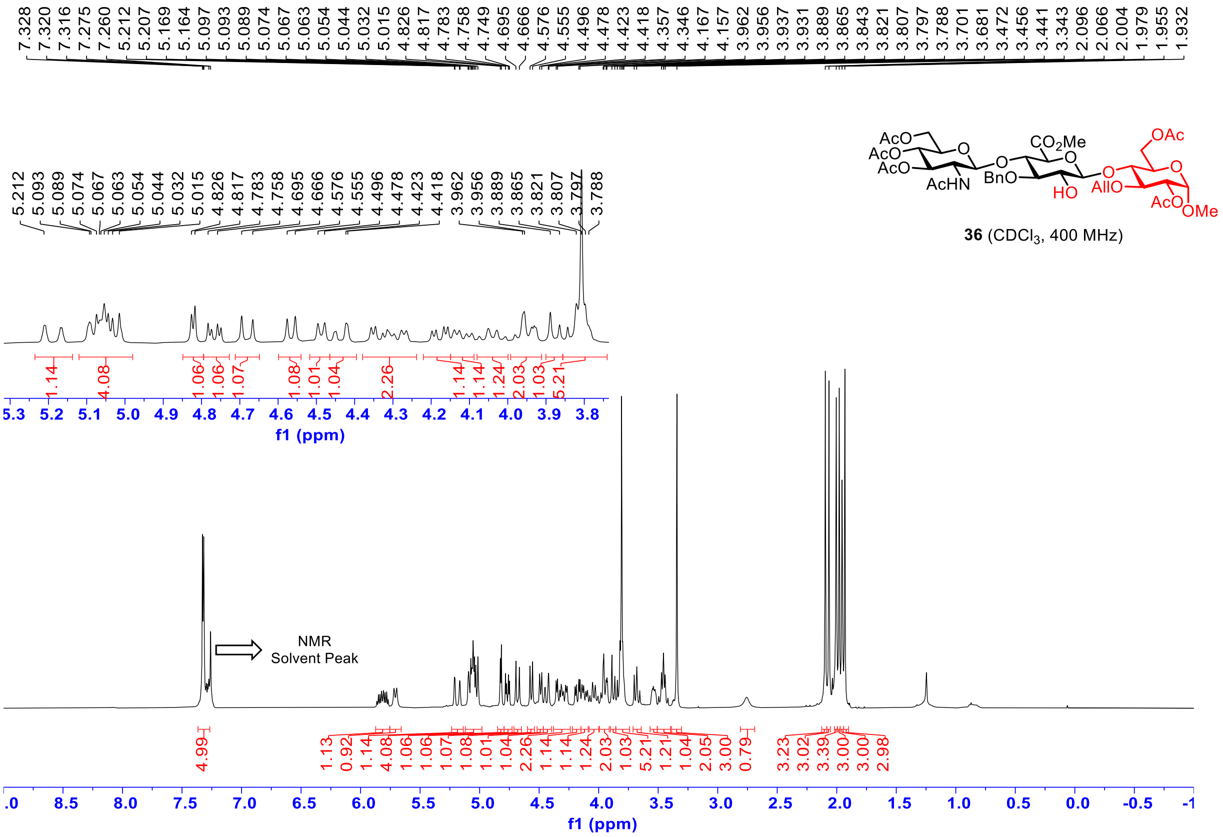

7.328
7.320
7.316
7.275
7.260
5.212
5.207
5.169
5.164
5.097
5.093
5.089
5.074
5.067
5.063
5.054
5.044
5.032
5.015
4.826
4.817
4.783
4.758
4.749
4.695
4.666
4.576
4.555
4.496
4.478
4.423
4.418
4.357
4.346
4.167
4.157
3.962
3.956
3.937
3.931
3.889
3.865
3.843
3.821
3.807
3.797
3.788
3.701
3.681
3.472
3.456
3.441
3.343
2.096
2.066
2.004
1.979
1.955
1.932
36 (CDCl3, 400 MHz)
NMR Solvent Peak
f1 (ppm)

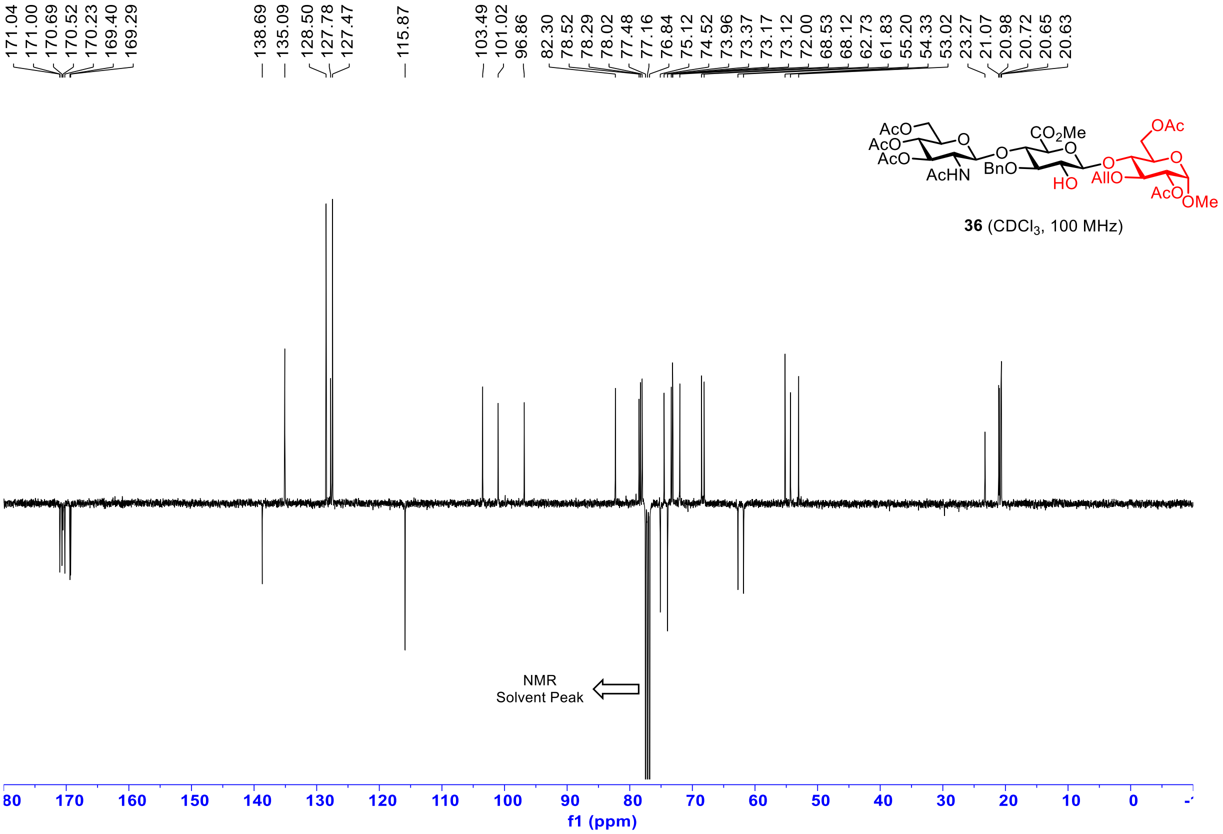

171.04
171.00
170.69
170.52
170.23
169.40
169.29
138.69
135.09
128.50
127.78
127.47
115.87
103.49
101.02
96.86
82.30
78.52
78.29
78.02
77.48
77.16
76.84
75.12
74.52
73.96
73.37
73.17
73.12
72.00
68.53
68.12
62.73
61.83
55.20
54.33
53.02
23.27
21.07
20.98
20.72
20.65
20.63
36 (CDCl3, 100 MHz)
NMR Solvent Peak
f1 (ppm)

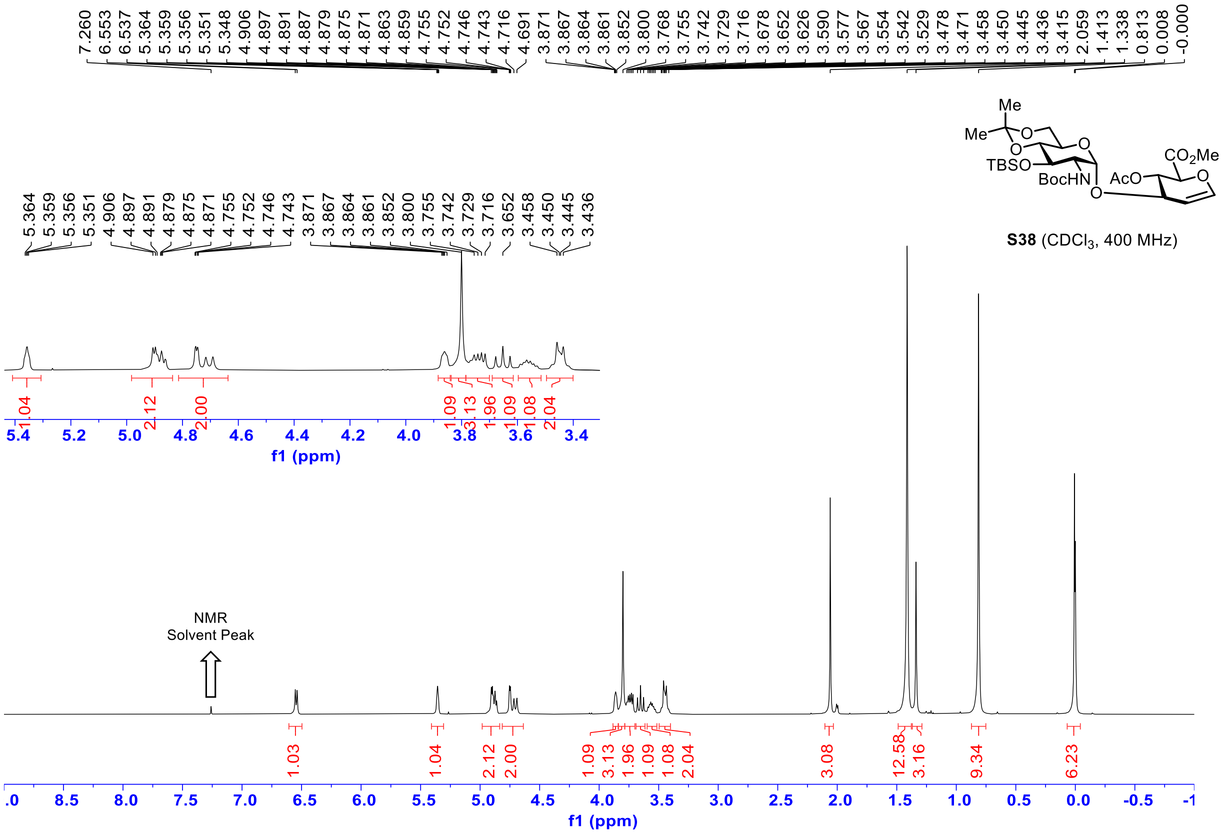
7.260 6.553 6.537 5.364 5.359 5.356 5.351 5.348 4.906 4.897 4.891 4.887 4.879 4.875 4.871 4.863 4.859 4.755 4.752 4.746 4.743 4.716 4.691 3.871 3.867 3.864 3.861 3.852 3.800 3.768 3.755 3.742 3.729 3.716 3.678 3.652 3.626 3.590 3.577 3.567 3.554 3.542 3.529 3.478 3.471 3.458 3.450 3.445 3.436 3.415 2.059 1.413 1.338 0.813 0.008 -0.000
Me
Me
O
O
TBSO
BocHN
O
CO2Me
AcO
O
O
S38 (CDCl3, 400 MHz)
NMR
Solvent Peak
1.03 1.04 2.12 2.00 1.09 3.13 1.96 1.09 1.08 2.04 3.08 12.58 3.16 9.34 6.23
f1 (ppm)

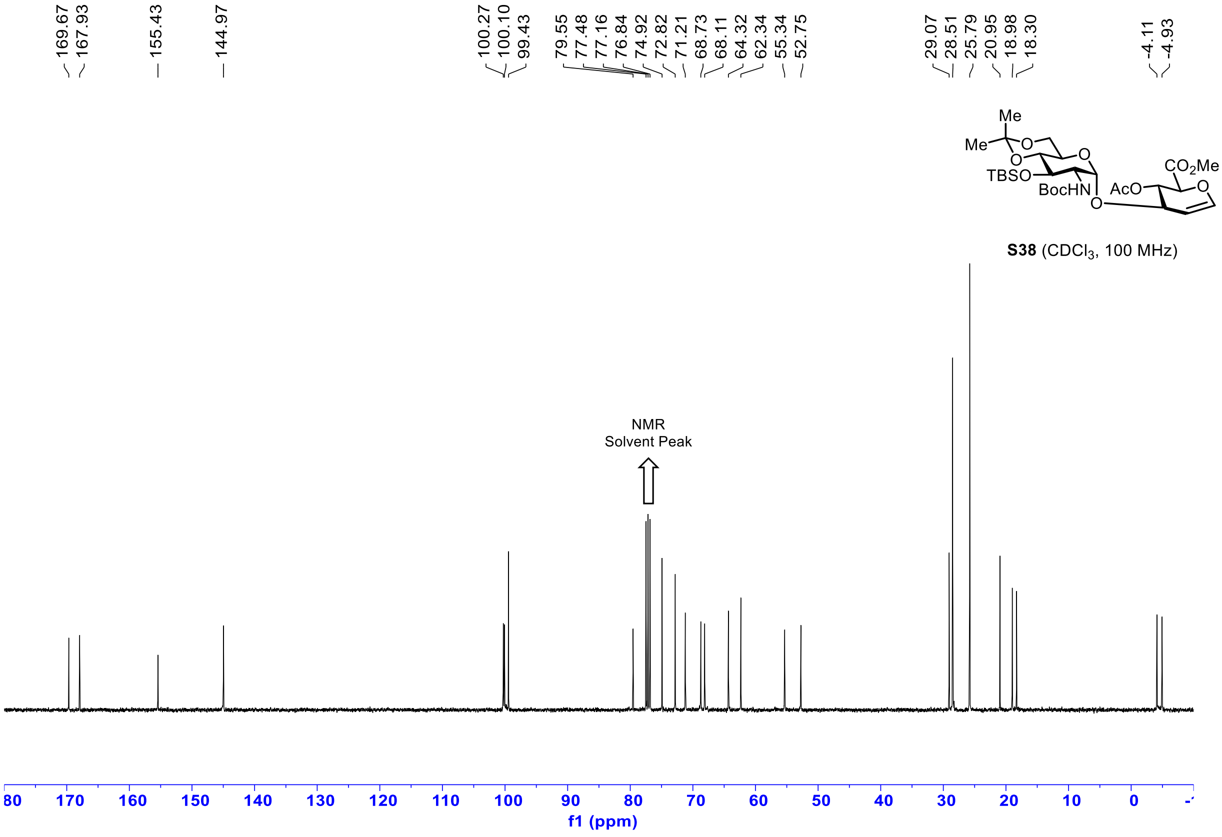
169.67 167.93 155.43 144.97 100.27 100.10 99.43 79.55 77.48 77.16 76.84 74.92 72.82 71.21 68.73 68.11 64.32 62.34 55.34 52.75 29.07 28.51 25.79 20.95 18.98 18.30 -4.11 -4.93
Me
Me
O
O
TBSO
BocHN
O
CO2Me
AcO
O
O
S38 (CDCl3, 100 MHz)
NMR
Solvent Peak
f1 (ppm)

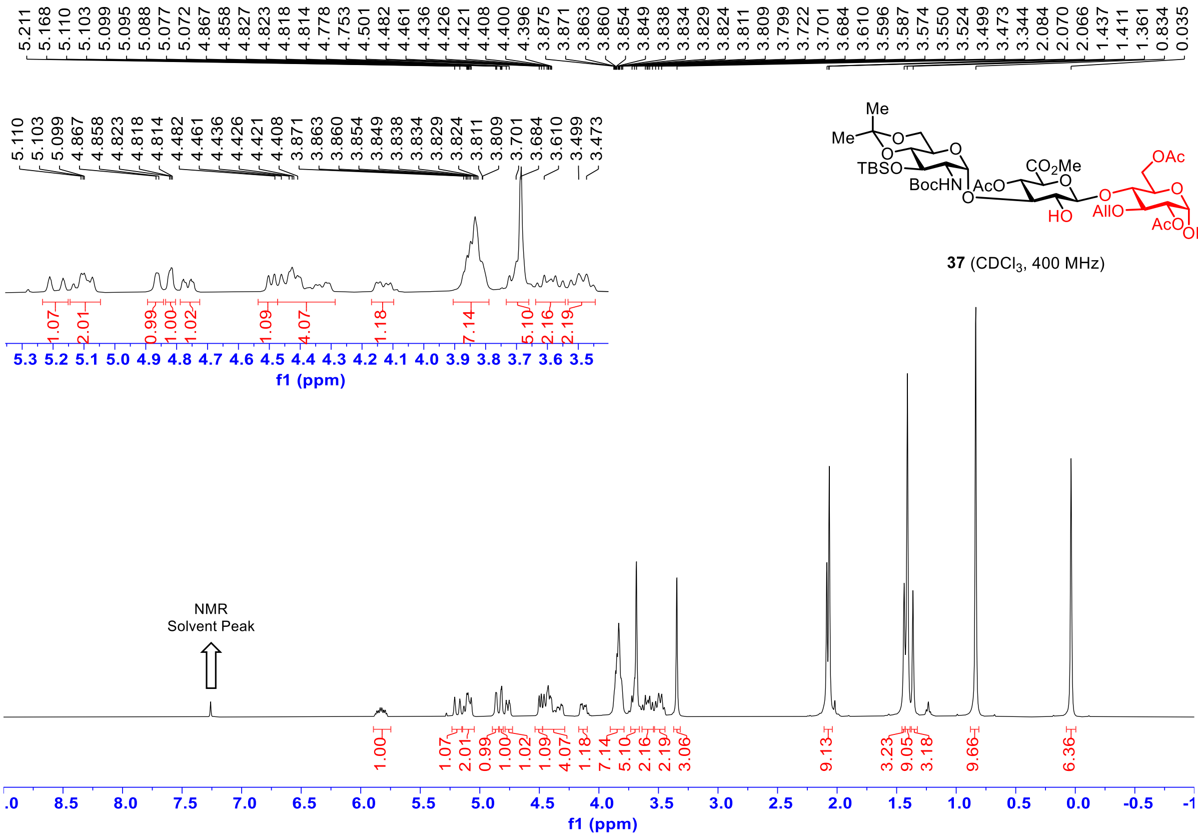

37 ($CDCl_3$, 400 MHz)

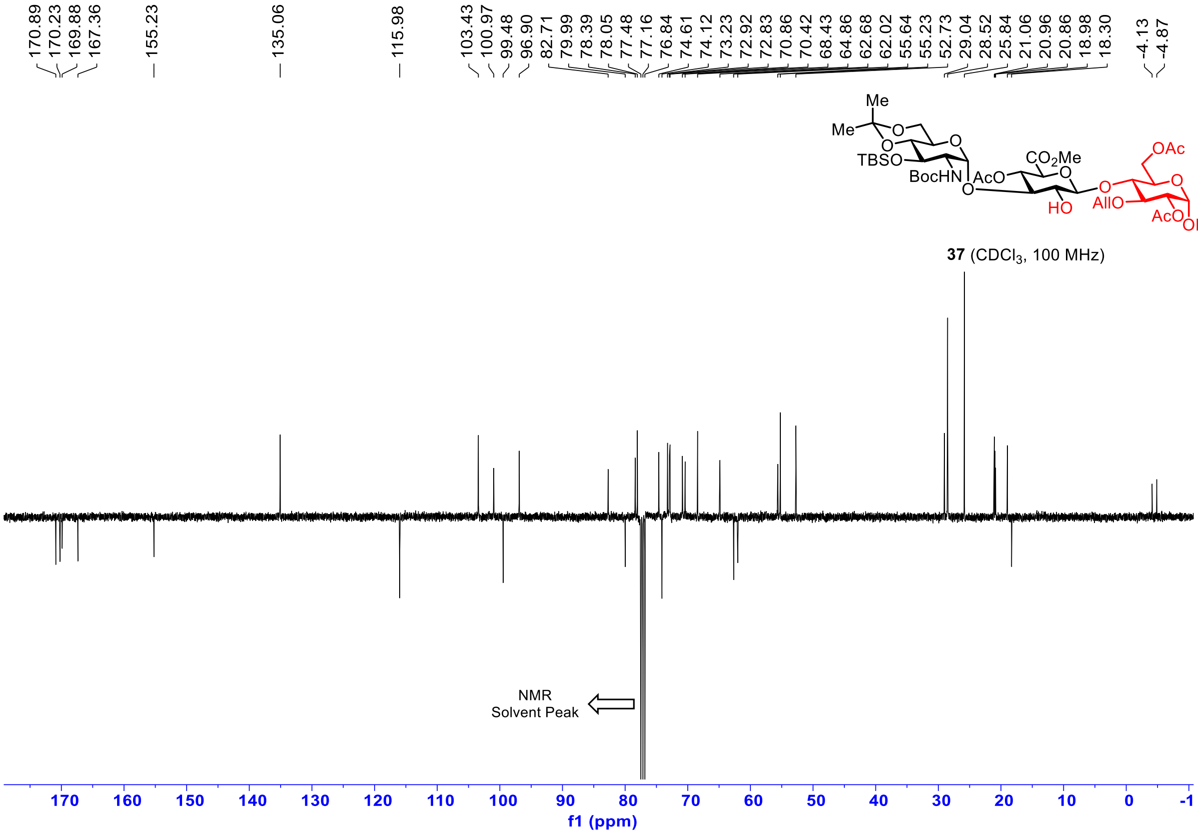

37 ($CDCl_3$, 100 MHz)

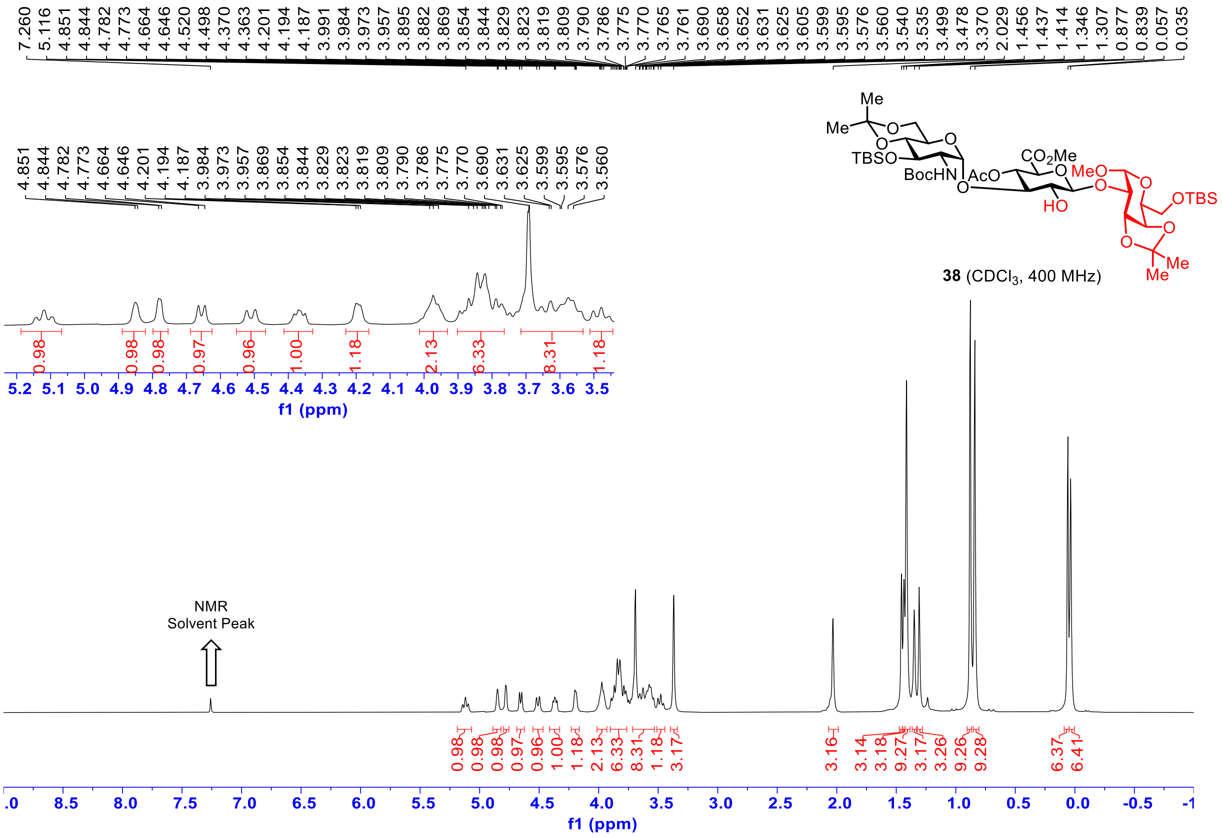
7.260 5.116 4.851 4.844 4.782 4.773 4.664 4.646 4.520 4.498 4.370 4.363 4.201 4.194 4.187 3.991 3.984 3.973 3.957 3.895 3.882 3.869 3.854 3.844 3.829 3.823 3.819 3.809 3.790 3.786 3.775 3.770 3.765 3.761 3.690 3.658 3.652 3.631 3.625 3.605 3.599 3.595 3.576 3.560 3.540 3.535 3.499 3.478 3.370 2.029 1.456 1.437 1.414 1.346 1.307 0.877 0.839 0.057 0.035
4.851 4.844 4.782 4.773 4.664 4.646 4.201 4.194 4.187 3.984 3.973 3.957 3.869 3.854 3.844 3.829 3.823 3.819 3.809 3.790 3.786 3.775 3.770 3.690 3.631 3.625 3.599 3.595 3.576 3.560
0.98 0.98 0.98 0.97 0.96 1.00 1.18 2.13 6.33 8.31 1.18
f1 (ppm)
Me
Me
O
O
O
TBSO
BocHN
AcO
CO2Me
O
MeO
O
O
OTBS
HO
O
O
Me
Me
38 (CDCl3, 400 MHz)
NMR
Solvent Peak
0.98 0.98 0.98 0.97 0.96 1.00 1.18 2.13 6.33 8.31 1.18 3.17
3.16 3.14 3.18 9.27 3.17 3.26 9.26 9.28 6.37 6.41
f1 (ppm)

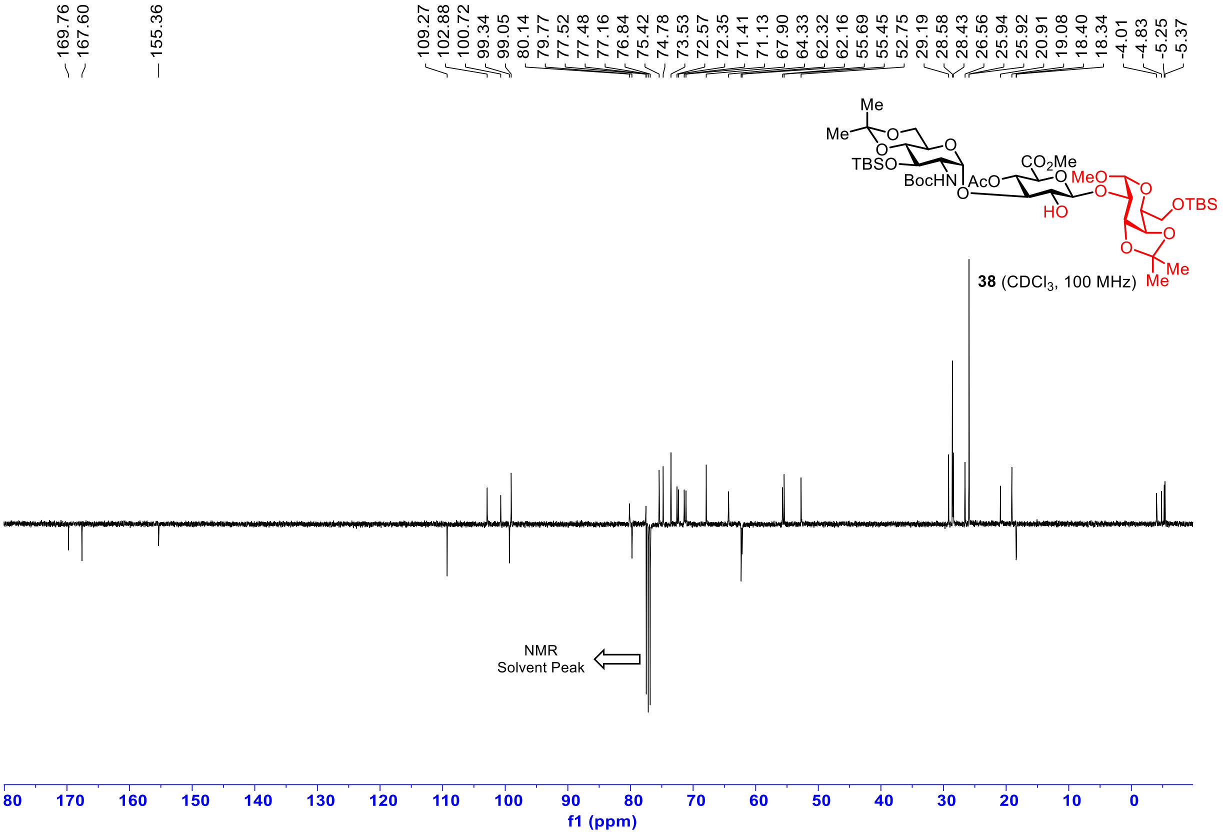
169.76 167.60 155.36 109.27 102.88 100.72 99.34 99.05 80.14 79.77 77.52 77.48 77.16 76.84 75.42 74.78 73.53 72.57 72.35 71.41 71.13 67.90 64.33 62.32 62.16 55.69 55.45 52.75 29.19 28.58 28.43 26.56 25.94 25.92 20.91 19.08 18.40 18.34 -4.01 -4.83 -5.25 -5.37
Me
Me
O
O
O
TBSO
BocHN
AcO
CO2Me
O
MeO
O
O
OTBS
HO
O
O
Me
Me
38 (CDCl3, 100 MHz)
NMR
Solvent Peak
f1 (ppm)

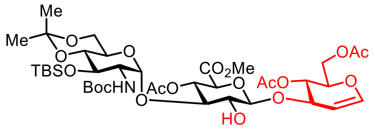

39 ($CDCl_3$, 400 MHz)

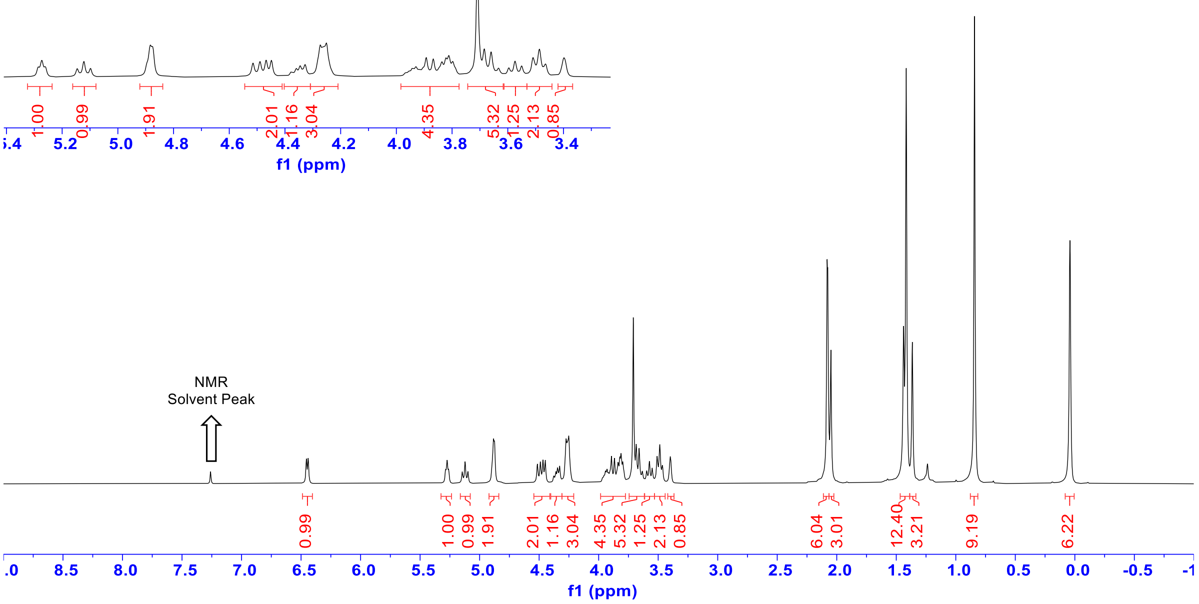

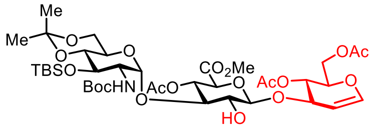

39 ($CDCl_3$, 100 MHz)

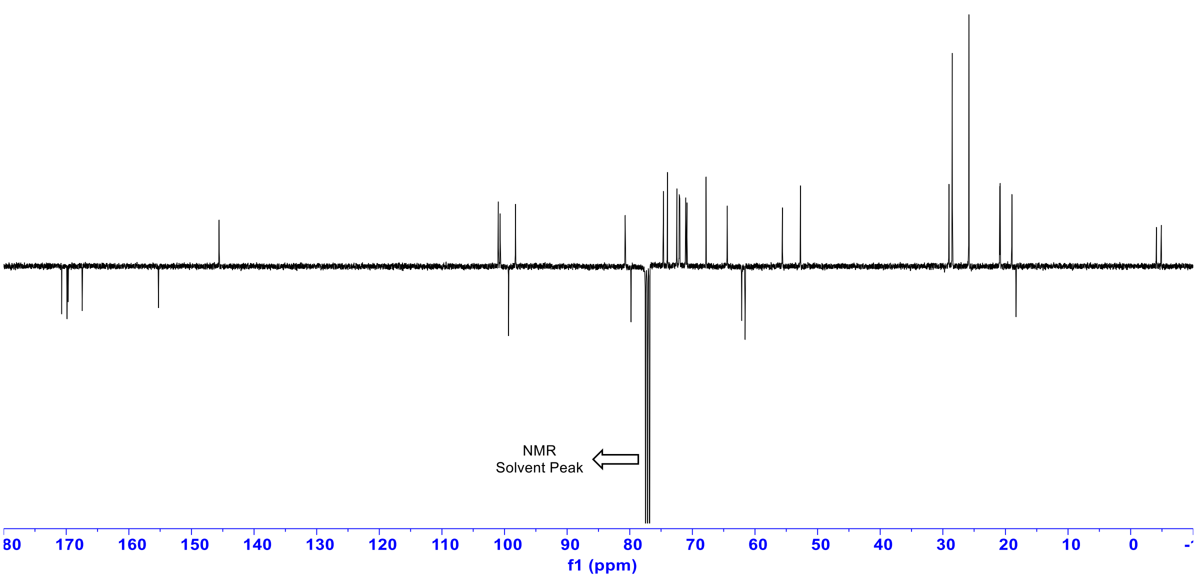

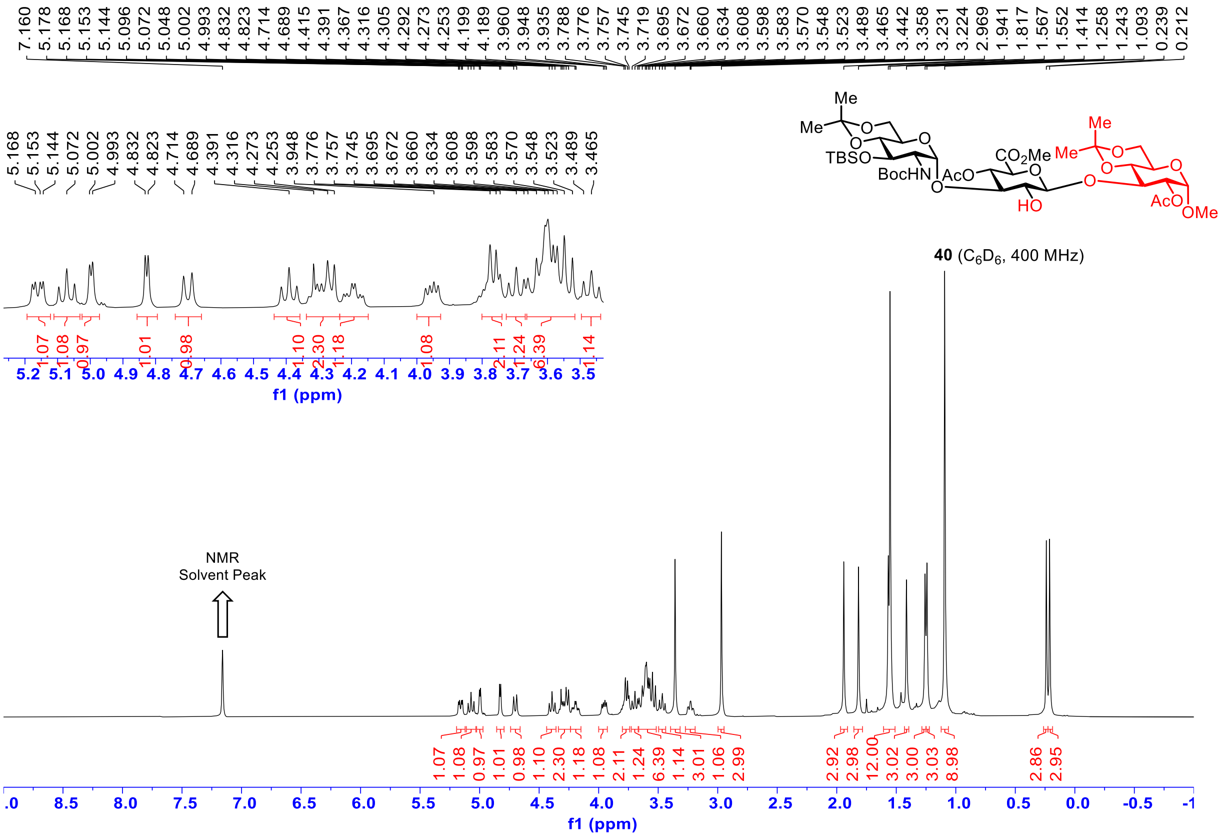
40 (C_6D_6, 400 MHz)
NMR Solvent Peak
f1 (ppm)

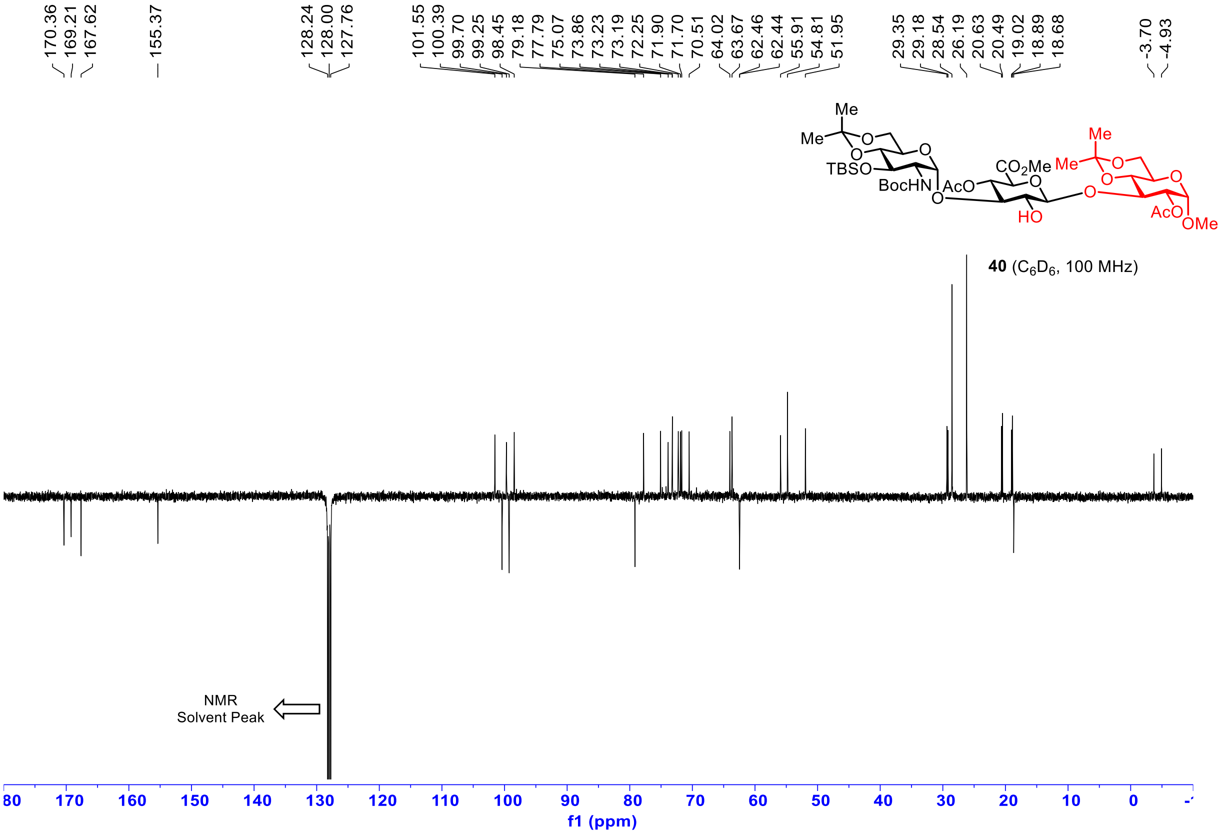
40 (C_6D_6, 100 MHz)
NMR Solvent Peak
f1 (ppm)

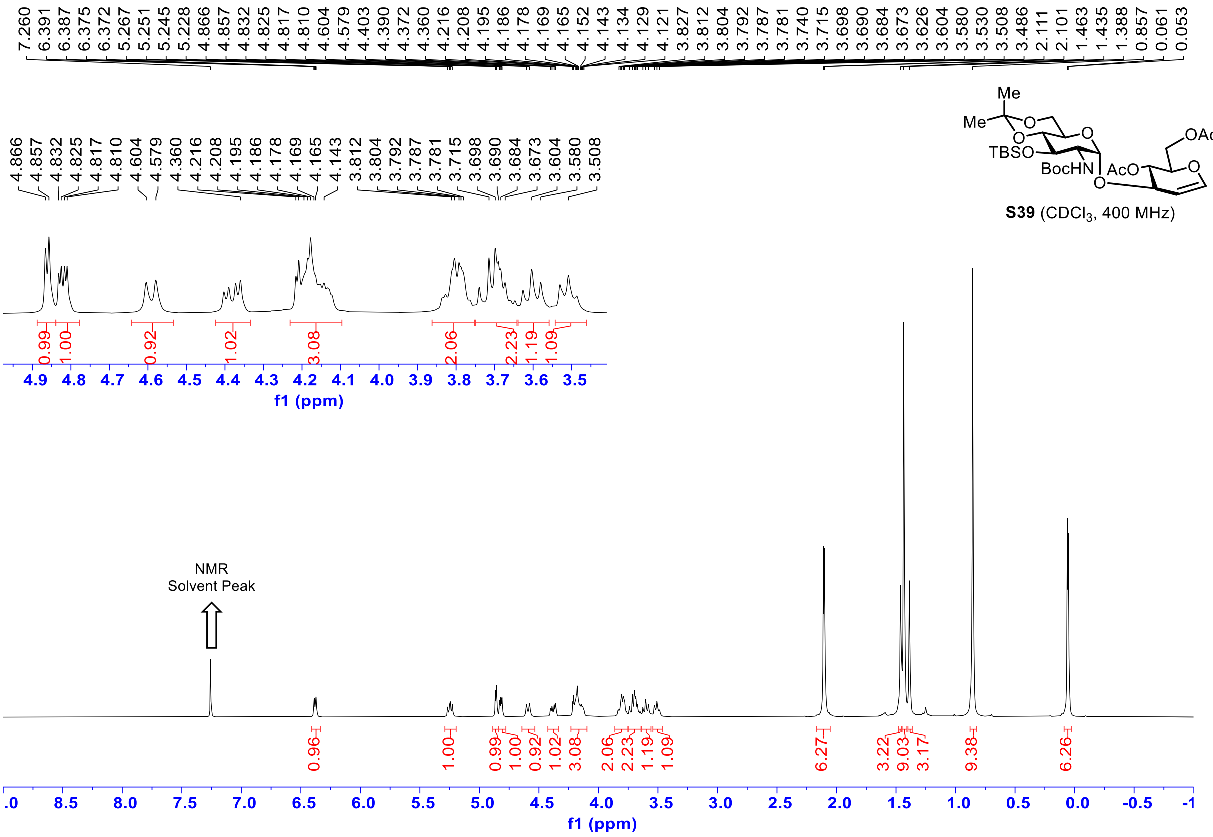
7.260 6.391 6.387 6.375 6.372 5.267 5.251 5.245 5.228 4.866 4.857 4.832 4.825 4.817 4.810 4.604 4.579 4.403 4.390 4.372 4.360 4.216 4.208 4.195 4.186 4.178 4.169 4.165 4.152 4.143 4.134 4.129 4.121 3.827 3.812 3.804 3.792 3.787 3.781 3.740 3.715 3.698 3.690 3.684 3.673 3.626 3.604 3.580 3.530 3.508 3.486 2.111 2.101 1.463 1.435 1.388 0.857 0.061 0.053
Me
Me
O
O
O
TBSO
BocHN
AcO
O
OAc
O
S39 (CDCl3, 400 MHz)
NMR Solvent Peak
f1 (ppm)

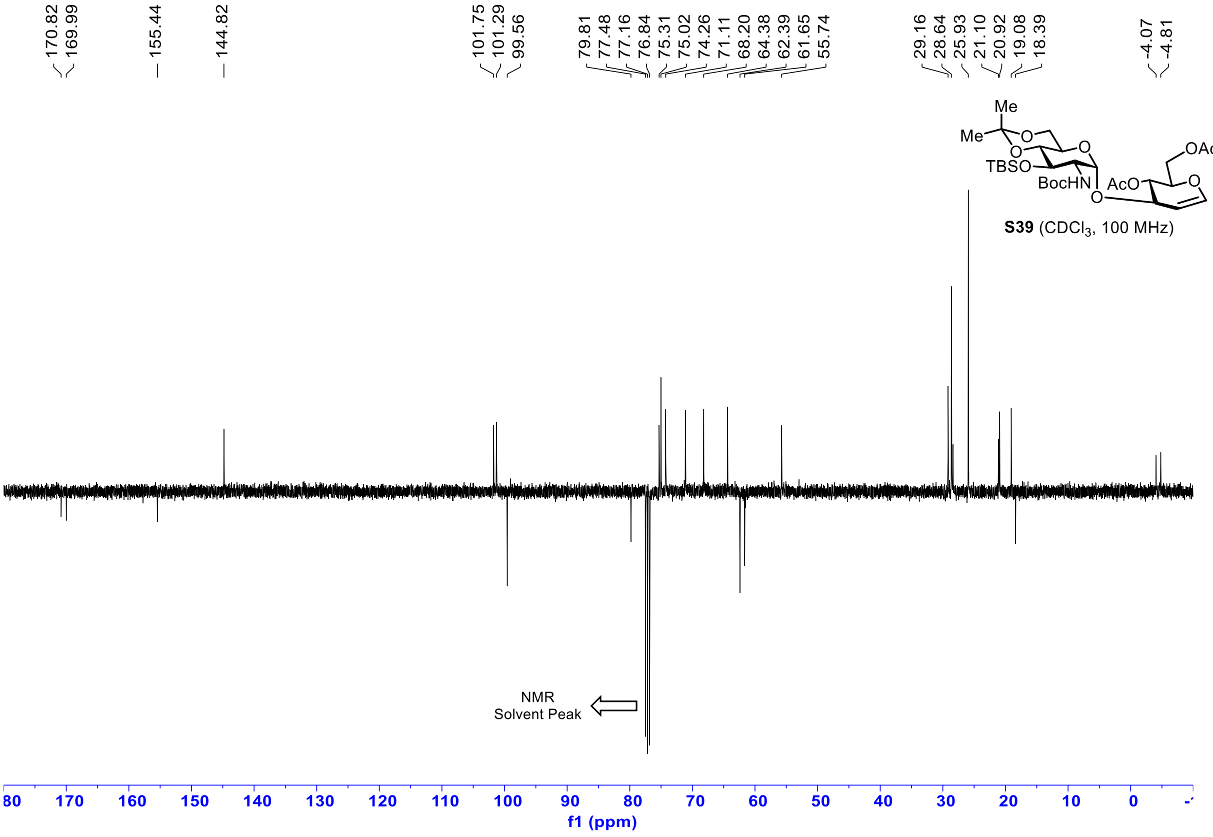
170.82 169.99 155.44 144.82 101.75 101.29 99.56 79.81 77.48 77.16 76.84 75.31 75.02 74.26 71.11 68.20 64.38 62.39 61.65 55.74 29.16 28.64 25.93 21.10 20.92 19.08 18.39 -4.07 -4.81
Me
Me
TBSO
BocHN
AcO
OAc
S39 (CDCl3, 100 MHz)
NMR Solvent Peak
f1 (ppm)

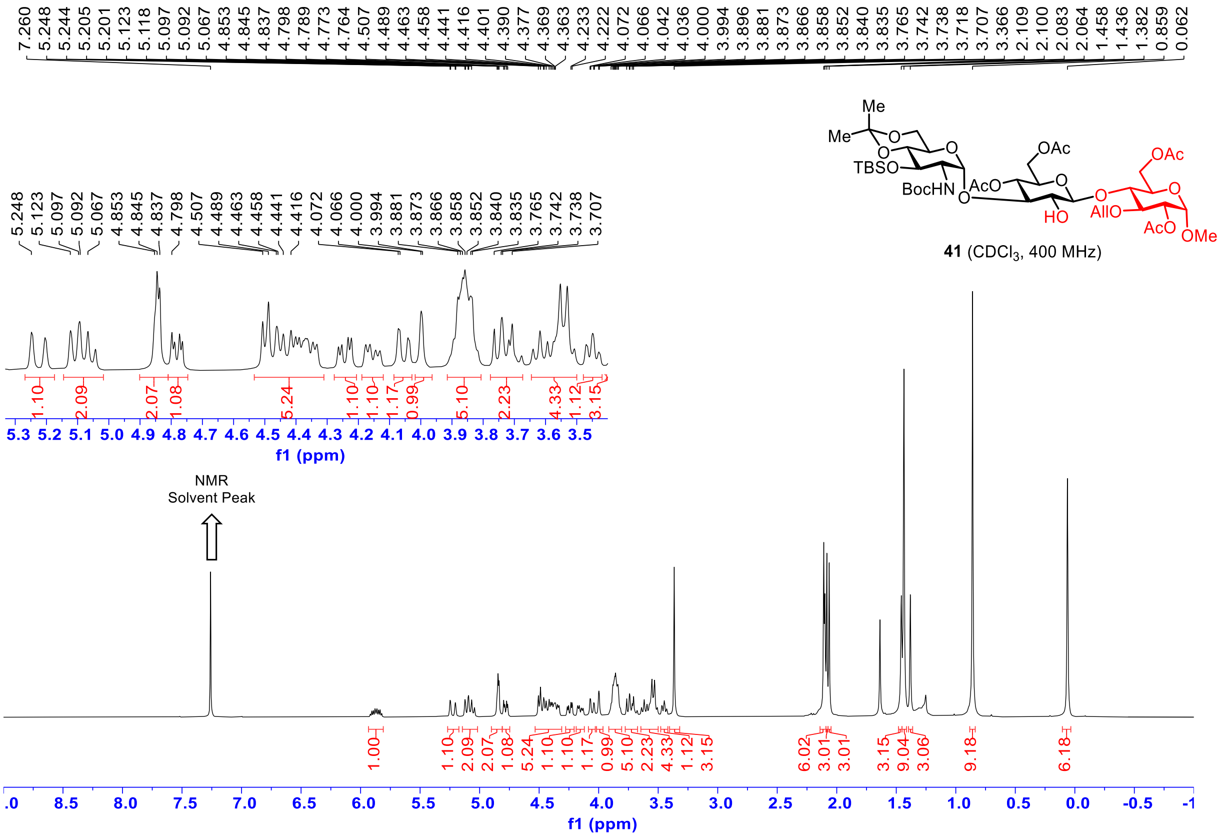
7.260 5.248 5.244 5.205 5.201 5.123 5.118 5.097 5.092 5.067 4.853 4.845 4.837 4.798 4.789 4.773 4.764 4.507 4.489 4.463 4.458 4.441 4.416 4.401 4.390 4.377 4.369 4.363 4.233 4.222 4.072 4.066 4.042 4.036 4.000 3.994 3.896 3.881 3.873 3.866 3.858 3.852 3.840 3.835 3.765 3.742 3.738 3.718 3.707 3.366 2.109 2.100 2.083 2.064 1.458 1.436 1.382 0.859 0.062
41 (CDCl3, 400 MHz)
NMR
Solvent Peak
f1 (ppm)

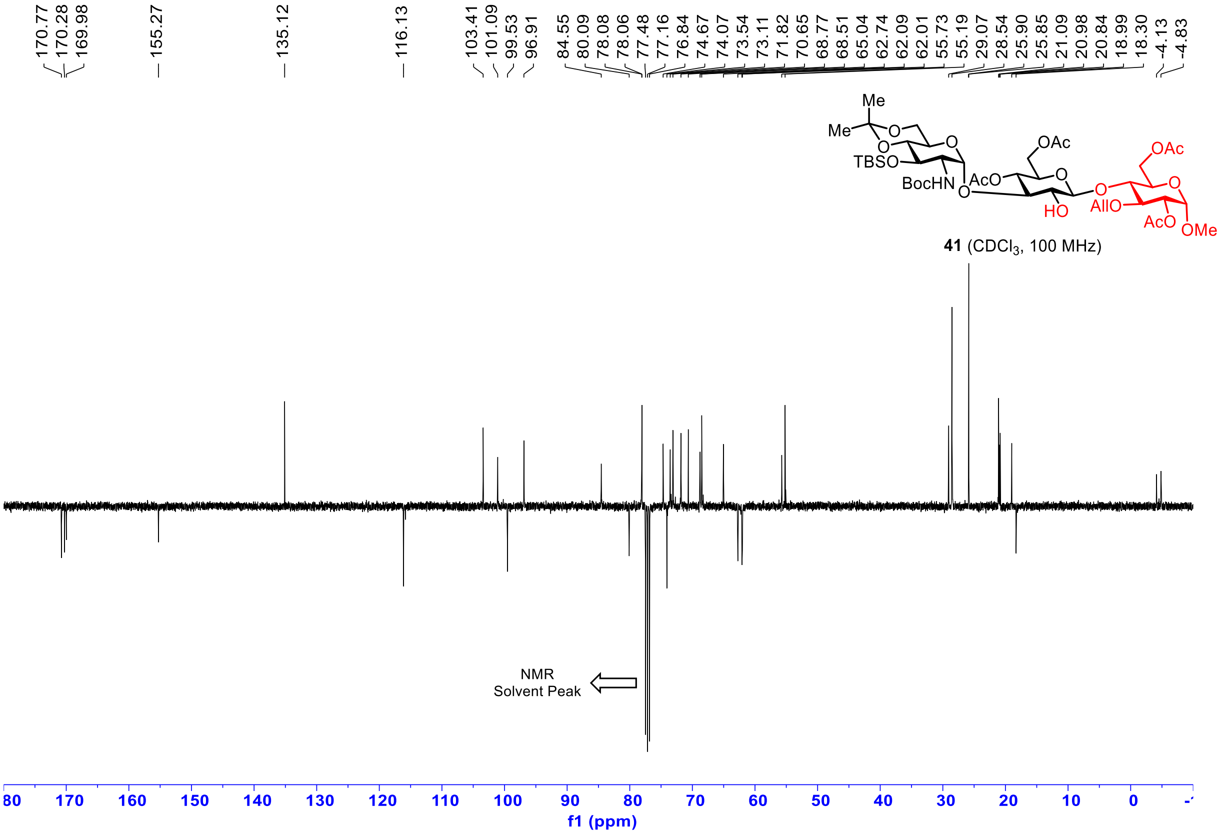
170.77 170.28 169.98 155.27 135.12 116.13 103.41 101.09 99.53 96.91 84.55 80.09 78.08 78.06 77.48 77.16 76.84 74.67 74.07 73.54 73.11 71.82 70.65 68.77 68.51 65.04 62.74 62.09 62.01 55.73 55.19 29.07 28.54 25.90 25.85 21.09 20.98 20.84 18.99 18.30 -4.13 -4.83
41 (CDCl3, 100 MHz)
NMR
Solvent Peak
f1 (ppm)

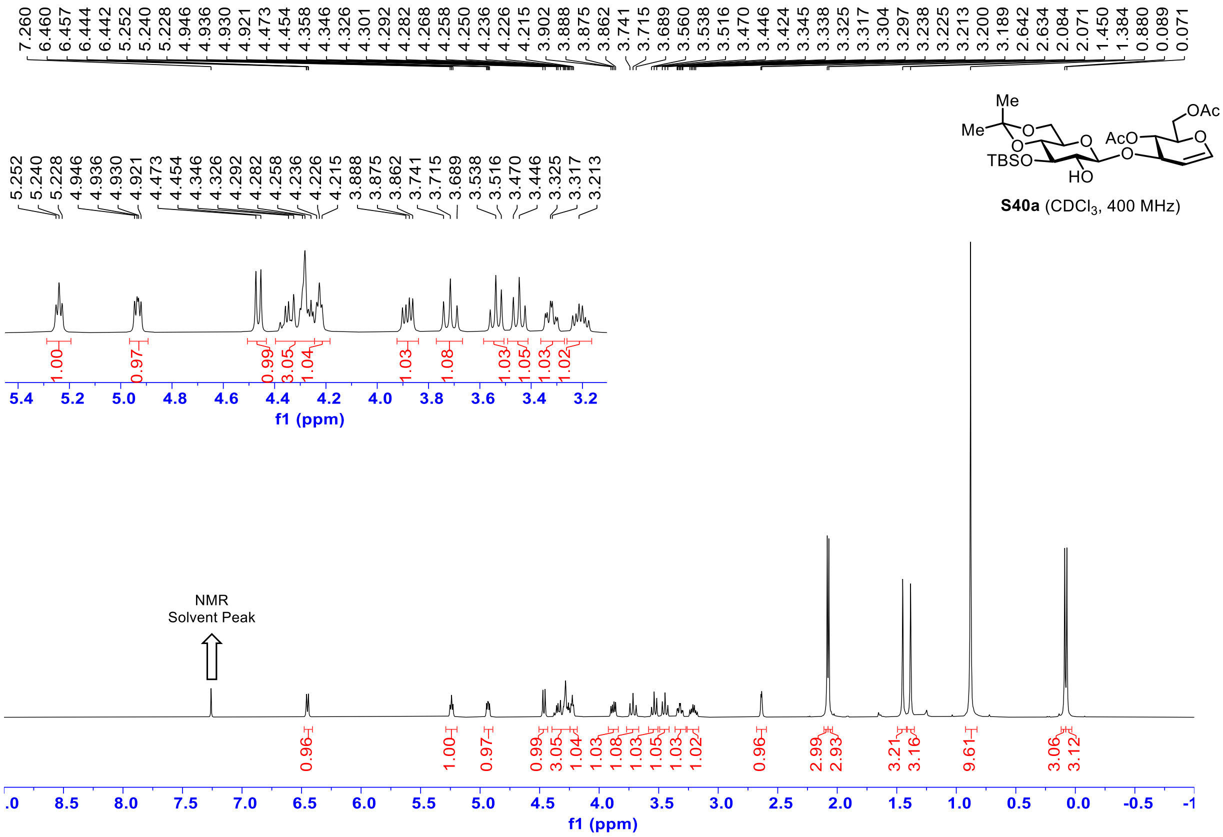

S40a (CDCl3, 400 MHz)
NMR Solvent Peak
f1 (ppm)

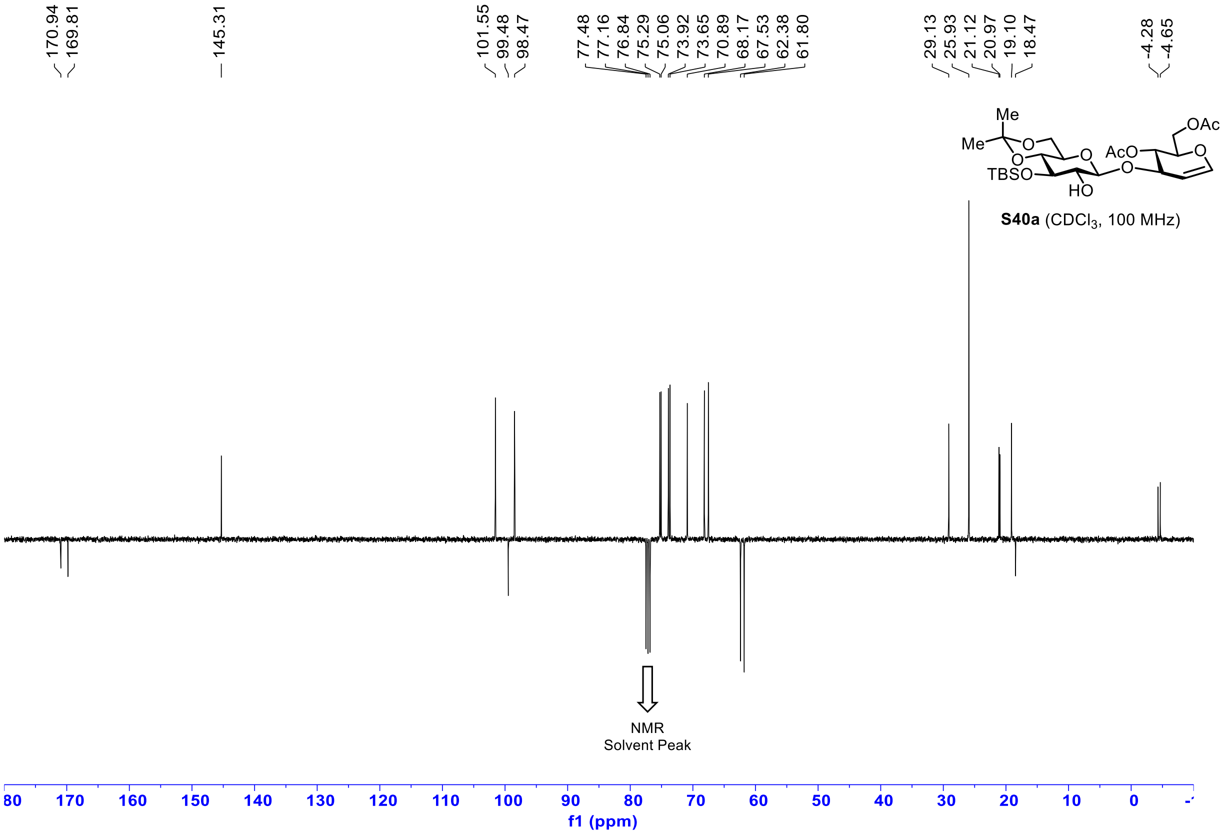

S40a (CDCl3, 100 MHz)
NMR Solvent Peak
f1 (ppm)

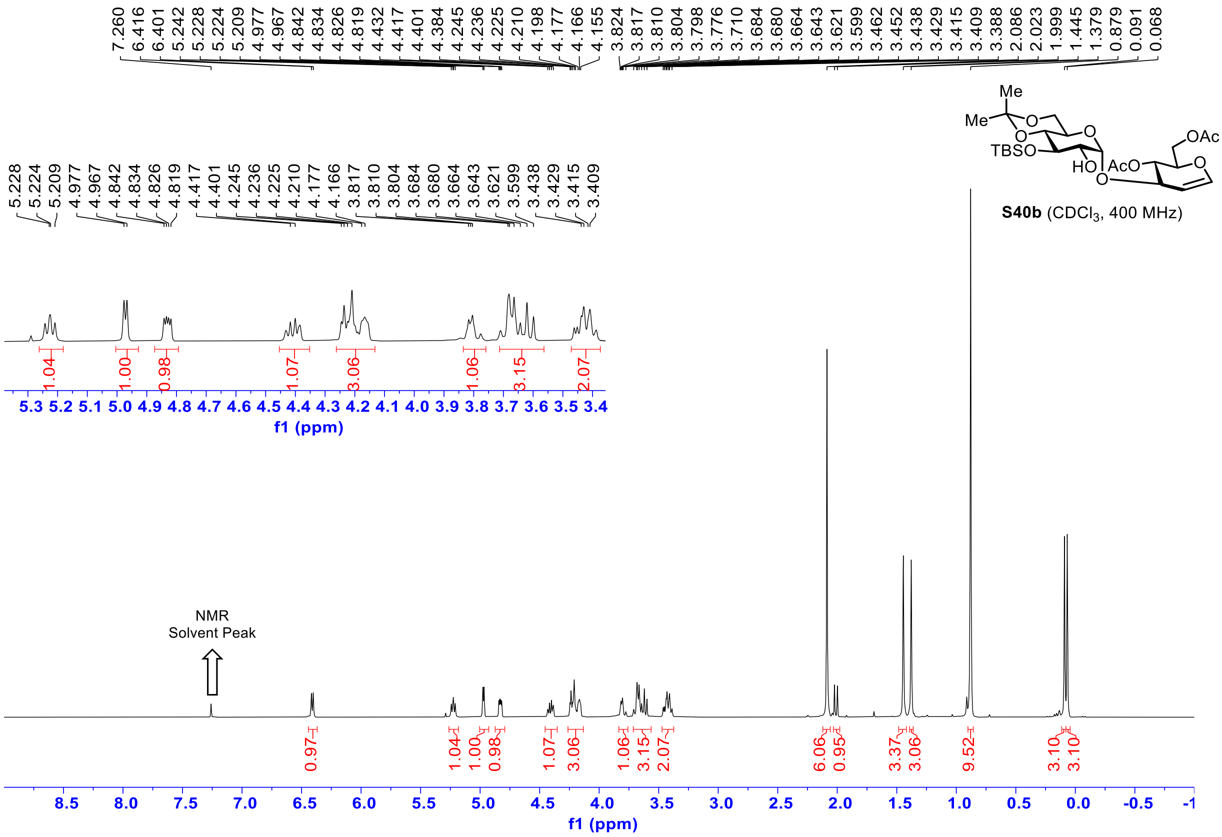
7.260 6.416 6.401 5.242 5.228 5.224 5.209 4.977 4.967 4.842 4.834 4.826 4.819 4.432 4.417 4.401 4.384 4.245 4.236 4.225 4.210 4.198 4.177 4.166 4.155 3.824 3.817 3.810 3.804 3.798 3.776 3.710 3.684 3.680 3.664 3.643 3.621 3.599 3.462 3.452 3.438 3.429 3.415 3.409 3.388 2.086 2.023 1.999 1.445 1.379 0.879 0.091 0.068
5.228 5.224 5.209 4.977 4.967 4.842 4.834 4.826 4.819 4.417 4.401 4.245 4.236 4.225 4.210 4.177 4.166 3.817 3.810 3.804 3.684 3.680 3.664 3.643 3.621 3.599 3.438 3.429 3.415 3.409
1.04 1.00 0.98 1.07 3.06 1.06 3.15 2.07
f1 (ppm)
Me
Me
O
O
TBSO
O
HO
AcO
O
OAc
O
S40b (CDCl$_3$, 400 MHz)
NMR
Solvent Peak
0.97 1.04 1.00 0.98 1.07 3.06 1.06 3.15 2.07 6.06 0.95 3.37 3.06 9.52 3.10 3.10
f1 (ppm)

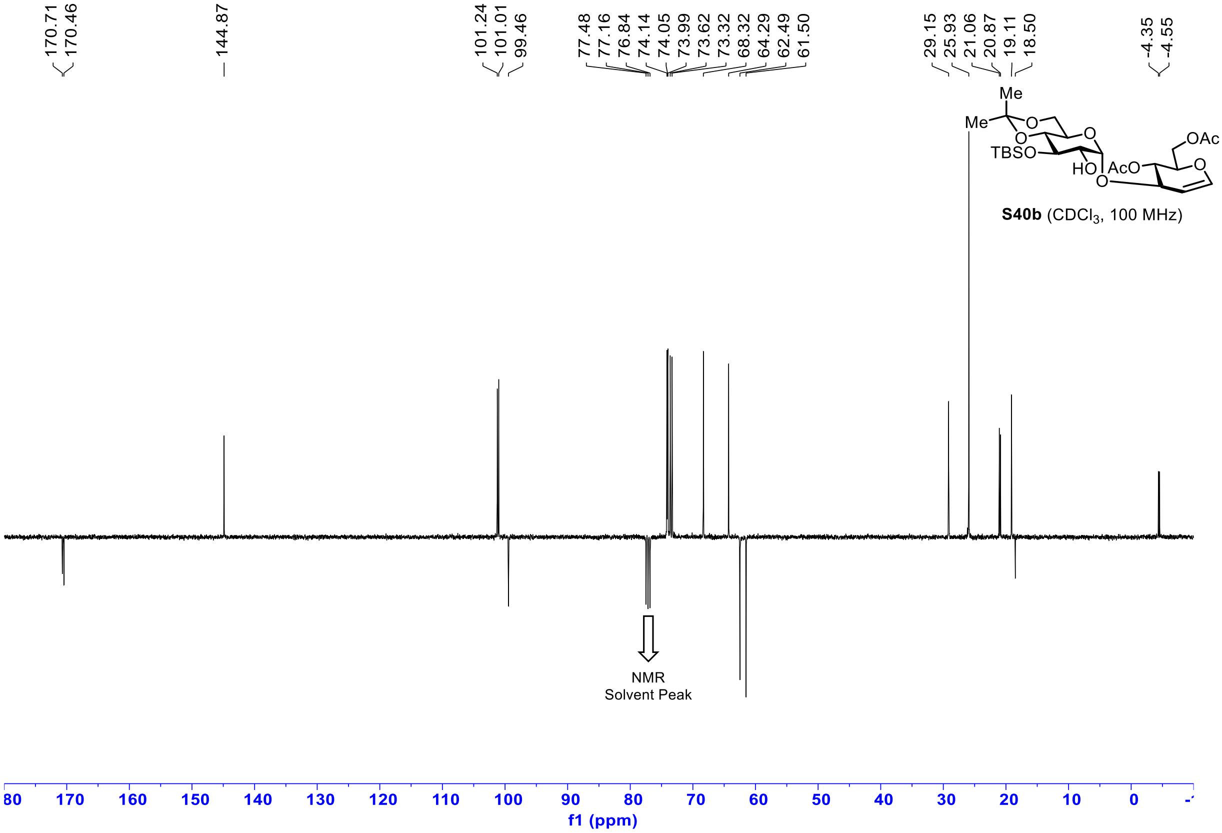
170.71 170.46 144.87 101.24 101.01 99.46 77.48 77.16 76.84 74.14 74.05 73.99 73.62 73.32 68.32 64.29 62.49 61.50 29.15 25.93 21.06 20.87 19.11 18.50 -4.35 -4.55
Me
Me
O
O
TBSO
O
HO
AcO
O
OAc
O
S40b (CDCl$_3$, 100 MHz)
NMR
Solvent Peak
f1 (ppm)

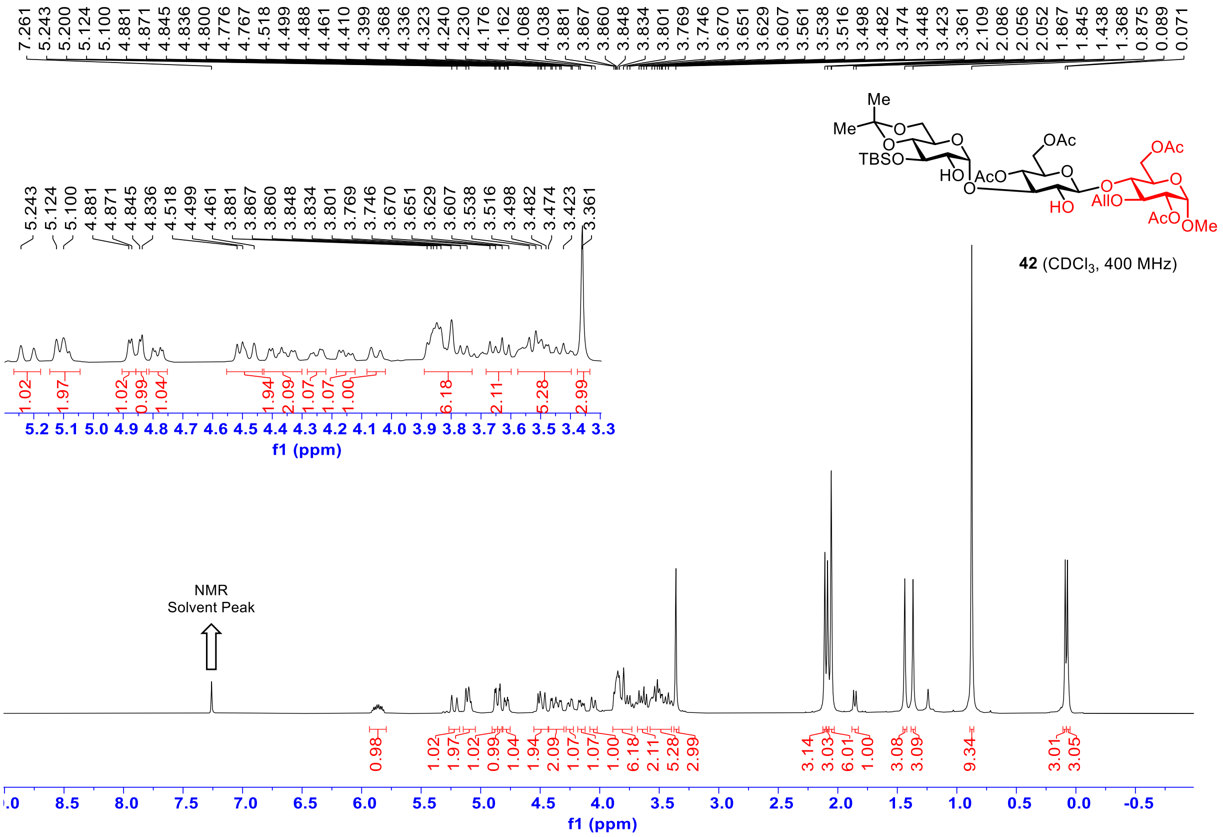
42 (CDCl3, 400 MHz)
NMR Solvent Peak
f1 (ppm)

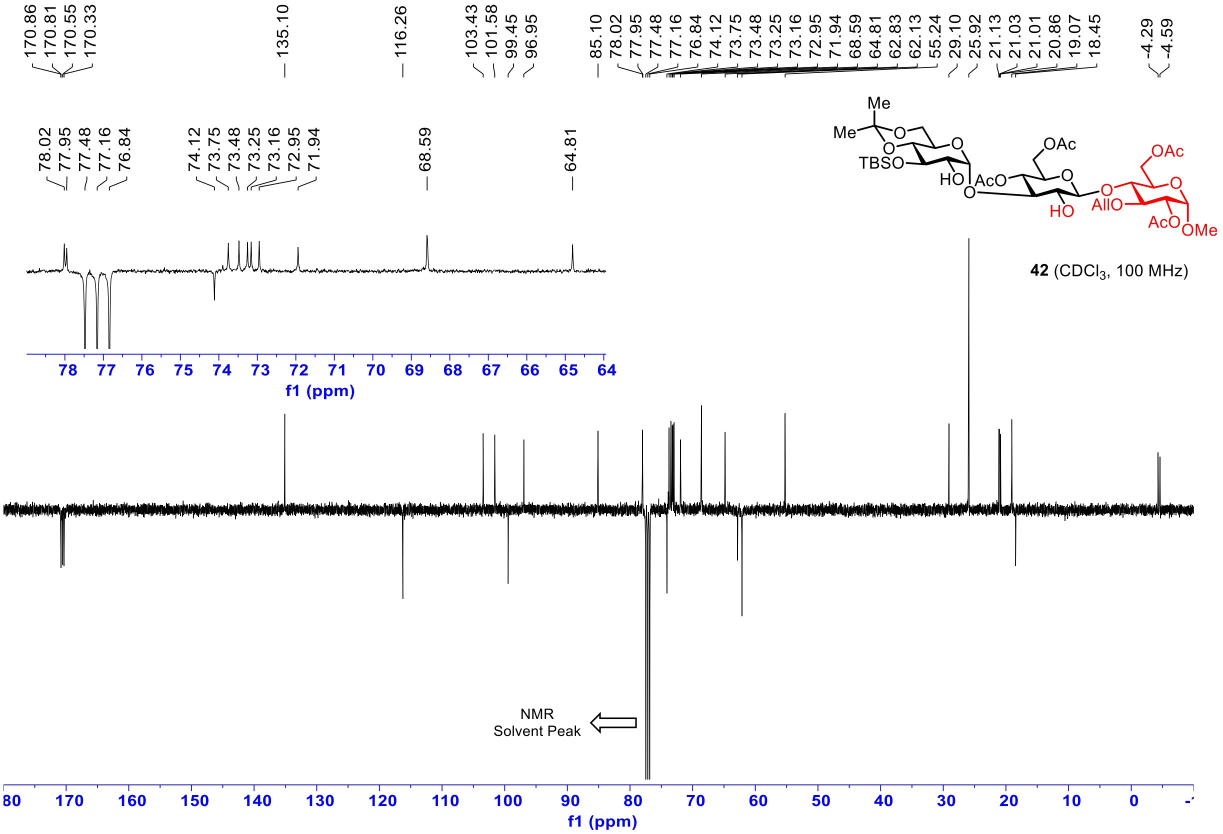
42 (CDCl3, 100 MHz)
NMR Solvent Peak
f1 (ppm)

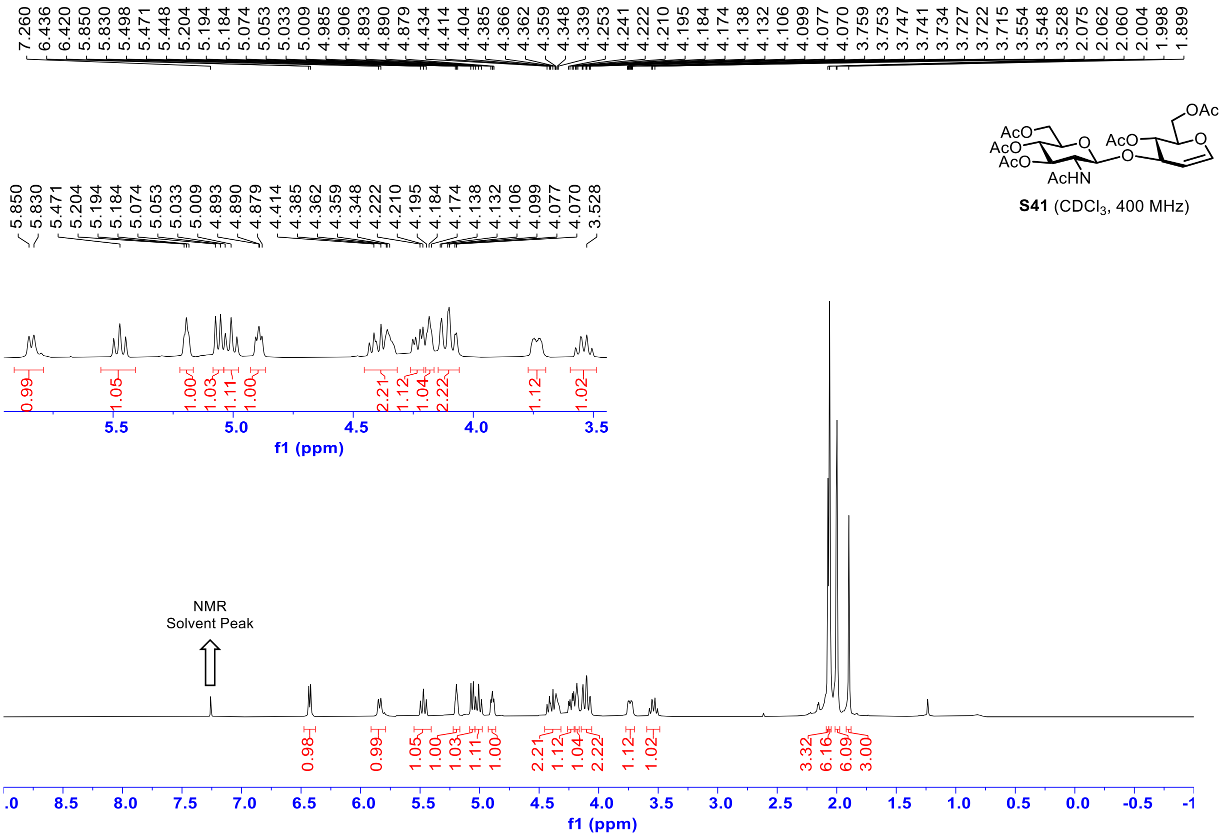

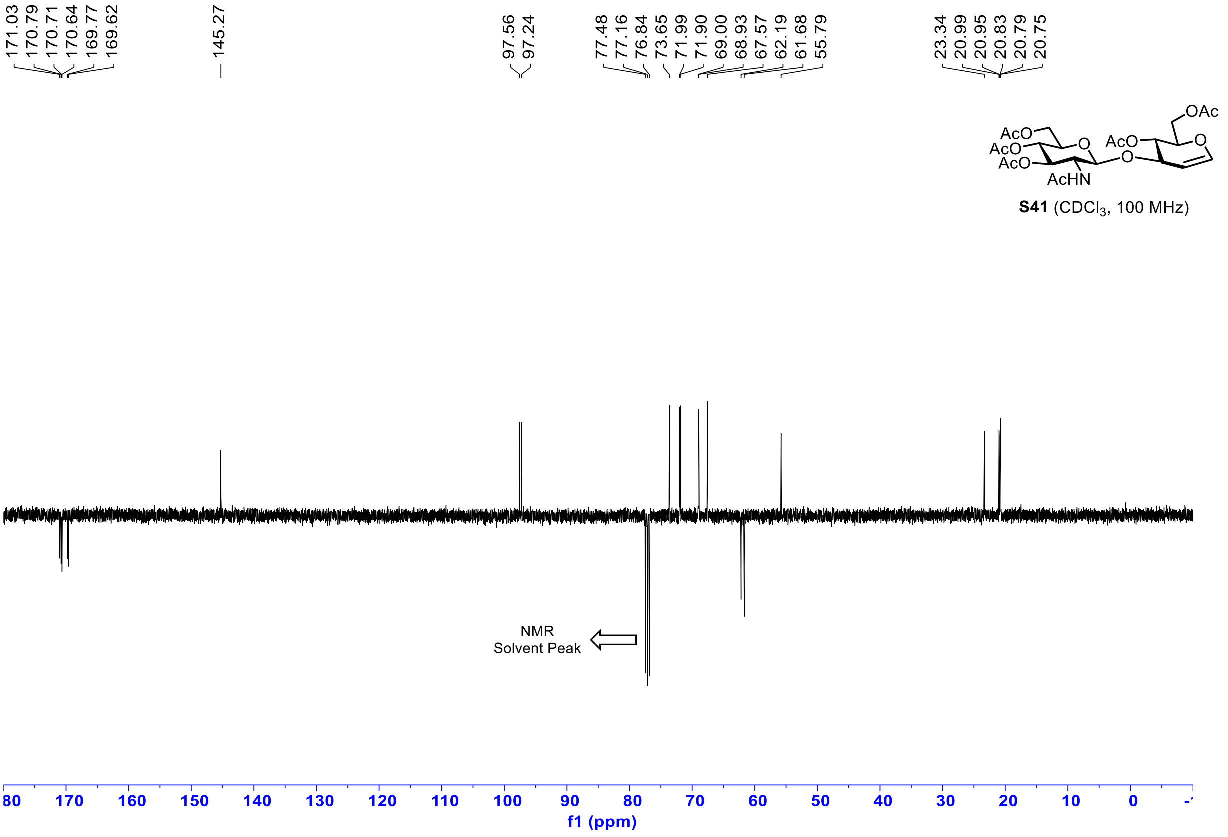

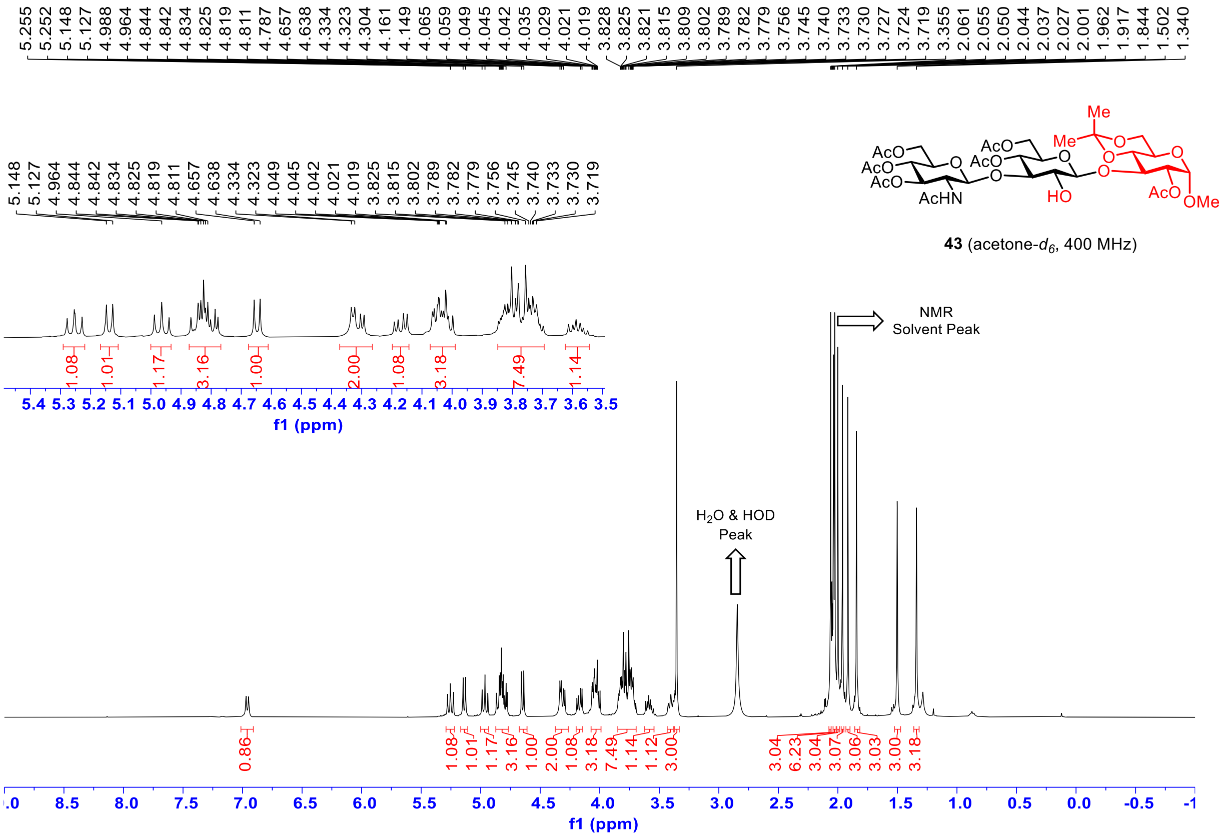

43 (acetone-d_6, 400 MHz)

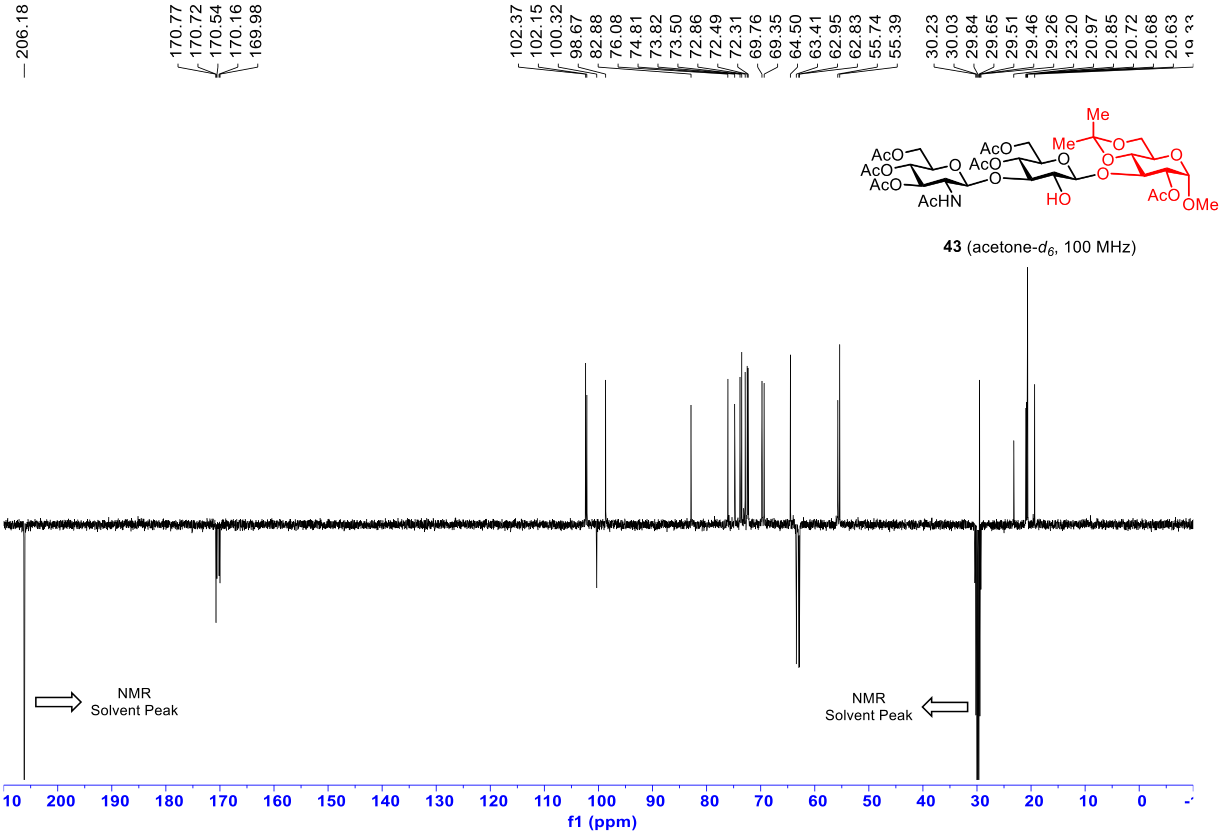

43 (acetone-d_6, 100 MHz)

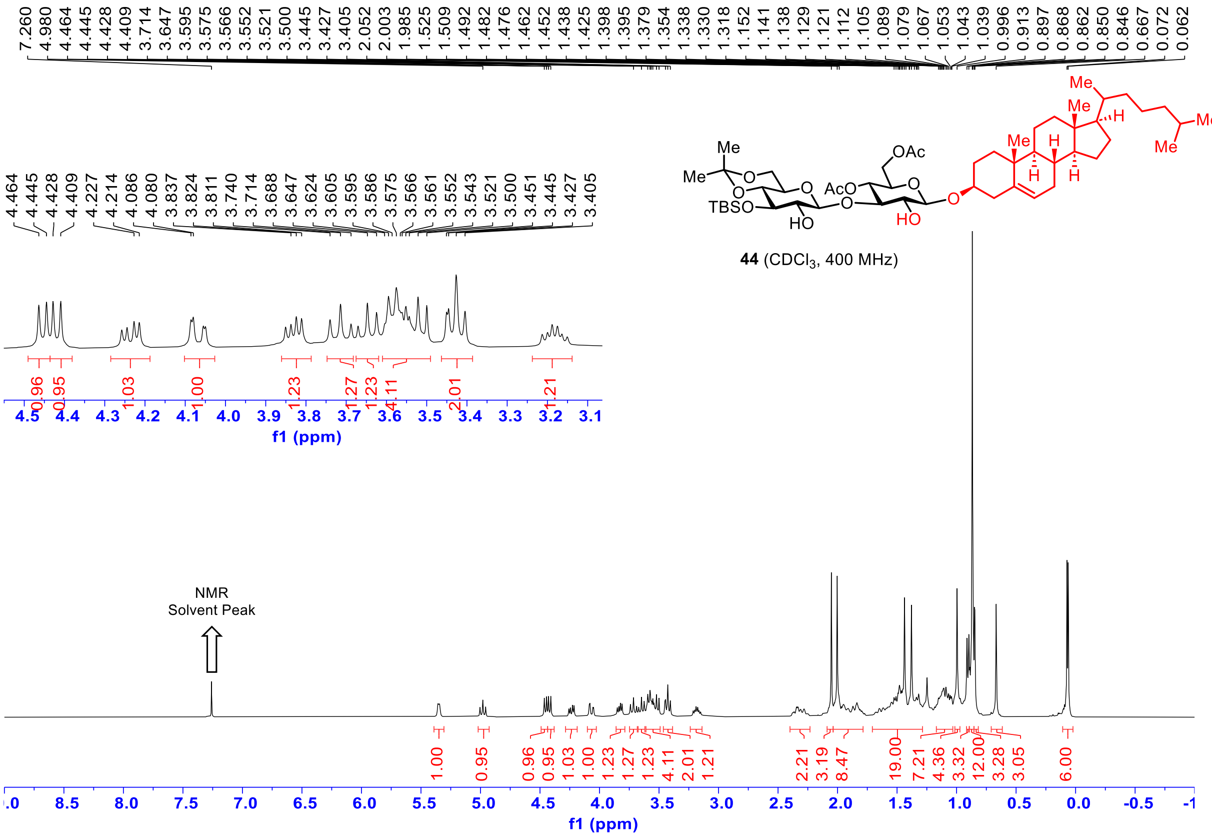
7.260 4.980 4.464 4.445 4.428 4.409 3.714 3.647 3.595 3.575 3.566 3.552 3.521 3.500 3.445 3.427 3.405 2.052 2.003 1.985 1.525 1.509 1.492 1.482 1.476 1.462 1.452 1.438 1.425 1.398 1.395 1.379 1.354 1.338 1.330 1.318 1.152 1.141 1.138 1.129 1.121 1.112 1.105 1.089 1.079 1.067 1.053 1.043 1.039 0.996 0.913 0.897 0.868 0.862 0.850 0.846 0.667 0.072 0.062
4.464 4.445 4.428 4.409 4.227 4.214 4.086 4.080 3.837 3.824 3.811 3.740 3.714 3.688 3.647 3.624 3.605 3.595 3.586 3.575 3.566 3.561 3.552 3.543 3.521 3.500 3.451 3.445 3.427 3.405
44 (CDCl3, 400 MHz)
NMR Solvent Peak
f1 (ppm)

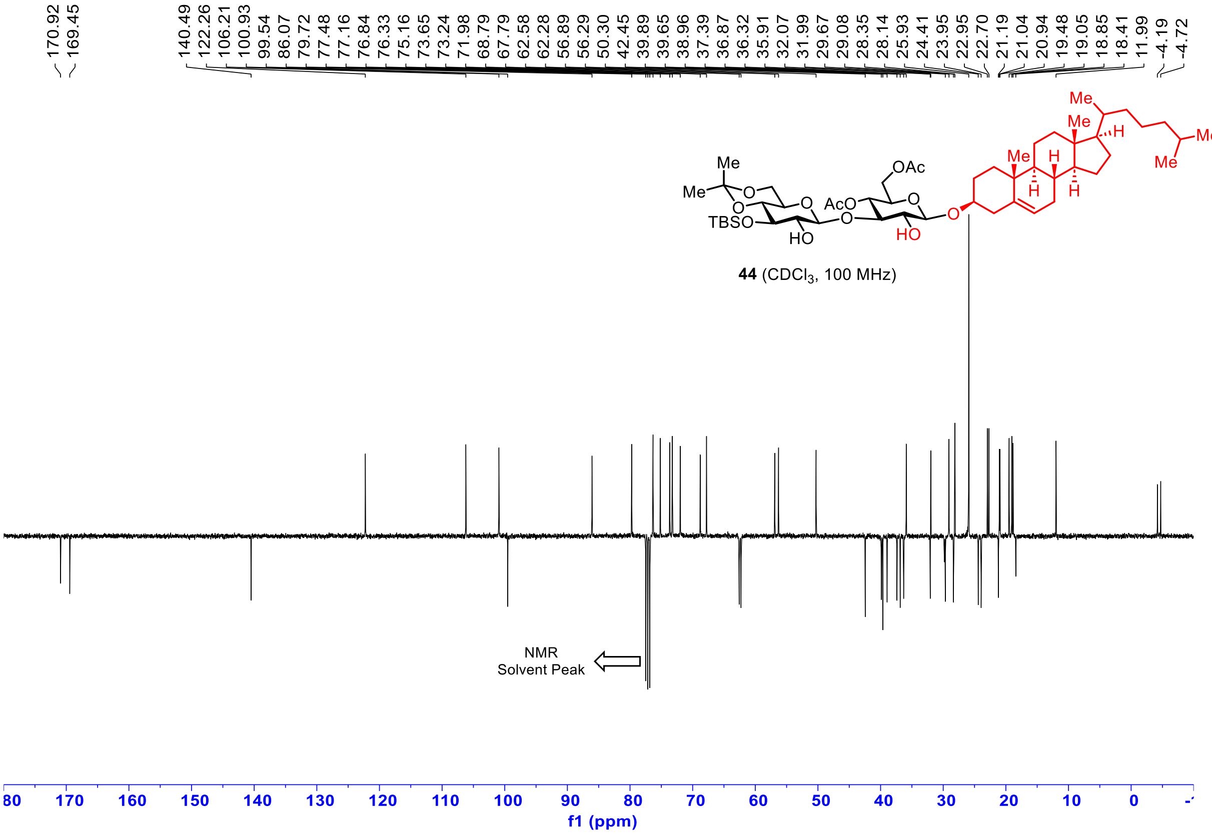
170.92 169.45 140.49 122.26 106.21 100.93 99.54 86.07 79.72 77.48 77.16 76.84 76.33 75.16 73.65 73.24 71.98 68.79 67.79 62.58 62.28 56.89 56.29 50.30 42.45 39.89 39.65 38.96 37.39 36.87 36.32 35.91 32.07 31.99 29.67 29.08 28.35 28.14 25.93 24.41 23.95 22.95 22.70 21.19 21.04 20.94 19.48 19.05 18.85 18.41 11.99 -4.19 -4.72
44 (CDCl3, 100 MHz)
NMR Solvent Peak
f1 (ppm)

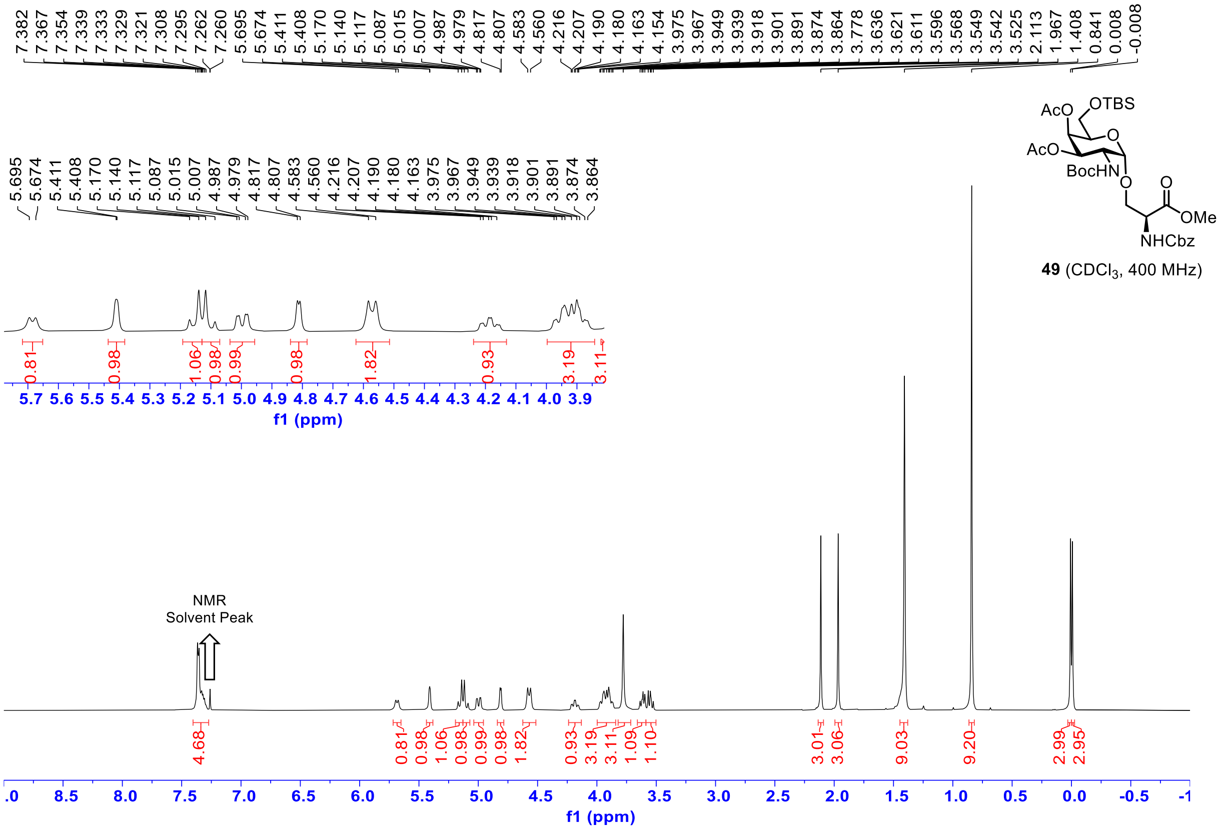
7.382 7.367 7.354 7.339 7.333 7.329 7.321 7.308 7.295 7.262 7.260 5.695 5.674 5.411 5.408 5.170 5.140 5.117 5.087 5.015 5.007 4.987 4.979 4.817 4.807 4.583 4.560 4.216 4.207 4.190 4.180 4.163 4.154 3.975 3.967 3.949 3.939 3.918 3.901 3.891 3.874 3.864 3.778 3.636 3.621 3.611 3.596 3.568 3.549 3.542 3.525 2.113 1.967 1.408 0.841 0.008 -0.008
AcO
OTBS
O
AcO
BocHN
O
O
OMe
NHCbz
49 (CDCl3, 400 MHz)
NMR
Solvent Peak
f1 (ppm)

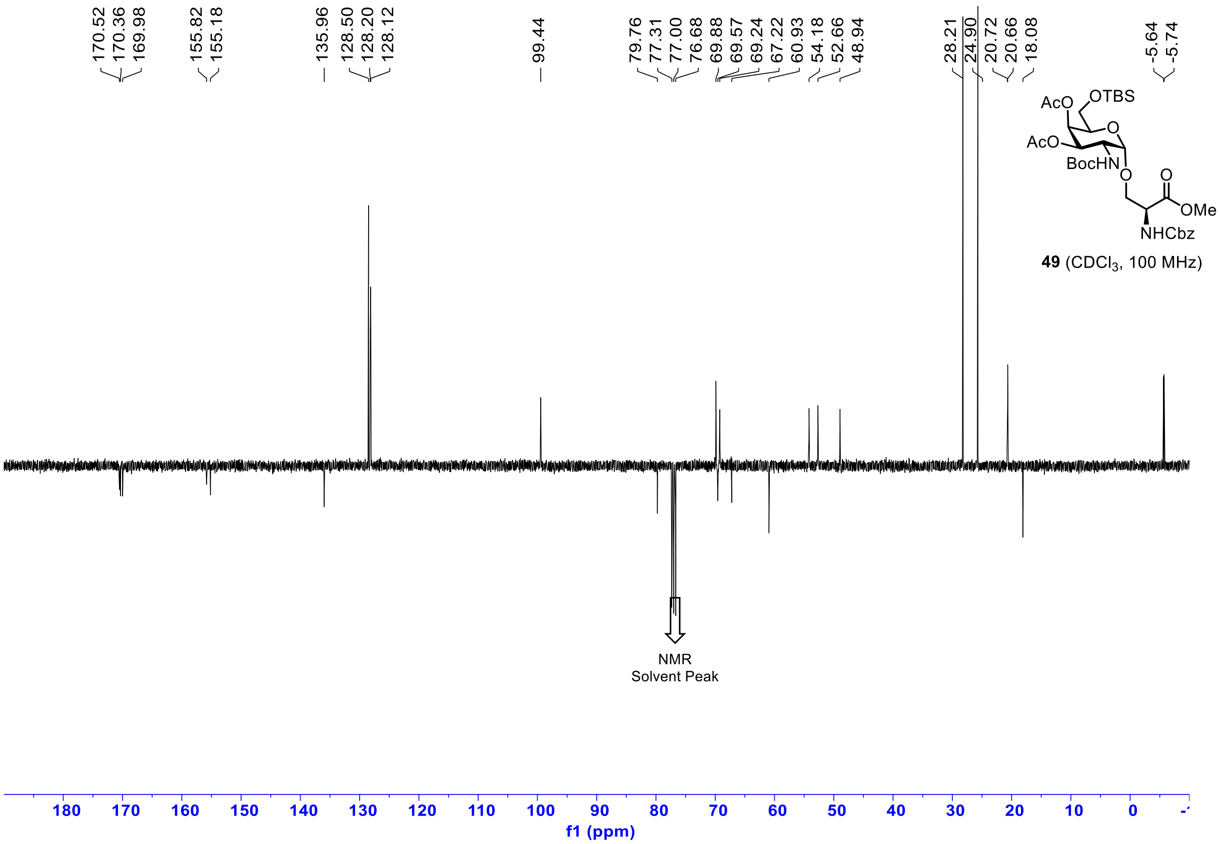
170.52 170.36 169.98 155.82 155.18 135.96 128.50 128.20 128.12 99.44 79.76 77.31 77.00 76.68 69.88 69.57 69.24 67.22 60.93 54.18 52.66 48.94 28.21 24.90 20.72 20.66 18.08 -5.64 -5.74
AcO
OTBS
O
AcO
BocHN
O
O
OMe
NHCbz
49 (CDCl3, 100 MHz)
NMR
Solvent Peak
f1 (ppm)

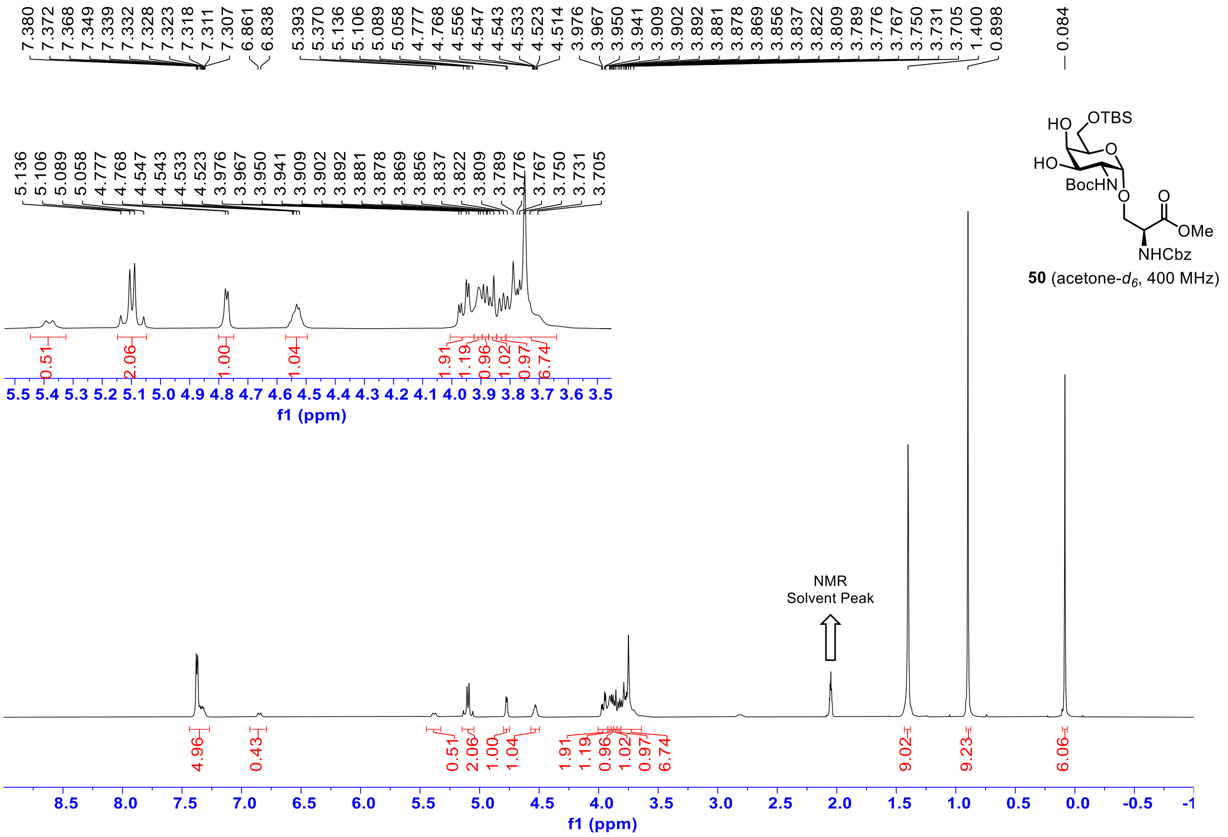
7.380 7.372 7.368 7.349 7.339 7.332 7.328 7.323 7.318 7.311 7.307 6.861 6.838 5.393 5.370 5.136 5.106 5.089 5.058 4.777 4.768 4.556 4.547 4.543 4.533 4.523 4.514 3.976 3.967 3.950 3.941 3.909 3.902 3.892 3.881 3.878 3.869 3.856 3.837 3.822 3.809 3.789 3.776 3.767 3.750 3.731 3.705 1.400 0.898 0.084
HO OTBS
HO
BocHN
O
O
OMe
NHCbz
50 (acetone-d_6, 400 MHz)
NMR
Solvent Peak
0.51 2.06 1.00 1.04 1.91 1.19 0.96 1.02 0.97 6.74
4.96 0.43 0.51 2.06 1.00 1.04 1.91 1.19 0.96 1.02 0.97 6.74 9.02 9.23 6.06
f1 (ppm)

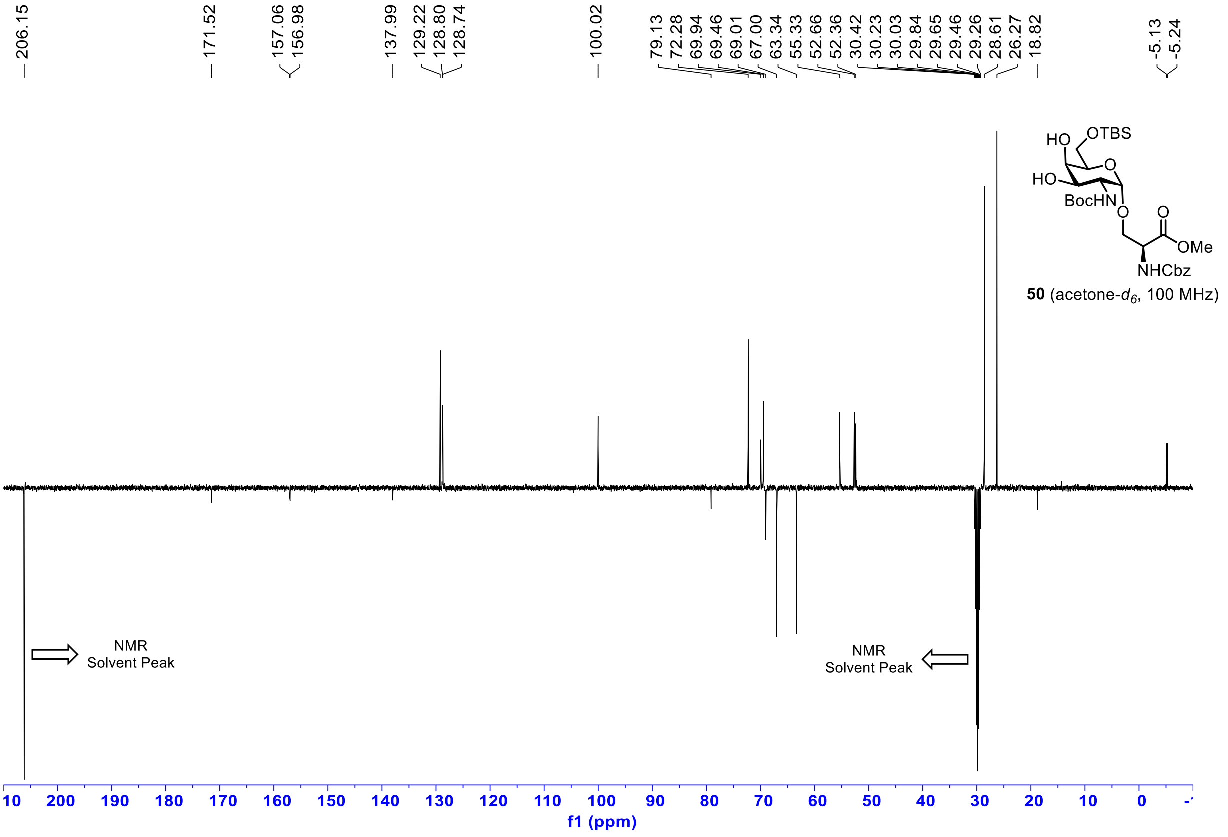
206.15 171.52 157.06 156.98 137.99 129.22 128.80 128.74 100.02 79.13 72.28 69.94 69.46 69.01 67.00 63.34 55.33 52.66 52.36 30.42 30.23 30.03 29.84 29.65 29.46 29.26 28.61 26.27 18.82 -5.13 -5.24
HO OTBS
HO
BocHN
O
O
OMe
NHCbz
50 (acetone-d_6, 100 MHz)
NMR
Solvent Peak
NMR
Solvent Peak
f1 (ppm)

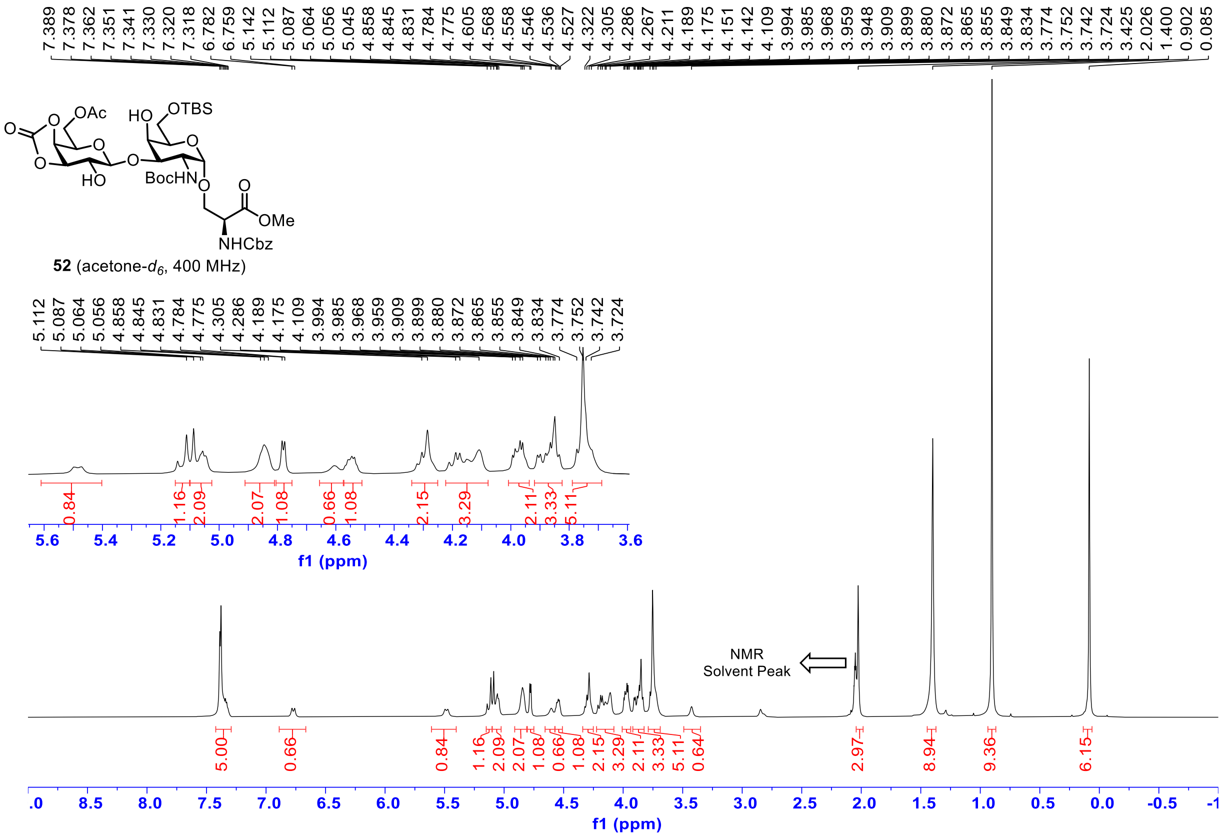

52 (acetone-d_6, 400 MHz)

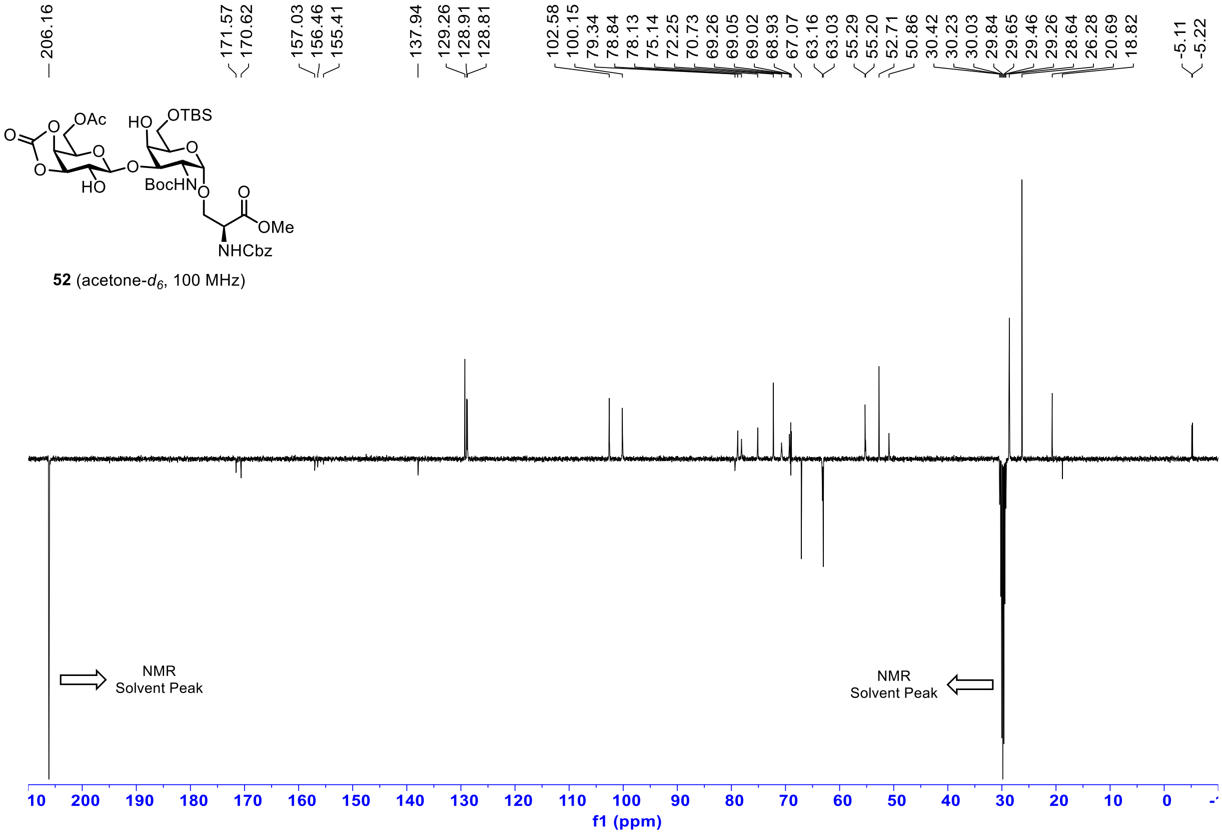

52 (acetone-d_6, 100 MHz)

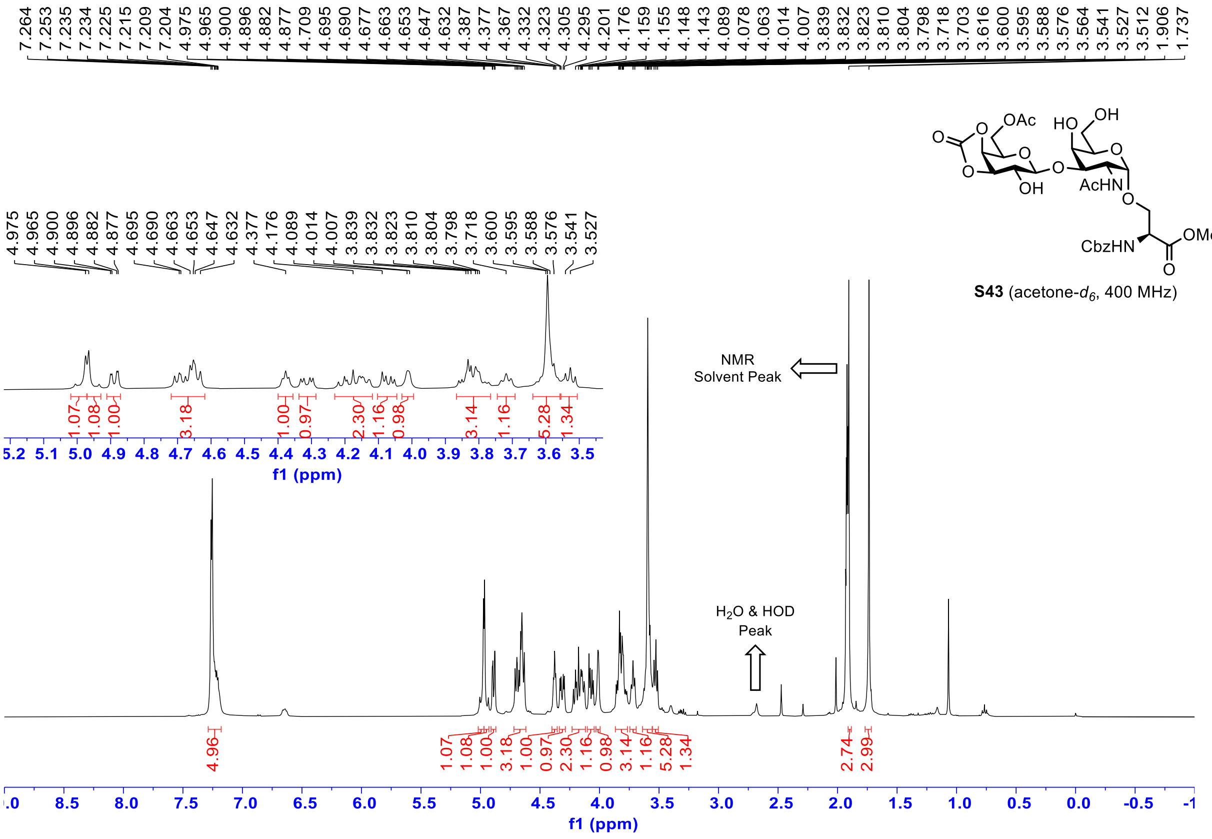
S43 (acetone-d_6, 400 MHz)
NMR Solvent Peak
H_2O & HOD Peak
f1 (ppm)
OAc
HO
OH
AcHN
CbzHN
OMe

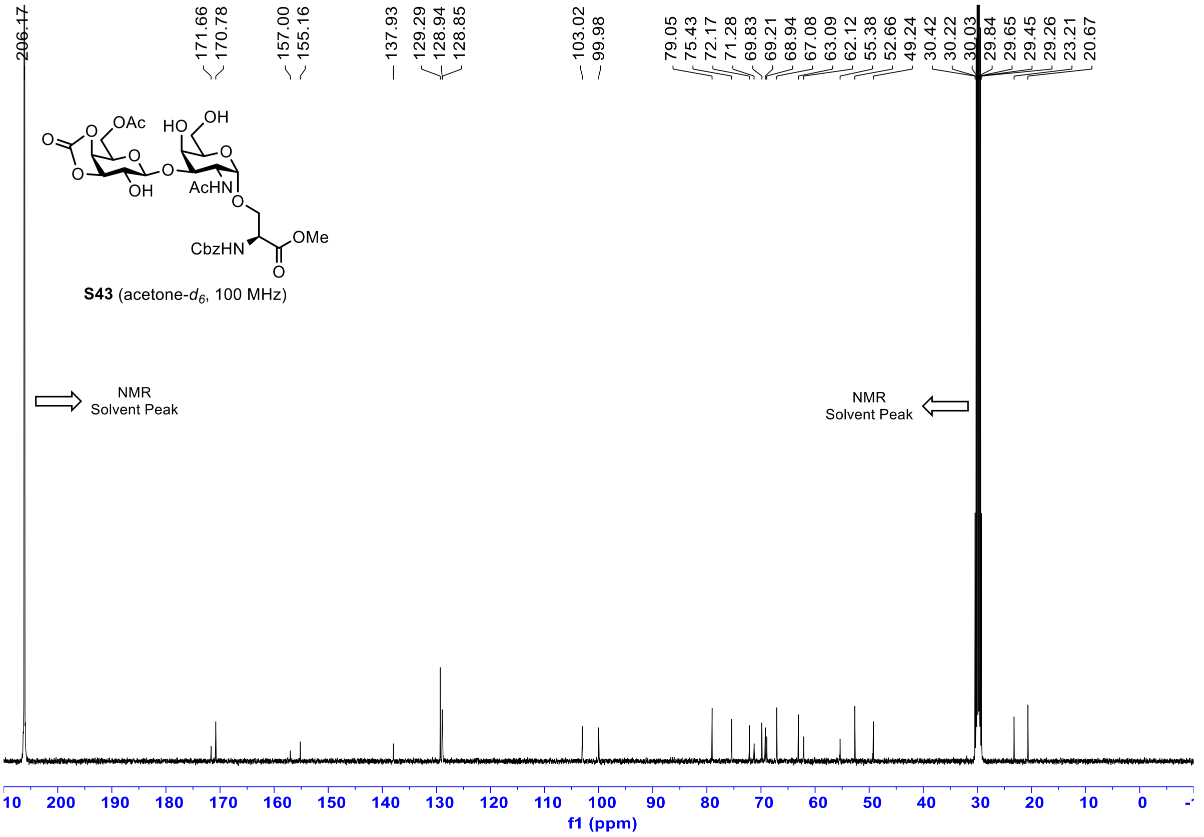
S43 (acetone-d_6, 100 MHz)
NMR Solvent Peak
NMR Solvent Peak
f1 (ppm)
OAc
HO
OH
AcHN
CbzHN
OMe

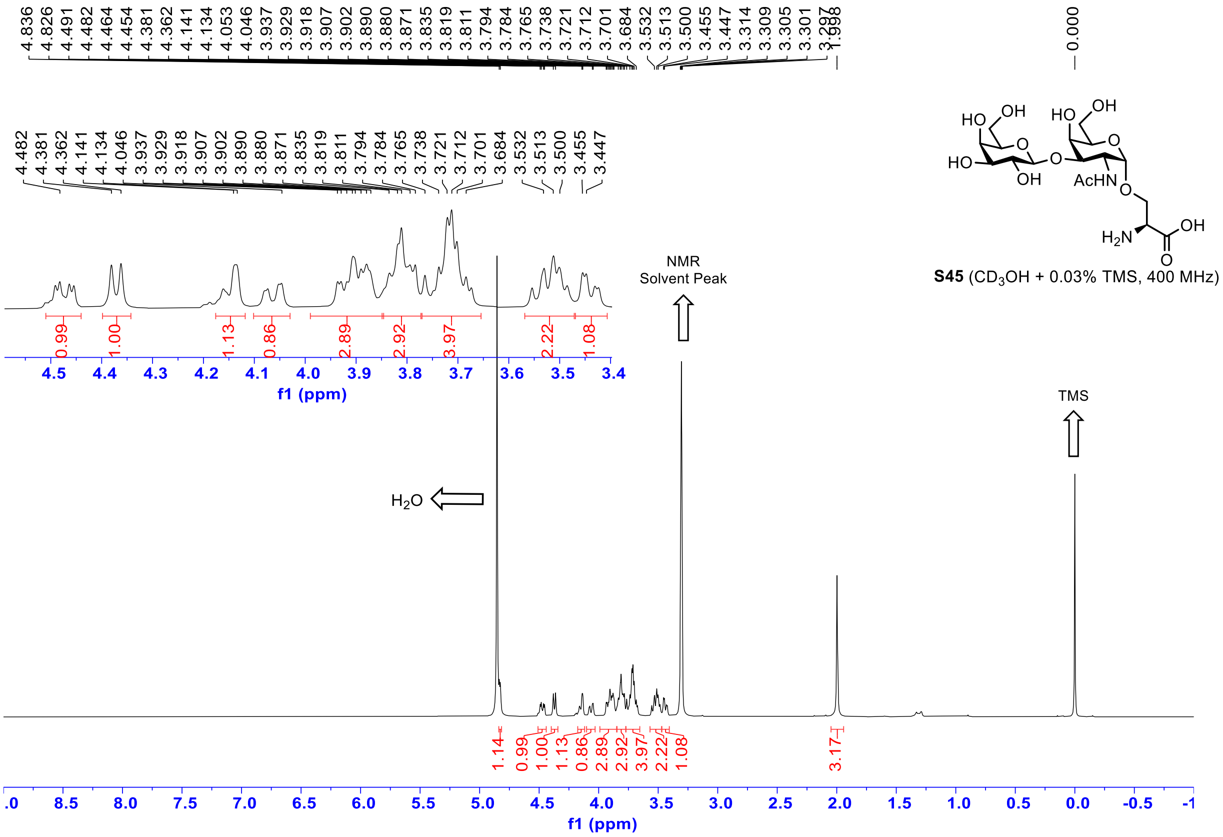
H2O
NMR
Solvent Peak
TMS
S45 (CD3OH + 0.03% TMS, 400 MHz)
f1 (ppm)

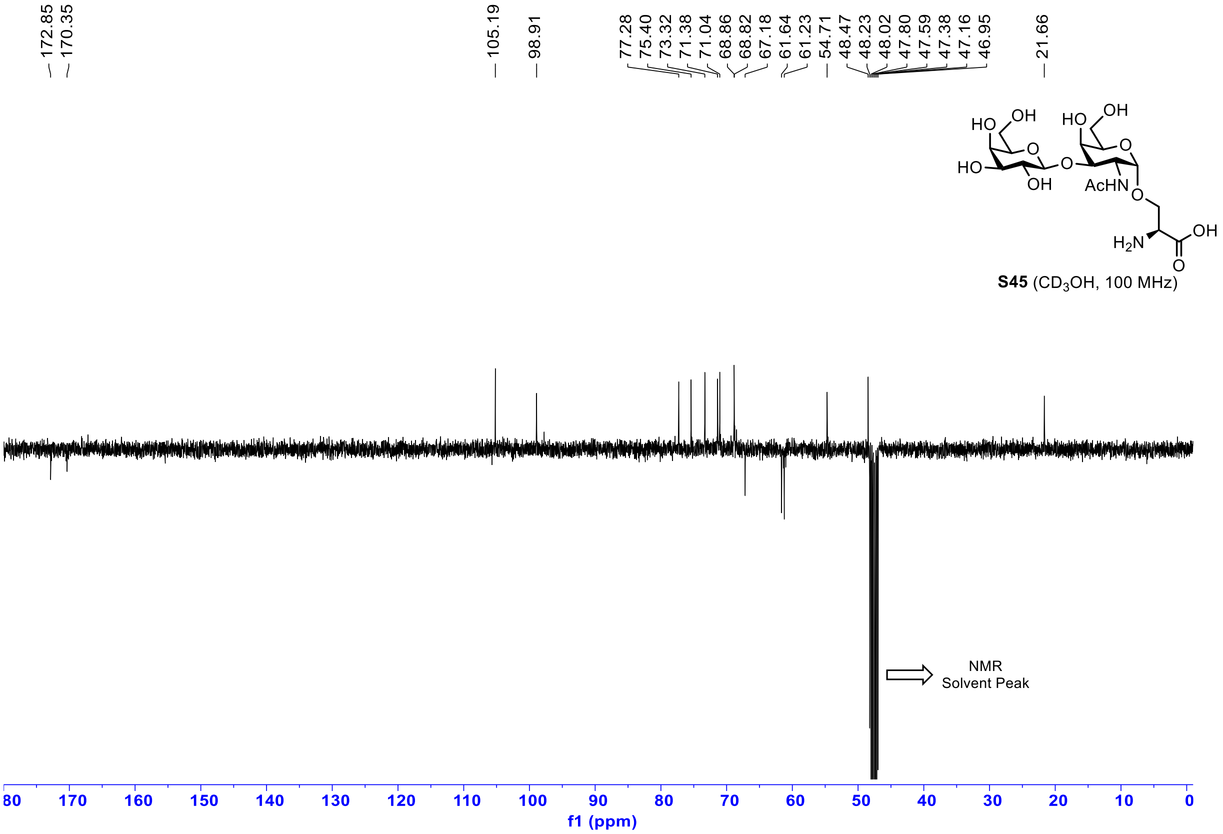
NMR
Solvent Peak
S45 (CD3OH, 100 MHz)
f1 (ppm)

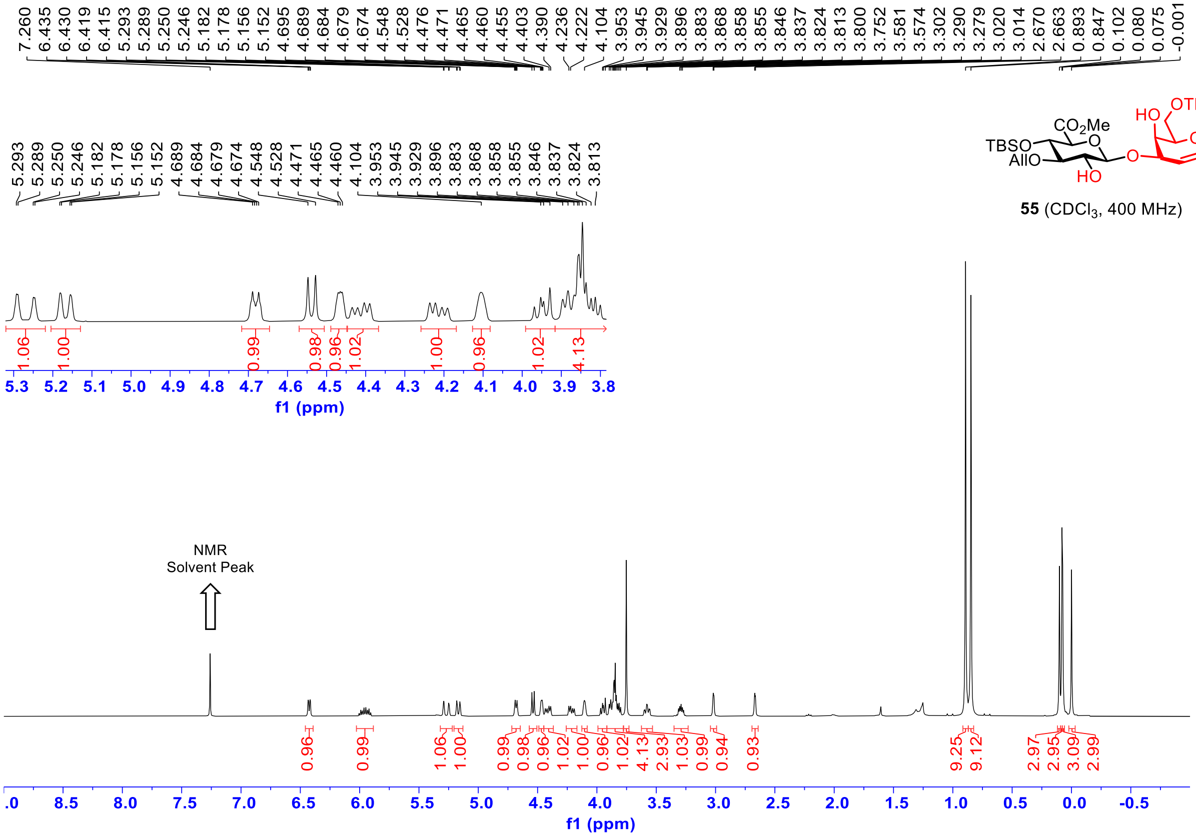

55 ($CDCl_3$, 400 MHz)

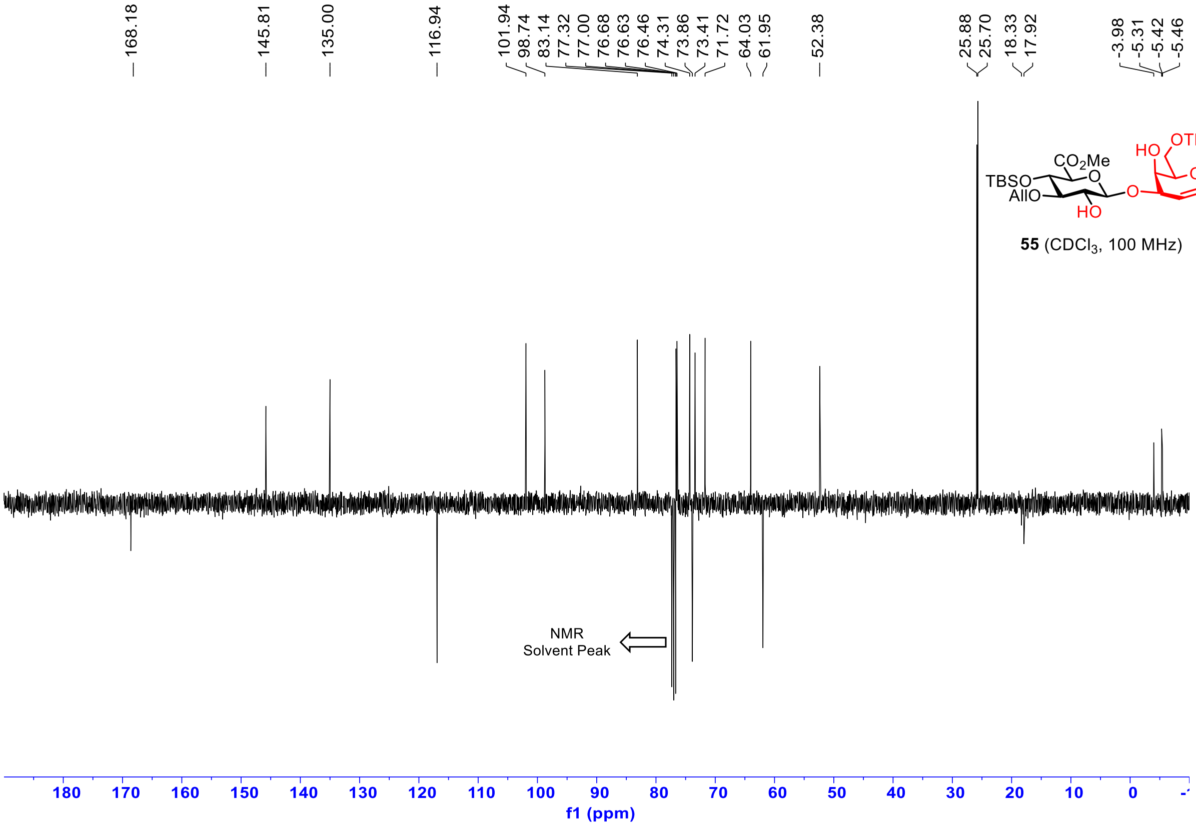

55 ($CDCl_3$, 100 MHz)

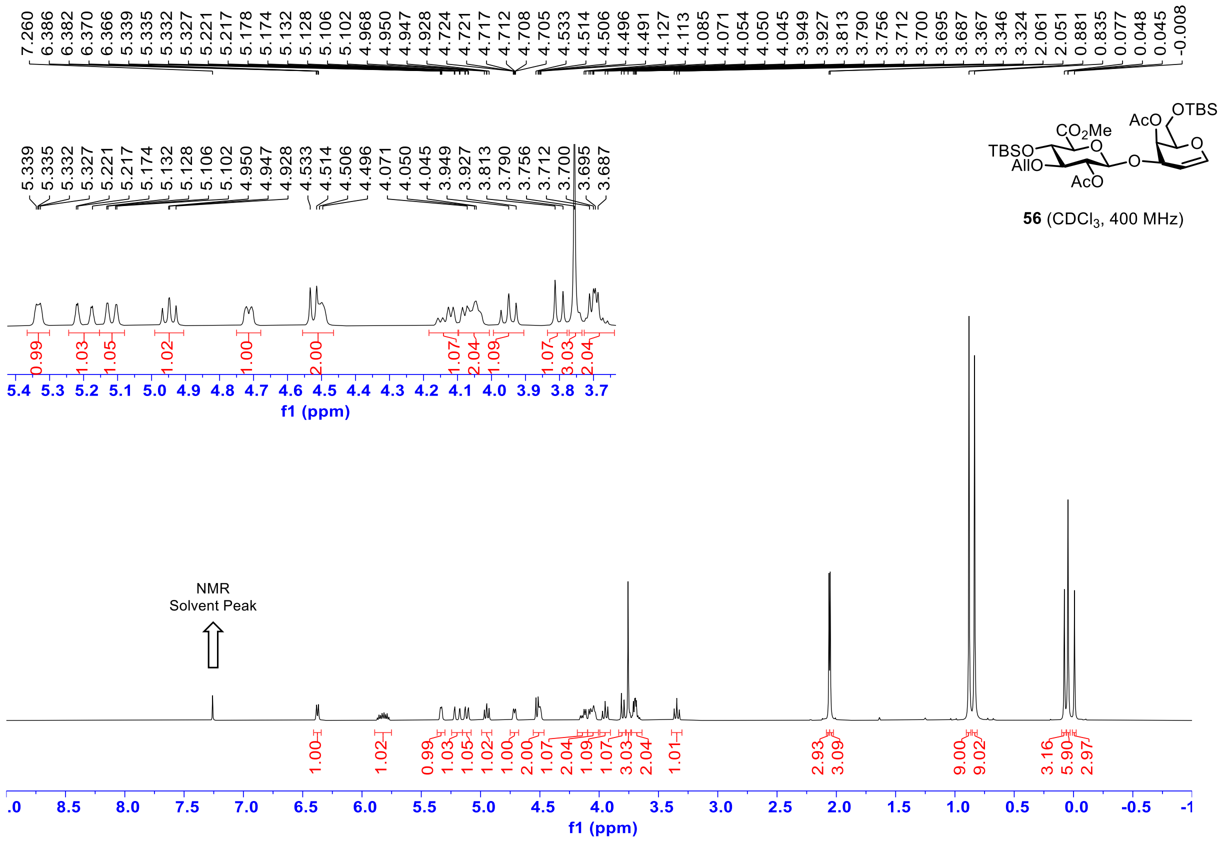

56 ($CDCl_3$, 400 MHz)

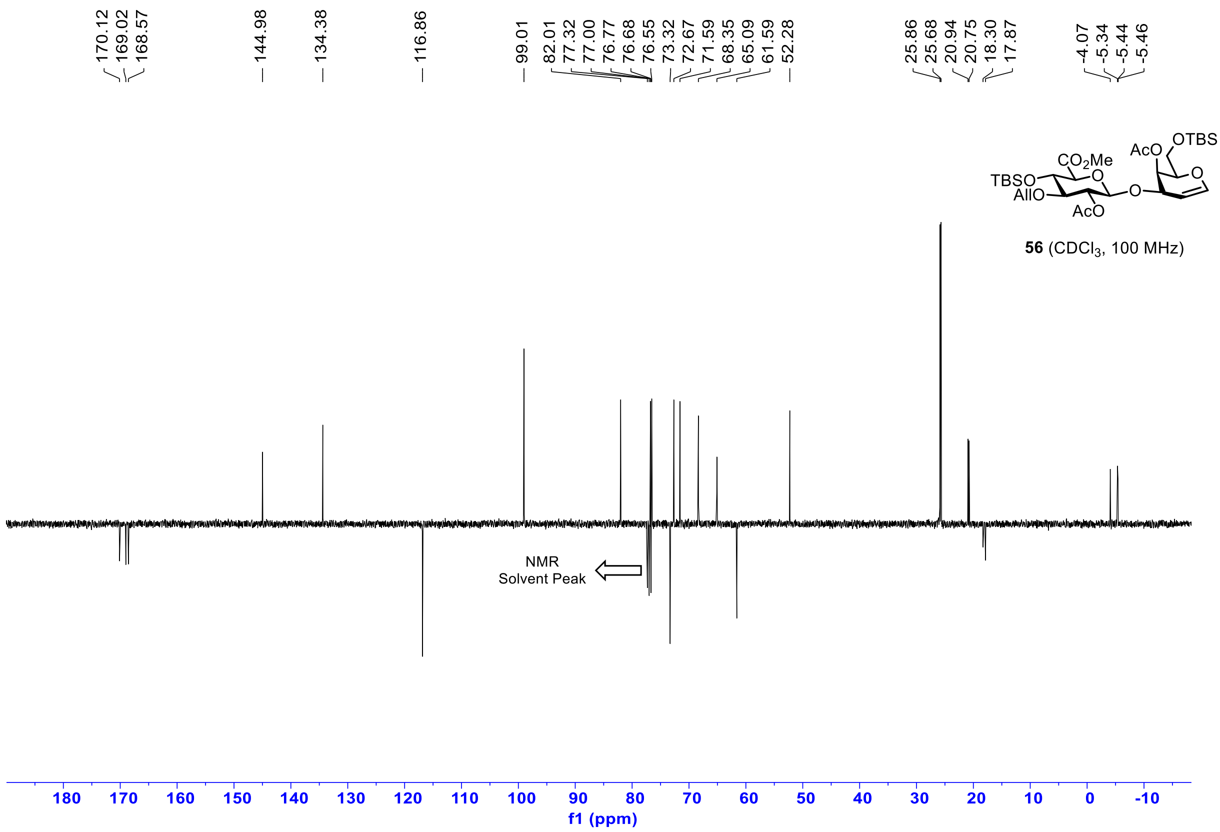

56 ($CDCl_3$, 100 MHz)

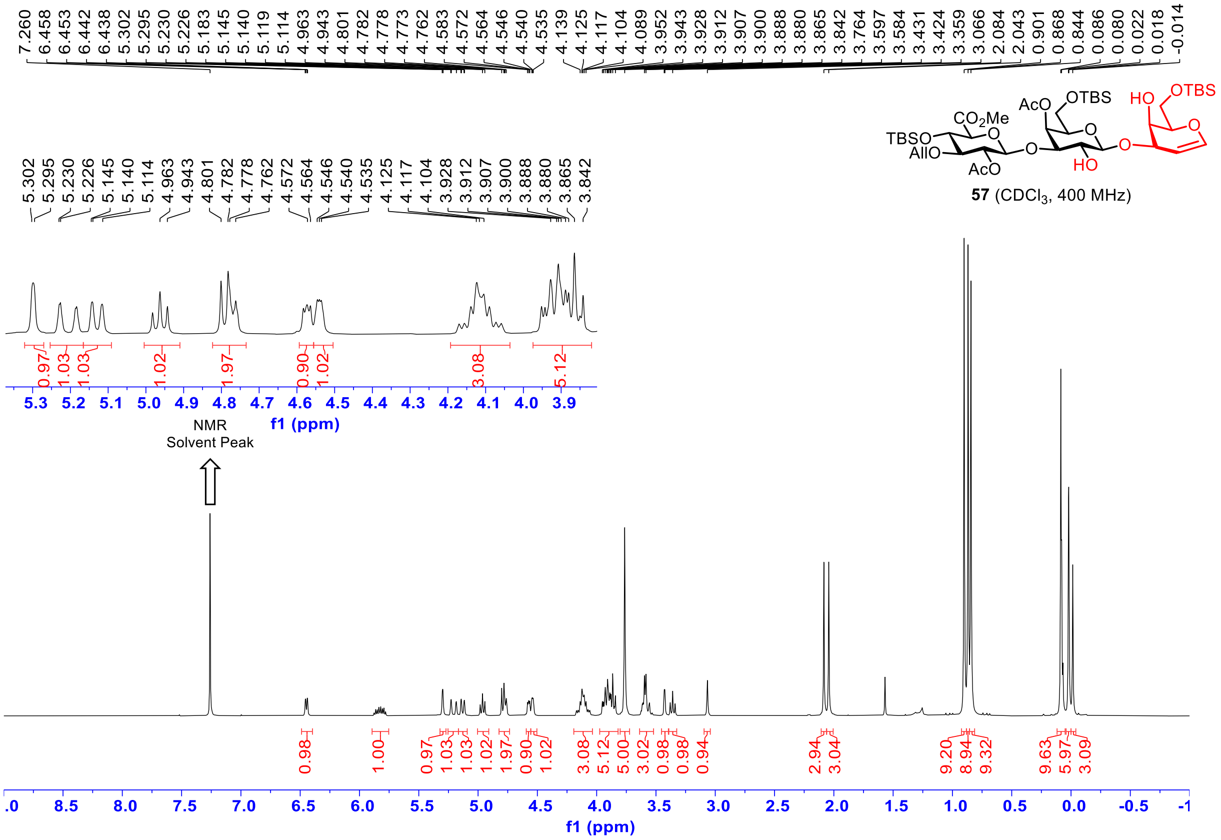
7.260
6.458
6.453
6.442
6.438
5.302
5.295
5.230
5.226
5.183
5.145
5.140
5.119
5.114
4.963
4.943
4.801
4.782
4.778
4.773
4.762
4.583
4.572
4.564
4.546
4.540
4.535
4.139
4.125
4.117
4.104
4.089
3.952
3.943
3.928
3.912
3.907
3.900
3.888
3.880
3.865
3.842
3.764
3.597
3.584
3.431
3.424
3.359
3.066
2.084
2.043
0.901
0.868
0.844
0.086
0.080
0.022
0.018
-0.014
TBSO
AllO
CO2Me
O
AcO
AcO
OTBS
O
O
HO
HO
OTBS
O
O
57 (CDCl3, 400 MHz)
NMR
Solvent Peak
f1 (ppm)
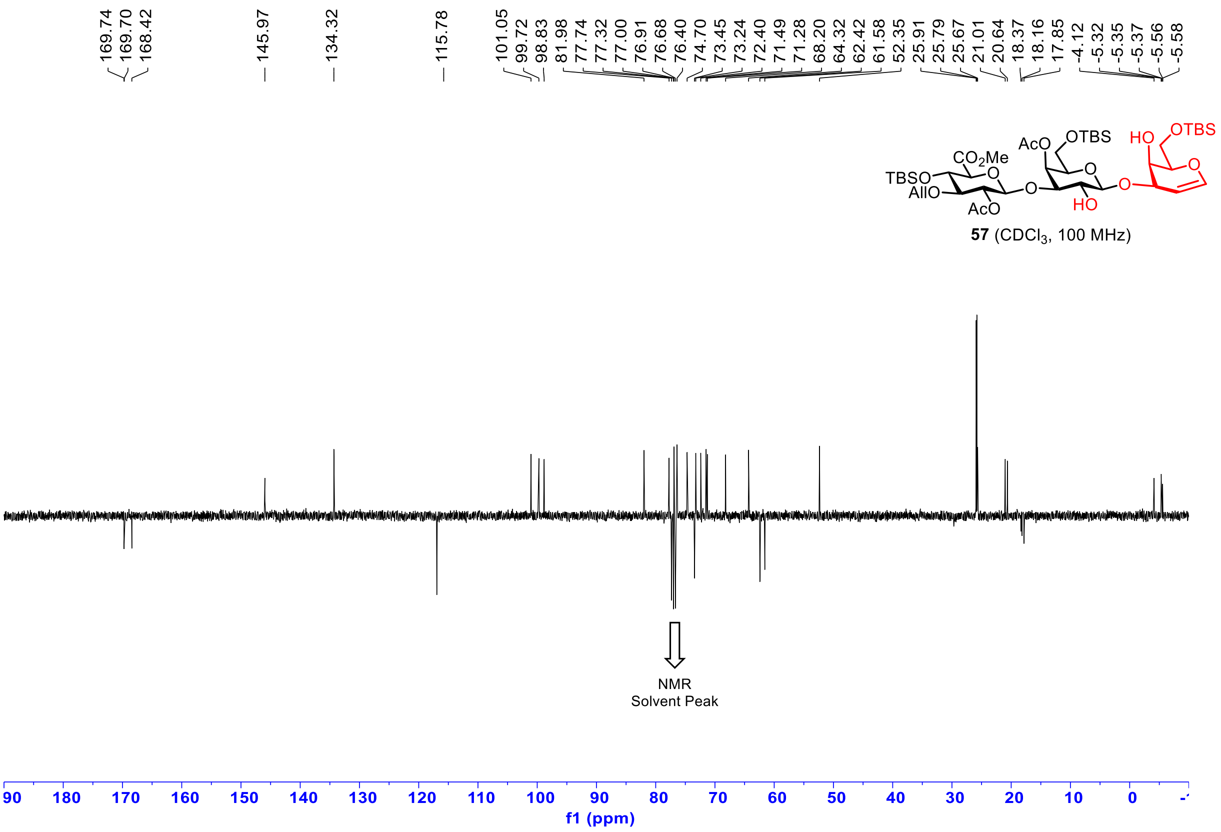
169.74
169.70
168.42
145.97
134.32
115.78
101.05
99.72
98.83
81.98
77.74
77.32
77.00
76.91
76.68
76.40
74.70
73.45
73.24
72.40
71.49
71.28
68.20
64.32
62.42
61.58
52.35
25.91
25.79
25.67
21.01
20.64
18.37
18.16
17.85
-4.12
-5.32
-5.35
-5.37
-5.56
-5.58
57 (CDCl3, 100 MHz)
NMR
Solvent Peak
f1 (ppm)

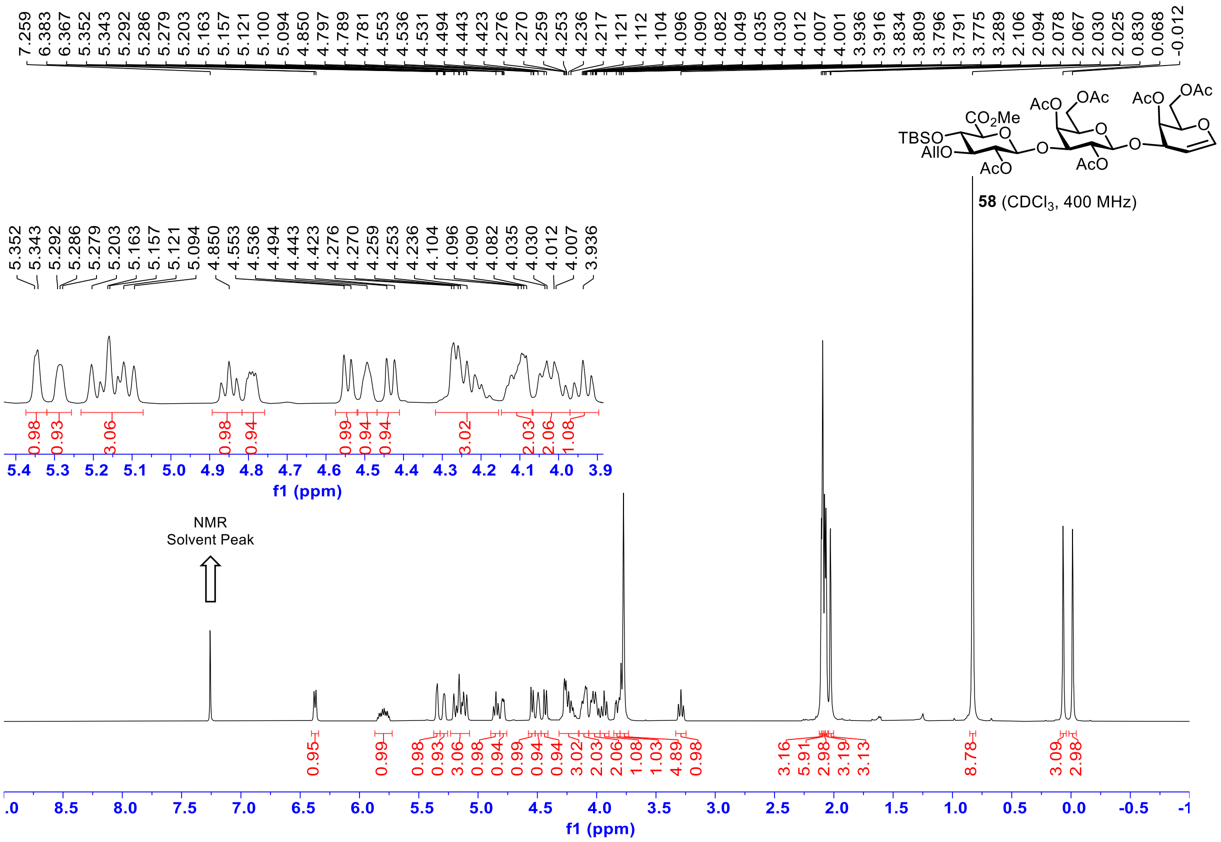
7.259 6.383 6.367 5.352 5.343 5.292 5.286 5.279 5.203 5.163 5.157 5.121 5.100 5.094 4.850 4.797 4.789 4.781 4.553 4.536 4.531 4.494 4.443 4.423 4.276 4.270 4.259 4.253 4.236 4.217 4.121 4.112 4.104 4.096 4.090 4.082 4.049 4.035 4.030 4.012 4.007 4.001 3.936 3.916 3.834 3.809 3.796 3.791 3.775 3.289 2.106 2.094 2.078 2.067 2.030 2.025 0.830 0.068 -0.012
TBSO
AllO
CO2Me
AcO
OAc
AcO
AcO
AcO
OAc
58 (CDCl3, 400 MHz)
5.352 5.343 5.292 5.286 5.279 5.203 5.163 5.157 5.121 5.094 4.850 4.553 4.536 4.494 4.443 4.423 4.276 4.270 4.259 4.253 4.236 4.104 4.096 4.090 4.082 4.035 4.030 4.012 4.007 3.936
0.98 0.93 3.06 0.98 0.94 0.99 0.94 0.94 3.02 2.03 2.06 1.08
f1 (ppm)
NMR
Solvent Peak
0.95 0.99 0.98 0.93 3.06 0.98 0.94 0.99 0.94 0.94 3.02 2.03 2.06 1.08 1.03 4.89 0.98 3.16 5.91 2.98 3.19 3.13 8.78 3.09 2.98
f1 (ppm)

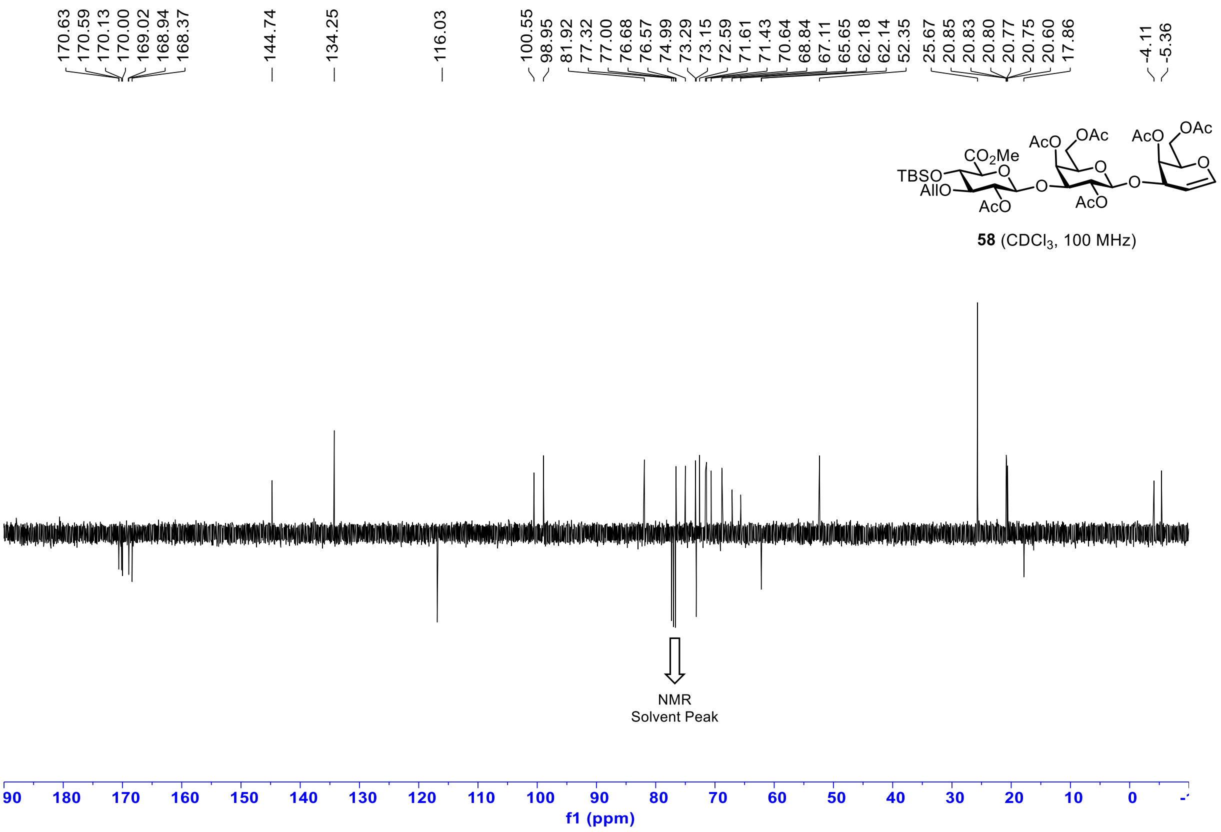
170.63 170.59 170.13 170.00 169.02 168.94 168.37 144.74 134.25 116.03 100.55 98.95 81.92 77.32 77.00 76.68 76.57 74.99 73.29 73.15 72.59 71.61 71.43 70.64 68.84 67.11 65.65 62.18 62.14 52.35 25.67 20.85 20.83 20.80 20.77 20.75 20.60 17.86 -4.11 -5.36
TBSO
AllO
CO2Me
AcO
OAc
AcO
AcO
AcO
OAc
58 (CDCl3, 100 MHz)
NMR
Solvent Peak
f1 (ppm)

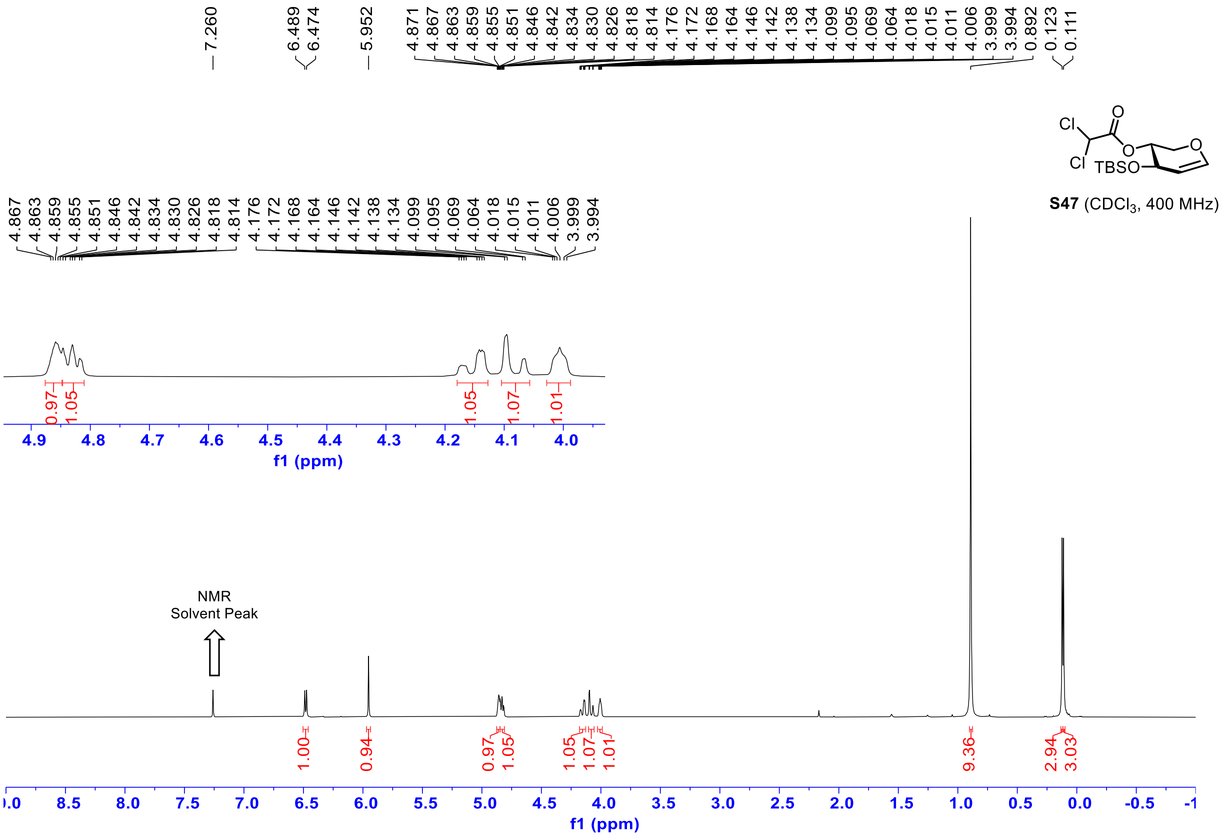

S47 (CDCl3, 400 MHz)
NMR Solvent Peak
f1 (ppm)

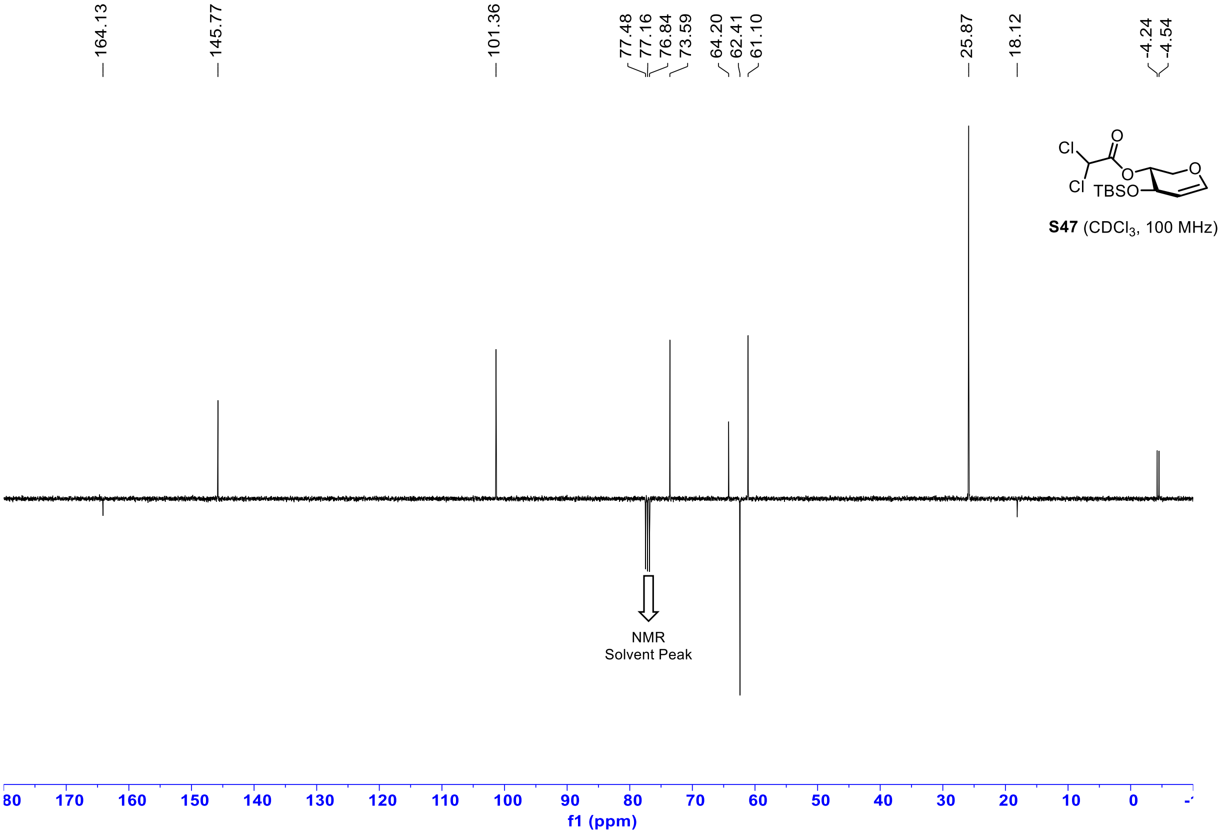

S47 (CDCl3, 100 MHz)
NMR Solvent Peak
f1 (ppm)

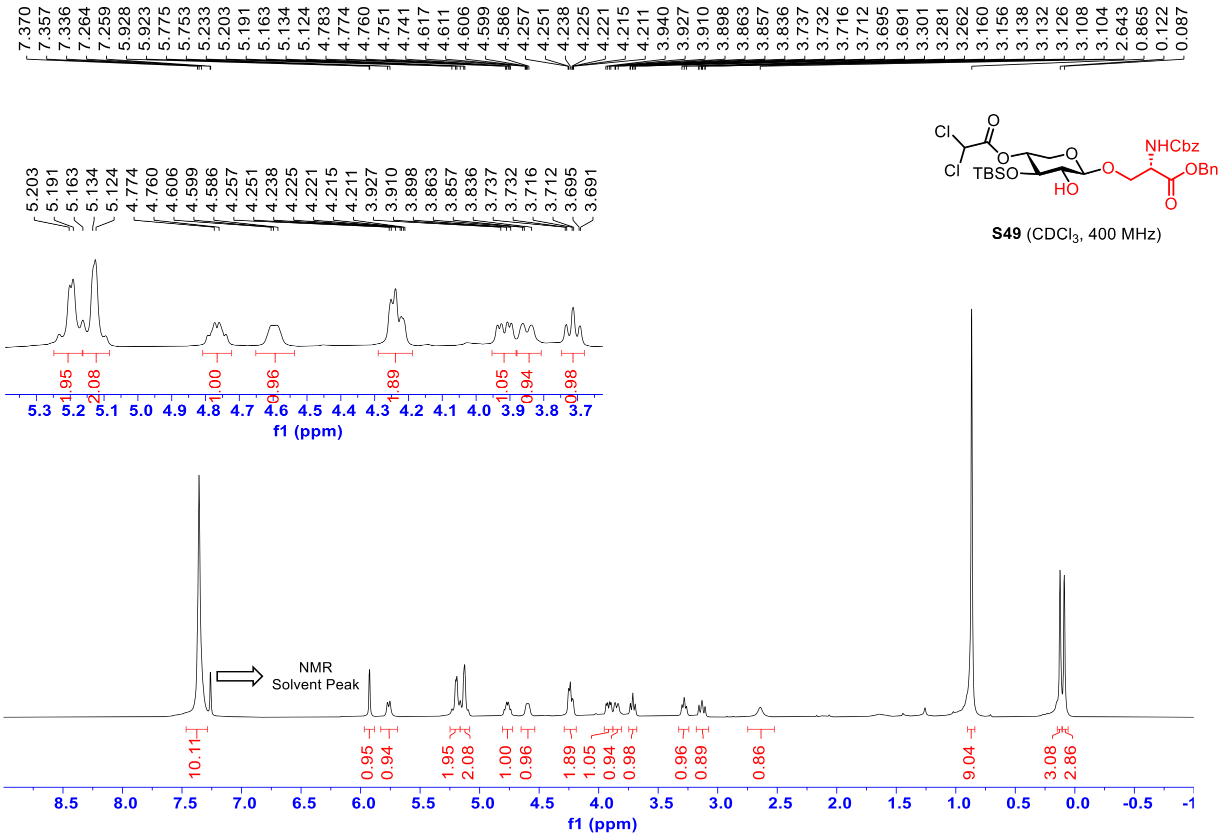
S49 (CDCl3, 400 MHz)
NMR
Solvent Peak
f1 (ppm)

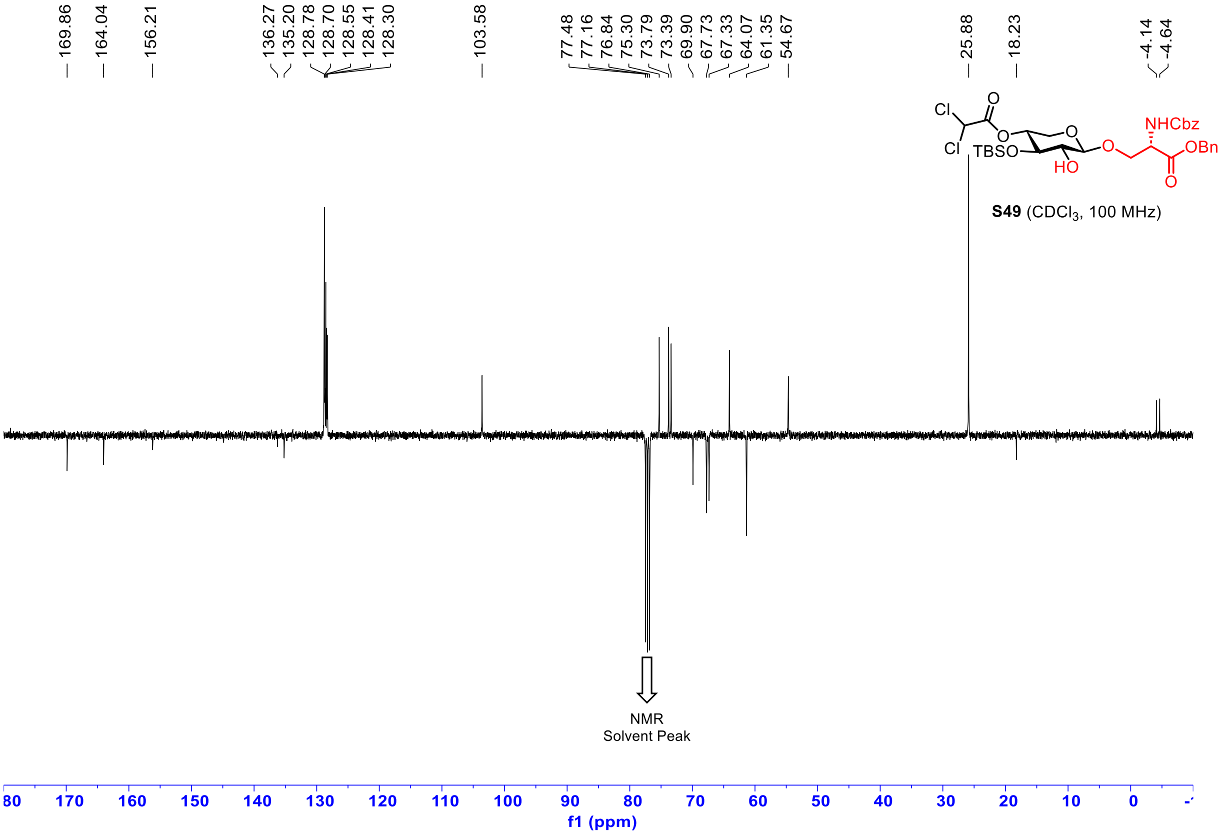
S49 (CDCl3, 100 MHz)
NMR
Solvent Peak
f1 (ppm)

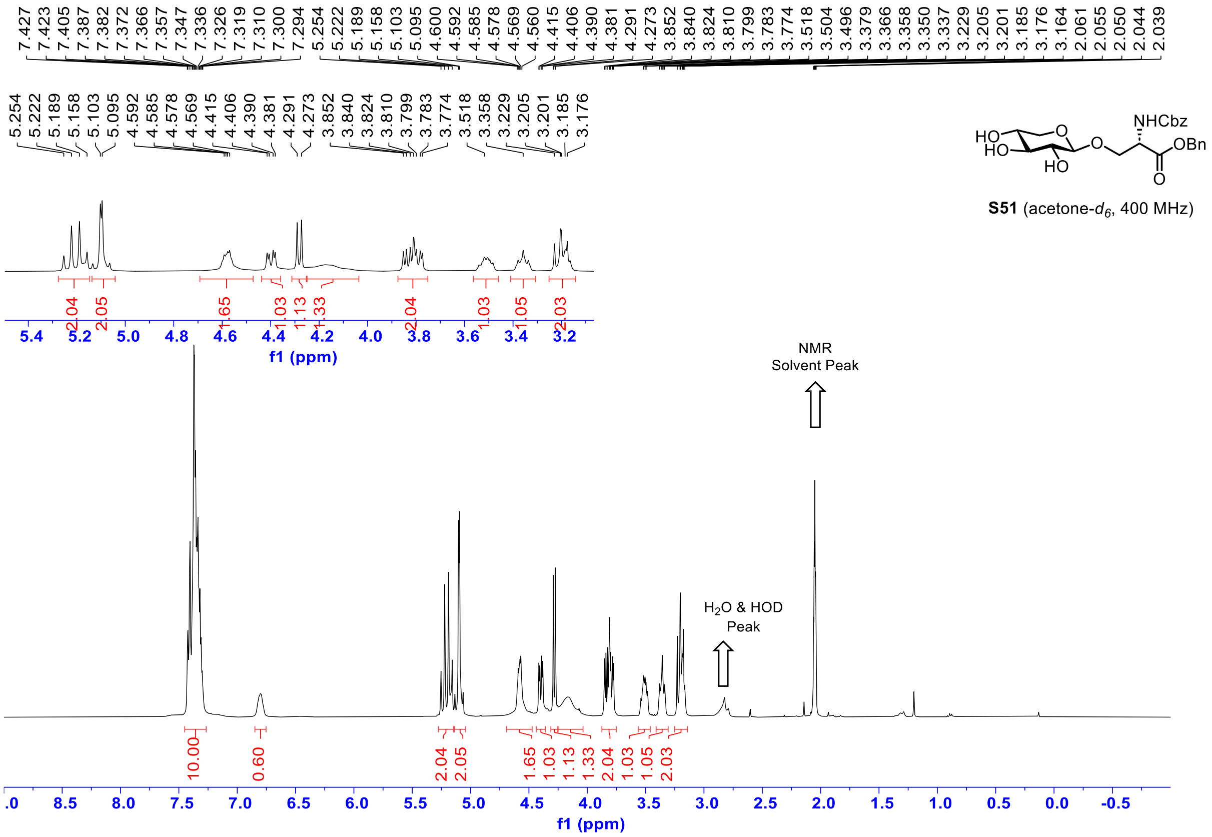

S51 (acetone-d_6, 400 MHz)

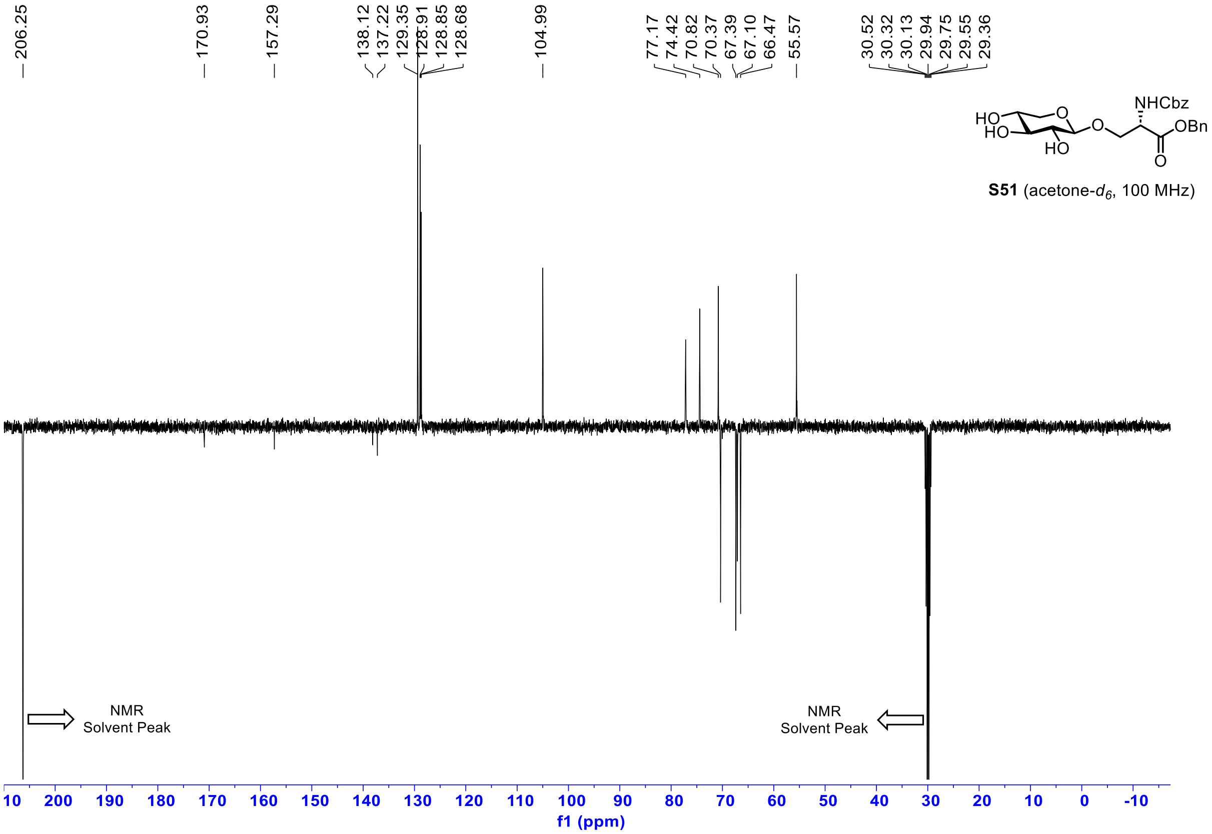

S51 (acetone-d_6, 100 MHz)

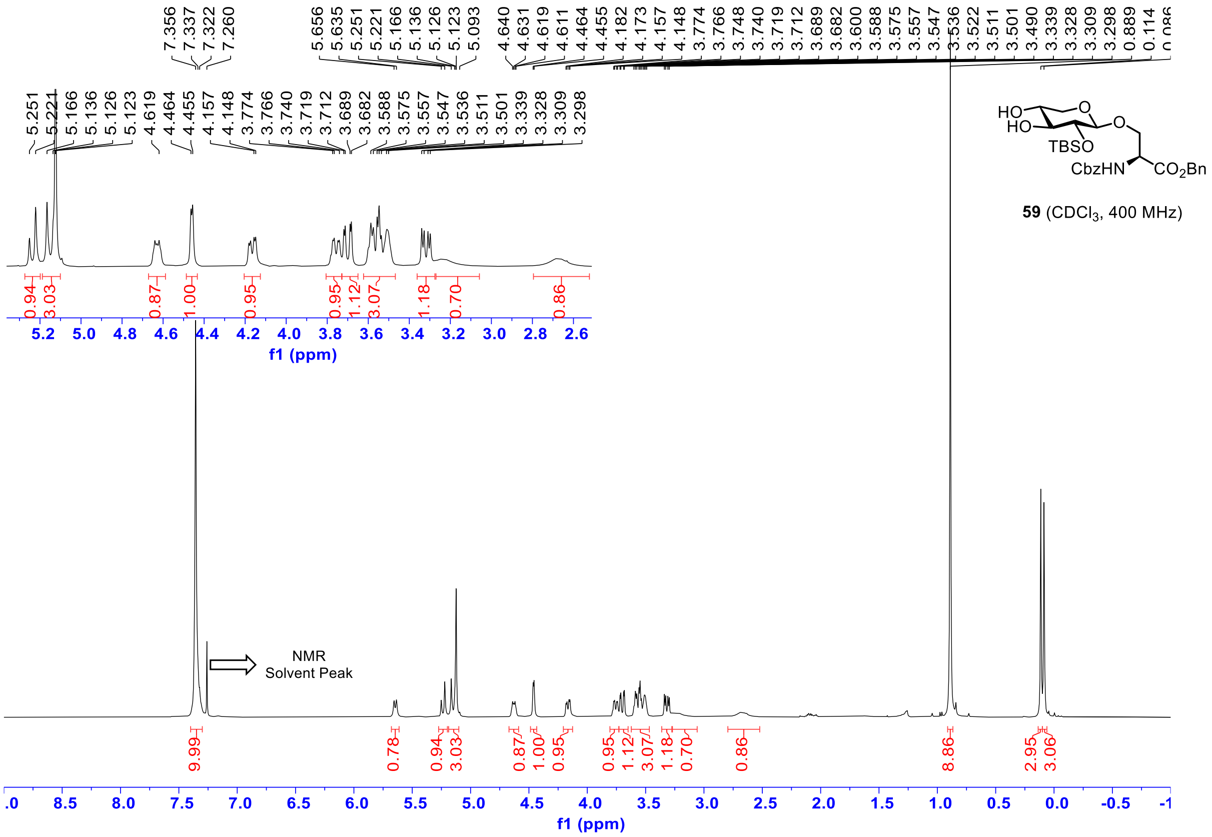
59 (CDCl3, 400 MHz)
NMR
Solvent Peak
f1 (ppm)

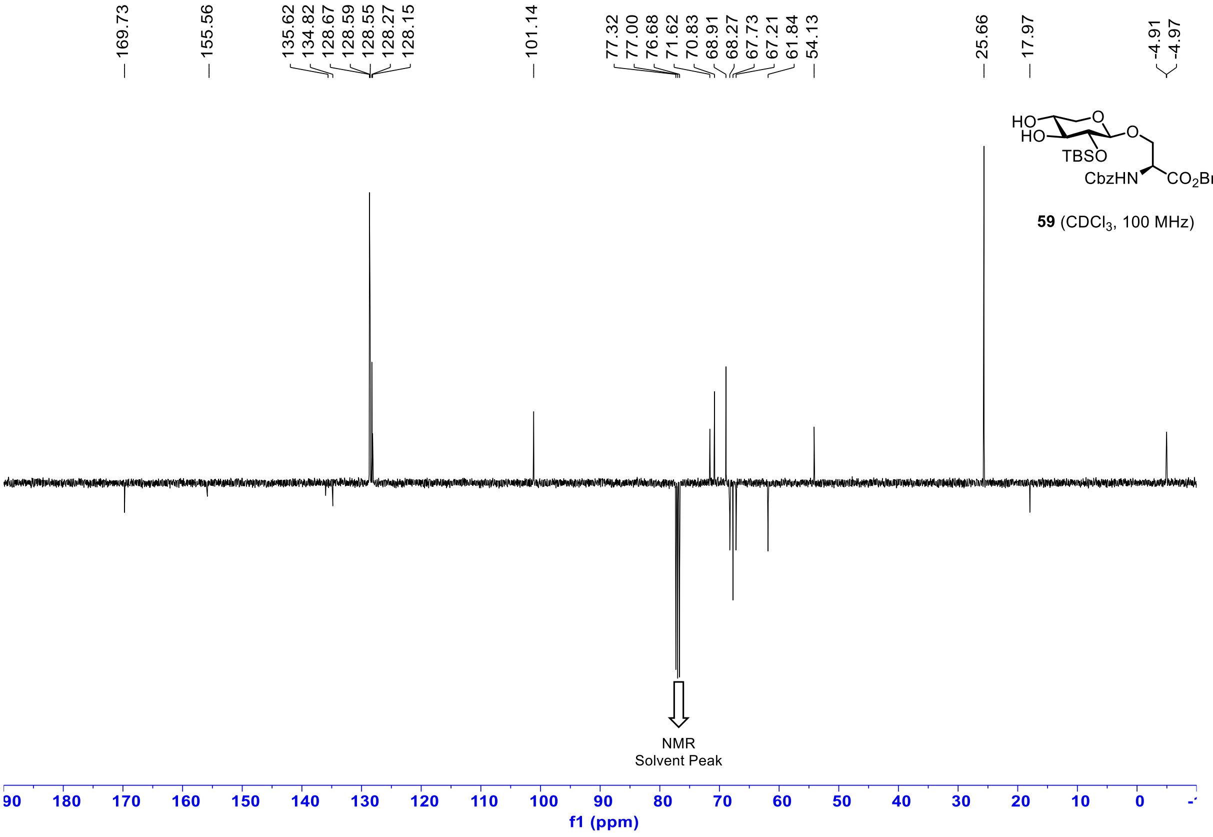
59 (CDCl3, 100 MHz)
169.73
155.56
135.62
134.82
128.67
128.59
128.55
128.27
128.15
101.14
77.32
77.00
76.68
71.62
70.83
68.91
68.27
67.73
67.21
61.84
54.13
25.66
17.97
-4.91
-4.97
NMR
Solvent Peak
f1 (ppm)

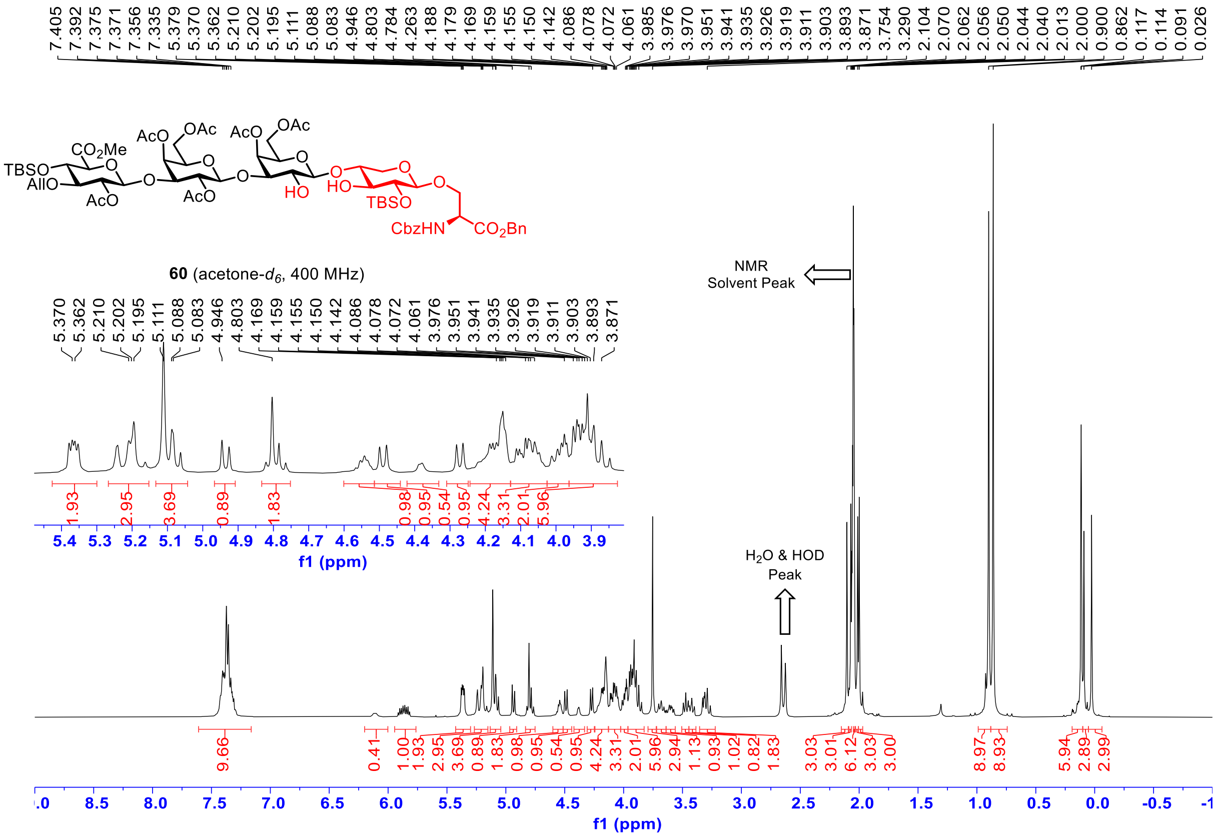
7.405 7.392 7.375 7.371 7.356 7.335 5.379 5.370 5.362 5.210 5.202 5.195 5.111 5.088 5.083 4.946 4.803 4.784 4.263 4.188 4.179 4.169 4.159 4.155 4.150 4.142 4.086 4.078 4.072 4.061 3.985 3.976 3.970 3.951 3.941 3.935 3.926 3.919 3.911 3.903 3.893 3.871 3.754 3.290 2.104 2.070 2.062 2.056 2.050 2.044 2.040 2.013 2.000 0.900 0.862 0.117 0.114 0.091 0.026
TBSO
AllO
CO_2Me
AcO
OAc
HO
TBSO
CbzHN
CO_2Bn
60 (acetone-d_6, 400 MHz)
5.370 5.362 5.210 5.202 5.195 5.111 5.088 5.083 4.946 4.803 4.169 4.159 4.155 4.150 4.142 4.086 4.078 4.072 4.061 3.976 3.951 3.941 3.935 3.926 3.919 3.911 3.903 3.893 3.871
NMR Solvent Peak
H_2O & HOD Peak
f1 (ppm)

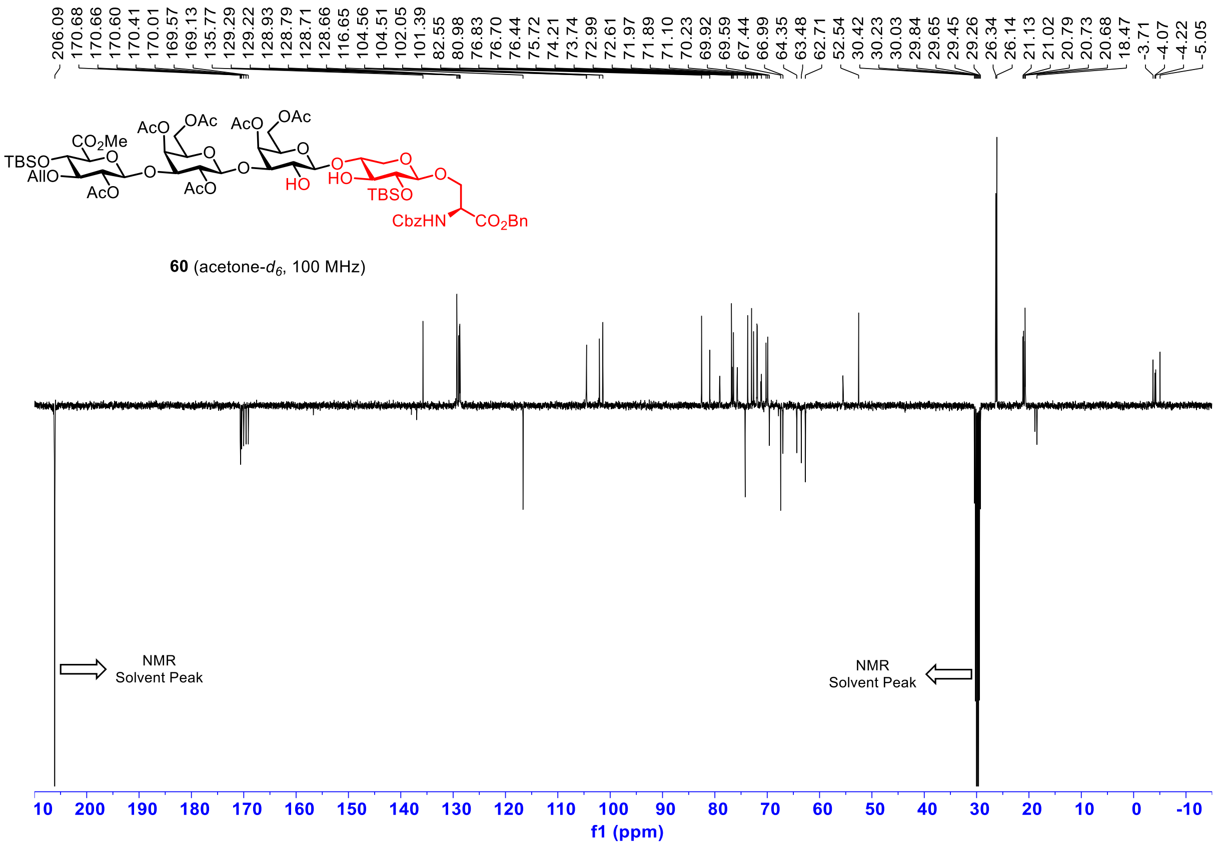
206.09 170.68 170.66 170.60 170.41 170.01 169.57 169.13 135.77 129.29 129.22 128.93 128.79 128.71 128.66 116.65 104.56 104.51 102.05 101.39 82.55 80.98 76.83 76.70 76.44 75.72 74.21 73.74 72.99 72.61 71.97 71.89 71.10 70.23 69.92 69.59 67.44 66.99 64.35 63.48 62.71 52.54 30.42 30.23 30.03 29.84 29.65 29.45 29.26 26.34 26.14 21.13 21.02 20.79 20.73 20.68 18.47 -3.71 -4.07 -4.22 -5.05
TBSO
AllO
CO_2Me
AcO
OAc
HO
TBSO
CbzHN
CO_2Bn
60 (acetone-d_6, 100 MHz)
NMR Solvent Peak
f1 (ppm)

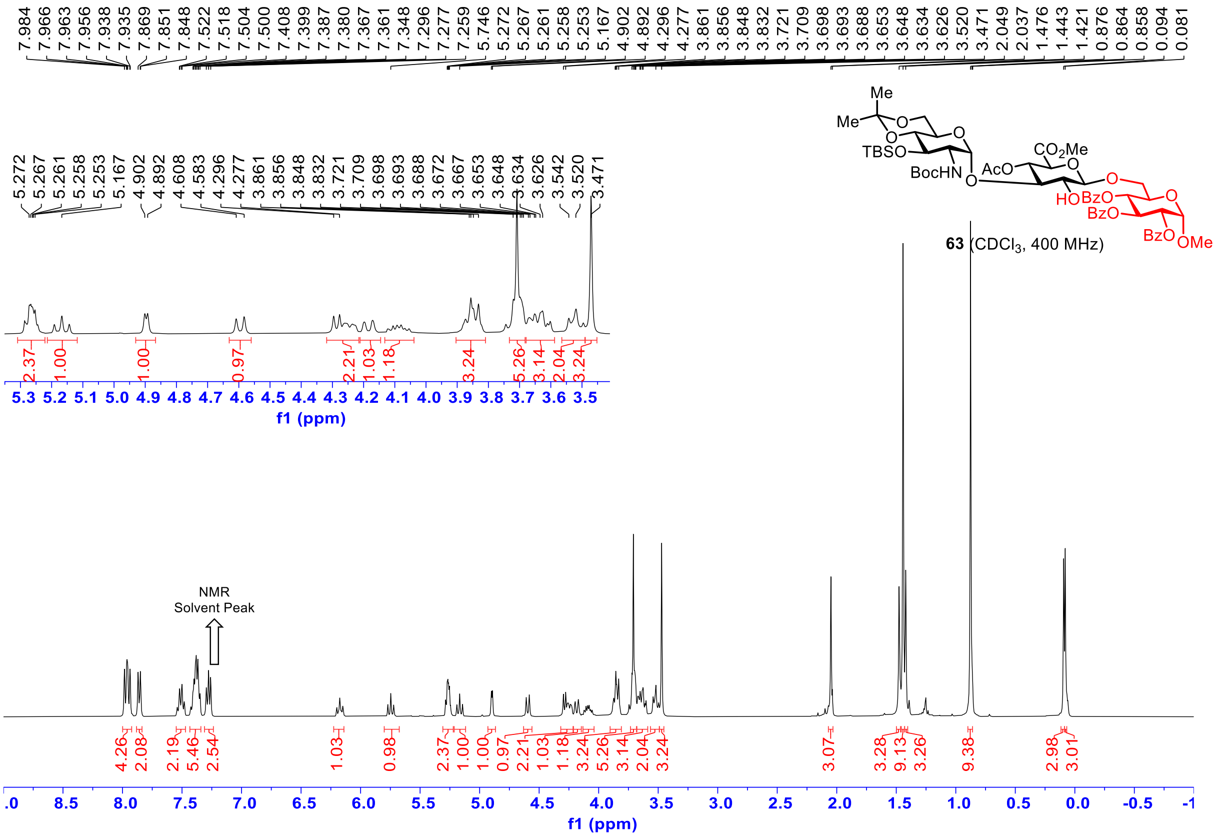
7.984 7.966 7.963 7.956 7.938 7.935 7.869 7.851 7.848 7.522 7.518 7.504 7.500 7.408 7.399 7.387 7.380 7.367 7.361 7.348 7.296 7.277 7.259 5.746 5.272 5.267 5.261 5.258 5.253 5.167 4.902 4.892 4.296 4.277 3.861 3.856 3.848 3.832 3.721 3.709 3.698 3.693 3.688 3.653 3.648 3.634 3.626 3.520 3.471 2.049 2.037 1.476 1.443 1.421 0.876 0.864 0.858 0.094 0.081
5.272 5.267 5.261 5.258 5.253 5.167 4.902 4.892 4.608 4.583 4.296 4.277 3.861 3.856 3.848 3.832 3.721 3.709 3.698 3.693 3.688 3.672 3.667 3.653 3.648 3.634 3.626 3.542 3.520 3.471
f1 (ppm)
63 (CDCl3, 400 MHz)
NMR Solvent Peak
f1 (ppm)

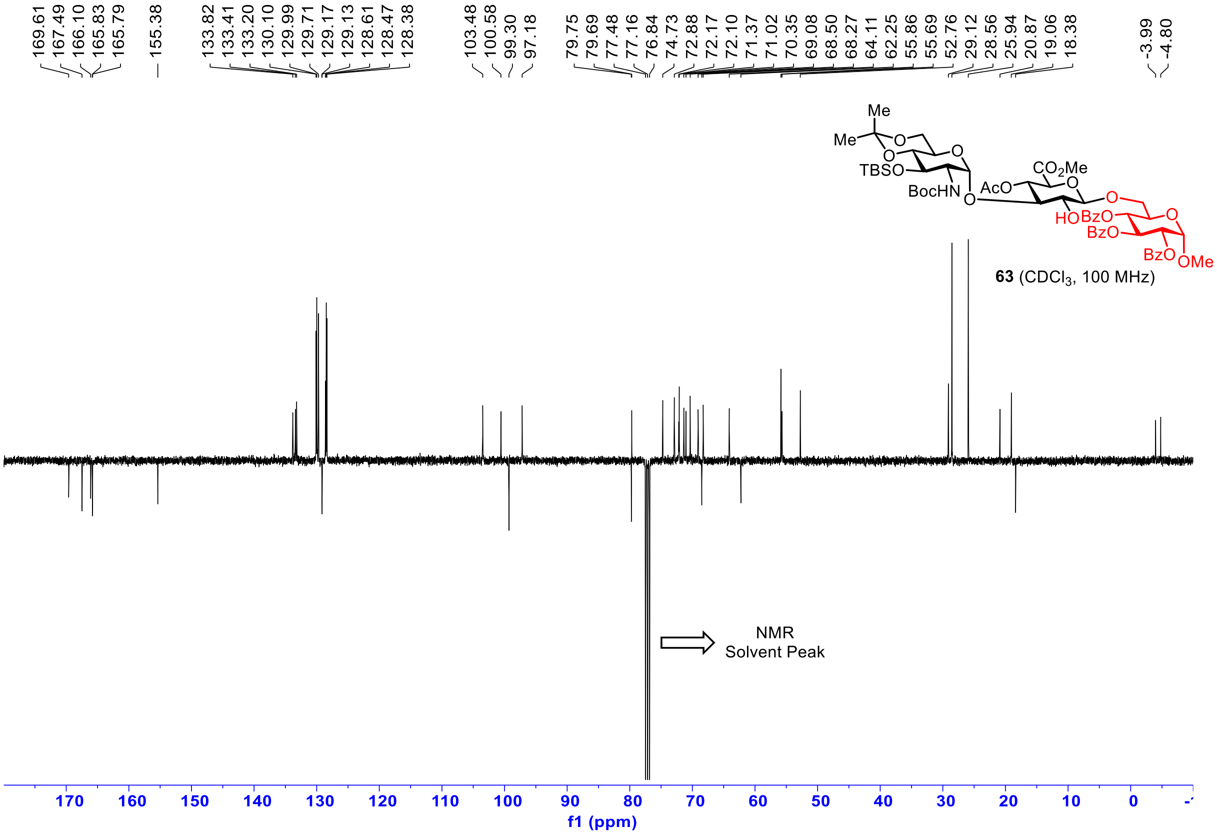
169.61 167.49 166.10 165.83 165.79 155.38 133.82 133.41 133.20 130.10 129.99 129.71 129.17 129.13 128.61 128.47 128.38 103.48 100.58 99.30 97.18 79.75 79.69 77.48 77.16 76.84 74.73 72.88 72.17 72.10 71.37 71.02 70.35 69.08 68.50 68.27 64.11 62.25 55.86 55.69 52.76 29.12 28.56 25.94 20.87 19.06 18.38 -3.99 -4.80
63 (CDCl3, 100 MHz)
NMR Solvent Peak
f1 (ppm)

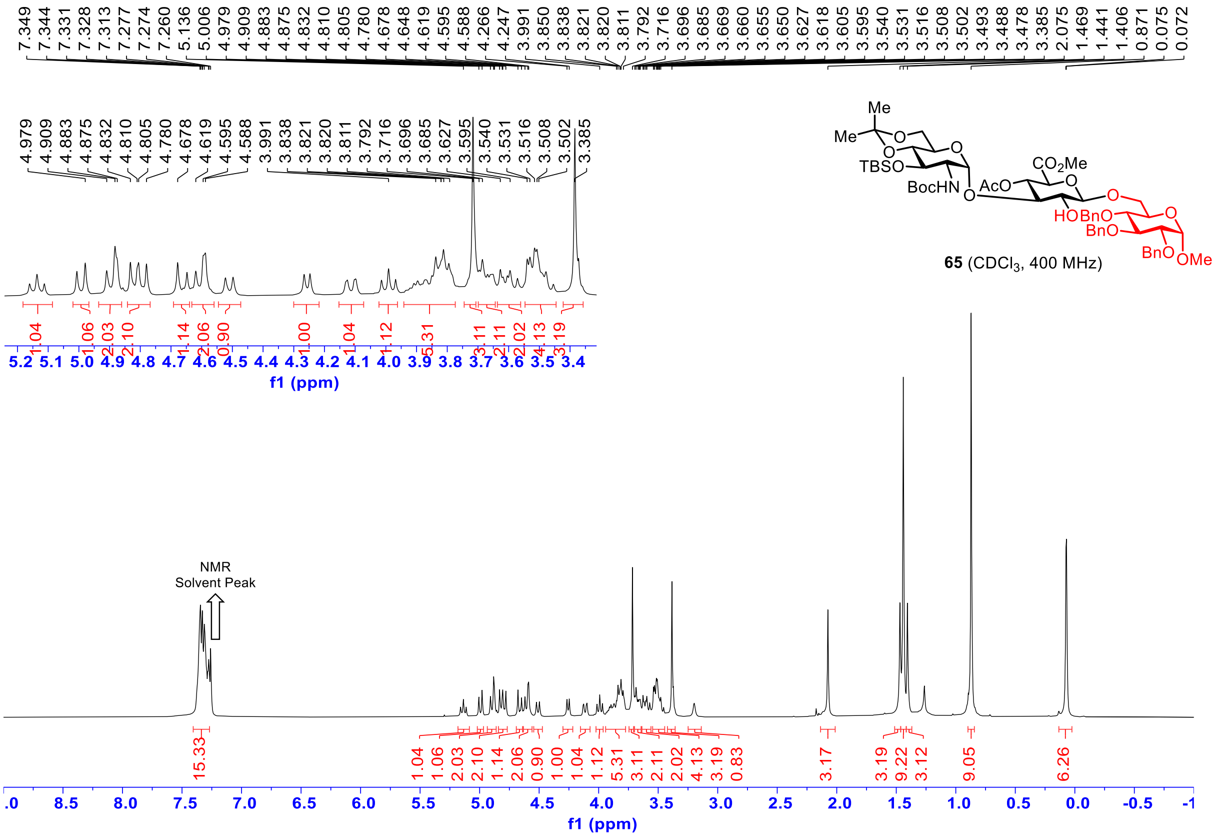
7.349 7.344 7.331 7.328 7.313 7.277 7.274 7.260 5.136 5.006 4.979 4.909 4.883 4.875 4.832 4.810 4.805 4.780 4.678 4.648 4.619 4.595 4.588 4.266 4.247 3.991 3.850 3.838 3.821 3.820 3.811 3.792 3.716 3.696 3.685 3.669 3.660 3.655 3.650 3.627 3.618 3.605 3.595 3.540 3.531 3.516 3.508 3.502 3.493 3.488 3.478 3.385 2.075 1.469 1.441 1.406 0.871 0.075 0.072
65 (CDCl3, 400 MHz)
NMR Solvent Peak
f1 (ppm)

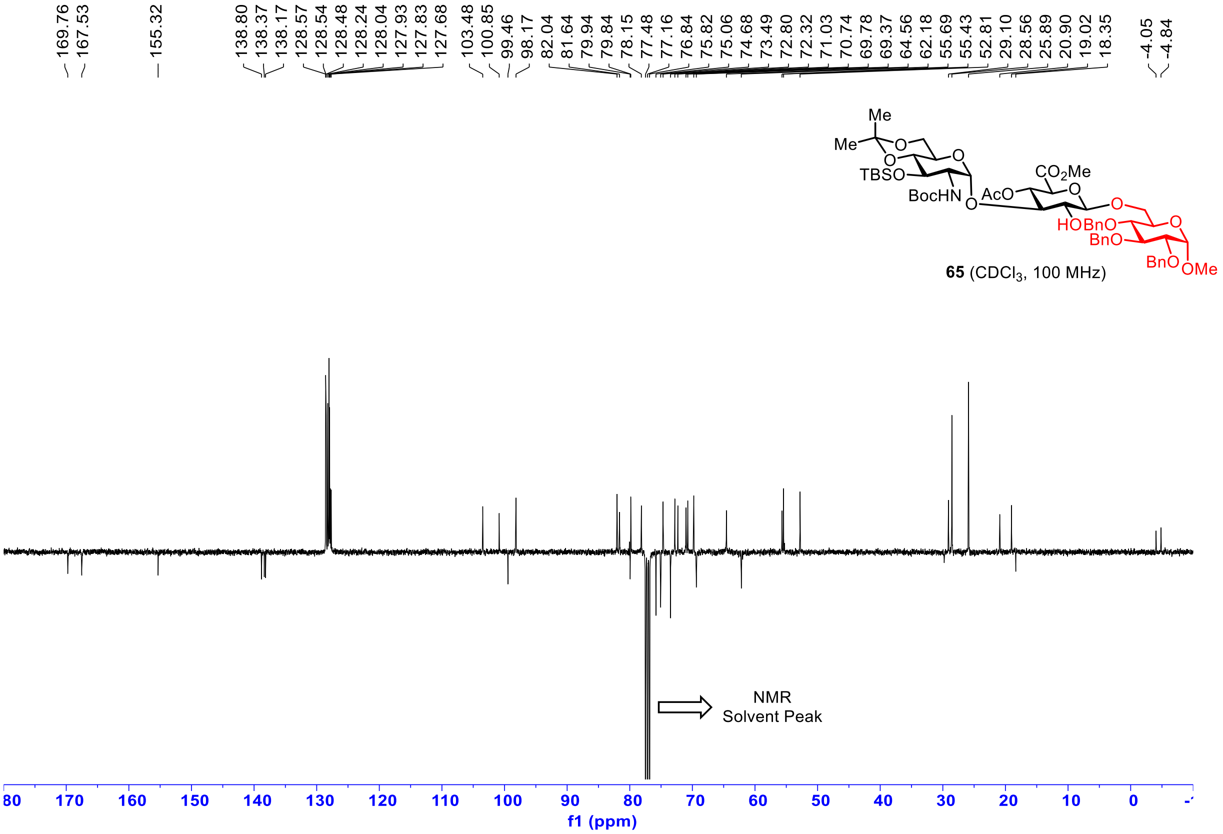
169.76 167.53 155.32 138.80 138.37 138.17 128.57 128.54 128.48 128.24 128.04 127.93 127.83 127.68 103.48 100.85 99.46 98.17 82.04 81.64 79.94 79.84 78.15 77.48 77.16 76.84 75.82 75.06 74.68 73.49 72.80 72.32 71.03 70.74 69.78 69.37 64.56 62.18 55.69 55.43 52.81 29.10 28.56 25.89 20.90 19.02 18.35 -4.05 -4.84
65 (CDCl3, 100 MHz)
NMR Solvent Peak
f1 (ppm)
